# C–C Bond Formation via Direct Functionalization of Indolizines with a Bichromophoric Ruthenium Photocatalyst

**DOI:** 10.1021/acsorginorgau.5c00110

**Published:** 2026-02-07

**Authors:** Kevin Klaus Stefanoni, René Wilhelm

**Affiliations:** Institute of Organic Chemistry, 26534 Clausthal University of Technology, Leibnizstr. 6, Clausthal-Zellerfeld 38678, Germany

**Keywords:** photoredox, indolizines, alkylation, C−C bond, catalyst design

## Abstract

An unprecedented photocatalyzed radical C­(sp^2^)–C­(sp^3^) alkylation protocol to prepare a range of substituted 3-alkylated indolizine derivatives, mediated by 2-mercaptothiazolidinium salts as radical sources and a new dyad-like Ruthenium complex as a photoredox catalyst, under green light irradiation, resulted in yields of up to 99%. The mild, robust, and chemoselective procedure employs inexpensive, air-insensitive, and readily accessible reagents, enabling convenient synthesis of the substituted indolizines. Moreover, different *N*-heteroarenes, such as 1*H*-indoles and 2*H*-indazoles, were successfully alkylated under the optimized conditions. The resulting alkylated products are scaffolds with significance for drug design in medicinal chemistry.

## Graphical abstract

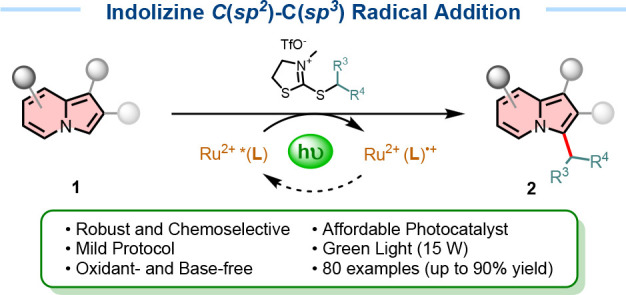

## Introduction

Indolizines are valuable nitrogen-containing heterocycles that own important biologically relevant properties, being indole bioisosteres.^1–3^ Over the last decades, various groups have been working on indolizine synthesis and also on their late-stage functionalization. The latter has been enabled by the advent of novel technologies and the introduction of milder synthetic protocols.^4–8^ However, because their biological potential is still largely unexplored,^9^ efforts need to be made to further investigate their pharmacological activities and develop more versatile methods to expand their applications in medicinal chemistry and materials science.

The formation of carbon–carbon (C–C) bonds has gained increasing importance in organic synthesis, as it allows the editing and building of the carbon backbone of every organic compound.^10^ Over the last decades, well-established methods such as cross-coupling reactions have been extensively employed, being considered one of the most straightforward approaches to gaining molecular complexity from simple building blocks. Specifically, preactivated electrophiles such as aryl-, vinyl-, or alkyl (pseudo)­halides are coupled with organometallic nucleophiles in an elegant and economical way. The direct C–H bond functionalization strategy offers notable advantages, such as shortening a synthetic route and reducing wasteful byproducts, compared to more traditional methods. Beyond simple academic curiosity, their practical potential is increasingly recognized for industrial applications. However, one of the main drawbacks is the need for substrate prefunctionalization, which often adds additional steps to a synthetic route.^11^ Therefore, the discovery of novel protocols for the direct coupling of nonfunctionalized starting materials such as heterocycles or arenes is of high importance.^12^

With the introduction of photoredox catalysis, it became possible to overcome that limit, and a door to further developments was opened.^13–16^ Under very mild conditions, photoredox catalysts enable different regioselectivities, and improve functional group tolerance, and expand the scope and versatility of C­(sp^2^)–H functionalization strategies. Within the past few years, chemists have investigated alkyl radical precursors for C–C bond formation (Figure 1). Despite their broad applicability, some of them are associated with certain issues. For instance, Katritzky salts^17^ are prepared from expensive and moisture-sensitive 2,4,6-triphenylpyrylium tetrafluoroborate, while alkyl-substituted dihydropyridines (DHPs)^18^ must be synthesized from expensive alkyl aldehydes, often resulting in unsatisfying yields. Conversely, 2-mercaptoimidazolium^19^ or thiazolidinium^20,21^ salts have recently emerged as inexpensive and bench-stable alternatives for the generation of alkyl radicals under mild photoredox conditions.

**Figure 1:**
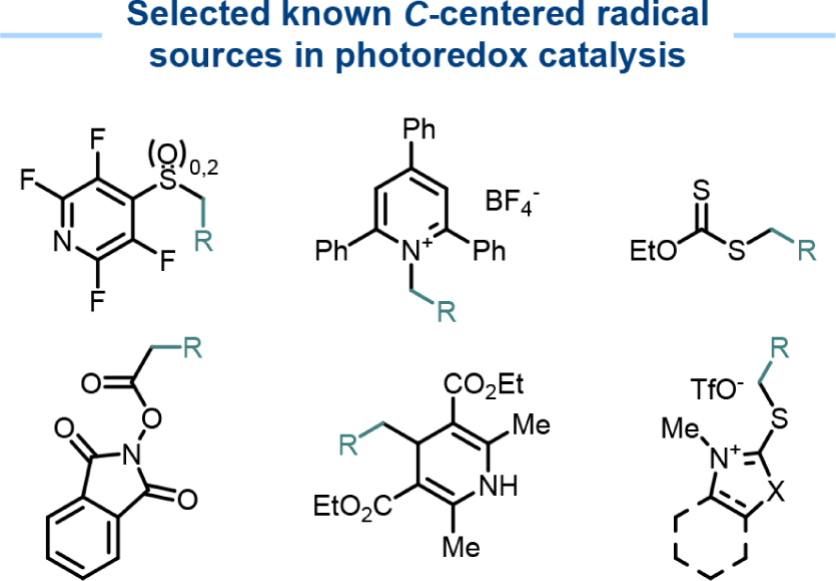
Selected known carbon-centered radical precursors for photoredox SET processes.

Pioneering work was conducted by Zemtsov et al.^22^ in 2019, in which silyl enol ethers were efficiently alkylated by employing 2-mercaptothiazolidinium salts and an iridium photocatalyst (Scheme 1a). Yields of the desired products were generally satisfactory, although in some cases 20 mol % of [Ir­(dtbbpy)­(ppy)_2_]­PF_6_ was required, and a basic scavenger was needed to trap the silyl protecting group and generate the alkylated ketone. More recently, Tian et al.^200^ in 2024 and Zhu et al.^201^ in 2025 explored the reactivity of 2-mercaptothiazolidinium salts toward the synthesis of alkylated isoquinolines and indolo­[2,1-*a*]­isoquinolines, respectively (Scheme 1b–c). In both cases, the carbon-centered radicals formed in the process were coupled with electron-poor sp^2^ systems in a Minisci-or Giese-type fashion. The protocols were general, and no further additives were needed to achieve good yields; however, Ir-photocatalysis was still required, and a powerful blue LED setup (30 W) was employed.

**Scheme 1:**
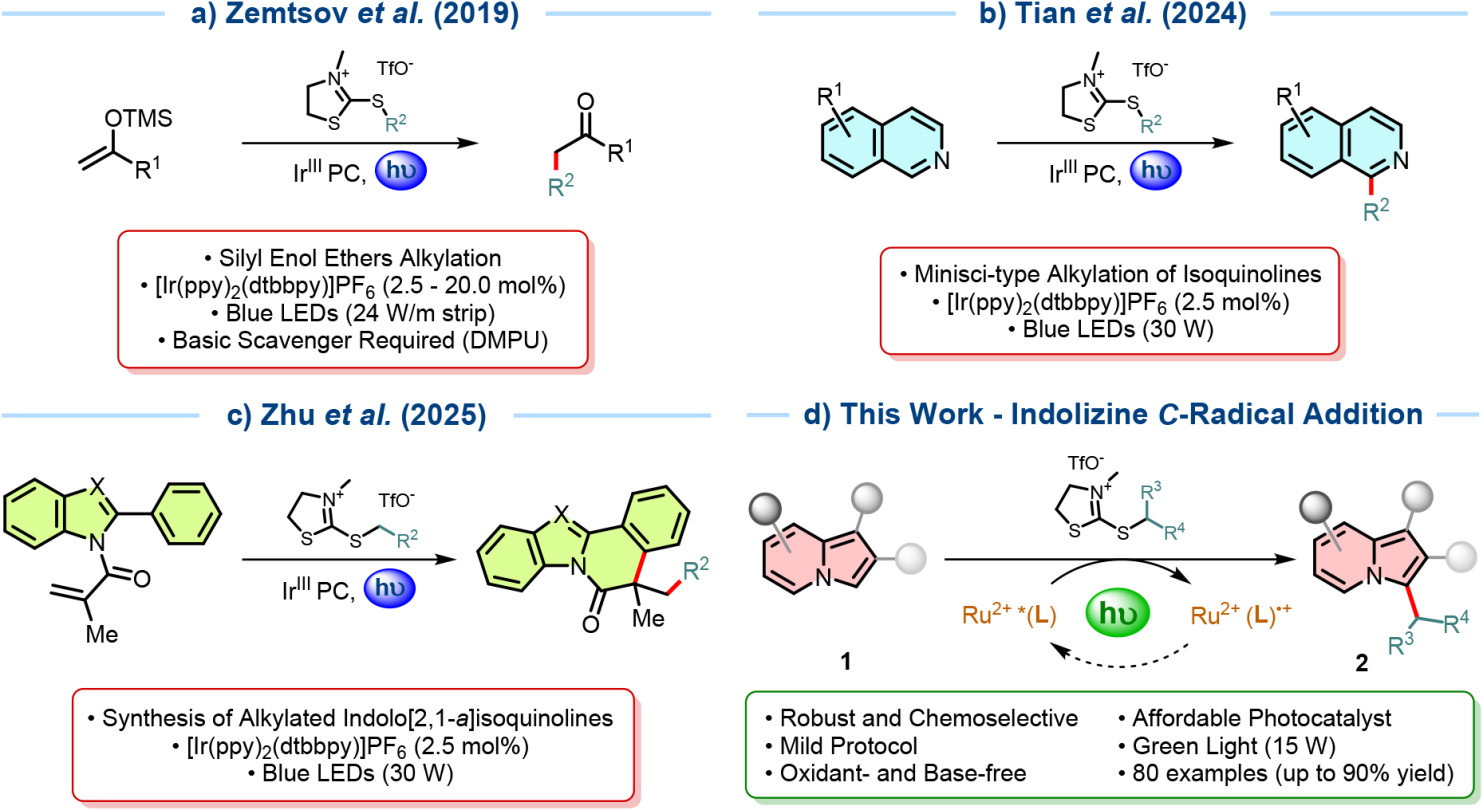
2-Mercaptothiazolidinium Salts as Alkyl Radical Sources

As part of our continuous effort to develop effective photocatalysts^23–26^ and methods for the direct functionalization of heterocycles,^27–29^ we wanted to apply such a system for the direct alkylation of electron-rich heteroaromatic compounds, such as indolizines. Herein, we present a photocatalyzed radical C­(sp^2^)–C­(sp^3^) alkylation protocol to prepare a range of substituted 3-alkylated indolizine derivatives **2**, mediated by 2-mercaptothiazolidinium salts as radical sources in *N*,*N*-dimethylacetamide (DMA) and a new dyad-like complex [Ru­(dpp)_2_(dMeODPAT-Tz-bpy)]­(PF_6_)_2_ (**PC2**) as the photoredox catalyst, under green light irradiation, with up to 90% yield (Scheme 1d).

## Results and Discussion

2-Phenylindolizine-1-carbonitrile **1a** was chosen as the model substrate for the optimization studies. Unlike our previous works, we found that for the presented reaction, a green LED excites the photocatalyst sufficiently. In general, a longer wavelength is always desirable, since side reactions and subsequent reactions are less likely to be promoted. Upon exploring our established photocatalyst **PC1** under these conditions, we were pleased to observe the formation of the desired alkylated product **2a** with 67% yield. Additionally, a secondary product, the 3,5-dialkylated indolizine **2a′** was formed in 18% yield (entry 1, Table 1). Interested by this outcome, we synthesized a series of related photocatalysts based on **PC1**, in which the triarylamine unit was diversely substituted (entries 2–4, Table 1). Among these, the best result was obtained by using **PC2**, which provided an improved yield of **2a** and a higher selectivity compared to the byproduct **2a′** (entry 2, Table 1). Sterically hindered and electron-rich triarylamines, as in the case of **PC3** and **PC4**, led to a slight switch in the selectivity in favor of the byproduct **2a′** (entries 3–4, Table 1). Common ruthenium-based photocatalysts (e.g., [Ru­(bpy)_3_]­(PF_6_)_2_ and [Ru­(phen)_3_]­(PF_6_)_2_) were not able to efficiently convert substrate **1a** (entries 5–6, Table 1). Only [Ru­(dpp)_3_]­(PF_6_)_2_ was able to fully convert the starting indolizine to product **2a**, albeit in a lower yield compared to **PC2** (entry 7, Table 1). Analogously, strongly reducing Ir­(ppy)_3_ was not efficient in converting indolizine **1a**, as the byproduct **2a′** was produced with 23% yield (entry 8, Table 1). 4-CzIPN, an organic photocatalyst, was not suitable for the reaction, yielding only 30% of the desired product (entry 9, Table 1). In conclusion, the designed **PC2** outperformed commercially available photocatalysts listed in Table 1. No product formation was observed in the control experiment without a photocatalyst, highlighting the importance of the latter for the developed protocol (entry 10, Table 1). Ultimately, **PC2** was used at 0.5 mol %, which was found to be the optimal catalyst loading. The photocatalyst was still visible after the reaction via TLC, but due to the low amount of applied photocatalyst, the latter was not isolated. Optimal radical source loading was kept at 1.5 equiv, as lower loadings did not lead to full conversion of the starting indolizine **1a** (entries 11–12, Table 1). Reducing the reaction time was beneficial, as within 2 h it was possible to achieve full conversion and limited formation of **2a**′****, hence resembling the optimized conditions (entry 14, Table 1). By employing a weaker photocatalytic setup (i.e., 3 W vs 15 W), only 19% of the desired product **2a** was isolated over 16 h of reaction time (entry 15, Table 1). This outcome highlights how the reaction kinetics are affected by the intensity of the LEDs used.

**Table 1:**
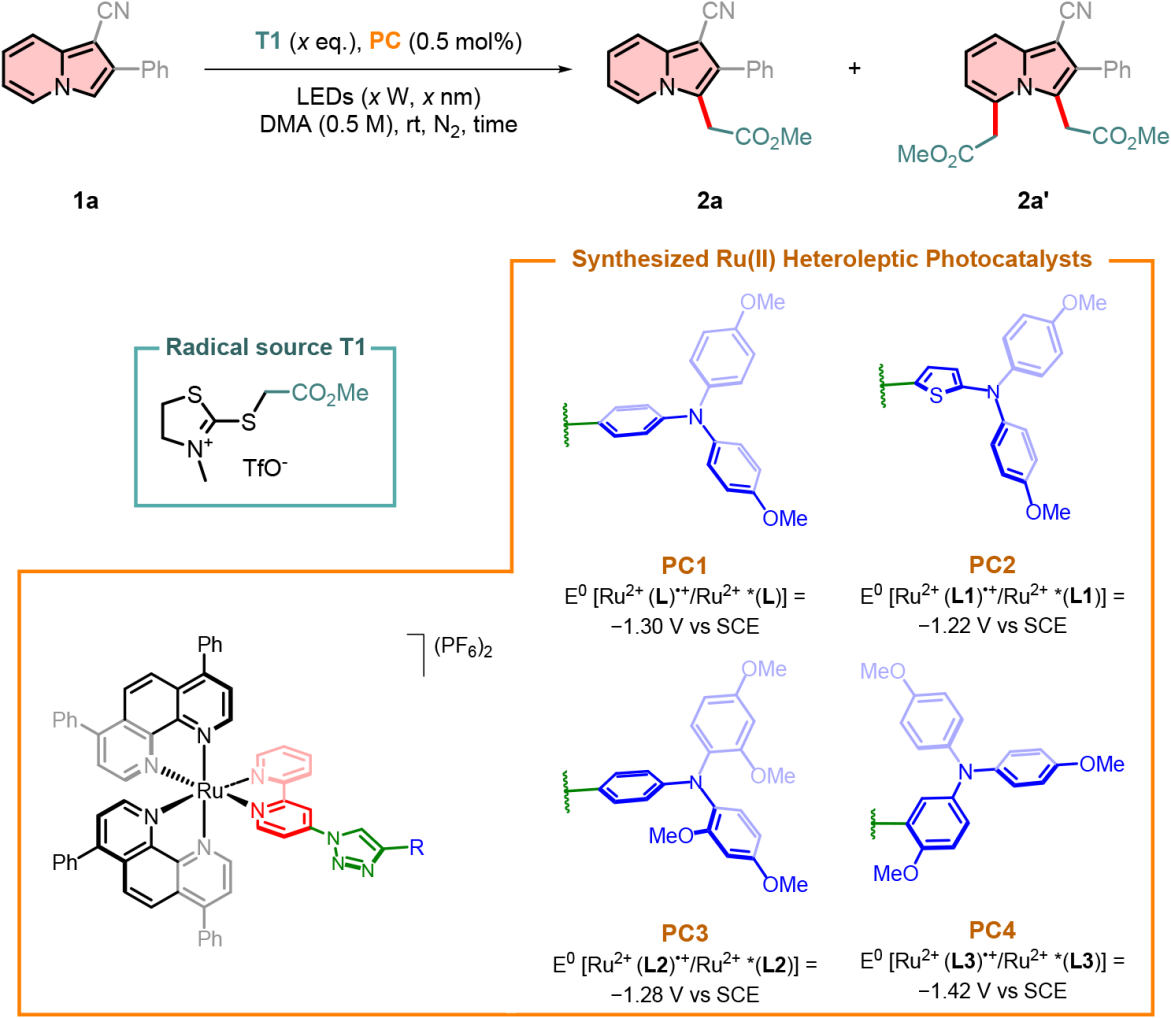
Optimization Studies for the Synthesis of Indolizine **2a** ^b^

Entry	Photoredox Catalyst	Radical Source eq	Time	LEDs	Yield^a^ (2a: 2a′: 1a)
1	**PC1**	1.5	3 h	green (15 W, 515 nm)	67%: 18%: 0%
2	**PC2**	1.5	3 h	green (15 W, 515 nm)	70%: 16%: 0%
3	**PC3**	1.5	3 h	green (15 W, 515 nm)	68%: 17%: 0%
4	**PC4**	1.5	3 h	green (15 W, 515 nm)	60%: 20%: 0%
5	[Ru(bpy)_3_](PF_6_)_2_	1.5	3 h	green (15 W, 515 nm)	47%: 0%: 50%
6	[Ru(phen)_3_](PF_6_)_2_	1.5	3 h	green (15 W, 515 nm)	43%: 0%: 57%
7	[Ru(dpp)_3_](PF_6_)_2_	1.5	3 h	green (15 W, 515 nm)	52%: 19%: 0%
8	Ir(ppy)_3_	1.5	3 h	green (15 W, 515 nm)	59%: 23%: 0%
9	4-CzIPN	1.5	3 h	green (15 W, 515 nm)	30%: 0%: 70%
10	–	1.5	3 h	green (15 W, 515 nm)	0%: 0%: 100%
11	**PC2**	1.1	3 h	green (15 W, 515 nm)	69%: 8%: 13%
12	**PC2**	1.25	3 h	green (15 W, 515 nm)	70%: 9%: 5%
13	**PC2**	1.5	1 h	green (15 W, 515 nm)	67%: 9%: 9%
14	**PC2**	1.5	2 h	green (15 W, 515 nm)	70%: 12%: 0% (isolated)
15	**PC2**	1.5	16 h	green (3 W, 515 nm)	19%: 0%: 81%

a Yields were determined by ^1^H NMR using dibromomethane as the internal standard, unless otherwise stated.

b Values for the excited-state redox potentials in V Vs SCE were obtained according to the literature.^30^

With the optimized conditions in hand, the scope and limitations of the reaction protocol were investigated (Scheme 2). Different radical sources of **T** were evaluated on various substituted 2-phenylindolizines **1**. Radical sources bearing an ester group, such as **T1**–**T3**, were initially applied. Consistent yields were obtained when both 2-(phenyl)­indolizine-1-carbonitrile **1a** and 2-phenylindolizine **1b** were employed (i.e., 64–72%). In contrast, a decrease in yields was observed with indolizine-1-carboxylates **1c** and **1d**, with the ethyl analogue producing the lowest amount of the desired product among them. In the case of radical source **T3**, a secondary radical was generated: due to steric and electronic factors, only indolizine **2b-3** was recovered in 51% yield. The presence of an electron-withdrawing group on the indolizine core was found to be unfavorable, as indolizine **2a-3** was isolated with a significantly lower yield of 14%.

**Scheme 2:**
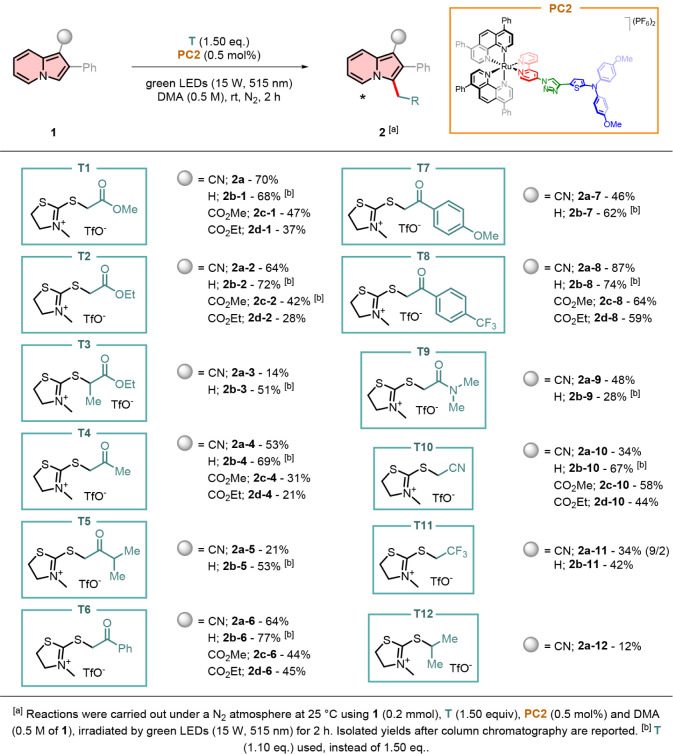
Radical Sources Substrate Scope for the Direct Radical Alkylation

Various 1-(indolizin-3-yl)­ketones were prepared by employing radical sources **T4**–**T8**. Simple acetone derivatives such as **2a-3** and **2b-3** were prepared in good yields (53% and 69%, respectively). Replacing the methyl group with a more electron-donating isopropyl group, as in **T5**, resulted in decreased yields of the desired product. This highlights the significant influence of electronic effects on the coupling efficiency between the radical source and the indolizine scaffold. Excellent results were achieved with acetophenone derivatives, where the substituent at the 4-position of the phenyl ring notably modulates the final product yields. Electron-withdrawing groups (e.g., CF_3_ in **T8**) enhance the formation of the alkylated indolizines, regardless of the substituent at position 1. Conversely, electron-rich phenyl rings (e.g., 4-MeOC_6_H_4_ in **T7**) generally diminish yields, with only indolizine **1b** being able to generate product **2b-7** in a consistent yield of 62%. In this context, indolizine-1-carboxylates **1c** and **1d** afforded slightly lower yields compared to **1a** and **1b**. However, in contrast to the previous analogues, indolizine **1d** outperformed the methyl derivative overall.

*N*,*N*-Dimethylacetamide derivatives were successfully isolated in good yields, starting from radical source **T9**. These compounds serve as promising precursors for the synthesis of potentially interesting and novel entactogens.^31,32^

Thereafter, different electron-withdrawing groups, other than a simple carbonyl on radical source **T**, were applied. In the case of **T10**, 1-(indolizin-3-yl)­acetonitriles **2a-d-10** were successfully isolated in good yields, ranging from 34 to 67%. Conversely to previous results, indolizine-1-carboxylates **1c** and **1d** demonstrated superior yields compared to indolizine **1a**. This shows how the slightly less electron-deficient **1c** and **1d** can be more easily coupled with a methylene radical that bears a strong electron-withdrawing group, and vice versa, according to Hammett parameters.^33^

If no electron-withdrawing group is capable of exerting a mesomeric effect (e.g., CF_3_ in **T11**) in the formed methylene radical, a decrease in yield is observed. Furthermore, for indolizine **1a**, a loss of selectivity is noted, resulting in the isolation of an inseparable mixture of regioisomeric products.

As a control experiment, radical source **T12** was applied under the optimized conditions. In this case, a secondary radical bearing a two-electron-donating group was generated, which successfully reacted only with substrate **1a.** However, product **2a-12** was isolated in only 12% yield, indicating the need for an electron-withdrawing group on the generated methylene radical.

Successively, we proceeded to evaluate the indolizine scope under the optimized reaction conditions (Scheme 3). Unsubstituted 1*H*-indolizines were investigated first: yields of the desired products **2e–2i** were consistent (64–77%), regardless of the substituent at position 4 of the phenyl substituent. A slightly reduced yield (59%) was observed for indolizine **2k**. This decrease may be attributed to steric hindrance caused by the methoxy group at the 2-position of the phenyl ring, which likely prevents the addition of the generated radical. The protocol proved to be respectful of OH functionalities, as product **2j** was isolated with 47% yield. Indolizine **1l**, a bioisostere of the gastroprotective drug Zolimidine,^34^ was efficiently coupled with the alkyl radical, forming product **2l** in 77% yield. Heteroaromatics (e.g., thiophene) were also tolerated, as shown by the successful synthesis of product **2m**, isolated in 63% yield. In contrast, further substitution of the 6-position of the indolizine core led to diminished yields, regardless of the electronic nature of the substituent on the 2-position. This trend is demonstrated by indolizine **2n** isolated in 49% yield. Conversely, an improved outcome was obtained when the substituent on the 6-position was modestly electron-withdrawing (e.g., Br vs Me), as for indolizine **2o**, which was obtained in 57% yield. Simple 2-methylindolizine **1p** was also evaluated, resulting in the successful isolation of product **2p** in a yield of 51%.

**Scheme 3:**
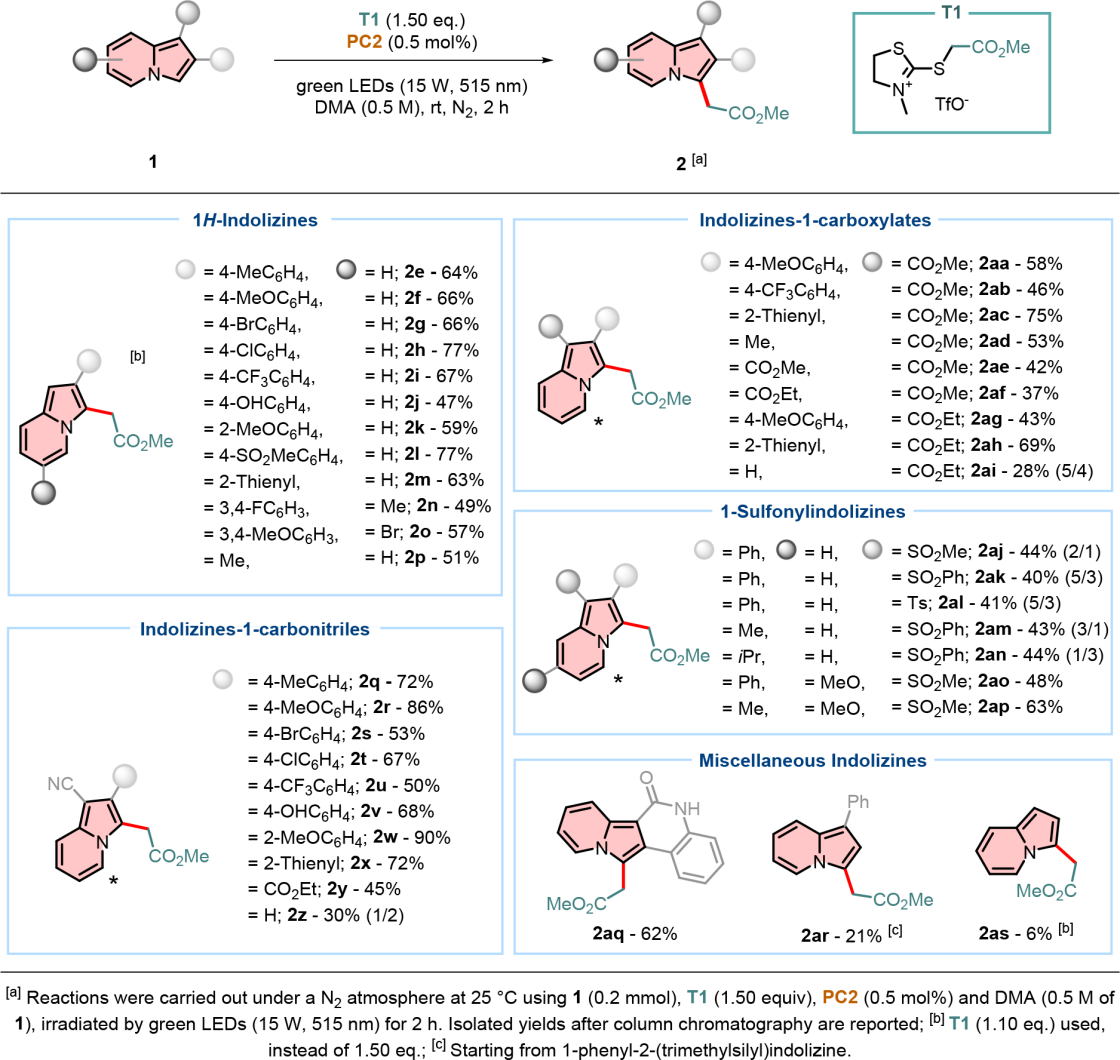
Indolizines Substrate Scope for the Direct Radical Alkylation

Compared to 1*H*-indolizines, indolizine-1-carbonitriles showed a dependence on the electronic nature of the substituent at the 2-position. The presence of strong electron-donating methoxy substituents, regardless of the position on the phenyl ring, significantly enhanced the reaction efficiency, converting substrates **1r** and **1w** into the desired indolizines in 86% and 90% yields, respectively. On the other side, electron-withdrawing substituents (e.g., halogens or CF_3_ group) inhibit the desired pathway, affording products **2s**, **2t**, and **2u** in 53%, 67%, and 50% yields, respectively. Simple *p*-tolyl or 2-thienyl substituents did not affect the reaction outcome, resulting in the formation of both products **2q** and **2x** in 72% yield, similar to indolizine **2a-1**. An OH group was well tolerated, as shown for product **2v**, which was isolated with a higher yield compared to its 1*H*-indolizine analogue **2j** (i.e., 68% vs 47% yield). Conversely, attaching an ethyl ester group at the 2-position of the indolizine core improved the yield of the desired product, with compound **2y** isolated in 45% yield, albeit this was lower than in previous examples of 2-phenylindolizine-1-carbonitriles. In the case of indolizine-1-carbonitrile **1z**, the absence of any substituent on the 2-position led to a decreased yield and a loss in selectivity. Product **2z** was isolated in 30% yield as an inseparable 1:2 mixture of the desired 3-alkylated and 5-alkylated products.

Indolizine-1-carboxylates were further investigated under the optimized conditions. Generally, as previously stated, methyl indolizine-1-carboxylates were higher yielding compared to their ethyl analogues, as shown for products **2aa** and **2ag**, isolated with 58% and 43% yields, respectively. In contrast to these findings, the introduction of a CF_3_ group at the 4-position of the phenyl ring—with respect to indolizine 1c—resulted in no significant change in the overall yield. Specifically, indolizine **2ab** was obtained in 46% yield, comparable to 47% for **2c-1**. The presence of a 2-thienyl substituent on the 2-position proved to be beneficial for the yields, compared to the phenyl derivatives **2c-1** and **2d-1**, as products **2ac** and **2ah** were isolated in 75% and 69% yield, respectively. Similarly, the absence of an aromatic substituent at the 2-position led to only a slight reduction in yield, with methyl 2-methylindolizine-1-carboxylate **1ad** affording the desired product in 53% yield. For indolizine-1,2-carboxylates, a clear trend was observed: higher yields were obtained with increasing numbers of methyl ester groups on the indolizine core. Specifically, desired products **2ae** and **2af** were isolated in yields of 42% and 37%, respectively. Ethyl indolizine-1-carboxylate **1ai** was also coupled with the radical source **T1**. Remarkably, similar to product **2z**, no selectivity was noted between the 3- and 5-positions on the indolizine core, resulting in an inseparable mixture of regioisomers that was isolated in 28% yield.

1-Sulfonylindolizines proved to be challenging substrates under the optimized conditions. Regardless of the substituents present at both the 1- and 2-positions, the reactions consistently yielded comparable results, with product yields ranging from 40% to 44% for compounds **2aj–2am**. The desired products were invariably obtained as a mixture with their corresponding 5-alkylated regioisomers, indicating limited regioselectivity under the current conditions. Notably, indolizine **1an** was the only case exhibiting reversed regioselectivity, with the 5-alkylated indolizine emerging as the major product. This outcome can be attributed to steric effects, as the isopropyl group at the 2-position hinders radical insertion at the 3-position, directing the reaction toward the 5-position. Further functionalization of the indolizine core at the 7-position with a methoxy group significantly enhanced both yield and regioselectivity. Thus, products **2ao** and **2ap** were obtained with improved yields (48% and 63%, respectively) as single regioisomers, in contrast to previous examples.

As a key motif for the synthesis of novel organic fluorophores,^35^ indolizino­[1,2-*c*]­quinolin-6­(5*H*)-one **1aq** was also investigated under the optimized conditions. The targeted compound **2aq** was successfully isolated in a satisfactory yield of 62%. 1-Phenyl-2-(trimethylsilyl)­indolizine **1ar** yielded an interesting outcome: although the isolated yield of **2ar** was modest (i.e., 21%), no TMS group was detected on the indolizine core after the reaction. The latter suggests a potential limitation in the protocol’s tolerance for certain functional groups. Another limitation was highlighted with the reaction of simple indolizine **1as**, as the desired product was obtained with a scarce 6% yield. This outcome points out the fact that substitution at either the 1- or 2-position is necessary for the reaction to occur efficiently.

To summarize, electronic factors mainly govern the addition of the generated electrophilic radicals to the indolizine core. The 3-position is the primary site of alkylation in indolizines featuring a higher electron density on the five-membered ring. The presence of strong electron-withdrawing substituents at positions 1 or 2, or the absence of a substituent at position 2, diminishes the selectivity by promoting the formation of 5-alkylated products. This shift occurs due to a redistribution of electron density toward the six-membered ring and the inability to stabilize a carbocation intermediate (as shown for **INT-III** in Scheme 10). Therefore, careful selection between the generated radical and indolizine electronic properties is crucial for optimizing reaction outcomes.

To assess the generality of our designed protocol, different *N*-heteroarenes were subjected to the optimized reaction conditions (Scheme 4). Like indolizines, indazoles are important nitrogen-containing aromatic compounds that play a crucial role in the field of pharmaceutical chemistry because of their wide range of biological activities.^36^ 2-(4-Methoxyphenyl)-2*H*-indazole **1at** was successfully coupled with radical source **T1**, yielding compound **2at** in 44% yield. A higher yield was obtained when the *N*-heteroarene substrate was a protected tryptophan **1au**: chemoselective C2 functionalization was achieved in a 71% yield. Because of its intrinsic simplicity and excellent functional group tolerance, our protocol is well-suited for the selective late-stage functionalization of more complex peptides.^37^

**Scheme 4:**
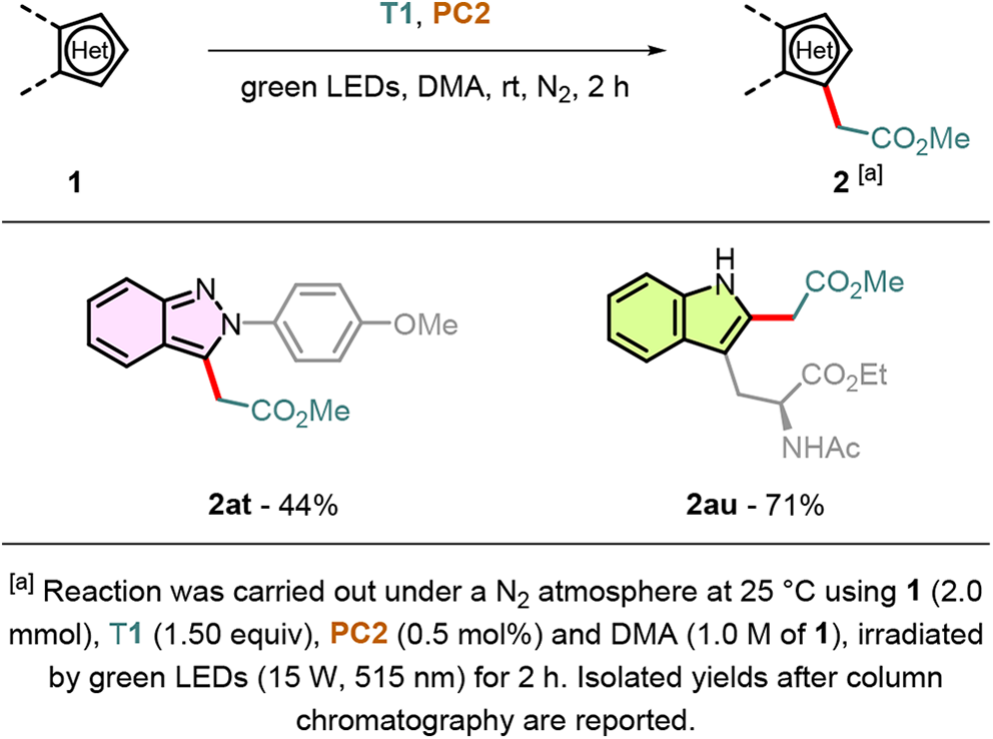
Substrate Scope of *N*-Heteroarenes

The versatility of the developed protocol was demonstrated through a 2.0 mmol-scale experiment, yielding indolizine **2a** in 69% under optimized conditions (Scheme 5). Notably, the reaction time required extension from 2 to 6 h, as only 27% of product **2a** was isolated after the initial 2-h period.

**Scheme 5:**
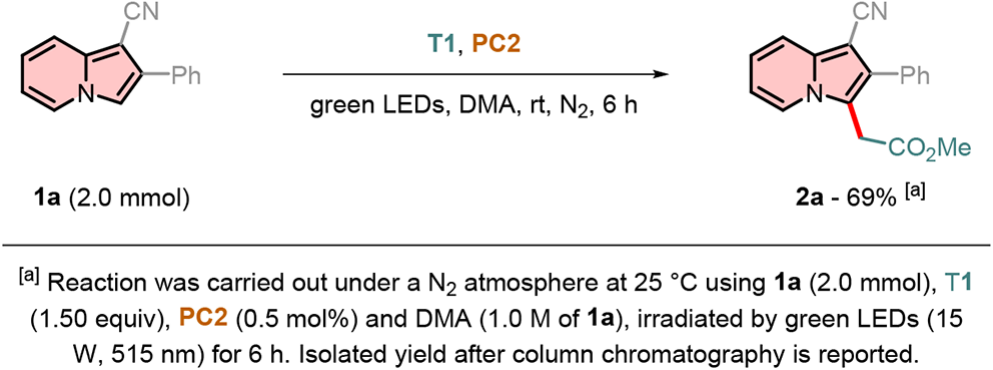
Reaction Scale-Up

Zolpidem was the first imidazo­[1,2-*a*]­pyridine drug to enter the market in 1992 and is used to treat insomnia.^38^ Despite its interesting pharmacological profile, its preparation requires multiple synthetic steps and the use of toxic reagents, such as SOCl_2_ to install the amide functional group.^39^ Indolizines are recognized as bioisosteres of imidazo­[1,2-*a*]­pyridines.^40^ Given their relevance in medicinal chemistry,^1^ we efficiently synthesized compound **2av-9** within a one-step procedure, achieving a synthetically useful yield of 53% (Scheme 6).

**Scheme 6:**
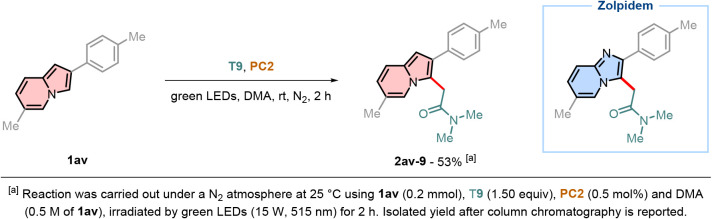
One-Step Synthesis of Zolpidem Bioisostere

Numerous imidazo­[1,2-*a*]­pyridines have been reported to exhibit antitubercular (anti-TB) activity.^41,42^ In 2022, Khetmalis et al. developed a series of new anti-TB drugs by tethering imidazo­[1,2-*a*]­pyridines to tetrahydropyridines, demonstrating promising biological activity.^43^ Inspired by these findings, we developed a new approach, starting from indolizine **1o**, to synthesize a functionalized bioisostere of a key anti-TB precursor. After preparing the desired intermediate **2o**, its efficient Suzuki coupling with the corresponding tetrahydropyridine derivative led to compound **3** with 90% yield (Scheme 7).

**Scheme 7:**
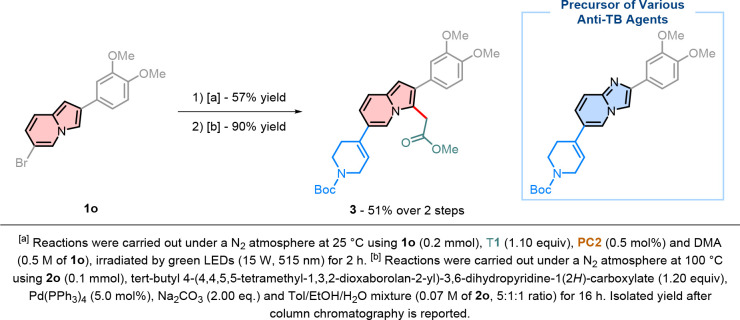
Synthesis of a Precursor for an Anti-TB Agents Bioisostere from **2o**

To showcase the synthetic versatility of the installed 2-methoxy-2-oxoethan-1-yl (−CH_2_CO_2_Me) group, indolizine **2a** was subjected to a series of further modifications. Initially, deprotonation of the activated methylene unit with a strong base, such as sodium hydride (NaH) enabled electrophilic trapping with 4-acetamidobenzenesulfonyl azide (*p*-ABSA), resulting in compound **4a** (Scheme 8a). This intermediate can be readily transformed into the corresponding ketone or reduced to synthesize a nonnatural α-amino acid. When an electrophile such as a haloalkane—specifically 1,4-dibromobutane—is employed, compound **4b** is prepared with 73% yield (Scheme 8b). Additionally, indolizine **2a** can participate in a Knoevenagel-type reaction with aldehydes; for our purposes, we utilized a Fluvastatin precursor, leading to the formation of compound **4c** (Scheme 8c). Indolizine **4c**, given its extended π-system, showed interesting spectroscopic properties. When the same optimized conditions are applied to indolizine **2a**, the formation of compound **2a′** was observed in good yield (Scheme 8d).

**Scheme 8:**
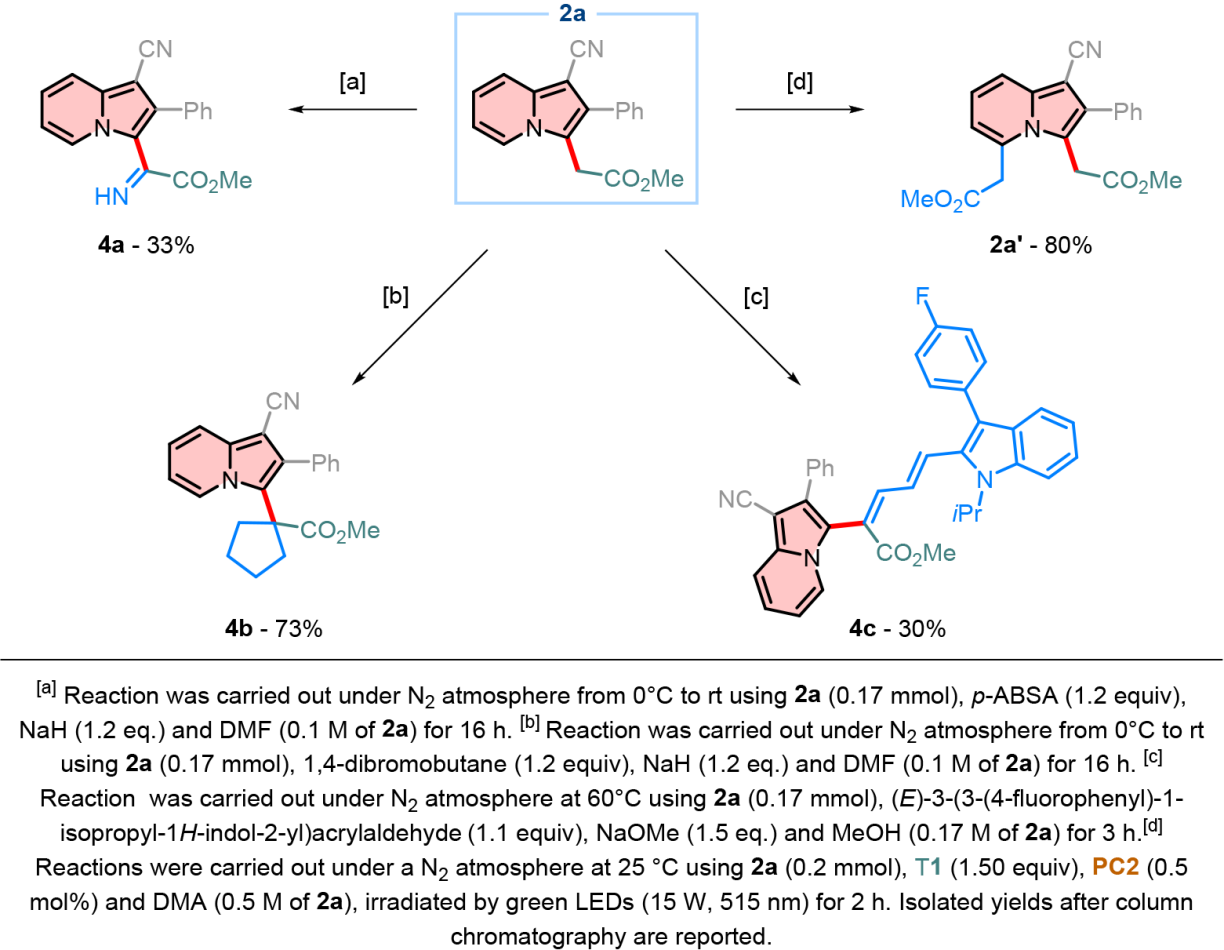
Derivatization of Indolizine **2a**

Upon selective hydrolysis of the ester functionality of indolizine **2a**, compound **4d** was produced in quantitative yield (Scheme 9a). The free carboxylic acid group in compound **4d** can be easily coupled with a variety of nucleophiles by means of the DCC/DMAP system (Scheme 9b). When *N*,*O*-dimethylhydroxylamine was employed, Weinreb amide **5a** was produced in 93% yield; these intermediates are particularly useful for the synthesis of other carbonyl compounds, such as ketones, when treated with organometallic compounds.^44^ The amino group of the potent entactogen drug MDA^45,46^ was also coupled with **4d**, yielding compound **5b** in good yield. Lastly, the coupling between the naturally occurring monoterpenoid (2*S*,5*R*)-menthol and indolizine **4d** produced ester **5c** in quantitative yield.

**Scheme 9:**
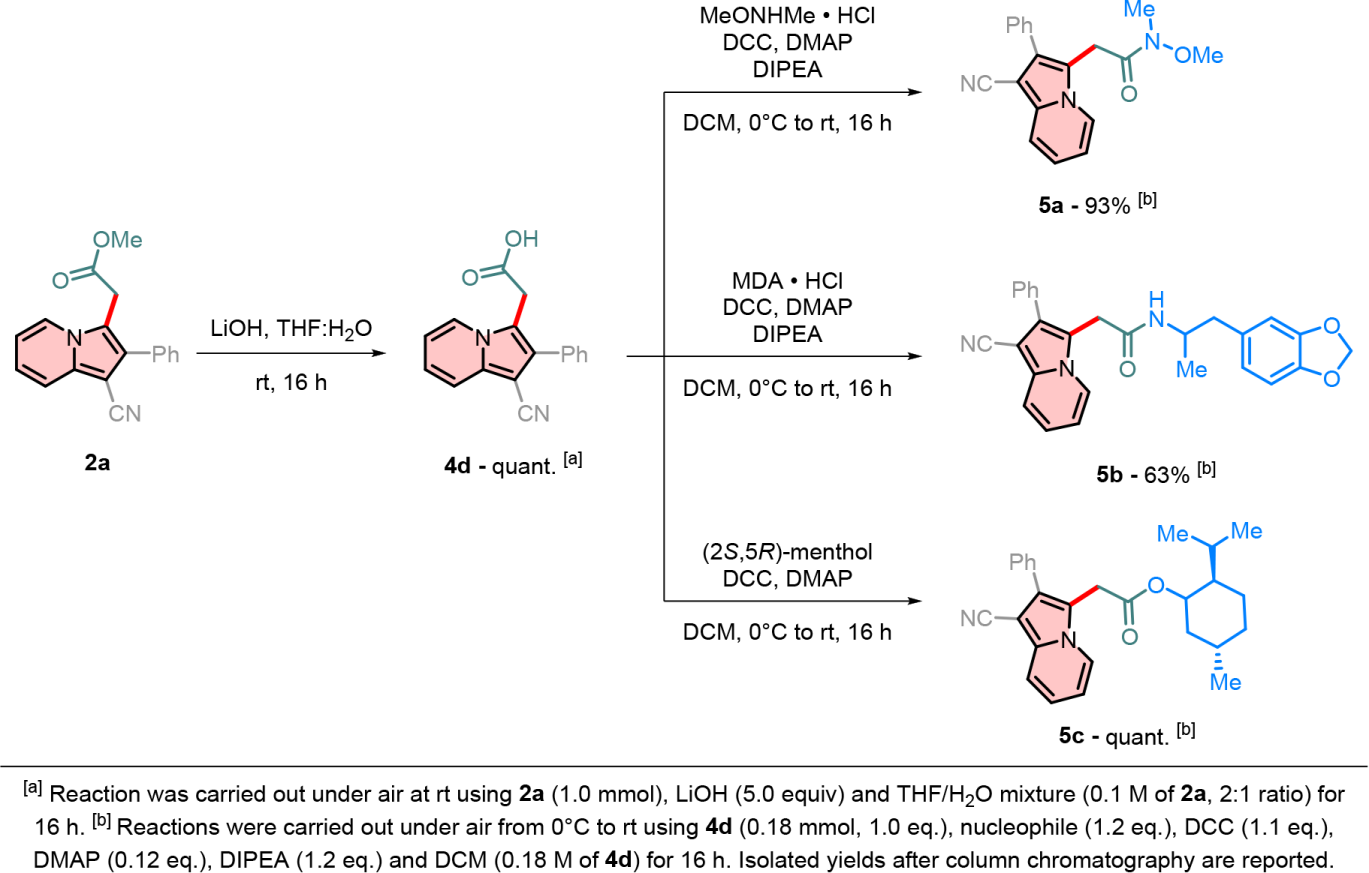
Derivatization of the Carboxylate Moiety

Based on our previous work^27,28^ and other literature reports,^47–49^ a plausible reaction mechanism for the indolizine photocatalytic radical C­(sp^2^)–C­(sp^3^) alkylation can be proposed (Scheme 10). First, photoredox catalyst **PC2** is excited (from Ru^2+^ (**L1**) to Ru^2+^ *­(**L1**) upon green light irradiation). The interaction between Ru^2+^ *­(**L1**) and the radical source T1 (*E*_p_ = −0.91 V vs SCE) can generate the alkyl radical via Single-Electron-Transfer (SET), because of the higher reducing power of **PC2** (*E* ^0^ [Ru^2+^ (**L1**)^•+^/Ru^2+^ *­(**L1**)] = −1.22 V vs SCE), following C–S bond fragmentation through a β-scission process (**INT-I**). This latter generates a molecule of 3-methylthiazolidine-2-thione as a byproduct, alongside the desired alkyl radical. The formed radical can be intercepted by indolizine **1** to produce **INT-II**. The resulting radical species can then be oxidized by Ru^2+^ (**L1**)^•+^ to carbocation **INT-III**, as the reduction potential of the oxidized catalyst is sufficiently high (*E* ^0^ [Ru^2+^ (**L1**)^•+^/Ru^2+^ (**L1**)] = +0.69 V vs SCE). This process regenerates the catalyst and closes the photoredox cycle. Ultimately, a proton is readily abstracted from **INT-III**, thereby restoring aromaticity and yielding the desired product **2**. Additionally, a competitive radical pathway such as a radical chain process cannot be ruled out, as the initial irreversible quenching appears to be the limiting factor for the overall quantum yield. The latter is consistent with the relatively long irradiation time that implies a low overall quantum yield (i.e., well below 10%), suggesting that all photons are absorbed at the given catalyst loading, but the reaction time is relatively long with the high-power LEDs.^28^

**Scheme 10:**
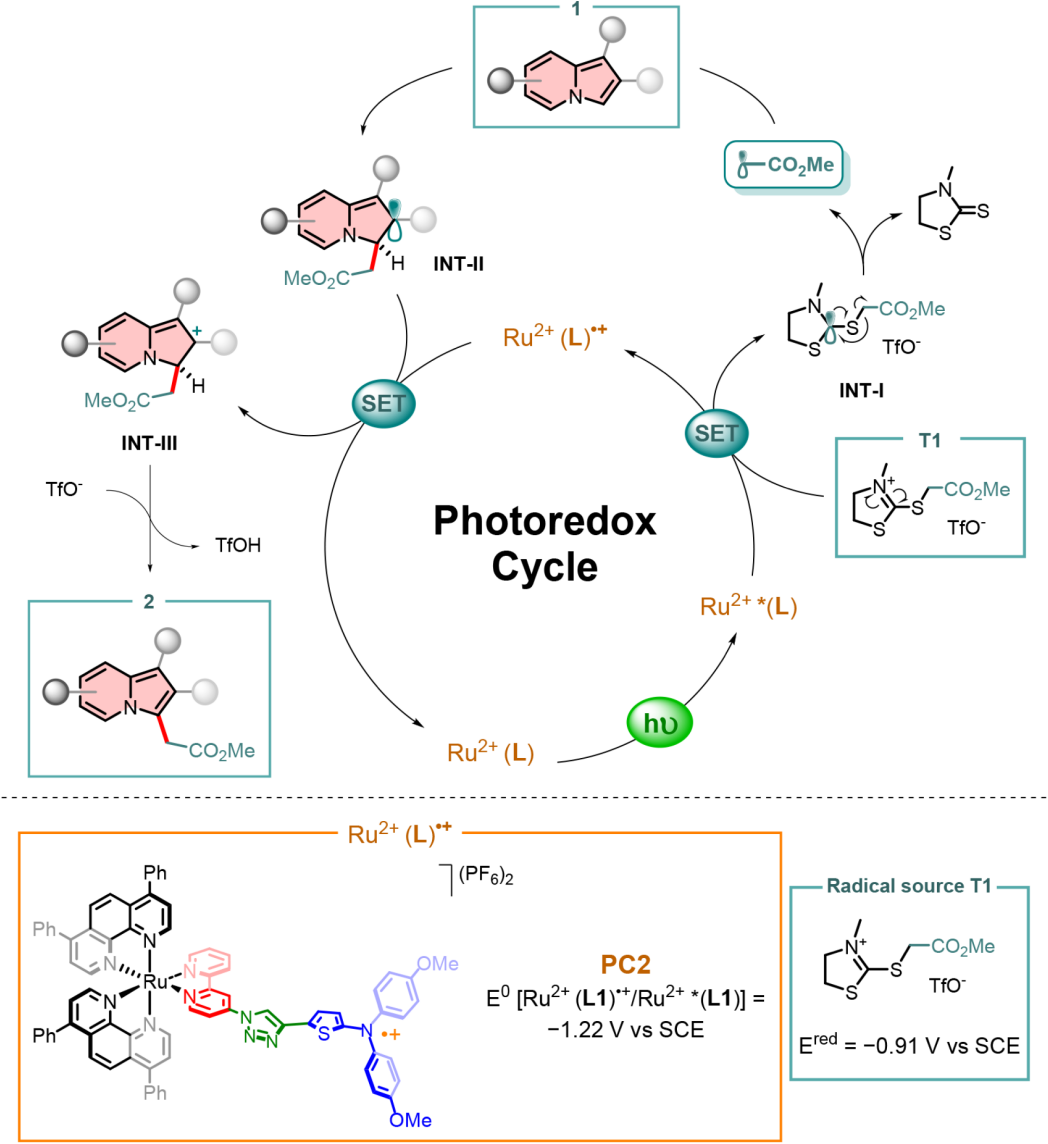
Proposed Reaction Mechanism

## Conclusion

In summary, a mild, robust, and chemoselective protocol for the radical C­(sp^2^)–C­(sp^3^) alkylation of indolizines has been established. The reaction employs inexpensive, air-insensitive, and readily accessible reagents, enabling the convenient synthesis of substituted indolizines, which can be further used as scaffolds for drug design in medicinal chemistry. The protocol has proven to be general and can be scaled up to gram-scale without significant loss in yields. Moreover, different *N*-heteroarenes, such as 1*H*-indoles and 2*H*-indazoles, have been successfully alkylated under the optimized conditions.

## Experimental Section

### General Experimental

All reactions were carried out under an atmosphere of nitrogen in oven-dried glassware, unless otherwise stated. All reactions that required heating were conducted by using a steel heat-on block. All chemicals were purchased and used without further purification unless otherwise mentioned. Anhydrous solvents were dried according to standard procedures before use and stored in a glovebox. All NMR-spectra spectra were measured using either BRUKER Digital AVANCE 400 MHz FT-NMR or BRUKER Digital AVANCE III 600 MHz FT-NMR. Chemical shifts are reported in ppm, and the coupling constants in Hz. All mass spectra were measured using a Hewlett-Packard Agilent LC/MSD-System Series HP 1100 with API-ES, and the detector is TOF. All UV–vis spectra were measured using a JASCO V-650 spectrophotometer or a JASCO V-760 spectrophotometer. The reactions were traced by thin-layer chromatography with silica gel 60 (F254, MERCK KGAA). For the detection of substances, quenching was used at either 254 or 366 nm with a UV lamp. Preparative column chromatography was conducted through silica gel 60 (230–400 mesh).

All cyclic voltammetry experiments were performed in 0.1 M [Bu_4_N]­[PF_6_] MeCN solution with an Autolab PGSTAT204 potentiostat/galvanostat (Metrohm). A cell with a three-electrode configuration was used. The glassy carbon working electrode (*d* = 2 mm) was polished before each measurement with a 0.03 μm Al_2_O_3_ slurry and then rinsed thoroughly with deionized water and MeCN. A platinum sheet was used as the counter electrode, while the reference electrode was Ag/AgCl (3.0 M KCl). The measurements were performed at a temperature of 22 °C under a nitrogen atmosphere after the solutions were bubbled with the same gas for 10 min. A prebubbler was also included in the experimental setup to prevent excessive evaporation of the solvent. The IUPAC plotting convention was used to plot the voltammograms. The initial potential was set to 0.0 V, and the scan proceeded in the oxidation direction up to the potential shown in the plot.

Reactions were performed in closed vials, illuminated from below with five Avonec 3 W High Power LEDs (https://www.avonec.de/3w-high-power-led/) affixed to a cooling block, and the setup was prevented from heating through a continuous airflow, as depicted in Figure S1. No filters were used during the irradiation process. The emission spectrum of the green LEDs used is shown in Figure S2.

### General Procedure for the Photoredox Experiments

#### General Procedure A

In a glovebox, to a vial filled with indolizine **1** (0.20 mmol, 1.00 equiv), radical source **T** (0.30 mmol, 1.50 equiv), and **PC2** (1.6 mg, 1.0 μmol, 0.5 mol %), DMA (0.4 mL, 0.50 M) was added. The vial was sealed, and the mixture was irradiated with green LEDs (15 W, 515 nm) for 2 h at rt outside of the glovebox. The reaction mixture was partitioned between H_2_O and Et_2_O, and the organic phase was extracted. The water phase was extracted with Et_2_O 3 times, and the combined organic phases were washed with H_2_O and brine. The organic phase was dried over Na_2_SO_4_, and the solvent was removed under reduced pressure. The residue was purified by flash column chromatography on silica gel to afford the functionalized product **2**.

#### General Procedure B

In a glovebox, to a vial filled with indolizine **1** (0.20 mmol, 1.00 equiv), radical source **T** (0.30 mmol, 1.50 equiv), and **PC2** (1.6 mg, 1.0 μmol, 0.5 mol %), DMA (0.4 mL, 0.50 M) was added. The vial was sealed, and the mixture was irradiated with green LEDs (15 W, 515 nm) for 2 h at rt outside of the glovebox. The reaction mixture was partitioned between H_2_O and Et_2_O, and the organic phase was extracted. The water phase was extracted with Et_2_O 3 times, and the combined organic phases were washed with H_2_O and brine. The organic phase was dried over Na_2_SO_4_, and the solvent was removed under reduced pressure. The residue was purified by flash column chromatography on silica gel to afford the functionalized product **2**, alongside the byproduct of the reaction, as an inseparable mixture. The mixture was dissolved in DCM/Et_2_O (0.25 M, 1:1), and an excess of methyl iodide (0.90 mmol, 3.0 equiv) was added. The resulting mixture was stirred for 3 h at 40 °C, after which the solvent was evaporated in vacuo. The residue was suspended in Et_2_O, the precipitated solid was filtered, washed with Et_2_O, and the filtrate was collected and concentrated in vacuo, yielding the desired product **2**.

#### General Procedure C

In a glovebox, to a vial filled with indolizine **1** (0.20 mmol, 1.00 equiv), radical source **T** (0.22 mmol, 1.10 equiv), and **PC2** (1.6 mg, 1.0 μmol, 0.5 mol %), DMA (0.4 mL, 0.50 M) was added. The vial was sealed, and the mixture was irradiated with green LEDs (15 W, 515 nm) for 2 h at rt outside of the glovebox. The reaction mixture was partitioned between H_2_O and Et_2_O, and the organic phase was extracted. The water phase was extracted with Et_2_O 3 times, and the combined organic phases were washed with H_2_O and brine. The organic phase was dried over Na_2_SO_4_, and the solvent was removed under reduced pressure. The residue was purified by flash column chromatography on silica gel to afford the functionalized product **2**.

#### General Procedure D

In a glovebox, to a vial filled with indolizine **1** (0.20 mmol, 1.00 equiv), radical source **T** (0.22 mmol, 1.10 equiv), and **PC2** (1.6 mg, 1.0 μmol, 0.5 mol %), DMA (0.4 mL, 0.50 M) was added. The vial was sealed, and the mixture was irradiated with green LEDs (15 W, 515 nm) for 2 h at rt outside of the glovebox. The reaction mixture was partitioned between H_2_O and Et_2_O, and the organic phase was extracted. The water phase was extracted with Et_2_O 3 times, and the combined organic phases were washed with H_2_O and brine. The organic phase was dried over Na_2_SO_4_, and the solvent was removed under reduced pressure. The residue was purified by flash column chromatography on silica gel to afford the functionalized product **2**, alongside the byproduct of the reaction, as an inseparable mixture. The mixture was dissolved in DCM/Et_2_O (0.25 M, 1:1), and an excess of methyl iodide (0.66 mmol, 3.0 equiv) was added. The resulting mixture was stirred for 3 h at 40 °C, after which the solvent was evaporated in vacuo. The residue was suspended in Et_2_O, the precipitated solid was filtered, washed with Et_2_O, and the filtrate was collected and concentrated in vacuo, yielding the desired product **2**.

## Supplementary Material



## Data Availability

The data underlying this study are available in the published article and its Supporting Information.

## Notes

Full experimental description, spectral data of the compounds, and additional spectroscopy data (PDF)
