# Supplementary material for: C–C Bond Formation via Direct Functionalization of Indolizines with a Bichromophoric Ruthenium Photocatalyst
Source: ACS Org Inorg Au. 2026 Feb 7;6(2):225–36. doi: 10.1021/acsorginorgau.5c00110 (PMC13047450; doi:10.1021/acsorginorgau.5c00110)
Supplement: Supplementary file 1 [file gg5c00110_si_001.pdf]

Supporting Information

C-C-Bond Formation *via* Direct Functionalization of Indolizines with Bichromophoric Ruthenium Photocatalyst

Kevin Klaus Stefanoni and René Wilhelm*

Institute of Organic Chemistry, Leibnizstr. 6, 38678, Clausthal-Zellerfeld, Clausthal University of Technology, Germany

Table of contents

1. General Experimental Information S3
2. Starting Materials Preparation S5
3. General Procedures for the Synthesis of Ligands S8
   3.1. General Procedure for the Synthesis of **L1** S8
   3.2. General Procedure for the Synthesis of **L2** S11
   3.3. General Procedure for the Synthesis of **L3** S14
4. General Procedures for the Synthesis of PC S17
   4.1. General Procedures for the Synthesis of **PC2** S17
   4.2. General Procedures for the Synthesis of **PC3** S18
   4.3. General Procedures for the Synthesis of **PC4** S18
5. General Procedures for the Photoredox Experiments S19
   5.1. General Procedure A S19
   5.2. General Procedure B S20
   5.3. General Procedure C S20
   5.4. General Procedure D S21
6. General Procedure for the Reaction Scale-Up S64
7. General Procedure for Suzuki Coupling of **2o** S65

8. General Procedures for Derivatization of Indolizine **2a** S65
9. General Procedure for Amidation/Esterification of **4d** S68
10. Absorption and Electrochemical Properties S71
11. NMR Spectra S74
12. References S193

1. General Experimental Information

All reactions were carried out under an atmosphere of nitrogen in oven-dried glassware, unless otherwise stated. All reactions that require heating were conducted using a steel heat-on block. All chemicals were purchased and used without further purification unless otherwise mentioned. Anhydrous solvents were dried according to standard procedures before usage and stored in a glovebox. All NMR-Spectra have been measured using either BRUKER Digital AVANCE 400 MHz FTNMR or a BRUKER Digital AVANCE III 600 MHz FT-NMR. The chemical shifts are reported in ppm, the coupling constants in Hz. All mass spectra have been measured using a Hewlett-Packard Agilent LC/MSD-System Series HP 1100 with API-ES and the detector is TOF. All UV-vis spectra have been measured using a JASCO V-650 spectrophotometer or a JASCO V-760 spectrophotometer. The reactions were traced by thin layer chromatography with silica gel 60 (F254, MERCK KGAA). For the detection of substances, quenching was used at either 254 nm or 366 nm with a UV lamp. The preparative column chromatography was conducted through silica gel 60 (230–400 mesh).

All cyclic voltammetry experiments were performed in 0.1 M $[Bu_4N][PF_6]$ MeCN solution with an Autolab PGSTAT204 potentiostat/galvanostat (Metrohm). A cell with a three-electrode configuration was used. The glassy carbon working electrode (*d*=2 mm) was polished before each measurement with a 0.03 µm Al_2O_3 slurry and then rinsed thoroughly with deionized water and MeCN. A platinum sheet was used as counter electrode, while the reference electrode was Ag/AgCl (3.0 M KCl). The measurements were performed at a temperature of 22 °C under a nitrogen atmosphere after bubbling the solutions with the same gas for 10 min. A pre-bubbler is also included in the experimental set-up, to prevent excessive evaporation of the solvent. The IUPAC plotting convention was used to plot voltammograms. The initial potential was set to 0.0 V, and the scan proceeded in the oxidation direction up to the potential shown in the plot.

Reactions were performed in closed vials illuminated from below with five Avonec 3 W High Power LEDs (https://www.avonec.de/3w-high-power-led/) affixed to a cooling block and the setup was prevented from heating through a continuous air flow, as depicted in **Figure S1**. No filters were used during the irradiation process. The emission spectrum of the used green LEDs is shown in **Figure S2** below.

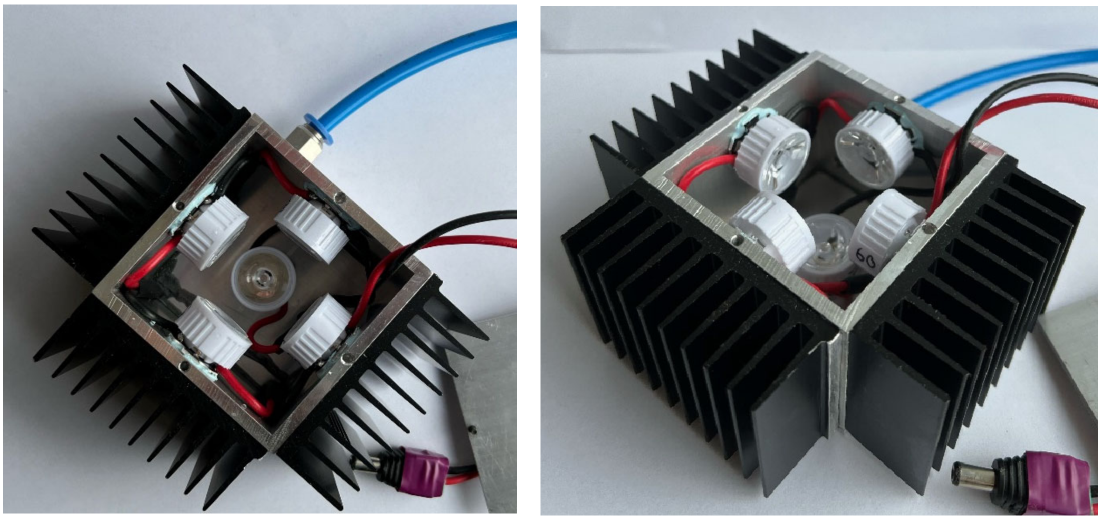

Figure S1. Custom LED photoreactor for reaction optimization and scale-up processes.

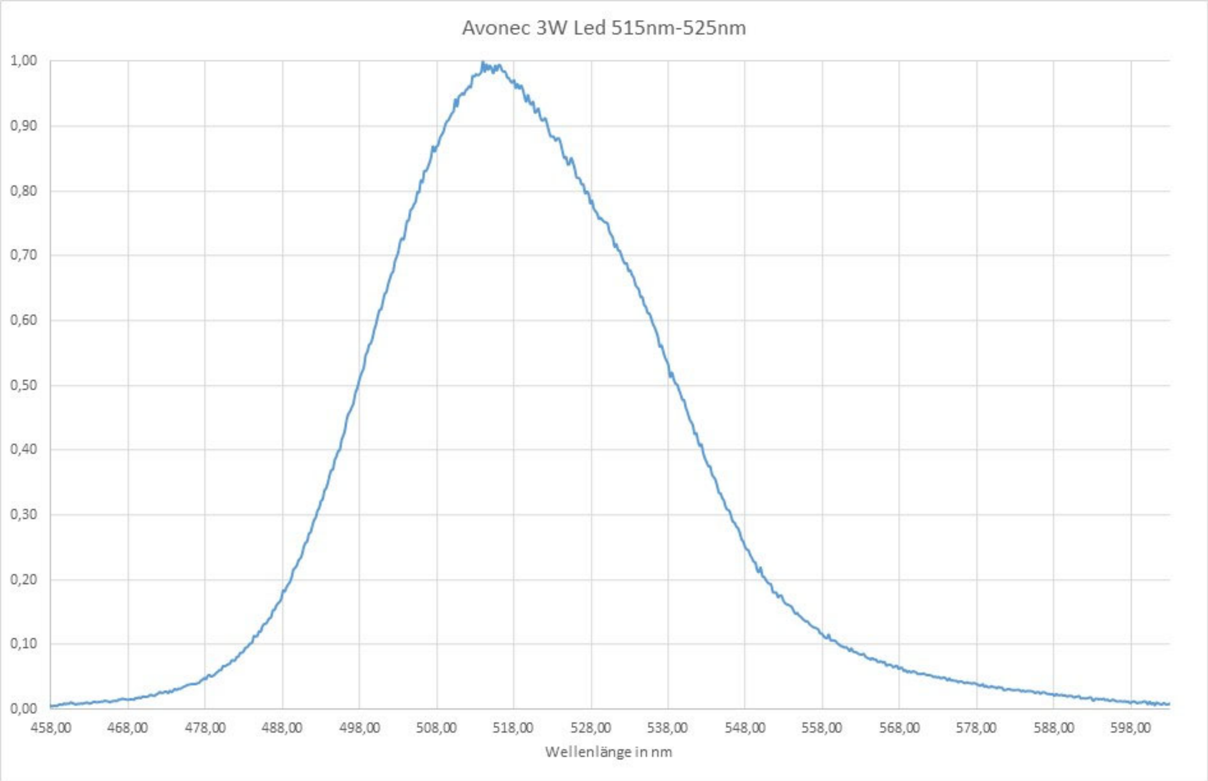

Figure S2. Emission spectrum of the used 515 nm LED lamp, as provided by the manufacturer.

2. Starting Materials Preparation

Indolizines **1a, 1c, 1d, 1q – 1y, 1aa – 1ah, 1aj – 1ap** were prepared by boiling an equimolar MeCN or Acetone or EtOH solution of the corresponding substituted picoline and α-bromoacetophenone with $NaHCO_3$ (2.0 eq.) for 16 h, according to literature method.[1] Indolizines **1b, 1e – 1p, 1av** were prepared according to the literature.[2,3] Indolizines **1aq** was prepared according to the literature.[4] Indolizines **1z, 1ai** were prepared according to the literature.[5] Indolizine **1ar** was prepared according to the literature.[6] Indolizine **1as** was prepared according to the literature.[7] Radical sources **T1-T12** were prepared according to the literature.[8,9] 4-azido-[2,2'-bipyridine] 1-oxide was prepared according to a published method.[10] Ru(II) precomplexes were prepared by heating an *o*DCB solution of ligand and $[Ru(COD)Cl_2]_n$ at 140°C for 3 h, according to literature.[11] **PC1** was prepared as described earlier in the literature.[12] Reproduced from *J. Org. Chem.* **2025**, *90*, 6491-6503. Copyright 2025 American Chemical Society.

Reference 1

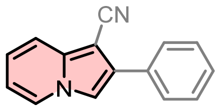

1a

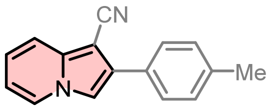

1q

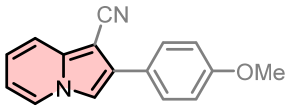

1r

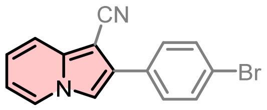

1s

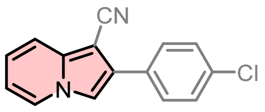

1t

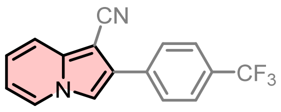

1u

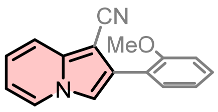

1v

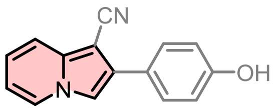

1w

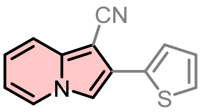

1x

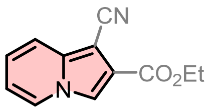

1y

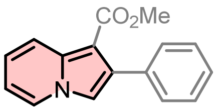

1c

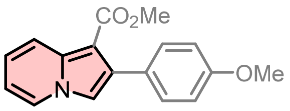

1aa

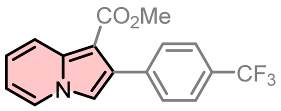

1ab

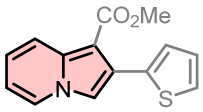

1ac

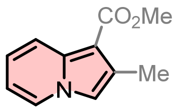

1ad

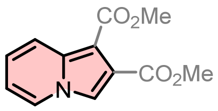

1ae

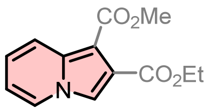

1af

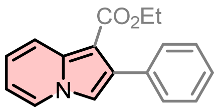

1d

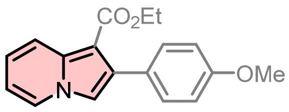

1ag

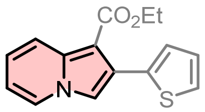

1ah

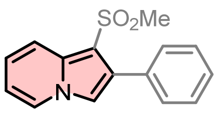

1aj

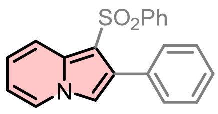

1ak

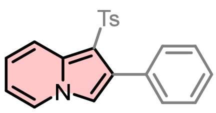

1al

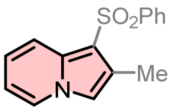

1am

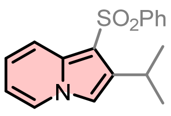

1an

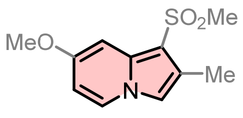

1ao

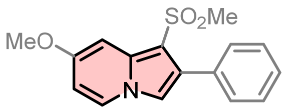

1ap

Reference 2,3

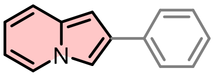

1b

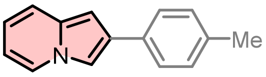

1e

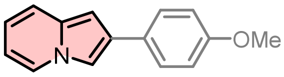

1f

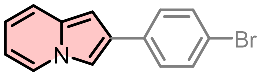

1g

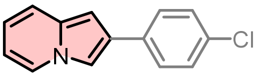

1h

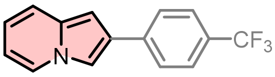

1i

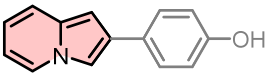

1j

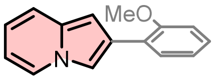

1k

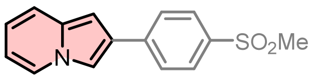

1l

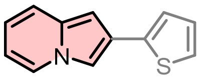

1m

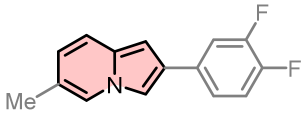

1n

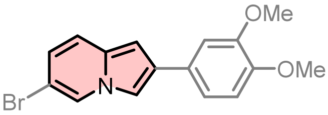

1o

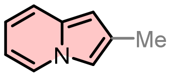

1p

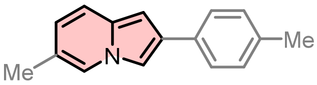

1av

Reference 4,5,6,7

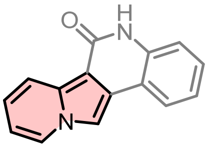

1ap

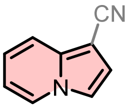

1z

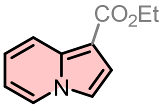

1ai

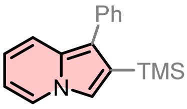

1aq

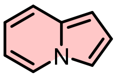

1ar

3. General procedure for synthesis of Ligands

3.1. General procedure for synthesis of L1

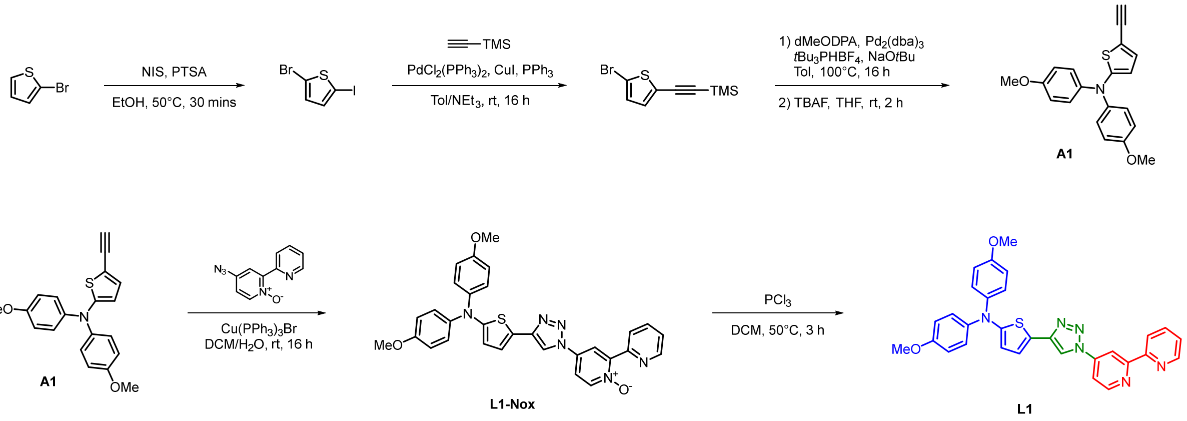

2-Bromo-5-iodothiophene[13] and ((5-bromothiophen-2-yl)ethynyl)trimethylsilane[14] were prepared according to published methods. The spectral data matched those of the literature.

5-Ethynyl-*N*,*N*-bis(4-methoxyphenyl)thiophen-2-amine (A1)

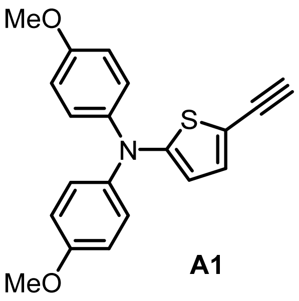

Under inert conditions, ((5-bromothiophen-2-yl)ethynyl)trimethylsilane (2.592 g, 10.00 mmol, 1.2 eq.), bis(4-methoxyphenyl)amine (1.911 g, 8.33 mmol, 1.0 eq.), $Pd_2(dba)_3$ (0.229 g, 0.25 mmol, 3 mol%), RuPhos (0.233 g, 0.50 mmol, 6 mol%) and NaO*t*Bu (0.881 g, 9.16 mmol, 1.1 eq.) in 1,4-dioxane (83 mL, 0.1 M) were heated to 110°C for 16 h. The reaction mixture was cooled to rt, filtered over a thin pad of Celite (EtOAc used as solvent) and the filtrate is concentrated in vacuo. The residue was purified by flash column chromatography on silica gel (PE:EtOAc, 95:5) to afford the intermediate product as a yellow oil. The intermediate was dissolved in THF (30 mL, 0.05 M) and a 1 M solution of TBAF (3.7 mL) was added dropwise on an ice bath. The reaction mixture was stirred for 2 h at rt and the solvent was later evaporated under reduced pressure. The residue is partitioned between H_2O and Et_2O, the organic phase is washed with H_2O/brine, dried over Na_2SO_4 and the solvent was removed under reduced pressure. The alkyne **A1** was obtained without further purification as a brown oil: 1.095 g, 3.3 mmol, 33% over 2 steps. **^{1}H NMR** (400 MHz, $CDCl_3$) δ 7.15 – 7.11 (m, 4H), 6.98 (d, J = 4.0 Hz, 1H), 6.86 –

6.82 (m, 4H), 6.19 (d, J = 4.0 Hz, 1H), 3.80 (s, 6H), 3.23 (s, 1H). **^{13}C{^{1}H} NMR** (100 MHz, $CDCl_3$) δ 156.6, 155.9, 140.9, 133.0, 125.6, 124.3, 114.8, 114.6, 113.1, 110.7, 79.7, 78.2, 55.6. **ESI-HRMS**: m/z calcd. for $C_{20}H_{17}NO_2SNa$ $[M+Na]^+$ 358.0878, found 358.0884.

4-(4-(5-(Bis(4-methoxyphenyl)amino)thiophen-2-yl)-1*H*-1,2,3-triazol-1-yl)-[2,2'-bipyridine] 1-oxide (L1-Nox)

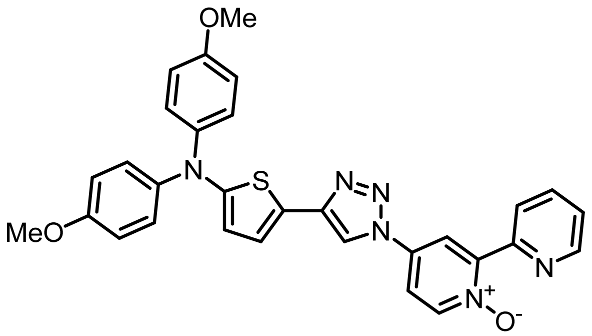

L1-Nox

To a degassed solution of DCM/H_2O (20 mL, 1:1, 0.05 M), 4-azido-[2,2'-bipyridine]-1-oxide (0.277 g, 1.30 mmol, 1.2 eq.), **A1** (0.363 g, 1.08 mmol, 1.0 eq.) and $Cu(PPh_3)_3Br$ (0.201 g, 0.11 mmol, 10 mol%) were added sequentially under inert atmosphere. The reaction mixture was stirred for 16 h at rt and later diluted with DCM. The organic phase was washed with H_2O/brine, dried over Na_2SO_4 and the solvent was removed under reduced pressure. The crude was purified by flash column chromatography on silica gel (DCM:MeOH, 98:2) to afford compound **L1-Nox** as a yellow/lime solid: 0.394 g, 7.19 mmol, 67%. **^{1}H NMR** (400 MHz, $CDCl_3$) δ z9.11 (d, J = 8.1 Hz, 1H), 8.77 – 8.76 (m, 1H), 8.60 (d, J = 3.2 Hz, 1H), 8.42 (d, J = 7.2 Hz, 1H), 8.09 (s, 1H), 7.92 – 7.87 (m, 2H), 7.42 (ddd, J = 7.6, 4.7, 1.1 Hz, 1H), 7.24 (d, J = 3.9 Hz, 1H), 7.17 – 7.15 (m, 4H), 6.87 – 6.84 (m, 4H), 6.42 (d, J = 3.9 Hz, 1H), 3.81 (s, 6H). **^{13}C{^{1}H} NMR** (125 MHz, $CDCl_3$) δ 156.4, 155.2, 149.6, 148.5, 145.0, 142.4, 141.2, 136.8, 133.2, 125.7, 125.3, 125.2, 124.5, 120.9, 117.2, 116.2, 115.1, 114.9, 114.8, 55.7. **IR** (neat, cm^{-1}): 3067, 2830, 1505, 1482, 1458, 1242, 1220, 1027, 1003, 819, 794, 764, 714, 676, 603, 588, 560, 521. **ESI-HRMS**: m/z calcd. for $C_{30}H_{24}N_6O_3SNa$ $[M+Na]^+$ 571.1528, found 571.1523.

5-(1-([2,2'-bipyridin]-4-yl)-1*H*-1,2,3-triazol-4-yl)-*N*,*N*-bis(4-methoxyphenyl)thiophen-2-amine (L1)

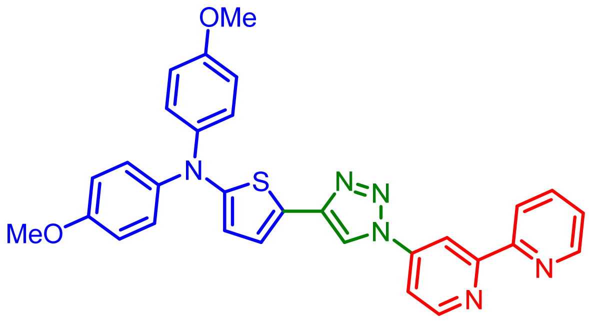

L1

To a solution of **L1-Nox** (0.393 g, 0.72 mmol, 1.0 eq.) in DCM (14 mL, 0.05 M), PCl_3 (0.295 g, 0.19 mL, 2.15 mmol, 3.0 eq.) was added at 0°C under inert atmosphere. The reaction mixture was refluxed for 3 h, then diluted with ice-cold H_2O and basified with 38% m/m NaOH solution. The aqueous layer was extracted with DCM, the combined organic phases are washed with H_2O/brine, dried over Na_2SO_4 and the solvent was removed under reduced pressure. Compound **L1** was obtained without further purification as a yellow lime fluffy solid: 0.381 g, 0.72 mmol, quantitative. If needed, **L1** can be purified by column chromatography on silica (DCM:MeOH, 99:1). **^{1}H NMR** (400 MHz, $CDCl_3$) δ 8.83 (d, J = 5.4 Hz, 1H), 8.71 (dd, J = 6.5, 3.3 Hz, 2H), 8.50 (d, J = 7.9 Hz, 1H), 8.22 (s, 1H), 7.95 (dd, J = 5.4, 2.2 Hz, 1H), 7.88 (td, J = 7.7, 1.7 Hz, 1H), 7.43 – 7.35 (m, 1H), 7.25 (d, J = 3.9 Hz, 1H), 7.21 – 7.12 (m, 4H), 6.93 – 6.80 (m, 4H), 6.44 (d, J = 3.9 Hz, 1H), 3.82 (s, 6H). **^{13}C{^{1}H} NMR** (125 MHz, $CDCl_3$) δ 156.4, 154.9, 151.2, 149.4, 144.7, 144.1, 141.3, 137.3, 125.3, 124.7, 124.3, 121.5, 121.5, 115.4, 115.2, 114.8, 114.0, 110.3, 55.7. **IR** (neat, cm^{-1}): 3115, 2928, 2833, 1587, 1505, 1459, 1242, 1028, 793, 698, 519. **ESI-HRMS**: m/z calcd. for $C_{30}H_{24}N_6O_2SNa$ $[M+Na]^+$ 555.1579, found 555.1574.

3.2. General procedure for synthesis of L2

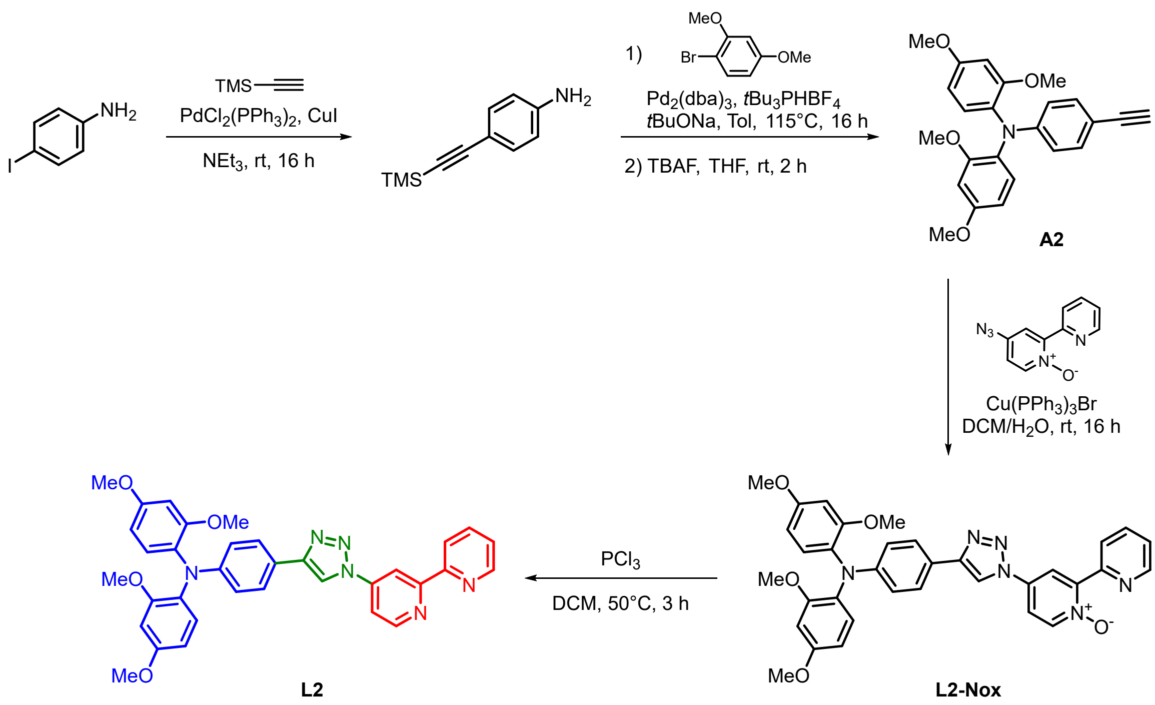

4-((Trimethylsilyl)ethynyl)aniline[15] was prepared according to published methods. The spectral data matched those of the literature.

N-(2,4-dimethoxyphenyl)-*N*-(4-ethynylphenyl)-2,4-dimethoxyaniline (A2)

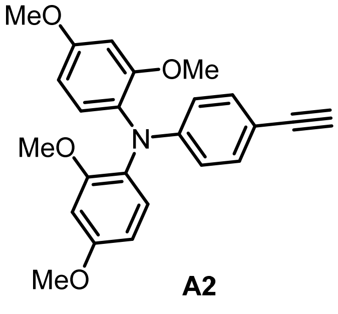

A2

Under inert conditions, 4-((trimethylsilyl)ethynyl)aniline (1.09 g, 5.76 mmol, 1.0 eq.), 2,4-dimethoxybromobenzene (3.12 g, 14.39 mmol, 2.5 eq.), $Pd_2(dba)_3$ (0.158 g, 0.17 mmol, 3 mol%), tBu$_3$PHBF$_4$ (0.100 g, 0.35 mmol, 6 mol%) and NaO*t*Bu (1.66 g, 17.27 mmol, 3.0 eq.) in Toluene (35 mL, 0.17 M) were heated to 110°C for 16 h. The reaction mixture was cooled to rt, filtered over a thin pad of Celite (EtOAc used as solvent) and the filtrate is concentrated in vacuo. The residue was purified by flash column chromatography on silica gel (PE:EtOAc, 6:1) to afford the intermediate product as a yellow solid. The intermediate was dissolved in THF (30 mL, 0.05 M) and a 1 M solution of TBAF (3.5 mL) was added dropwise on an ice bath. The reaction mixture was stirred for 2 h at rt and the solvent

was later evaporated under reduced pressure. The residue is partitioned between H_2O and Et_2O, the organic phase is washed with H_2O/brine, dried over Na_2SO_4 and the solvent was removed under reduced pressure. The alkyne **A2** was obtained without further purification as a brown solid: 1.07 g, 2.75 mmol, 46% over 2 steps. **^{1}H NMR** (400 MHz, CD_2Cl_2) δ 7.24 (d, J = 8.6 Hz, 2H), 7.20 – 7.14 (m, 2H), 6.56 (d, J = 2.7 Hz, 2H), 6.48 (dd, J = 8.6, 2.7 Hz, 2H), 6.33 – 6.28 (m, 2H), 3.80 (s, 6H), 3.76 (s, 6H), 2.98 (s, 1H). **^{13}C{^{1}H} NMR** (100 MHz, CD_2Cl_2) δ 159.8, 157.3, 150.1, 132.9, 131.0, 127.4, 113.7, 109.8, 105.4, 100.2, 85.2, 74.9, 56.0, 55.9. **ESI-HRMS**: m/z calcd. for $C_{24}H_{23}NO_4Na$ $[M+Na]^+$ 412.1519, found 412.1519.

4-(4-(4-(Bis(2,4-dimethoxyphenyl)amino)phenyl)-1H-1,2,3-triazol-1-yl)-[2,2'-bipyridine] 1-oxide (L2-Nox)

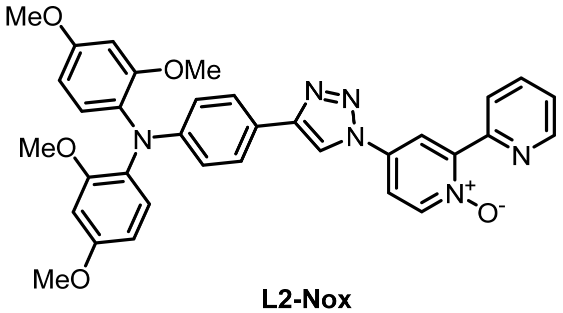

L2-Nox

To a degassed solution of DCM/H_2O (10 mL, 1:1, 0.10 M), 4-azido-[2,2'-bipyridine]-1-oxide (0.256 g, 1.20 mmol, 1.2 eq.), **A2** (0.390 g, 1.00 mmol, 1.0 eq.) and $Cu(PPh_3)_3Br$ (0.186 g, 0.20 mmol, 10 mol%) were added sequentially under inert atmosphere. The reaction mixture was stirred for 16 h at rt and later diluted with DCM. The organic phase was washed with H_2O/brine, dried over Na_2SO_4 and the solvent was removed under reduced pressure. The crude was purified by flash column chromatography on silica gel (DCM:MeOH, 98:2 to 96:4) to afford compound **L2-Nox** as a yellow solid: 0.507 g, 0.84 mmol, 84%. **^{1}H NMR** (400 MHz, CD_2Cl_2) δ 9.06 (d, J = 8.1 Hz, 1H), 8.76 (s, 1H), 8.65 (d, J = 3.2 Hz, 1H), 8.34 (s, 1H), 8.21 (s, 1H), 7.95 – 7.76 (m, 2H), 7.66 – 7.55 (m, 2H), 7.42 (s, 1H), 7.28 (d, J = 8.6 Hz, 2H), 6.58 (d, J = 2.7 Hz, 2H), 6.54 – 6.37 (m, 4H), 3.81 (s, 6H), 3.78 (s, 6H). **^{13}C{^{1}H} NMR** (100 MHz, CD_2Cl_2) δ 159.7, 157.4, 150.2, 149.9, 149.8, 136.8, 131.0, 127.7, 126.7, 118.7, 116.3, 115.7, 114.3, 105.4, 100.2, 56.0, 55.9. **IR** (neat, cm^{-1}): 2932, 2833, 1614, 1581, 1455, 1238, 1204, 1155, 1025, 823, 670. **ESI-HRMS**: m/z calcd. for $C_{34}H_{30}N_6O_5Na$ $[M+Na]^+$ 625.2170, found 625.2164.

***N*-(4-(1-([2,2'-bipyridin]-4-yl)-1*H*-1,2,3-triazol-4-yl)phenyl)-*N*-(2,4-dimethoxyphenyl)-2,4-dimethoxyaniline (L2)**

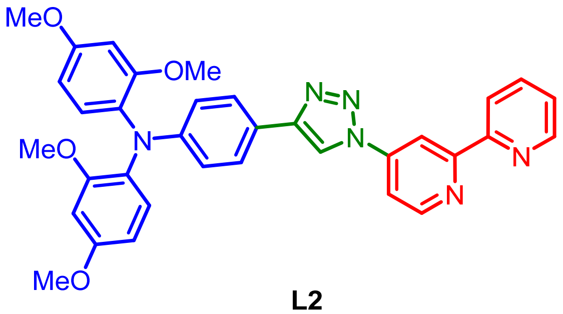

L2

To a solution of **L2-Nox** (0.500 g, 0.83 mmol, 1.0 eq.) in DCM (17 mL, 0.05 M), PCl_3 (0.342 g, 0.22 mL, 2.49 mmol, 3.0 eq.) was added at 0°C under inert atmosphere. The reaction mixture was refluxed for 3 h, then diluted with ice-cold H_2O and basified with 38% m/m NaOH solution. The aqueous layer was extracted with DCM, the combined organic phases are washed with H_2O/brine, dried over Na_2SO_4 and the solvent was removed under reduced pressure. Compound **L2** was obtained without further purification as a yellow solid: 0.368 g, 0.62 mmol, 75%. If needed, **L2** can be purified by column chromatography on silica (DCM:MeOH, 99:1). **^{1}H NMR** (400 MHz, CD_2Cl_2) δ 8.79 (d, J = 6.2 Hz, 2H), 8.70 (d, J = 4.3 Hz, 1H), 8.51 (d, J = 8.0 Hz, 1H), 8.33 (s, 1H), 7.95 – 7.82 (m, 2H), 7.68 – 7.58 (m, 2H), 7.45 – 7.33 (m, 1H), 7.29 (d, J = 8.6 Hz, 2H), 6.59 (d, J = 2.7 Hz, 2H), 6.54 – 6.42 (m, 4H), 3.82 (s, 6H), 3.79 (s, 6H). **^{13}C{^{1}H} NMR** (100 MHz, CD_2Cl_2) δ 159.7, 157.4, 151.3, 150.2, 149.7, 149.6, 144.6, 137.5, 131.0, 127.7, 126.7, 124.9, 121.5, 118.9, 115.7, 114.4, 114.0, 110.5, 105.4, 100.3, 56.0, 55.9. **IR** (neat, cm^{-1}): 3095, 3004, 2939, 2839, 1582, 1562, 1507, 1461, 1408, 1336, 1299, 1245, 1205, 1108, 1029, 937, 858, 819, 790, 744, 707, 637, 571, 502. **ESI-HRMS**: m/z calcd. for $C_{34}H_{30}N_6O_4Na$ $[M+Na]^+$ 609.2221, found 609.2219.

3.3. General procedure for synthesis of L3

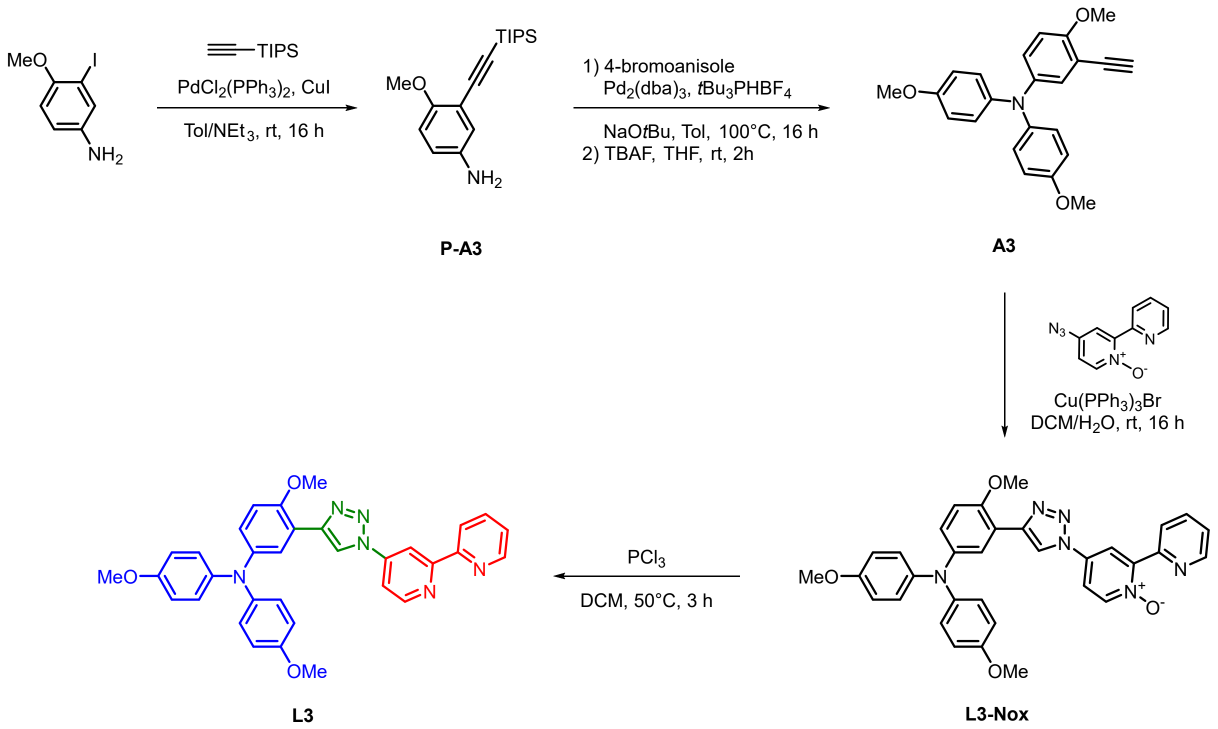

4-Methoxy-3-((triisopropylsilyl)ethynyl)aniline (P-A3)

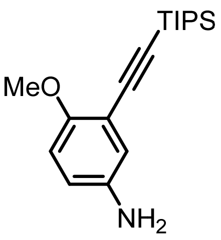

P-A3

Under inert conditions, to a suspension of 3-iodo-4-methoxyaniline (4.981 g, 20.0 mmol, 1.0 eq.), $PdCl_2(PPh_3)_2$ (0.562 g, 0.8 mmol, 4 mol%), CuI (0.076 g, 0.4 mmol, 2 mol%) in Tol/Et_3N (30 mL, 1:1, 0.6 M), ethynyltriisopropylsilane (5.85 mL, 26.0 mmol, 1.3 eq.) is added and the resulting mixture is stirred at rt for 16 h. The reaction mixture is partitioned between H_2O and Et_2O, the organic phase is washed with NH_3/brine, dried over Na_2SO_4 and the solvent was removed under reduced pressure. The protected alkyne **P-A3** was obtained after column chromatography (PE:EtOAc, 4:1) as a red wax: 5.398 g, 17.8 mmol, 89%. **^{1}H NMR** (400 MHz, $CDCl_3$) δ 6.81 (d, J = 2.6 Hz, 1H), 6.72 (d, J = 8.6 Hz, 1H), 6.63 (d, J = 7.8 Hz, 1H), 3.81 (s, 3H), 1.14 (s, 21H). **^{13}C{^{1}H} NMR** (100 MHz, $CDCl_3$) δ 154.6, 120.8, 117.1, 113.9, 113.4, 103.4, 94.7, 57.0, 18.8, 11.5.

3-Ethynyl-4-methoxy-*N*,*N*-bis(4-methoxyphenyl)aniline (A3)

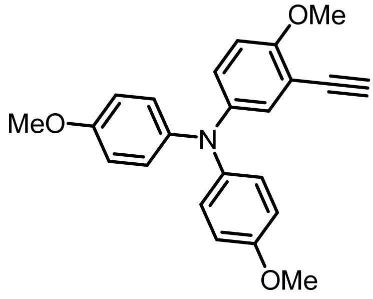

A3

Under inert conditions, **P-A3** (2.50 g, 8.24 mmol, 1.0 eq.), 4-bromoanisole (2.58 mL, 20.59 mmol, 2.5 eq.), $Pd_2(dba)_3$ (0.226 g, 0.25 mmol, 3 mol%), *t*Bu_3PHBF_4 (0.143 g, 0.50 mmol, 6 mol%) and NaO*t*Bu (2.37 g, 24.71 mmol, 3.0 eq.) in Toluene (48 mL, 0.17 M) were heated to 110°C for 16 h. The reaction mixture was cooled to rt, filtered over a thin pad of Celite (EtOAc used as solvent) and the filtrate is concentrated in vacuo. The residue was purified by flash column chromatography on silica gel (PE:EtOAc, 20:1) to afford the intermediate product as a yellow solid. The intermediate was dissolved in THF (60 mL, 0.05 M) and a 1 M solution of TBAF (8.0 mL) was added dropwise on an ice bath. The reaction mixture was stirred for 2 h at rt and the solvent was later evaporated under reduced pressure. The residue is partitioned between H_2O and Et_2O, the organic phase is washed with H_2O/brine, dried over Na_2SO_4 and the solvent was removed under reduced pressure. The residue was purified by flash column chromatography on silica gel (PE:EtOAc, 9:1) to afford alkyne **A3** as a brown oil: 2.52 g, 7.17 mmol, 87% over 2 steps. **^{1}H NMR** (400 MHz, $CDCl_3$) δ 7.14 (d, J = 2.9 Hz, 1H), 7.04 – 6.91 (m, 5H), 6.87 – 6.77 (m, 4H), 6.76 (d, J = 9.0 Hz, 1H), 3.87 (s, 3H), 3.79 (s, 6H), 3.26 (s, 1H). **^{13}C{^{1}H} NMR** (100 MHz, $CDCl_3$) δ 155.9, 155.4, 142.0, 141.6, 128.6, 125.3, 125.1, 124.0, 114.7, 114.4, 111.7, 111.7, 81.2, 80.1, 60.5, 56.3, 55.6. **ESI-HRMS**: m/z calcd. for $C_{23}H_{21}NO_3$ $[M]^+$ 359.1521, found 359.1514.

4-(4-(5-(Bis(4-methoxyphenyl)amino)-2-methoxyphenyl)-1*H*-1,2,3-triazol-1-yl)-[2,2'-bipyridine] 1-oxide (L3-Nox)

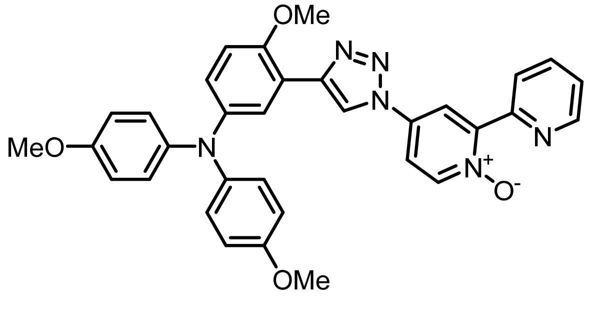

L3-Nox

To a degassed solution of DCM/H_2O (10 mL, 1:1, 0.10 M), 4-azido-[2,2'-bipyridine]-1-oxide (0.213 g, 1.00 mmol, 1.2 eq.), **A3** (0.300 g, 0.83 mmol, 1.0 eq.) and $Cu(PPh_3)_3Br$ (0.155 g, 0.17 mmol, 10 mol%) were added sequentially under inert atmosphere. The reaction mixture was stirred for 16 h at rt and later diluted with DCM. The organic phase was washed with H_2O/brine, dried over Na_2SO_4 and the solvent was removed under reduced pressure. The crude was purified by flash column chromatography on silica gel (DCM:MeOH, 98:2) to afford compound **L3-Nox** as a yellow solid: 0.388 g, 0.74 mmol, 89%. **^{1}H NMR** (400 MHz, CD_2Cl_2) δ 9.07 (d, J = 8.1 Hz, 1H), 8.82 (s, 1H), 8.71 (d, J = 3.2 Hz, 1H), 8.66 (s, 1H), 8.40 (d, J = 7.0 Hz, 1H), 8.05 (d, J = 2.8 Hz, 1H), 7.97 – 7.81 (m, 2H), 7.47 (d, J = 7.0 Hz, 1H), 7.11 – 7.02 (m, 5H), 6.97 (d, J = 8.9 Hz, 1H), 6.92 – 6.73 (m, 4H), 4.00 (s, 3H), 3.82 (s, 6H). **^{13}C{^{1}H} NMR** (100 MHz, CD_2Cl_2) δ 155.7, 151.7, 149.9, 144.6, 142.8, 142.1, 136.8, 125.5, 124.7, 122.7, 120.7, 119.4, 118.0, 116.6, 114.9, 112.4, 56.2, 55.8. **IR** (neat, cm^{-1}): 3044, 2931, 2832, 2584, 1495, 1233, 1023, 813, 578. **ESI-HRMS**: m/z calcd. for $C_{33}H_{28}N_6O_4$ $[M]^+$ 572.2172, found 572.2172.

3-(1-([2,2'-Bipyridin]-4-yl)-1*H*-1,2,3-triazol-4-yl)-4-methoxy-*N*,*N*-bis(4-methoxyphenyl)aniline (L3)

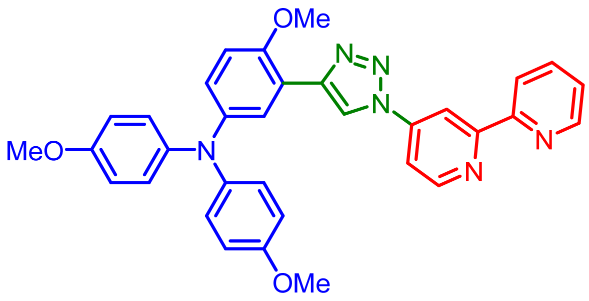

L3

To a solution of **L3-Nox** (0.380 g, 0.66 mmol, 1.0 eq.) in DCM (13 mL, 0.05 M), PCl_3 (0.273 g, 0.17 mL, 1.99 mmol, 3.0 eq.) was added at 0°C under inert atmosphere. The reaction mixture was refluxed for 3 h, then diluted with ice-cold H_2O and basified with 38% m/m NaOH solution. The aqueous layer was extracted with DCM, the combined organic phases are washed with H_2O/brine, dried over Na_2SO_4 and the solvent was removed under reduced pressure. Compound **L3** was obtained without further purification as a yellow solid: 0.282 g, 0.50 mmol, 76%. If needed, **L3** can be purified by column chromatography on silica (DCM:MeOH, 99:1). **^{1}H NMR** (400 MHz, CD_2Cl_2) δ 8.84 (d, J = 2.2 Hz, 1H), 8.80 (d, J = 5.4 Hz, 1H), 8.77 – 8.64 (m, 2H), 8.51 (dt, J = 8.0, 1.1 Hz, 1H), 8.04 (s, 1H), 7.96 – 7.80 (m, 2H), 7.39 (ddd, J = 7.5, 4.8, 1.2 Hz, 1H), 7.28 – 6.89 (m, 6H), 6.89 – 6.68 (m, 4H), 3.99 (s, 3H), 3.78 (s, 6H). **^{13}C{^{1}H} NMR** (100 MHz, CD_2Cl_2) δ 158.7, 155.3, 151.3, 149.6, 144.7, 144.4, 137.5, 125.5, 124.9, 121.6, 120.8, 114.9, 114.7, 114.3, 112.3, 110.8, 56.2, 55.8. **IR** (neat, cm^{-1}): 3036, 2932, 2837, 1585, 1562, 1498, 1237, 1021, 831, 804, 790, 723, 703, 618, 580, 523. **ESI-HRMS**: m/z calcd. for $C_{33}H_{28}N_6O_3Na$ $[M+Na]^+$ 579.2121, found 579.2116.

4. General Procedure for the Synthesis of PC

4.1. General procedure for synthesis of PC2

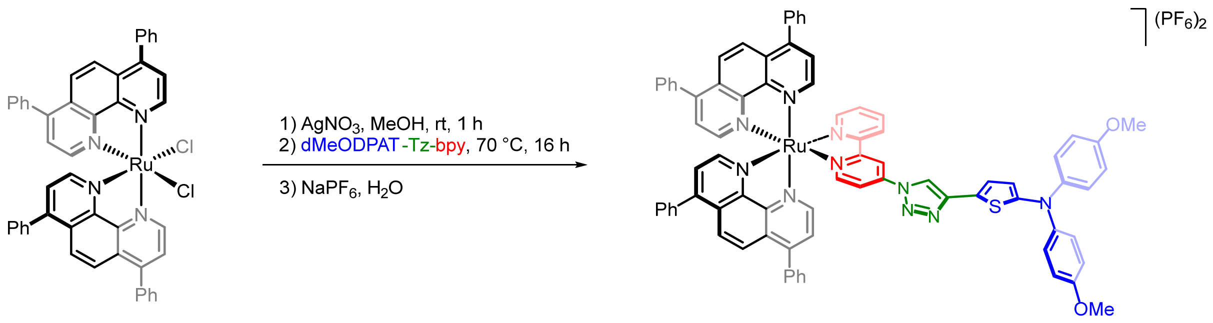

PC2

Cis-dichlorobis(4,7-diphenyl-1,10-phenanthroline)ruthenium (64.8 mg 77.0 µmol, 1.0 eq.) was mixed with $AgNO_3$ (26.3 mg, 155.0 µmol, 2.0 eq.) in MeOH (5 mL, 0.015 M) and the reaction mixture was stirred for 1 h at rt. The resulting suspension was filtered and then mixed with **L1** (45.3 mg, 85.0 µmol, 1.1 eq.). The mixture was heated at reflux for 16 h and later it was cooled down to rt. The solvent was evaporated under reduced pressure and the resulting solid was dissolved in the minimum amount of MeOH. The product is precipitated by addition of a $NaPF_6$ solution in distilled H_2O, washed sequentially with distilled H_2O/hexane/Et_2O and dried in vacuo. Complex **PC2** was obtained following the general procedure, as a deep orange solid: 0.135 g, 84.8 µmol, 99%. **^{1}H NMR** (400 MHz, Acetone-*d*^{6}) δ 9.30 (d, J = 2.4 Hz, 1H), 9.10 (d, J = 8.1 Hz, 1H), 9.00 (s, 1H), 8.89 (d, J = 5.5 Hz, 1H), 8.69 (dd, J = 14.3, 5.5 Hz, 2H), 8.54 (dd, J = 5.5, 1.2 Hz, 2H), 8.39 – 8.29 (m, 4H), 8.25 (dd, J = 13.3, 6.8 Hz, 2H), 8.02 (dd, J = 6.3, 2.3 Hz, 1H), 7.96 (dd, J = 14.2, 5.5 Hz, 2H), 7.81 (d, J = 5.5 Hz, 1H), 7.75 (dd, J = 5.5, 1.4 Hz, 2H), 7.72 – 7.53 (m, 20H), 7.26 (d, J = 4.0 Hz, 1H), 7.14 (td, J = 6.5, 2.3 Hz, 4H), 6.95 – 6.85 (m, 4H), 6.36 (d, J = 3.9 Hz, 1H), 3.79 (s, 6H). **^{13}C{^{1}H} NMR** (100 MHz, Acetone-*d*^{6}) δ 157.7, 150.1, 149.5, 141.8, 136.7, 130.8, 130.8, 130.6, 130.1, 129.9, 127.3, 127.1, 126.3, 126.0, 115.6, 55.8. **^{19}F{^{1}H} NMR** (377 MHz, Acetone-*d*^{6}) δ -71.29 (d, 12F). **IR** (neat, cm^{-1}): 1614, 1505, 1243, 1027, 830, 700, 556, 493. **ESI-HRMS**: m/z calcd. for $C_{52}H_{42}N_8O_2Ru$ [(M-N$_2$)]$^{2+}$ 635.1645, found 635.1642.

4.2. General procedure for synthesis of PC3

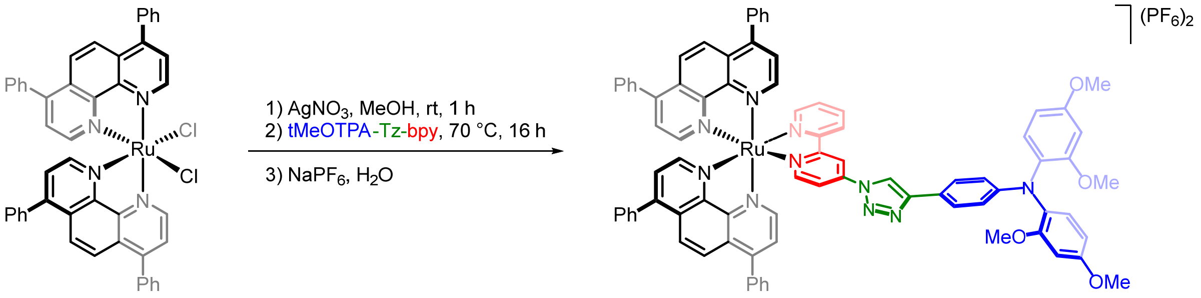

PC3

Cis-dichlorobis(4,7-diphenyl-1,10-phenanthroline)ruthenium (64.8 mg 77.0 µmol, 1.0 eq.) was mixed with $AgNO_3$ (26.3 mg, 155.0 µmol, 2.0 eq.) in MeOH (5 mL, 0.015 M) and the reaction mixture was stirred for 1 h at rt. The resulting suspension was filtered and then mixed with **L2** (50.0 mg, 85.0 µmol, 1.1 eq.). The mixture was heated at reflux for 16 h and later it was cooled down to rt. The solvent was evaporated under reduced pressure and the resulting solid was dissolved in the minimum amount of MeOH. The product is precipitated by addition of a $NaPF_6$ solution in distilled H_2O, washed sequentially with distilled H_2O/hexane/Et_2O and dried in vacuo. Complex **PC2** was obtained following the general procedure, as a deep orange solid: 77.7 mg, 47.3 µmol, 61%. **^{1}H NMR** (400 MHz, Acetone-*d*^{6}) δ 9.41 – 9.22 (m, 1H), 9.11 (d, J = 8.0 Hz, 1H), 9.03 (s, 1H), 8.89 (d, J = 5.5 Hz, 1H), 8.71 (d, J = 5.5 Hz, 1H), 8.54 (dd, J = 5.6, 1.8 Hz, 2H), 8.41 – 8.22 (m, 6H), 8.04 (dd, J = 6.3, 2.2 Hz, 1H), 7.96 (dd, J = 13.3, 5.5 Hz, 2H), 7.84 – 7.53 (m, 25H), 7.40 (d, J = 4.3 Hz, 2H), 7.28 (d, J = 8.6 Hz, 2H), 6.68 (d, J = 2.8 Hz, 2H), 6.53 (dd, J = 8.6, 2.8 Hz, 2H), 6.43 (d, J = 8.3 Hz, 2H), 3.80 (d, J = 12.2 Hz, 12H). **^{13}C{^{1}H} NMR** (100 MHz, Acetone-*d*^{6}) δ 160.5, 158.1, 154.6, 153.5, 150.1, 150.0, 149.5, 139.1, 136.7, 136.6, 131.7, 130.8, 130.8, 130.6, 130.1, 130.1, 129.9, 128.0, 127.3, 127.1, 118.8, 117.7, 115.5, 114.5, 106.2, 100.6, 56.0, 55.8. **^{19}F{^{1}H} NMR** (377 MHz, Acetone-*d*^{6}) δ -71.38 (d, 12F). **IR** (neat, cm^{-1}): 3060, 2942, 2835, 1614, 1506, 1207, 1020, 832, 702, 556. **ESI-HRMS**: m/z calcd. for $C_{82}H_{62}N_{10}O_4Ru$ $[M]^{2+}$ 676.1999, found 676.1995.

4.3. General procedure for synthesis of PC4

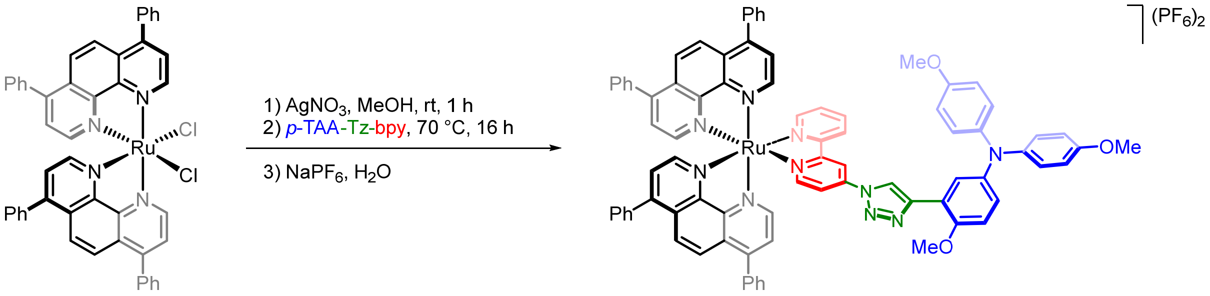

PC4

Cis-dichlorobis(4,7-diphenyl-1,10-phenanthroline)ruthenium (64.8 mg 77.0 µmol, 1.0 eq.) was mixed with $AgNO_3$ (26.3 mg, 155.0 µmol, 2.0 eq.) in MeOH (5 mL, 0.015 M) and the reaction mixture was stirred for 1 h at rt. The resulting suspension was filtered and then mixed with **L3** (50.0 mg, 85.0 µmol, 1.1 eq.). The mixture was heated at reflux for 16 h and later it was cooled down to rt. The solvent was evaporated under reduced pressure and the resulting solid was dissolved in the minimum amount of MeOH. The product is precipitated by addition of a $NaPF_6$ solution in distilled H_2O, washed sequentially with distilled H_2O/hexane/Et_2O and dried in vacuo. Complex **PC4** was obtained following the general procedure, as a deep orange solid: 113.8 mg, 70.8 µmol, 92%. **^{1}H NMR** (400 MHz, Acetone-d^6) δ 9.38 – 6.72 (m, 51H), 4.00 (s, 3H), 3.75 (s, 6H). **^{13}C{^{1}H} NMR** (100 MHz, Acetone-d^6) δ 156.5, 153.6, 152.4, 150.1, 145.2, 142.6, 136.7, 136.6, 130.8, 130.8, 130.7, 130.1, 129.9, 127.3, 127.2, 126.1, 126.0, 118.3, 115.6, 115.5, 55.7. **^{19}F{^{1}H} NMR** (377 MHz, Acetone-d^6) δ -71.36 (d, 12F). **IR** (neat, cm^{-1}): 3056, 2946, 2838, 1615, 1499, 1239, 1021, 832, 765, 702, 556. **ESI-HRMS**: m/z calcd. for $C_{82}H_{60}N_{10}O_3Ru$ $[M]^{2+}$ 661.1947, found 661.1947.

5. General Procedure for the Photoredox Experiments

5.1. General Procedure A

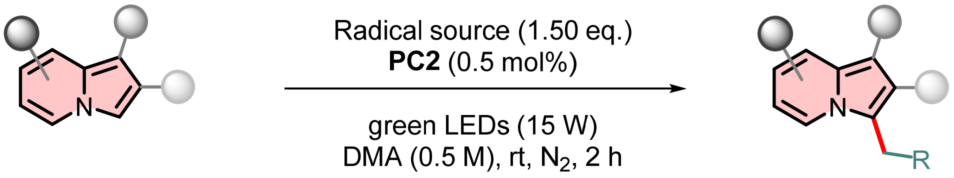

1 2

In a glovebox, to a vial filled with the indolizine **1** (0.20 mmol, 1.00 eq.), radical source **T** (0.30 mmol, 1.50 eq.), **PC2** (1.6 mg, 1.0 µmol, 0.5 mol%) was added DMA (0.4 mL, 0.50 M). The vial was sealed and the mixture was irradiated with green LEDs (15 W, 515 nm) for 2 h at rt outside of the glovebox. The reaction mixture was partitioned between H_2O and Et_2O and the organic phase was extracted. The water phase was extracted with Et_2O 3 times and the combined organic phases were washed with H_2O and brine. The organic phase was dried over Na_2SO_4 and the solvent was removed under reduced pressure. The residue was purified by flash column chromatography on silica gel to afford the functionalized product **2**.

5.2. General Procedure B

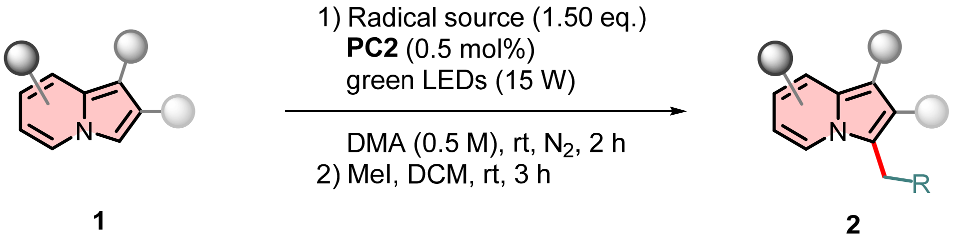

In a glovebox, to a vial filled with the indolizine **1** (0.20 mmol, 1.00 eq.), radical source **T** (0.30 mmol, 1.50 eq.), **PC2** (1.6 mg, 1.0 µmol, 0.5 mol%) was added DMA (0.4 mL, 0.50 M). The vial was sealed and the mixture was irradiated with green LEDs (15 W, 515 nm) for 2 h at rt outside of the glovebox. The reaction mixture was partitioned between H_2O and Et_2O and the organic phase was extracted. The water phase was extracted with Et_2O 3 times and the combined organic phases were washed with H_2O and brine. The organic phase was dried over Na_2SO_4 and the solvent was removed under reduced pressure. The residue was purified by flash column chromatography on silica gel to afford the functionalized product **2**, alongside the byproduct of the reaction, as an inseparable mixture. The mixture is dissolved in DCM/Et_2O (0.25 M, 1:1) and an excess of methyl iodide (0.90 mmol, 3.0 eq.) is added. The resulting mixture is stirred for 3 h at 40°C, after which time the solvent is evaporated in vacuo. The residue is suspended in Et_2O, the precipitated solid is filtered, washed with Et_2O and the filtrate is collected and concentrated in vacuo, yielding the desired product **2**.

5.3. General Procedure C

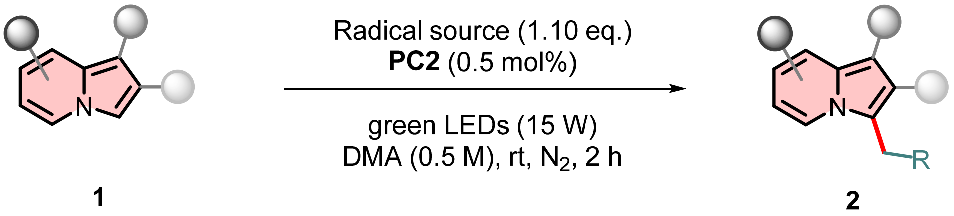

In a glovebox, to a vial filled with the indolizine **1** (0.20 mmol, 1.00 eq.), radical source **T** (0.22 mmol, 1.10 eq.), **PC2** (1.6 mg, 1.0 µmol, 0.5 mol%) was added DMA (0.4 mL, 0.50 M). The vial was sealed and the mixture was irradiated with green LEDs (15 W, 515 nm) for 2 h at rt outside of the glovebox. The reaction mixture was partitioned between H_2O and Et_2O and the organic phase was extracted. The water phase was extracted with Et_2O 3 times and the combined organic phases were washed with H_2O and brine. The organic phase was dried over Na_2SO_4 and the solvent was removed under reduced pressure. The residue was purified by flash column chromatography on silica gel to afford the functionalized product **2**.

5.4. General Procedure D

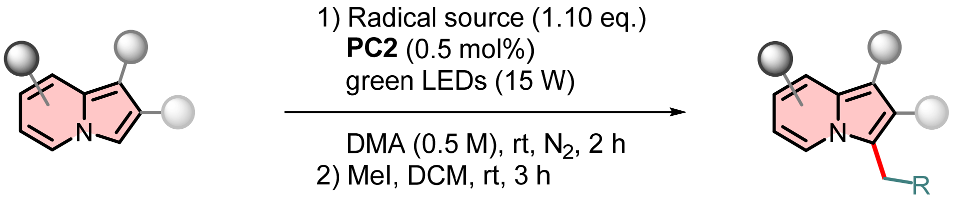

1 **2**

In a glovebox, to a vial filled with the indolizine **1** (0.20 mmol, 1.00 eq.), radical source **T** (0.22 mmol, 1.10 eq.), **PC2** (1.6 mg, 1.0 µmol, 0.5 mol%) was added DMA (0.4 mL, 0.50 M). The vial was sealed and the mixture was irradiated with green LEDs (15 W, 515 nm) for 2 h at rt outside of the glovebox. The reaction mixture was partitioned between H_2O and Et_2O and the organic phase was extracted. The water phase was extracted with Et_2O 3 times and the combined organic phases were washed with H_2O and brine. The organic phase was dried over Na_2SO_4 and the solvent was removed under reduced pressure. The residue was purified by flash column chromatography on silica gel to afford the functionalized product **2**, alongside the byproduct of the reaction, as an inseparable mixture. The mixture is dissolved in DCM/Et_2O (0.25 M, 1:1) and an excess of methyl iodide (0.66 mmol, 3.0 eq.) is added. The resulting mixture is stirred for 3 h at 40°C, after which time the solvent is evaporated in vacuo. The residue is suspended in Et_2O, the precipitated solid is filtered, washed with Et_2O and the filtrate is collected and concentrated in vacuo, yielding the desired product **2**.

Methyl 2-(1-cyano-2-phenylindolizin-3-yl)acetate (2a)

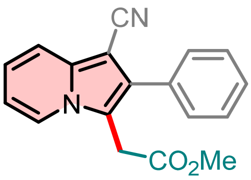

2a

Product **2a** was obtained following the general procedure **A** using 2-(phenyl)indolizine-1-carbonitrile **1a** (43.6 mg, 0.20 mmol, 1.00 eq.) and 2-((2-methoxy-2-oxoethyl)thio)-3-methyl-4,5-dihydrothiazol-3-ium triflate **T1** (106.6 mg, 0.30 mmol, 1.50 eq.). The crude was purified by column chromatography on silica (PE:EtOAc, 4:1) to get the pure product as a brown wax: 40.6 mg, 0.14 mmol, 70%. **^{1}H NMR** (400 MHz, $CDCl_3$) δ 8.01 (d, J = 7.0 Hz, 1H), 7.67 (d, J = 9.0 Hz, 1H), 7.61 – 7.52 (m, 2H), 7.48 (t, J = 7.5 Hz, 2H), 7.44 – 7.37 (m, 1H), 7.11 (dd, J = 8.9, 6.6 Hz, 1H), 6.91 – 6.77 (m, 1H), 3.93 (s, 2H), 3.75 (s, 3H). **^{13}C{^{1}H} NMR** (100 MHz, $CDCl_3$) δ 169.8, 138.2, 132.0, 131.8, 129.7, 129.1, 129.0, 128.3, 127.6, 123.9, 122.6, 117.9, 116.8, 115.2, 113.5, 82.0, 52.7, 30.8. **IR** (neat, cm^{-1}): 3110, 3042, 2947, 2921, 2852, 2213, 1739, 1511, 1434, 1397, 1339, 1314, 1271, 1205, 1154, 992, 738, 698, 657, 550, 491, 456. **ESI-HRMS**: m/z calcd. for $C_{18}H_{14}N_2O_2Na$ $[M+Na]^+$ 313.0947, found 313.0955.

Dimethyl 2,2'-(1-cyano-2-phenylindolizine-3,5-diyl)diacetate (2a')

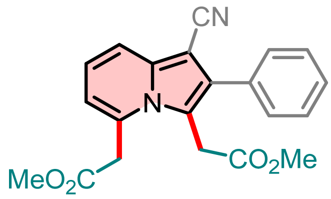

2a'

Product **2a'** was obtained following the general procedure **A** using 2-(phenyl)indolizine-1-carbonitrile **1a** (43.6 mg, 0.20 mmol, 1.00 eq.) and 2-((2-methoxy-2-oxoethyl)thio)-3-methyl-4,5-dihydrothiazol-3-ium triflate **T1** (106.6 mg, 0.30 mmol, 1.50 eq.). The crude was purified by column chromatography on silica (PE:EtOAc, 3:1) to get the pure product as a brown wax: 8.7 mg, 0.02 mmol, 12%. **^{1}H NMR** (400 MHz, $CDCl_3$) δ 7.66 (dd, J = 8.8, 1.4 Hz, 1H), 7.50 – 7.40 (m, 5H), 7.05 (dd, J = 8.9, 6.8 Hz, 1H), 6.68 (dd, J = 6.9, 1.4 Hz, 1H), 4.11 (s, 2H), 4.07 (s, 2H), 3.72 (s, 3H), 3.72 (s, 3H). **^{13}C{^{1}H} NMR** (100 MHz, $CDCl_3$) δ 171.6, 170.0, 140.5, 134.1, 132.3, 131.9, 129.9, 129.8, 128.8, 128.3, 126.9, 122.2, 118.0, 117.7, 117.0, 116.4, 84.1, 52.9, 52.7, 39.1, 32.9. **IR** (neat, cm^{-1}): 2952, 2847, 2206, 1734, 1523, 1435, 1155, 1001, 910, 731, 701. **ESI-HRMS**: m/z calcd. for $C_{21}H_{18}N_2O_4Na$ $[M+Na]^+$ 385.1164, found 385.1159.

Methyl 2-(2-phenylindolizin-3-yl)acetate (2b-1)

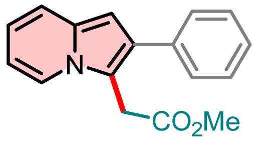

2b-1

Product **2b-1** was obtained following the general procedure **C** using 2-phenylindolizine **1b** (38.6 mg, 0.20 mmol, 1.00 eq.) and 2-((2-methoxy-2-oxoethyl)thio)-3-methyl-4,5-dihydrothiazol-3-ium triflate **T1** (78.2 mg, 0.22 mmol, 1.10 eq.). The crude was purified by column chromatography on silica (PE:EtOAc, 98:2) to get the pure product as a yellowish oil: 36.4 mg, 0.14 mmol, 68%. **^{1}H NMR** (400 MHz, $CDCl_3$) δ 7.91 (dq, J = 7.3, 1.1 Hz, 1H), 7.62 – 7.55 (m, 2H), 7.52 – 7.39 (m, 3H), 7.39 – 7.32 (m, 1H), 6.75 (ddd, J = 8.9, 6.5, 1.1 Hz, 1H), 6.64 – 6.57 (m, 2H), 4.01 (s, 2H), 3.77 (s, 3H). **^{13}C{^{1}H} NMR** (100 MHz, $CDCl_3$) δ 170.9, 136.1, 132.9, 130.2, 129.9, 129.3, 128.7, 128.3, 126.8, 122.5, 119.2, 117.2, 112.8, 110.9, 99.1, 52.5, 31.4. **IR** (neat, cm^{-1}): 3054, 2951, 2852, 1732, 1603, 1433, 1362, 1301, 1156, 1005, 757, 725, 699, 492. **ESI-HRMS**: m/z calcd. for $C_{17}H_{15}NO_2Na$ $[M+Na]^+$ 288.1000, found 288.0995.

Methyl 3-(2-methoxy-2-oxoethyl)-2-phenylindolizine-1-carboxylate (2c-1)

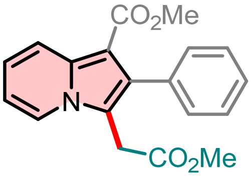

2c-1

Product **2c-1** was obtained following the general procedure **A** using methyl 2-phenylindolizine-1-carboxylate **1c** (50.2 mg, 0.20 mmol, 1.00 eq.) and 2-((2-methoxy-2-oxoethyl)thio)-3-methyl-4,5-dihydrothiazol-3-ium triflate **T1** (106.6 mg, 0.30 mmol, 1.50 eq.). The crude was purified by column chromatography on silica (PE:EtOAc, 5:1) to get the pure product as a colorless oil: 30.3 mg, 0.09 mmol, 47%. Alongside the pure product, a mixed fraction of **2c-1** and its regioisomer, methyl 5-(2-methoxy-2-oxoethyl)-2-phenylindolizine-1-carboxylate **2c-1'**, was isolated: 11.7 mg, 0.04 mmol, 18%, 1.5:1 ratio respectively. **^{1}H NMR** (400 MHz, $CDCl_3$) δ 8.32 (dt, J = 9.1, 1.2 Hz, 1H), 7.95 (dt, J = 7.0, 1.2 Hz, 1H), 7.51 – 7.32 (m, 5H), 7.14 (ddd, J = 9.1, 6.7, 1.1 Hz, 1H), 6.84 (td, J = 6.8, 1.3 Hz, 1H), 3.79 (s, 2H), 3.72 (s, 3H), 3.71 (s, 3H). **^{13}C{^{1}H} NMR** (100 MHz, $CDCl_3$) δ 170.3, 165.4, 136.3, 134.7, 132.0, 130.6, 127.7, 127.3, 123.2, 122.5, 120.4, 116.6, 113.1, 102.0, 52.5, 50.7, 30.7. **IR** (neat, cm^{-1}): 2949, 1735, 1683, 1505, 1437, 1393, 1319, 1173, 1066, 1030, 915, 786, 736, 701, 644. **ESI-HRMS**: m/z calcd. for $C_{19}H_{17}NO_4Na$ $[M+Na]^+$ 346.1055, found 346.1050.

Ethyl 3-(2-methoxy-2-oxoethyl)-2-phenylindolizine-1-carboxylate (2d-1)

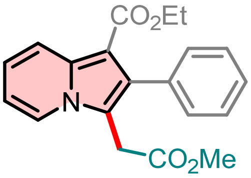

2d-1

Product **2d-1** was obtained following the general procedure **A** using ethyl 2-phenylindolizine-1-carboxylate **1d** (53.1 mg, 0.20 mmol, 1.00 eq.) and 2-((2-methoxy-2-oxoethyl)thio)-3-methyl-4,5-dihydrothiazol-3-ium triflate **T1** (106.6 mg, 0.30 mmol, 1.50 eq.). The crude was purified by column chromatography on silica (PE:EtOAc, 6:1) to get the pure product as a colorless oil: 25.9 mg, 0.07 mmol, 37%. **^{1}H NMR** (400 MHz, $CDCl_3$) δ 8.34 (dt, J = 9.1, 1.3 Hz, 1H), 7.95 (dt, J = 7.0, 1.2 Hz, 1H), 7.56 – 7.30 (m, 5H), 7.13 (ddd, J = 9.1, 6.7, 1.1 Hz, 1H), 6.83 (td, J = 6.8, 1.4 Hz, 1H), 4.18 (q, J = 7.1 Hz, 2H), 3.79 (s, 2H), 3.70 (s, 3H), 1.13 (t, J = 7.1 Hz, 3H). **^{13}C{^{1}H} NMR** (100 MHz, $CDCl_3$) δ 170.3, 165.0, 136.3, 134.8, 131.9, 130.6, 127.7, 127.3, 123.2, 122.4, 120.4, 116.4, 113.0, 102.4, 59.3, 52.5, 30.7, 14.2. **IR** (neat, cm^{-1}): 3055, 2952, 1736, 1678, 1506, 1402, 1316, 1243, 1176, 1065, 1038, 737, 701, 644. **ESI-HRMS**: m/z calcd. for $C_{20}H_{19}NO_4Na$ $[M+Na]^+$ 360.1212, found 360.1206.

Ethyl 5-(2-methoxy-2-oxoethyl)-2-phenylindolizine-1-carboxylate (2d-1')

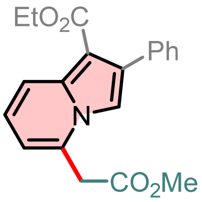

2d-1'

Product **2d-1'** was obtained following the general procedure **A** using ethyl 2-phenylindolizine-1-carboxylate **1d** (53.1 mg, 0.20 mmol, 1.00 eq.) and 2-((2-methoxy-2-oxoethyl)thio)-3-methyl-4,5-dihydrothiazol-3-ium triflate **T1** (106.6 mg, 0.30 mmol, 1.50 eq.). The crude was purified by column chromatography on silica (PE:EtOAc, 6:1) to get the pure product as a colorless oil: 2.0 mg, 0.006 mmol, 3%. **^{1}H NMR** (400 MHz, $CDCl_3$) δ 8.30 (d, J = 9.2 Hz, 1H), 7.57 – 7.48 (m, 2H), 7.39 (dddd, J = 12.9, 7.8, 7.1, 1.3 Hz, 3H), 7.21 (s, 1H), 7.11 (dd, J = 9.2, 6.8 Hz, 1H), 6.73 (dd, J = 6.9, 1.2 Hz, 1H), 4.26 (q, J = 7.1 Hz, 2H), 3.94 (s, 2H), 3.73 (s, 3H), 1.25 – 1.21 (m, 3H). **^{13}C{^{1}H} NMR** (100 MHz, $CDCl_3$) δ 169.0, 135.2, 133.2, 130.2, 127.7, 127.3, 122.5, 119.7, 114.3, 111.1, 59.6, 52.9, 38.7, 29.9, 14.4. **IR** (neat, cm^{-1}): 2927, 2853, 1737, 1680, 1512, 1423, 1297, 1253, 1154, 1073, 741, 698, 663. **ESI-HRMS**: m/z calcd. for $C_{20}H_{19}NO_4Na$ $[M+Na]^+$ 360.1212, found 360.1206.

Ethyl 2-(1-cyano-2-phenylindolizin-3-yl)acetate (2a-2)

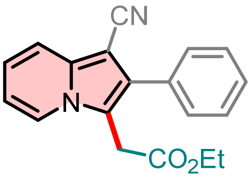

2a-2

Product **2a-2** was obtained following the general procedure **A** using 2-(phenyl)indolizine-1-carbonitrile **1a** (43.6 mg, 0.20 mmol, 1.00 eq.) and 2-((2-ethoxy-2-oxoethyl)thio)-3-methyl-4,5-dihydrothiazol-3-ium triflate **T2** (110.8 mg, 0.30 mmol, 1.50 eq.). The crude was purified by column chromatography on silica (PE:EtOAc, 4:1) to get the pure product as a colorless oil: 39.0 mg, 0.13 mmol, 64%. **^{1}H NMR** (400 MHz, $CDCl_3$) δ 8.05 (dt, J = 7.1, 1.0 Hz, 1H), 7.70 (dt, J = 9.0, 1.2 Hz, 1H), 7.64 – 7.54 (m, 2H), 7.55 – 7.47 (m, 2H), 7.46 – 7.39 (m, 1H), 7.14 (ddd, J = 8.9, 6.7, 1.0 Hz, 1H), 6.86 (td, J = 6.9, 1.3 Hz, 1H), 4.23 (q, J = 7.1 Hz, 2H), 3.92 (s, 2H), 1.29 (t, J = 7.2 Hz, 3H). **^{13}C{^{1}H} NMR** (100 MHz, $CDCl_3$) δ 169.4, 138.2, 132.1, 131.9, 129.7, 129.1, 129.0, 128.3, 127.6, 123.9, 122.6, 117.9, 116.8, 115.4, 113.5, 82.0, 61.8, 31.2, 14.3. **IR** (neat, cm^{-1}): 2990, 2924, 2853, 2207, 1715, 1511, 1449, 1331, 1312, 1263, 1192, 1025, 874, 741, 707, 492, 452. **ESI-HRMS**: m/z calcd. for $C_{19}H_{16}N_2O_2Na$ $[M+Na]^+$ 327.1109, found 327.1104.

Ethyl 2-(2-phenylindolizin-3-yl)acetate (2b-2)

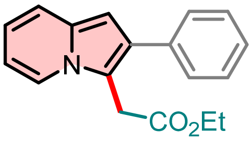

2b-2

Product **2b-2** was obtained following the general procedure **C** using 2-phenylindolizine **1b** (38.6 mg, 0.20 mmol, 1.00 eq.) and 2-((2-ethoxy-2-oxoethyl)thio)-3-methyl-4,5-dihydrothiazol-3-ium triflate **T2** (81.2 mg, 0.22 mmol, 1.10 eq.). The crude was purified by column chromatography on silica (PE:EtOAc, 95:5) to get the pure product as a yellowish oil: 40.0 mg, 0.14 mmol, 72%. ^{1}**H NMR** (400 MHz, $CDCl_3$) δ 7.95 (dt, J = 7.0, 1.1 Hz, 1H), 7.65 – 7.58 (m, 2H), 7.51 – 7.39 (m, 3H), 7.39 – 7.32 (m, 1H), 6.80 – 6.71 (m, 1H), 6.65 – 6.57 (m, 2H), 4.24 (q, J = 7.2 Hz, 2H), 3.99 (s, 2H), 1.29 (t, J = 7.2 Hz, 3H). ^{13}**C{^{1}H} NMR** (100 MHz, $CDCl_3$) δ 170.3, 136.2, 132.8, 130.3, 129.9, 129.3, 128.7, 128.2, 126.8, 122.6, 119.1, 117.2, 113.0, 110.8, 99.1, 61.4, 31.6, 14.3. **IR** (neat, cm^{-1}): 3055, 2979, 2907, 1729, 1603, 1452, 1362, 1302, 1157, 1029, 759, 727, 701. **ESI-HRMS**: m/z calcd. for $C_{18}H_{17}NO_2Na$ $[M+Na]^+$ 302.1157, found 302.1151.

Methyl 3-(2-ethoxy-2-oxoethyl)-2-phenylindolizine-1-carboxylate (2c-2)

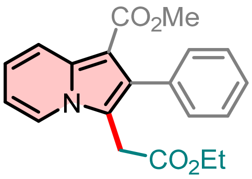

2c-2

Product **2c-2** was obtained following the general procedure **A** using methyl 2-phenylindolizine-1-carboxylate **1c** (50.2 mg, 0.20 mmol, 1.00 eq.) and 2-((2-ethoxy-2-oxoethyl)thio)-3-methyl-4,5-dihydrothiazol-3-ium triflate **T2** (110.8 mg, 0.30 mmol, 1.50 eq.). The crude was purified by column chromatography on silica (PE:EtOAc, 5:1) to get the pure product as a colorless oil: 19.0 mg, 0.06 mmol, 28%. Alongside the pure product, a mixed fraction of **2c-2** and its regioisomer, methyl 5-(2-ethoxy-2-oxoethyl)-2-phenylindolizine-1-carboxylate **2c-2'**, was isolated: 16.1 mg, 0.05 mmol, 24%, 2:1 ratio respectively. ^{1}**H NMR** (400 MHz, $CDCl_3$) δ 8.31 (dt, J = 9.2, 1.2 Hz, 1H), 7.98 (dt, J = 7.0, 1.1 Hz, 1H), 7.46 – 7.35 (m, 5H), 7.14 (ddd, J = 9.1, 6.7, 1.1 Hz, 1H), 6.83 (td, J = 6.8, 1.3 Hz, 1H), 4.17 (q, J = 7.1 Hz, 2H), 3.77 (s, 2H), 3.72 (s, 3H), 1.24 (t, J = 7.1 Hz, 3H). ^{13}**C{^{1}H} NMR** (100 MHz, $CDCl_3$) δ 169.8, 165.4, 136.3, 134.7, 131.9, 130.6, 127.8, 127.7, 127.3, 123.3, 122.5, 120.4, 116.8, 113.0, 102.0, 61.5, 50.7, 31.0, 14.3. **IR** (neat, cm^{-1}): 2982, 2947, 1731, 1683, 1505, 1439, 1392, 1332, 1127, 1065, 1018, 915, 785, 769, 734, 699, 642, 499. **ESI-HRMS**: m/z calcd. for $C_{20}H_{19}NO_4Na$ $[M+Na]^+$ 360.1212, found 360.1206.

Ethyl 3-(2-ethoxy-2-oxoethyl)-2-phenylindolizine-1-carboxylate (2d-2)

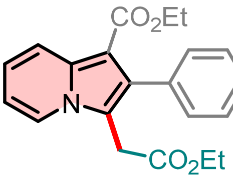

2d-2

Product **2d-2** was obtained following the general procedure **A** using ethyl 2-phenylindolizine-1-carboxylate **1d** (53.1 mg, 0.20 mmol, 1.00 eq.) and 2-((2-ethoxy-2-oxoethyl)thio)-3-methyl-4,5-dihydrothiazol-3-ium triflate **T2** (110.8 mg, 0.30 mmol, 1.50 eq.). The crude was purified by column chromatography on silica (PE:EtOAc, 7:1) to get the pure product as a colorless oil: 21.6 mg, 0.06 mmol, 31%. Alongside the pure product, a mixed fraction of **2d-2** and its regioisomer, ethyl 5-(2-ethoxy-2-oxoethyl)-2-phenylindolizine-1-carboxylate **2d-2'**, was isolated: 20.0 mg, 0.06 mmol, 28%, 2:1 ratio respectively. Following the general procedure **C** using ethyl 2-phenylindolizine-1-carboxylate **1d** (53.1 mg, 0.20 mmol, 1.00 eq.) and 2-((2-ethoxy-2-oxoethyl)thio)-3-methyl-4,5-dihydrothiazol-3-ium triflate **T2** (81.2 mg, 0.22 mmol, 1.10 eq.), the pure product was obtained as a colorless oil: 29.6 mg, 0.08 mmol, 42%. **^{1}H NMR** (400 MHz, $CDCl_3$) δ 8.33 (dt, J = 9.1, 1.2 Hz, 1H), 7.98 (dt, J = 7.0, 1.1 Hz, 1H), 7.46 – 7.31 (m, 5H), 7.13 (ddd, J = 9.1, 6.7, 1.1 Hz, 1H), 6.83 (td, J = 6.8, 1.3 Hz, 1H), 4.17 (q, J = 7.1 Hz, 4H), 3.77 (s, 2H), 1.24 (t, J = 7.1 Hz, 3H), 1.13 (t, J = 7.1 Hz, 3H). **^{13}C{^{1}H} NMR** (100 MHz, $CDCl_3$) δ 169.8, 165.0, 136.3, 134.9, 131.9, 130.7, 127.6, 127.2, 123.2, 122.4, 120.3, 116.7, 112.9, 102.3, 61.5, 59.3, 31.0, 14.2. **IR** (neat, cm^{-1}): 2979, 2930, 1731, 1678, 1506, 1402, 1316, 1243, 1179, 1065, 1028, 738, 700. **ESI-HRMS**: m/z calcd. for $C_{21}H_{21}NO_4Na$ $[M+Na]^+$ 374.1368, found 374.1363.

Ethyl 5-(2-ethoxy-2-oxoethyl)-2-phenylindolizine-1-carboxylate (2d-2')

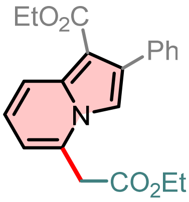

2d-2'

Product **2d-2'** was obtained following the general procedure **A** using ethyl 2-phenylindolizine-1-carboxylate **1d** (53.1 mg, 0.20 mmol, 1.00 eq.) and 2-((2-ethoxy-2-oxoethyl)thio)-3-methyl-4,5-dihydrothiazol-3-ium triflate **T2** (106.6 mg, 0.30 mmol, 1.50 eq.). The crude was purified by column chromatography on silica (PE:EtOAc, 6:1) to get the pure product as a colorless oil: 2.0 mg, 0.006 mmol, 3%. **^{1}H NMR** (400 MHz, $CDCl_3$) δ 8.29 (d, J = 9.1 Hz, 1H), 7.52 – 7.50 (m, 2H), 7.37 (ddd, J = 12.7, 7.8, 6.2 Hz, 3H), 7.21 (s, 1H), 7.09 (d, J = 6.9 Hz, 1H), 6.73 (dd, J = 6.8, 1.2 Hz, 1H), 4.25 (q, J = 7.1 Hz, 2H), 4.22 – 4.16 (m, 2H), 3.91 (s, 2H), 1.24 – 1.20 (m, 6H).**^{13}C{^{1}H} NMR** (100 MHz, $CDCl_3$) δ

168.5, 165.1, 137.7, 135.2, 133.2, 130.2, 130.1, 127.7, 127.2, 122.5, 119.6, 114.2, 111.2, 102.6, 61.9, 59.5, 39.0, 14.4, 14.3. **IR** (neat, cm^{-1}): 2979, 2929, 2853, 1733, 1680, 1511, 1422, 1251, 1153, 1073, 1025, 741, 699. **ESI-HRMS**: m/z calcd. for $C_{21}H_{21}NO_4Na$ $[M+Na]^+$ 374.1368, found 374.1363.

Ethyl 2-(1-cyano-2-phenylindolizin-3-yl)propanoate (2a-3)

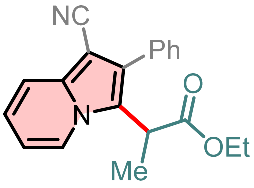

2a-3

Product **2a-3** was obtained following the general procedure **A** using 2-(phenyl)indolizine-1-carbonitrile **1a** (43.6 mg, 0.20 mmol, 1.00 eq.) and 2-((1-ethoxy-1-oxopropan-2-yl)thio)-3-methyl-4,5-dihydrothiazol-3-ium triflate **T3** (115.0 mg, 0.30 mmol, 1.50 eq.). The crude was purified by column chromatography on silica (PE:EtOAc, 6:1) to get the pure product as a colorless oil: 9.0 mg, 0.03 mmol, 14%. **^{1}H NMR** (400 MHz, $CDCl_3$) δ 8.10 (dt, J = 7.1, 1.1 Hz, 1H), 7.70 (dt, J = 8.9, 1.2 Hz, 1H), 7.57 – 7.41 (m, 5H), 7.11 (ddd, J = 9.0, 6.7, 1.0 Hz, 1H), 6.81 (td, J = 6.9, 1.3 Hz, 1H), 4.38 – 4.33 (m, 1H), 4.22 – 4.13 (m, 2H), 1.55 (dd, J = 7.4, 1.1 Hz, 3H), 1.19 (td, J = 7.1, 1.1 Hz, 3H). **^{13}C{^{1}H} NMR** (100 MHz, $CDCl_3$) δ 172.5, 138.2, 132.5, 131.2, 129.9, 129.2, 128.9, 128.3, 127.6, 124.8, 122.3, 121.0, 118.3, 116.8, 113.3, 82.6, 61.8, 36.4, 14.2, 14.2. **IR** (neat, cm^{-1}): 2982, 2938, 2205, 1726, 1511, 1448, 1393, 1316, 1188, 1099, 1016, 742, 700, 495. **ESI-HRMS**: m/z calcd. for $C_{20}H_{18}N_2O_2Na$ $[M+Na]^+$ 341.1266, found 341.1260.

Ethyl 2-(2-phenylindolizin-3-yl)propanoate (2b-3)

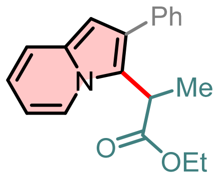

2b-3

Product **2b-3** was obtained following the general procedure **C** using 2-phenylindolizine **1b** (38.6 mg, 0.20 mmol, 1.00 eq.) and 2-((1-ethoxy-1-oxopropan-2-yl)thio)-3-methyl-4,5-dihydrothiazol-3-ium triflate **T3** (84.4 mg, 0.22 mmol, 1.10 eq.). The crude was purified by column chromatography on silica (PE:EtOAc, 99:1) to get the pure product as a colorless oil: 29.7 mg, 0.10 mmol, 51%. **^{1}H NMR** (400 MHz, $CDCl_3$) δ 8.03 – 7.96 (m, 1H), 7.60 – 7.52 (m, 2H), 7.51 – 7.40 (m, 3H), 7.39 – 7.33 (m, 1H), 6.71 (ddd, J = 9.0, 6.5, 1.0 Hz, 1H), 6.58 – 6.50 (m, 2H), 4.45 (q, J = 7.3 Hz, 1H), 4.26 – 4.03 (m, 2H), 1.58 (dd, J = 7.4, 1.1 Hz, 3H), 1.20 (t, J = 7.1 Hz, 3H). **^{13}C{^{1}H} NMR** (100 MHz, $CDCl_3$) δ 173.4, 136.7, 132.7, 129.6, 129.2, 128.9, 128.5, 128.2, 126.8, 123.4, 119.4, 118.7, 116.8, 110.5, 99.7, 61.3, 36.6, 14.3, 14.1. **IR** (neat, cm^{-1}): 3055, 2980, 2936, 1724, 1448, 1362, 1298, 1094, 757, 726, 699. **ESI-HRMS**: m/z calcd. for $C_{19}H_{19}NO_2Na$ $[M+Na]^+$ 316.1313, found 316.1308.

3-(2-Oxopropyl)-2-phenylindolizine-1-carbonitrile (2a-4)

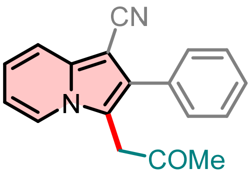

2a-4

Product **2a-4** was obtained following the general procedure **A** using 2-(phenyl)indolizine-1-carbonitrile **1a** (43.6 mg, 0.20 mmol, 1.00 eq.) and 3-methyl-2-((2-oxopropyl)thio)-4,5-dihydrothiazol-3-ium triflate **T4** (101.8 mg, 0.30 mmol, 1.50 eq.). The crude was purified by column chromatography on silica (PE:EtOAc, 3:1 to 2:1) to get the pure product as an off-white wax: 29.0 mg, 0.11 mmol, 53%. **^{1}H NMR** (400 MHz, $CDCl_3$) δ 7.81 (dt, J = 7.0, 1.1 Hz, 1H), 7.69 (dt, J = 8.9, 1.2 Hz, 1H), 7.63 – 7.37 (m, 5H), 7.13 (ddd, J = 9.0, 6.8, 1.0 Hz, 1H), 6.83 (td, J = 6.9, 1.3 Hz, 1H), 4.03 (s, 2H), 2.16 (s, 3H). **^{13}C{^{1}H} NMR** (100 MHz, $CDCl_3$) δ 203.8, 138.2, 132.2, 131.7, 129.4, 129.0, 128.3, 123.7, 122.6, 117.9, 116.6, 115.9, 113.5, 82.2, 39.8, 29.2. **IR** (neat, cm^{-1}): 3114, 3052, 2919, 2209, 1719, 1510, 1393, 1354, 1328, 1154, 1076, 769, 742, 707, 541, 470, 459. **ESI-HRMS**: m/z calcd. for $C_{18}H_{14}N_2ONa$ $[M+Na]^+$ 297.1004, found 297.0998.

3-(2-Oxopropyl)-2-phenylindolizine (2b-4)

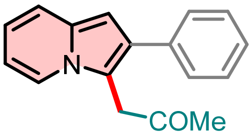

2b-4

Product **2b-4** was obtained following the general procedure **C** using 2-phenylindolizine **1b** (38.6 mg, 0.20 mmol, 1.00 eq.) and 3-methyl-2-((2-oxopropyl)thio)-4,5-dihydrothiazol-3-ium triflate **T4** (74.7 mg, 0.22 mmol, 1.10 eq.). The crude was purified by column chromatography on silica (PE:EtOAc, 9:1) to get the pure product as a colorless oil: 34.4 mg, 0.14 mmol, 69%. **^{1}H NMR** (400 MHz, $CDCl_3$) δ 7.70 (dd, J = 7.1, 1.1 Hz, 1H), 7.49 – 7.40 (m, 5H), 7.39 – 7.32 (m, 1H), 6.74 (ddd, J = 9.0, 6.5, 1.1 Hz, 1H), 6.61 (s, 1H), 6.58 (td, J = 6.7, 1.3 Hz, 1H), 4.07 (s, 2H), 2.06 (s, 3H). **^{13}C{^{1}H} NMR** (100 MHz, $CDCl_3$) δ 205.6, 136.4, 132.9, 130.1, 129.8, 129.2, 128.8, 127.7, 126.9, 122.2, 119.2, 117.3, 113.5, 111.1, 99.5, 40.6, 28.9. **IR** (neat, cm^{-1}): 3052, 2922, 2853, 1711, 1603, 1452, 1357, 1299, 1157, 912, 756, 725, 699, 533. **ESI-HRMS**: m/z calcd. for $C_{17}H_{15}NONa$ $[M+Na]^+$ 272.1051, found 272.1046.

Methyl 3-(2-oxopropyl)-2-phenylindolizine-1-carboxylate (2c-4)

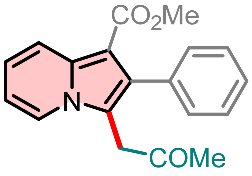

2c-4

Product **2c-4** was obtained following the general procedure **A** using methyl 2-phenylindolizine-1-carboxylate **1c** (50.2 mg, 0.20 mmol, 1.00 eq.) and 3-methyl-2-((2-oxopropyl)thio)-4,5-dihydrothiazol-3-ium triflate **T4** (101.8 mg, 0.30 mmol, 1.50 eq.). The crude was purified by column chromatography on silica (PE:EtOAc, 4:1) to get the pure product as a yellow oil: 19.1 mg, 0.06 mmol, 31%. **^{1}H NMR** (400 MHz, $CDCl_3$) δ 8.30 (dt, J = 9.1, 1.2 Hz, 1H), 7.83 (dt, J = 7.0, 1.1 Hz, 1H), 7.52 – 7.29 (m, 5H), 7.13 (ddd, J = 9.1, 6.7, 1.1 Hz, 1H), 6.81 (td, J = 6.8, 1.3 Hz, 1H), 3.84 (s, 2H), 3.71 (s, 3H), 2.04 (s, 3H). **^{13}C{^{1}H} NMR** (100 MHz, $CDCl_3$) δ 204.8, 165.3, 136.3, 135.0, 131.8, 130.3, 128.0, 127.4, 123.2, 122.7, 120.4, 117.1, 113.2, 102.2, 50.7, 40.1, 29.1. **IR** (neat, cm^{-1}): 3049, 2945, 2853, 1685, 1502, 1448, 1388, 1357, 1334, 1233, 1127, 1067, 2018, 764, 732, 702, 539, 452. **ESI-HRMS**: m/z calcd. for $C_{19}H_{17}NO_3Na$ $[M+Na]^+$ 330.1106, found 330.1101.

Ethyl 3-(2-oxopropyl)-2-phenylindolizine-1-carboxylate (2d-4)

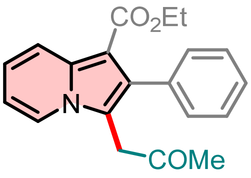

2d-4

Product **2d-4** was obtained following the general procedure **A** using ethyl 2-phenylindolizine-1-carboxylate **1d** (53.1 mg, 0.20 mmol, 1.00 eq.) and 3-methyl-2-((2-oxopropyl)thio)-4,5-dihydrothiazol-3-ium triflate **T4** (101.8 mg, 0.30 mmol, 1.50 eq.). The crude was purified by column chromatography on silica (PE:EtOAc, 4:1) to get the pure product as a colorless oil: 13.5 mg, 0.04 mmol, 21%. **^{1}H NMR** (400 MHz, $CDCl_3$) δ 8.32 (dt, J = 9.1, 1.3 Hz, 1H), 7.83 (dt, J = 6.9, 1.2 Hz, 1H), 7.47 – 7.29 (m, 5H), 7.13 (ddd, J = 9.1, 6.7, 1.1 Hz, 1H), 6.81 (td, J = 6.9, 1.4 Hz, 1H), 4.17 (q, J = 7.1 Hz, 2H), 3.84 (s, 2H), 2.03 (s, 3H), 1.12 (t, J = 7.1 Hz, 3H). **^{13}C{^{1}H} NMR** (100 MHz, $CDCl_3$) δ 204.8, 164.9, 136.3, 135.2, 131.8, 130.4, 127.9, 127.3, 123.1, 122.6, 120.4, 117.0, 113.2, 102.6, 59.3, 40.1, 29.0, 14.2. **IR** (neat, cm^{-1}): 2978, 1677, 1505, 1384, 1356, 1325, 1243, 1176, 1067, 1040, 736, 701, 541. **ESI-HRMS**: m/z calcd. for $C_{20}H_{19}NO_3Na$ $[M+Na]^+$ 344.1263, found 344.1257.

3-(3-Methyl-2-oxobutyl)-2-phenylindolizine-1-carbonitrile (2a-5)

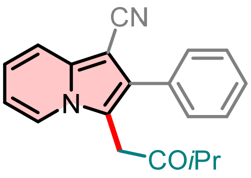

2a-5

Product **2a-5** was obtained following the general procedure **A** using 2-(phenyl)indolizine-1-carbonitrile **1a** (43.6 mg, 0.20 mmol, 1.00 eq.) and 3-methyl-2-((3-methyl-2-oxobutyl)thio)-4,5-dihydrothiazol-3-ium triflate **T5** (110.2 mg, 0.30 mmol, 1.50 eq.). The crude was purified by column chromatography on silica (PE:EtOAc, 4:1) to get the pure product as a white wax: 12.4 mg, 0.04 mmol, 21%. **^{1}H NMR** (400 MHz, $CDCl_3$) δ 7.77 (dt, J = 7.0, 1.1 Hz, 1H), 7.69 (dt, J = 8.9, 1.2 Hz, 1H), 7.49 – 7.47 (m, 2H), 7.45 – 7.42 (m, 3H), 7.12 (ddd, J = 9.0, 6.7, 1.0 Hz, 1H), 6.82 (td, J = 6.9, 1.3 Hz, 1H), 4.08 (s, 2H), 2.68 (p, J = 6.9 Hz, 1H), 1.09 (d, J = 6.9 Hz, 6H). **^{13}C{^{1}H} NMR** (100 MHz, $CDCl_3$) δ z209.9, 138.3, 132.4, 131.7, 129.6, 129.1, 129.0, 128.9, 128.3, 127.6, 123.8, 122.5, 118.0, 116.8, 116.2, 113.4, 82.1, 40.3, 36.9, 18.4. **IR** (neat, cm^{-1}): 3107, 2963, 2929, 2871, 2205, 1713, 1505, 1383, 1352, 1302, 1043, 801, 769, 750, 708, 497. **ESI-HRMS**: m/z calcd. for $C_{20}H_{18}N_2ONa$ $[M+Na]^+$ 325.1311, found 325.1311.

3-Methyl-1-(2-phenylindolizin-3-yl)butan-2-one (2b-5)

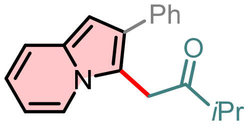

2b-5

Product **2b-5** was obtained following the general procedure **C** using 2-phenylindolizine **1b** (38.6 mg, 0.20 mmol, 1.00 eq.) and 3-methyl-2-((3-methyl-2-oxobutyl)thio)-4,5-dihydrothiazol-3-ium triflate **T5** (80.8 mg, 0.22 mmol, 1.10 eq.). The crude was purified by column chromatography on silica (PE:EtOAc, 98:2) to get the pure product as a yellow oil: 29.2 mg, 0.11 mmol, 53%. **^{1}H NMR** (400 MHz, $CDCl_3$) δ 7.72 – 7.63 (m, 1H), 7.53 – 7.31 (m, 6H), 6.72 (ddd, J = 9.0, 6.5, 1.0 Hz, 1H), 6.60 (d, J = 0.9 Hz, 1H), 6.56 (td, J = 6.7, 1.3 Hz, 1H), 4.13 (s, 2H), 2.62 (p, J = 6.9 Hz, 1H), 1.02 (d, J = 6.9 Hz, 6H). **^{13}C{^{1}H} NMR** (100 MHz, $CDCl_3$) δ 211.2, 136.5, 132.9, 129.8, 129.2, 128.7, 126.8, 122.3, 119.2, 117.1, 113.7, 110.9, 99.4, 39.6, 37.9, 18.4. **IR** (neat, cm^{-1}): 3051, 2965, 2928, 2870, 1705, 1603, 1452, 1363, 1299, 1031, 759, 726, 698, 486. **ESI-HRMS**: m/z calcd. for $C_{19}H_{19}NONa$ $[M+Na]^+$ 300.1364, found 300.1359.

3-(2-Oxo-2-phenylethyl)-2-phenylindolizine-1-carbonitrile (2a-6)

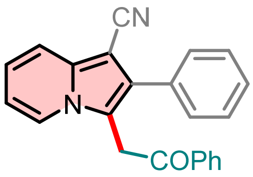

2a-6

Product **2a-6** was obtained following the general procedure **A** using 2-(phenyl)indolizine-1-carbonitrile **1a** (43.6 mg, 0.20 mmol, 1.00 eq.) and 3-methyl-2-((2-oxo-2-phenylethyl)thio)-4,5-dihydrothiazol-3-ium triflate **T6** (120.3 mg, 0.30 mmol, 1.50 eq.). The crude was purified by column chromatography on silica (PE:EtOAc, 4:1) to get the pure product as a yellow oil: 43.3 mg, 0.13 mmol, 64%. **^{1}H NMR** (400 MHz, $CDCl_3$) δ 7.90 (ddt, J = 16.6, 7.0, 1.2 Hz, 3H), 7.69 (dt, J = 8.9, 1.2 Hz, 1H), 7.64 – 7.54 (m, 1H), 7.54 – 7.35 (m, 7H), 7.12 (ddd, J = 8.9, 6.7, 1.0 Hz, 1H), 6.80 (td, J = 6.9, 1.3 Hz, 1H), 4.62 (s, 2H). **^{13}C{^{1}H} NMR** (100 MHz, $CDCl_3$) δ 194.8, 138.4, 135.6, 134.1, 132.5, 131.8, 129.6, 129.1, 129.0, 128.6, 128.3, 124.1, 122.6, 117.9, 116.9, 116.1, 113.4, 82.0, 35.1. **IR** (neat, cm^{-1}): 3055, 2955, 2922, 2853, 2206, 1676, 1510, 1447, 1392, 1329, 1263, 1209, 1074, 979, 742, 706, 687, 645, 601, 578, 493, 455. **ESI-HRMS**: m/z calcd. for $C_{23}H_{16}N_2ONa$ $[M+Na]^+$ 359.1161, found 359.1160.

3-(2-Oxo-2-phenylethyl)-2-phenylindolizine (2b-6)

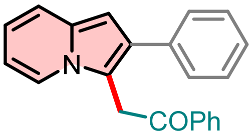

2b-6

Product **2b-6** was obtained following the general procedure **C** using 2-phenylindolizine **1b** (38.6 mg, 0.20 mmol, 1.00 eq.) and 3-methyl-2-((2-oxo-2-phenylethyl)thio)-4,5-dihydrothiazol-3-ium triflate **T6** (88.3 mg, 0.22 mmol, 1.10 eq.). The crude was purified by column chromatography on silica (PE:EtOAc, 95:5) to get the pure product as a yellow oil: 47.7 mg, 0.15 mmol, 77%. **^{1}H NMR** (400 MHz, $CDCl_3$) 1H NMR (400 MHz, CDC13) δ 7.89 (d, J = 1.2 Hz, 1H), 7.74 (dd, J = 7.1, 1.1 Hz, 1H), 7.54 – 7.29 (m, 10H), 6.68 (ddd, J = 9.0, 6.5, 1.1 Hz, 1H), 6.58 (d, J = 0.9 Hz, 1H), 6.50 (td, J = 6.8, 1.4 Hz, 1H), 4.63 (s, 2H). **^{13}C{^{1}H} NMR** (100 MHz, $CDCl_3$) δ 195.6, 136.6, 136.0, 133.5, 133.2, 133.0, 130.1, 129.8, 129.2, 128.7, 128.7, 128.6, 128.4, 128.4, 126.7, 122.6, 119.1, 117.0, 113.4, 110.7, 99.3, 35.7. **IR** (neat, cm^{-1}): 3056, 2925, 2854, 1674, 1595, 1447, 1362, 1328, 1301, 1213, 1162, 984, 907, 723, 686, 589, 556. **ESI-HRMS**: m/z calcd. for $C_{22}H_{17}NONa$ $[M+Na]^+$ 334.1208, found 334.1202.

Methyl 3-(2-oxo-2-phenylethyl)-2-phenylindolizine-1-carboxylate (2c-6)

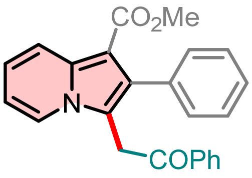

2c-6

Product **2c-6** was obtained following the general procedure **A** using methyl 2-phenylindolizine-1-carboxylate **1c** (50.2 mg, 0.20 mmol, 1.00 eq.) and 3-methyl-2-((2-oxo-2-phenylethyl)thio)-4,5-dihydrothiazol-3-ium triflate **T6** (120.3 mg, 0.30 mmol, 1.50 eq.). The crude was purified by column chromatography on silica (PE:EtOAc, 7:1) to get the pure product as a yellow wax: 32.4 mg, 0.09 mmol, 44%. **^{1}H NMR** (400 MHz, $CDCl_3$) δ 8.31 (dd, J = 9.1, 1.3 Hz, 1H), 7.92 (dd, J = 6.9, 1.4 Hz, 1H), 7.86 – 7.76 (m, 2H), 7.59 – 7.52 (m, 1H), 7.45 – 7.33 (m, 7H), 7.16 – 7.09 (m, 1H), 6.78 (td, J = 6.8, 1.4 Hz, 1H), 4.45 (s, 2H), 3.72 (s, 3H). **^{13}C{^{1}H} NMR** (100 MHz, $CDCl_3$) δ 195.5, 165.4, 136.5, 135.8, 135.0, 133.8, 131.8, 130.6, 128.8, 128.7, 127.9, 127.4, 123.7, 122.5, 120.3, 117.2, 112.9, 102.0, 50.6, 35.1. **IR** (neat, cm^{-1}): 2922, 2853, 1686, 1661, 1505, 1446, 1391, 1350, 1327, 1243, 1201, 1148, 1064, 1030, 979, 839, 785, 739, 703, 688, 636, 599, 576. **ESI-HRMS**: m/z calcd. for $C_{24}H_{19}NO_3Na$ $[M+Na]^+$ 392.1263, found 392.1257.

Ethyl 3-(2-oxo-2-phenylethyl)-2-phenylindolizine-1-carboxylate (2d-6)

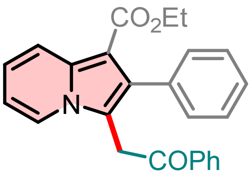

2d-6

Product **2d-6** was obtained following the general procedure **A** using ethyl 2-phenylindolizine-1-carboxylate **1d** (53.1 mg, 0.20 mmol, 1.00 eq.) and 3-methyl-2-((2-oxo-2-phenylethyl)thio)-4,5-dihydrothiazol-3-ium triflate **T6** (120.3 mg, 0.30 mmol, 1.50 eq.). The crude was purified by column chromatography on silica (PE:EtOAc, 8:1) to get the pure product as a yellow oil: 34.1 mg, 0.09 mmol, 45%. **^{1}H NMR** (400 MHz, $CDCl_3$) δ 8.33 (dt, J = 9.1, 1.2 Hz, 1H), 7.92 (dt, J = 7.0, 1.1 Hz, 1H), 7.89 – 7.68 (m, 2H), 7.59 – 7.51 (m, 1H), 7.42 – 7.34 (m, 6H), 7.11 (ddd, J = 9.1, 6.7, 1.1 Hz, 1H), 6.78 (td, J = 6.8, 1.4 Hz, 1H), 4.45 (s, 2H), 4.19 (q, J = 7.1 Hz, 2H), 1.14 (t, J = 7.1 Hz, 3H). **^{13}C{^{1}H} NMR** (100 MHz, $CDCl_3$) δ 195.5, 165.0, 136.5, 135.8, 135.2, 133.7, 131.8, 130.6, 128.8, 128.6, 127.8, 127.3, 123.6, 122.4, 120.3, 117.0, 112.9, 102.3, 59.2, 35.0, 14.3. **IR** (neat, cm^{-1}): 3053, 2975, 2924, 1683, 1656, 1507, 1415, 1358, 1317, 1252, 1203, 1180, 1151, 1067, 1043, 979, 912, 784, 745, 686, 634, 599, 552. **ESI-HRMS**: m/z calcd. for $C_{25}H_{21}NO_3Na$ $[M+Na]^+$ 406.1409, found 406.1413.

3-(2-(4-Methoxyphenyl)-2-oxoethyl)-2-phenylindolizine-1-carbonitrile (2a-7)

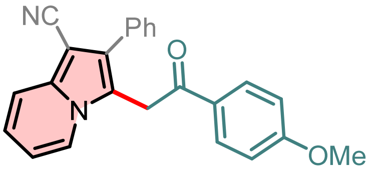

2a-7

Product **2a-7** was obtained following the general procedure **B** using 2-(phenyl)indolizine-1-carbonitrile **1a** (43.6 mg, 0.20 mmol, 1.00 eq.) and 2-((2-(4-methoxyphenyl)-2-oxoethyl)thio)-3-methyl-4,5-dihydrothiazol-3-ium triflate **T7** (129.4 mg, 0.30 mmol, 1.50 eq.). The crude was purified by column chromatography on silica (PE:EtOAc, 3:1) to get the pure product as a yellow wax: 33.6 mg, 0.09 mmol, 46%. **^{1}H NMR** (400 MHz, $CDCl_3$) δ 7.98 – 7.82 (m, 3H), 7.69 (dd, J = 8.9, 1.2 Hz, 1H), 7.53 – 7.38 (m, 5H), 7.12 (ddd, J = 9.0, 6.7, 1.0 Hz, 1H), 6.94 – 6.85 (m, 2H), 6.84 – 6.77 (m, 1H), 4.56 (s, 2H), 3.88 (s, 3H). **^{13}C{^{1}H} NMR** (100 MHz, $CDCl_3$) δ 193.3, 164.3, 138.4, 135.3, 132.6, 131.6, 131.0, 129.6, 129.1, 129.1, 128.6, 128.3, 125.1, 124.3, 122.5, 117.9, 117.0, 116.5, 114.1, 114.0, 113.3, 82.0, 55.7, 29.8. **IR** (neat, cm^{-1}): 2926, 2853, 2208, 1670, 1597, 1570, 1508, 1420, 1309, 1260, 1212, 1172, 1025, 974, 838, 770, 747, 707, 638, 594, 577, 493. **ESI-HRMS**: m/z calcd. for $C_{24}H_{18}N_2O_2Na$ $[M+Na]^+$ 389.1266, found 389.1260.

1-(4-Methoxyphenyl)-2-(2-phenylindolizin-3-yl)ethan-1-one (2b-7)

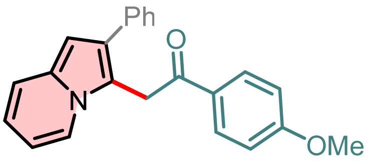

2b-7

Product **2b-7** was obtained following the general procedure **C** using 2-phenylindolizine **1b** (38.6 mg, 0.20 mmol, 1.00 eq.) and 2-((2-(4-methoxyphenyl)-2-oxoethyl)thio)-3-methyl-4,5-dihydrothiazol-3-ium triflate **T7** (94.9 mg, 0.22 mmol, 1.10 eq.). The crude was purified by column chromatography on silica (PE:EtOAc, 8:1) to get the pure product as a yellow oil: 42.5 mg, 0.12 mmol, 62%. **^{1}H NMR** (400 MHz, $CDCl_3$) δ 7.89 (d, J = 8.9 Hz, 2H), 7.85 – 7.75 (m, 1H), 7.48 – 7.32 (m, 6H), 6.89 – 6.82 (m, 2H), 6.71 (ddd, J = 9.0, 6.5, 1.1 Hz, 1H), 6.61 (d, J = 0.8 Hz, 1H), 6.53 (td, J = 6.8, 1.4 Hz, 1H), 4.61 (s, 2H), 3.85 (s, 3H). **^{13}C{^{1}H} NMR** (100 MHz, $CDCl_3$) δ 194.2, 163.8, 136.6, 132.9, 130.9, 130.6, 129.6, 129.2, 129.0, 128.7, 126.7, 122.7, 119.0, 117.0, 113.9, 113.8, 113.7, 110.6, 99.2, 55.5, 35.4. **IR** (neat, cm^{-1}): 3053, 2930, 2838, 1672, 1596, 1509, 1452, 1361, 1300, 1255, 1220, 1166, 1025, 982, 833, 757, 726, 699, 557, 429. **ESI-HRMS**: m/z calcd. for $C_{23}H_{19}NO_2Na$ $[M+Na]^+$ 364.1313, found 364.1308.

3-(2-Oxo-2-(4-(trifluoromethyl)phenyl)ethyl)-2-phenylindolizine-1-carbonitrile (2a-8)

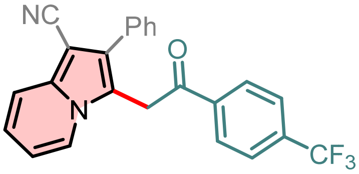

2a-8

Product **2a-8** was obtained following the general procedure **A** using 2-(phenyl)indolizine-1-carbonitrile **1a** (43.6 mg, 0.20 mmol, 1.00 eq.) and 3-methyl-2-((2-oxo-2-(4-(trifluoromethyl)phenyl)ethyl)thio)-4,5-dihydrothiazol-3-ium triflate **T8** (140.2 mg, 0.30 mmol, 1.50 eq.). The crude was purified by column chromatography on silica (PE:EtOAc, 4:1) to get the pure product as a yellow wax: 70.1 mg, 0.17 mmol, 87%. **^{1}H NMR** (400 MHz, $CDCl_3$) δ 7.97 (d, J = 8.1 Hz, 2H), 7.90 (dt, J = 7.0, 1.1 Hz, 1H), 7.71 – 7.63 (m, 3H), 7.51 – 7.40 (m, 5H), 7.12 (ddd, J = 9.0, 6.7, 1.0 Hz, 1H), 6.81 (td, J = 6.9, 1.3 Hz, 1H), 4.63 (s, 2H). **^{13}C{^{1}H} NMR** (100 MHz, $CDCl_3$) δ 194.0, 138.4, 138.1, 135.2 (q, J = 34 Hz), 132.3, 131.9, 129.5, 129.2, 129.1, 129.0, 128.9, 128.4, 128.2, 127.4, 126.2, 126.0, 126.0, 125.9, 125.9, 124.8, 124.0, 122.8, 122.1, 117.9, 117.7, 116.7, 115.3, 113.5, 113.4, 82.1, 35.4. **^{19}F{^{1}H} NMR** (377 MHz, $CDCl_3$) δ -63.68 (s, 3F). **IR** (neat, cm^{-1}): 3111, 3052, 2926, 2209, 1686, 1511, 1410, 1313, 1211, 1110, 1065, 1017, 983, 840, 801, 744, 700, 597, 491. **ESI-HRMS**: m/z calcd. for $C_{24}H_{15}F_3N_2ONa$ $[M+Na]^+$ 427.1034, found 427.1029.

2-(2-Phenylindolizin-3-yl)-1-(4-(trifluoromethyl)phenyl)ethan-1-one (2b-8)

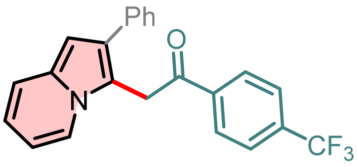

2b-8

Product **2b-8** was obtained following the general procedure **C** using 2-phenylindolizine **1b** (38.6 mg, 0.20 mmol, 1.00 eq.) and 3-methyl-2-((2-oxo-2-(4-(trifluoromethyl)phenyl)ethyl)thio)-4,5-dihydrothiazol-3-ium triflate **T8** (103.3 mg, 0.22 mmol, 1.10 eq.). The crude was purified by column chromatography on silica (PE:EtOAc, 98:2) to get the pure product as a yellow wax: 56.0 mg, 0.15 mmol, 74%. **^{1}H NMR** (400 MHz, $CDCl_3$) δ 7.91 (d, J = 8.2 Hz, 2H), 7.82 (dd, J = 7.2, 1.1 Hz, 1H), 7.60 (d, J = 8.4 Hz, 2H), 7.51 – 7.37 (m, 6H), 6.74 (ddd, J = 8.9, 6.6, 1.1 Hz, 1H), 6.61 (d, J = 0.8 Hz, 1H), 6.57 (td, J = 6.8, 1.3 Hz, 1H), 4.67 (s, 2H). **^{13}C{^{1}H} NMR** (100 MHz, $CDCl_3$) 13C NMR (101 MHz, CDCl3) δ 194.7, 138.5, 136.5, 134.6 (q, J = 32 Hz), 133.2, 130.0, 129.2, 129.0, 129.0, 127.1, 125.7 (q, J = 4 Hz), 122.6, 119.2, 117.4, 112.5, 111.0, 99.6, 36.1. **^{19}F{^{1}H} NMR** (377 MHz, $CDCl_3$) δ -63.64 (s, 3F). **IR** (neat, cm^{-1}): 3077, 2928, 2876, 1670, 1510, 1449, 1409, 1362, 1311, 1202, 1163, 1119, 1064, 1015, 982, 842, 764, 735, 704, 606, 556, 499. **ESI-HRMS**: m/z calcd. for $C_{23}H_{16}F_3NONa$ $[M+Na]^+$ 402.1082, found 402.1076.

Ethyl 3-(2-oxo-2-(4-(trifluoromethyl)phenyl)ethyl)-2-phenylindolizine-1-carboxylate (2c-8)

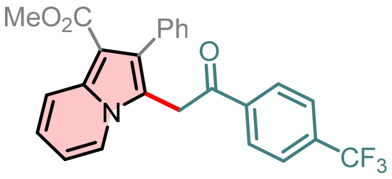

2c-8

Product **2c-8** was obtained following the general procedure **A** using methyl 2-phenylindolizine-1-carboxylate **1c** (50.2 mg, 0.20 mmol, 1.00 eq.) and 3-methyl-2-((2-oxo-2-(4-(trifluoromethyl)phenyl)ethyl)thio)-4,5-dihydrothiazol-3-ium triflate **T8** (140.2 mg, 0.30 mmol, 1.50 eq.). The crude was purified by column chromatography (PE:EtOAc, 7:1) to get the pure product as a yellow oil: 55.7 mg, 0.13 mmol, 64%. **^{1}H NMR** (400 MHz, $CDCl_3$) δ 8.31 (dt, J = 9.1, 1.2 Hz, 1H), 7.96 (dt, J = 7.1, 1.1 Hz, 1H), 7.83 (d, J = 8.1 Hz, 2H), 7.62 (d, J = 8.3 Hz, 2H), 7.48 – 7.36 (m, 3H), 7.36 – 7.28 (m, 2H), 7.13 (ddd, J = 9.1, 6.7, 1.1 Hz, 1H), 6.80 (td, J = 6.9, 1.3 Hz, 1H), 4.47 (s, 2H), 3.72 (s, 3H). **^{13}C{^{1}H} NMR** (100 MHz, $CDCl_3$) δ 194.6, 171.3, 165.3, 138.3, 136.6, 134.9 (q, J = 32 Hz), 134.9, , 131.9, 130.5, 129.2, 129.0, 128.0, 127.6, 127.5, 125.8 (q, J = 3 Hz), 124.9, 123.5, 122.7, 122.2, 120.4, 116.3, 113.1, 102.1, 50.7, 35.3. **^{19}F{^{1}H} NMR** (377 MHz, $CDCl_3$) δ -63.69 (s, 3F). **IR** (neat, cm^{-1}): 2953,

2923, 2853, 1690, 1504, 1438, 1408, 1391, 1320, 1243, 1170, 1125, 1062, 1029, 985, 837, 805, 770, 750, 704, 640, 606, 496. **ESI-HRMS**: m/z calcd. for $C_{25}H_{18}F_3NO_3Na$ $[M+Na]^+$ 460.1136, found 460.1131.

Ethyl 3-(2-oxo-2-(4-(trifluoromethyl)phenyl)ethyl)-2-phenylindolizine-1-carboxylate (2d-8)

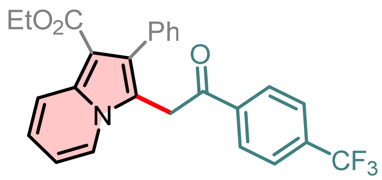

2d-8

Product **2d-8** was obtained following the general procedure **A** using ethyl 2-phenylindolizine-1-carboxylate **1d** (53.1 mg, 0.20 mmol, 1.00 eq.) and 3-methyl-2-((2-oxo-2-(4-(trifluoromethyl)phenyl)ethyl)thio)-4,5-dihydrothiazol-3-ium triflate **T8** (140.2 mg, 0.30 mmol, 1.50 eq.). The crude was purified by column chromatography on silica (PE:EtOAc, 9:1) to get the pure product as a yellow wax: 53.7 mg, 0.12 mmol, 59%. **^{1}H NMR** (400 MHz, $CDCl_3$) δ 8.31 (dt, J = 9.2, 1.2 Hz, 1H), 7.95 (dt, J = 7.0, 1.1 Hz, 1H), 7.89 – 7.75 (m, 2H), 7.60 (d, J = 8.2 Hz, 2H), 7.47 – 7.27 (m, 5H), 7.11 (ddd, J = 9.1, 6.7, 1.1 Hz, 1H), 6.78 (td, J = 6.8, 1.4 Hz, 1H), 4.45 (s, 2H), 4.16 (q, J = 7.1 Hz, 2H), 1.12 (t, J = 7.1 Hz, 3H). **^{13}C{^{1}H} NMR** (100 MHz, $CDCl_3$) δ 194.6, 164.9, 138.4, 136.6, 135.3, 135.1, 135.0, 134.7, 131.9, 130.6, 129.2, 129.0, 127.9, 127.6, 127.5, 125.8, 125.8, 125.7, 125.7, 124.9, 123.5, 122.6, 122.2, 120.4, 116.1, 113.1, 102.5, 59.3, 35.3, 14.2. **^{19}F{^{1}H} NMR** (377 MHz, $CDCl_3$) δ -63.68 (s, 3F). **IR** (neat, cm^{-1}): 3078, 2954, 2909, 1682, 1504, 1410, 1322, 1240, 1175, 1125, 1063, 1039, 842, 773, 750, 705, 640, 606, 497. **ESI-HRMS**: m/z calcd. for $C_{26}H_{20}F_3NO_3Na$ $[M+Na]^+$ 474.1293, found 474.1287.

2-(1-Cyano-2-phenylindolizin-3-yl)-*N*,*N*-dimethylacetamide (2a-9)

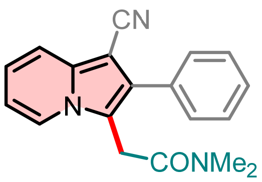

2a-9

Product **2a-9** was obtained following the general procedure **A** using 2-(phenyl)indolizine-1-carbonitrile **1a** (43.6 mg, 0.20 mmol, 1.00 eq.) and 2-((2-(dimethylamino)-2-oxoethyl)thio)-3-methyl-4,5-dihydrothiazol-3-ium triflate **T9** (110.5 mg, 0.30 mmol, 1.50 eq.). The crude was purified by column chromatography on silica (PE:EtOAc, 2:1 to 1:2) to get the pure product as a white wax: 29.3 mg, 0.10

mmol, 48%. ^{1}**H NMR** (400 MHz, $CDCl_3$) δ 8.28 – 8.04 (m, 1H), 7.65 (dt, J = 9.1, 1.3 Hz, 1H), 7.56 – 7.33 (m, 5H), 7.10 (ddd, J = 9.0, 6.7, 1.1 Hz, 1H), 6.81 (td, J = 6.8, 1.3 Hz, 1H), 3.98 (s, 2H), 2.94 (s, 3H), 2.85 (s, 3H). ^{13}**C{^{1}H} NMR** (100 MHz, $CDCl_3$) δ 168.1, 138.3, 132.6, 131.0, 129.6, 129.0, 128.2, 127.6, 124.9, 122.6, 117.6, 117.0, 116.7, 113.2, 81.6, 37.5, 35.9, 30.3. **IR** (neat, cm^{-1}): 3109, 2924, 2854, 2203, 1650, 1494, 1389, 1230, 1130, 843, 765, 741, 701, 604, 493, 455. **ESI-HRMS**: m/z calcd. for $C_{19}H_{17}N_3ONa$ $[M+Na]^+$ 326.1269, found 326.1264.

_N,N_-Dimethyl-2-(2-phenylindolizin-3-yl)acetamide (2b-9)

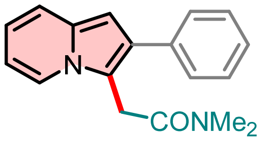

2b-9

Product **2b-9** was obtained following the general procedure **C** using 2-phenylindolizine **1b** (38.6 mg, 0.20 mmol, 1.00 eq.) and 2-((2-(dimethylamino)-2-oxoethyl)thio)-3-methyl-4,5-dihydrothiazol-3-ium triflate **T9** (85.0 mg, 0.22 mmol, 1.10 eq.). The crude was purified by column chromatography on silica (PE:EtOAc, 2:1) to get the pure product as a colorless oil: 15.7 mg, 0.06 mmol, 28%. ^{1}**H NMR** (400 MHz, $CDCl_3$) δ 8.10 (d, J = 7.1 Hz, 1H), 7.46 – 7.32 (m, 6H), 6.71 (dd, J = 9.0, 6.4 Hz, 1H), 6.57 (d, J = 8.2 Hz, 2H), 4.08 (s, 2H), 2.91 (s, 3H), 2.76 (s, 3H). ^{13}**C{^{1}H} NMR** (100 MHz, $CDCl_3$) δ 169.2, 136.8, 132.9, 129.3, 129.2, 128.7, 126.7, 123.5, 118.9, 117.1, 114.0, 110.7, 99.0, 37.5, 36.0, 31.3. **IR** (neat, cm^{-1}): 3437, 3051, 2923, 2854, 1639, 1497, 1393, 1362, 1302, 1231, 1106, 1027, 908, 753, 700, 596, 563, 457. **ESI-HRMS**: m/z calcd. for $C_{18}H_{18}N_2ONa$ $[M+Na]^+$ 301.1317, found 301.1311.

3-(Cyanomethyl)-2-phenylindolizine-1-carbonitrile (2a-10)

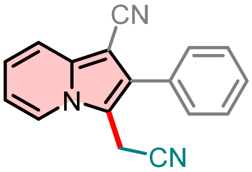

2a-10

Product **2a-10** was obtained following the general procedure **B** using 2-(phenyl)indolizine-1-carbonitrile **1a** (43.6 mg, 0.20 mmol, 1.00 eq.) and 2-((cyanomethyl)thio)-3-methyl-4,5-dihydrothiazol-3-ium triflate **T10** (96.7 mg, 0.30 mmol, 1.50 eq.). The crude was purified by column chromatography on silica (PE:EtOAc, 4:1 to 3:1) to get a brown wax: 17.4 mg, 0.68 mmol, 34%. ^{1}**H NMR** (400 MHz, $CDCl_3$) δ 8.04 (d, J = 7.0 Hz, 1H), 7.79 – 7.76 (m, 1H), 7.58 – 7.46 (m, 5H), 7.28 – 7.24 (m, 1H), 7.02 (td, J = 6.9, 1.3 Hz, 1H), 4.03 (s, 2H). ^{13}**C{^{1}H} NMR** (100 MHz, $CDCl_3$) δ 138.5, 132.5, 131.1, 129.6, 129.4, 129.3,

128.9, 127.7, 123.5, 123.1, 118.4, 116.0, 114.9, 114.5, 109.9, 82.8, 14.3. **IR** (neat, cm^{-1}): 2957, 2922, 2853, 2203, 1509, 1390, 1073, 792, 735, 707, 435. **ESI-HRMS**: m/z calcd. for $C_{17}H_{11}N_3Na$ $[M+Na]^+$ 280.0851, found 280.0845.

2-(2-Phenylindolizin-3-yl)acetonitrile (2b-10)

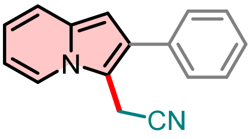

2b-10

Product **2b-10** was obtained following the general procedure **C** using 2-phenylindolizine **1b** (38.6 mg, 0.20 mmol, 1.00 eq.) and 2-((cyanomethyl)thio)-3-methyl-4,5-dihydrothiazol-3-ium triflate **T10** (70.9 mg, 0.22 mmol, 1.10 eq.). The crude was purified by column chromatography on silica (PE:EtOAc, 8:1) to get the pure product as a yellow wax: 31.1 mg, 0.13 mmol, 67%. **^{1}H NMR** (400 MHz, $CDCl_3$) δ 7.87 (dq, J = 7.0, 1.2 Hz, 1H), 7.62 – 7.31 (m, 6H), 6.84 (ddd, J = 9.0, 6.5, 1.0 Hz, 1H), 6.73 (td, J = 6.8, 1.3 Hz, 1H), 6.61 (d, J = 0.8 Hz, 1H), 4.06 (s, 2H). **^{13}C{^{1}H} NMR** (100 MHz, $CDCl_3$) δ 135.2, 133.5, 130.2, 129.2, 129.0, 127.3, 121.7, 119.4, 118.0, 115.9, 111.7, 107.2, 99.6, 14.5. **IR** (neat, cm^{-1}): 3072, 3031, 2918, 2851, 2246, 1450, 1406, 1366, 1302, 905, 749, 722, 698, 559, 491, 427. **ESI-HRMS**: m/z calcd. for $C_{16}H_{12}N_2Na$ $[M+Na]^+$ 255.0898, found 255.0893.

Methyl 3-(cyanomethyl)-2-phenylindolizine-1-carboxylate (2c-10)

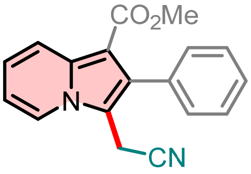

2c-10

Product **2c-10** was obtained following the general procedure **A** using methyl 2-phenylindolizine-1-carboxylate **1c** (50.2 mg, 0.20 mmol, 1.00 eq.) and 2-((cyanomethyl)thio)-3-methyl-4,5-dihydrothiazol-3-ium triflate **T10** (96.7 mg, 0.30 mmol, 1.50 eq.). The crude was purified by column chromatography on silica (PE:EtOAc, 5:1 to 4:1) to get the pure product as a brown wax: 33.7 mg, 0.12 mmol, 58%. **^{1}H NMR** (400 MHz, $CDCl_3$) δ 8.36 (dq, J = 9.1, 1.1 Hz, 1H), 7.99 (dq, J = 6.9, 1.0 Hz, 1H), 7.49 – 7.33 (m, 5H), 7.26 – 7.19 (m, 1H), 7.00 – 6.94 (m, 1H), 3.85 (s, 2H), 3.72 (d, J = 0.7 Hz, 3H). **^{13}C{^{1}H} NMR** (100 MHz, $CDCl_3$) δ 165.0, 136.7, 133.7, 132.4, 130.4, 129.9, 128.1, 127.9, 127.9, 123.2, 122.5, 120.8, 115.3, 113.9, 111.2, 102.5, 50.8, 14.0. **IR** (neat, cm^{-1}): 3077, 2922, 2853, 2247, 1682, 1502, 1439, 1331,

1232, 1189, 1133, 1062, 1017, 768, 748, 705, 502. **ESI-HRMS**: m/z calcd. for $C_{18}H_{14}N_2O_2Na$ $[M+Na]^+$ 313.0953, found 313.0947.

Ethyl 3-(cyanomethyl)-2-phenylindolizine-1-carboxylate (2d-10)

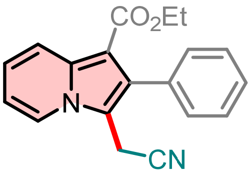

2d-10

Product **2d-10** was obtained following the general procedure **A** using ethyl 2-phenylindolizine-1-carboxylate **1d** (53.1 mg, 0.20 mmol, 1.00 eq.) and 2-((cyanomethyl)thio)-3-methyl-4,5-dihydrothiazol-3-ium triflate **T10** (96.7 mg, 0.30 mmol, 1.50 eq.). The crude was purified by column chromatography on silica (PE:EtOAc, 5:1) to get the pure product as a white wax: 26.6 mg, 0.09 mmol, 44%. **^{1}H NMR** (400 MHz, $CDCl_3$) δ 8.38 (d, J = 9.1 Hz, 1H), 7.99 (d, J = 7.0 Hz, 1H), 7.48 – 7.32 (m, 5H), 7.22 (ddd, J = 9.1, 6.8, 1.1 Hz, 1H), 6.96 (td, J = 6.8, 1.3 Hz, 1H), 4.18 (q, J = 7.1 Hz, 2H), 3.85 (s, 2H), 1.13 (t, J = 7.1 Hz, 3H). **^{13}C{^{1}H} NMR** (100 MHz, $CDCl_3$) δ 164.6, 136.7, 133.9, 132.3, 130.4, 130.1, 128.0, 127.8, 127.8, 123.1, 122.5, 120.7, 115.3, 113.9, 111.0, 102.9, 59.5, 14.2, 14.0. **IR** (neat, cm^{-1}): 2978, 2924, 2853, 1672, 1506, 1403, 1319, 1244, 1182, 1150, 1064, 773, 739, 699, 496. **ESI-HRMS**: m/z calcd. for $C_{19}H_{16}N_2O_2Na$ $[M+Na]^+$ 327.1109, found 327.1104.

2-Phenyl-3-(2,2,2-trifluoroethyl)indolizine-1-carbonitrile (2a-11) + 2-phenyl-5-(2,2,2-trifluoroethyl)indolizine-1-carbonitrile (2a-11')

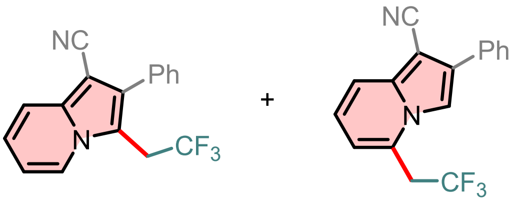

2a-11 + 2a-11' (4.5:1)

Products **2a-11 + 2a-11'** were obtained following the general procedure **A** using 2-(phenyl)indolizine-1-carbonitrile **1a** (43.6 mg, 0.20 mmol, 1.00 eq.) and 3-methyl-2-((2,2,2-trifluoroethyl)thio)-4,5-dihydrothiazol-3-ium triflate **T11** (109.6 mg, 0.30 mmol, 1.50 eq.). The crude was purified by column chromatography on silica (PE:EtOAc, 4:1) to get the inseparable product mixture as a colorless oil: 20.7 mg, 0.07 mmol, 34%. **^{1}H NMR** (400 MHz, $CDCl_3$) (major) δ 8.09 (dq, J = 7.2, 1.0 Hz, 1H), 7.73 (dt, J = 8.9, 1.2 Hz, 1H), 7.57 – 7.37 (m, 5H), 7.20 (ddd, J = 9.0, 6.7, 1.0 Hz, 1H), 6.91 (td, J = 6.9, 1.3 Hz,

1H), 3.77 (q, J = 9.8 Hz, 2H). **^{1}H NMR** (400 MHz, $CDCl_3$) (minor) δ 7.83 – 7.77 (m, 2H), 7.52 (s, 1H), 7.49 – 7.38 (m, 4H), 7.12 (dd, J = 9.0, 6.9 Hz, 1H), 6.84 (d, J = 6.5 Hz, 1H), 3.80 (q, J = 9.8 Hz, 2H). **^{13}C{^{1}H} NMR** (100 MHz, $CDCl_3$) 13C NMR (101 MHz, CDCl3) δ 138.6, 134.4, 131.6, 129.7, 129.2, 129.1, 128.7, 128.5, 127.6, 123.9, 123.2, 122.3, 118.2, 118.1, 116.3, 113.8, 83.4, 37.1 (q, J = 31 Hz), 30.2 (q, J = 32 Hz). **^{19}F{^{1}H} NMR** (377 MHz, $CDCl_3$) δ -63.59 (s, 3F, major), -63.90 (s, 3F, minor). **IR** (neat, cm^{-1}): 3138, 3057, 2953, 2211, 1514, 1363, 1249, 1133, 1106, 1026, 933, 908, 830, 744, 702, 648, 624, 494, 453. **ESI-HRMS**: m/z calcd. for $C_{17}H_{11}F_3N_2Na$ $[M+Na]^+$ 323.0772, found 323.0767.

2-Phenyl-3-(2,2,2-trifluoroethyl)indolizine (2b-11)

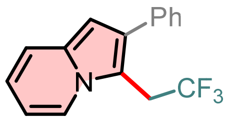

2b-11

Product **2b-11** was obtained following the general procedure **A** using 2-phenylindolizine **1b** (38.6 mg, 0.20 mmol, 1.00 eq.) and 3-methyl-2-((2,2,2-trifluoroethyl)thio)-4,5-dihydrothiazol-3-ium triflate **T11** (109.6 mg, 0.30 mmol, 1.50 eq.). The crude was purified by column chromatography on silica (PE:EtOAc, 99:1) to get the pure product as a colorless oil: 23.0 mg, 0.08 mmol, 42%. **^{1}H NMR** (400 MHz, $CDCl_3$) δ 7.95 (d, J = 7.2 Hz, 1H), 7.57 – 7.34 (m, 6H), 6.80 (ddd, J = 9.0, 6.5, 1.0 Hz, 1H), 6.69 – 6.56 (m, 2H), 3.80 (q, J = 10.1 Hz, 2H). **^{13}C{^{1}H} NMR** (100 MHz, $CDCl_3$) δ 135.9, 133.6, 132.0, 130.5, 129.4, 128.8, 128.7, 128.5, 128.1, 127.5, 127.1, 124.8, 122.6 (q, J = 3 Hz), 119.2, 117.8, 111.1, 100.2, 30.4 (q, J = 30 Hz). **^{19}F{^{1}H} NMR** (377 MHz, $CDCl_3$) δ -63.69 (s, 3F). **IR** (neat, cm^{-1}): 3056, 2925, 1352, 1305, 1250, 1128, 1023, 931, 758, 726, 700. **ESI-HRMS**: m/z calcd. for $C_{16}H_{12}F_3NONa$ $[M+Na+O]^+$ 314.0763, found 314.0763.

3-Isopropyl-2-phenylindolizine-1-carbonitrile (2a-12)

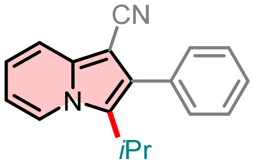

2a-12

Product **2a-12** was obtained following the general procedure **A** using 2-(phenyl)indolizine-1-carbonitrile **1a** (43.6 mg, 0.20 mmol, 1.00 eq.) and 2-(isopropylthio)-3-methyl-4,5-dihydrothiazol-3-ium triflate **T12** (97.6 mg, 0.30 mmol, 1.50 eq.). The crude was purified by column chromatography on silica (PE:EtOAc, 5:1) to get the pure product as a colorless wax: 6.2 mg, 0.02 mmol, 12%. **^{1}H NMR**

(400 MHz, $CDCl_3$) δ 7.83 – 7.80 (m, 2H), 7.60 (d, J = 8.8 Hz, 1H), 7.50 – 7.47 (m, 3H), 7.41 – 7.37 (m, 1H), 7.13 (dd, J = 8.9, 7.1 Hz, 1H), 6.69 (d, J = 7.0 Hz, 1H), 3.31 (p, J = 6.6 Hz, 1H), 1.45 (d, J = 6.8 Hz, 6H). **^{13}C{^{1}H} NMR** (100 MHz, $CDCl_3$) δ 144.3, 140.4, 132.8, 131.8, 129.2, 128.2, 127.6, 123.3, 117.5, 115.6, 108.6, 108.4, 80.6, 29.6, 20.3. **IR** (neat, cm^{-1}): 3132, 2922, 2206, 1515, 1483, 1414, 1225, 1166, 757, 692, 538, 482. **ESI-HRMS**: m/z calcd. for $C_{18}H_{16}N_2Na$ $[M+Na]^+$ 283.1206, found 283.1208.

Methyl 2-(2-(*p*-tolyl)indolizin-3-yl)acetate (2e)

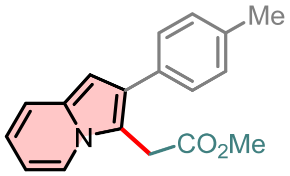

2e

Product **2e** was obtained following the general procedure **C** using 2-(*p*-tolyl)indolizine **1e** (41.4 mg, 0.20 mmol, 1.00 eq.) and 2-((2-methoxy-2-oxoethyl)thio)-3-methyl-4,5-dihydrothiazol-3-ium triflate **T1** (78.2 mg, 0.22 mmol, 1.10 eq.). The crude was purified by column chromatography on silica (PE:EtOAc, 95:5) to get the pure product as a colorless oil: 35.6 mg, 0.13 mmol, 64%. **^{1}H NMR** (400 MHz, $CDCl_3$) δ 7.92 (d, J = 7.1 Hz, 1H), 7.50 (d, J = 8.0 Hz, 2H), 7.43 (dt, J = 8.9, 1.2 Hz, 1H), 7.36 – 7.27 (m, 2H), 6.75 (ddd, J = 9.0, 6.5, 1.1 Hz, 1H), 6.70 – 6.48 (m, 2H), 4.02 (s, 2H), 3.77 (s, 3H), 2.44 (s, 3H). **^{13}C{^{1}H} NMR** (100 MHz, $CDCl_3$) δ 170.9, 136.5, 133.2, 132.8, 129.8, 129.4, 129.1, 122.5, 119.1, 117.1, 112.6, 110.7, 99.1, 52.4, 31.4, 21.3. **IR** (neat, cm^{-1}): 3025, 2952, 2917, 2856, 1729, 1431, 1360, 1299, 1248, 1156, 997, 812, 771, 718, 500. **ESI-HRMS**: m/z calcd. for $C_{18}H_{17}NO_2Na$ $[M+Na]^+$ 302.1157, found 302.1151.

Methyl 2-(2-(4-methoxyphenyl)indolizin-3-yl)acetate (2f)

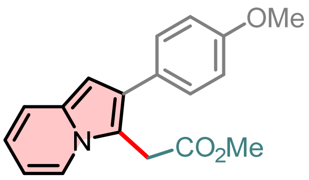

2f

Product **2f** was obtained following the general procedure **C** using 2-(4-methoxyphenyl)indolizine **1f** (44.6 mg, 0.20 mmol, 1.00 eq.) and 2-((2-methoxy-2-oxoethyl)thio)-3-methyl-4,5-dihydrothiazol-3-ium triflate **T1** (78.2 mg, 0.22 mmol, 1.10 eq.). The crude was purified by column chromatography on silica (PE:EtOAc, 9:1) to get the pure product as a colorless oil: 38.8 mg, 0.13 mmol, 66%. **^{1}H NMR** (400

MHz, $CDCl_3$) δ 7.91 (dt, J = 7.1, 0.9 Hz, 1H), 7.56 – 7.48 (m, 2H), 7.42 (dd, J = 8.9, 1.3 Hz, 1H), 7.06 – 6.98 (m, 2H), 6.79 – 6.70 (m, 1H), 6.63 – 6.54 (m, 2H), 3.99 (s, 2H), 3.88 (s, 3H), 3.77 (s, 3H). **$^{13}C\{^1H\}$ NMR** (100 MHz, $CDCl_3$) δ 170.9, 158.7, 132.8, 130.3, 129.6, 128.6, 122.4, 119.0, 117.1, 114.2, 112.5, 110.7, 99.0, 55.4, 52.5, 31.4. **IR** (neat, cm^{-1}): 2999, 2951, 2835, 1731, 1611, 1521, 1434, 1361, 1244, 1175, 1029, 836, 770, 728, 559. **ESI-HRMS**: m/z calcd. for $C_{18}H_{17}NO_3Na$ $[M+Na]^+$ 318.1106, found 318.1101.

Methyl 2-(2-(4-bromophenyl)indolizin-3-yl)acetate (2g)

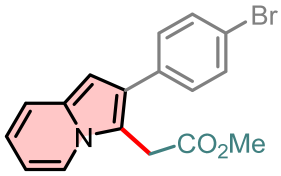

2g

Product **2g** was obtained following the general procedure **C** using 2-(4-bromophenyl)indolizine **1g** (54.4 mg, 0.20 mmol, 1.00 eq.) and 2-((2-methoxy-2-oxoethyl)thio)-3-methyl-4,5-dihydrothiazol-3-ium triflate **T1** (78.2 mg, 0.22 mmol, 1.10 eq.). The crude was purified by column chromatography on silica (PE:EtOAc, 95:5) to get the pure product as a colorless oil: 45.1 mg, 0.13 mmol, 66%. **^1H NMR** (400 MHz, $CDCl_3$) δ 7.92 (dd, J = 7.1, 1.1 Hz, 1H), 7.69 – 7.52 (m, 2H), 7.55 – 7.29 (m, 3H), 6.76 (ddd, J = 9.1, 6.6, 1.1 Hz, 1H), 6.70 – 6.36 (m, 2H), 3.96 (s, 2H), 3.77 (s, 3H). **$^{13}C\{^1H\}$ NMR** (100 MHz, $CDCl_3$) δ 170.6, 135.1, 132.9, 131.8, 130.8, 128.6, 122.5, 121.0, 119.1, 117.5, 112.7, 111.1, 98.9, 52.5, 31.3. **IR** (neat, cm^{-1}): 2947, 1728, 1430, 1361, 1327, 1306, 1156, 1072, 996, 826, 772, 728, 666, 545, 502. **ESI-HRMS**: m/z calcd. for $C_{17}H_{14}NBrO_2Na$ $[M+Na]^+$ 366.0106, found 366.0100.

Methyl 2-(2-(4-chlorophenyl)indolizin-3-yl)acetate (2h)

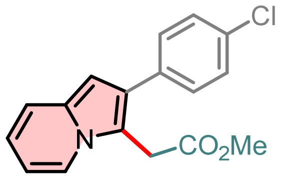

2h

Product **2h** was obtained following the general procedure **C** using 2-(4-chlorophenyl)indolizine **1h** (45.6 mg, 0.20 mmol, 1.00 eq.) and 2-((2-methoxy-2-oxoethyl)thio)-3-methyl-4,5-dihydrothiazol-3-ium triflate **T1** (78.2 mg, 0.22 mmol, 1.10 eq.). The crude was purified by column chromatography on silica (PE:EtOAc, 96:4) to get the pure product as a yellow oil: 46.0 mg, 0.15 mmol, 77%. **^1H NMR** (400 MHz, $CDCl_3$) δ 7.92 (dq, J = 7.0, 1.1 Hz, 1H), 7.56 – 7.49 (m, 2H), 7.47 – 7.38 (m, 3H), 6.76 (ddd, J =

9.0, 6.5, 1.0 Hz, 1H), 6.61 (td, J = 6.8, 1.4 Hz, 1H), 6.58 (d, J = 0.9 Hz, 1H), 3.97 (s, 2H), 3.77 (s, 3H). **^{13}C{^{1}H} NMR** (100 MHz, $CDCl_3$) δ 170.6, 134.6, 132.9, 132.8, 130.5, 128.8, 128.6, 122.5, 119.1, 117.5, 112.7, 111.1, 99.0, 52.5, 31.3. **IR** (neat, cm^{-1}): 2949, 1730, 1483, 1432, 1361, 1306, 1156, 1092, 1012, 834, 768, 725, 500, 468. **ESI-HRMS**: m/z calcd. for $C_{17}H_{14}NClO_2Na$ $[M+Na]^+$ 322.0611, found 322.0605.

Methyl 2-(2-(4-(trifluoromethyl)phenyl)indolizin-3-yl)acetate (2i)

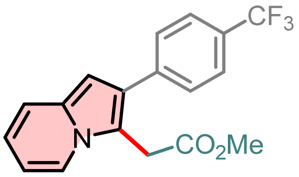

2i

Product **2i** was obtained following the general procedure **C** using 2-(4-(trifluoromethyl)phenyl)indolizine **1i** (52.2 mg, 0.20 mmol, 1.00 eq.) and 2-((2-methoxy-2-oxoethyl)thio)-3-methyl-4,5-dihydrothiazol-3-ium triflate **T1** (78.2 mg, 0.22 mmol, 1.10 eq.). The crude was purified by column chromatography on silica (PE:EtOAc, 95:5) to get the pure product as a white wax: 44.5 mg, 0.13 mmol, 67%. **^{1}H NMR** (400 MHz, $CDCl_3$) δ 7.94 (dd, J = 7.1, 1.2 Hz, 1H), 7.71 (d, J = 1.0 Hz, 4H), 7.44 (dt, J = 9.0, 1.3 Hz, 1H), 6.89 – 6.71 (m, 1H), 6.71 – 6.49 (m, 2H), 3.99 (s, 2H), 3.78 (s, 3H). **^{13}C{^{1}H} NMR** (100 MHz, $CDCl_3$) δ 170.5, 139.9, 133.1, 128.8 (q, J = 32 Hz), 128.6, 125.9, 125.6 (q, J = 4 Hz), 123.2, 122.6, 119.3, 117.7, 113.1, 111.3, 99.1, 52.6, 31.3. **^{19}F{^{1}H} NMR** (377 MHz, $CDCl_3$) δ -62.78 (s, 3F). **IR** (neat, cm^{-1}): 2923, 2853, 1736, 1615, 1434, 1319, 1156, 1102, 1065, 1015, 907, 849, 766, 726, 685, 612. **ESI-HRMS**: m/z calcd. for $C_{18}H_{14}NF_3O_2Na$ $[M+Na]^+$ 356.0854, found 356.0869.

Methyl 2-(2-(4-hydroxyphenyl)indolizin-3-yl)acetate (2j)

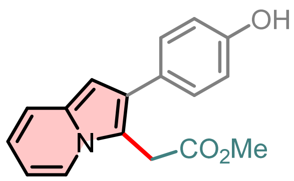

2j

Product **2j** was obtained following the general procedure **D** using 2-(4-hydroxyphenyl)indolizine **1j** (41.8 mg, 0.20 mmol, 1.00 eq.) and 2-((2-methoxy-2-oxoethyl)thio)-3-methyl-4,5-dihydrothiazol-3-ium triflate **T1** (78.2 mg, 0.22 mmol, 1.10 eq.). The crude was purified by column chromatography on silica (PE:EtOAc, 4:1) to get the pure product as a yellow oil: 26.2 mg, 0.09 mmol, 47%. **^{1}H NMR** (400 MHz, $CDCl_3$) δ 7.89 (dd, J = 7.1, 1.1 Hz, 1H), 7.48 – 7.36 (m, 3H), 6.96 – 6.89 (m, 2H), 6.79 – 6.69 (m, 1H), 6.64 – 6.50 (m, 2H), 5.17 (br s, 1H), 3.98 (s, 2H), 3.76 (s, 3H). **^{13}C{^{1}H} NMR** (100 MHz, $CDCl_3$) δ 171.0, 154.8, 132.8, 130.5, 129.6, 128.7, 122.5, 119.0, 117.2, 115.7, 112.5, 110.7, 99.0, 52.5, 31.4. **IR** (neat, cm^{-1}): 3363, 2951, 2924, 2852, 1730, 1613, 1523, 1435, 1360, 1159, 1005, 838, 768, 726, 542. **ESI-HRMS**: m/z calcd. for $C_{17}H_{15}NO_3Na$ $[M+Na]^+$ 304.0944, found 304.0944.

Methyl 2-(2-(2-methoxyphenyl)indolizin-3-yl)acetate (2k)

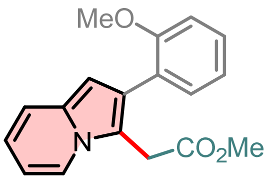

2k

Product **2k** was obtained following the general procedure **C** using 2-(2-methoxyphenyl)indolizine **1k** (44.6 mg, 0.20 mmol, 1.00 eq.) and 2-((2-methoxy-2-oxoethyl)thio)-3-methyl-4,5-dihydrothiazol-3-ium triflate **T1** (78.2 mg, 0.22 mmol, 1.10 eq.). The crude was purified by column chromatography on silica (PE:EtOAc, 9:1) to get the pure product as a yellow oil: 34.8 mg, 0.12 mmol, 59%. **^{1}H NMR** (400 MHz, $CDCl_3$) δ 7.83 (dd, J = 7.2, 1.2 Hz, 1H), 7.42 (dt, J = 7.4, 1.8 Hz, 2H), 7.36 (ddd, J = 8.3, 7.5, 1.8 Hz, 1H), 7.07 (td, J = 7.5, 1.1 Hz, 1H), 7.02 (dd, J = 8.3, 1.1 Hz, 1H), 6.72 (ddd, J = 8.9, 6.5, 1.0 Hz, 1H), 6.61 – 6.53 (m, 2H), 3.88 (s, 2H), 3.79 (s, 3H), 3.74 (s, 3H). **^{13}C{^{1}H} NMR** (100 MHz, $CDCl_3$) δ 171.0, 156.7, 132.6, 132.3, 128.5, 125.5, 124.9, 122.3, 120.8, 119.2, 116.7, 114.3, 111.0, 110.5, 100.3, 55.5, 52.2, 31.5. **IR** (neat, cm^{-1}): 3020, 2948, 2846, 1727, 1491, 1432, 1361, 1296, 1242, 1024, 1000, 858, 820, 764, 724, 590, 557, 499, 431. **ESI-HRMS**: m/z calcd. for $C_{18}H_{17}NO_3Na$ $[M+Na]^+$ 318.1101, found 318.1101.

Methyl 2-(2-(4-(methylsulfonyl)phenyl)indolizin-3-yl)acetate (2l)

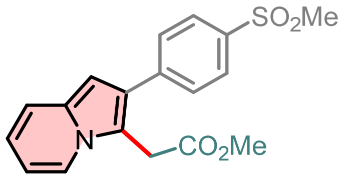

2l

Product **2l** was obtained following the general procedure **D** using 2-(4-(methylsulfonyl)phenyl)indolizine **1l** (54.2 mg, 0.20 mmol, 1.00 eq.) and 2-((2-methoxy-2-oxoethyl)thio)-3-methyl-4,5-dihydrothiazol-3-ium triflate **T1** (78.2 mg, 0.22 mmol, 1.10 eq.). The crude was purified by column chromatography on silica (PE:EtOAc, 2:1) to get the pure product as a yellow solid: 53.0 mg, 0.15 mmol, 77%. **^{1}H NMR** (400 MHz, $CDCl_3$) δ 8.06 – 7.98 (m, 2H), 7.98 – 7.88 (m, 1H), 7.84 – 7.72 (m, 2H), 7.43 (dd, J = 9.0, 1.4 Hz, 1H), 6.78 (dd, J = 9.0, 6.5 Hz, 1H), 6.70 – 6.56 (m, 2H), 3.97 (s, 2H), 3.77 (s, 3H), 3.11 (s, 3H).**^{13}C{^{1}H} NMR** (100 MHz, $CDCl_3$) δ 170.3, 142.0, 138.4, 135.3, 133.1, 129.8, 127.8, 125.1, 122.6, 119.3, 117.9, 113.2, 111.5, 99.2, 52.6, 44.7, 31.3. **IR** (neat, cm^{-1}): 2923, 2852, 1732, 1434, 1340, 1300, 1143, 959, 850, 765, 734, 581, 541, 515. **ESI-HRMS**: m/z calcd. for $C_{18}H_{17}NSO_4Na$ $[M+Na]^+$ 366.0770, found 366.0770.

Methyl 2-(2-(thiophen-2-yl)indolizin-3-yl)acetate (2m)

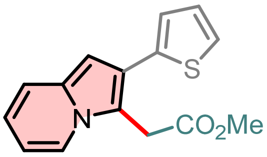

2m

Product **2m** was obtained following the general procedure **C** using 22-(thiophen-2-yl)indolizine **1m** (39.8 mg, 0.20 mmol, 1.00 eq.) and 2-((2-methoxy-2-oxoethyl)thio)-3-methyl-4,5-dihydrothiazol-3-ium triflate **T1** (78.2 mg, 0.22 mmol, 1.10 eq.). The crude was purified by column chromatography on silica (PE:EtOAc, 95:5) to get the pure product as a yellow oil: 34.0 mg, 0.13 mmol, 63%. **^{1}H NMR** (400 MHz, $CDCl_3$) δ 7.92 (dd, J = 7.1, 1.2 Hz, 1H), 7.39 (d, J = 8.9 Hz, 1H), 7.33 (dd, J = 5.2, 1.2 Hz, 1H), 7.28 (dd, J = 3.5, 1.2 Hz, 1H), 7.14 (dd, J = 5.2, 3.5 Hz, 1H), 6.75 (ddd, J = 9.0, 6.5, 1.0 Hz, 1H), 6.64 (d, J = 0.8 Hz, 1H), 6.60 (td, J = 6.8, 1.3 Hz, 1H), 4.11 (s, 2H), 3.75 (s, 3H). **^{13}C{^{1}H} NMR** (100 MHz, $CDCl_3$) δ 170.4, 138.1, 132.9, 127.9, 125.1, 124.9, 122.5, 119.0, 117.6, 112.8, 111.1, 99.2, 52.5, 31.4. **IR** (neat, cm^{-1}): 3105, 2949, 2844, 1731, 1433, 1362, 1323, 1151, 996, 847, 765, 697, 432. **ESI-HRMS**: m/z calcd. for $C_{15}H_{13}NSO_2Na$ $[M+Na]^+$ 294.0559, found 294.0559.

Methyl 2-(2-(3,4-difluorophenyl)-6-methylindolizin-3-yl)acetate (2n)

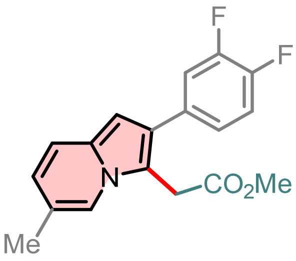

2n

Product **2n** was obtained following the general procedure **C** using 2-(3,4-difluorophenyl)-6-methylindolizine **1n** (48.6 mg, 0.20 mmol, 1.00 eq.) and 2-((2-methoxy-2-oxoethyl)thio)-3-methyl-4,5-dihydrothiazol-3-ium triflate **T1** (78.2 mg, 0.22 mmol, 1.10 eq.). The crude was purified by column chromatography on silica (PE:EtOAc, 95:5) to get the pure product as a colorless oil: 31.1 mg, 0.10 mmol, 49%. **^{1}H NMR** (400 MHz, $CDCl_3$) δ 7.71 (p, J = 1.3 Hz, 1H), 7.42 (ddd, J = 11.7, 7.7, 2.1 Hz, 1H), 7.34 (dd, J = 9.1, 1.0 Hz, 1H), 7.32 – 7.18 (m, 2H), 6.65 (dd, J = 9.1, 1.4 Hz, 1H), 6.51 (d, J = 0.8 Hz, 1H), 3.95 (s, 2H), 3.80 (s, 3H), 2.32 (s, 3H). **^{13}C{^{1}H} NMR** (100 MHz, $CDCl_3$) δ 170.7, 151.2 (dd, J = 86, 13 Hz), 148.7 (dd, J = 86, 12 Hz), 133.5, 133.5, 133.4, 133.4, 131.8, 127.5, 127.5, 125.1, 125.1, 125.0, 125.0, 121.0, 120.6, 119.9, 118.6, 118.0, 117.8, 117.5, 117.3, 112.4, 98.7, 52.6, 31.3, 18.8. **^{19}F{^{1}H} NMR** (377 MHz, $CDCl_3$) δ -138.35 (d, J = 21.5 Hz, 1F), -141.69 (d, J = 21.5 Hz, 1F). **IR** (neat, cm^{-1}): 2952, 2862, 1733, 1601, 1531, 1421, 1309, 1272, 1164, 1116, 1009, 897, 794, 772, 645, 574. **ESI-HRMS**: m/z calcd. for $C_{18}H_{15}NF_2O_2Na$ $[M+Na]^+$ 338.0963, found 338.0963.

Methyl 2-(6-bromo-2-(3,4-dimethoxyphenyl)indolizin-3-yl)acetate (2o)

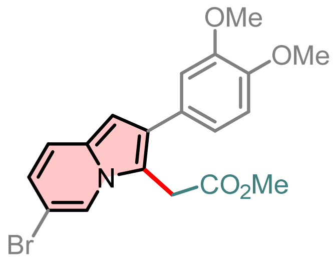

2o

Product **2o** was obtained following the general procedure **C** using 6-bromo-2-(3,4-dimethoxyphenyl)indolizine **1o** (66.4 mg, 0.20 mmol, 1.00 eq.) and 2-((2-methoxy-2-oxoethyl)thio)-3-methyl-4,5-dihydrothiazol-3-ium triflate **T1** (78.2 mg, 0.22 mmol, 1.10 eq.). The crude was purified by column chromatography on silica (PE:EtOAc, 4:1) to get the pure product as a yellow oil: 45.9 mg, 0.11 mmol, 57%. **^{1}H NMR** (400 MHz, $CDCl_3$) δ 8.10 (dt, J = 1.7, 1.0 Hz, 1H), 7.29 (dd, J = 9.4, 0.9 Hz, 1H), 7.16 (d, J = 2.0 Hz, 1H), 7.08 (dd, J = 8.2, 2.0 Hz, 1H), 6.96 (d, J = 8.2 Hz, 1H), 6.79 (dd, J = 9.4, 1.6 Hz, 1H), 6.60 (d, J = 0.8 Hz, 1H), 4.01 – 3.89 (m, 8H), 3.78 (s, 3H). **^{13}C{^{1}H} NMR** (100 MHz, $CDCl_3$)

δ 170.4, 149.0, 148.3, 131.1, 130.4, 128.2, 122.5, 121.3, 120.6, 119.6, 113.3, 112.6, 111.5, 105.8, 100.3, 56.0, 56.0, 52.6, 31.3. **IR** (neat, cm^{-1}): 3005, 2952, 2839, 1725, 1583, 1518, 1449, 1356, 1334, 1232, 1154, 1024, 970, 907, 870, 791, 763, 725, 702, 629, 589, 569. **ESI-HRMS**: m/z calcd. for $C_{19}H_{18}NBrO_4Na$ $[M+Na]^+$ 426.0311, found 426.0311.

Methyl 2-(2-methylindolizin-3-yl)acetate (2p)

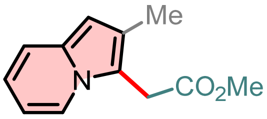

2p

Product **2p** was obtained following the general procedure **C** using 2-methylindolizine **1p** (26.2 mg, 0.20 mmol, 1.00 eq.) and 2-((2-methoxy-2-oxoethyl)thio)-3-methyl-4,5-dihydrothiazol-3-ium triflate **T1** (78.2 mg, 0.22 mmol, 1.10 eq.). The crude was purified by column chromatography on silica (PE:EtOAc, 7:1) to get the pure product as a yellow oil: 20.8 mg, 0.10 mmol, 51%. **^{1}H NMR** (400 MHz, $CDCl_3$) δ 7.83 (dd, J = 7.1, 1.2 Hz, 1H), 7.31 (d, J = 8.9 Hz, 1H), 6.66 (ddd, J = 9.0, 6.5, 1.1 Hz, 1H), 6.51 (td, J = 6.8, 1.4 Hz, 1H), 6.32 (s, 1H), 3.89 (s, 2H), 3.69 (s, 3H), 2.34 (s, 3H). **^{13}C{^{1}H} NMR** (100 MHz, $CDCl_3$) δ 170.5, 132.5, 123.7, 122.1, 118.4, 116.4, 114.6, 110.0, 99.9, 52.3, 30.5, 11.9. **IR** (neat, cm^{-1}): 2951, 1731, 1433, 1302, 1158, 1023, 761, 725, 426. **ESI-HRMS**: m/z calcd. for $C_{12}H_{13}NO_2Na$ $[M+Na]^+$ 226.0838, found 226.0834.

Methyl 2-(1-cyano-2-(*p*-tolyl)indolizin-3-yl)acetate (2q)

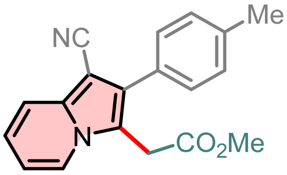

2q

Product **2q** was obtained following the general procedure **A** using 1-cyano-2-(p-tolyl)indolizine **1q** (46.4 mg, 0.20 mmol, 1.00 eq.) and 2-((2-methoxy-2-oxoethyl)thio)-3-methyl-4,5-dihydrothiazol-3-ium triflate **T1** (106.6 mg, 0.30 mmol, 1.50 eq.). The crude was purified by column chromatography on silica (PE:EtOAc, 4:1) to get the pure product as a white wax: 44.1 mg, 0.14 mmol, 72%. **^{1}H NMR** (400 MHz, $CDCl_3$) δ 8.02 (dt, J = 7.0, 1.1 Hz, 1H), 7.68 (dt, J = 9.0, 1.2 Hz, 1H), 7.55 – 7.42 (m, 2H), 7.31 (d, J = 7.8 Hz, 2H), 7.13 (ddd, J = 8.9, 6.7, 1.0 Hz, 1H), 6.85 (td, J = 6.9, 1.3 Hz, 1H), 3.94 (s, 2H), 3.76 (s, 3H), 2.42 (s, 3H). **^{13}C{^{1}H} NMR** (100 MHz, $CDCl_3$) δ 169.9, 138.2, 131.9, 129.8, 129.7, 129.5, 129.1,

127.4, 123.8, 122.5, 117.8, 116.9, 115.0, 113.4, 82.0, 52.7, 30.9, 21.4.**IR** (neat, cm^{-1}): 3111, 2946, 2922, 2852, 2204, 1735, 1505, 1436, 1393, 1338, 1204, 1175, 823, 748, 542, 499, 458. **ESI-HRMS**: m/z calcd. for $C_{19}H_{16}N_2O_2Na$ $[M+Na]^+$ 327.1104, found 327.1104.

Methyl 2-(1-cyano-2-(4-methoxyphenyl)indolizin-3-yl)acetate (2r)

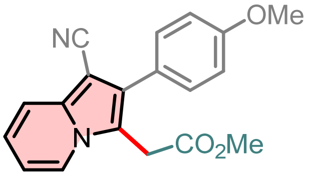

2r

Product **2r** was obtained following the general procedure **B** using 1-cyano-2-(4-methoxyphenyl)indolizine **1r** (49.6 mg, 0.20 mmol, 1.00 eq.) and 2-((2-methoxy-2-oxoethyl)thio)-3-methyl-4,5-dihydrothiazol-3-ium triflate **T1** (106.6 mg, 0.30 mmol, 1.50 eq.). The crude was purified by column chromatography on silica (PE:EtOAc, 3:1) to get the pure product as a yellow oil: 54.8 mg, 0.17 mmol, 86%. **^{1}H NMR** (400 MHz, $CDCl_3$) δ 8.02 (dt, J = 7.1, 1.2 Hz, 1H), 7.68 (dt, J = 9.0, 1.2 Hz, 1H), 7.57 – 7.41 (m, 2H), 7.13 (ddd, J = 8.9, 6.7, 1.0 Hz, 1H), 7.08 – 6.96 (m, 2H), 6.85 (td, J = 6.9, 1.3 Hz, 1H), 3.93 (s, 2H), 3.87 (s, 3H), 3.77 (s, 3H). **^{13}C{^{1}H} NMR** (100 MHz, $CDCl_3$) δ 170.0, 159.7, 138.2, 131.7, 130.9, 124.4, 123.8, 122.5, 117.8, 117.0, 114.9, 114.5, 113.4, 82.0, 55.5, 52.8, 30.9. **IR** (neat, cm^{-1}): 3081, 2944, 2843, 2207, 1740, 1612, 1504, 1435, 1395, 1339, 1291, 1245, 1205, 1171, 1028, 830, 748, 656, 531, 509. **ESI-HRMS**: m/z calcd. for $C_{19}H_{16}N_2O_3Na$ $[M+Na]^+$ 343.1053, found 343.1053.

Methyl 2-(2-(4-bromophenyl)-1-cyanoindolizin-3-yl)acetate (2s)

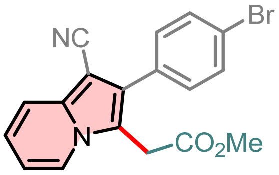

2s

Product **2s** was obtained following the general procedure **A** using 2-(4-bromophenyl)-1-cyanoindolizine **1s** (59.4 mg, 0.20 mmol, 1.00 eq.) and 2-((2-methoxy-2-oxoethyl)thio)-3-methyl-4,5-dihydrothiazol-3-ium triflate **T1** (106.6 mg, 0.30 mmol, 1.50 eq.). The crude was purified by column chromatography on silica (PE:EtOAc, 7:2) to get the pure product as a off-white solid: 38.9 mg, 0.11 mmol, 53%. **^{1}H NMR** (400 MHz, $CDCl_3$) δ 8.03 (dd, J = 7.0, 1.1 Hz, 1H), 7.69 (dt, J = 8.9, 1.2 Hz, 1H), 7.66 – 7.59 (m, 2H), 7.49 – 7.41 (m, 2H), 7.19 – 7.10 (m, 1H), 6.88 (td, J = 6.9, 1.3 Hz, 1H), 3.90 (s, 2H), 3.77 (s, 3H).**^{13}C{^{1}H}**

NMR (100 MHz, $CDCl_3$) δ 169.6, 138.3, 132.2, 131.3, 131.0, 130.7, 129.1, 123.9, 123.0, 122.9, 122.8, 118.0, 116.5, 115.2, 113.8, 81.9, 52.9, 30.8. **IR** (neat, cm^{-1}): 3085, 2922, 2852, 2207, 1738, 1490, 1434, 1409, 1390, 1337, 1318, 1204, 1173, 1077, 1012, 830, 747, 647, 555, 496, 457. **ESI-HRMS**: m/z calcd. for $C_{18}H_{13}N_2O_2BrNa$ $[M+Na]^+$ 391.0053, found 391.0053.

Methyl 2-(2-(4-chlorophenyl)-1-cyanoindolizin-3-yl)acetate (2t)

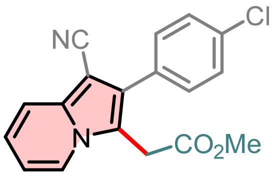

2t

Product **2t** was obtained following the general procedure **A** using 2-(4-chlorophenyl)-1-cyanoindolizine **1t** (50.2 mg, 0.20 mmol, 1.00 eq.) and 2-((2-methoxy-2-oxoethyl)thio)-3-methyl-4,5-dihydrothiazol-3-ium triflate **T1** (106.6 mg, 0.30 mmol, 1.50 eq.). The crude was purified by column chromatography on silica (PE:EtOAc, 7:2) to get the pure product as a white solid: 43.5 mg, 0.13 mmol, 67%. **^{1}H NMR** (400 MHz, $CDCl_3$) δ 8.04 (d, J = 6.9 Hz, 1H), 7.69 (dd, J = 9.0, 1.3 Hz, 1H), 7.59 – 7.49 (m, 2H), 7.49 – 7.42 (m, 2H), 7.19 – 7.11 (m, 1H), 6.88 (td, J = 6.9, 1.3 Hz, 1H), 3.91 (s, 2H), 3.77 (s, 3H). **^{13}C{^{1}H} NMR** (100 MHz, $CDCl_3$) δ 169.7, 138.2, 134.5, 131.0, 130.6, 130.5, 129.3, 129.2, 128.8, 123.9, 122.9, 117.9, 116.5, 115.3, 113.7, 81.9, 52.8, 30.8. **IR** (neat, cm^{-1}): 3084, 2922, 2851, 2208, 1737, 1489, 1434, 1393, 1338, 1204, 1157, 1093, 1014,833, 747, 652, 498, 459. **ESI-HRMS**: m/z calcd. for $C_{18}H_{13}N_2O_2ClNa$ $[M+Na]^+$ 347.0558, found 347.0558.

Methyl 2-(1-cyano-2-(4-(trifluoromethyl)phenyl)indolizin-3-yl)acetate (2u)

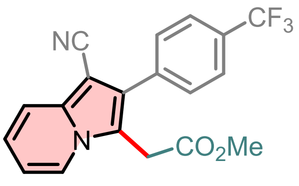

2u

Product **2u** was obtained following the general procedure **A** using 1-cyano-2-(4-(trifluoromethyl)phenyl)indolizine **1u** (57.2 mg, 0.20 mmol, 1.00 eq.) and 2-((2-methoxy-2-oxoethyl)thio)-3-methyl-4,5-dihydrothiazol-3-ium triflate **T1** (106.6 mg, 0.30 mmol, 1.50 eq.). The crude was purified by column chromatography on silica (PE:EtOAc, 4:1) to get the pure product as a white solid: 35.5 mg, 0.10 mmol, 50%. **^{1}H NMR** (400 MHz, $CDCl_3$) δ 8.07 (dt, J = 7.0, 1.2 Hz, 1H), 7.77 (d, J = 8.2 Hz, 2H), 7.74 – 7.68 (m, 3H), 7.18 (ddd, J = 8.9, 6.7, 1.0 Hz, 1H), 6.91 (td, J = 6.9, 1.3

Hz, 1H), 3.93 (s, 2H), 3.78 (s, 3H). **$^{13}C\{^1H\}$ NMR** (100 MHz, $CDCl_3$) δ 169.6, 149.9, 138.4, 135.8, 130.3 (t, J = 17 Hz), 130.1, 128.6, 126.0 (q, J = 4 Hz), 125.6, 124.0, 123.2, 122.8, 118.1, 116.4, 115.6, 113.9, 82.0, 52.9, 30.8. **$^{19}F\{^1H\}$ NMR** (377 MHz, $CDCl_3$) δ -63.10 (s, 3F). **IR** (neat, cm^{-1}): 3083, 2946, 2925, 2852, 2212, 1738, 1618, 1508, 1437, 1400, 1322, 1206, 1159, 1106, 1061, 1015, 862, 836, 748, 695, 647, 594, 500, 461, 413. **ESI-HRMS**: m/z calcd. for $C_{19}H_{13}F_3N_2O_2Na$ $[M+Na]^+$ 381.0821, found 381.0821.

Methyl 2-(1-cyano-2-(4-hydroxyphenyl)indolizin-3-yl)acetate (2v)

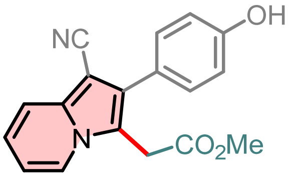

2v

Product **2v** was obtained following the general procedure **A** using 1-cyano-2-(4-hydroxyphenyl)indolizine **1v** (46.8 mg, 0.20 mmol, 1.00 eq.) and 2-((2-methoxy-2-oxoethyl)thio)-3-methyl-4,5-dihydrothiazol-3-ium triflate **T1** (106.6 mg, 0.30 mmol, 1.50 eq.). The crude was purified by column chromatography on silica (PE:EtOAc, 2:1 to 1:1) to get the pure product as an off-white solid: 41.6 mg, 0.14 mmol, 68%. **^1H NMR** (400 MHz, Acetone-d^6) δ 8.69 (s, 1H), 8.29 (dt, J = 7.0, 1.1 Hz, 1H), 7.66 (dt, J = 8.9, 1.2 Hz, 1H), 7.48 – 7.35 (m, 2H), 7.24 (ddd, J = 8.9, 6.7, 1.0 Hz, 1H), 7.04 – 6.93 (m, 3H), 4.06 (s, 2H), 3.71 (s, 3H). **$^{13}C\{^1H\}$ NMR** (100 MHz, Acetone-d^6) δ 170.6, 158.4, 138.4, 131.8, 131.6, 125.6, 124.2, 123.6, 117.7, 116.9, 116.8, 116.6, 116.5, 113.9, 82.1, 52.7, 30.8. **IR** (neat, cm^{-1}): 3280, 2953, 2923, 2852, 2203, 1732, 1613, 1504, 1439, 1416, 1344, 1266, 1207, 1170, 840, 732, 654, 529, 513, 465. **ESI-HRMS**: m/z calcd. for $C_{18}H_{14}N_2O_3Na$ $[M+Na]^+$ 329.0897, found 329.0897.

Methyl 2-(1-cyano-2-(2-methoxyphenyl)indolizin-3-yl)acetate (2w)

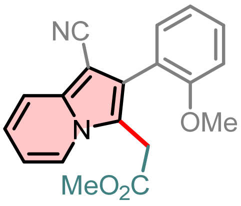

2w

Product **2w** was obtained following the general procedure **B** using 1-cyano-2-(2-methoxyphenyl)indolizine **1w** (49.6 mg, 0.20 mmol, 1.00 eq.) and 2-((2-methoxy-2-oxoethyl)thio)-3-methyl-4,5-dihydrothiazol-3-ium triflate **T1** (106.6 mg, 0.30 mmol, 1.50 eq.). The crude was purified

by column chromatography on silica (PE:EtOAc, 3:1) to get the pure product as a yellow oil: 57.5 mg, 0.18 mmol, 90%. ^{1}**H NMR** (400 MHz, $CDCl_3$) δ 7.96 (dt, J = 7.0, 1.1 Hz, 1H), 7.69 (dt, J = 8.9, 1.2 Hz, 1H), 7.48 – 7.36 (m, 2H), 7.14 – 7.01 (m, 3H), 6.83 (td, J = 6.9, 1.3 Hz, 1H), 3.82 (s, 2H), 3.81 (s, 3H), 3.74 (s, 3H). ^{13}**C{^{1}H} NMR** (100 MHz, $CDCl_3$) δ 170.0, 156.8, 138.1, 132.2, 130.1, 127.9, 123.8, 122.2, 121.1, 120.7, 117.9, 116.5, 113.1, 111.4, 111.4, 83.1, 55.6, 52.5, 31.0. **IR** (neat, cm^{-1}): 2921, 2852, 2210, 1739, 1712, 1459, 1377, 1245, 1160, 908, 734. **ESI-HRMS**: m/z calcd. for $C_{19}H_{16}N_2O_3Na$ $[M+Na]^+$ 343.1053, found 343.1053.

Methyl 2-(1-cyano-2-(thiophen-2-yl)indolizin-3-yl)acetate (2x)

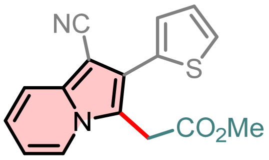

2x

Product **2x** was obtained following the general procedure **A** using 1-cyano-2-(thiophen-2-yl)indolizine **1x** (44.8 mg, 0.20 mmol, 1.00 eq.) and 2-((2-methoxy-2-oxoethyl)thio)-3-methyl-4,5-dihydrothiazol-3-ium triflate **T1** (106.6 mg, 0.30 mmol, 1.50 eq.). The crude was purified by column chromatography on silica (PE:EtOAc, 4:1) to get the pure product as a colorless oil: 42.4 mg, 0.14 mmol, 72%. ^{1}**H NMR** (400 MHz, $CDCl_3$) δ 8.02 (dq, J = 7.1, 0.9 Hz, 1H), 7.67 (dq, J = 9.0, 1.0 Hz, 1H), 7.44 (ddt, J = 7.8, 3.7, 1.0 Hz, 2H), 7.23 – 7.09 (m, 2H), 6.87 (tt, J = 6.9, 1.0 Hz, 1H), 4.08 (s, 2H), 3.76 (s, 3H).^{13}**C{^{1}H} NMR** (100 MHz, $CDCl_3$) δ 169.5, 138.4, 132.8, 128.0, 128.0, 127.0, 124.6, 123.9, 123.0, 117.9, 116.6, 115.5, 113.8, 82.1, 52.8, 31.0. **IR** (neat, cm^{-1}): 3111, 2951, 2922, 2852, 2214, 1740, 1502, 1431, 1393, 1314, 1205, 1155, 1039, 842 750, 694, 491, 447. **ESI-HRMS**: m/z calcd. for $C_{16}H_{12}N_2O_2SNa$ $[M+Na]^+$ 319.0512, found 319.0496.

Ethyl 1-cyano-3-(2-methoxy-2-oxoethyl)indolizine-2-carboxylate (2y)

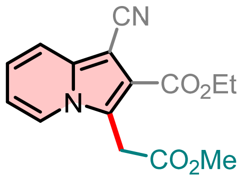

2y

Product **2y** was obtained following the general procedure **B** using ethyl 1-cyanoindolizine-2-carboxylate **1y** (42.8 mg, 0.20 mmol, 1.00 eq.) and 2-((2-methoxy-2-oxoethyl)thio)-3-methyl-4,5-dihydrothiazol-3-ium triflate **T1** (106.6 mg, 0.30 mmol, 1.50 eq.). The crude was purified by column chromatography on

silica (PE:EtOAc, 5:2) to get the pure product as a white solid: 25.7 mg, 0.09 mmol, 45%. **^{1}H NMR** (400 MHz, $CDCl_3$) δ 7.95 (dt, J = 7.2, 1.1 Hz, 1H), 7.69 (dt, J = 9.1, 1.3 Hz, 1H), 7.15 (ddd, J = 9.1, 6.7, 1.0 Hz, 1H), 6.89 (td, J = 6.9, 1.3 Hz, 1H), 4.46 – 4.41 (m, 4H), 3.71 (s, 3H), 1.45 (t, J = 7.1 Hz, 3H). **^{13}C{^{1}H} NMR** (100 MHz, $CDCl_3$) δ 169.2, 163.2, 138.3, 123.7, 123.7, 123.0, 118.9, 118.8, 115.4, 114.7, 83.1, 61.5, 52.7, 30.3, 14.2. **IR** (neat, cm^{-1}): 2994, 2959, 2851, 2217, 1723, 1699, 1556, 1514, 1431, 1346, 1269, 1248, 1211, 1165, 1020, 977, 821, 740, 494, 459, 426. **ESI-HRMS**: m/z calcd. for $C_{15}H_{14}N_2O_4Na$ $[M+Na]^+$ 309.0846, found 309.0846.

Methyl 2-(1-cyanoindolizin-3-yl)acetate + methyl 2-(1-cyanoindolizin-5-yl)acetate (2z + 2z')

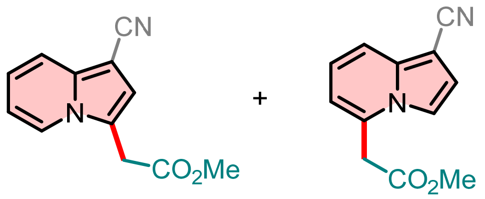

2z + 2z' (1:2)

Products **2z + 2z'** was obtained following the general procedure **A** using 1-cyanoindolizine **1z** (28.4 mg, 0.20 mmol, 1.00 eq.) and 2-((2-methoxy-2-oxoethyl)thio)-3-methyl-4,5-dihydrothiazol-3-ium triflate **T1** (106.6 mg, 0.30 mmol, 1.50 eq.). The crude was purified by column chromatography on silica (PE:EtOAc, 4:1) to get the pure product mixture as a colorless oil: 13.0 mg, 0.06 mmol, 30%. **^{1}H NMR** (400 MHz, $CDCl_3$, minor) δ 7.98 (dt, J = 7.1, 1.1 Hz, 1H), 7.66 (d, J = 9.0 Hz, 1H), 7.08 (d, J = 4.6 Hz, 1H), 6.96 (s, 1H), 6.83 (td, J = 6.9, 1.3 Hz, 1H), 3.92 (s, 2H), 3.73 (s, 3H). **^{13}C{^{1}H} NMR** (100 MHz, $CDCl_3$) δ 169.4, 168.6, 138.7, 138.6, 131.1, 123.8, 122.4, 122.2, 118.2, 118.0, 117.4, 117.3, 117.2, 116.9, 116.9, 114.6, 113.3, 111.6, 83.0, 81.5, 52.9, 52.7, 38.6, 32.1. **IR** (neat, cm^{-1}): 3143, 2951, 2847, 2210, 1722, 1524, 1435, 1344, 1313, 1291, 1252, 1162, 995, 777, 744, 701, 516. **ESI-HRMS**: m/z calcd. for $C_{12}H_{10}N_2O_2Na$ $[M+Na]^+$ 237.0634, found 237.0634.

Methyl 3-(2-methoxy-2-oxoethyl)-2-(4-methoxyphenyl)indolizine-1-carboxylate (2aa)

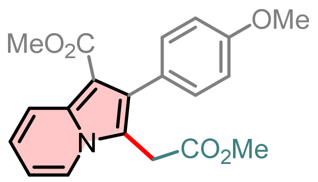

2aa

Product **2aa** was obtained following the general procedure **A** using methyl 2-(2-methoxyphenyl)indolizine-1-carboxylate **1aa** (56.2 mg, 0.20 mmol, 1.00 eq.) and 2-((2-methoxy-2-

oxoethyl)thio)-3-methyl-4,5-dihydrothiazol-3-ium triflate **T1** (106.6 mg, 0.30 mmol, 1.50 eq.). The crude was purified by column chromatography on silica (PE:EtOAc, 3:1) to get the pure product as a colorless oil: 41.1 mg, 0.12 mmol, 58%. **^{1}H NMR** (400 MHz, $CDCl_3$) δ 8.30 (dt, J = 9.1, 1.3 Hz, 1H), 7.95 (dt, J = 7.0, 1.2 Hz, 1H), 7.37 – 7.29 (m, 2H), 7.12 (ddd, J = 9.1, 6.7, 1.1 Hz, 1H), 7.02 – 6.93 (m, 2H), 6.82 (td, J = 6.8, 1.3 Hz, 1H), 3.87 (s, 3H), 3.79 (s, 2H), 3.74 (s, 3H), 3.71 (s, 3H). **^{13}C{^{1}H} NMR** (100 MHz, $CDCl_3$) δ 170.3, 165.4, 158.9, 136.3, 131.7, 131.7, 131.1, 126.7, 123.1, 122.4, 120.3, 116.6, 113.3, 113.2, 113.0, 102.0, 55.3, 52.5, 50.6, 30.7. **IR** (neat, cm^{-1}): 2998, 2950, 2836, 1735, 1682, 1613, 1503, 1440, 1393, 1242, 1172, 1065, 1029, 910, 832, 783, 727, 640, 525. **ESI-HRMS**: m/z calcd. for $C_{20}H_{19}NO_5Na$ $[M+Na]^+$ 376.1155, found 376.1155.

Methyl 3-(2-methoxy-2-oxoethyl)-2-(4-(trifluoromethyl)phenyl)indolizine-1-carboxylate (2ab)

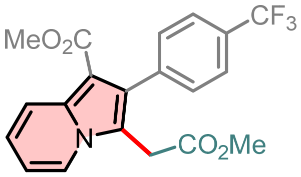

2ab

Product **2ab** was obtained following the general procedure **A** using methyl 2-(4-(trifluoromethyl)phenyl)indolizine-1-carboxylate **1ab** (63.8 mg, 0.20 mmol, 1.00 eq.) and 2-((2-methoxy-2-oxoethyl)thio)-3-methyl-4,5-dihydrothiazol-3-ium triflate **T1** (106.6 mg, 0.30 mmol, 1.50 eq.). The crude was purified by column chromatography on silica (PE:EtOAc, 6:1) to get the pure product as a colorless oil: 36.3 mg, 0.09 mmol, 46%. Alongside the pure product, a mixed fraction of **2ab** and its regioisomer, methyl 5-(2-methoxy-2-oxoethyl)-2-(4-(trifluoromethyl)phenyl)indolizine-1-carboxylate **2ab'**, was isolated: 10.0 mg, 0.03 mmol, 13%, 1.4:1 ratio respectively. **^{1}H NMR** (400 MHz, $CDCl_3$) δ 8.31 (dt, J = 9.1, 1.3 Hz, 1H), 7.98 (dt, J = 7.1, 1.2 Hz, 1H), 7.69 (d, J = 8.1 Hz, 2H), 7.52 (d, J = 8.0 Hz, 2H), 7.17 (ddd, J = 9.2, 6.7, 1.1 Hz, 1H), 6.87 (td, J = 6.8, 1.3 Hz, 1H), 3.76 (s, 2H), 3.73 – 3.72 (m, 6H). **^{13}C{^{1}H} NMR** (100 MHz, $CDCl_3$) δ 170.0, 165.1, 138.6, 136.4, 131.0, 130.5, 129.5 (q, J = 33 Hz), 125.9, 124.7 (q, J = 4 Hz), 123.3, 123.1, 123.0, 120.5, 116.7, 113.4, 102.0, 52.6, 50.8, 30.6. **^{19}F{^{1}H} NMR** (377 MHz, $CDCl_3$) δ -62.89 (s, 3F). **IR** (neat, cm^{-1}): 2952, 2925, 2853, 1737, 1686, 1619, 1505, 1444, 1395, 1321, 1119, 1058, 1018, 840, 784, 738, 688. **ESI-HRMS**: m/z calcd. for $C_{20}H_{16}F_3NO_4Na$ $[M+Na]^+$ 414.0924, found 414.0924.

Methyl 3-(2-methoxy-2-oxoethyl)-2-(thiophen-2-yl)indolizine-1-carboxylate (2ac)

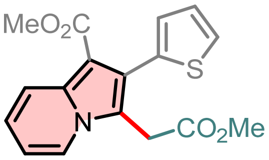

2ac

Product **2ac** was obtained following the general procedure **A** using methyl 2-(thiophen-2-yl)indolizine-1-carboxylate **1ac** (51.4 mg, 0.20 mmol, 1.00 eq.) and 2-((2-methoxy-2-oxoethyl)thio)-3-methyl-4,5-dihydrothiazol-3-ium triflate **T1** (106.6 mg, 0.30 mmol, 1.50 eq.). The crude was purified by column chromatography on silica (PE:EtOAc, 4:1) to get the pure product as a colorless oil: 49.4 mg, 0.15 mmol, 75%. **^{1}H NMR** (400 MHz, $CDCl_3$) δ 8.30 (dt, J = 9.2, 1.2 Hz, 1H), 7.96 (dt, J = 7.0, 1.1 Hz, 1H), 7.44 (dd, J = 4.6, 1.7 Hz, 1H), 7.18 – 7.06 (m, 3H), 6.83 (td, J = 6.8, 1.3 Hz, 1H), 3.89 (s, 2H), 3.78 (s, 3H), 3.72 (s, 3H). **^{13}C{^{1}H} NMR** (100 MHz, $CDCl_3$) δ 170.0, 165.0, 136.3, 135.0, 128.4, 126.6, 123.9, 123.2, 122.7, 120.4, 118.0, 113.3, 102.8, 52.5, 50.7, 30.7. **IR** (neat, cm^{-1}): 3104, 2949, 2851, 1734, 1682, 1505, 1435, 1391, 1319, 1245, 1170, 1122, 1009, 910, 838, 781, 697. **ESI-HRMS**: m/z calcd. for $C_{17}H_{15}NSO_4Na$ $[M+Na]^+$ 352.0614, found 352.0614.

Methyl 3-(2-methoxy-2-oxoethyl)-2-methylindolizine-1-carboxylate (2ad)

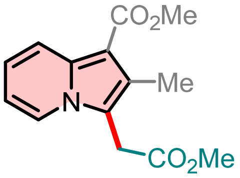

2ad

Product **2ad** was obtained following the general procedure **A** using methyl 2-methylindolizine-1-carboxylate **1ad** (37.8 mg, 0.20 mmol, 1.00 eq.) and 2-((2-methoxy-2-oxoethyl)thio)-3-methyl-4,5-dihydrothiazol-3-ium triflate **T1** (106.6 mg, 0.30 mmol, 1.50 eq.). The crude was purified by column chromatography on silica (PE:EtOAc, 6:1) to get the pure product as a colorless oil: 27.7 mg, 0.11 mmol, 53%. **^{1}H NMR** (400 MHz, $CDCl_3$) δ 8.29 – 8.12 (m, 1H), 7.94 (dq, J = 7.0, 1.0 Hz, 1H), 7.09 – 7.02 (m, 1H), 6.75 (tdd, J = 6.8, 1.4, 0.7 Hz, 1H), 3.93 – 3.87 (m, 5H), 3.68 (s, 3H), 2.52 (s, 3H). **^{13}C{^{1}H} NMR** (100 MHz, $CDCl_3$) δ 170.0, 166.1, 136.4, 126.9, 122.9, 121.9, 119.8, 115.6, 112.5, 102.3, 52.4, 50.6, 30.0, 11.7. **IR** (neat, cm^{-1}): 2951, 2848, 0735, 1679, 1505, 1436, 1392, 1321, 1213, 1107, 1056, 1028, 918, 781, 734, 645. **ESI-HRMS**: m/z calcd. for $C_{14}H_{15}NO_4Na$ $[M+Na]^+$ 284.0893, found 284.0893.

Dimethyl 3-(2-methoxy-2-oxoethyl)indolizine-1,2-dicarboxylate (2ae)

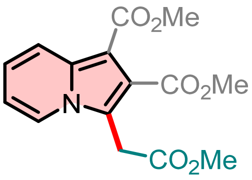

2ae

Product **2ae** was obtained following the general procedure **B** using dimethyl indolizine-1,2-dicarboxylate **1ae** (46.6 mg, 0.20 mmol, 1.00 eq.) and 2-((2-methoxy-2-oxoethyl)thio)-3-methyl-4,5-dihydrothiazol-3-ium triflate **T1** (106.6 mg, 0.30 mmol, 1.50 eq.). The crude was purified by column chromatography on silica (PE:EtOAc, 3:1) to get the pure product as a colorless oil: 25.6 mg, 0.08 mmol, 42%. **^{1}H NMR** (400 MHz, $CDCl_3$) δ 8.16 (d, J = 9.2 Hz, 1H), 7.92 (d, J = 7.1 Hz, 1H), 7.11 (ddd, J = 9.2, 6.6, 1.0 Hz, 1H), 6.82 (td, J = 6.9, 1.3 Hz, 1H), 4.08 (s, 2H), 3.94 (s, 3H), 3.88 (s, 3H), 3.69 (s, 3H). **^{13}C{^{1}H} NMR** (100 MHz, $CDCl_3$) δ 169.3, 166.3, 164.3, 135.7, 123.4, 123.3, 121.5, 120.5, 119.2, 113.9, 102.3, 52.7, 52.5, 51.4, 30.5. **IR** (neat, cm^{-1}): 2997, 2952, 2849, 1733, 1689, 1508, 1451, 1341, 1201, 1090, 994, 827, 735. **ESI-HRMS**: m/z calcd. for $C_{15}H_{15}NO_6Na$ $[M+Na]^+$ 328.0792, found 328.0792.

2-Ethyl 1-methyl 3-(2-methoxy-2-oxoethyl)indolizine-1,2-dicarboxylate (2af)

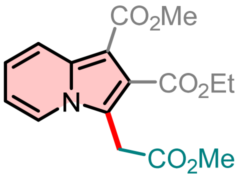

2af

Product **2af** was obtained following the general procedure **B** using 2-ethyl 1-methyl indolizine-1,2-dicarboxylate **1af** (49.4 mg, 0.20 mmol, 1.00 eq.) and 2-((2-methoxy-2-oxoethyl)thio)-3-methyl-4,5-dihydrothiazol-3-ium triflate **T1** (106.6 mg, 0.30 mmol, 1.50 eq.). The crude was purified by column chromatography on silica (PE:EtOAc, 3:1) to get the pure product as a yellow oil: 23.2 mg, 0.07 mmol, 37%. **^{1}H NMR** (400 MHz, $CDCl_3$) δ 8.22 – 8.14 (m, 1H), 7.93 (d, J = 7.1 Hz, 1H), 7.11 (ddd, J = 9.2, 6.7, 1.1 Hz, 1H), 6.82 (td, J = 6.9, 1.3 Hz, 1H), 4.42 (q, J = 7.1 Hz, 2H), 4.08 (s, 2H), 3.88 (s, 3H), 3.69 (s, 3H), 1.39 (t, J = 7.1 Hz, 3H). **^{13}C{^{1}H} NMR** (100 MHz, $CDCl_3$) δ 169.3, 165.8, 164.4, 135.8, 123.4, 123.2, 121.8, 120.5, 119.0, 113.8, 102.2, 61.5, 52.6, 51.3, 30.6, 14.3. **IR** (neat, cm^{-1}): 2983, 2952, 2844, 1733, 1691, 1509, 1448, 1203, 1091, 1015, 740. **ESI-HRMS**: m/z calcd. for $C_{16}H_{17}NO_6Na$ $[M+Na]^+$ 342.0948, found 342.0948.

Ethyl 3-(2-methoxy-2-oxoethyl)-2-(4-methoxyphenyl)indolizine-1-carboxylate (2ag)

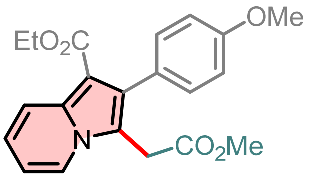

2ag

Product **2ag** was obtained following the general procedure **A** using ethyl 2-(2-methoxyphenyl)indolizine-1-carboxylate **1ag** (59.0 mg, 0.20 mmol, 1.00 eq.) and 2-((2-methoxy-2-oxoethyl)thio)-3-methyl-4,5-dihydrothiazol-3-ium triflate **T1** (106.6 mg, 0.30 mmol, 1.50 eq.). The crude was purified by column chromatography on silica (PE:EtOAc, 4:1) to get the pure product as a colorless oil: 32.0 mg, 0.09 mmol, 43%. **^{1}H NMR** (400 MHz, $CDCl_3$) δ 8.31 (dt, J = 9.1, 1.2 Hz, 1H), 7.94 (dt, J = 7.0, 1.1 Hz, 1H), 7.38 – 7.28 (m, 2H), 7.12 (ddd, J = 9.1, 6.7, 1.1 Hz, 1H), 7.02 – 6.92 (m, 2H), 6.82 (td, J = 6.8, 1.3 Hz, 1H), 4.21 (q, J = 7.1 Hz, 2H), 3.87 (s, 3H), 3.79 (s, 2H), 3.71 (s, 3H), 1.19 (t, J = 7.1 Hz, 3H). **^{13}C{^{1}H} NMR** (100 MHz, $CDCl_3$) δ 170.3, 165.0, 159.0, 136.2, 131.7, 131.7, 126.9, 123.1, 122.3, 120.3, 116.5, 113.2, 112.9, 102.3, 59.3, 55.4, 52.5, 30.7, 14.4. **IR** (neat, cm^{-1}): 2952, 2837, 1736, 1676, 1503, 1435, 1403, 1241, 1173, 1064, 1030, 830, 783, 734, 640, 525. **ESI-HRMS**: m/z calcd. for $C_{21}H_{21}NO_5Na$ $[M+Na]^+$ 390.1312, found 390.1312.

Ethyl 3-(2-methoxy-2-oxoethyl)-2-(thiophen-2-yl)indolizine-1-carboxylate (2ah)

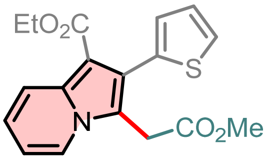

2ah

Product **2ah** was obtained following the general procedure **A** using ethyl 2-(thiophen-2-yl)indolizine-1-carboxylate **1ah** (54.2 mg, 0.20 mmol, 1.00 eq.) and 2-((2-methoxy-2-oxoethyl)thio)-3-methyl-4,5-dihydrothiazol-3-ium triflate **T1** (106.6 mg, 0.30 mmol, 1.50 eq.). The crude was purified by column chromatography on silica (PE:EtOAc, 4:1) to get the pure product as a colorless oil: 47.2 mg, 0.14 mmol, 69%. **^{1}H NMR** (400 MHz, $CDCl_3$) δ 8.30 (dt, J = 9.1, 1.2 Hz, 1H), 7.94 (dt, J = 7.0, 1.1 Hz, 1H), 7.42 (dd, J = 5.0, 1.4 Hz, 1H), 7.18 – 7.06 (m, 3H), 6.82 (td, J = 6.9, 1.3 Hz, 1H), 4.24 (q, J = 7.1 Hz, 2H), 3.88 (s, 2H), 3.70 (s, 3H), 1.21 (t, J = 7.1 Hz, 3H). **^{13}C{^{1}H} NMR** (100 MHz, $CDCl_3$) δ 170.0, 164.6, 136.3, 135.1, 128.4, 126.5, 126.4, 123.8, 123.1, 122.6, 120.3, 117.9, 113.2, 103.2, 59.4, 52.5, 30.7, 14.3. **IR** (neat, cm^{-1}): 3104, 2979, 2952, 2848, 1735, 1677, 1505, 1417, 1318, 1243, 1172, 1121, 1032, 910, 848, 781, 731. **ESI-HRMS**: m/z calcd. for $C_{18}H_{17}NSO_4Na$ $[M+Na]^+$ 366.0770, found 366.0770.

Ethyl 3-(2-methoxy-2-oxoethyl)indolizine-1-carboxylate + Ethyl 5-(2-methoxy-2-oxoethyl)indolizine-1-carboxylate (2ai + 2ai')

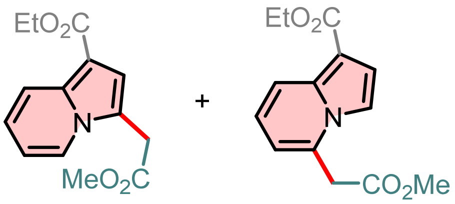

2ai + 2ai'

Product **2ai + 2ai'** was obtained following the general procedure **A** using ethyl indolizine-1-carboxylate **1ai** (37.8 mg, 0.20 mmol, 1.00 eq.) and 2-((2-methoxy-2-oxoethyl)thio)-3-methyl-4,5-dihydrothiazol-3-ium triflate **T1** (106.6 mg, 0.30 mmol, 1.50 eq.). The crude was purified by column chromatography on silica (PE:EtOAc, 4:1) to get the pure product mixture as a colorless oil: 14.8 mg, 0.06 mmol, 28%, 1.25:1 ratio. **^{1}H NMR** (400 MHz, $CDCl_3$) δ 8.23 (dd, J = 9.2, 1.3 Hz, 2H), 7.94 (d, J = 7.1 Hz, 1H), 7.33 (d, J = 3.1 Hz, 1H), 7.20 (d, J = 3.0 Hz, 2H), 7.15 – 7.01 (m, 2H), 6.78 (td, J = 6.8, 1.3 Hz, 1H), 6.69 (dd, J = 6.8, 1.2 Hz, 1H), 4.37 (p, J = 7.0 Hz, 4H), 3.92 (d, J = 5.3 Hz, 4H), 3.71 (d, J = 6.5 Hz, 5H), 1.41 (td, J = 7.1, 4.3 Hz, 5H). **^{13}C{^{1}H} NMR** (100 MHz, $CDCl_3$) δ 169.9, 169.0, 165.1, 165.0, 136.5, 136.4, 130.4, 123.4, 122.2, 122.0, 120.1, 119.2, 117.3, 116.8, 116.8, 114.0, 112.7, 111.0, 105.1, 103.6, 59.7, 59.6, 52.8, 52.5, 38.8, 32.3, 14.8.**IR** (neat, cm^{-1}): 2954, 1736, 1680, 1509, 1434, 1296, 1155, 1106, 1052, 776, 740. **ESI-HRMS**: m/z calcd. for $C_{14}H_{15}NO_4Na$ $[M+Na]^+$ 284.0893, found 284.0893.

Methyl 2-(1-(methylsulfonyl)-2-phenylindolizin-3-yl)acetate + methyl 2-(1-(methylsulfonyl)-2-phenylindolizin-5-yl)acetate (2aj + 2aj')

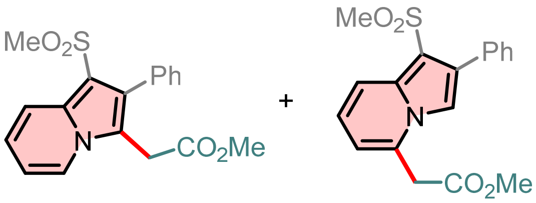

2aj + 2aj'

Product **2aj + 2aj'** was obtained following the general procedure **B** using 1-(methylsulfonyl)-2-phenylindolizine **1aj** (54.2 mg, 0.20 mmol, 1.00 eq.) and 2-((2-methoxy-2-oxoethyl)thio)-3-methyl-4,5-dihydrothiazol-3-ium triflate **T1** (106.6 mg, 0.30 mmol, 1.50 eq.). The crude was purified by column chromatography on silica (PE:EtOAc, 2:1) to get the pure product as a yellow oil: 30.4 mg, 0.09 mmol, 44%. **^{1}H NMR** (400 MHz, $CDCl_3$) δ 8.34 (dt, J = 9.2, 1.2 Hz, 1H), 7.96 (dd, J = 7.0, 1.2 Hz, 1H), 7.64 (dd, J = 7.8, 1.7 Hz, 1H), 7.51 – 7.39 (m, 7H), 7.20 – 7.10 (m, 2H), 6.89 (td, J = 6.8, 1.3 Hz, 1H), 3.96 (s, 1H), 3.77 (s, 2H), 3.75 (s, 1H), 3.72 (s, 3H), 2.86 (s, 1H), 2.81 (s, 3H). **^{13}C{^{1}H} NMR** (100 MHz, $CDCl_3$) δ 169.9, 168.7, 135.5, 134.2, 132.7, 132.6, 131.9, 131.2, 131.0, 130.7, 130.6, 130.0, 129.4,

129.2, 128.9, 128.5, 128.3, 128.3, 128.2, 123.1, 123.1, 123.0, 119.0, 118.4, 117.1, 114.9, 113.6, 111.5, 109.6, 109.0, 52.9, 52.7, 45.8, 45.7, 38.8, 38.6, 30.6. **IR** (neat, cm^{-1}): 2957, 2925, 2855, 1733, 1498, 1294, 1121, 1003, 956, 797, 748, 704,540, 511. **ESI-HRMS**: m/z calcd. for $C_{18}H_{17}NSO_4Na$ $[M+Na]^+$ 366.0770, found 366.0770.

Methyl 2-(1-(phenylsulfonyl)-2-phenylindolizin-3-yl)acetate + methyl 2-(1-(phenylsulfonyl)-2-phenylindolizin-5-yl)acetate (2ak + 2ak')

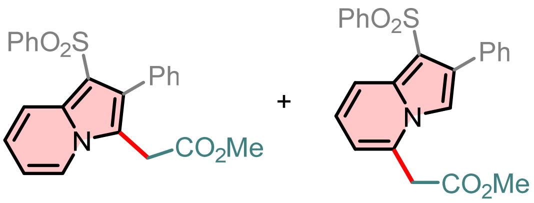

2ak + 2ak'

Product **2ak + 2ak'** was obtained following the general procedure **B** using 1-(phenylsulfonyl)-2-phenylindolizine **1ak** (66.6 mg, 0.20 mmol, 1.00 eq.) and 2-((2-methoxy-2-oxoethyl)thio)-3-methyl-4,5-dihydrothiazol-3-ium triflate **T1** (106.6 mg, 0.30 mmol, 1.50 eq.). The crude was purified by column chromatography on silica (PE:EtOAc, 4:1 to 3:1) to get the pure product mixture as a colorless oil: 32.7 mg, 0.08 mmol, 40%. **^{1}H NMR** (400 MHz, $CDCl_3$) δ 8.56 – 8.43 (m, 2H), 7.94 (dt, J = 7.0, 1.1 Hz, 1H), 7.59 – 7.50 (m, 1H), 7.50 – 7.42 (m, 2H), 7.43 – 7.30 (m, 8H), 7.27 – 7.12 (m, 7H), 6.88 (td, J = 6.9, 1.3 Hz, 1H), 6.80 (dd, J = 6.9, 1.2 Hz, 1H), 3.91 (s, 1H), 3.71 (s, 2H), 3.68 (s, 2H), 3.65 (s, 3H). **^{13}C{^{1}H} NMR** (100 MHz, $CDCl_3$) δ 169.7, 168.7, 144.2, 144.2, 135.6, 134.4, 132.7, 132.1, 132.0, 131.9, 131.5, 131.3, 130.8, 130.2, 130.0, 128.5, 128.5, 128.1, 128.0, 127.7, 127.7, 126.6, 126.5, 123.3, 123.3, 123.2, 119.1, 118.4, 117.3, 114.9, 113.5, 111.8, 110.0, 109.4, 52.9, 52.5, 38.5, 30.4. **IR** (neat, cm^{-1}): 3059, 2951, 2850, 1734, 1497, 1445, 1290, 1136, 1086, 998, 912, 719, 687, 629, 589, 550. **ESI-HRMS**: m/z calcd. for $C_{23}H_{19}NSO_4Na$ $[M+Na]^+$ 428.0927, found 428.0918.

Methyl 2-(2-phenyl-1-tosylindolizin-3-yl)acetate + methyl 2-(2-phenyl-1-tosylindolizin-5-yl)acetate (2al + 2al')

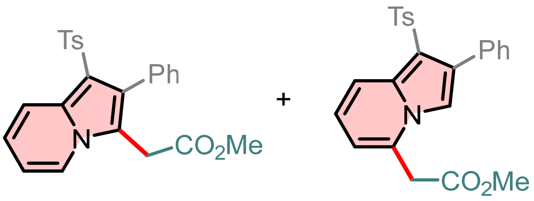

2al + 2al'

Product **2al + 2al'** was obtained following the general procedure **B** using 2-phenyl-1-tosylindolizine **1al** (69.4 mg, 0.20 mmol, 1.00 eq.) and 2-((2-methoxy-2-oxoethyl)thio)-3-methyl-4,5-dihydrothiazol-3-ium triflate **T1** (106.6 mg, 0.30 mmol, 1.50 eq.). The crude was purified by column chromatography on silica (PE:EtOAc, 3:1) to get the pure product mixture as a yellow oil: 34.3 mg, 0.08 mmol, 41%, 1.67:1 ratio. **^{1}H NMR** (400 MHz, $CDCl_3$) δ 8.54 – 8.41 (m, 2H), 7.93 (dt, J = 7.0, 1.2 Hz, 1H), 7.47 – 7.29 (m, 10H), 7.25 – 7.11 (m, 4H), 7.04 (dd, J = 8.1, 4.4 Hz, 3H), 6.87 (td, J = 6.9, 1.3 Hz, 1H), 6.78 (dd, J = 6.9, 1.1 Hz, 1H), 3.90 (s, 1H), 3.71 (s, 2H), 3.68 (s, 2H), 3.65 (s, 3H), 2.31 (d, J = 3.0 Hz, 5H). **^{13}C{^{1}H} NMR** (100 MHz, $CDCl_3$) δ 169.8, 168.7, 142.8, 142.7, 141.5, 141.4, 135.5, 134.2, 132.8, 132.0, 131.4, 131.3, 130.8, 130.1, 129.9, 129.2, 129.1, 128.1, 127.9, 127.7, 127.7, 126.7, 126.5, 123.3, 123.2, 123.0, 119.1, 118.4, 117.2, 114.8, 113.4, 111.7, 110.4, 109.8, 52.8, 52.5, 38.5, 30.5, 21.5, 21.2. **IR** (neat, cm^{-1}): 3057, 2952, 2852, 1735, 1494, 1288, 1135, 1087, 912, 729, 700, 658, 577, 536. **ESI-HRMS**: m/z calcd. for $C_{24}H_{21}NSO_4Na$ $[M+Na]^+$ 442.1084, found 442.1084.

Methyl 2-(2-methyl-1-(phenylsulfonyl)indolizin-3-yl)acetate + methyl 2-(2-methyl-1-(phenylsulfonyl)indolizin-5-yl)acetate (2am + 2am')

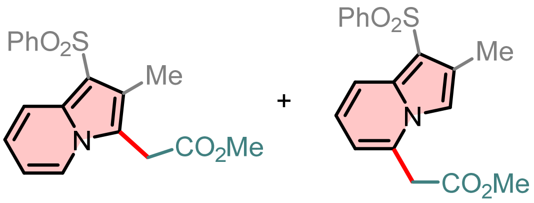

2am + 2am'

Product **2am + 2am'** was obtained following the general procedure **B** using 2-methyl-1-(phenylsulfonyl)indolizine **1am** (54.2 mg, 0.20 mmol, 1.00 eq.) and 2-((2-methoxy-2-oxoethyl)thio)-3-methyl-4,5-dihydrothiazol-3-ium triflate **T1** (106.6 mg, 0.30 mmol, 1.50 eq.). The crude was purified by column chromatography on silica (PE:EtOAc, 3:1) to get the pure product mixture as a colorless oil: 29.5 mg, 0.09 mmol, 43%, 2.9:1 ratio. **^{1}H NMR** (400 MHz, $CDCl_3$, major) δ 8.23 (dt, J = 9.1, 1.2 Hz, 1H), 7.86 – 7.82 (m, 2H), 7.40 – 7.30 (m, 3H), 7.08 – 7.00 (m, 1H), 6.73 (td, J = 6.8, 1.3 Hz, 1H), 3.75 (s, 2H), 3.58 (s, 3H), 2.34 (s, 3H). **^{13}C{^{1}H} NMR** (100 MHz, $CDCl_3$) δ 169.6, 168.8, 144.7, 144.6, 135.7, 134.8, 132.3, 132.2, 130.0, 129.0, 126.6, 126.1, 126.1, 124.5, 123.2, 122.9, 122.9, 118.0, 117.2, 116.3, 114.4, 113.1, 111.2, 109.5, 108.4, 52.8, 52.5, 38.6, 30.0, 11.8, 10.4. **IR** (neat, cm^{-1}): 2952, 2927, 2851, 1733, 1497, 1444, 1296, 1148, 1125, 1080, 720, 688, 588, 546. **ESI-HRMS**: m/z calcd. for $C_{18}H_{17}NSO_4Na$ $[M+Na]^+$ 366.0770, found 366.0770.

Methyl 2-(2-isopropyl-1-(phenylsulfonyl)indolizin-5-yl)acetate + methyl 2-(2-isopropyl-1-(phenylsulfonyl)indolizin-3-yl)acetate (2an + 2an')

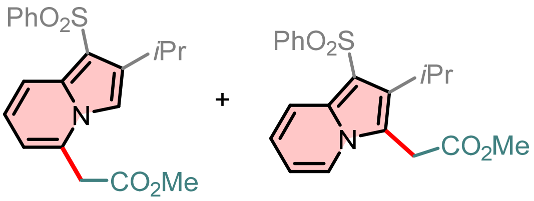

2an + 2an'

Product **2an** + **2an'** was obtained following the general procedure **A** using 2-isopropyl-1-(phenylsulfonyl)indolizine **1an** (59.8 mg, 0.20 mmol, 1.00 eq.) and 2-((2-methoxy-2-oxoethyl)thio)-3-methyl-4,5-dihydrothiazol-3-ium triflate **T1** (106.6 mg, 0.30 mmol, 1.50 eq.). The crude was purified by column chromatography on silica (PE:EtOAc, 3:1) to get the pure product mixture as a colorless oil: 32.4 mg, 0.09 mmol, 44%, 3:1 ratio. **^{1}H NMR** (400 MHz, $CDCl_3$, major) δ 8.29 (dd, J = 9.2, 1.2 Hz, 1H), 7.91 (dd, J = 8.3, 1.6 Hz, 2H), 7.50 – 7.38 (m, 3H), 7.09 (s, 1H), 6.73 (dd, J = 6.9, 1.2 Hz, 1H), 3.89 (s, 2H), 3.72 (s, 3H), 3.63 (p, J = 6.8 Hz, 1H), 1.22 (d, J = 6.8 Hz, 6H). **^{13}C{^{1}H} NMR** (100 MHz, $CDCl_3$) δ 169.7, 168.8, 145.1, 145.0, 138.9, 135.5, 134.6, 132.2, 132.2, 130.3, 129.0, 129.0, 126.1, 122.8, 122.8, 122.8, 118.7, 117.6, 115.6, 114.4, 113.2, 108.9, 108.2, 107.4, 52.8, 52.6, 38.6, 25.1, 24.7, 22.6. **IR** (neat, cm^{-1}): 2958, 2927, 2853, 1736, 1493, 1444, 1280, 1139, 1082, 788, 750, 717, 688, 668, 616, 572, 551. **ESI-HRMS**: m/z calcd. for $C_{20}H_{21}NSO_4Na$ $[M+Na]^+$ 394.1084, found 394.1082.

Methyl 2-(7-methoxy-1-(methylsulfonyl)-2-phenylindolizin-3-yl)acetate (2ao)

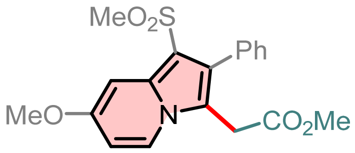

2ao

Product **2ao** was obtained following the general procedure **B** using 7-methoxy-1-(methylsulfonyl)-2-phenylindolizine **1ao** (60.2 mg, 0.20 mmol, 1.00 eq.) and 2-((2-methoxy-2-oxoethyl)thio)-3-methyl-4,5-dihydrothiazol-3-ium triflate **T1** (106.6 mg, 0.30 mmol, 1.50 eq.). The crude was purified by column chromatography on silica (PE:EtOAc, 3:1 to 2:1) to get the pure product as a yellow wax: 35.4 mg, 0.09 mmol, 48%. **^{1}H NMR** (400 MHz, $CDCl_3$) δ 7.82 (dd, J = 7.6, 0.7 Hz, 1H), 7.62 (d, J = 2.6 Hz, 1H), 7.50 – 7.39 (m, 5H), 6.59 (dd, J = 7.6, 2.7 Hz, 1H), 3.90 (s, 3H), 3.71 (s, 3H), 3.70 (s, 2H), 2.77 (s, 3H). **^{13}C{^{1}H} NMR** (100 MHz, $CDCl_3$) δ 170.1, 156.4, 136.1, 132.1, 131.2, 128.9, 128.3, 128.2, 128.2, 124.5, 115.7, 108.6, 106.7, 95.8, 55.7, 52.6, 45.7, 30.5. **IR** (neat, cm^{-1}): 2961, 2921, 2854, 1742, 1650, 1442,

1342, 1286, 1206, 1171, 1124, 1070, 1023, 1001, 945, 831, 794, 763, 702, 641, 555, 517, 502, 427. **ESI-HRMS**: m/z calcd. for $C_{19}H_{19}NSO_5Na$ $[M+Na]^+$ 396.0876, found 396.0876.

Methyl 2-(7-methoxy-1-(methylsulfonyl)-2-methylindolizin-3-yl)acetate (2ap)

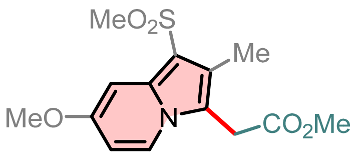

2ap

Product **2ap** was obtained following the general procedure **A** using 7-methoxy-1-(methylsulfonyl)-2-methylindolizine **1ap** (47.8 mg, 0.20 mmol, 1.00 eq.) and 2-((2-methoxy-2-oxoethyl)thio)-3-methyl-4,5-dihydrothiazol-3-ium triflate **T1** (106.6 mg, 0.30 mmol, 1.50 eq.). The crude was purified by column chromatography on silica (PE:EtOAc, 2:1) to get the pure product as a white wax: 39.0 mg, 0.13 mmol, 63%. **^{1}H NMR** (400 MHz, $CDCl_3$) δ 7.82 (d, J = 7.6 Hz, 1H), 7.40 (d, J = 2.6 Hz, 1H), 6.51 (dd, J = 7.6, 2.7 Hz, 1H), 3.86 (s, 3H), 3.81 (s, 2H), 3.68 (s, 3H), 3.06 (s, 3H), 2.42 (s, 3H). **^{13}C{^{1}H} NMR** (100 MHz, $CDCl_3$) δ 169.8, 156.3, 136.4, 124.5, 123.4, 114.6, 107.8, 105.7, 95.0, 55.7, 52.5, 45.9, 29.9, 10.3. **IR** (neat, cm^{-1}): 2924, 2852, 1728, 1647, 1504, 1485, 1409, 1345, 1275, 1203, 1154, 1105, 1032, 994, 952, 840, 792, 749, 708, 581, 556, 510. **ESI-HRMS**: m/z calcd. for $C_{14}H_{17}NSO_5Na$ $[M+Na]^+$ 334.0720, found 334.0720.

Methyl 2-(6-oxo-5,6-dihydroindolizino[1,2-*c*]quinolin-12-yl)acetate (2aq)

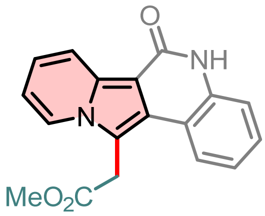

2aq

Product **2aq** was obtained following the general procedure **A** using 6-oxo-5,6-dihydroindolizino[1,2-*c*]quinoline **1aq** (48.6 mg, 0.20 mmol, 1.00 eq.) and 2-((2-methoxy-2-oxoethyl)thio)-3-methyl-4,5-dihydrothiazol-3-ium triflate **T1** (106.6 mg, 0.30 mmol, 1.50 eq.). The crude was purified by column chromatography on silica (PE:EtOAc, 1:1 to 2:3) to get the pure product as a off-white solid: 38.3 mg, 0.12 mmol, 62%. **^{1}H NMR** (400 MHz, DMSO-d^6) δ 11.04 (s, 1H), 8.65 (d, J = 7.0 Hz, 1H), 8.42 (dt, J = 8.8, 1.3 Hz, 1H), 8.10 (d, J = 8.0 Hz, 1H), 7.42 (d, J = 4.2 Hz, 2H), 7.34 – 7.26 (m, 1H), 7.23 (dt, J = 8.3, 4.2 Hz, 1H), 7.15 (td, J = 6.8, 1.5 Hz, 1H), 4.67 (s, 2H), 3.68 (s, 3H). **^{13}C{^{1}H} NMR** (100 MHz,

DMSO-d^6) δ 170.1, 159.6, 137.8, 131.2, 127.8, 123.8, 123.5, 123.1, 121.6, 121.2, 118.7, 116.2, 115.7, 114.2, 111.1, 102.2, 52.2, 31.0. **IR** (neat, cm^{-1}): 3402, 2852, 1724, 1656, 1589, 1504, 1422, 1319, 1154, 993, 731, 703, 549, 471. **ESI-HRMS**: m/z calcd. for $C_{18}H_{14}N_2O_3Na$ $[M+Na]^+$ 329.0897, found 329.0897.

Methyl 2-(1-phenylindolizin-3-yl)acetate (2ar)

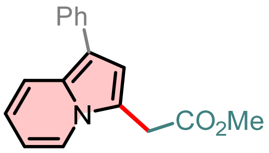

2ar

Product **2ar** was obtained following the general procedure **A** using 1-phenyl-2-(trimethylsilyl)indolizine **1ar** (53.0 mg, 0.20 mmol, 1.00 eq.) and 2-((2-methoxy-2-oxoethyl)thio)-3-methyl-4,5-dihydrothiazol-3-ium triflate **T1** (106.6 mg, 0.30 mmol, 1.50 eq.). The crude was purified by column chromatography on silica (PE:EtOAc, 7:1) to get the pure desililated product as a yellow oil: 11.6 mg, 0.04 mmol, 21%. **^{1}H NMR** (400 MHz, $CDCl_3$) δ 7.77 (dt, J = 7.1, 1.1 Hz, 1H), 7.67 (dt, J = 9.1, 1.3 Hz, 1H), 7.53 – 7.47 (m, 2H), 7.33 (dd, J = 8.5, 7.0 Hz, 2H), 7.17 – 7.10 (m, 1H), 6.84 (s, 1H), 6.68 (ddd, J = 9.1, 6.5, 1.1 Hz, 1H), 6.51 (ddd, J = 7.6, 6.6, 1.3 Hz, 1H), 3.88 (s, 2H), 3.64 (s, 3H). **^{13}C{^{1}H} NMR** (100 MHz, $CDCl_3$) δ 170.3, 136.4, 129.9, 128.8, 127.6, 125.4, 122.7, 118.5, 117.6, 116.4, 114.3, 111.2, 52.5, 32.6. **IR** (neat, cm^{-1}): 2950, 1730, 1599, 1514, 1434, 1155, 1009, 764, 697, 421. **ESI-HRMS**: m/z calcd. for $C_{17}H_{15}NO_2Na$ $[M+Na]^+$ 288.0995, found 288.0998.

Methyl 2-(indolizin-3-yl)acetate (2as)

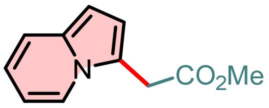

2as

Product **2as** was obtained following the general procedure **C** using indolizine **1as** (23.4 mg, 0.20 mmol, 1.00 eq.) and 2-((2-methoxy-2-oxoethyl)thio)-3-methyl-4,5-dihydrothiazol-3-ium triflate **T1** (78.2 mg, 0.22 mmol, 1.10 eq.). The crude was purified by column chromatography on silica (PE:EtOAc, 7:1) to get the pure product as a colorless oil: 2.1 mg, 0.01 mmol, 6%. **^{1}H NMR** (400 MHz, $CDCl_3$) δ 7.84 (dt, J = 7.1, 1.1 Hz, 1H), 7.40 (dt, J = 9.0, 1.3 Hz, 1H), 6.72 (d, J = 3.9 Hz, 1H), 6.68 (ddd, J = 9.0, 6.5, 1.2 Hz, 1H), 6.56 (td, J = 6.8, 1.3 Hz, 1H), 6.44 (dd, J = 3.9, 0.9 Hz, 1H), 3.94 (s, 2H), 3.70 (s, 3H). **^{13}C{^{1}H} NMR** (100 MHz, $CDCl_3$) δ 170.5, 133.5, 122.3, 119.5, 116.4, 114.4, 110.6, 98.7, 52.4, 32.7. **IR** (neat,

cm^{-1}): 2951, 2924, 2852, 1730, 1434, 1155, 1007, 726, 418. **ESI-HRMS**: m/z calcd. for $C_{11}H_{11}NO_2Na$ $[M+Na]^+$ 212.0682, found 212.0677.

Methyl 2-(2-(4-methoxyphenyl)-2*H*-indazol-3-yl)acetate (2at)

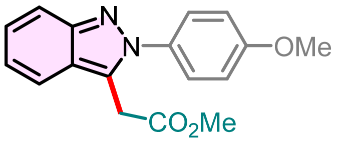

2at

Product **2at** was obtained following the general procedure **A** using 2-(4-methoxyphenyl)-2*H*-indazole **1at** (44.8 mg, 0.20 mmol, 1.00 eq.) and 2-((2-methoxy-2-oxoethyl)thio)-3-methyl-4,5-dihydrothiazol-3-ium triflate **T1** (106.6 mg, 0.30 mmol, 1.50 eq.). The crude was purified by column chromatography on silica (PE:EtOAc, 4:1) to get the pure product as a yellow oil: 26.1 mg, 0.08 mmol, 44%. **^{1}H NMR** (400 MHz, $CDCl_3$) δ 7.75 (dd, J = 8.8, 1.0 Hz, 1H), 7.65 (dt, J = 8.5, 1.1 Hz, 1H), 7.55 – 7.46 (m, 2H), 7.35 (ddd, J = 8.8, 6.6, 1.1 Hz, 1H), 7.14 (ddd, J = 8.7, 6.6, 0.9 Hz, 1H), 7.08 – 7.00 (m, 2H), 4.03 (s, 2H), 3.89 (s, 3H), 3.70 (s, 3H). **^{13}C{^{1}H} NMR** (100 MHz, $CDCl_3$) δ 169.5, 160.2, 148.6, 132.5, 128.3, 127.6, 126.9, 122.0, 121.8, 119.7, 117.9, 114.5, 55.8, 52.6, 31.5. **IR** (neat, cm^{-1}): 2922, 2853, 1730, 1631, 1514, 1437, 1396, 1379, 1336, 1289, 1248, 1206, 1178, 1107, 1030, 985, 833, 778, 752, 666, 636, 596, 541, 503. **ESI-HRMS**: m/z calcd. for $C_{17}H_{16}N_2O_3Na$ $[M+Na]^+$ 319.1053, found 319.1053.

Ethyl (*S*)-2-acetamido-3-(2-(2-methoxy-2-oxoethyl)-1*H*-indol-3-yl)propanoate (2au)

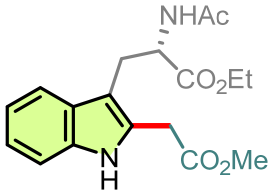

2au

Product **2au** was obtained following the general procedure **A** using ethyl acetyl-*L*-tryptophanate **1au** (54.8 mg, 0.20 mmol, 1.00 eq.) and 2-((2-methoxy-2-oxoethyl)thio)-3-methyl-4,5-dihydrothiazol-3-ium triflate **T1** (106.6 mg, 0.30 mmol, 1.50 eq.). The crude was purified by column chromatography on silica (PE:EtOAc, 1:3) to get the pure product as a pink oil: 49.1 mg, 0.14 mmol, 71%. **^{1}H NMR** (400 MHz, $CDCl_3$) δ 8.61 (s, 1H), 7.50 (dd, J = 7.9, 1.1 Hz, 1H), 7.32 (dt, J = 8.1, 1.0 Hz, 1H), 7.17 (ddd, J = 8.2, 7.0, 1.2 Hz, 1H), 7.10 (ddd, J = 8.0, 7.0, 1.1 Hz, 1H), 6.30 (d, J = 7.6 Hz, 1H), 4.87 (dt, J = 7.6, 5.9 Hz, 1H), 4.21 – 4.01 (m, 2H), 3.79 (s, 2H), 3.78 (s, 3H), 3.32 – 3.20 (m, 2H), 1.95 (s, 3H), 1.19 (t, J = 7.1 Hz, 3H). **^{13}C{^{1}H} NMR** (100 MHz, $CDCl_3$) δ 172.3, 171.3, 170.1, 135.8, 128.3, 128.3, 122.4, 119.9, 118.6, 111.0, 108.1, 61.7, 53.1, 52.7, 31.7, 26.9, 23.2, 14.1. **IR** (neat, cm^{-1}): 3334, 3233, 2922, 2853,

1742, 1718, 1526, 1434, 1377, 1259, 1221, 1183, 1145, 1007, 749, 693, 562, 430. **ESI-HRMS**: m/z calcd. for $C_{18}H_{22}N_2O_5Na$ $[M+Na]^+$ 369.1421, found 369.1418.

N,N-Dimethyl-2-(6-methyl-2-(*p*-tolyl)indolizin-3-yl)acetamide (2av-9)

Na+

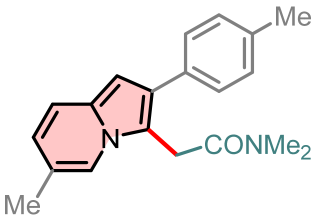

2av-9

Product **2av-9** was obtained following the general procedure **D** using 2-(6-methyl-2-(*p*-tolyl)indolizine **1av** (44.2 mg, 0.20 mmol, 1.00 eq.) and 2-((2-(dimethylamino)-2-oxoethyl)thio)-3-methyl-4,5-dihydrothiazol-3-ium triflate **T9** (110.5 mg, 0.30 mmol, 1.50 eq.). The crude was purified by column chromatography on silica (PE:EtOAc, 3:1) to get the pure product as a yellow oil: 32.6 mg, 0.11 mmol, 53%. ^{1}**H NMR** (400 MHz, $CDCl_3$) δ 7.75 (t, J = 1.2 Hz, 1H), 7.28 – 7.09 (m, 5H), 6.48 (dd, J = 9.1, 1.5 Hz, 1H), 6.39 (d, J = 0.9 Hz, 1H), 3.95 (s, 2H), 2.84 (s, 3H), 2.70 (s, 3H), 2.32 (s, 3H), 2.18 (s, 3H). ^{13}**C{^{1}H} NMR** (100 MHz, $CDCl_3$) δ 169.4, 136.2, 133.9, 131.7, 129.4, 129.1, 128.7, 120.8, 120.4, 119.8, 118.3, 113.5, 98.6, 37.5, 36.0, 31.4, 21.3, 18.9. **IR** (neat, cm^{-1}): 2922, 2856, 1640, 1395, 1260, 1135, 909, 791, 728, 585, 508. **ESI-HRMS**: m/z calcd. for $C_{20}H_{23}N_2O$ $[M+H]^+$ 307.1805, found 307.1805.

6. General Procedure for the Reaction Scale-Up

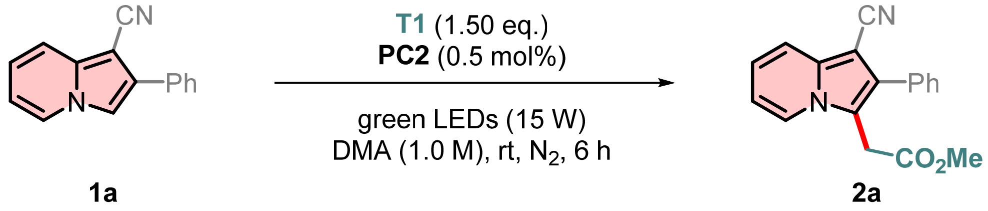

In a glovebox, to a vial filled with the 2-(phenyl)indolizine-1-carbonitrile **1a** (436.5 mg, 2.00 mmol, 1.00 eq.), radical source **T1** (1.066 g, 3.00 mmol, 1.50 eq.), **PC2** (16.0 mg, 10.0 µmol, 0.5 mol%) was added DMA (4.0 mL, 0.50 M). The vial was sealed and the mixture was irradiated with green LEDs (15 W, 525 nm) for 2 h at rt outside of the glovebox. The reaction mixture was partitioned between H_2O and Et_2O and the organic phase was extracted. The water phase was extracted with Et_2O 3 times and the combined organic phases were washed with H_2O and brine. The organic phase was dried over Na_2SO_4 and the solvent was removed under reduced pressure. The crude was purified by column

chromatography on silica (PE:EtOAc, 3:1) to get the pure product **2a** as an off-white solid: 398.1 mg, 1.37 mmol, 69%. The spectral data were in accordance with those measured.

7. General Procedure for Suzuki Coupling of 2o

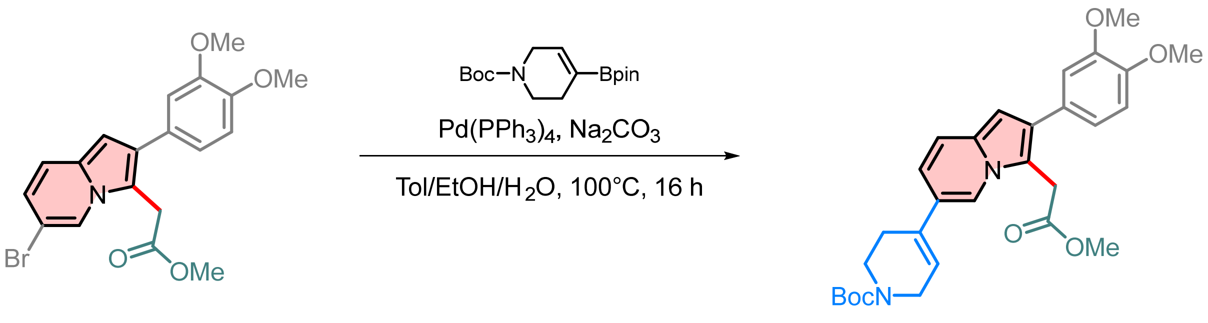

2o **3**

Under inert atmosphere, methyl 2-(6-bromo-2-(3,4-dimethoxyphenyl)indolizin-3-yl)acetate **2o** (40.4 mg, 0.10 mmol, 1.00 eq.), tert-butyl 4-(4,4,5,5-tetramethyl-1,3,2-dioxaborolan-2-yl)-3,6-dihydropyridine-1(2*H*)-carboxylate (62.4 mg, 0.12 mmol, 1.20 eq.), $Pd(PPh_3)_4$ (1.8 mg, 8.2 µmol, 5.0 mol%), Na_2CO_3 (72.5 mg, 0.68 mmol, 2.0 eq.) were suspended in Tol:EtOH:H_2O (1.4 mL, 0.07 M, 5:1:1 ratio). The resulting mixture was stirred at 105°C for 16 h. The mixture was allowed to cool down to rt, filtered through a pad of Celite (eluting with EtOAc) and the filtrate was concentrated under reduced pressure. The crude was purified by column chromatography on silica (PE:EtOAc, 2:1) to get the pure product **3** as a yellow oil: 47.6 mg, 0.09 mmol, 90%. **^{1}H NMR** (400 MHz, $CDCl_3$) δ 7.96 (s, 1H), 7.35 (dd, J = 9.4, 0.9 Hz, 1H), 7.20 (d, J = 2.0 Hz, 1H), 7.14 – 7.07 (m, 1H), 6.96 (d, J = 8.2 Hz, 1H), 6.89 (dd, J = 9.4, 1.6 Hz, 1H), 6.54 (s, 1H), 6.07 (s, 1H), 4.11 (t, J = 2.1 Hz, 2H), 3.99 (s, 2H), 3.95 (s, 3H), 3.93 (s, 3H), 3.76 (s, 3H), 3.68 (t, J = 5.8 Hz, 2H), 2.56 (d, J = 5.9 Hz, 2H), 1.51 (s, 9H). **^{13}C{^{1}H} NMR** (100 MHz, $CDCl_3$) δ 170.7, 154.9, 149.0, 148.1, 131.8, 130.1, 128.7, 124.1, 121.3, 118.6, 118.5, 115.7, 113.0, 112.6, 111.5, 99.2, 79.8, 56.0, 55.9, 52.5, 41.2, 31.6, 28.6, 26.9. **IR** (neat, cm^{-1}): 2974, 2932, 2836, 1734, 1687, 1422, 1365, 1234, 1161, 1113, 1025, 911, 797, 764, 727, 645. **ESI-HRMS**: m/z calcd. for $C_{29}H_{34}N_2O_6Na$ $[M+Na]^+$ 529.2309, found 529.2309.

8. General Procedures for Derivatization of Indolizine 2a

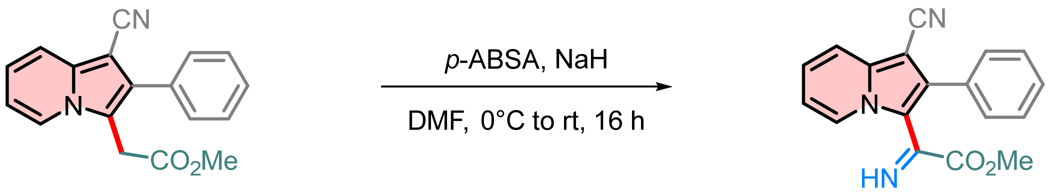

2a **4a**

To a mixture of methyl 2-(1-cyano-2-phenylindolizin-3-yl)acetate **2a** (50.0 mg, 0.17 mmol, 1.0 equiv.) and NaH (8.3 mg, 0.21 mmol, 1.2 eq.) in anhydrous DMF (1.7 mL, 0.10 M), *p*-ABSA (49.7, 0.21 mmol, 1.2 eq.) was added. The reaction mixture was stirred at room temperature for overnight. Water was added to the mixture and the aqueous layer was extracted three times with Et_2O, dried over Na_2SO_4, filtered, and concentrated in vacuo to give crude mixture. The crude was purified by column chromatography on silica (PE:EtOAc, 3:1) to get the pure product **4a** as a yellow oil: 17.4 mg, 0.05 mmol, 33% (5:1, E/Z). **^{1}H NMR** (400 MHz, $CDCl_3$) δ 10.97 (s, 1H), 9.60 (d, J = 7.1 Hz, 1H), 7.79 (d, J = 8.9 Hz, 1H), 7.51 – 7.43 (m, 5H), 7.35 (ddd, J = 8.9, 6.8, 1.1 Hz, 1H), 7.00 (td, J = 7.0, 1.4 Hz, 1H), 3.10 (s, 3H). **^{13}C{^{1}H} NMR** (100 MHz, $CDCl_3$) δ 163.8, 160.5, 140.0, 138.6, 132.7, 130.1, 129.7, 129.1, 128.8, 128.8, 128.1, 126.1, 117.6, 117.0, 115.8, 114.8, 84.6, 52.7.. **IR** (neat, cm^{-1}): 3246, 2949, 2923, 2852, 2206, 1736, 1632, 1585, 1507, 1428, 1366, 1317, 1221, 1082, 981, 913, 876, 786, 743, 697, 623, 585, 492, 454, 419. **ESI-HRMS**: m/z calcd. for $C_{18}H_{13}N_3O_2Na$ $[M+Na]^+$ 326.0900, found 326.0900.

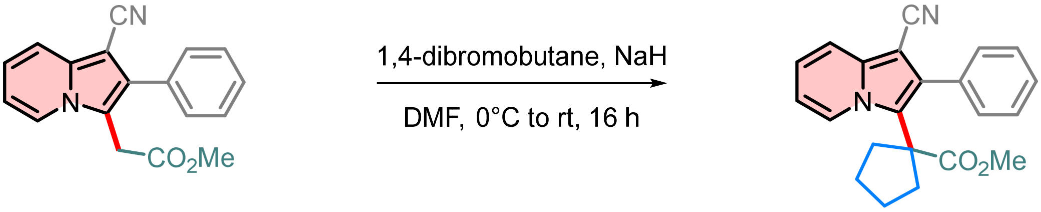

2a **4b**

To a mixture of methyl 2-(1-cyano-2-phenylindolizin-3-yl)acetate **2a** (50.0 mg, 0.17 mmol, 1.0 equiv.) NaH (15.2 mg, 0.38 mmol, 2.2 eq.) is added. After 1 h of stirring at rt, 1,4-dibromobutane (24 µL, 0.21 mmol, 1.2 equiv.) is added. The reaction mixture was stirred at room temperature for overnight. Water was added to the mixture and the aqueous layer was extracted three times with Et_2O, dried over Na_2SO_4, filtered, and concentrated in vacuo to give crude mixture. The crude was purified by column chromatography on silica (PE:EtOAc, 3:1) to get the pure product **4b** as a colorless oil: 43.0 mg, 0.12 mmol, 73%. **^{1}H NMR** (400 MHz, $CDCl_3$) δ 8.08 (dt, J = 7.4, 1.1 Hz, 1H), 7.64 (dt, J = 8.9, 1.3 Hz, 1H), 7.49 – 7.39 (m, 5H), 7.09 (ddd, J = 8.9, 6.7, 0.9 Hz, 1H), 6.78 (td, J = 7.0, 1.4 Hz, 1H), 3.70 (s, 3H),

2.59 – 2.46 (m, 2H), 1.88 – 1.79 (m, 2H), 1.65 – 1.47 (m, 4H). **^{13}C{^{1}H} NMR** (100 MHz, $CDCl_3$) δ 175.6, 137.5, 134.2, 131.1, 130.4, 129.1, 128.2, 128.2, 128.0, 127.6, 125.5, 124.0, 122.2, 117.9, 116.5, 113.1, 85.2, 54.7, 53.1, 36.3, 24.5. **IR** (neat, cm^{-1}): 2953, 2925, 2873, 2853, 2206, 1709, 1512, 1431, 1329, 1235, 1171, 1070, 1004, 913, 748, 696, 495. **ESI-HRMS**: m/z calcd. for $C_{22}H_{20}N_2O_2Na$ $[M+Na]^+$ 367.1417, found 367.1417.

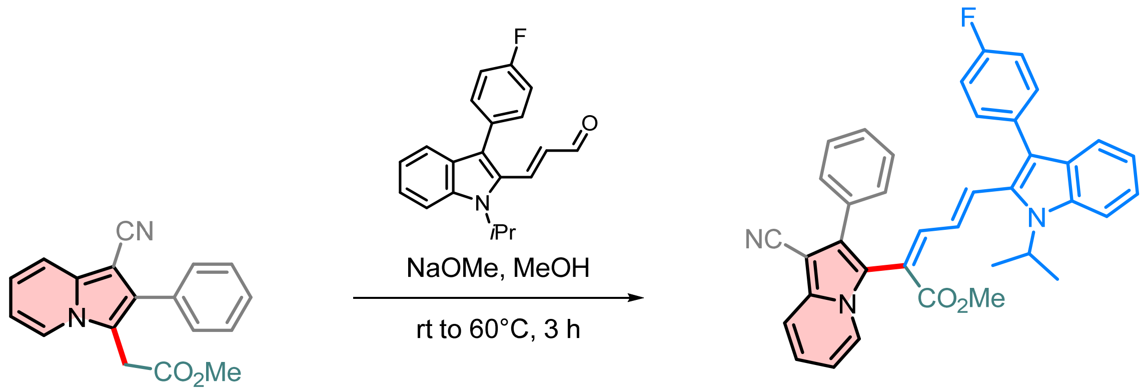

2a **4c**

To a solution of methyl 2-(1-cyano-2-phenylindolizin-3-yl)acetate **2a** (50.0 mg, 0.17 mmol, 1.0 eq.) in MeOH (1.0 mL, 0.17 M), NaOMe (14.0 mg, 0.26 mmol, 1.5 eq.) and (*E*)-3-(3-(4-fluorophenyl)-1-isopropyl-1*H*-indol-2-yl)acrylaldehyde (58.2 mg, 0.19 mmol, 1.1 eq.) are added sequentially at rt. The reaction mixture is warmed up for 3 h at 60°C. Water was added to the mixture and the aqueous layer was extracted three times with Et_2O, dried over Na_2SO_4, filtered, and concentrated in vacuo to give crude mixture. The crude was purified by column chromatography on silica (PE:EtOAc, 7:1) to get the pure product **4c** as an orange oil: 28.5 mg, 0.05 mmol, 30%. The product can be further purified by recrystallization in EtOH. **^{1}H NMR** (400 MHz, $CDCl_3$) δ 7.89 – 7.69 (m, 2H), 7.61 (dt, J = 7.0, 1.1 Hz, 1H), 7.47 (d, J = 8.5 Hz, 1H), 7.45 – 7.38 (m, 2H), 7.39 – 7.31 (m, 3H), 7.32 – 7.25 (m, 1H), 7.25 – 7.15 (m, 2H), 7.15 – 6.96 (m, 4H), 6.96 – 6.81 (m, 2H), 6.78 (td, J = 6.8, 1.3 Hz, 1H), 5.91 (dd, J = 15.7, 11.7 Hz, 1H), 4.65 (p, J = 7.0 Hz, 1H), 3.67 (s, 3H), 1.63 – 1.46 (m, 6H). **^{13}C{^{1}H} NMR** (100 MHz, $CDCl_3$) δ 166.8, 163.1, 160.6, 147.8, 138.6, 136.7, 132.3, 132.0, 131.9, 131.9, 131.7, 131.6, 130.5, 130.5, 128.9, 128.8, 128.7, 127.9, 126.3, 124.1, 123.8, 122.8, 120.4, 120.1, 119.3, 118.2, 116.8, 116.6, 115.6, 115.4, 113.3, 112.0, 82.0, 52.5, 47.8, 21.9, 21.8. **^{19}F{^{1}H} NMR** (377 MHz, $CDCl_3$) δ -113.90 (s, 1F). **IR** (neat, cm^{-1}): 3058, 3029, 2924, 2206, 1697, 1598, 1510, 1440, 1340, 1212, 1152, 1077 978, 835, 744, 701, 566, 491. **ESI-HRMS**: m/z calcd. for $C_{38}H_{30}FN_3O_2Na$ $[M+Na]^+$ 602.2214, found 602.2211.

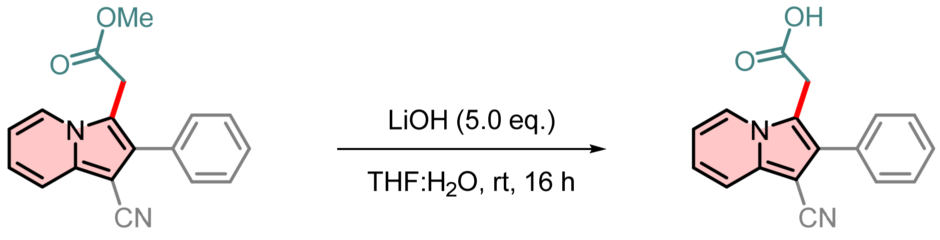

2a **4d**

Methyl 2-(1-cyano-2-phenylindolizin-3-yl)acetate **2a** (290.3 mg, 1.0 mmol, 1.0 eq.) was dissolved in THF:H_2O (10 mL, 0.1 M, 2:1 ratio), LiOH (210.0 mg, 5.0 mmol, 5.0 eq.) added, and the resulting solution stirred until completion. The reaction was quenched with 1 M HCl, extracted with EtOAc, and the organic extracts washed with brine, dried over MgSO4, filtered, and then concentrated in vacuo to give the hydrolysed product **4d** as a brown wax: 277.0 mg, 1.0 mmol, quant.. ^{1}**H NMR** (400 MHz, DMSO-*d*^{6}) δ 12.87 (bs, 1H), 8.41 (dt, J = 7.1, 1.1 Hz, 1H), 7.71 (dt, J = 8.9, 1.2 Hz, 1H), 7.63 – 7.53 (m, 2H), 7.53 – 7.42 (m, 3H), 7.28 (ddd, J = 8.9, 6.7, 1.0 Hz, 1H), 7.03 (td, J = 6.9, 1.3 Hz, 1H), 4.00 (s, 2H). ^{13}**C{**^{1}**H} NMR** (100 MHz, DMSO-*d*^{6}) δ 170.8, 137.2, 132.0, 129.7, 129.2, 129.0, 128.1, 125.5, 123.5, 117.3, 116.7, 116.5, 113.3, 79.9, 30.2. **IR** (neat, cm^{-1}): 3003, 2215, 1715, 1509, 1390, 1264, 1189, 851, 743, 706,647, 615, 495, 455. **ESI-HRMS**: m/z calcd. for $C_{17}H_{12}N_2O_2Na$ $[M+Na]^+$ 299.0791, found 299.0791.

9. General Procedure for Amidation/Esterification of 4d

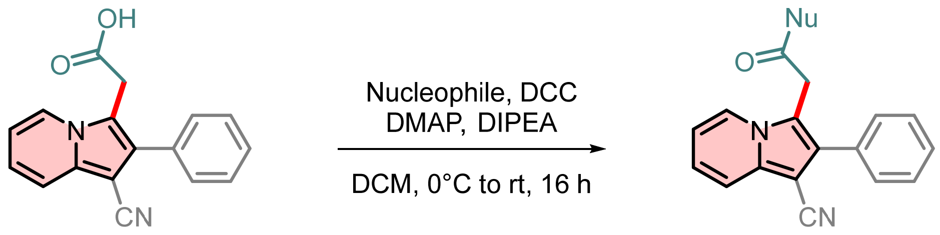

4d **5**

To a solution of 2-(1-cyano-2-phenylindolizin-3-yl)acetic acid **4d** (50.0 mg, 0.18 mmol, 1.0 eq.) in DCM (1.0 mL, 0.18 M), was added the respective amine or alcohol (0.22 mmol, 1.2 eq.) and DCC (41.1 mg, 0.20 mmol, 1.1 eq.). The solution was cooled to 0 °C, and DMAP (2.7 mg, 0.02 mmol, 0.12 eq.) and DIPEA (38 µL, 0.22 mmol, 1.2 eq.) were added. The mixture was stirred at 0°C for 0.5 h then was allowed to warm up to room temperature and further stirred for 4 h. The resulting suspension was filtered and the filtrate was washed successively with 1 N HCl, saturated aqueous $NaHCO_3$ and water, dried over Na_2SO_4 and concentrated under reduced pressure.

2-(1-Cyano-2-phenylindolizin-3-yl)-*N*-methoxy-*N*-methylacetamide (5a)

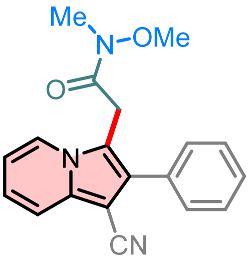

5a

Product **5a** was obtained following the general procedure using *N*,*O*-dimethylhydroxylammonium chloride (21.2 mg, 0.22 mmol, 1.2 eq.). The crude was purified by column chromatography on silica (PE:EtOAc, 1:2) to get the pure product as a white solid: 54.0 mg, 0.17 mmol, 93%. **^{1}H NMR** (400 MHz, $CDCl_3$) δ 8.09 (d, J = 7.1 Hz, 1H), 7.67 (d, J = 8.9 Hz, 1H), 7.63 – 7.29 (m, 5H), 7.11 (dd, J = 9.0, 6.6 Hz, 1H), 6.82 (t, J = 6.9 Hz, 1H), 4.10 (s, 2H), 3.48 (s, 3H), 3.20 (s, 3H). **^{13}C{^{1}H} NMR** (100 MHz, $CDCl_3$) δ 169.5, 138.2, 132.6, 131.6, 129.7, 128.9, 128.1, 124.5, 122.5, 117.8, 117.0, 116.3, 113.3, 81.8, 61.3, 32.4. **IR** (neat, cm^{-1}): 3323, 2927, 2849, 2204, 1624, 1568, 1383, 1309, 1269, 1242, 1086, 995, 891 771, 752, 710, 640, 497, 416. **ESI-HRMS**: m/z calcd. for $C_{19}H_{17}N_3O_2Na$ $[M+Na]^+$ 342.1213, found 342.1213.

***N*-(1-(Benzo[*d*][1,3]dioxol-5-yl)propan-2-yl)-2-(1-cyano-2-phenylindolizin-3-yl)acetamide (5b)**

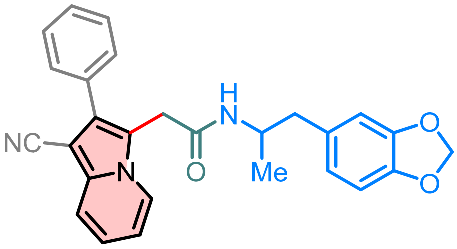

5b

Product **5b** was obtained following the general procedure using 1-(benzo[*d*][1,3]dioxol-5-yl)propan-2-amine hydrochloride (42.9 mg, 0.20 mmol, 1.1 eq.). The crude was purified by column chromatography on silica (PE:EtOAc, 3:1 to 1:1) to get the pure product as an off-white solid: 50.4 mg, 0.11 mmol, 63%. **^{1}H NMR** (400 MHz, $CDCl_3$) δ 7.90 (dd, J = 7.0, 1.2 Hz, 1H), 7.68 (dt, J = 9.0, 1.2 Hz, 1H), 7.53 – 7.33 (m, 5H), 7.15 (ddd, J = 8.9, 6.7, 1.0 Hz, 1H), 6.83 (td, J = 6.8, 1.3 Hz, 1H), 6.57 (d, J = 7.8 Hz, 1H), 6.40 (d, J = 1.7 Hz, 1H), 6.33 (dd, J = 7.9, 1.7 Hz, 1H), 5.92 (q, J = 1.5 Hz, 2H), 5.31 (d, J = 8.0 Hz, 1H), 4.27 – 4.07 (m, 1H), 3.85 – 3.66 (m, 2H), 2.69 – 2.40 (m, 2H), 1.08 (d, J = 6.6 Hz, 3H). **^{13}C{^{1}H} NMR** (100 MHz, $CDCl_3$) δ 167.4, 147.8, 146.3, 138.4, 131.9, 131.8, 131.0, 129.4, 129.2, 128.5, 123.9, 123.0, 121.9, 118.0, 116.6, 116.0, 113.7, 109.3, 108.3, 101.1, 82.1, 46.6, 41.9, 33.1, 20.4. **IR** (neat, cm$^-$

[1]): 3278, 2971, 2926, 2851,2208, 1644, 1544, 1489, 1441, 1244, 1038, 926, 739, 702, 491. **ESI-HRMS**: m/z calcd. for $C_{27}H_{23}N_3O_3Na$ $[M+Na]^+$ 460.1637, found 460.1632.

(2*S*,5*R*)-2-Isopropyl-5-methylcyclohexyl 2-(1-cyano-2-phenylindolizin-3-yl)acetate (5c)

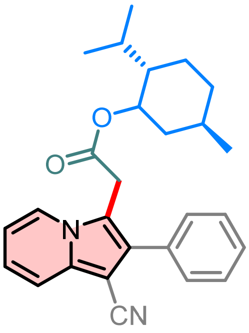

5c

Product **5c** was obtained following the general procedure using (2*S*,5*R*)-menthol (34.0 mg, 0.22 mmol, 1.2 eq.). The crude was purified by column chromatography on silica (PE:EtOAc, 6:1) to get the pure product as a yellow oil: 78.1 mg, 0.18 mmol, quant.. **^{1}H NMR** (400 MHz, $CDCl_3$) δ 8.04 (dd, J = 7.0, 1.3 Hz, 1H), 7.70 (dd, J = 8.9, 1.3 Hz, 1H), 7.64 – 7.55 (m, 2H), 7.54 – 7.45 (m, 2H), 7.46 – 7.39 (m, 1H), 7.18 – 7.10 (m, 1H), 6.86 (td, J = 6.9, 1.3 Hz, 1H), 4.73 (td, J = 10.9, 4.4 Hz, 1H), 4.00 – 3.81 (m, 2H), 2.07 – 1.95 (m, 1H), 1.73 – 1.55 (m, 3H), 1.55 – 1.40 (m, 1H), 1.33 (tt, J = 10.9, 3.2 Hz, 1H), 1.08 (s, 2H), 0.91 (d, J = 6.5 Hz, 4H), 0.81 (d, J = 7.0 Hz, 3H), 0.67 (d, J = 7.0 Hz, 3H). **^{13}C{^{1}H} NMR** (100 MHz, $CDCl_3$) δ 169.0, 138.2, 132.2, 131.7, 129.7, 128.9, 128.2, 123.9, 122.5, 117.9, 116.8, 115.8, 113.3, 81.9, 47.1, 40.8, 34.1, 31.5, 31.4, 26.3, 23.3, 22.0, 20.8, 16.2. **IR** (neat, cm^{-1}): 2954, 2917, 2869, 2847, 2205, 1718, 1513, 1449, 1314, 1207, 1166, 960, 800, 743, 696, 490, 450. **ESI-HRMS**: m/z calcd. for $C_{27}H_{30}N_2O_2Na$ $[M+Na]^+$ 437.2199, found 437.2199.

3-Methylthiazolidine-2-thione

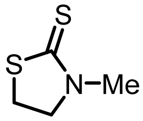

3-Methylthiazolidine-2-thione is always obtained as byproduct of the reactions as an off-white solid. **^{1}H NMR** (400 MHz, $CDCl_3$) δ 4.10 (dd, J = 8.6, 7.4 Hz, 2H), 3.45 – 3.10 (m, 5H). **^{13}C{^{1}H} NMR** (100 MHz, $CDCl_3$) δ 196.7, 58.9, 36.7, 27.0. **IR** (neat, cm^{-1}): 2921, 2861, 1666, 1502, 1391, 1308, 1226, 1103, 1022, 981, 906, 594, 540, 456. **ESI-HRMS**: m/z calcd. for $C_4H_7NS_2Na$ $[M+Na]^+$ 155.9912, found 155.9912.

10. Absorption and Electrochemical Properties

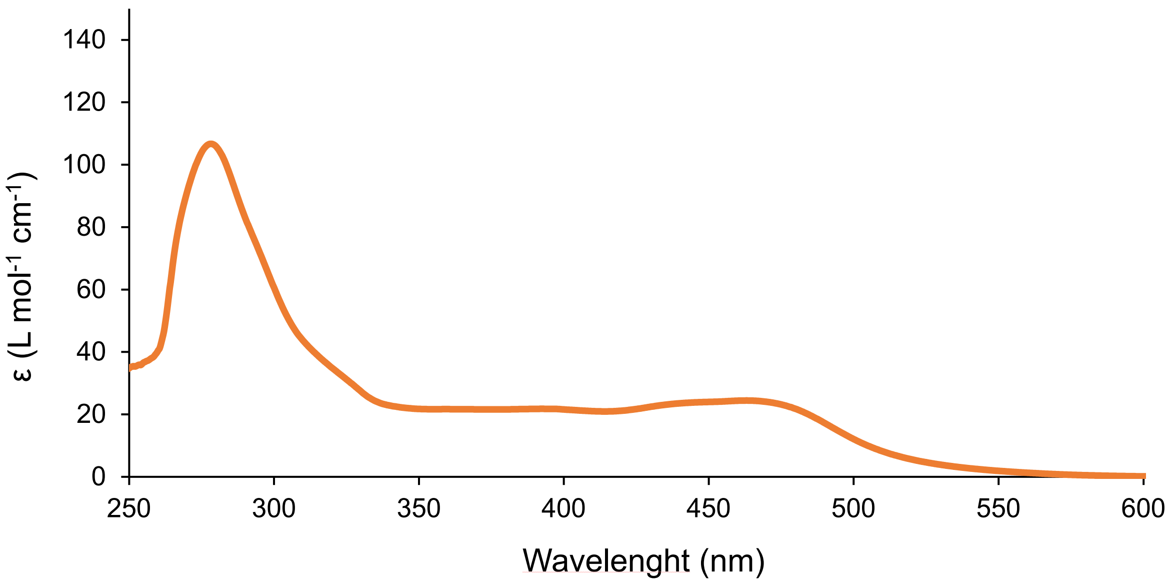

Figure S3. UV-vis spectrum of **PC2** (5 μM) in MeCN.

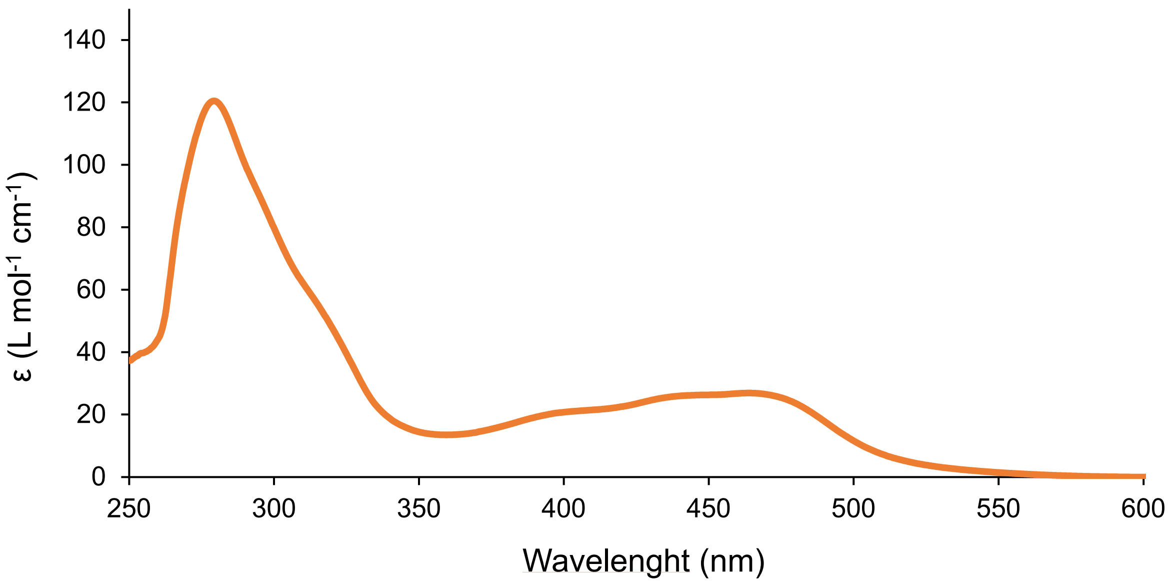

Figure S4. UV-vis spectrum of **PC3** (5 μM) in MeCN.

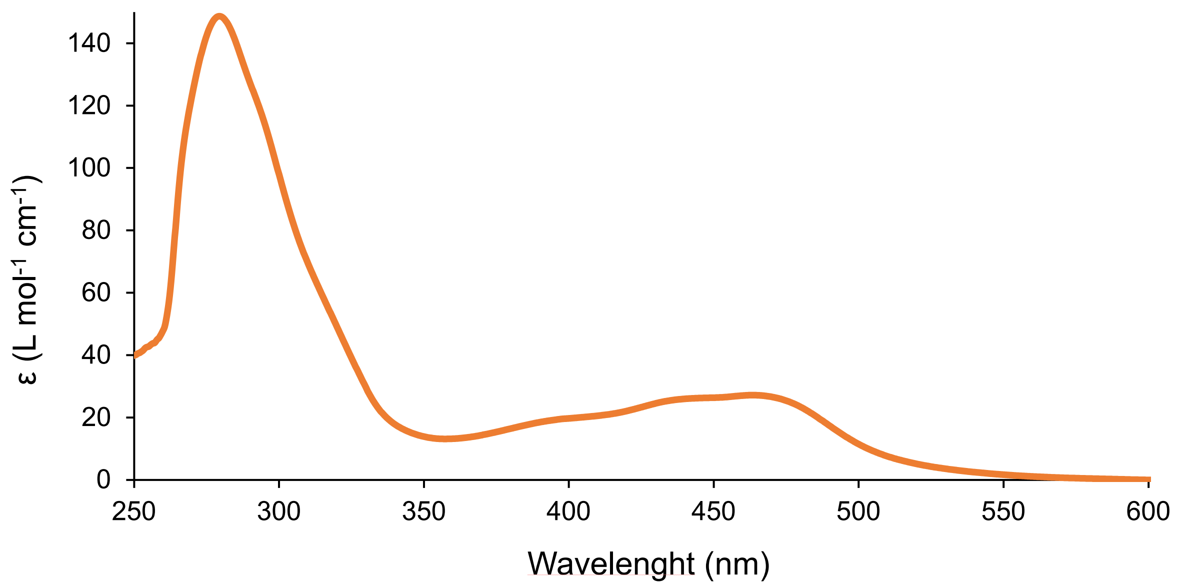

Figure S5. UV-vis spectrum of **PC4** (5 µM) in MeCN.

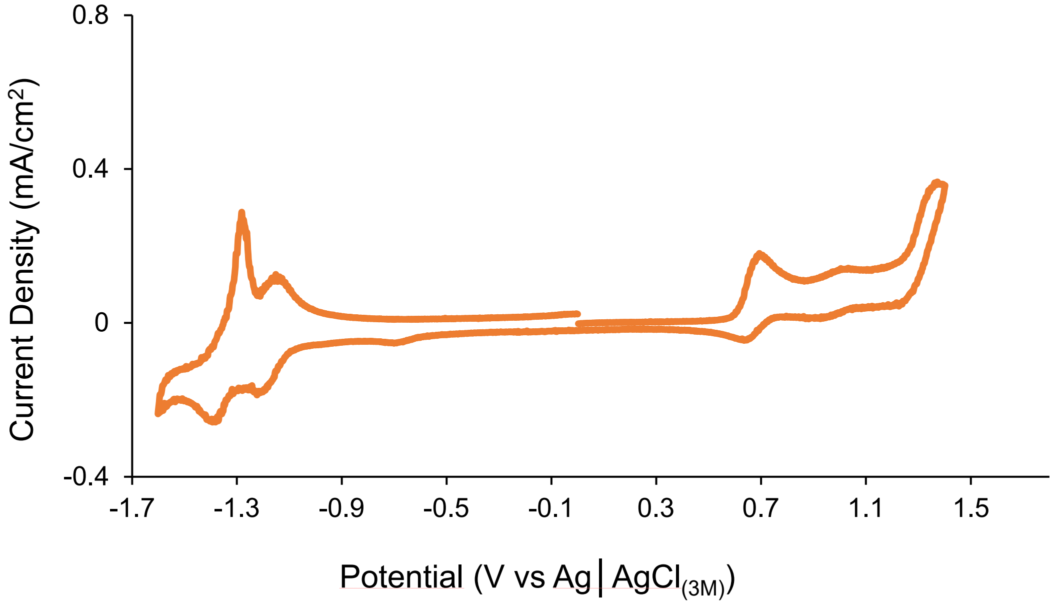

Figure S6. Cyclic voltammogram of **PC2** (2 mM) in a supporting electrolyte 0.1 M [Bu_4N][PF_6] in MeCN referenced to Ag/Ag^+ couple at 0.1 Vs^{-1} sweep rate.

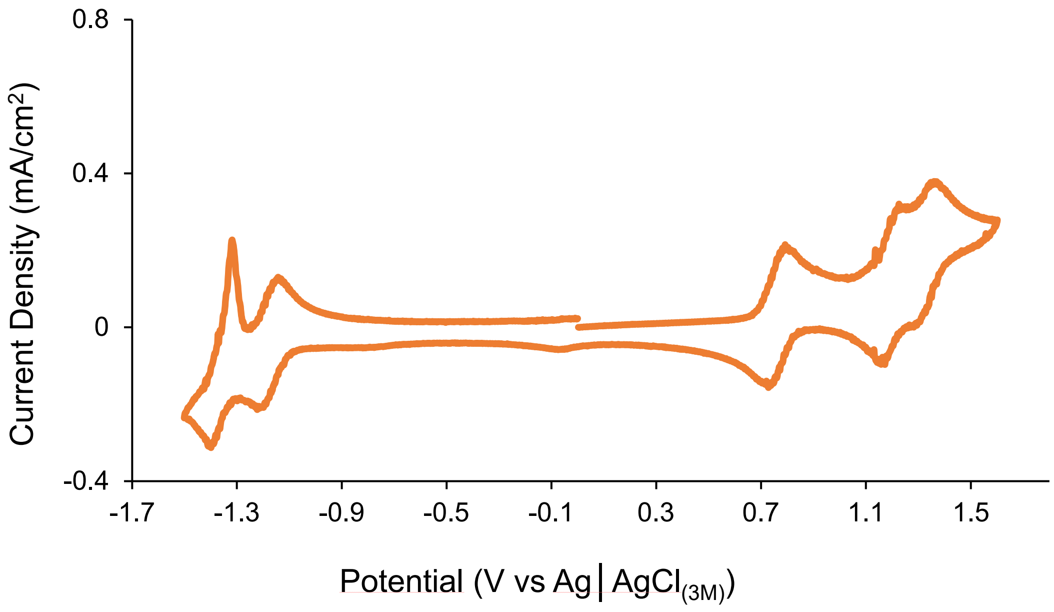

Figure S7. Cyclic voltammogram of **PC3** (2 mM) in a supporting electrolyte 0.1 M $[Bu_4N][PF_6]$ in MeCN referenced to Ag/Ag^+ couple at 0.1 Vs^{-1} sweep rate.

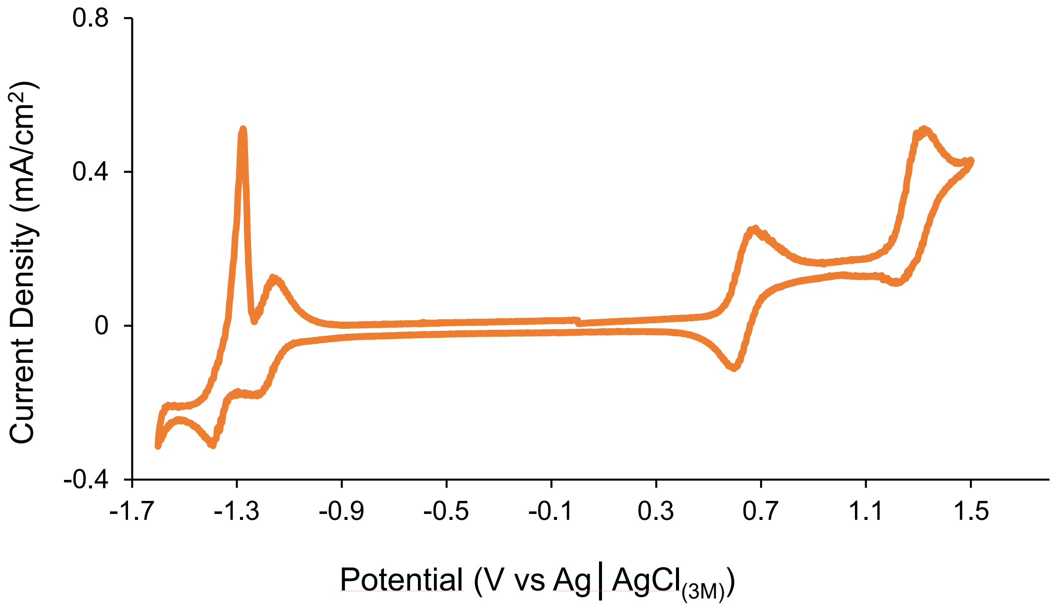

Figure S8. Cyclic voltammogram of **PC4** (2 mM) in a supporting electrolyte 0.1 M $[Bu_4N][PF_6]$ in MeCN referenced to Ag/Ag^+ couple at 0.1 Vs^{-1} sweep rate.

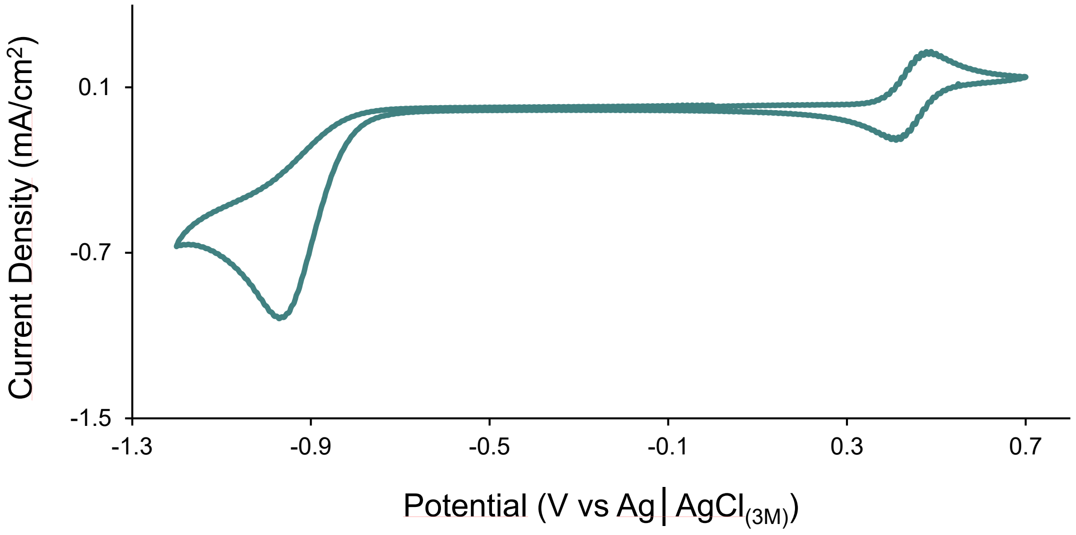

Figure S9. Cyclic voltammogram of **Radical Source T1** (2 mM) in a supporting electrolyte 0.1 M $[Bu_4N][PF_6]$ in MeCN referenced to Ag/Ag^+ couple at 0.1 Vs^{-1} sweep rate. Ferrocene/Ferrocenium oxidation is showed at + 0.42 V.

11. NMR Spectra

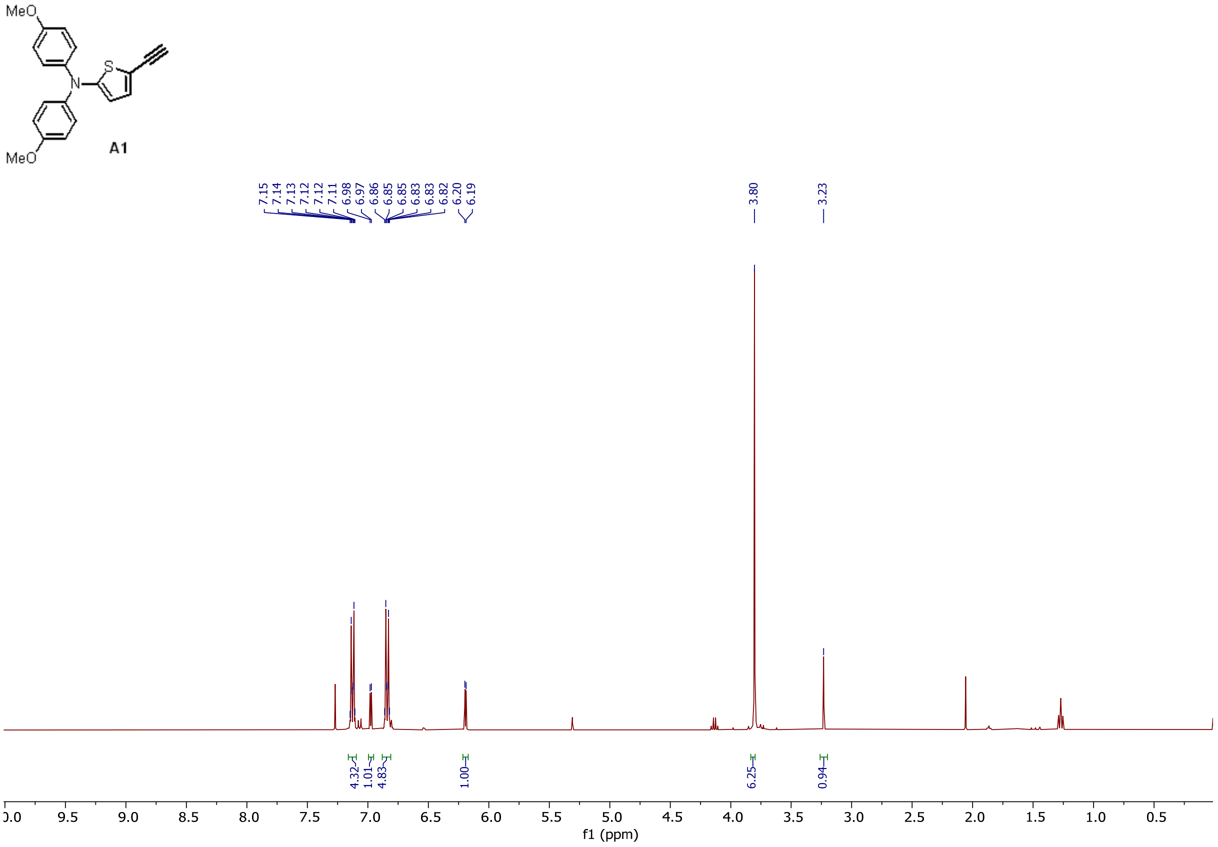

^1H NMR (400 MHz, $CDCl_3$) of **A1**.

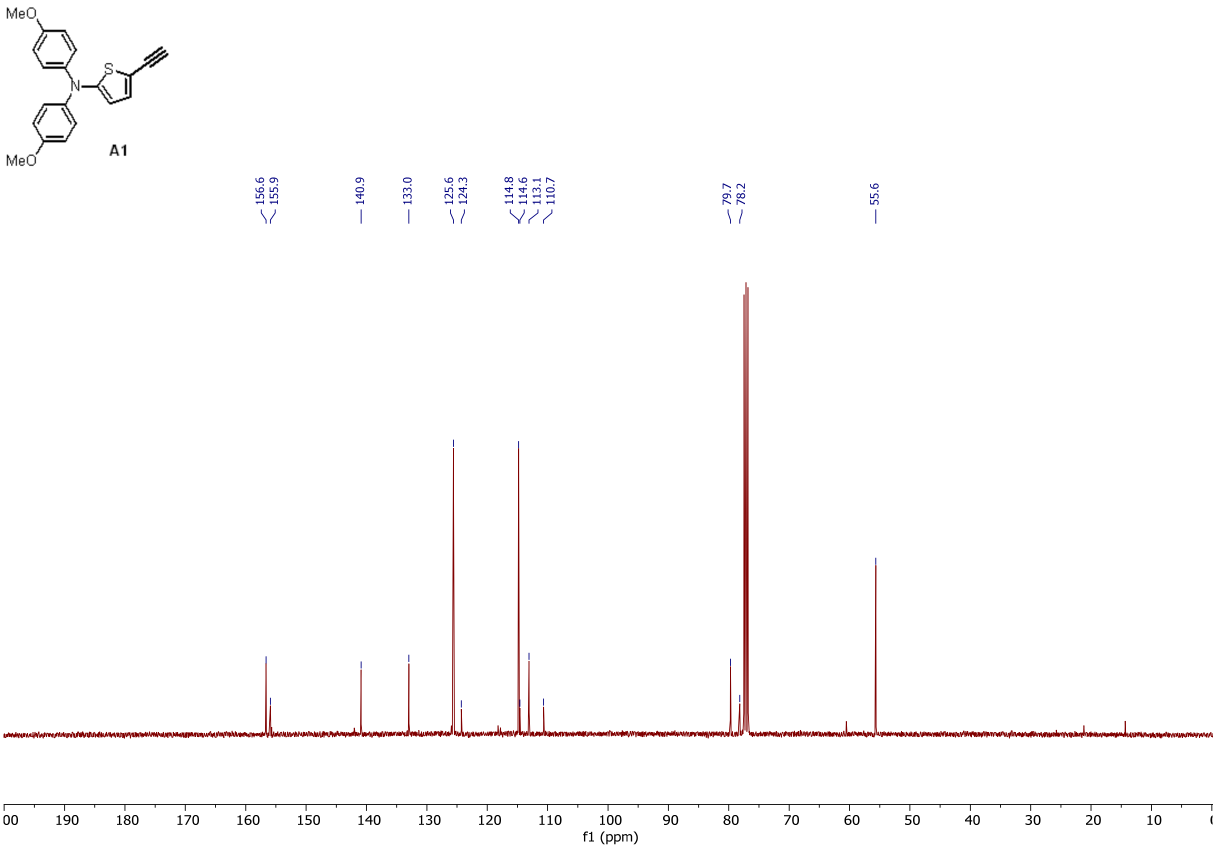

$^{13}C\{^{1}H\}$ NMR (100 MHz, $CDCl_3$) of **A1**.

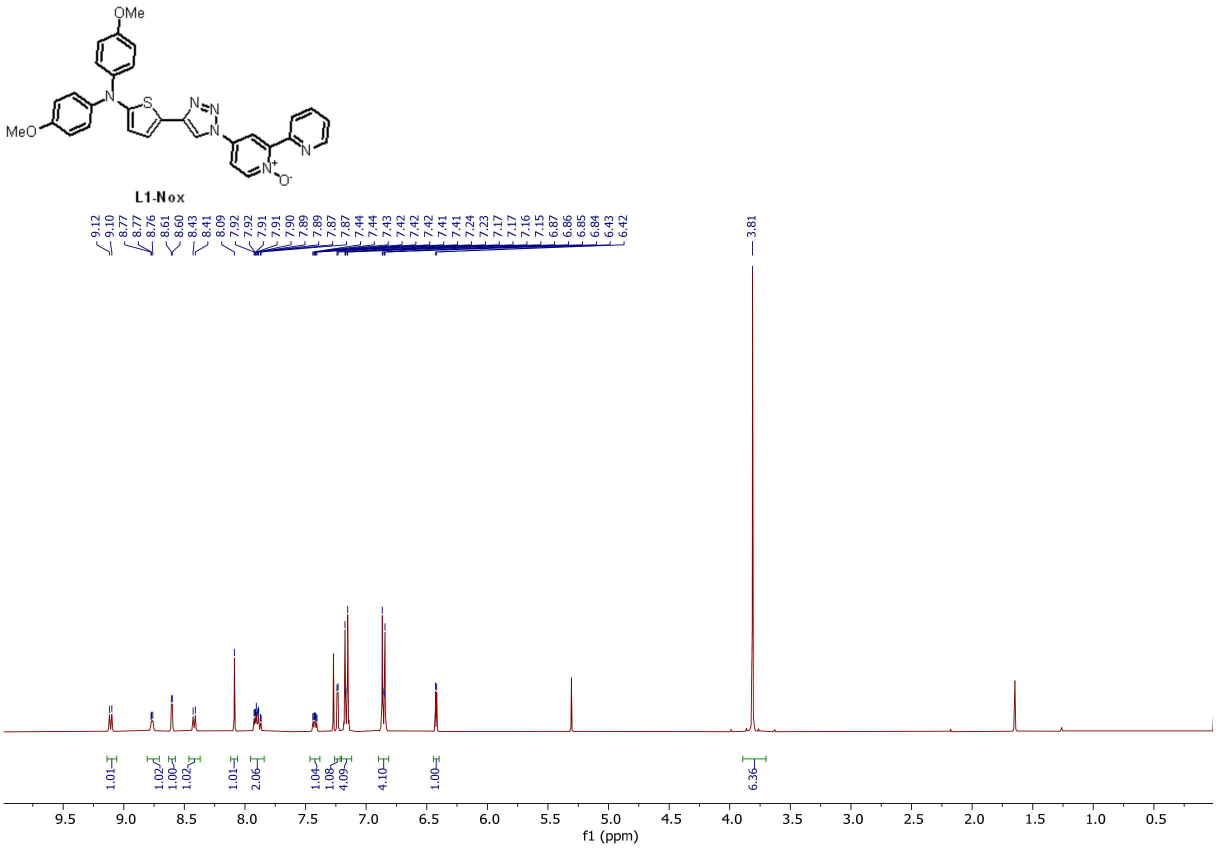

^{1}H NMR (400 MHz, $CDCl_3$) of **L1-Nox**.

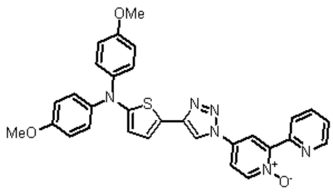

L1-Nox

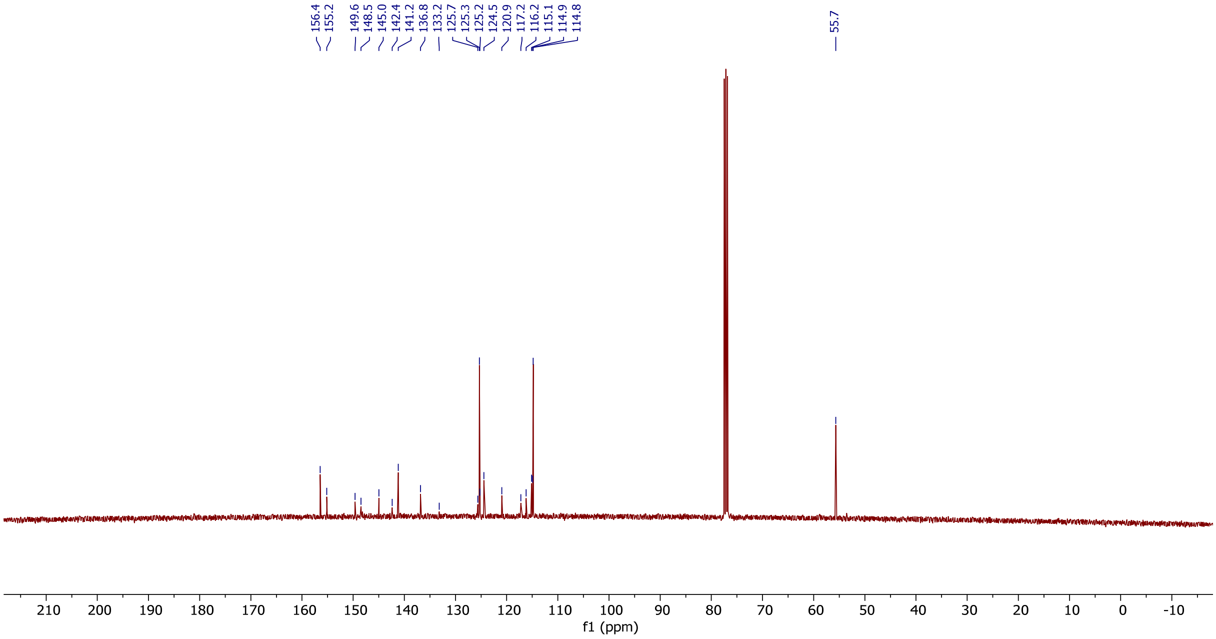

$^{13}C\{^{1}H\}$ NMR (100 MHz, $CDCl_3$) of **L1-Nox**.

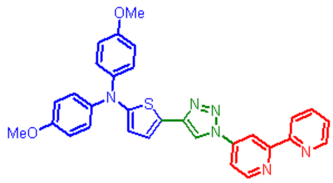

L1

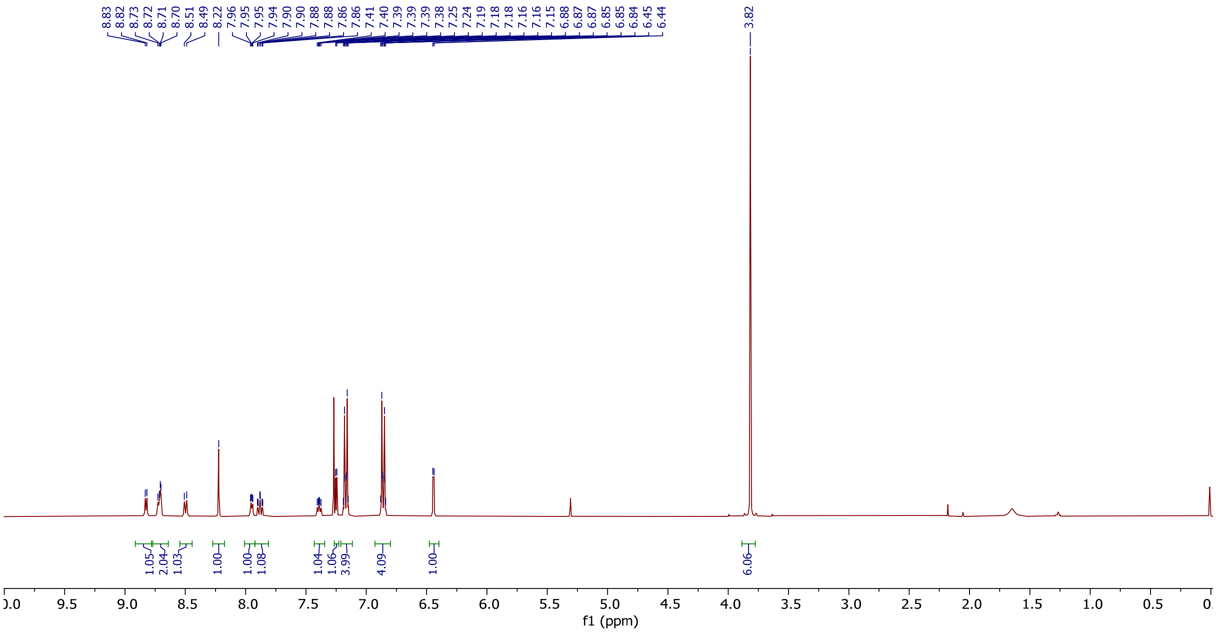

^{1}H NMR (400 MHz, $CDCl_3$) of **L1**.

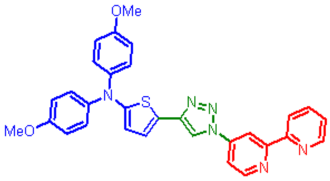

L1

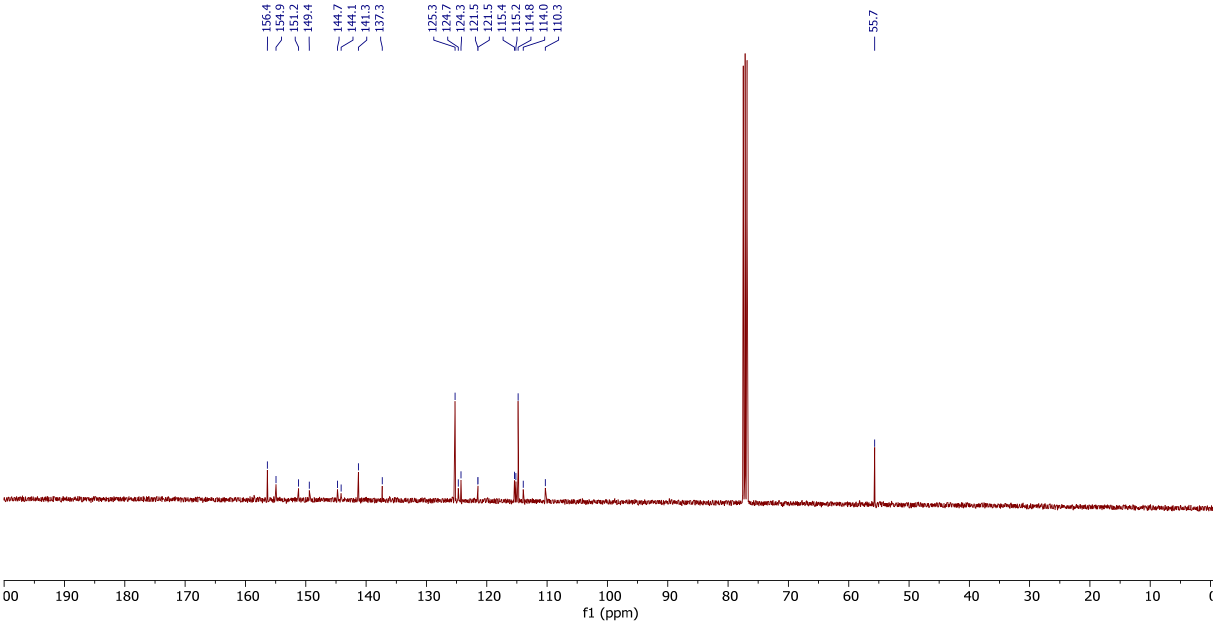

$^{13}C\{^{1}H\}$ NMR (100 MHz, $CDCl_3$) of **L1.**

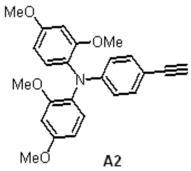

A2

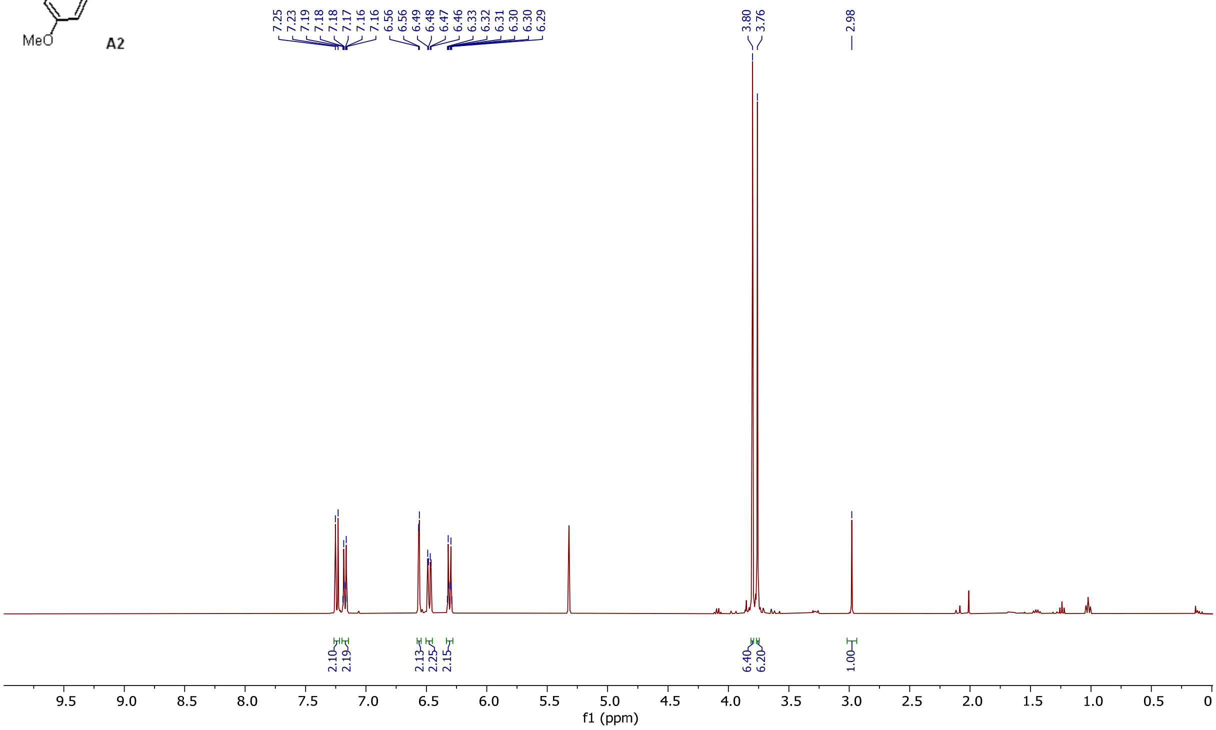

^{1}H NMR (400 MHz, CD_2Cl_2) of **A2**.

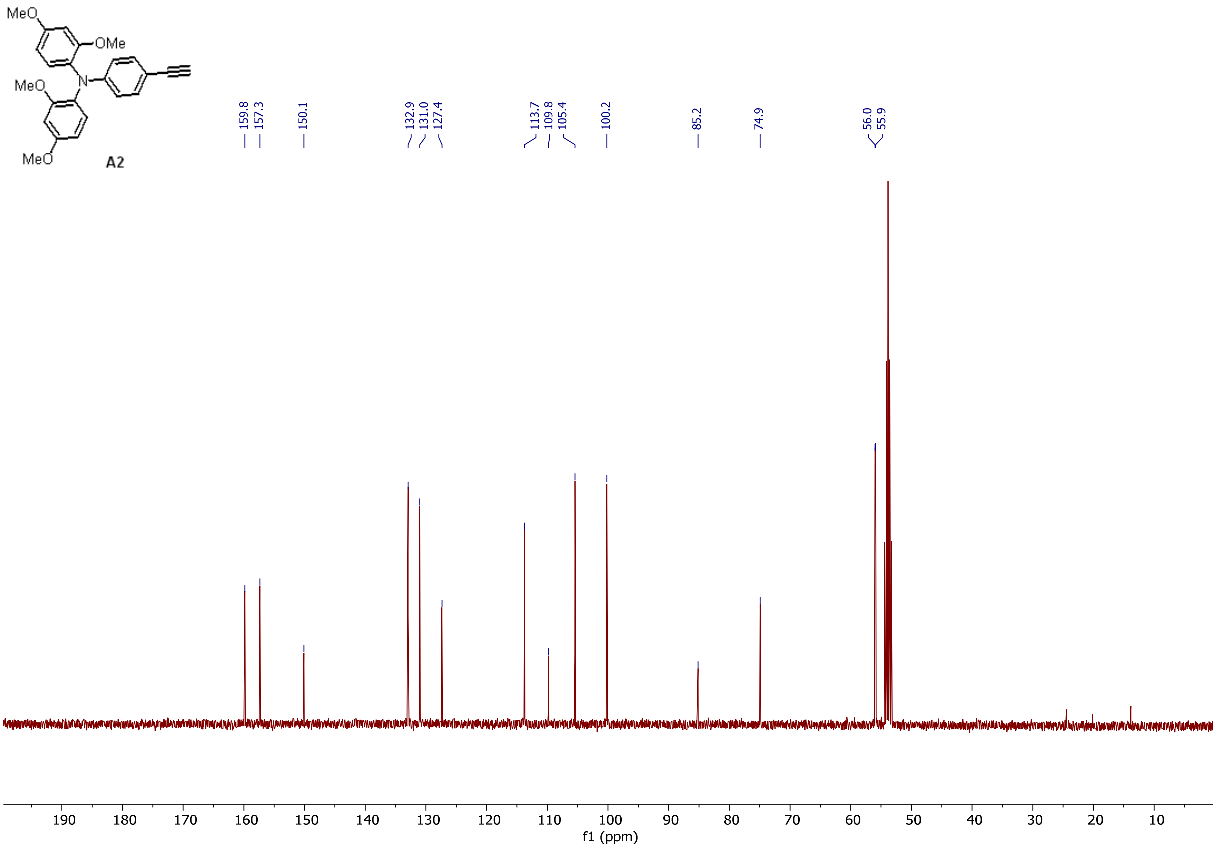

$^{13}C\{^{1}H\}$ NMR (100 MHz, CD_2Cl_2) of **A2**.

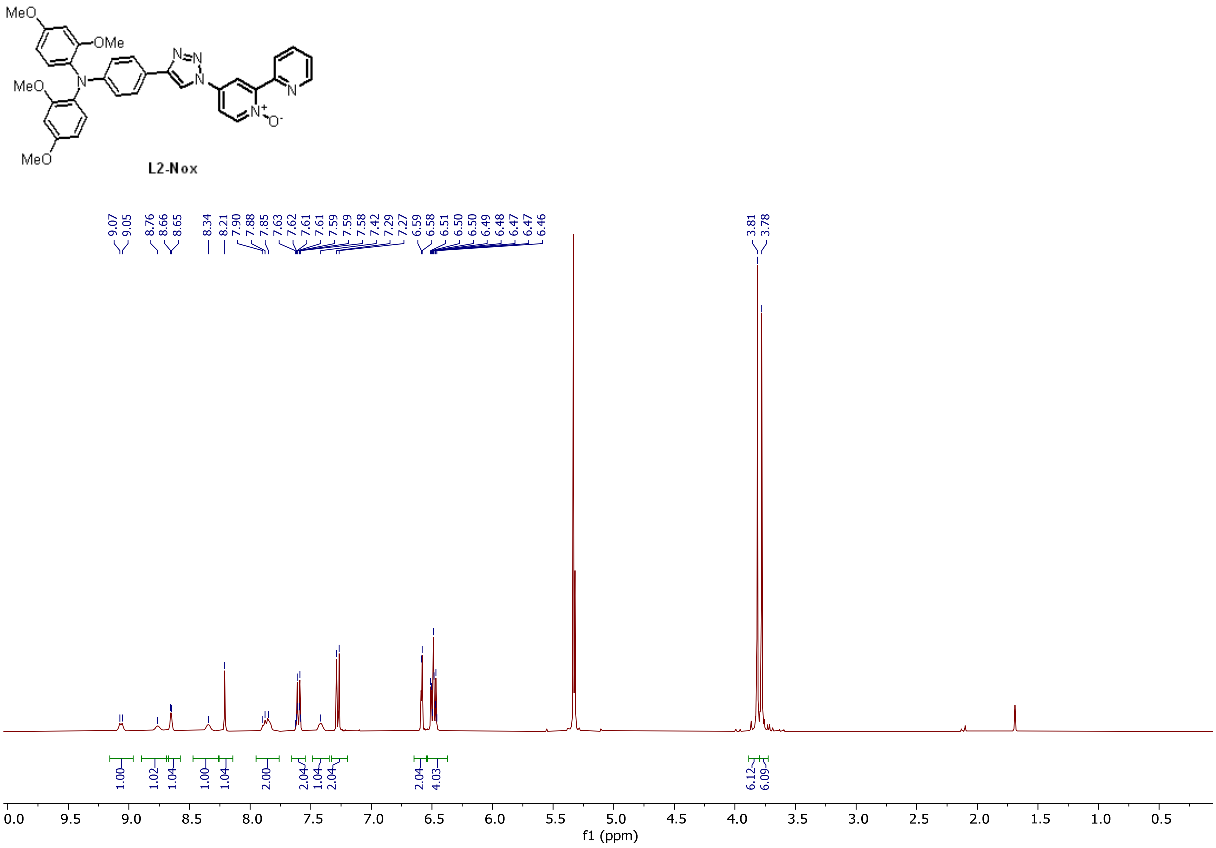

^{1}H NMR (400 MHz, CD_2Cl_2) of **L2-Nox**.

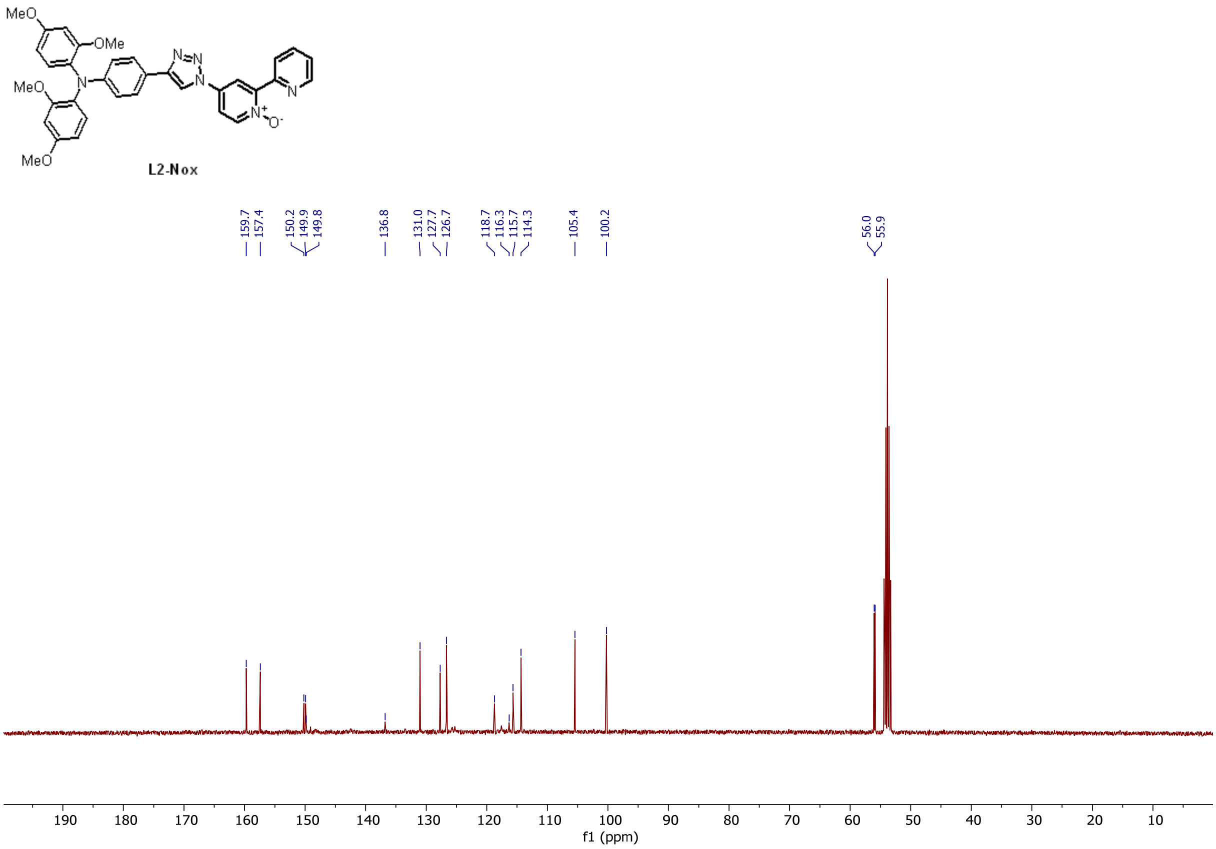

$^{13}C\{^{1}H\}$ NMR (100 MHz, CD_2Cl_2) of **L2-Nox**.

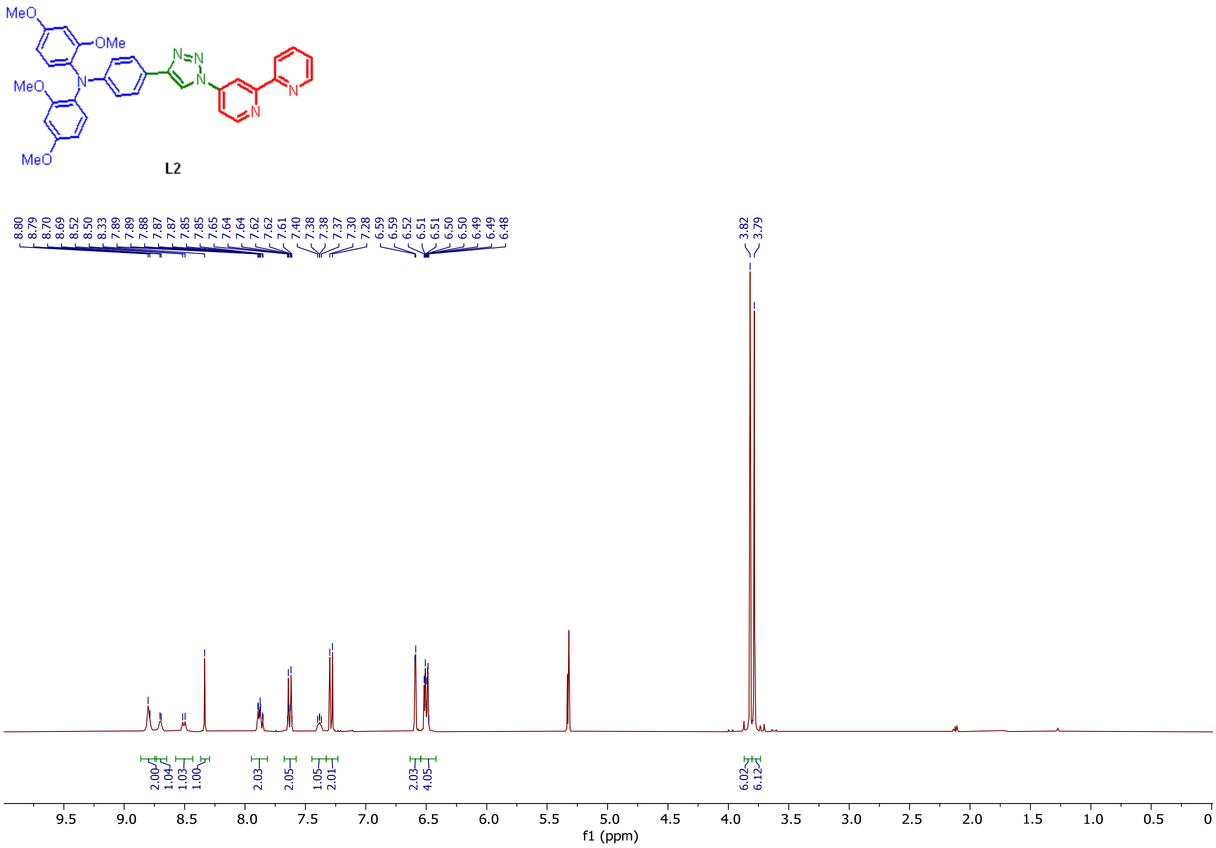

^{1}H NMR (400 MHz, CD_2Cl_2) of **L2**.

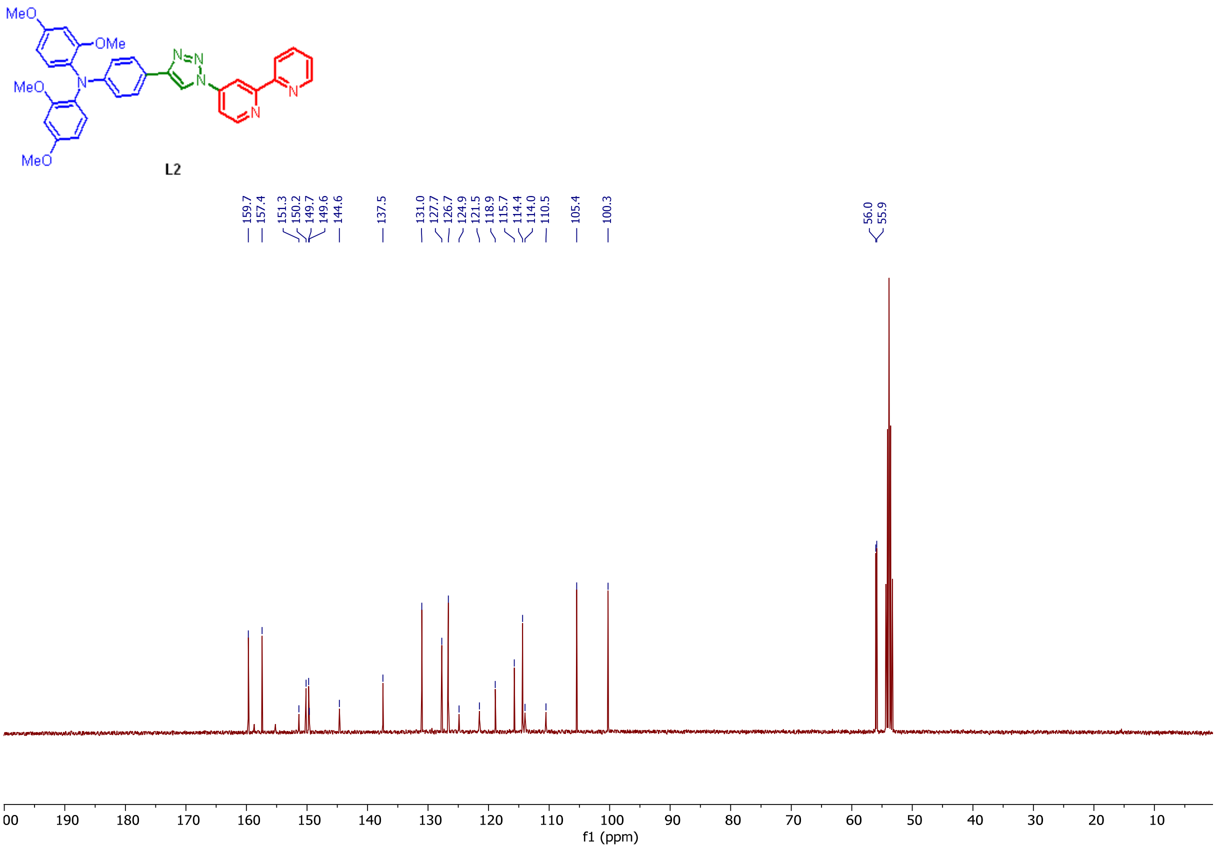

$^{13}C\{^{1}H\}$ NMR (100 MHz, CD_2Cl_2) of **L2**.

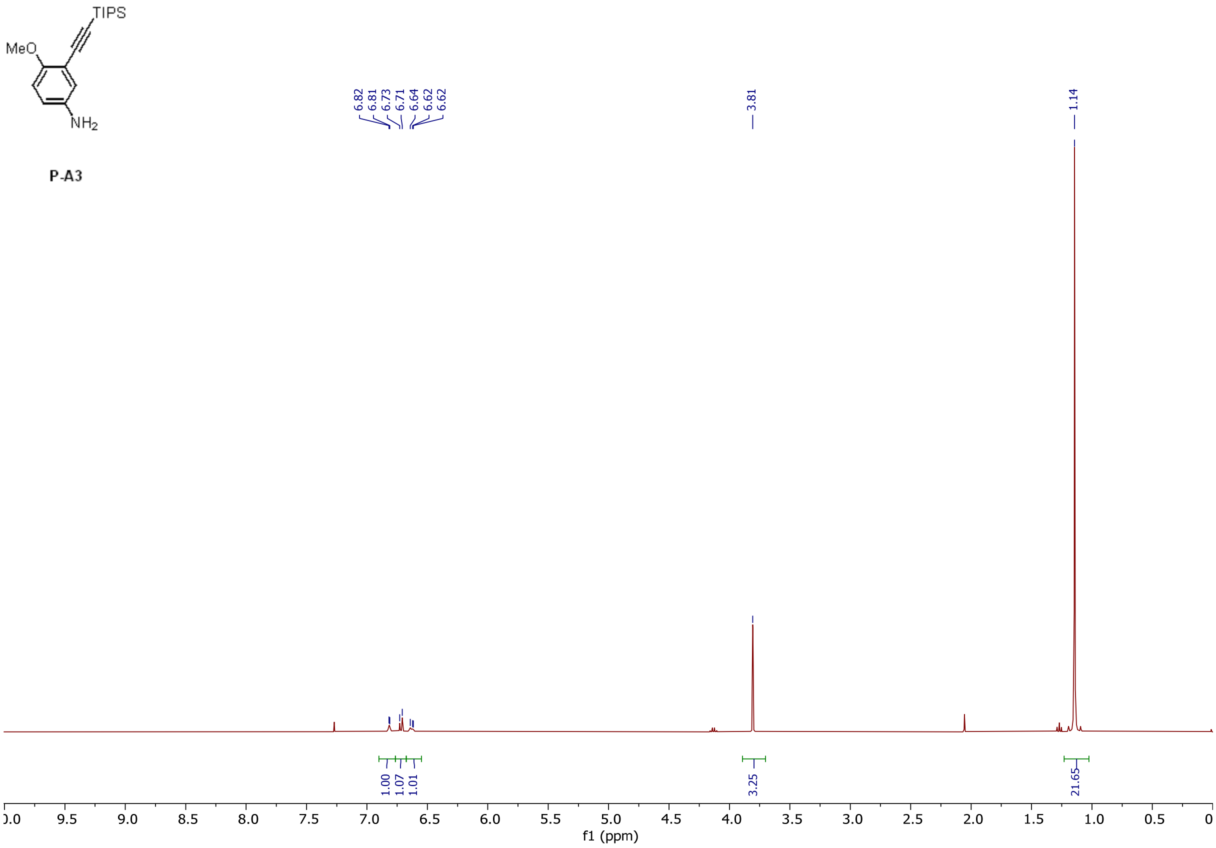

^{1}H NMR (400 MHz, $CDCl_3$) of **P-A3**.

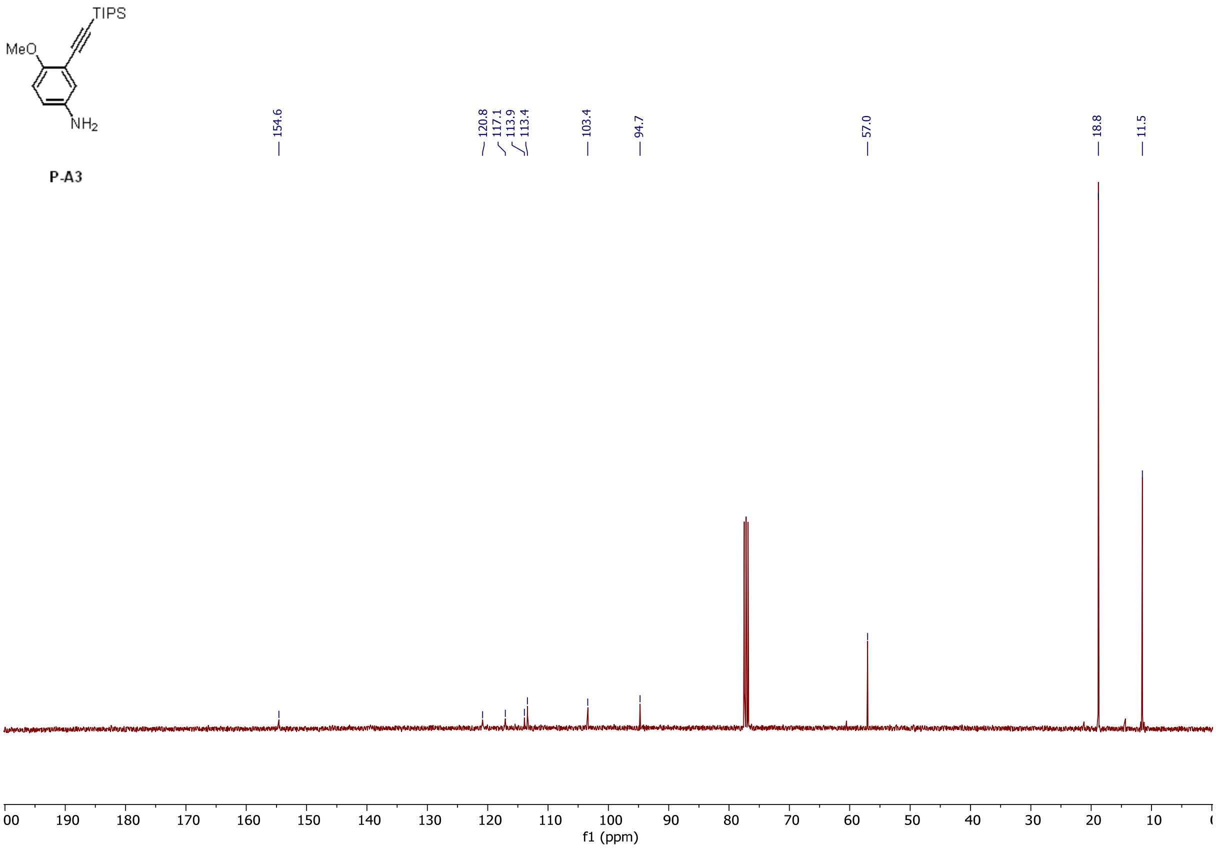

$^{13}C\{^{1}H\}$ NMR (100 MHz, $CDCl_3$) of **P-A3**.

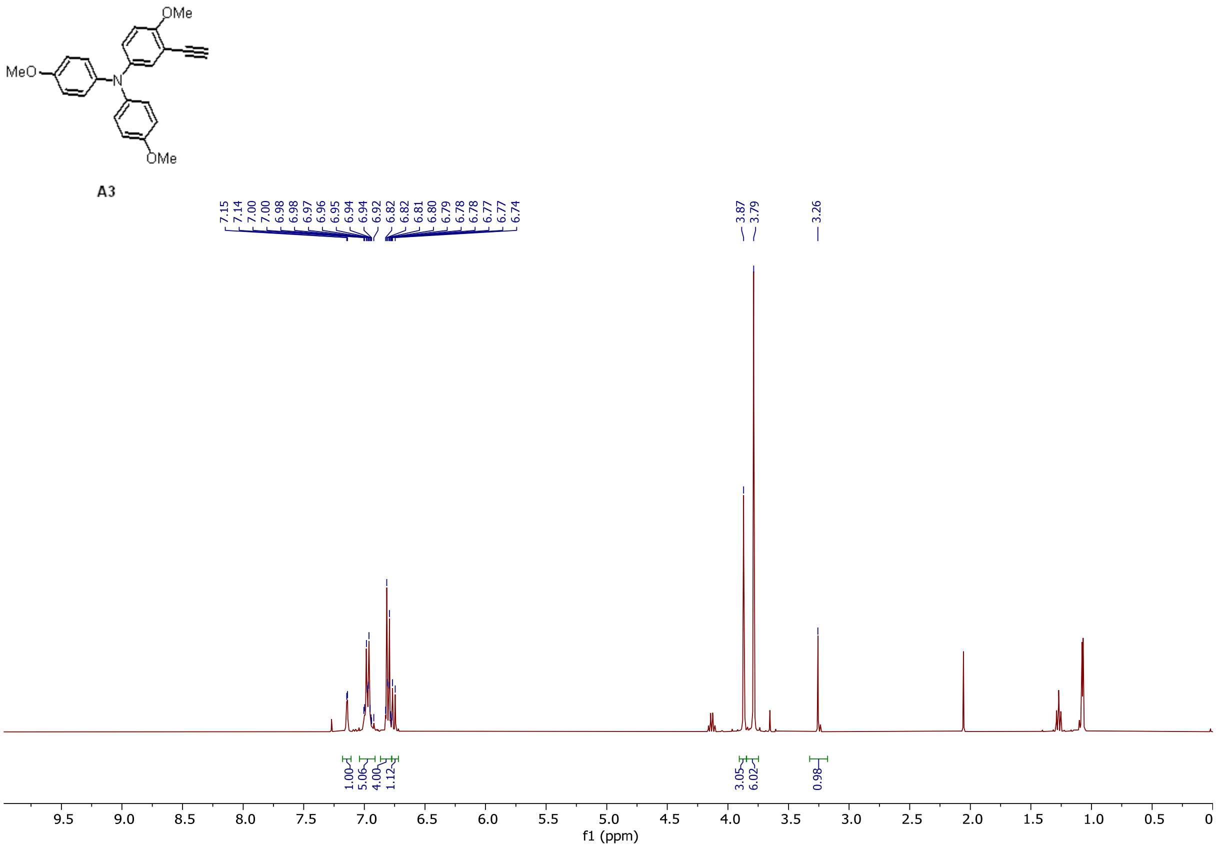

^{1}H NMR (400 MHz, $CDCl_3$) of **A3**.

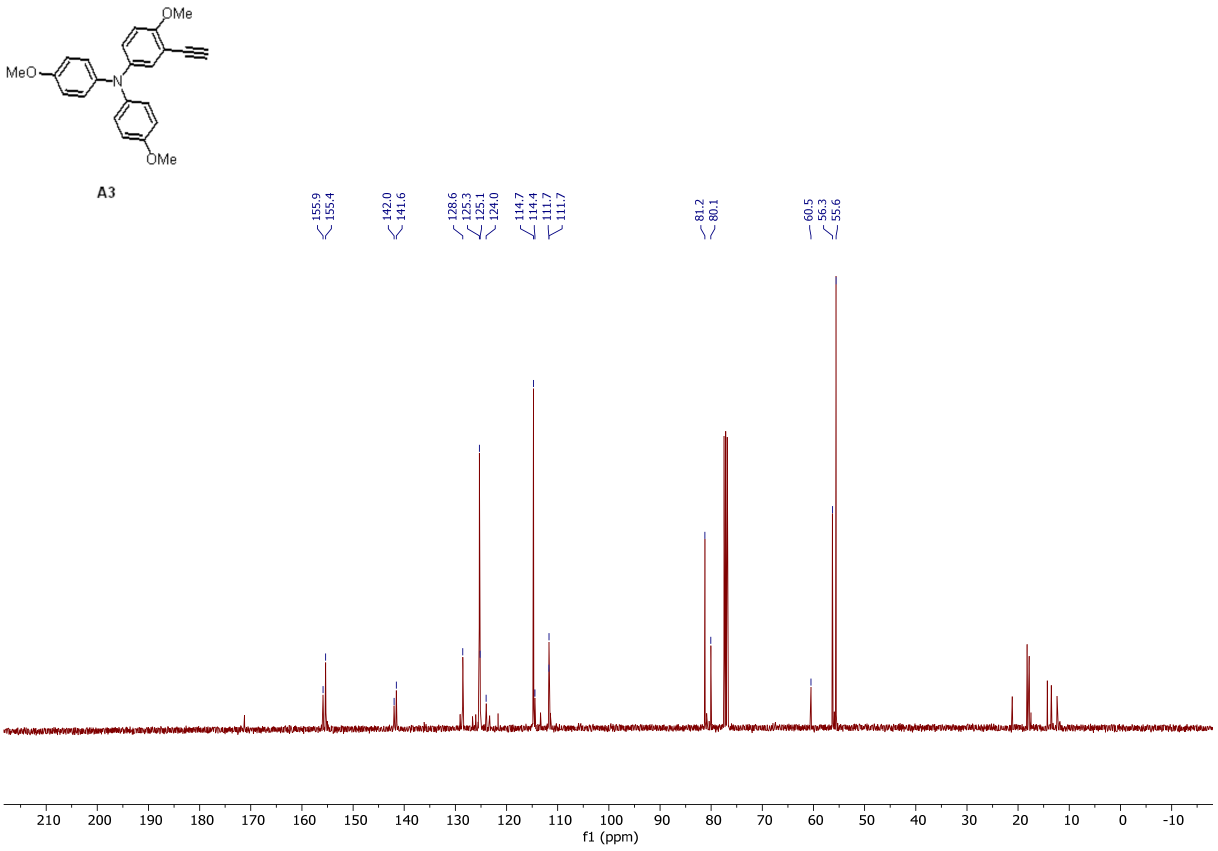

$^{13}C\{^1H\}$ NMR (100 MHz, $CDCl_3$) of **A3**.

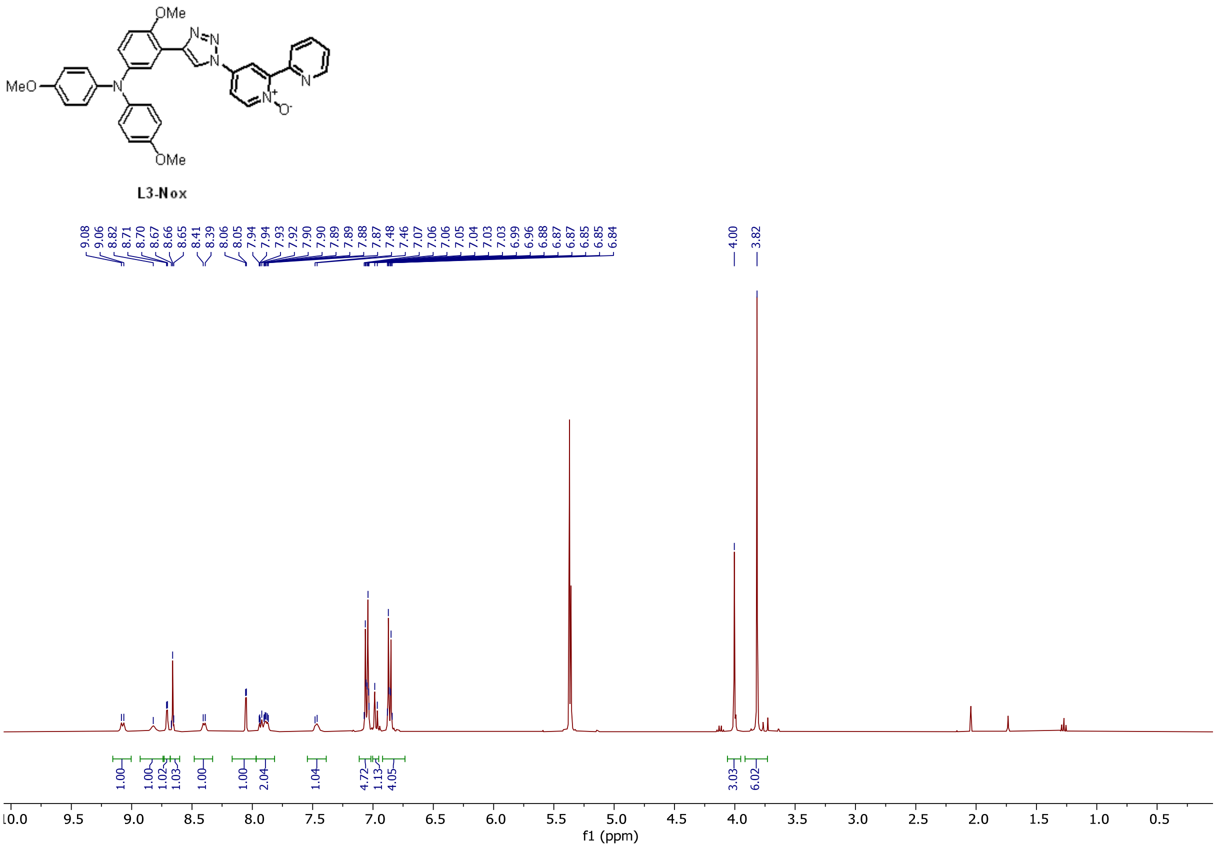

^1H NMR (400 MHz, CD_2Cl_2) of **L3-Nox**.

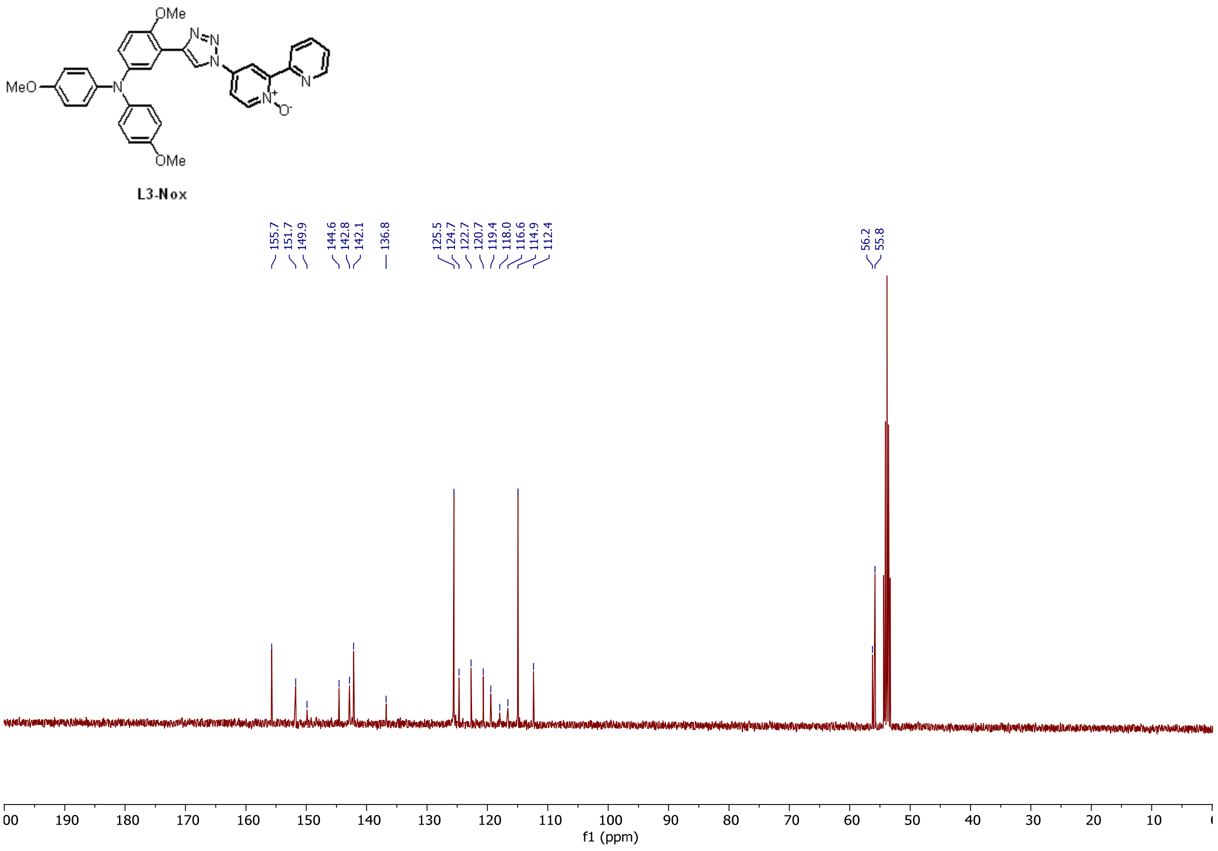

$^{13}C\{^{1}H\}$ NMR (100 MHz, CD_2Cl_2) of **L3-Nox**.

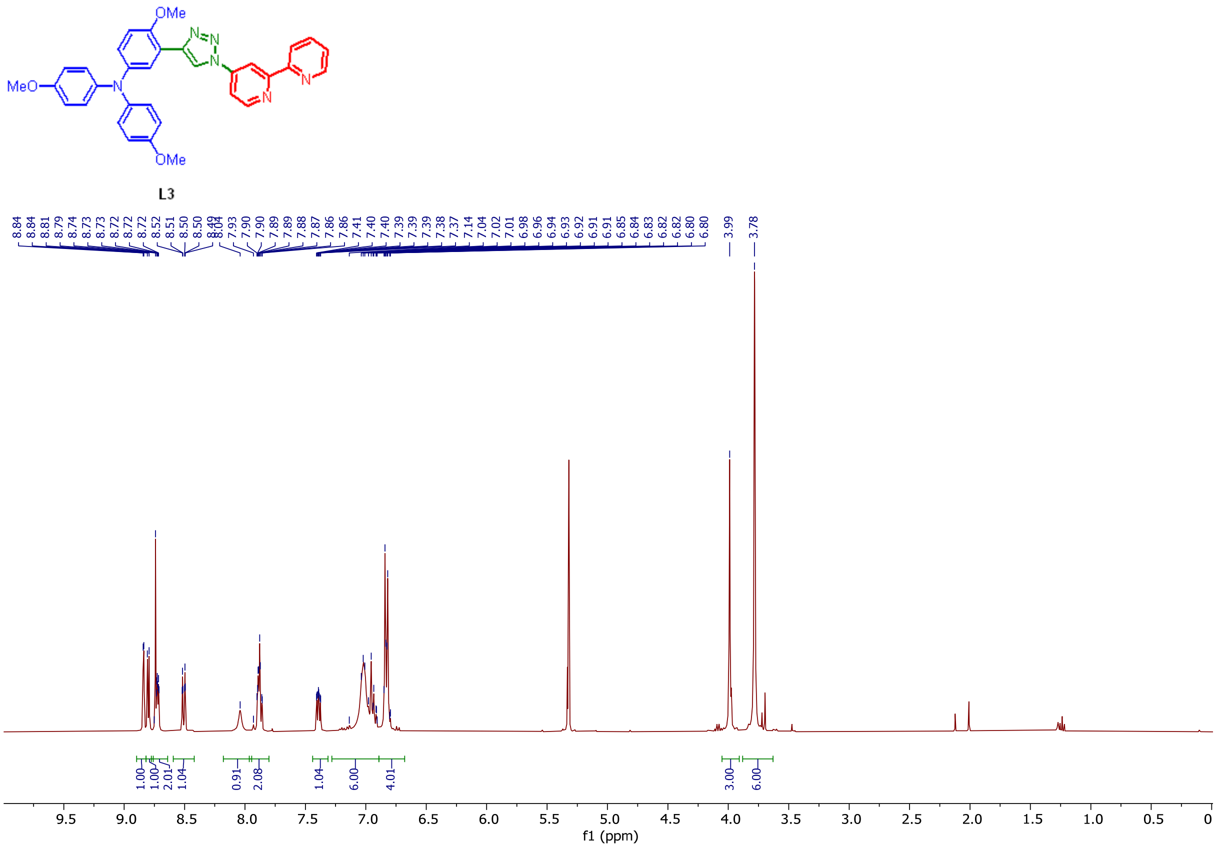

^{1}H NMR (400 MHz, CD_2Cl_2) of **L3**.

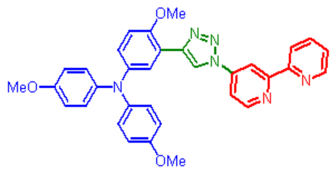

L3

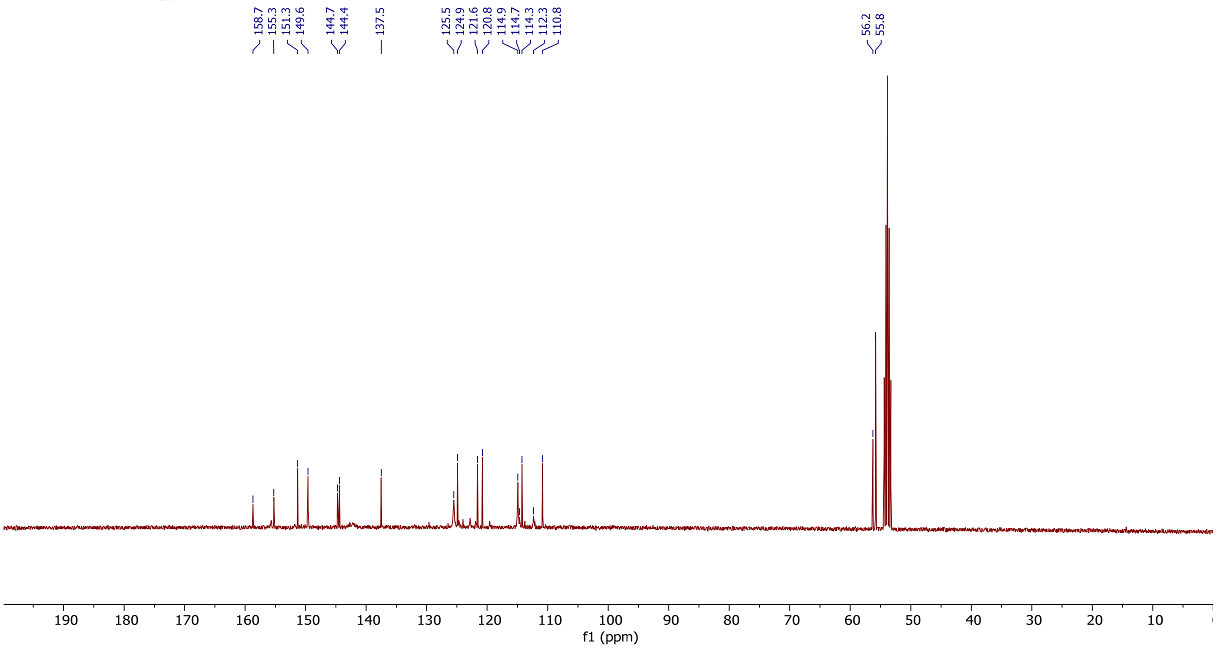

$^{13}C\{^{1}H\}$ NMR (100 MHz, CD_2Cl_2) of **L3**.

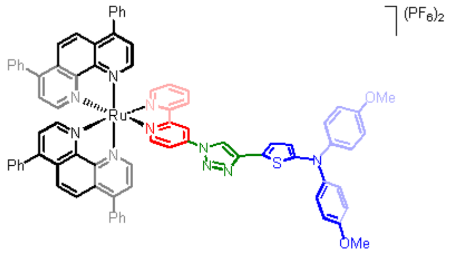

PC2

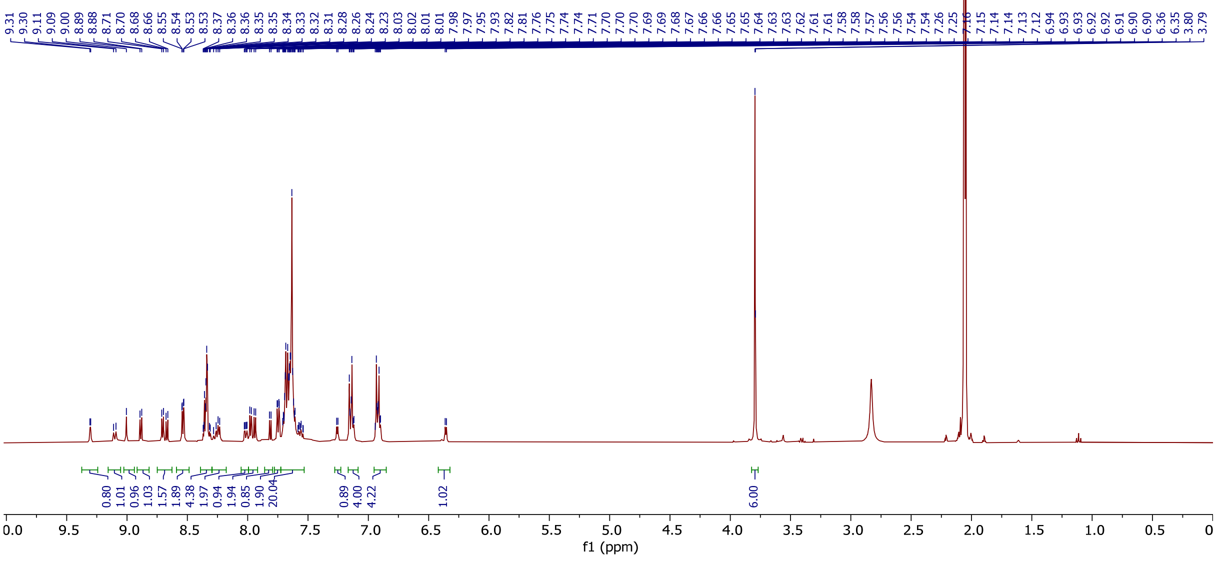

^{1}H NMR (400 MHz, Acetone-d^{6}) of **PC2**.

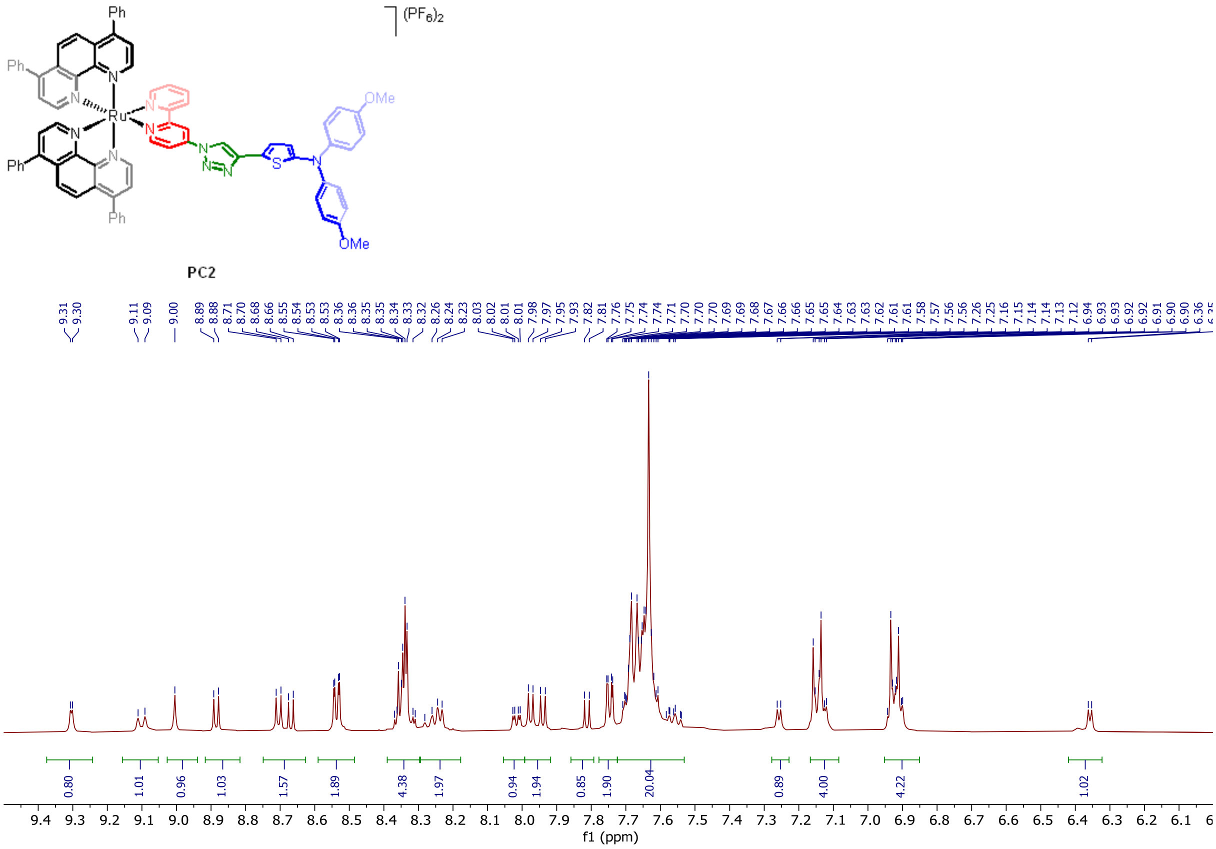

^{1}H NMR (400 MHz, Acetone-*d*[6]) of **PC2** (expansion aromatic area 6.0 – 9.5 ppm).

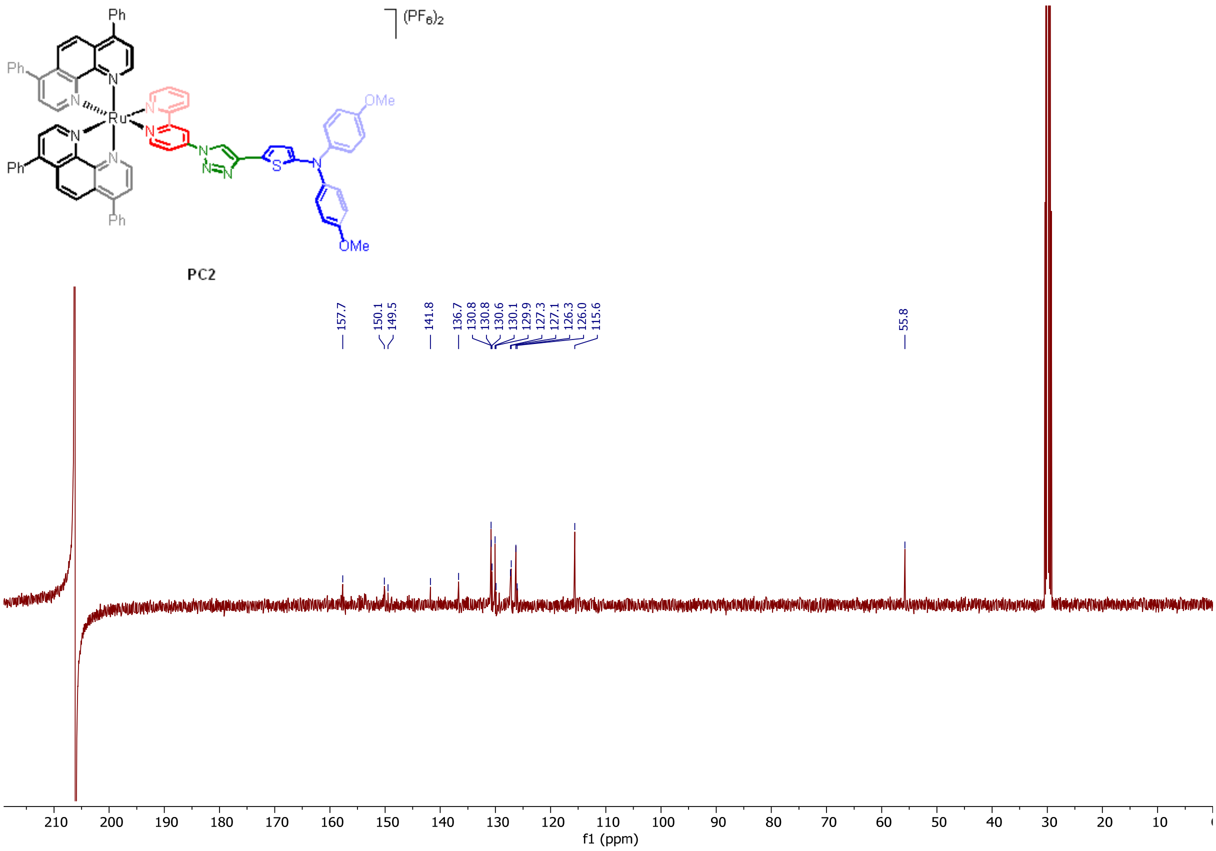

^{13}C{^{1}H} NMR (100 MHz, Acetone-*d*[6]) of **PC2**.

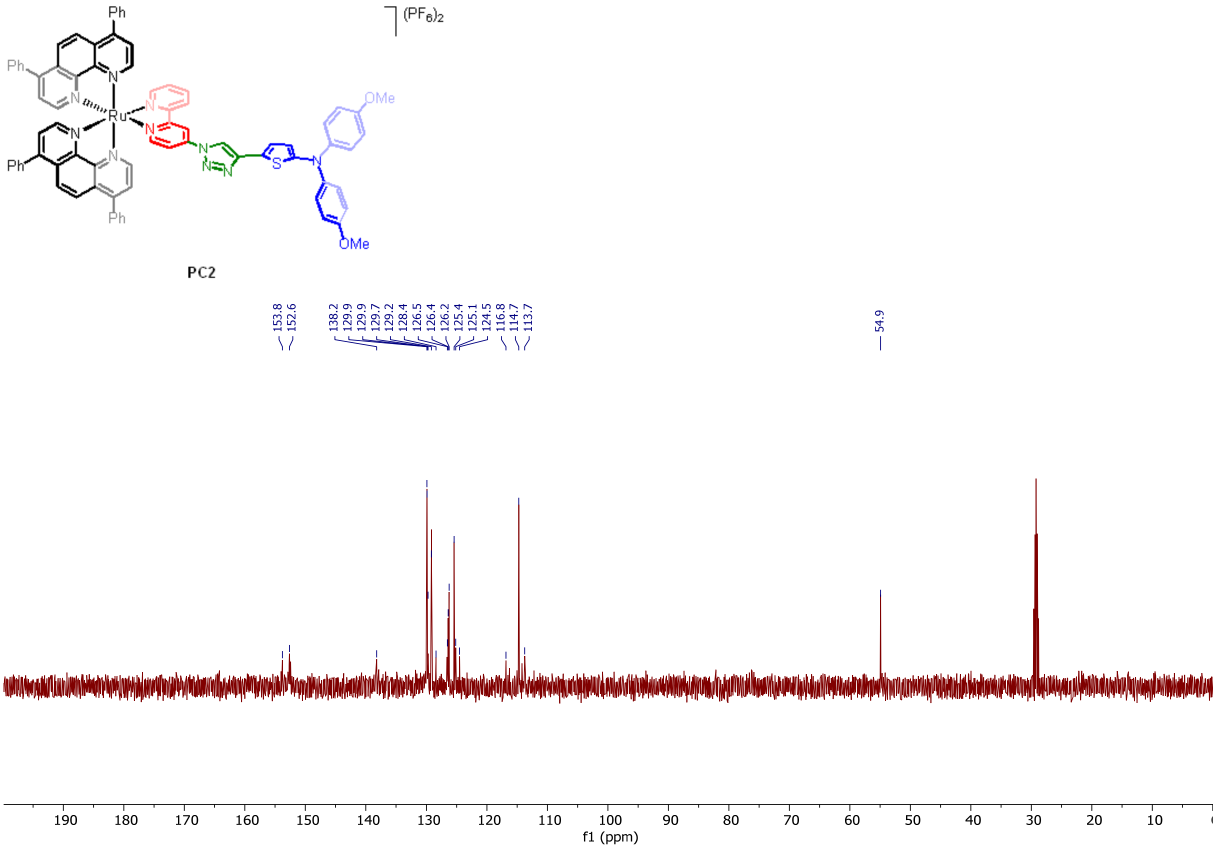

^{13}C-DEPT135 NMR (100 MHz, Acetone-d^6) of **PC2**.

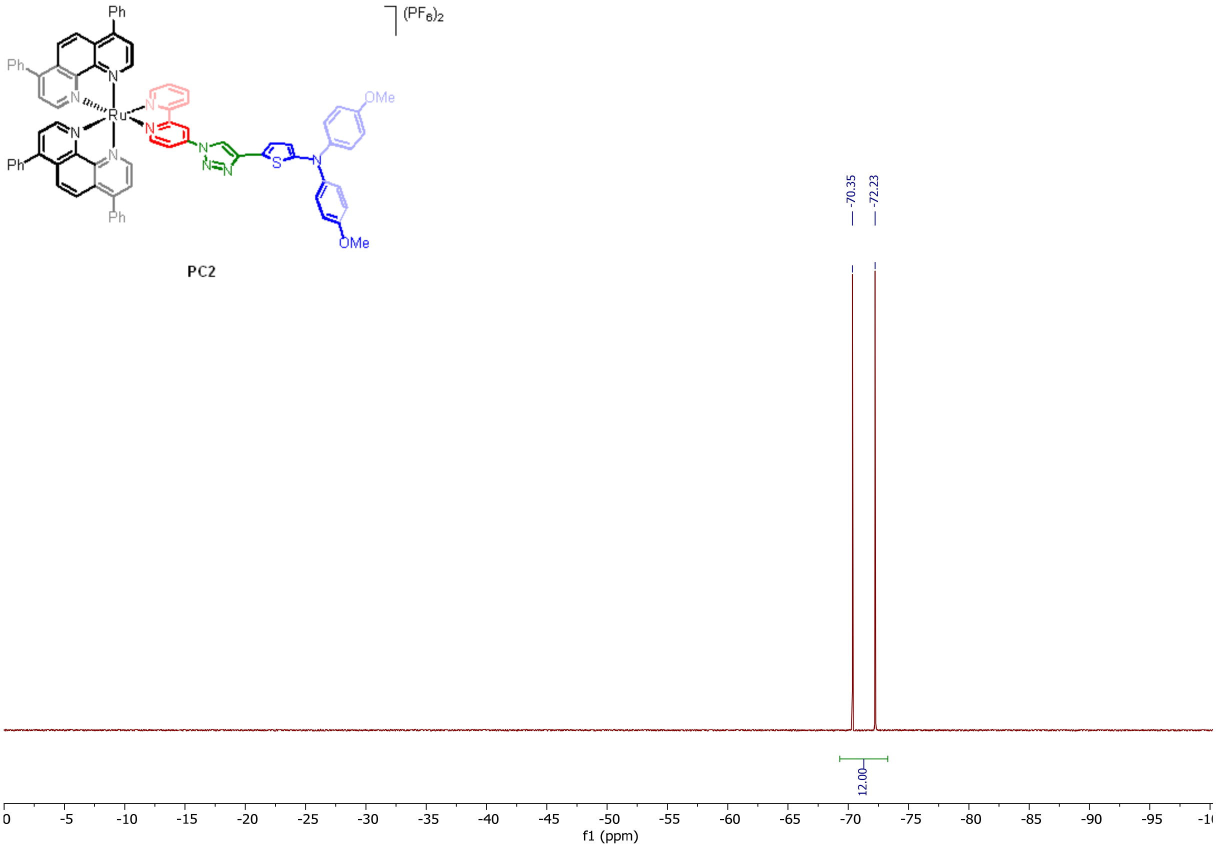

^{19}F{^{1}H} NMR (377 MHz, Acetone-d^6) of **PC2**.

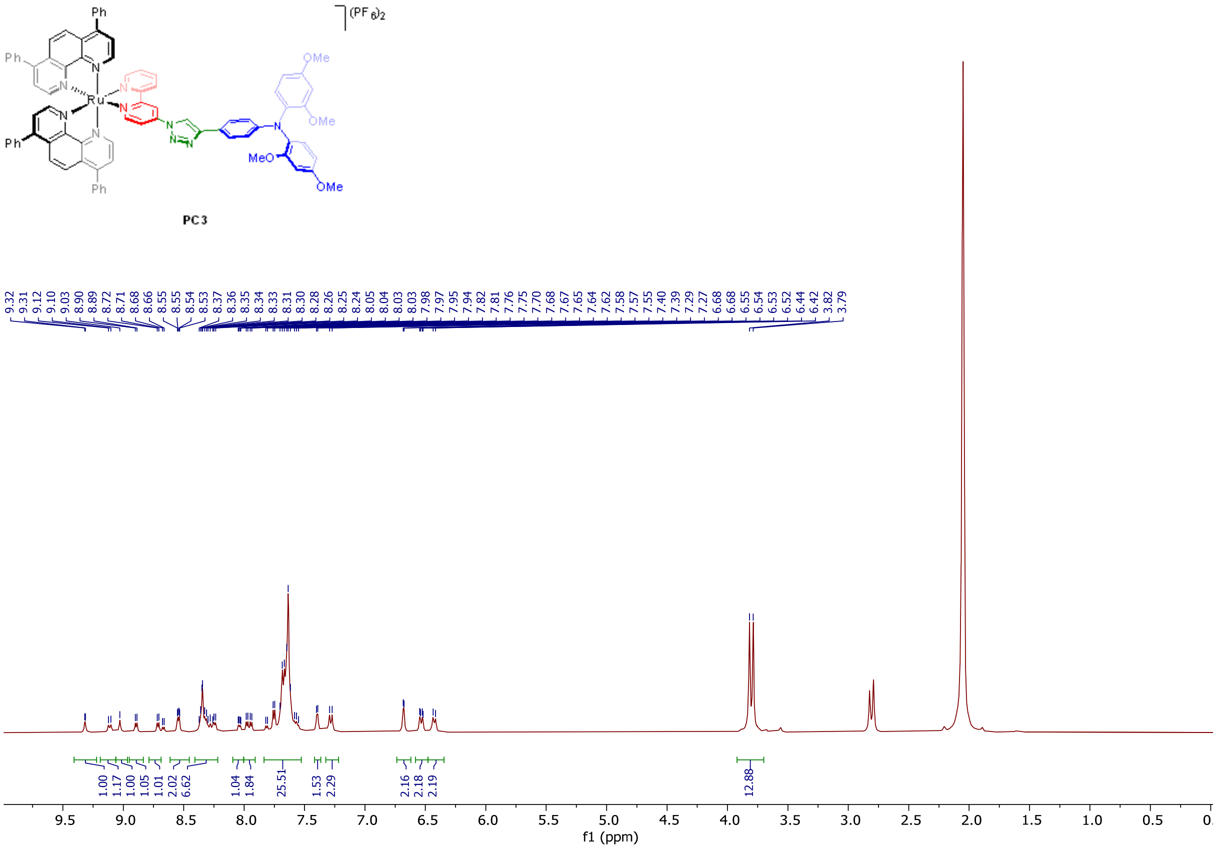

^{1}H NMR (400 MHz, Acetone-d^6) of **PC3**.

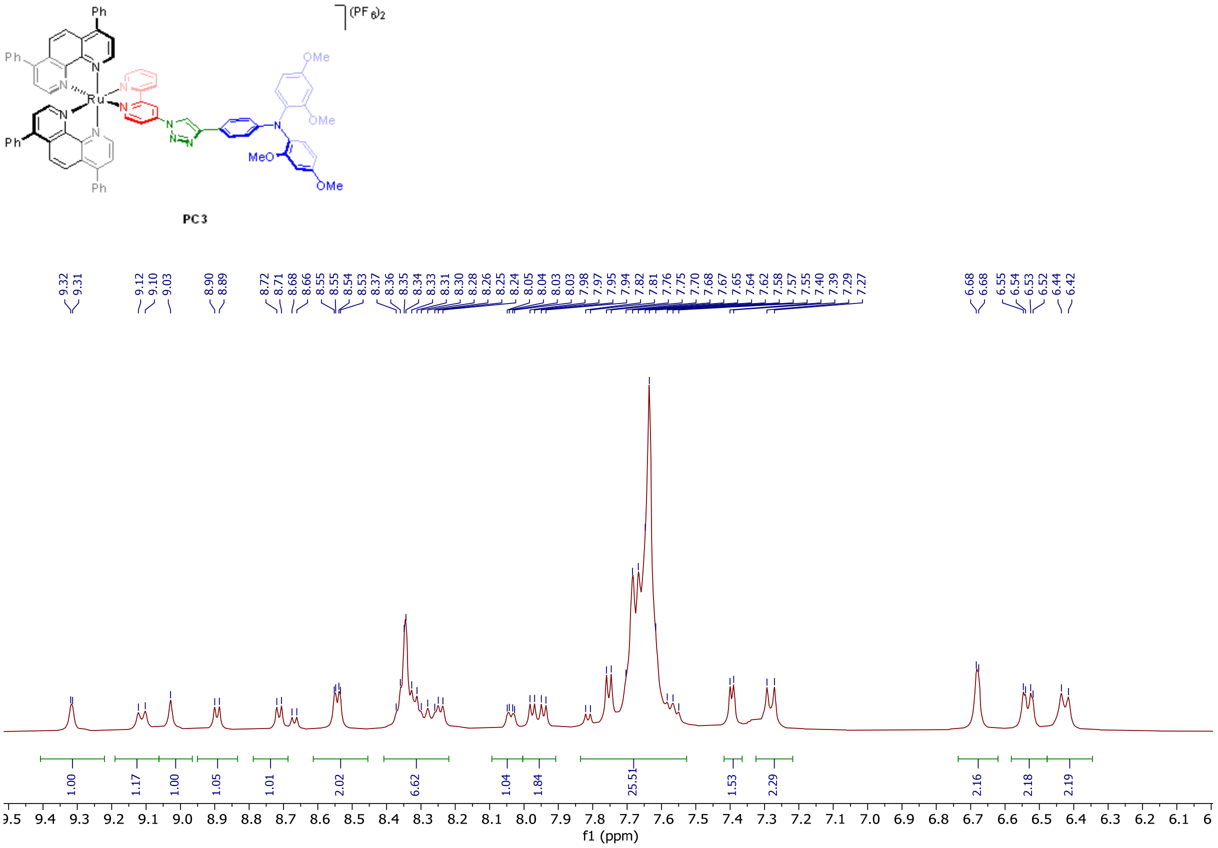

^{1}H NMR (400 MHz, Acetone-d^6) of **PC3** (expansion aromatic area 6.0 – 9.5 ppm).

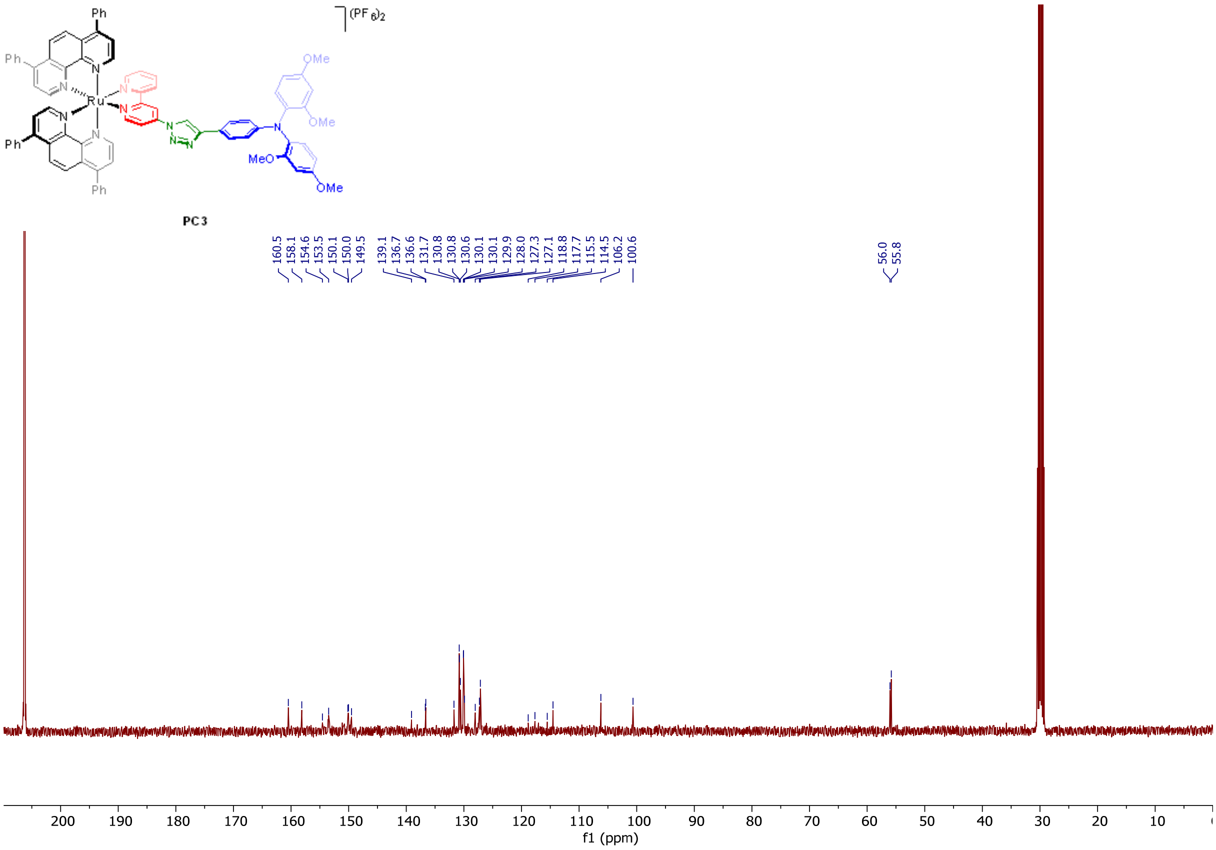

$^{13}C\{^1H\}$ NMR (100 MHz, Acetone-d^6) of **PC3**.

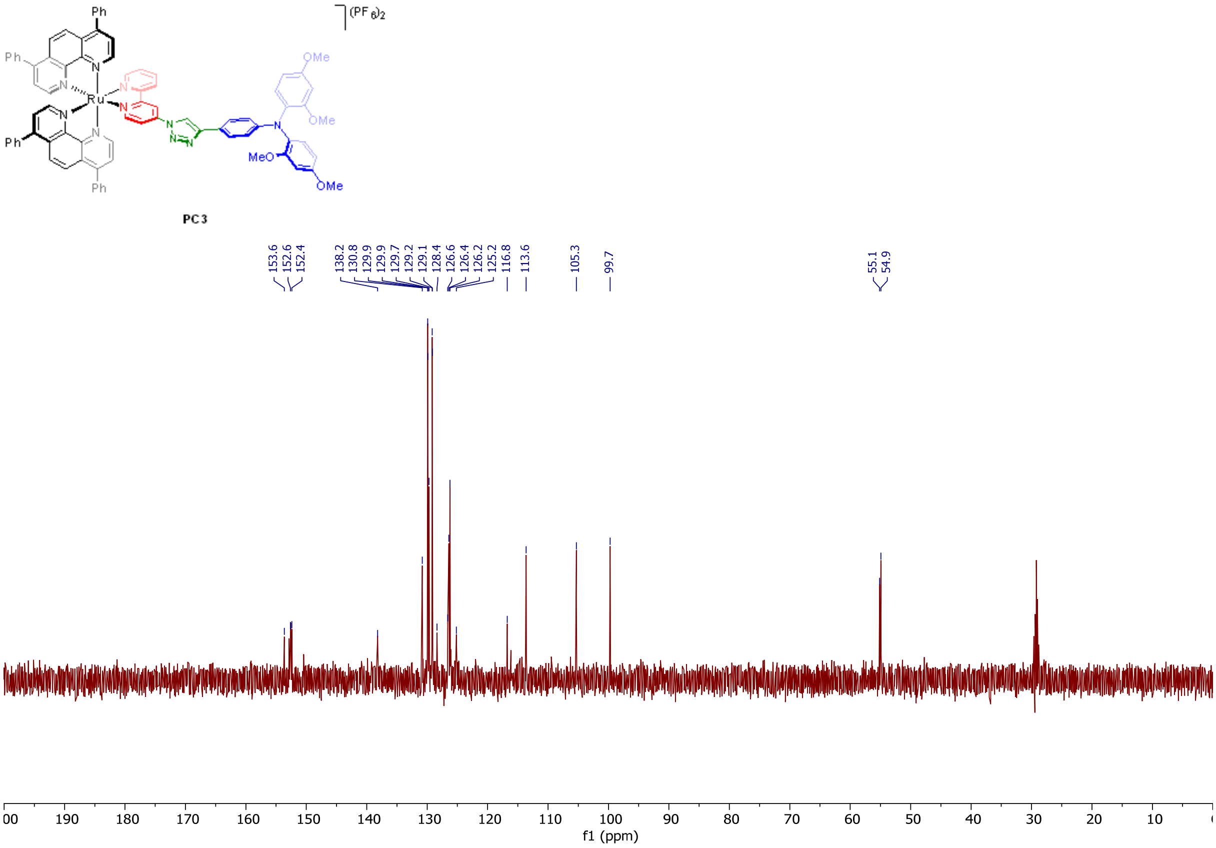

^{13}C-DEPT135 NMR (100 MHz, Acetone-d^6) of **PC3**.

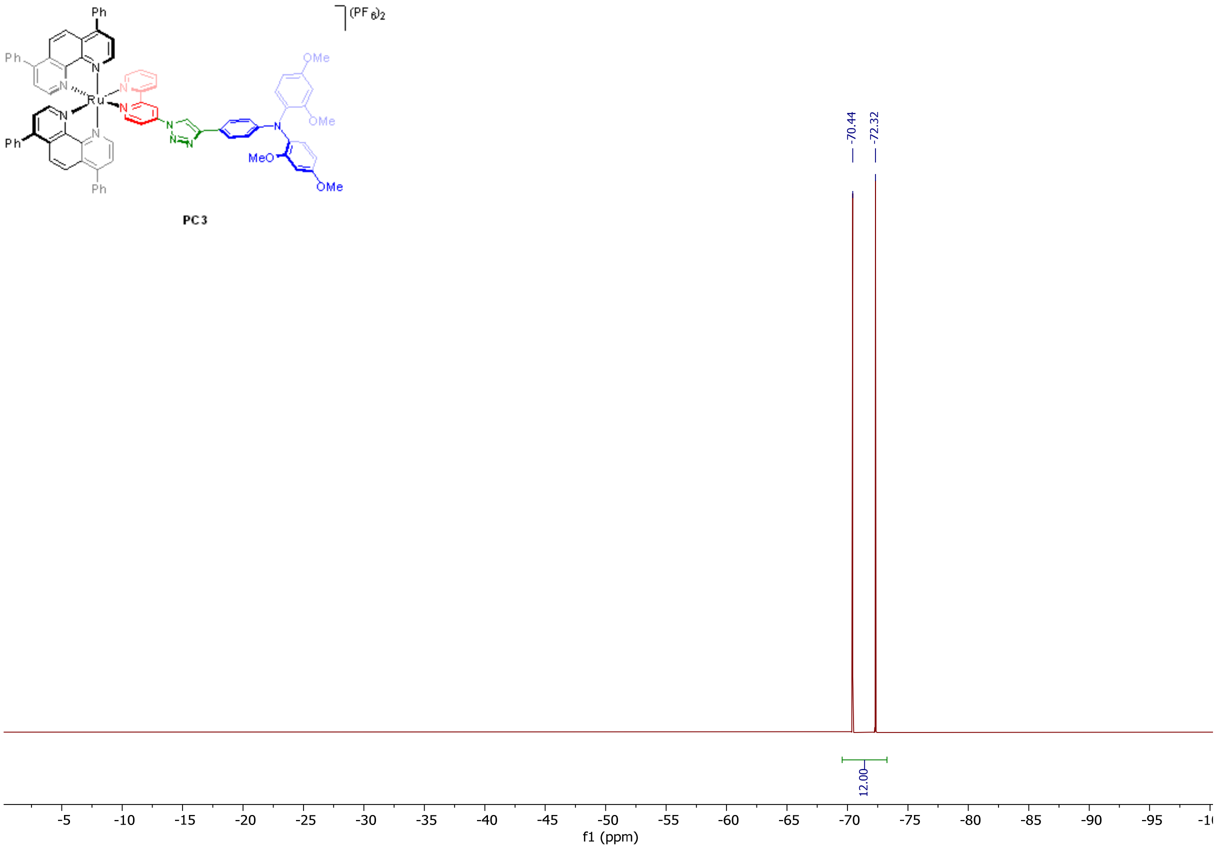

$^{19}F\{^{1}H\}$ NMR (377 MHz, Acetone-d^6) of **PC3**.

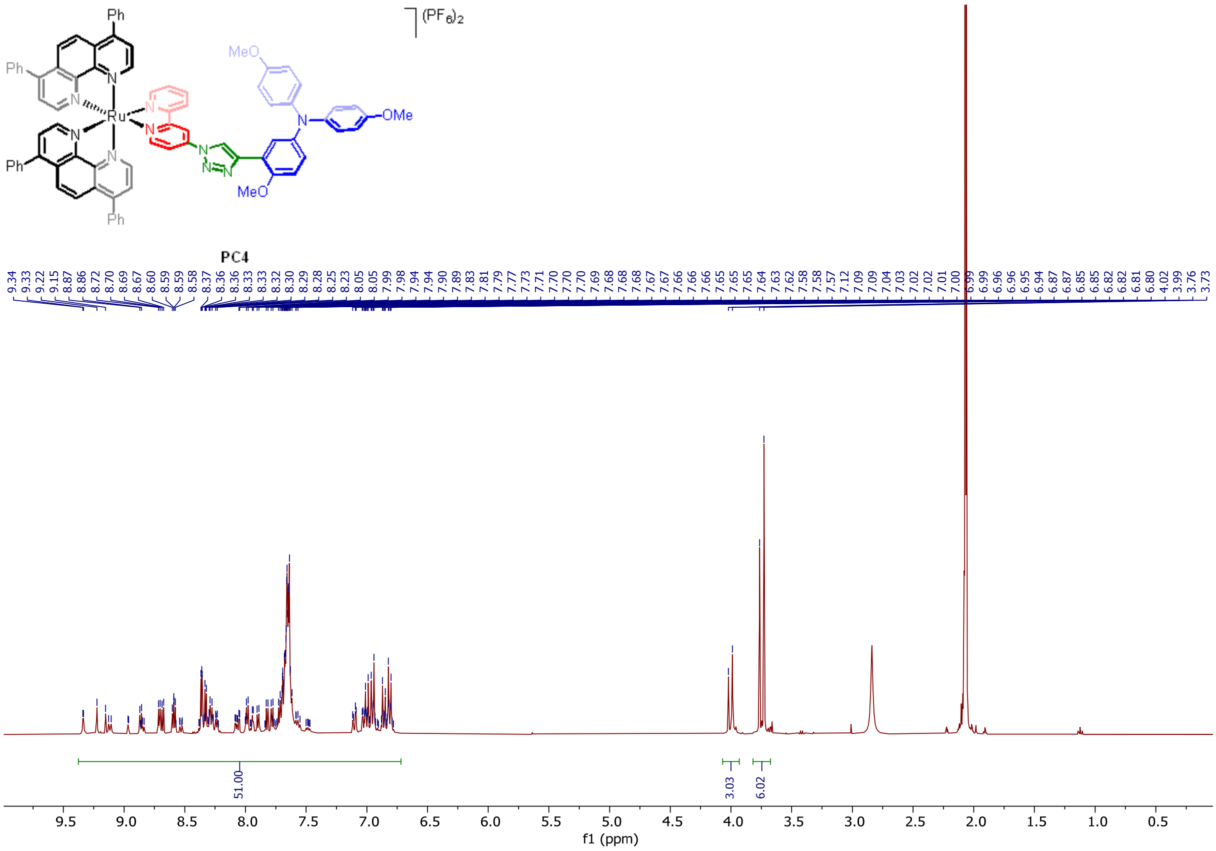

^{1}H NMR (400 MHz, Acetone-d^6) of **PC4**.

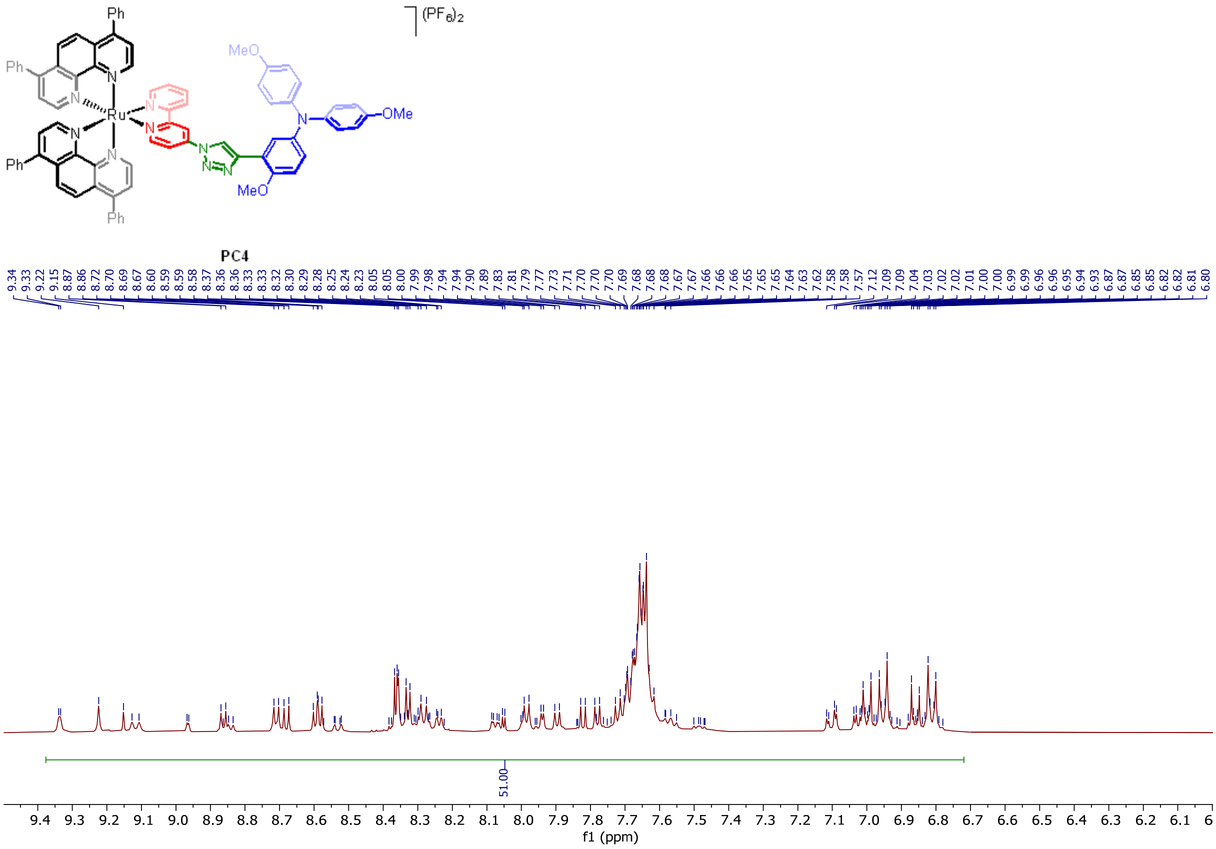

^{1}H NMR (400 MHz, Acetone-d^{6}) of **PC4** (expansion aromatic area 6.0 – 9.5 ppm).

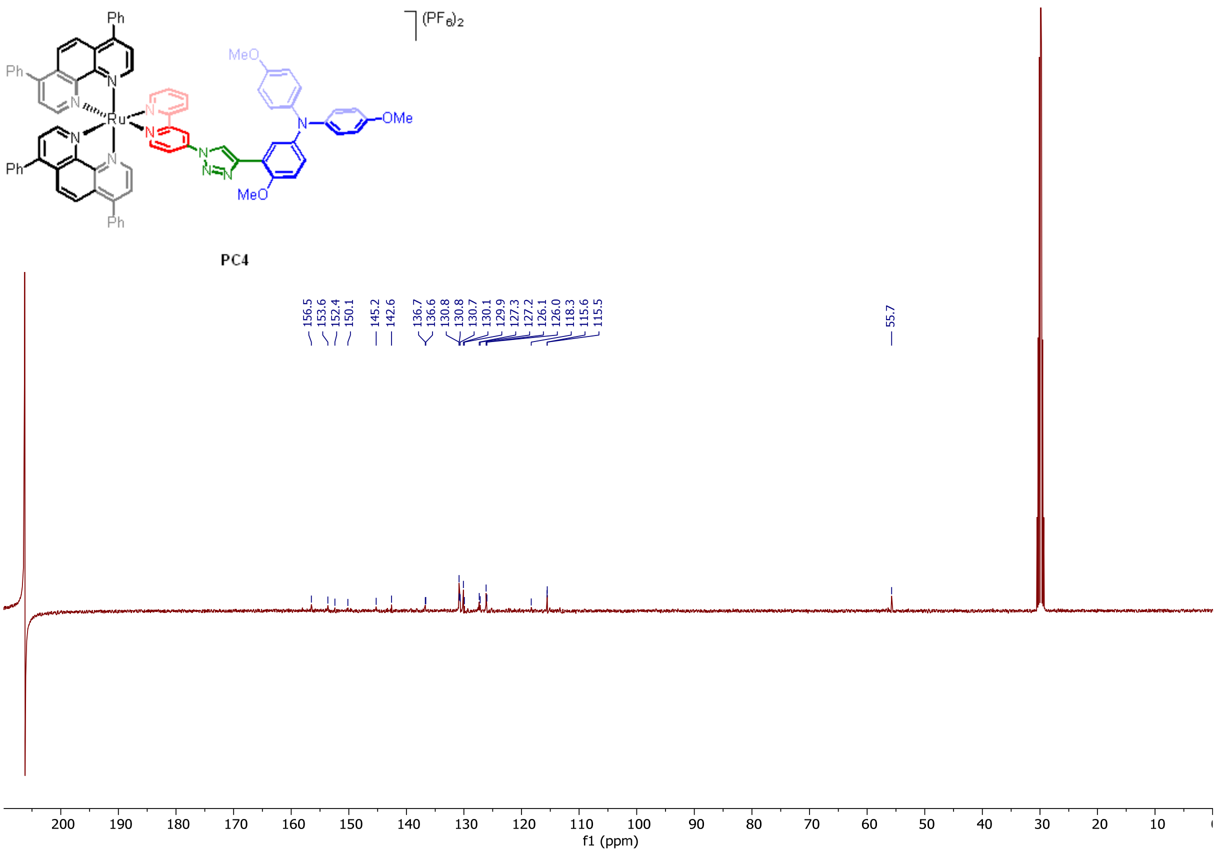

$^{13}C\{^{1}H\}$ NMR (100 MHz, Acetone-d^{6}) of **PC4**.

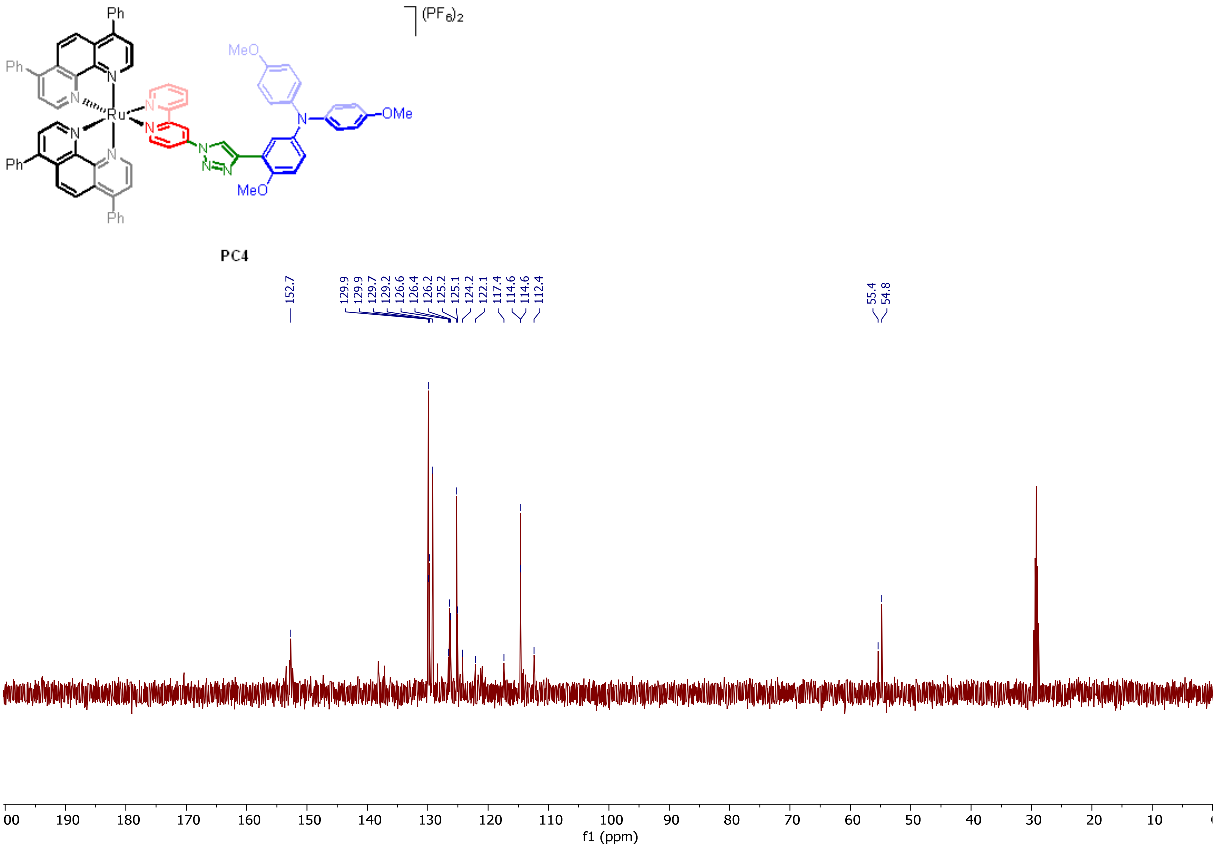

^{13}C-DEPT135 NMR (100 MHz, Acetone-d^6) of **PC4**.

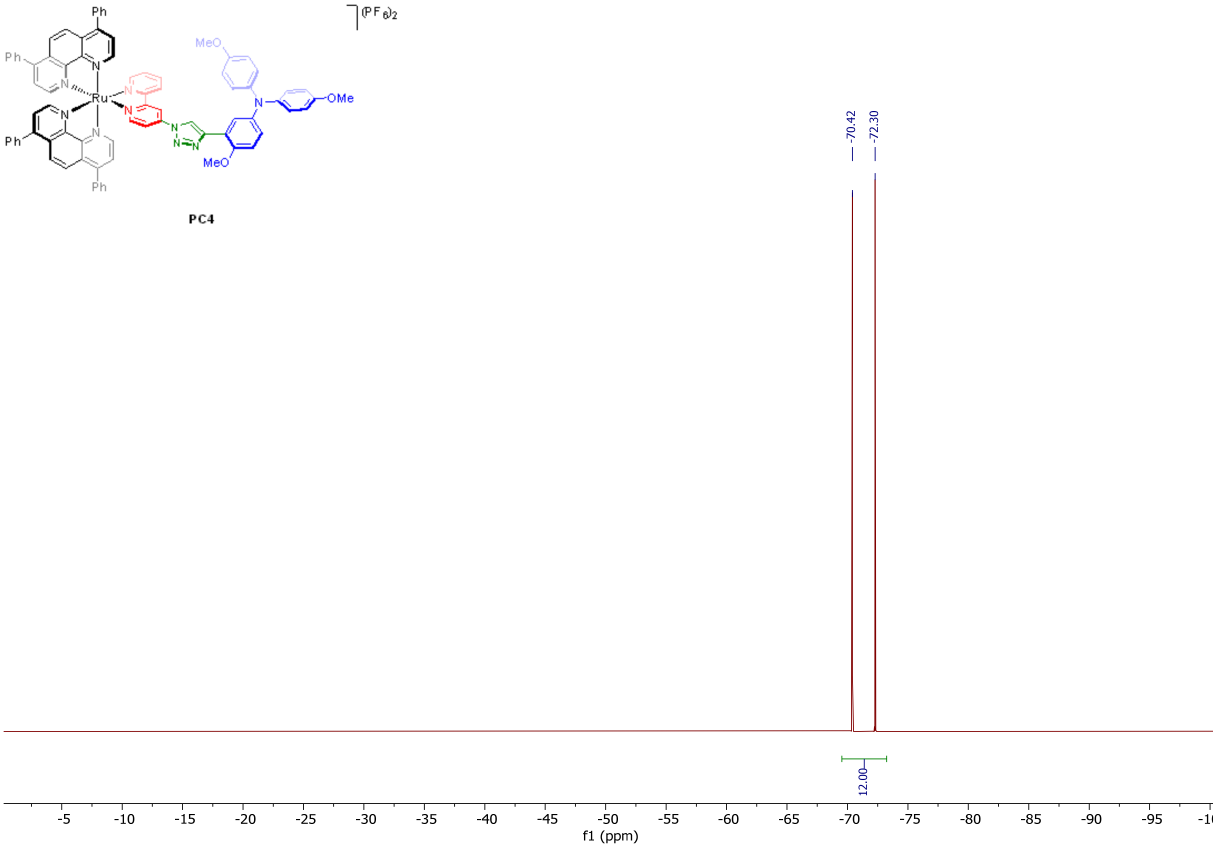

^{19}F{^{1}H} NMR (377 MHz, Acetone-d^6) of **PC4**.

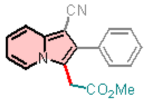

2a

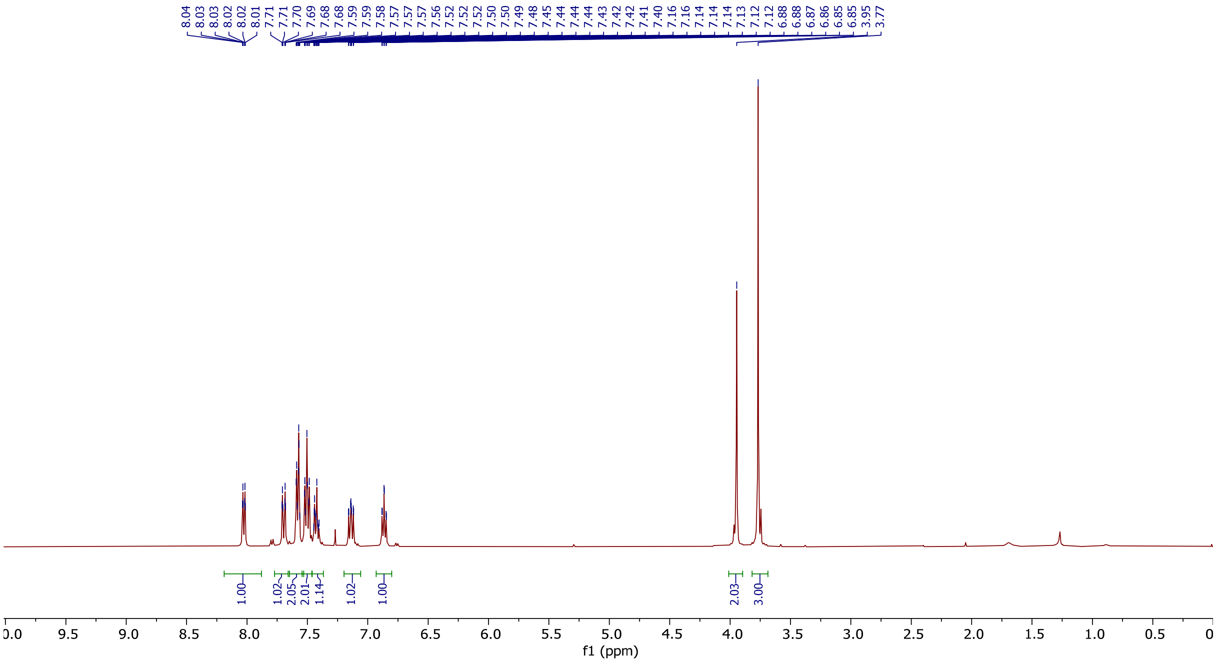

^{1}H NMR (400 MHz, $CDCl_3$) of **2a**.

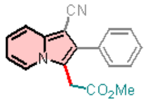

2a

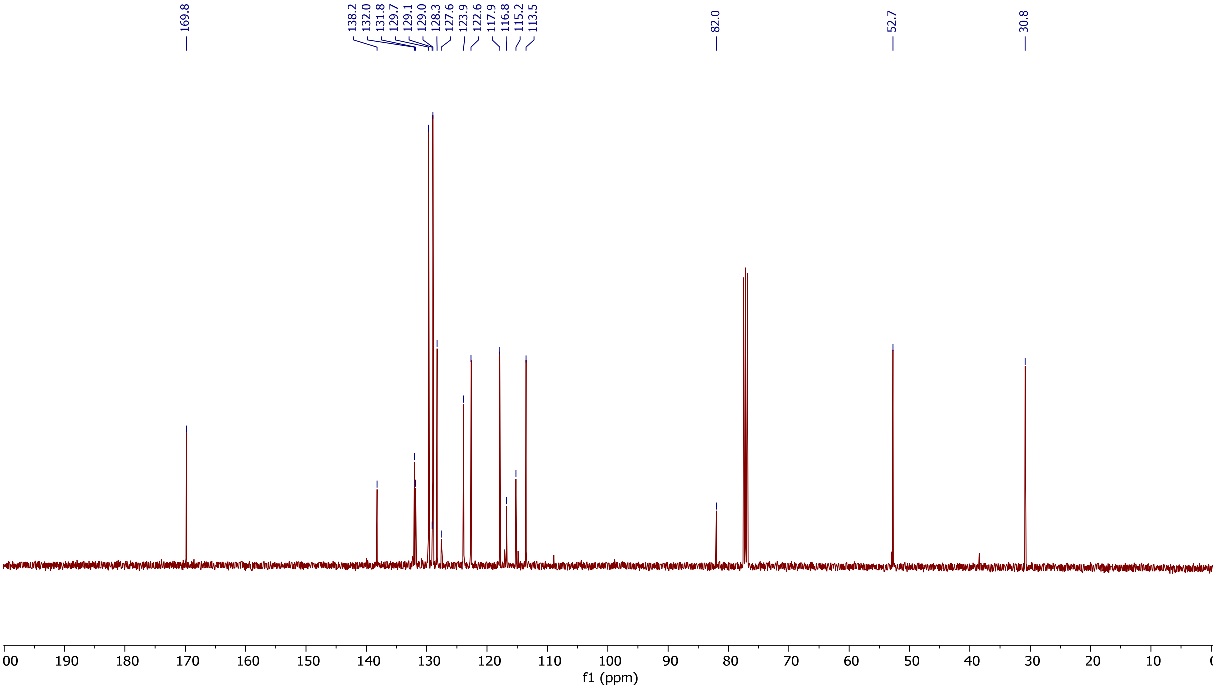

^{13}C{^{1}H} NMR (100 MHz, $CDCl_3$) of **2a**.

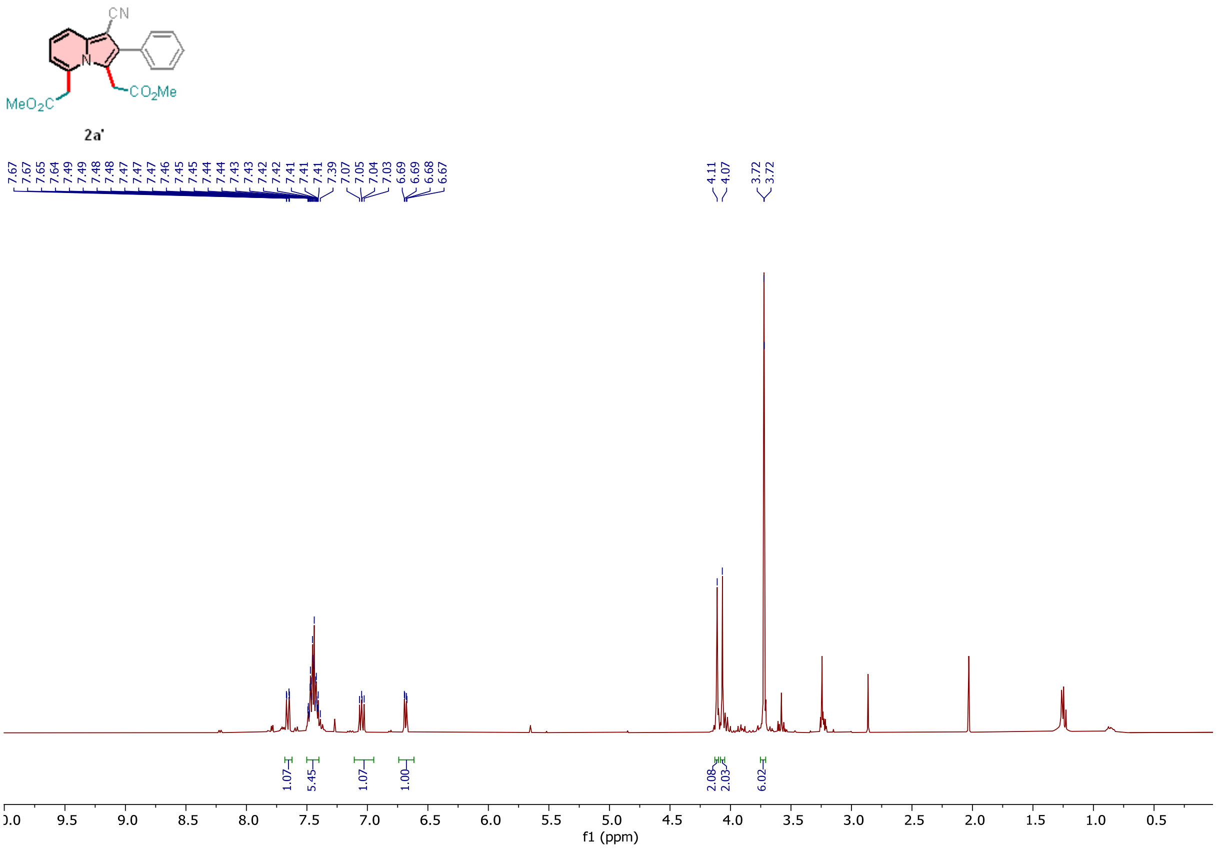

^{1}H NMR (400 MHz, $CDCl_3$) of **2a'**.

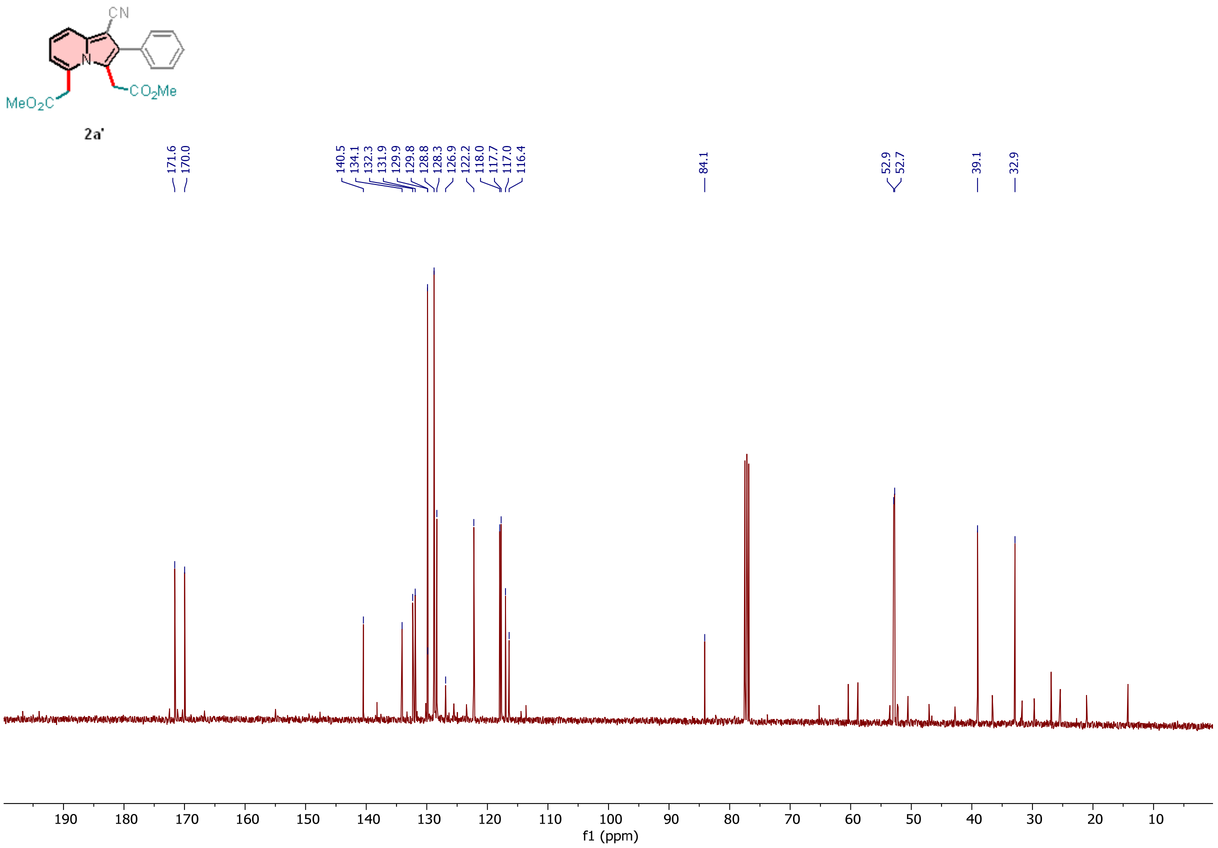

$^{13}C\{^{1}H\}$ NMR (100 MHz, $CDCl_3$) of **2a'**.

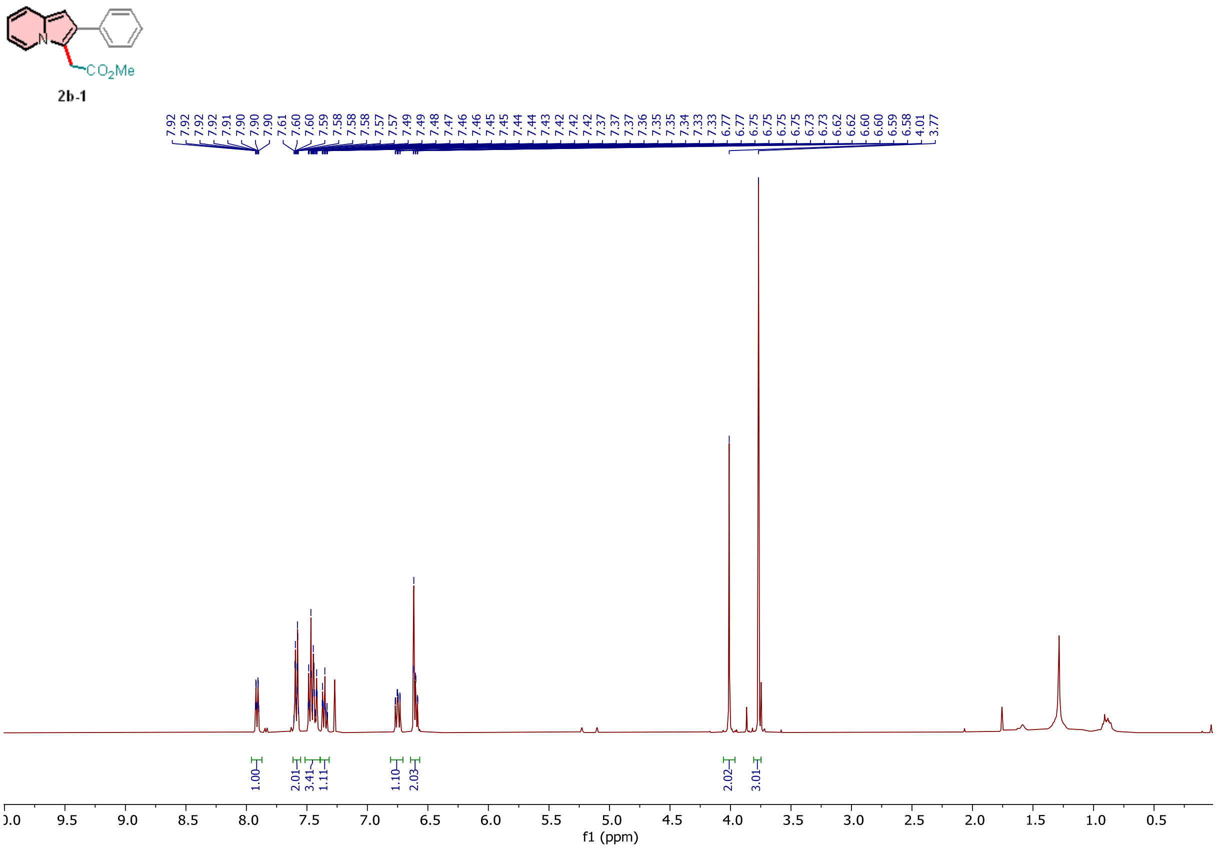

^{1}H NMR (400 MHz, $CDCl_3$) of **2b-1**.

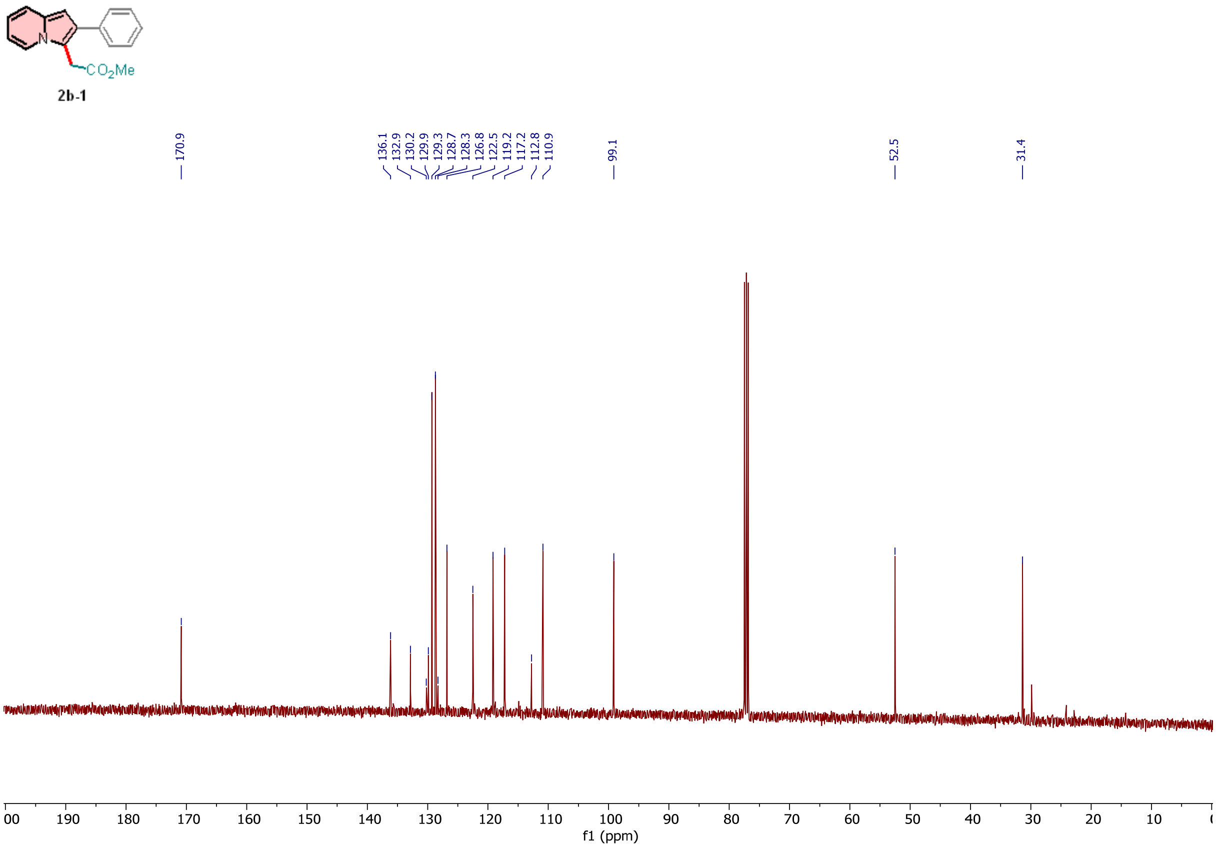

$^{13}C\{^{1}H\}$ NMR (100 MHz, $CDCl_3$) of **2b-1**.

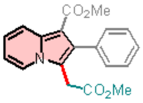

2c-1

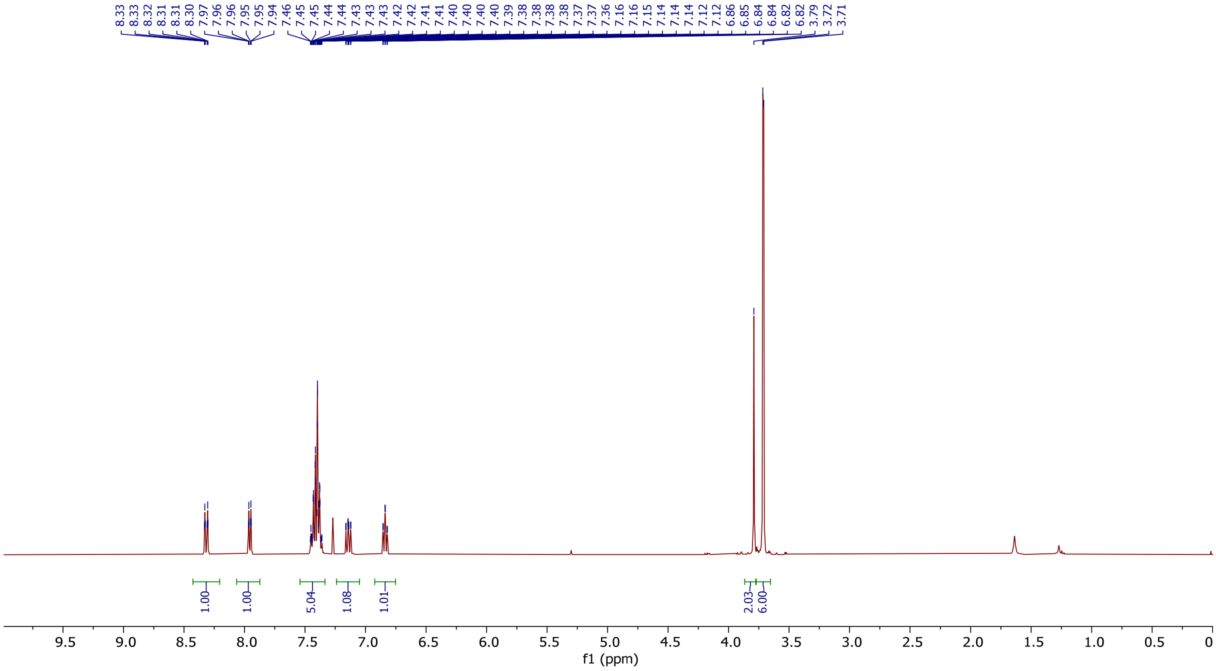

^{1}H NMR (400 MHz, $CDCl_3$) of **2c-1**.

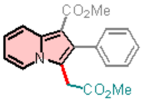

2c-1

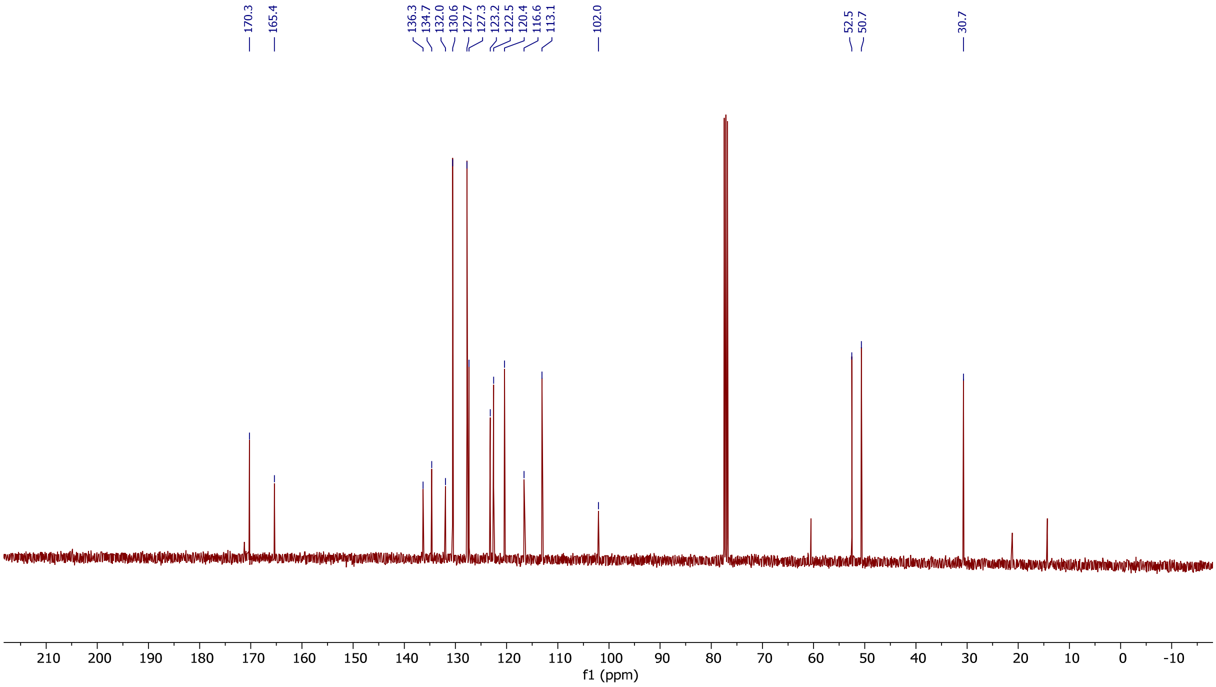

$^{13}C\{^1H\}$ NMR (100 MHz, $CDCl_3$) of **2c-1**.

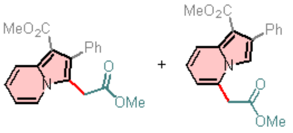

2c-1 + **2c-1'** (1.5:1)

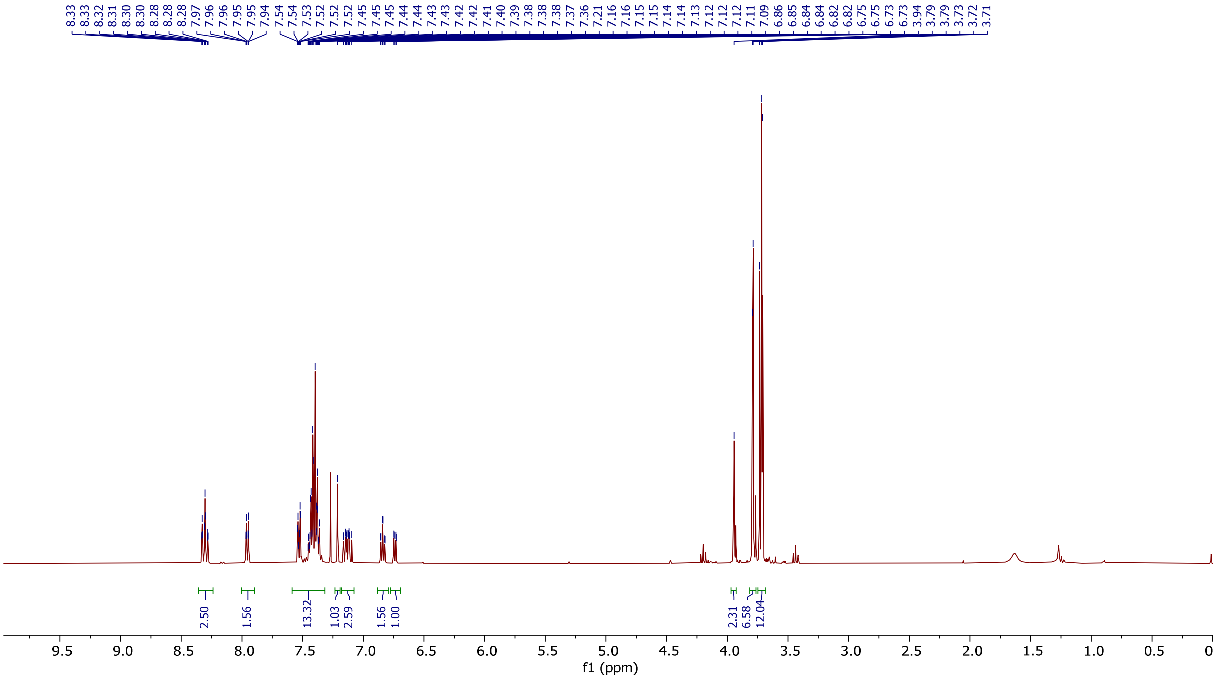

^{1}H NMR (400 MHz, $CDCl_3$) of **2c-1 + 2c-1'**.

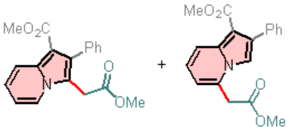

2c-1 + **2c-1'** (1.5:1)

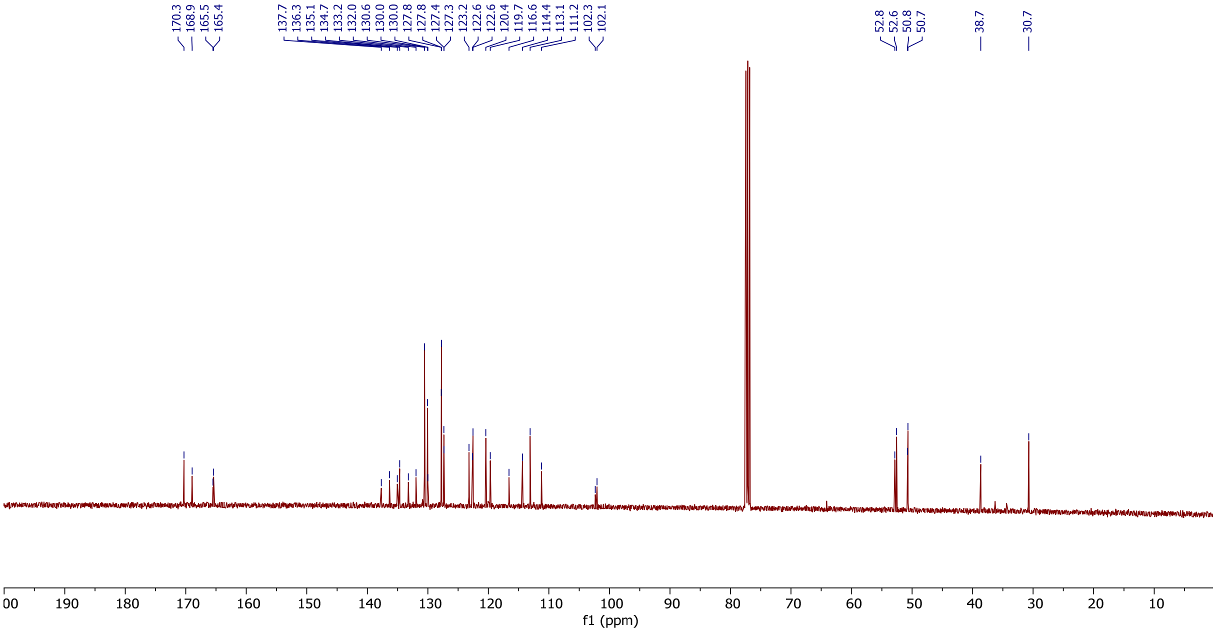

^{13}C{^{1}H} NMR (100 MHz, $CDCl_3$) of **2c-1 + 2c-1'**.

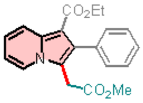

2d-1

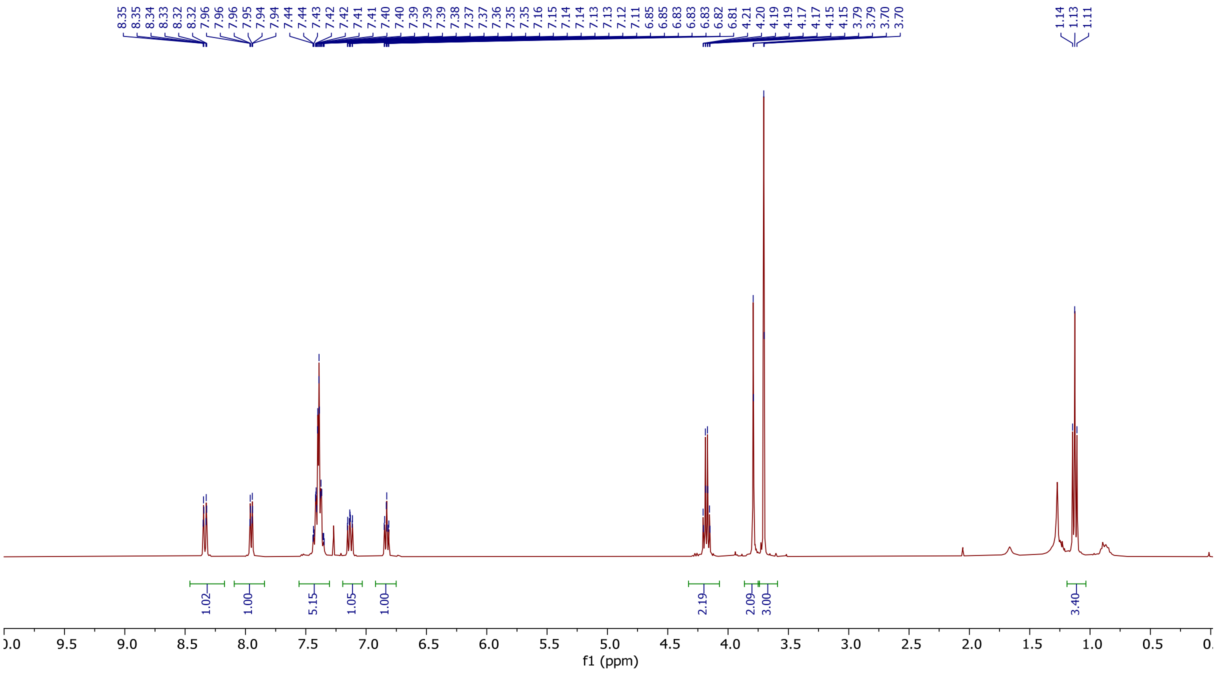

^{1}H NMR (400 MHz, $CDCl_3$) of **2d-1**.

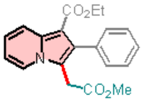

2d-1

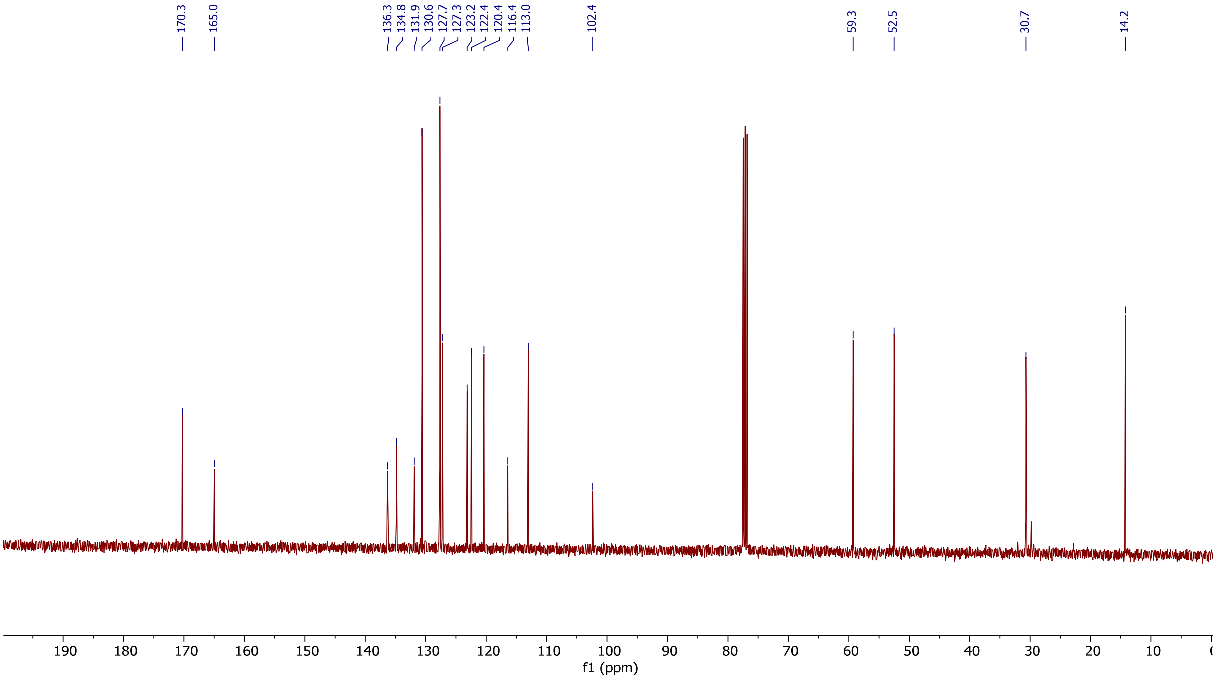

$^{13}C\{^{1}H\}$ NMR (100 MHz, $CDCl_3$) of **2d-1**.

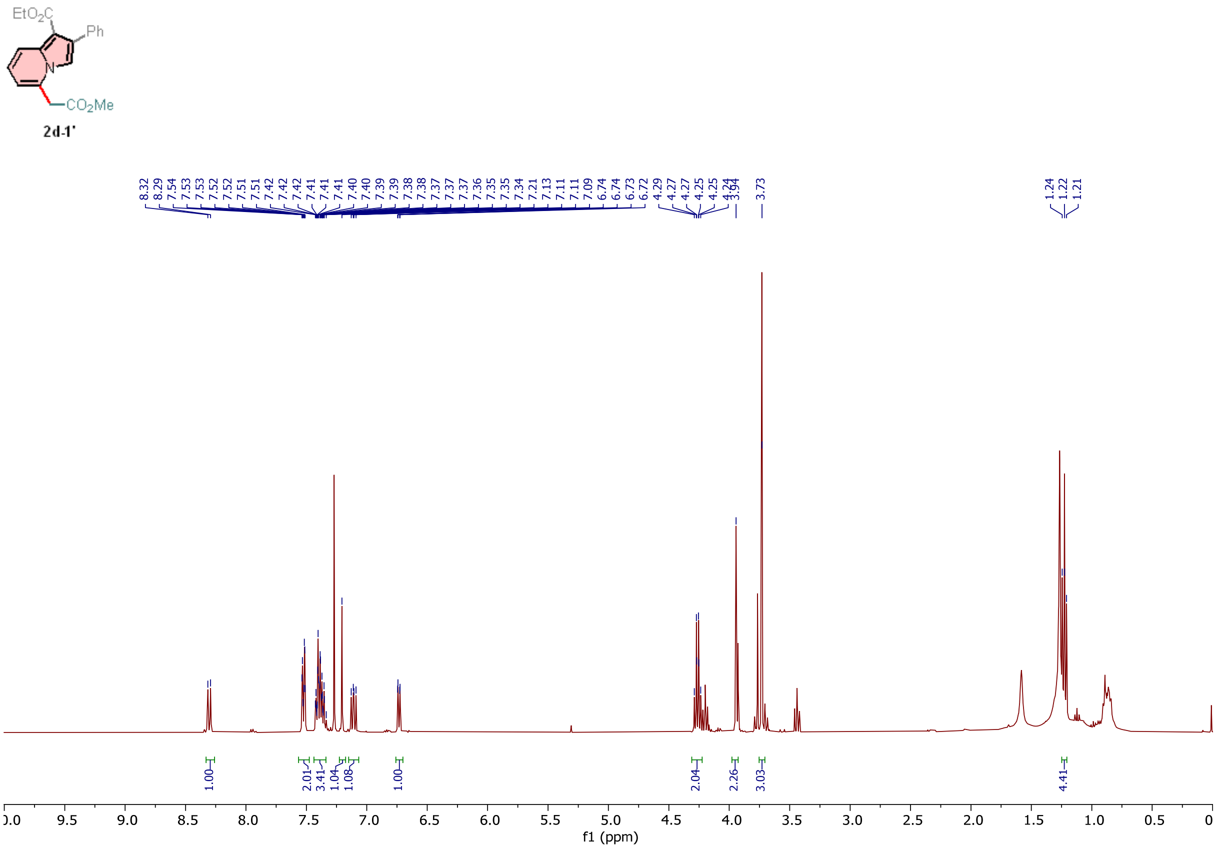

^{1}H NMR (400 MHz, CDCl$_3$) of **2d-1'**.

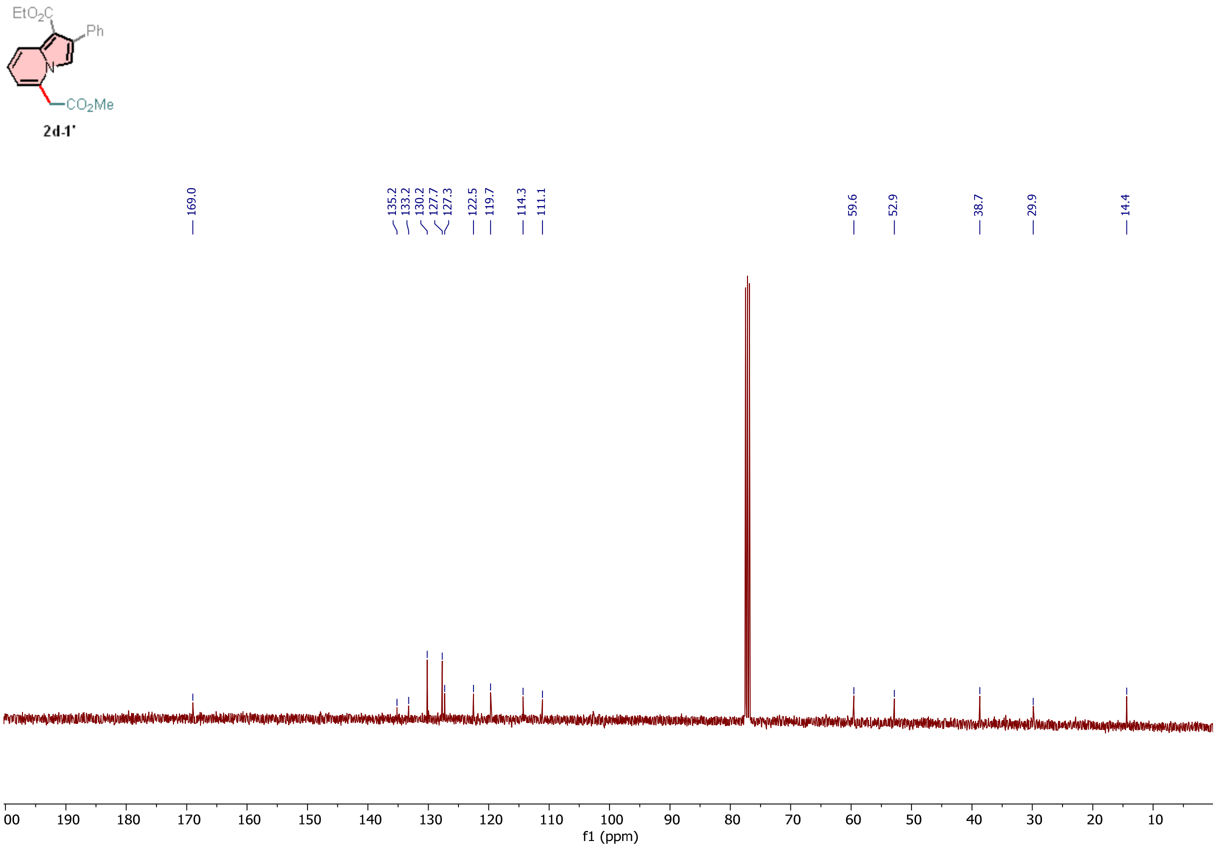

^{13}C{^{1}H} NMR (100 MHz, CDCl$_3$) of **2d-1'**.

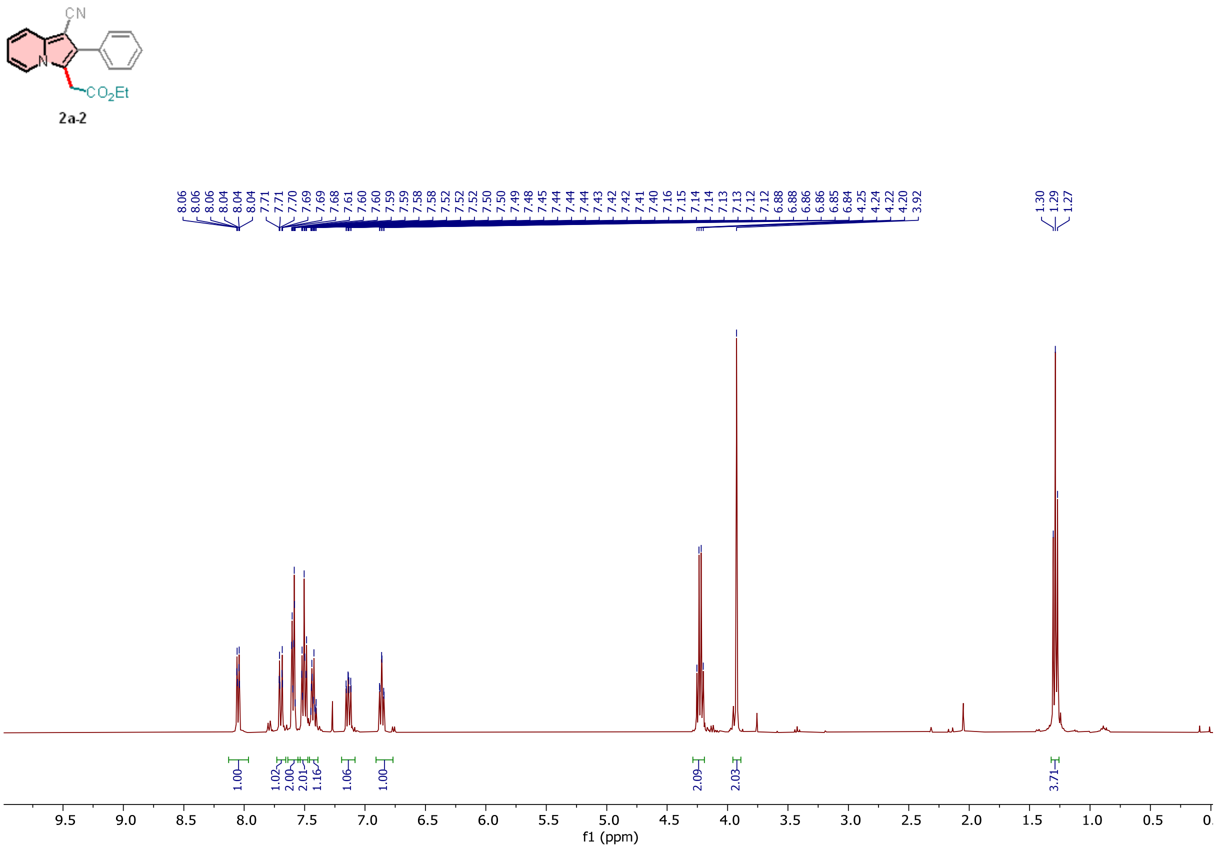

^{1}H NMR (400 MHz, CDCl$_3$) of **2a-2**.

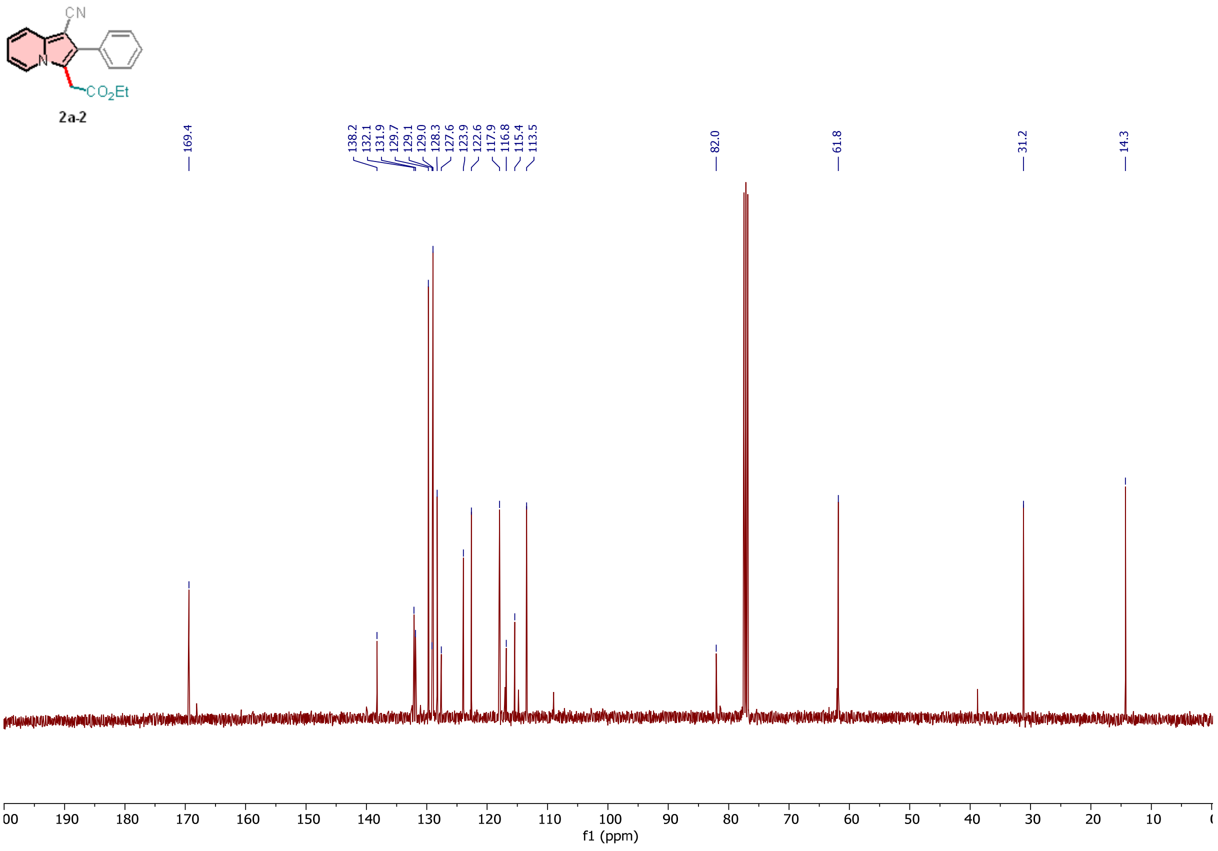

^{13}C{^{1}H} NMR (100 MHz, CDCl$_3$) of **2a-2**.

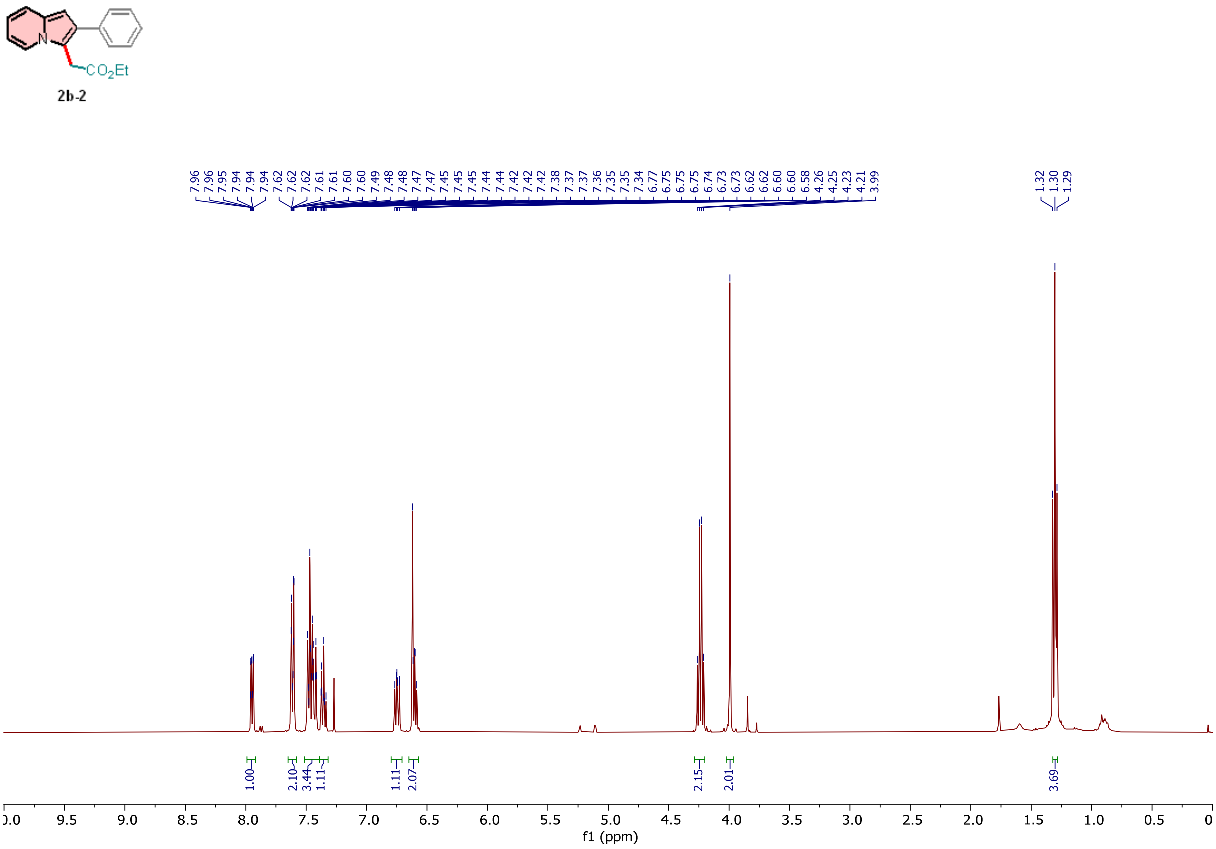

^{1}H NMR (400 MHz, $CDCl_3$) of **2b-2**.

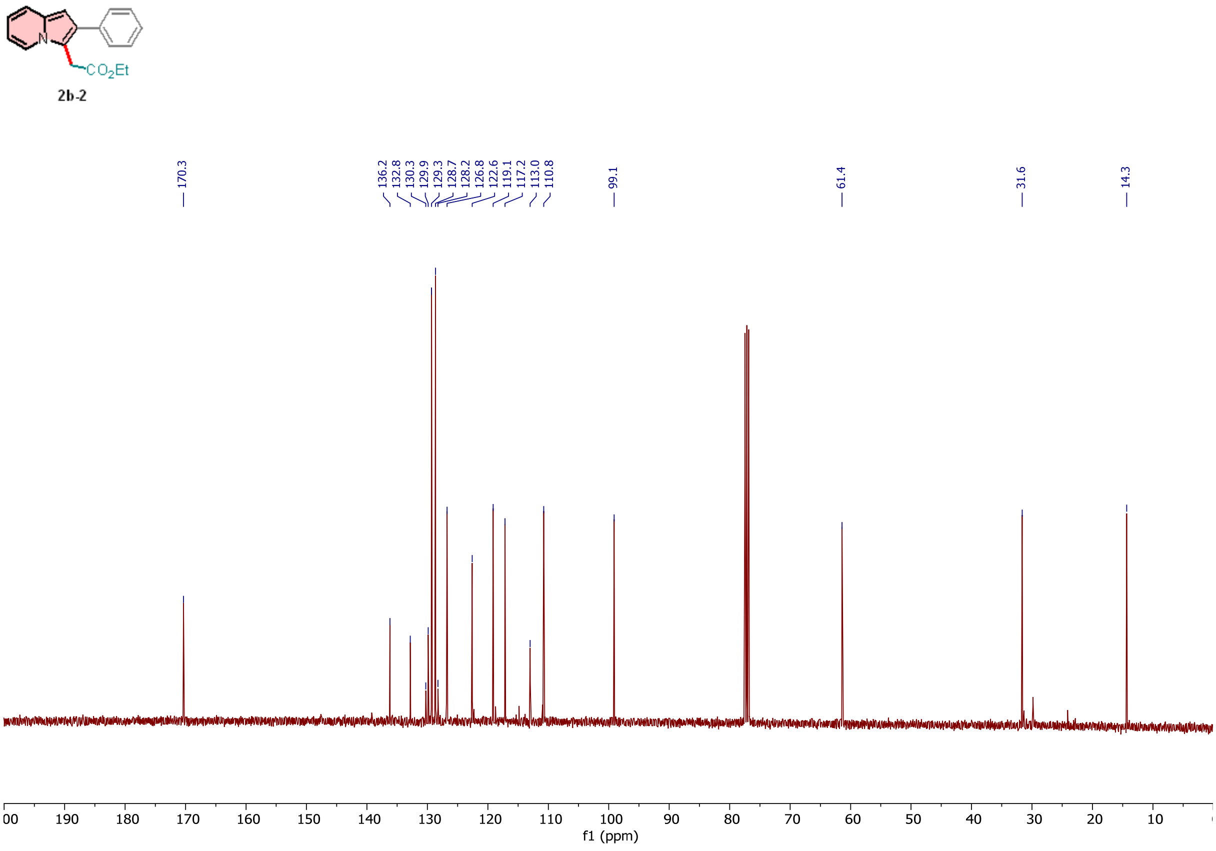

$^{13}C\{^{1}H\}$ NMR (100 MHz, $CDCl_3$) of **2b-2**.

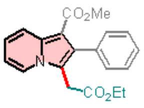

2c-2

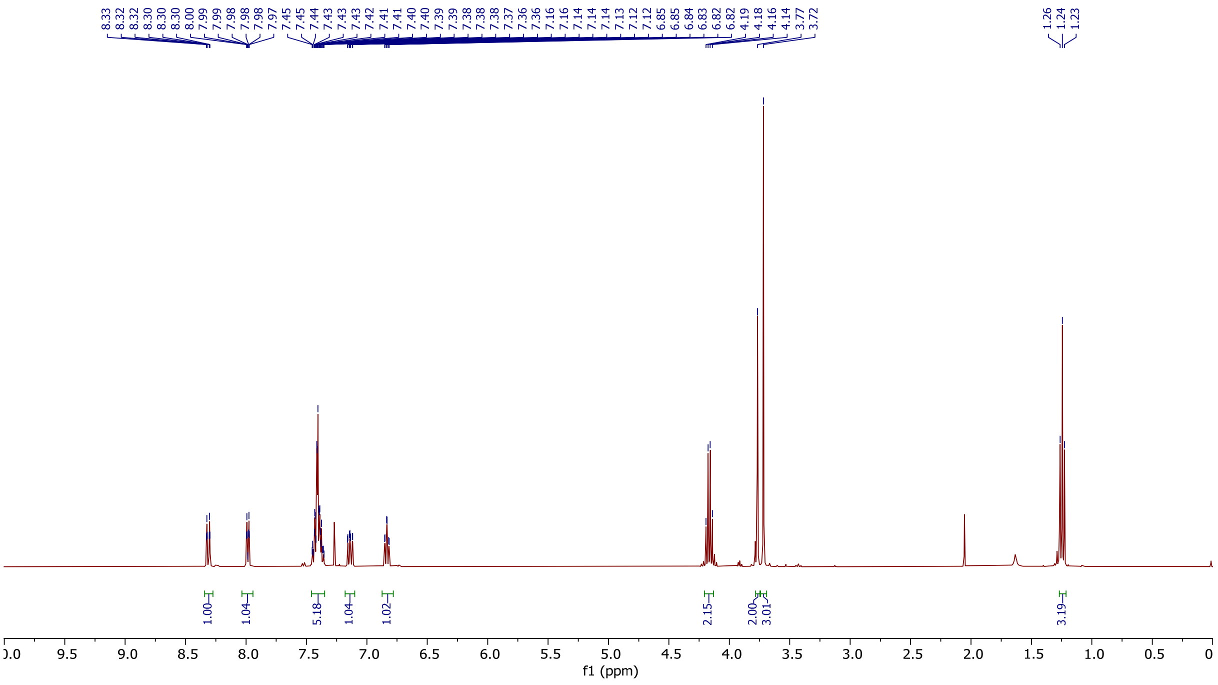

^{1}H NMR (400 MHz, $CDCl_3$) of **2c-2**.

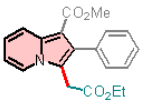

2c-2

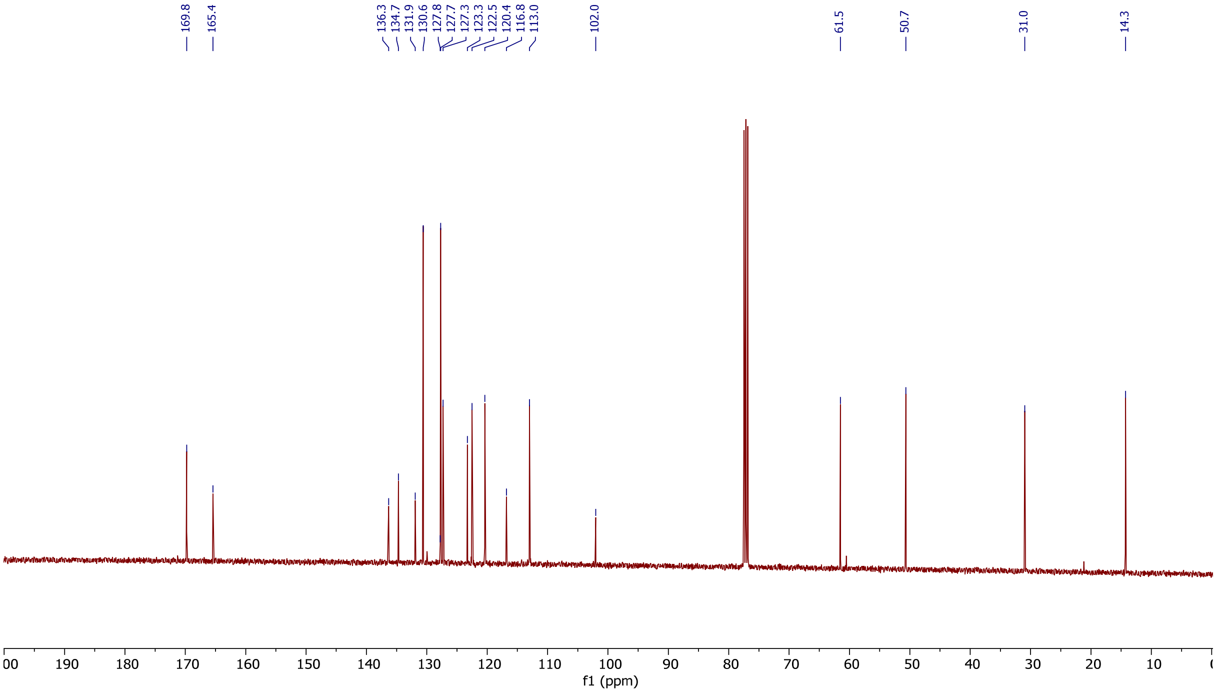

$^{13}C\{^{1}H\}$ NMR (100 MHz, $CDCl_3$) of **2c-2**.

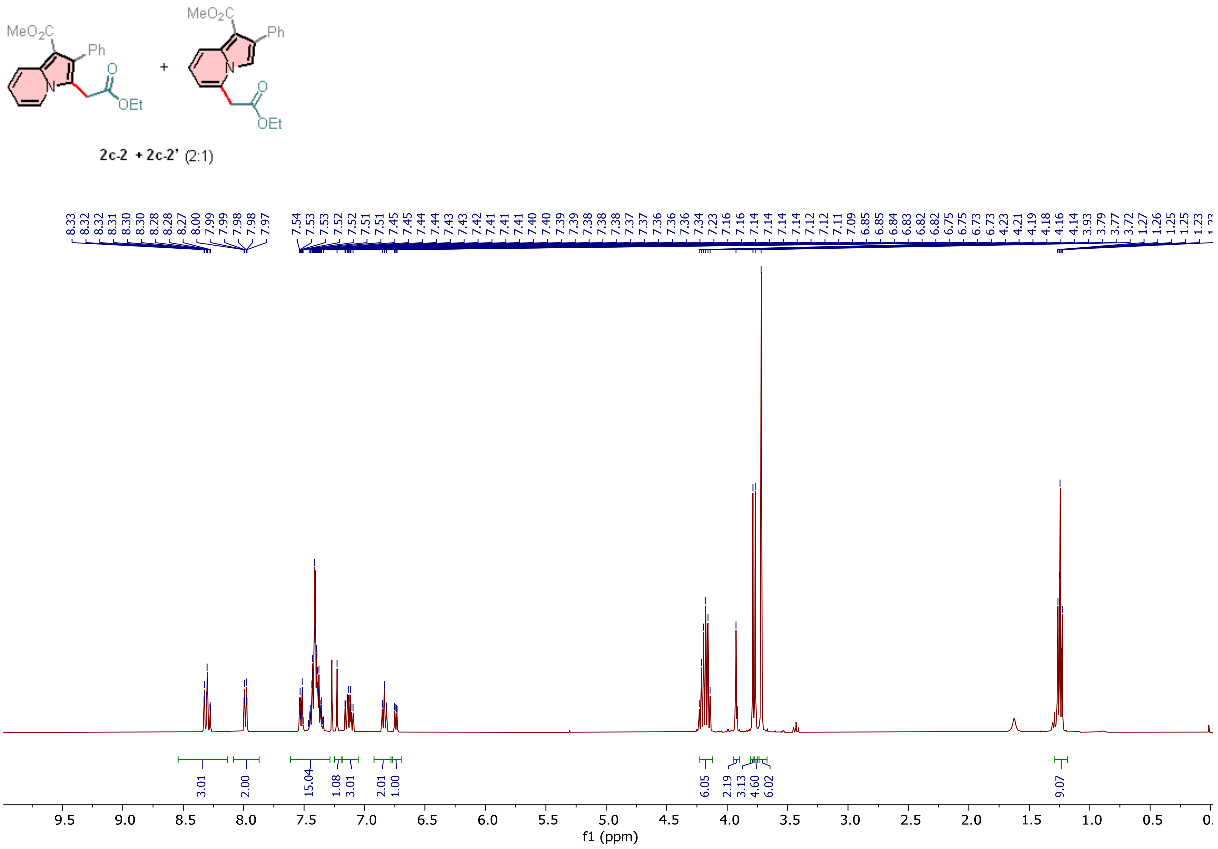

^{1}H NMR (400 MHz, $CDCl_3$) of **2c-2 + 2c-2'**.

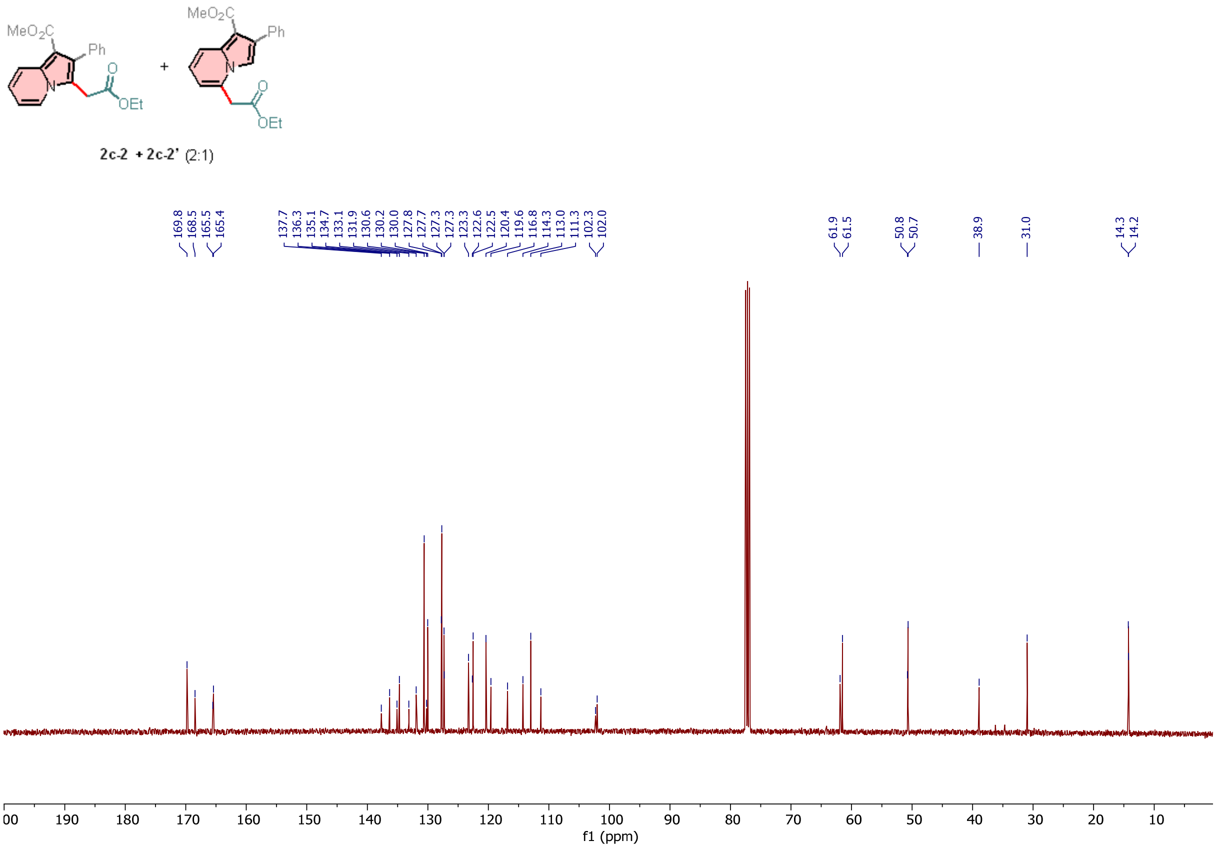

$^{13}C\{^{1}H\}$ NMR (100 MHz, $CDCl_3$) of **2c-2 + 2c-2'**.

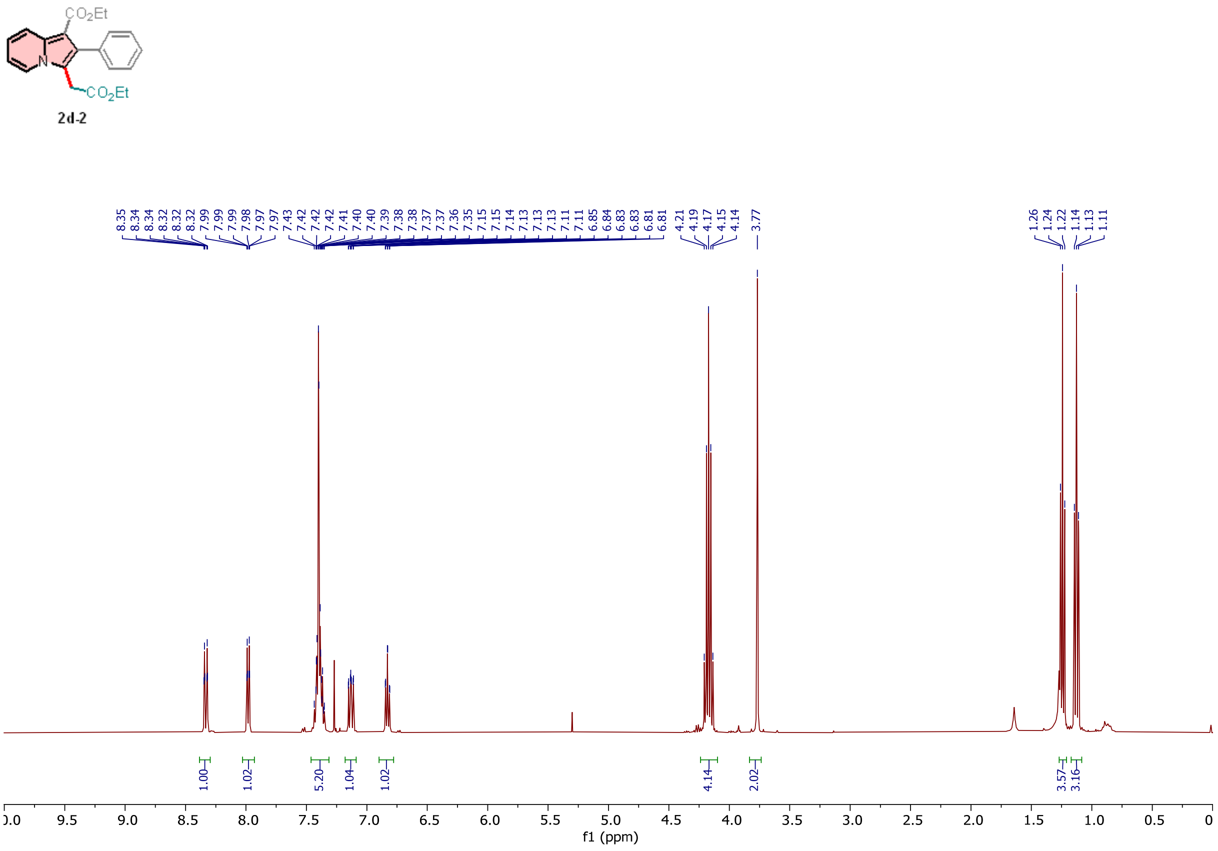

^{1}H NMR (400 MHz, $CDCl_3$) of **2d-2**.

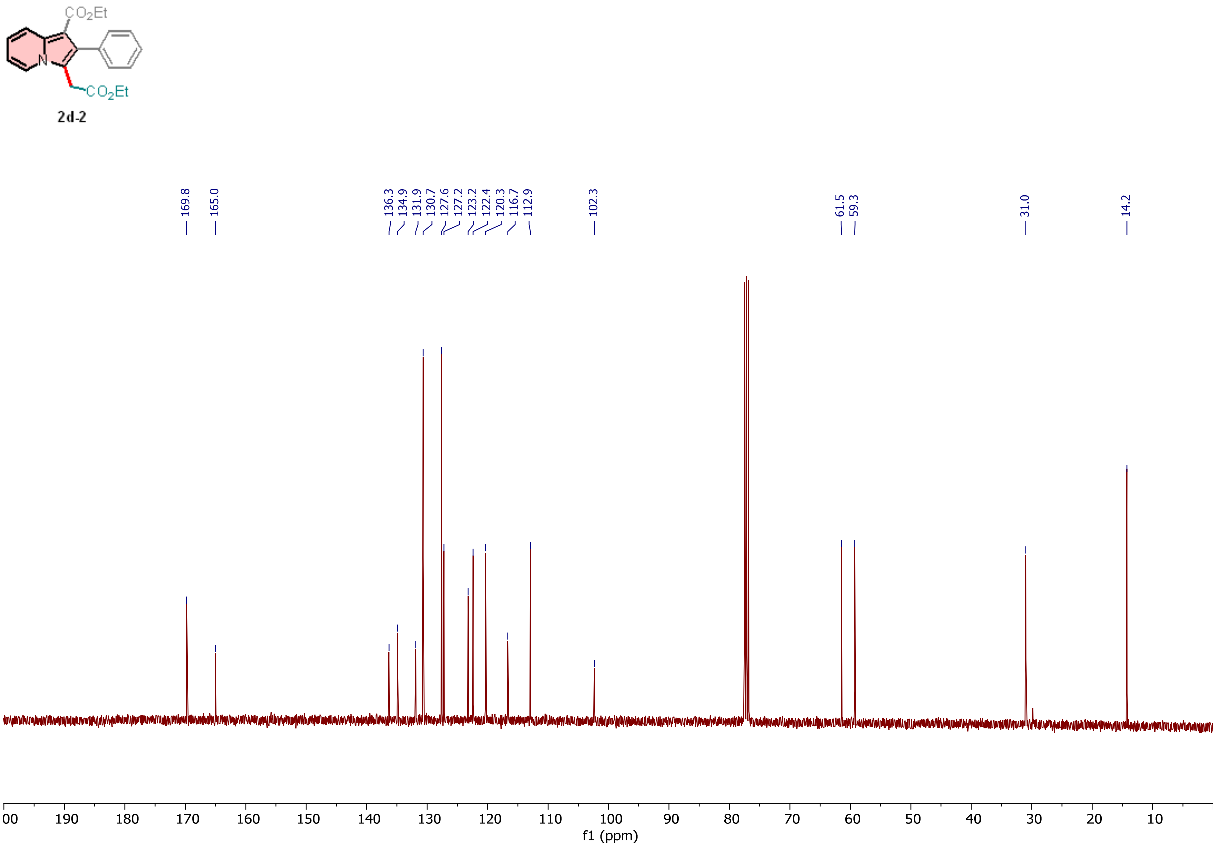

$^{13}C\{^{1}H\}$ NMR (100 MHz, $CDCl_3$) of **2d-2**.

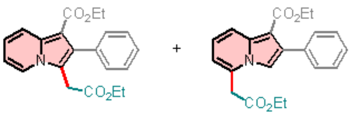

2X + 2X' (2:1)

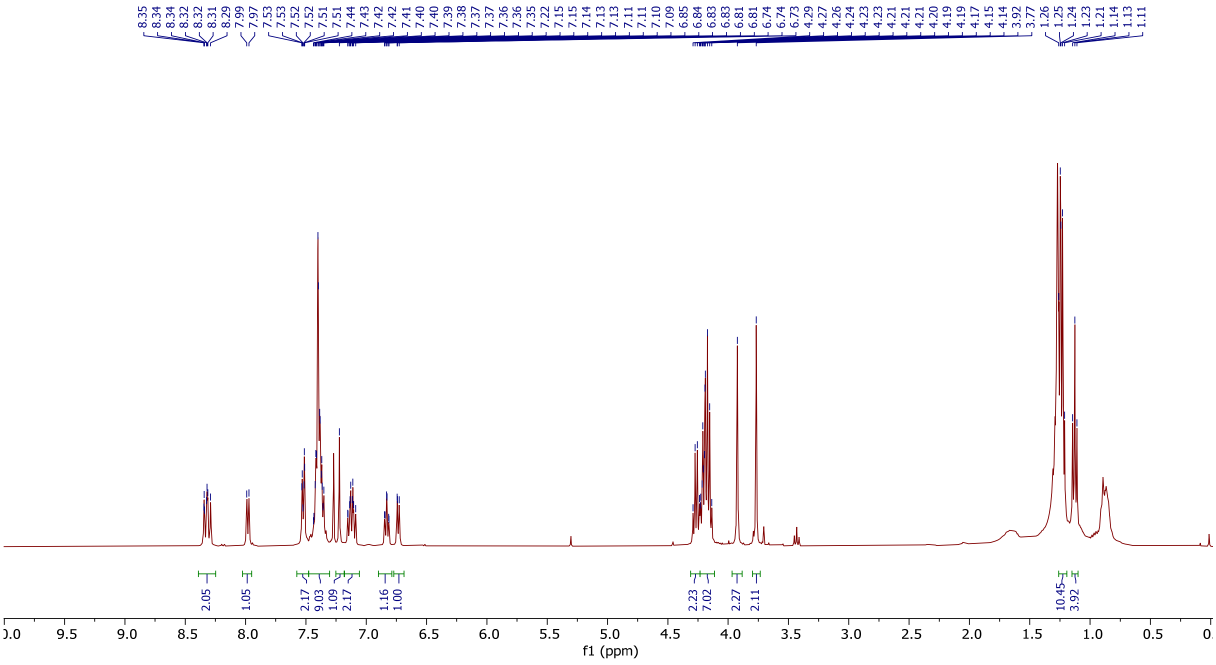

^{1}H NMR (400 MHz, $CDCl_3$) of **2d-2 + 2d-2'**.

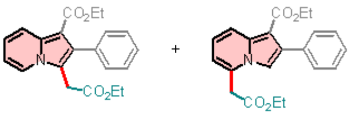

2d-2 + 2d-2' (2:1)

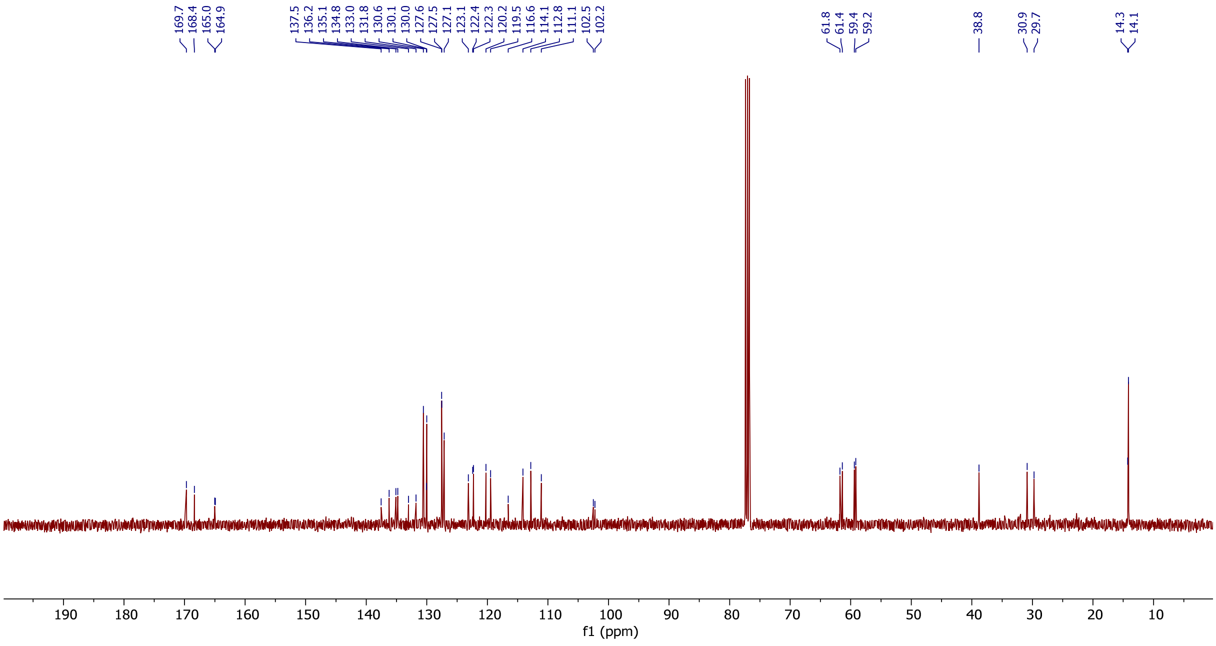

$^{13}C\{^1H\}$ NMR (100 MHz, $CDCl_3$) of **2d-2 + 2d-2'**.

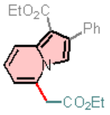

2d-2'

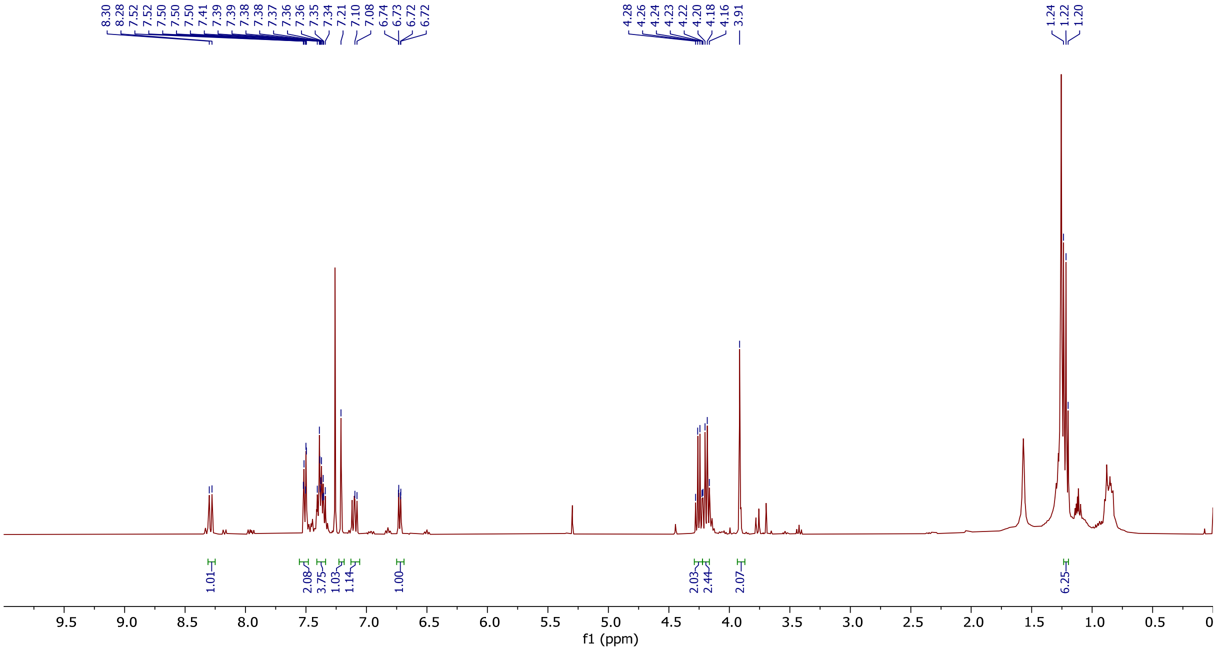

^{1}H NMR (400 MHz, $CDCl_3$) of **2d-2'**.

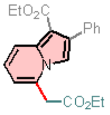

2d-2'

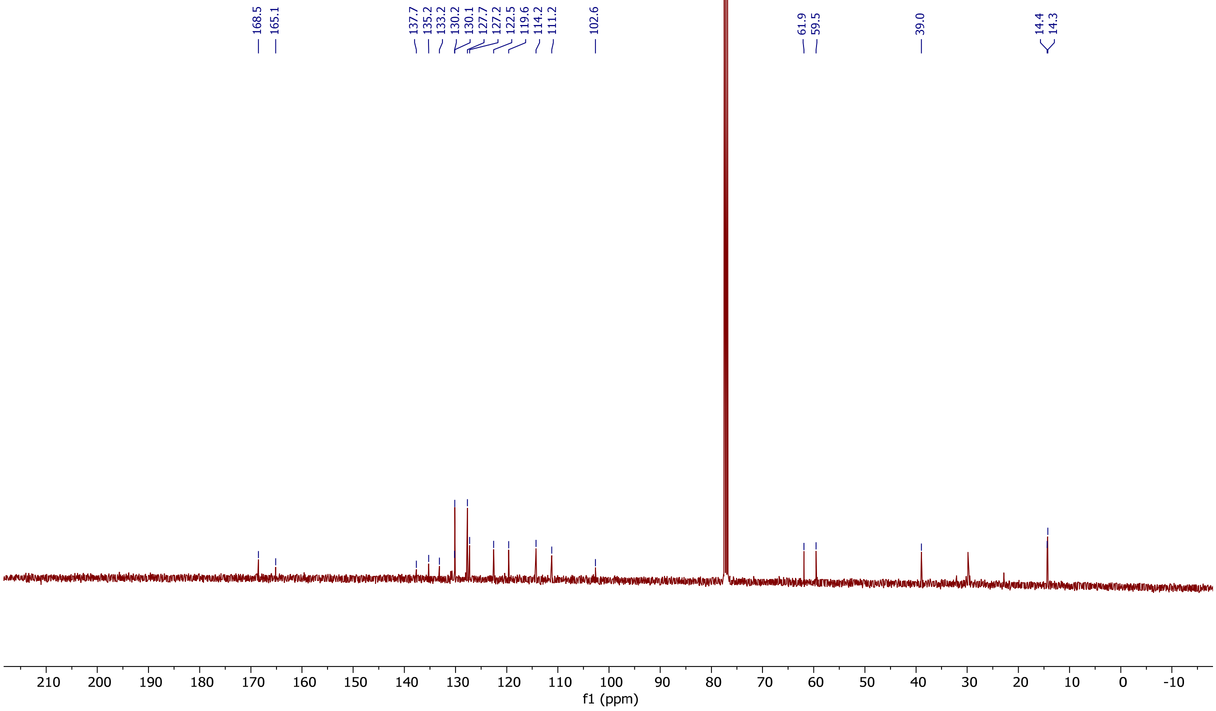

^{13}C{^{1}H} NMR (100 MHz, $CDCl_3$) of **2d-2'**.

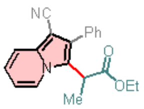

2a-3

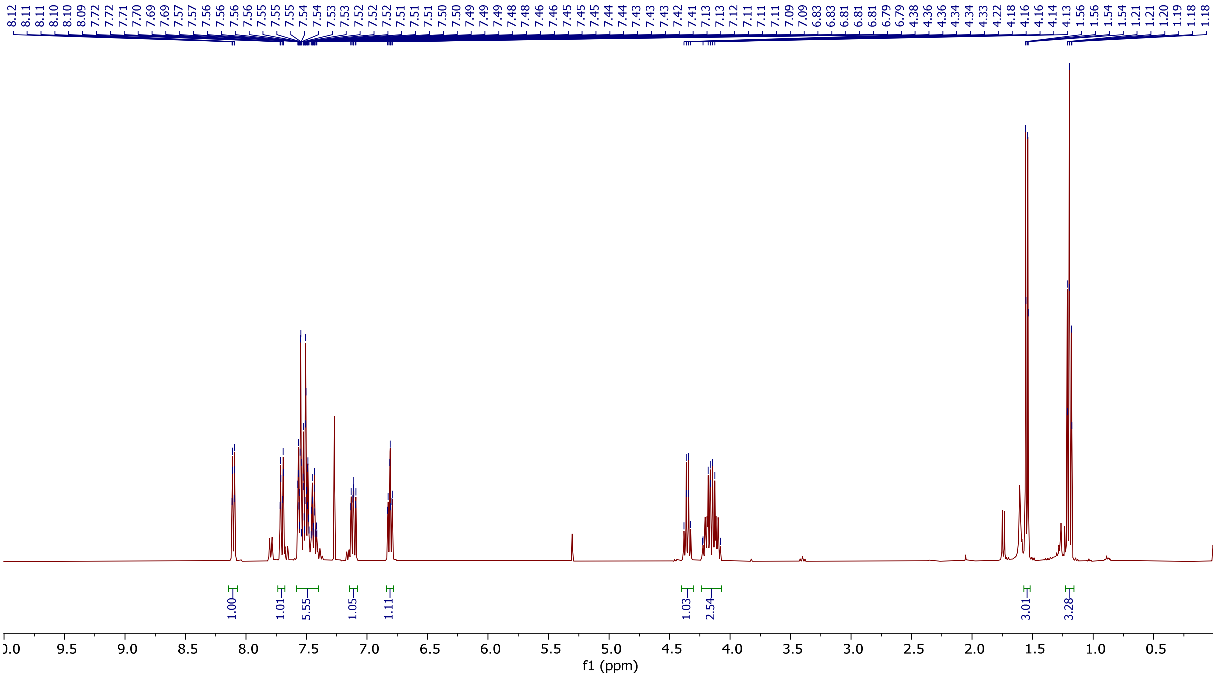

^{1}H NMR (400 MHz, $CDCl_3$) of **2a-3**.

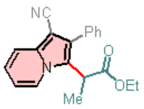

2a-3

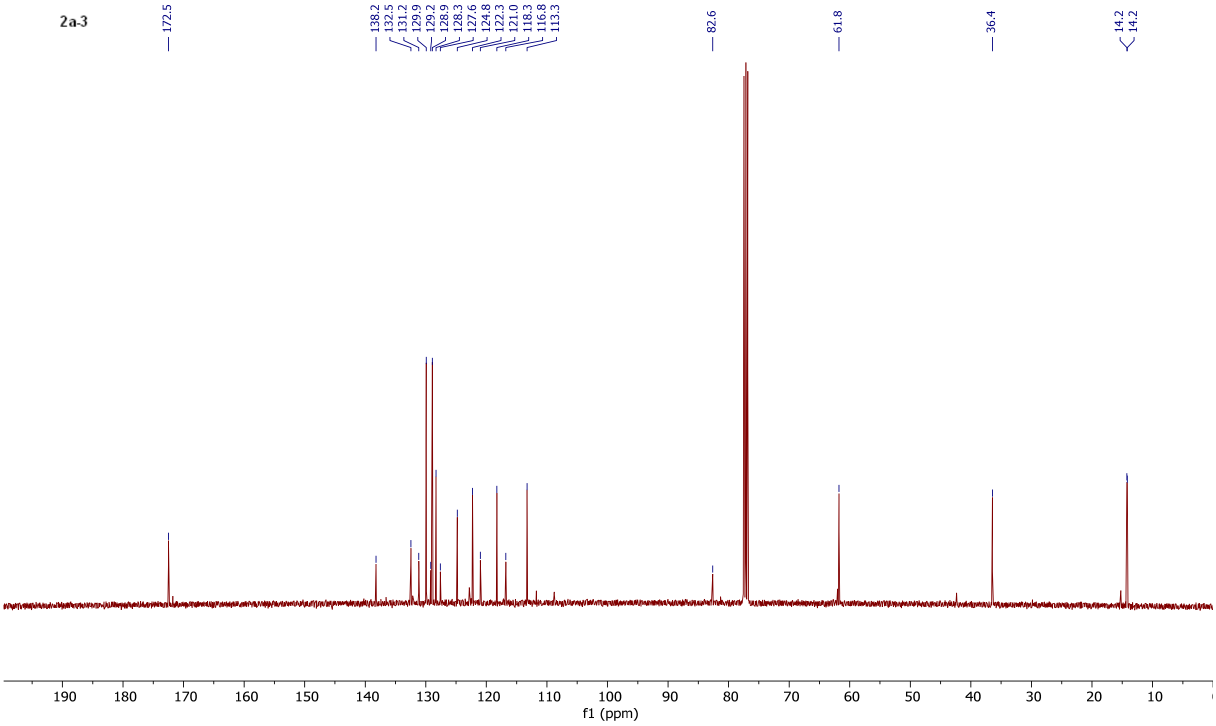

$^{13}C\{^{1}H\}$ NMR (100 MHz, $CDCl_3$) of **2a-3**.

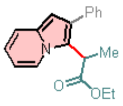

2b-3

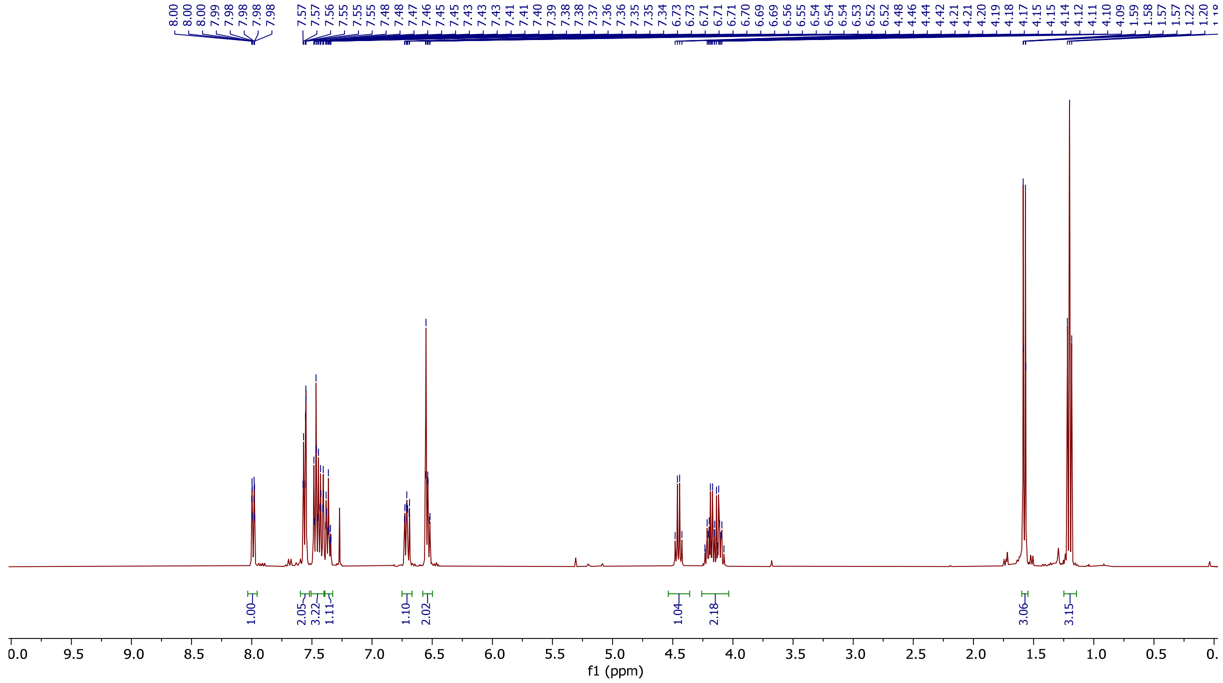

^{1}H NMR (400 MHz, $CDCl_3$) of **2b-3**.

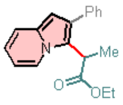

2b-3

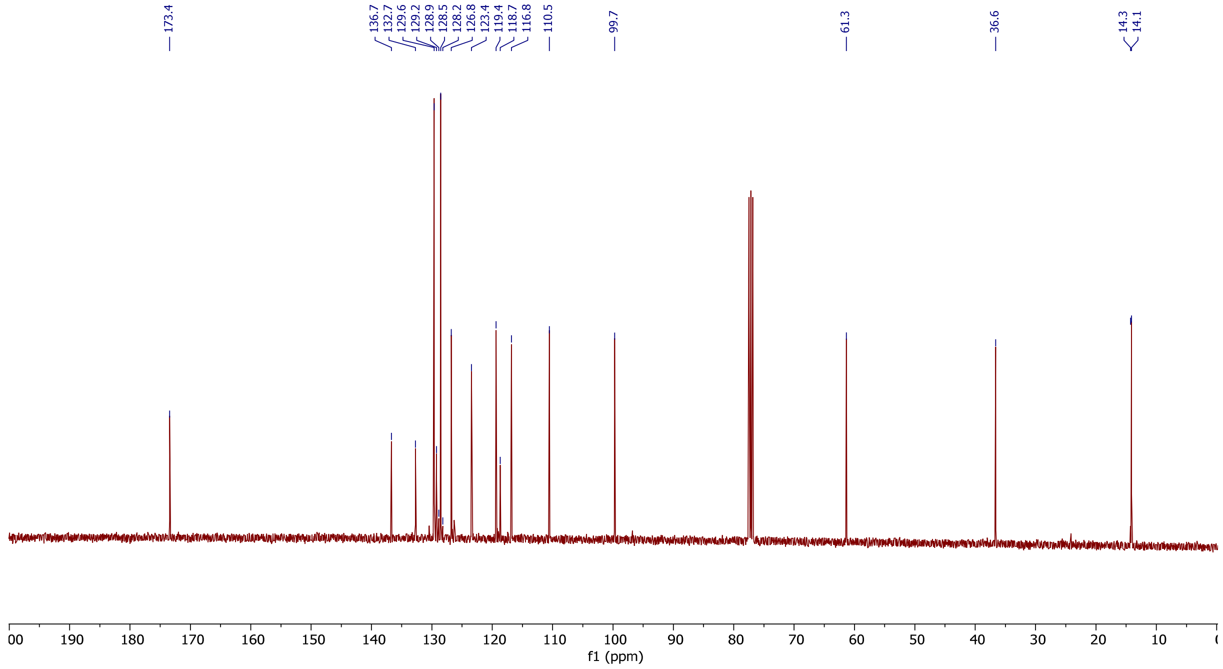

$^{13}C\{^{1}H\}$ NMR (100 MHz, $CDCl_3$) of **2b-3**.

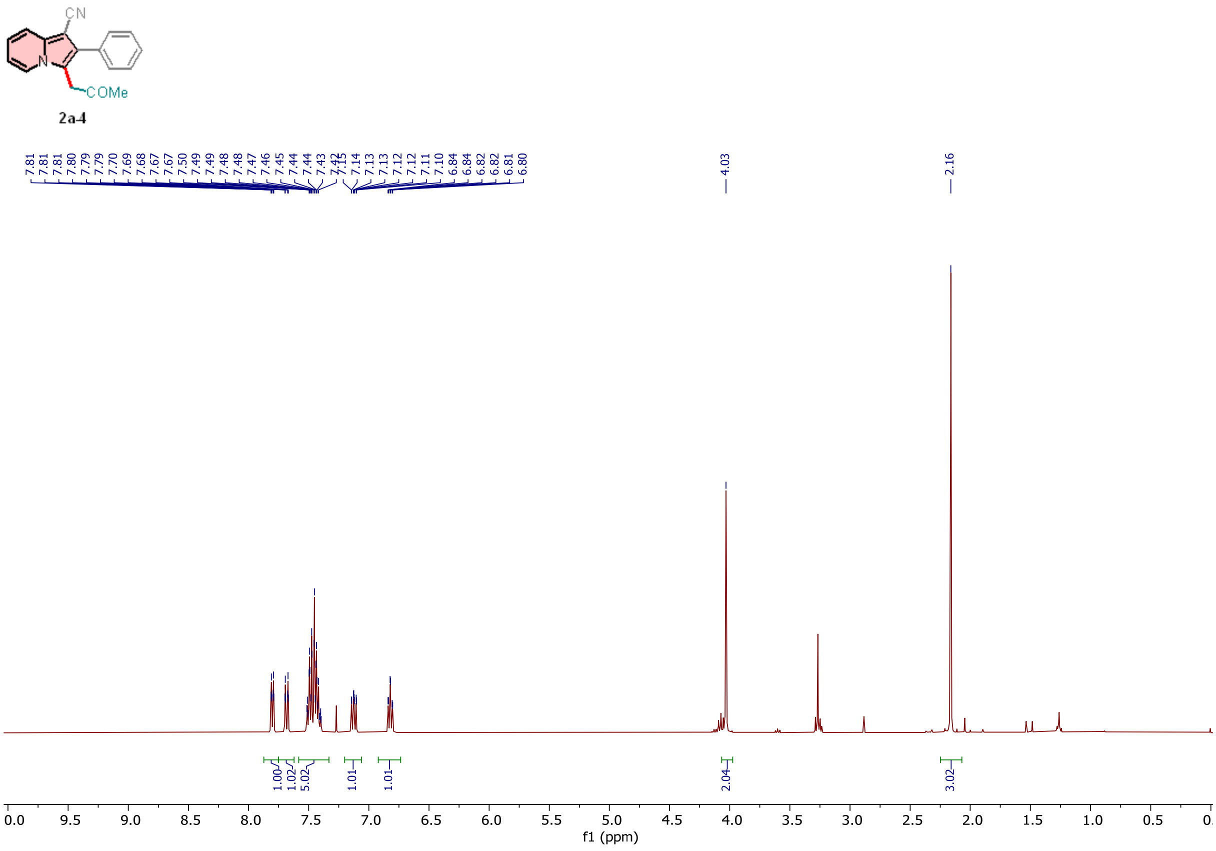

^1H NMR (400 MHz, $CDCl_3$) of **2a-4**.

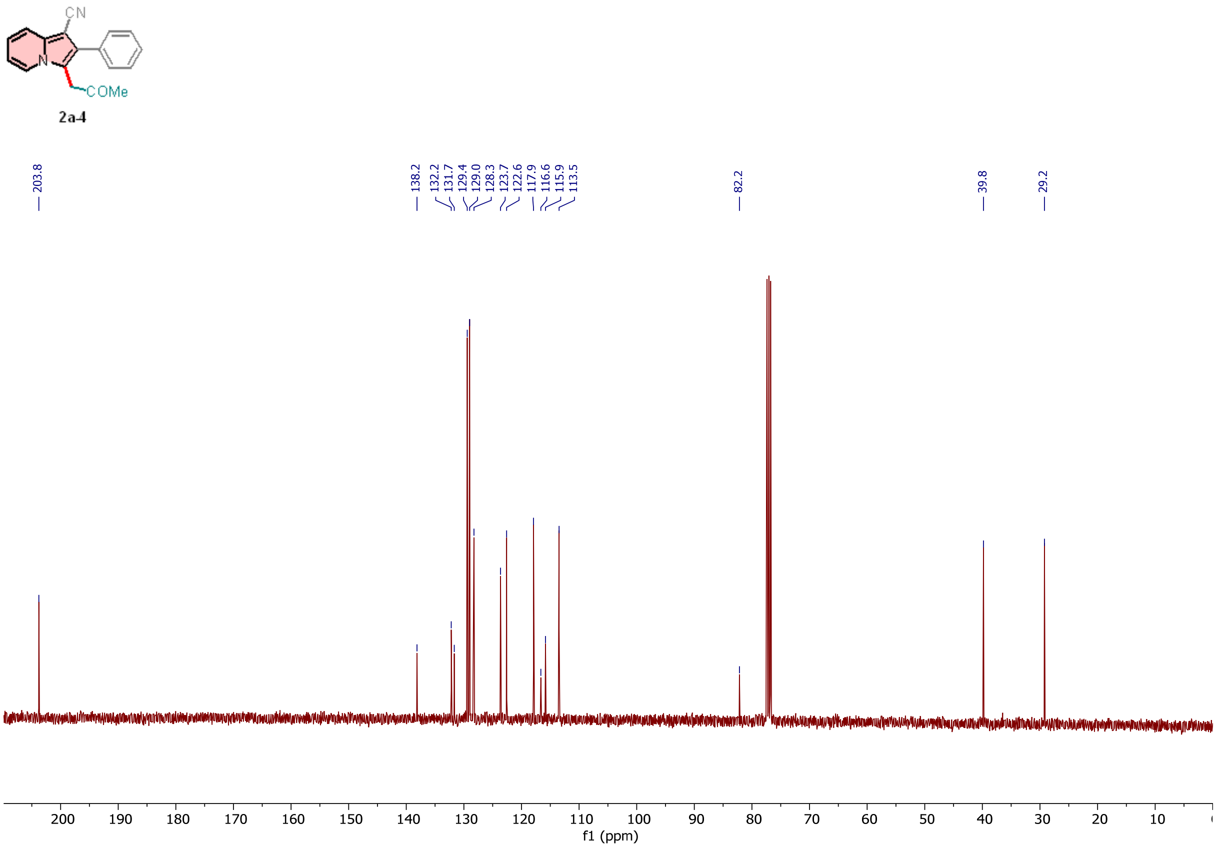

$^{13}C\{^1H\}$ NMR (100 MHz, $CDCl_3$) of **2a-4.**

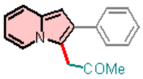

2b-4

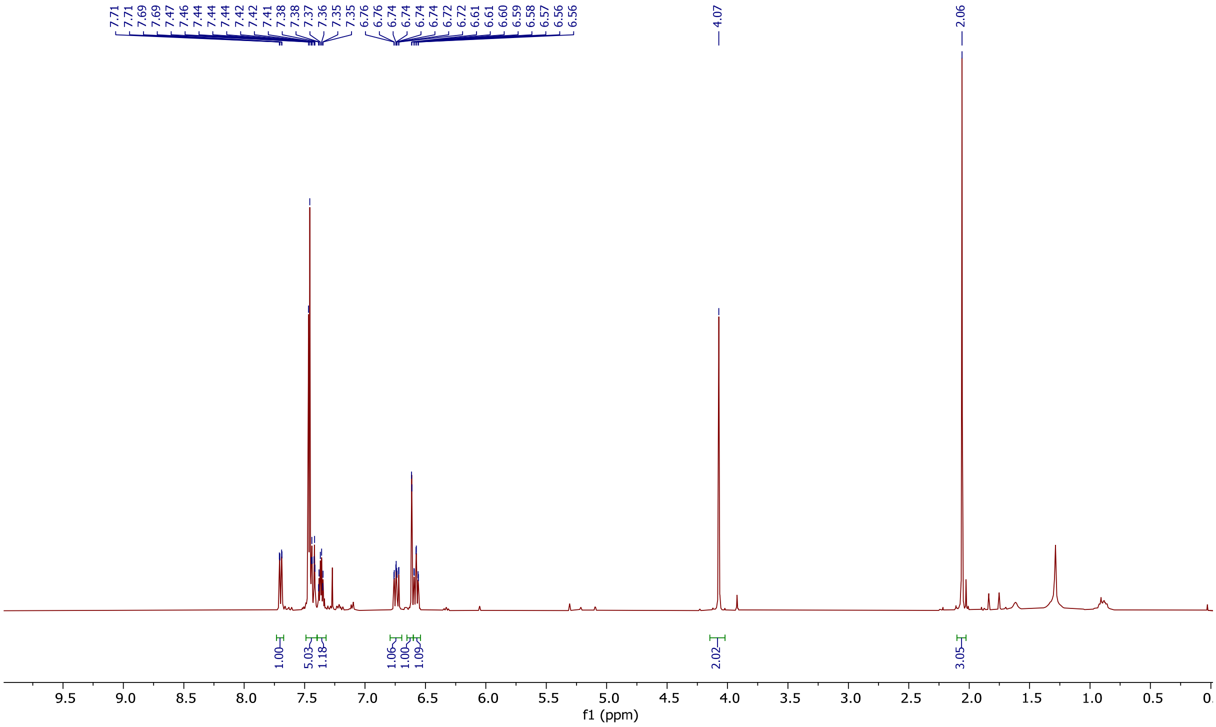

^{1}H NMR (400 MHz, $CDCl_3$) of **2b-4**.

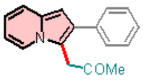

2b-4

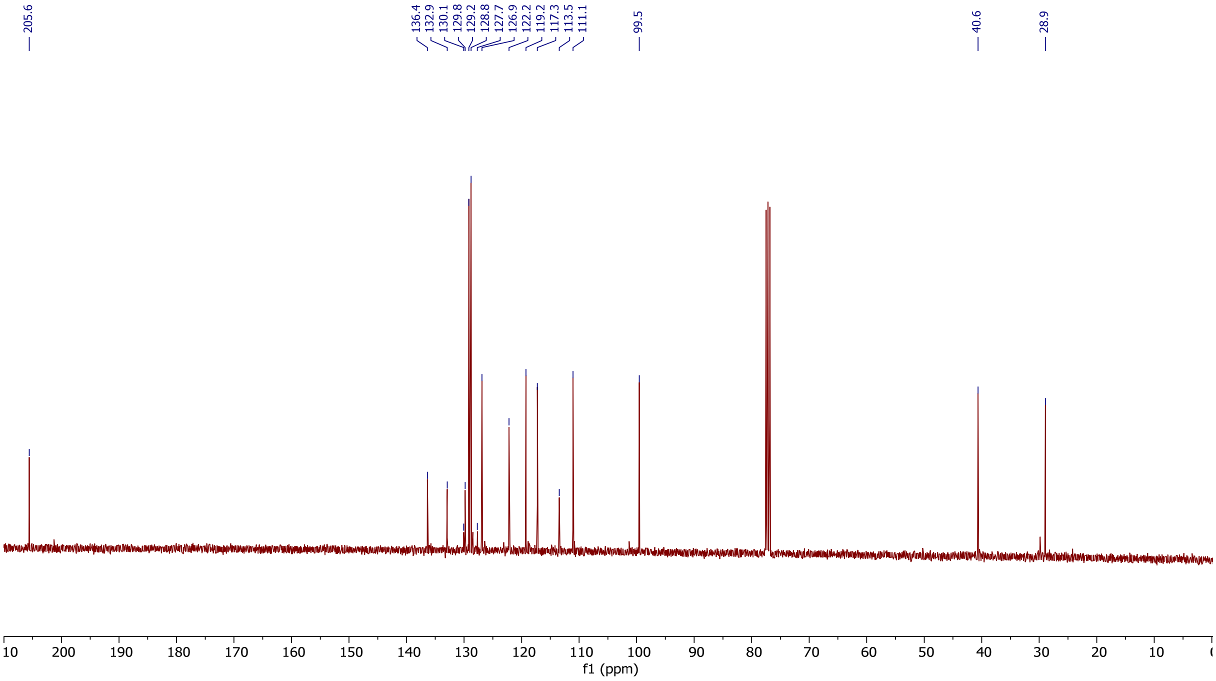

$^{13}C\{^{1}H\}$ NMR (100 MHz, $CDCl_3$) of **2b-4**.

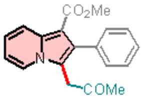

2c-4

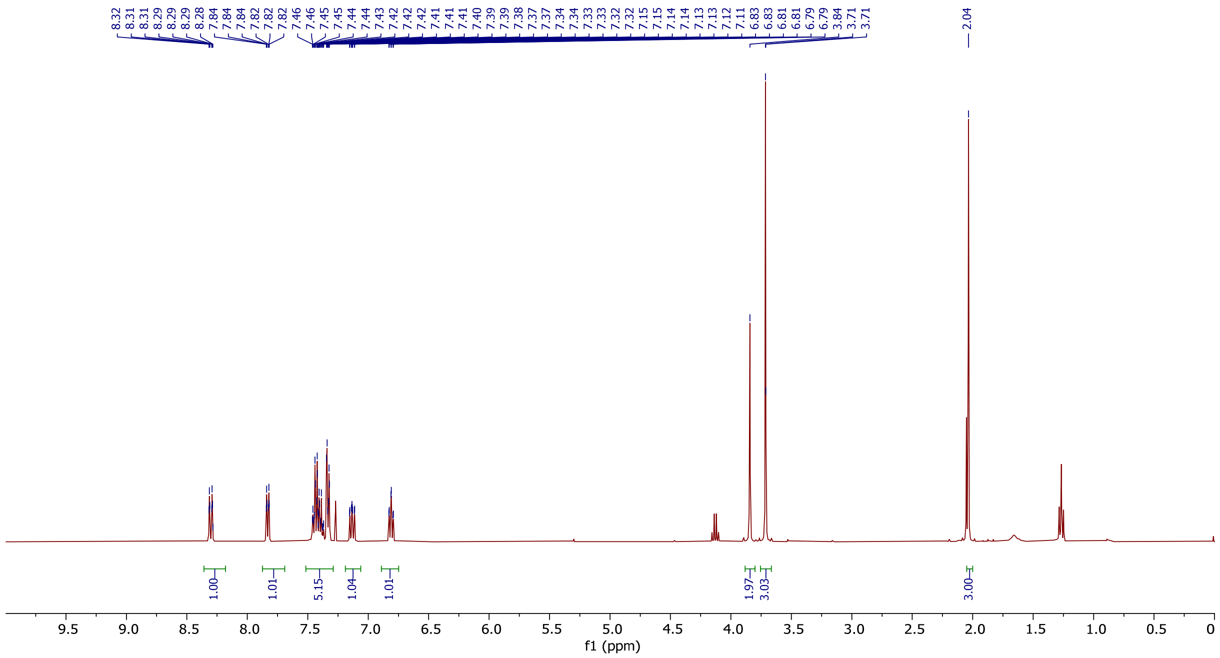

^{1}H NMR (400 MHz, $CDCl_3$) of **2c-4**.

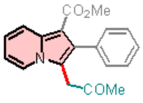

2c-4

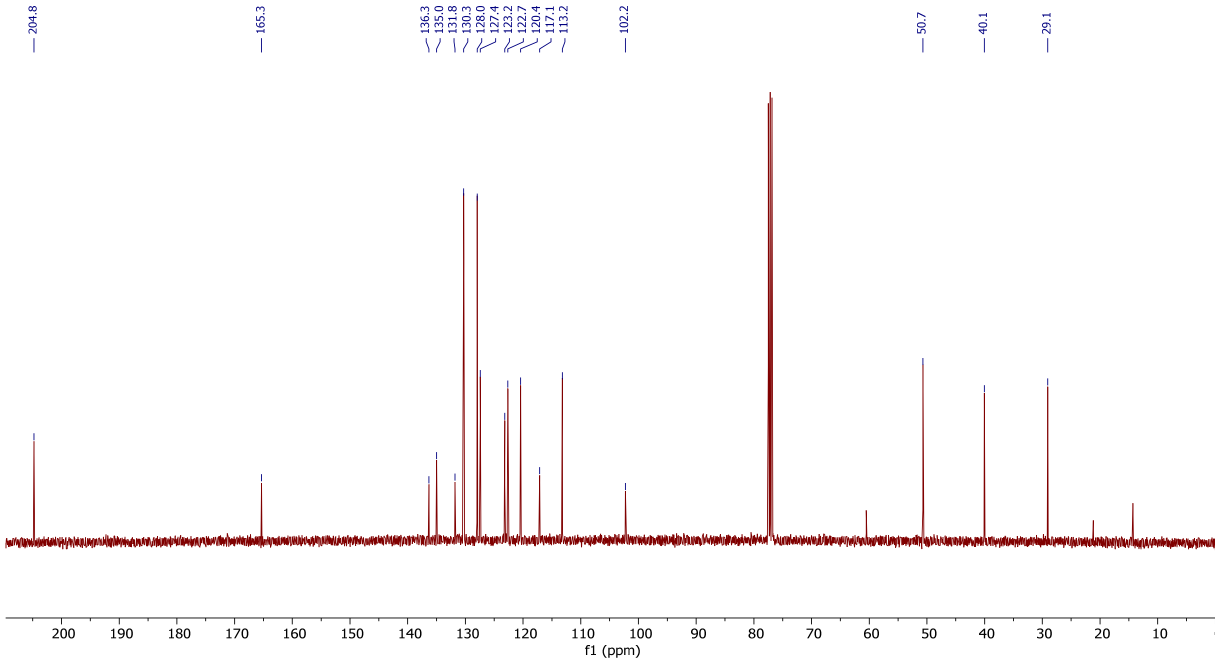

^{13}C{^{1}H} NMR (100 MHz, $CDCl_3$) of **2c-4**.

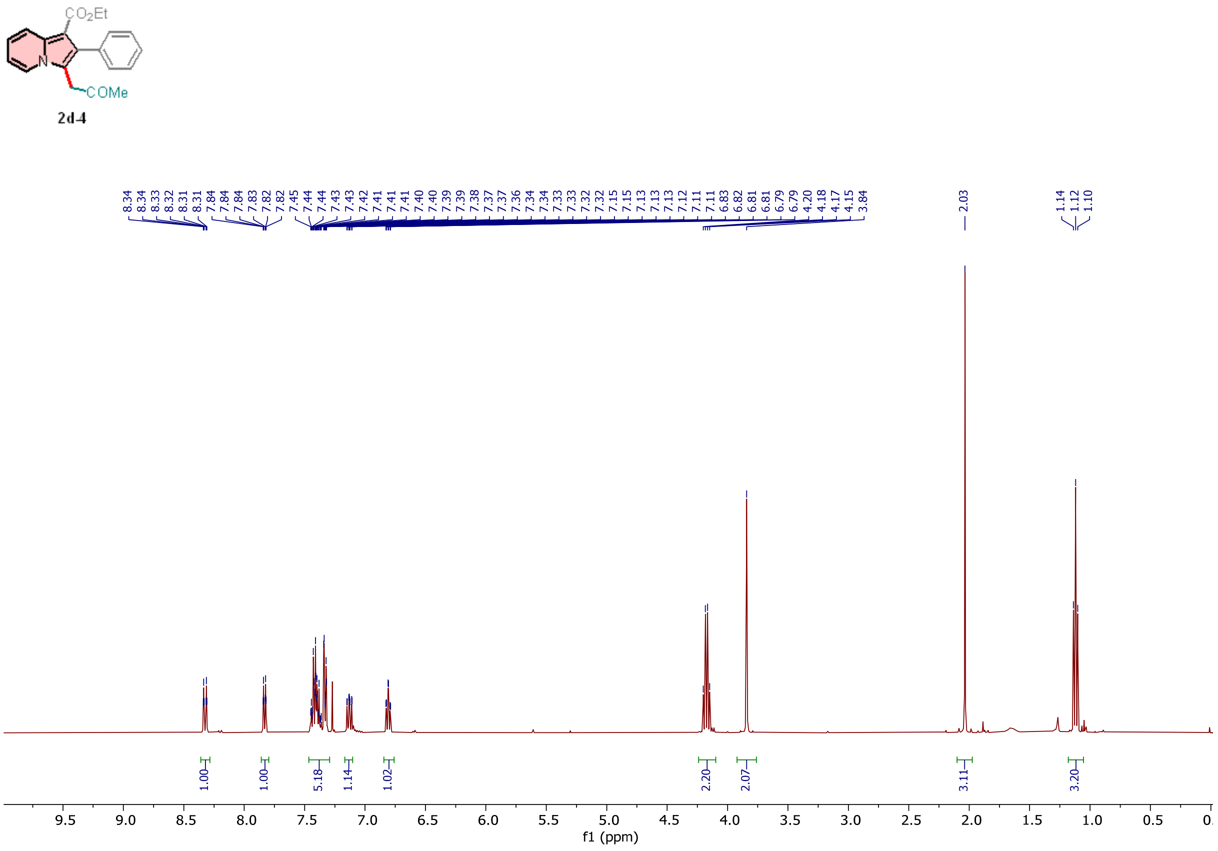

^1H NMR (400 MHz, $CDCl_3$) of **2d-4**.

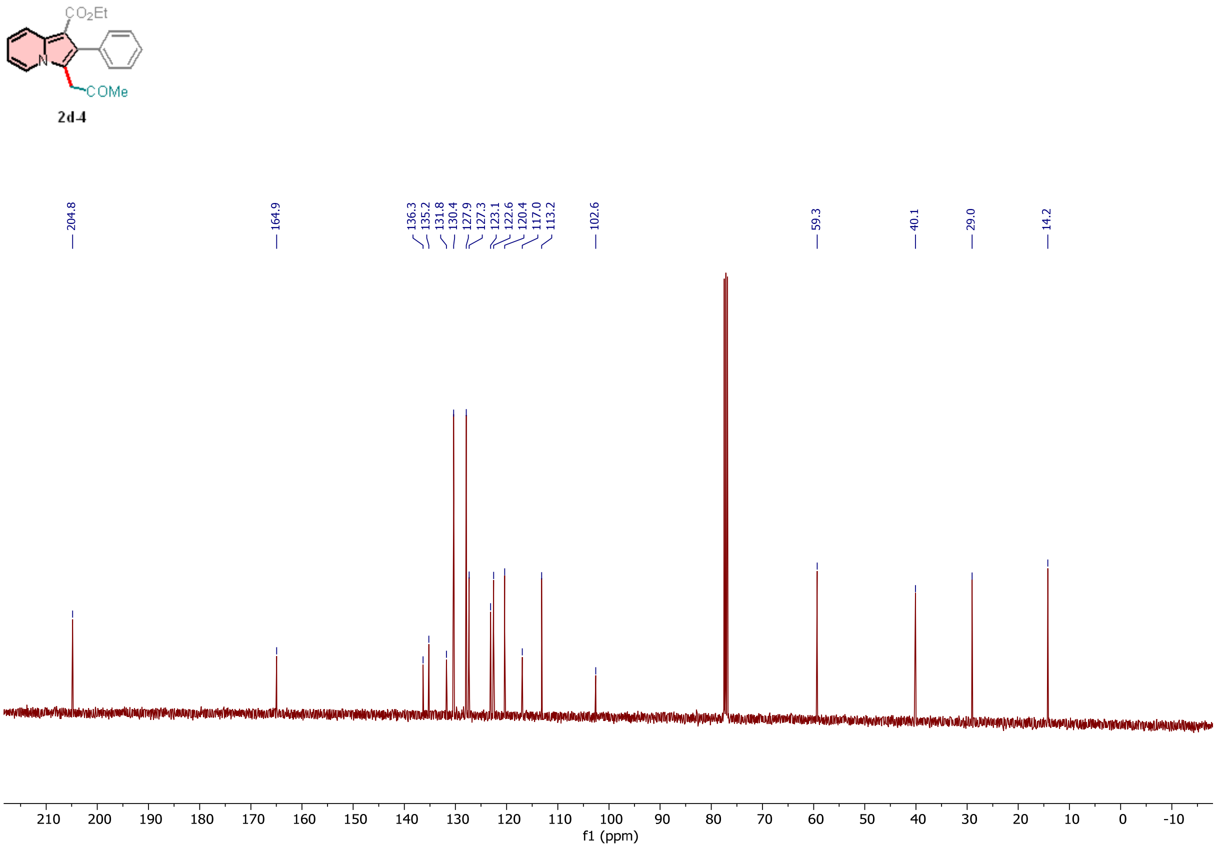

$^{13}C\{^1H\}$ NMR (100 MHz, $CDCl_3$) of **2d-4**.

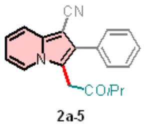

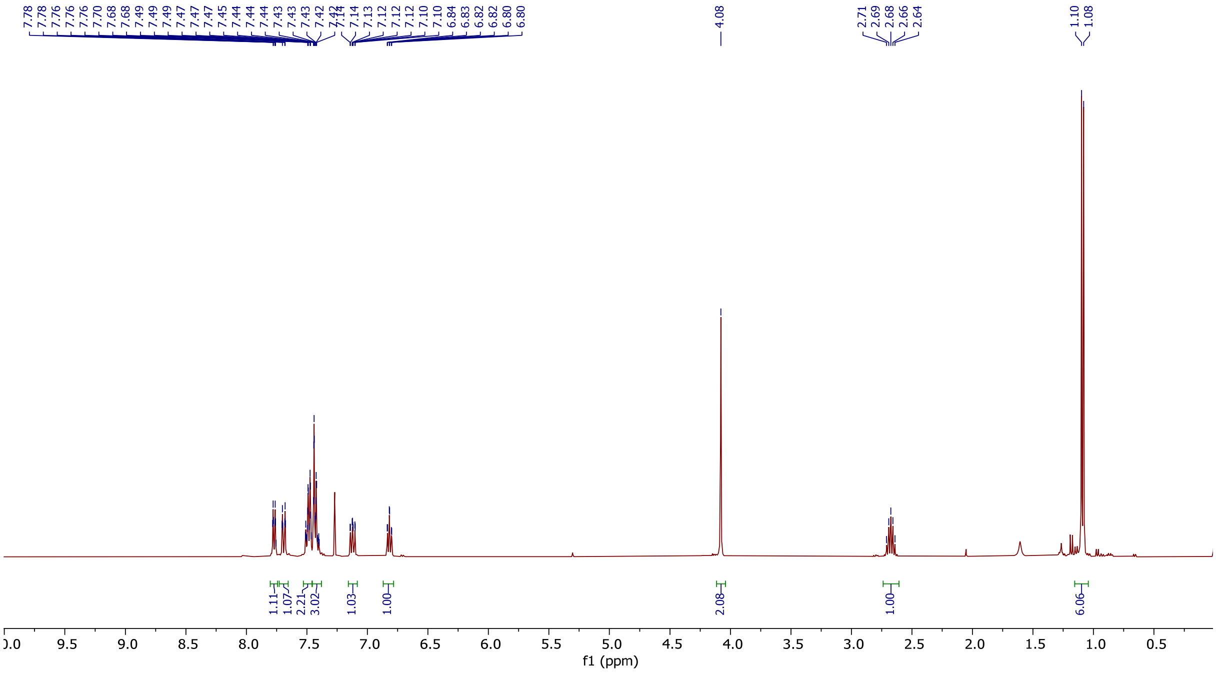

^{1}H NMR (400 MHz, $CDCl_3$) of **2a-5**.

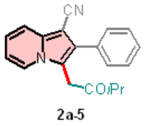

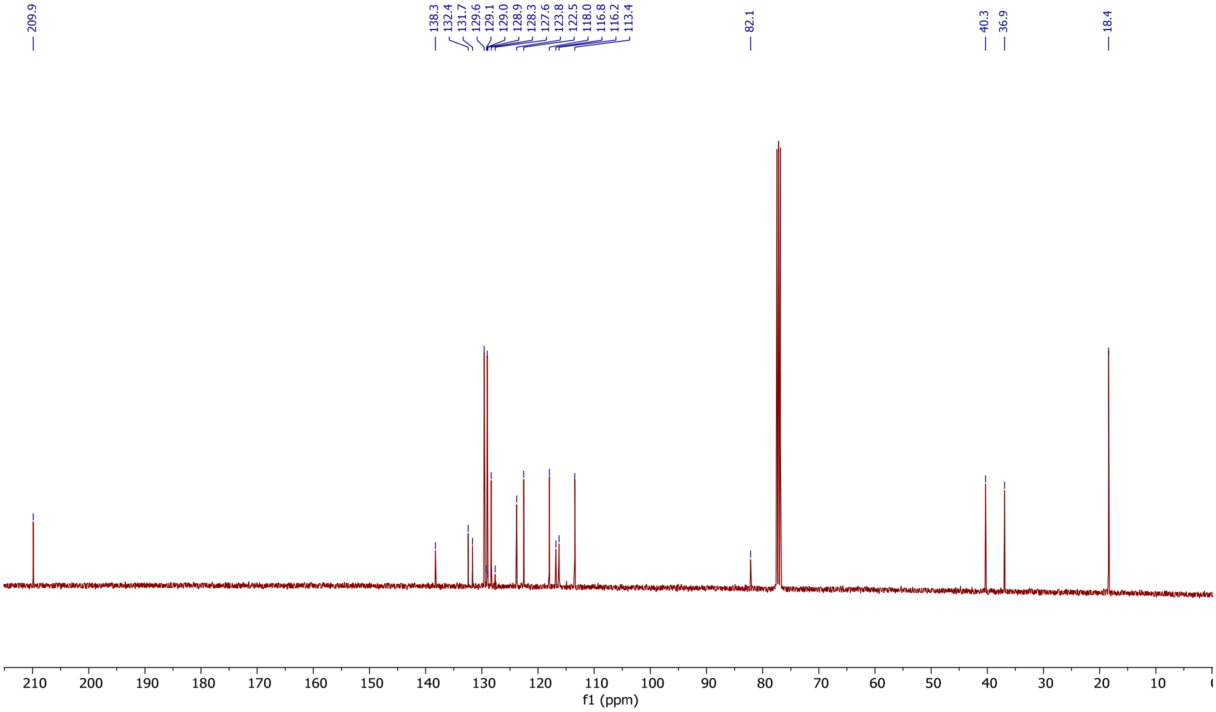

$^{13}C\{^1H\}$ NMR (100 MHz, $CDCl_3$) of **2a-5**.

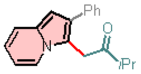

2b-5

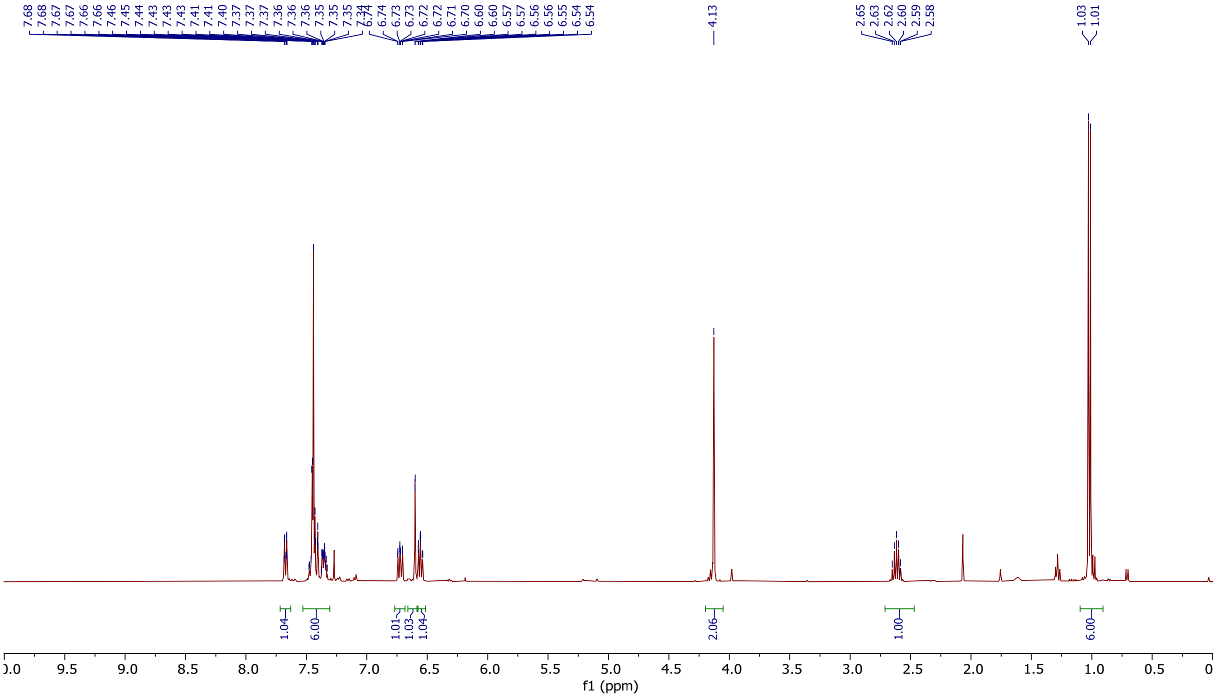

^{1}H NMR (400 MHz, $CDCl_3$) of **2b-5**.

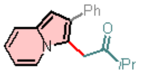

2b-5

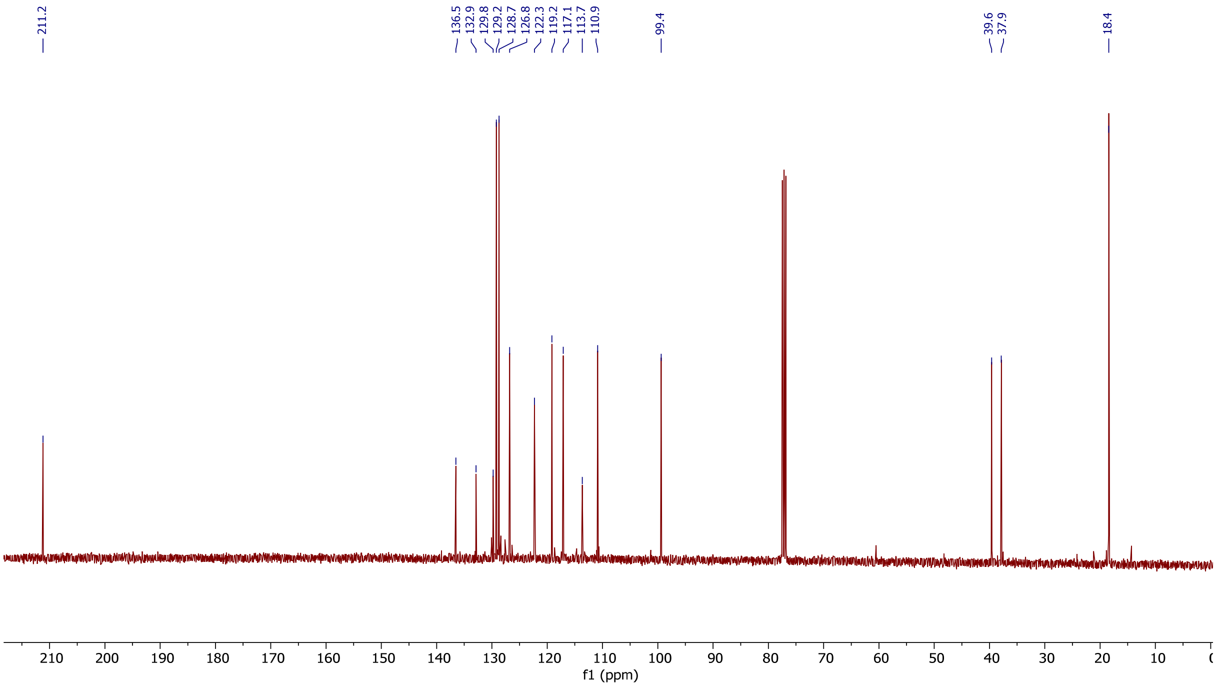

$^{13}C\{^{1}H\}$ NMR (100 MHz, $CDCl_3$) of **2b-5**.

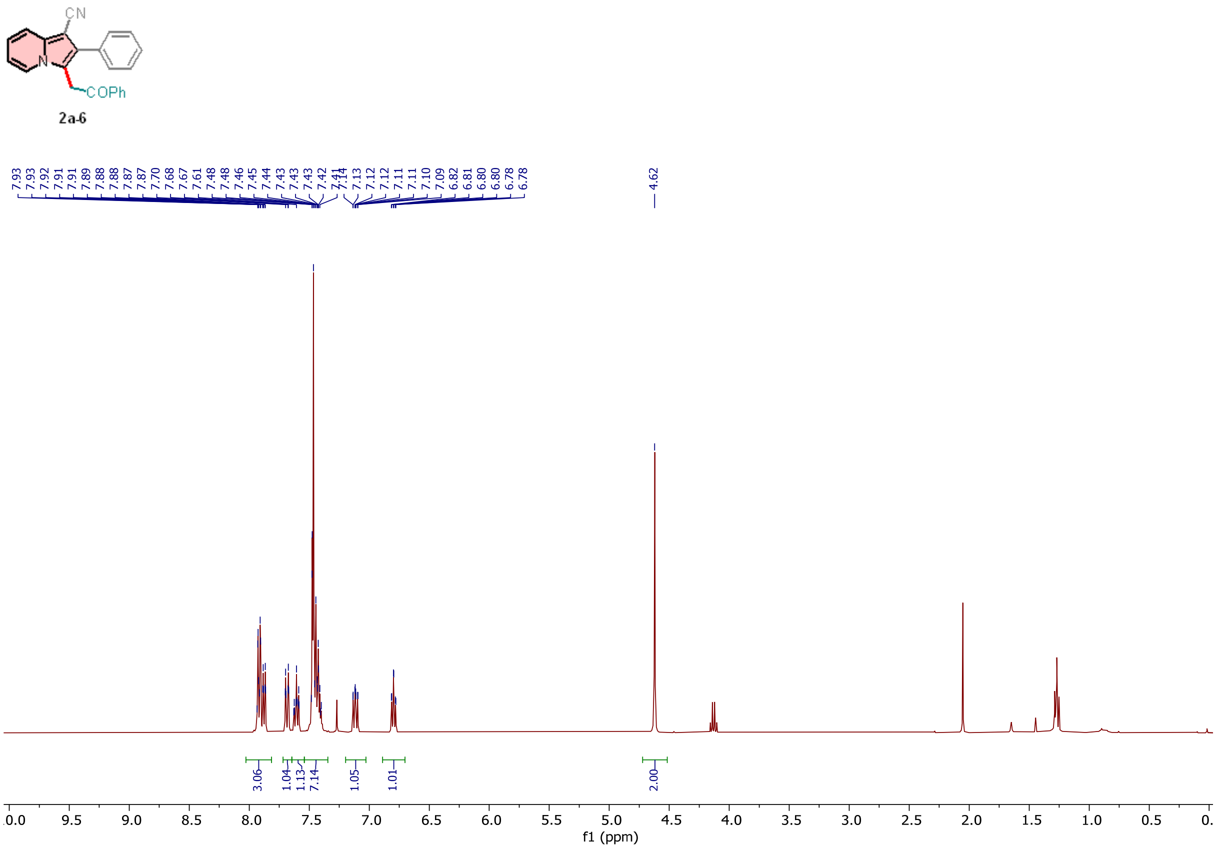

^{1}H NMR (400 MHz, $CDCl_3$) of **2a-6**.

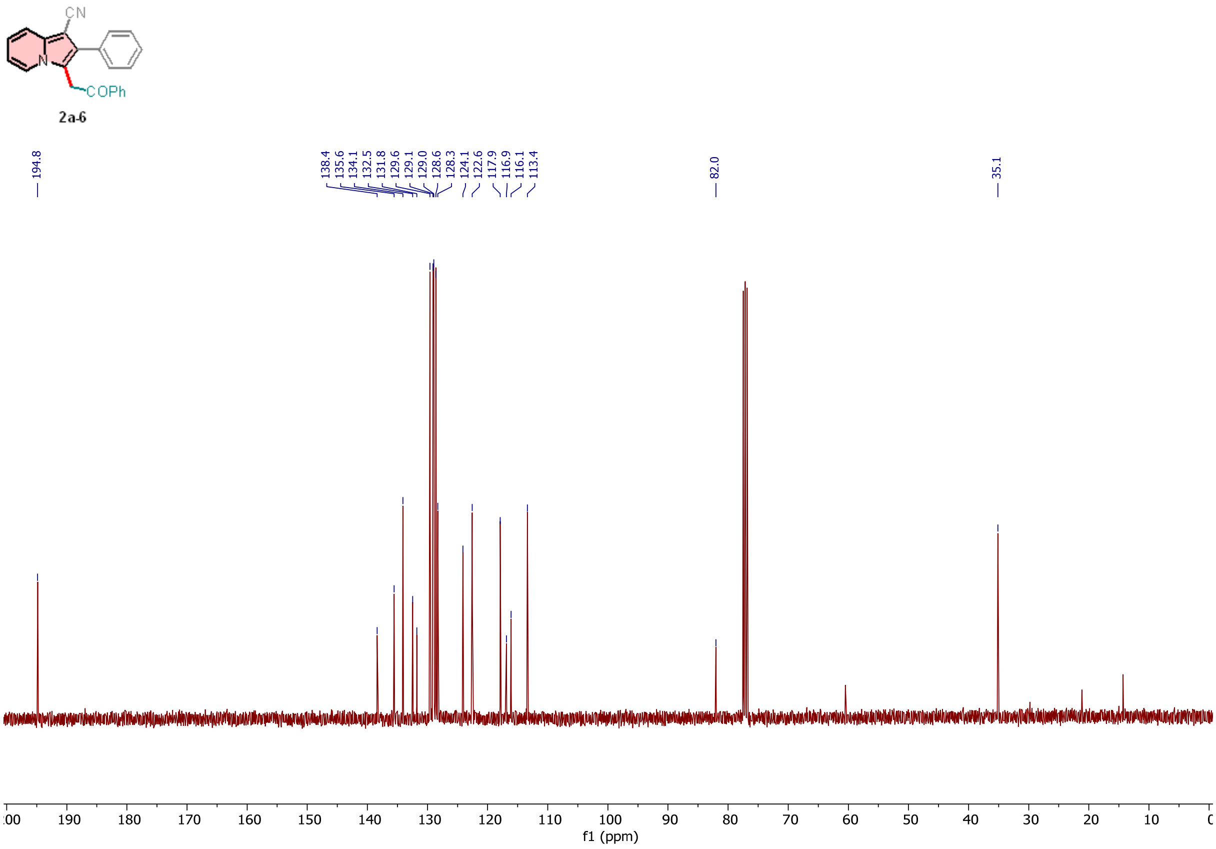

$^{13}C\{^1H\}$ NMR (100 MHz, $CDCl_3$) of **2a-6**.

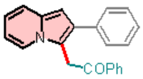

2b-6

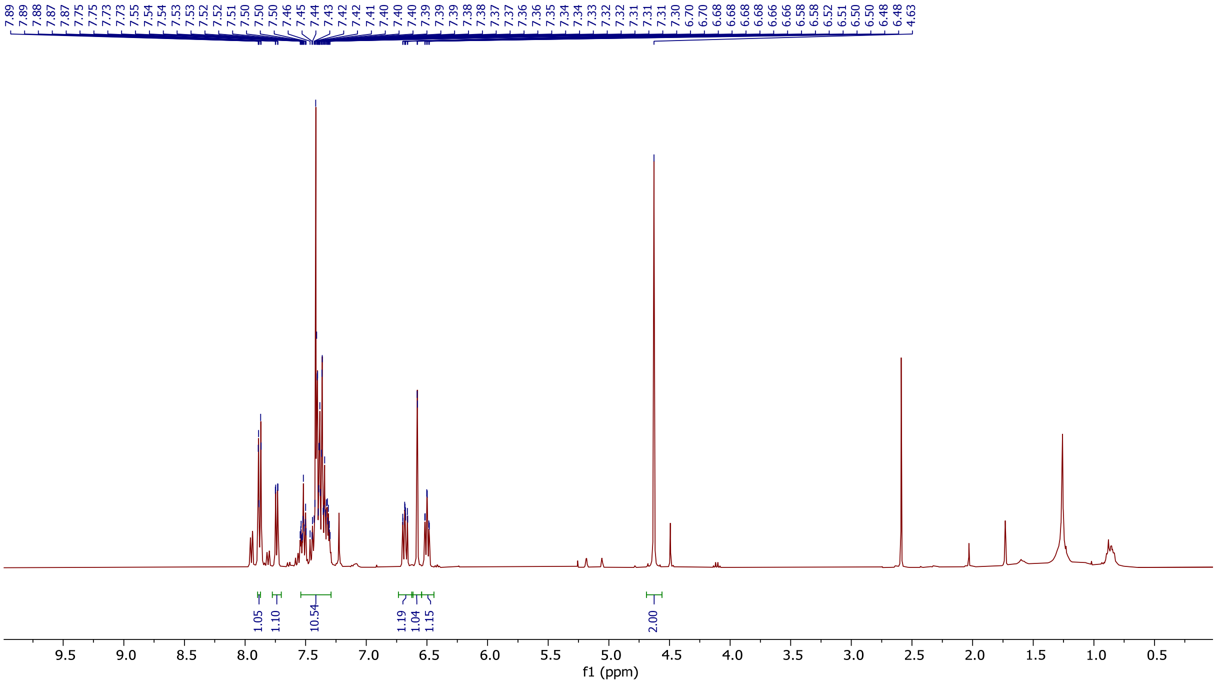

^{1}H NMR (400 MHz, $CDCl_3$) of **2b-6**.

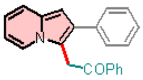

2b-6

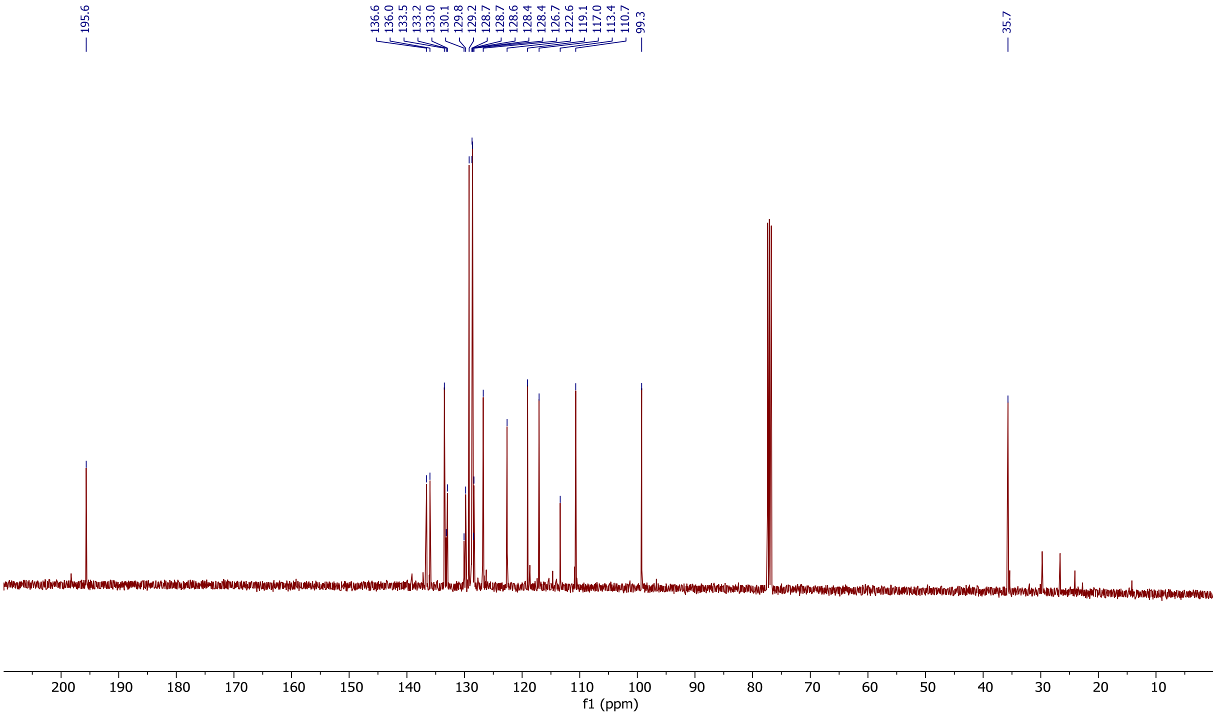

$^{13}C\{^{1}H\}$ NMR (100 MHz, $CDCl_3$) of **2b-6**.

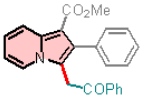

2c-6

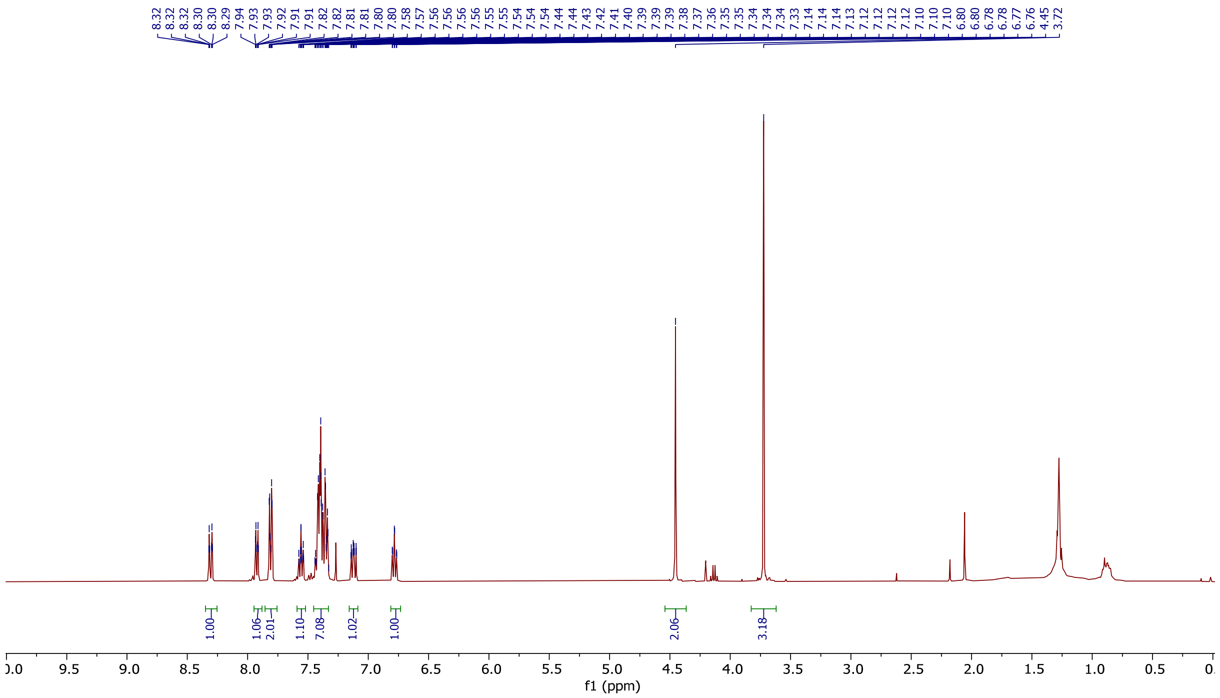

^{1}H NMR (400 MHz, $CDCl_3$) of **2c-6**.

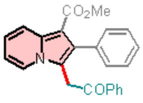

2c-6

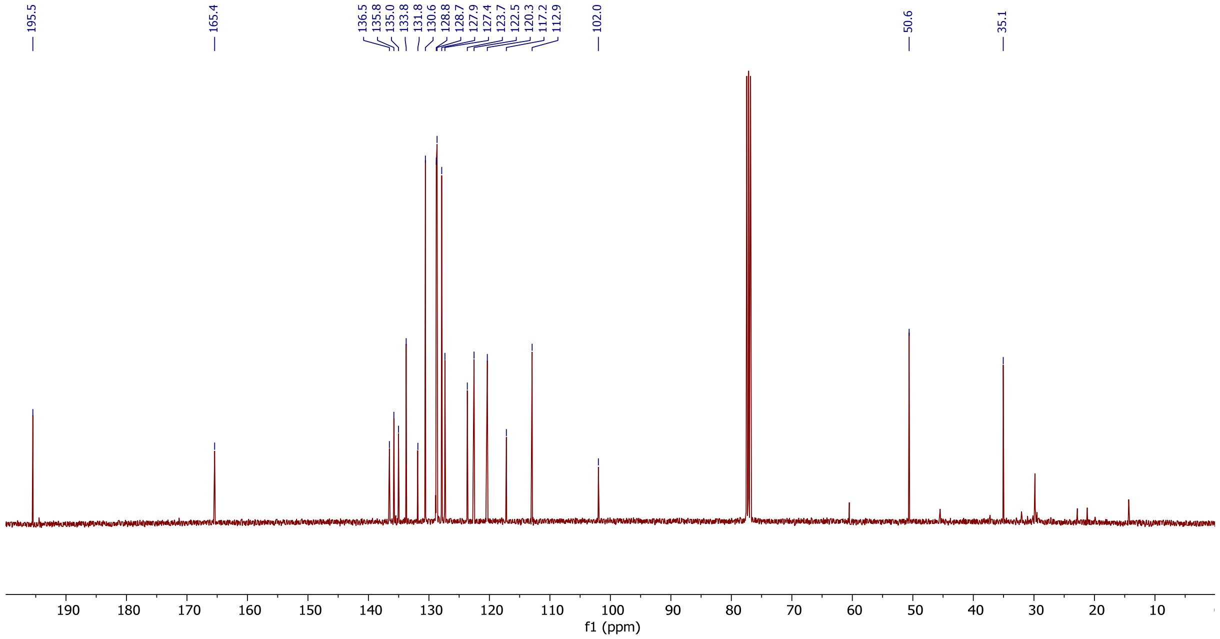

$^{13}C\{^{1}H\}$ NMR (100 MHz, $CDCl_3$) of **2c-6**.

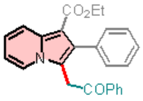

2d-6

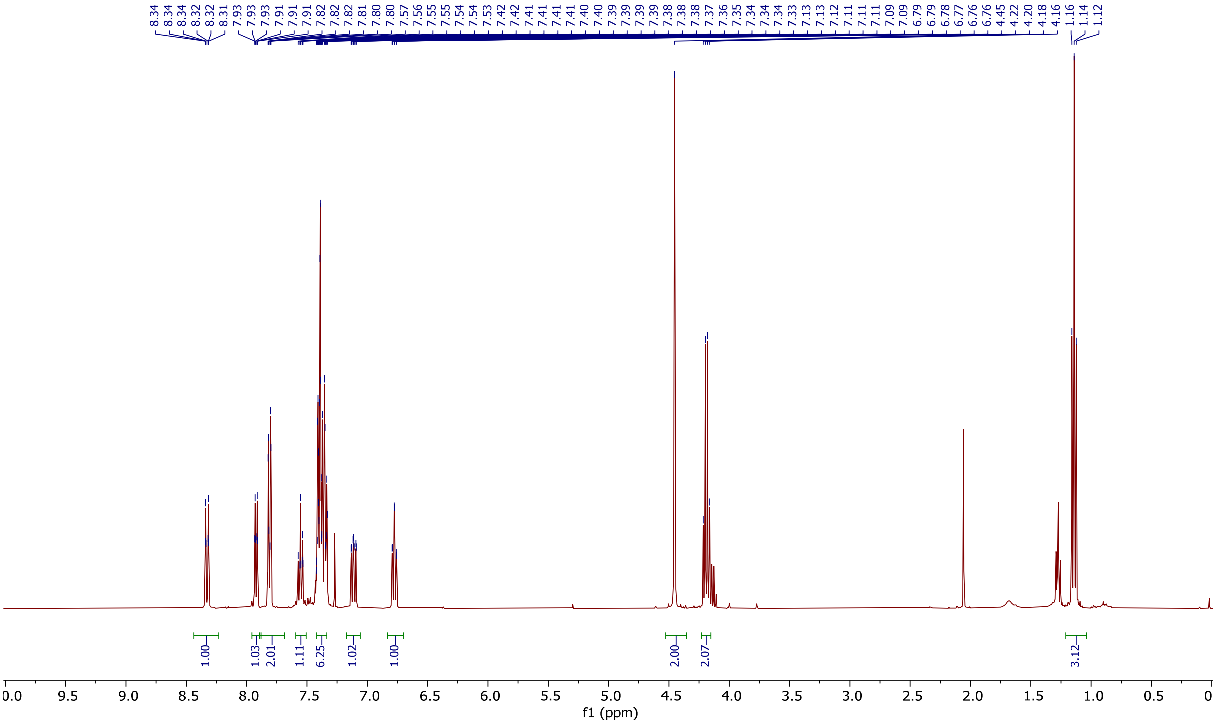

^{1}H NMR (400 MHz, CDCl$_3$) of **2d-6**.

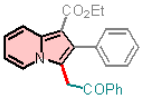

2d-6

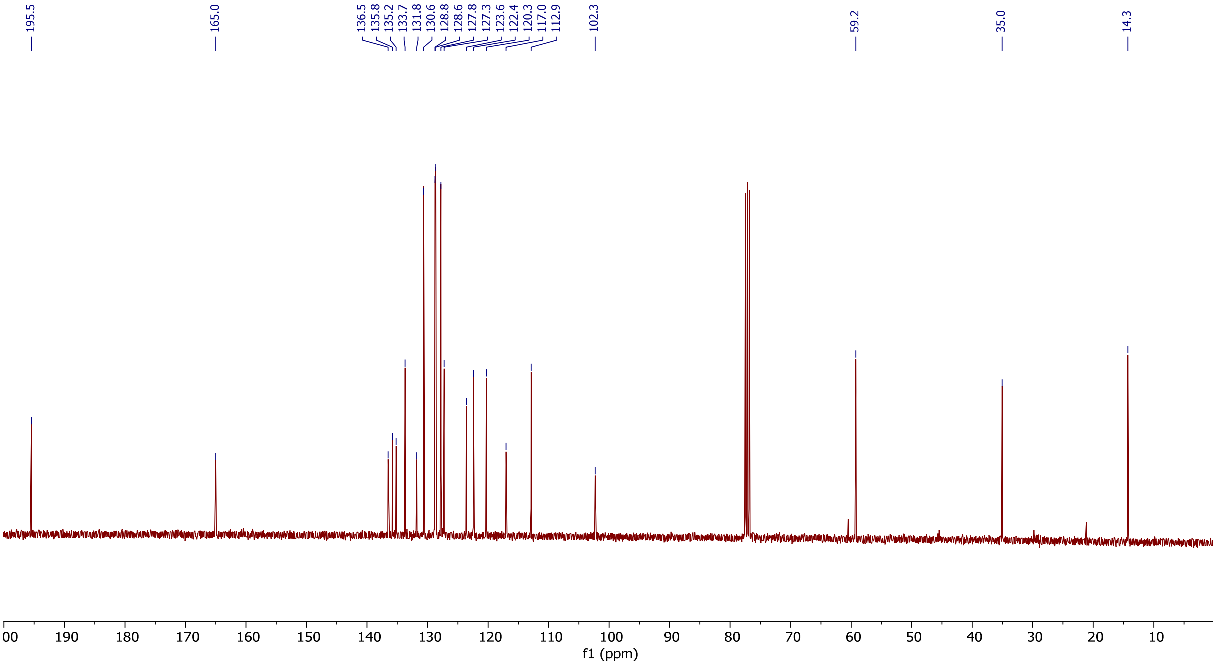

^{13}C{^{1}H} NMR (100 MHz, CDCl$_3$) of **2d-6**.

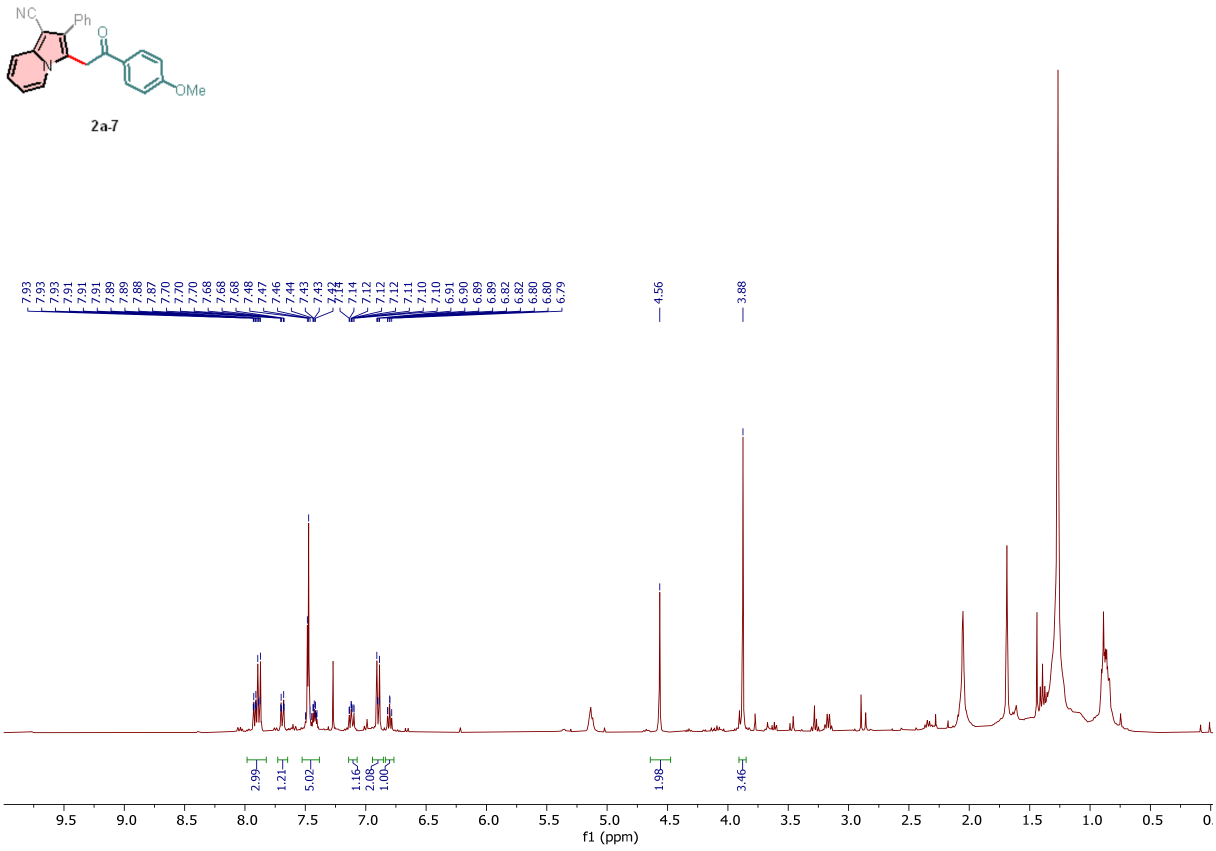

^{1}H NMR (400 MHz, $CDCl_3$) of **2a-7**.

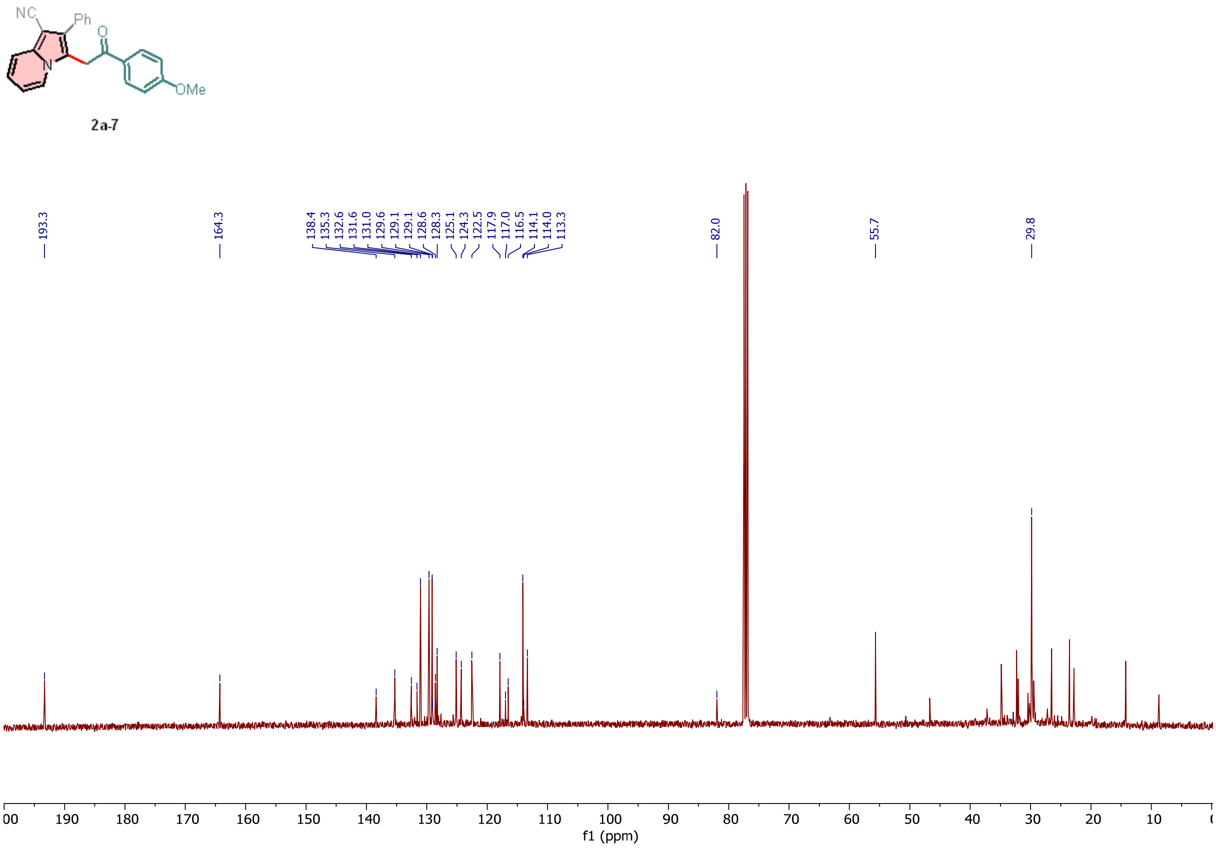

$^{13}C\{^{1}H\}$ NMR (100 MHz, $CDCl_3$) of **2a-7**.

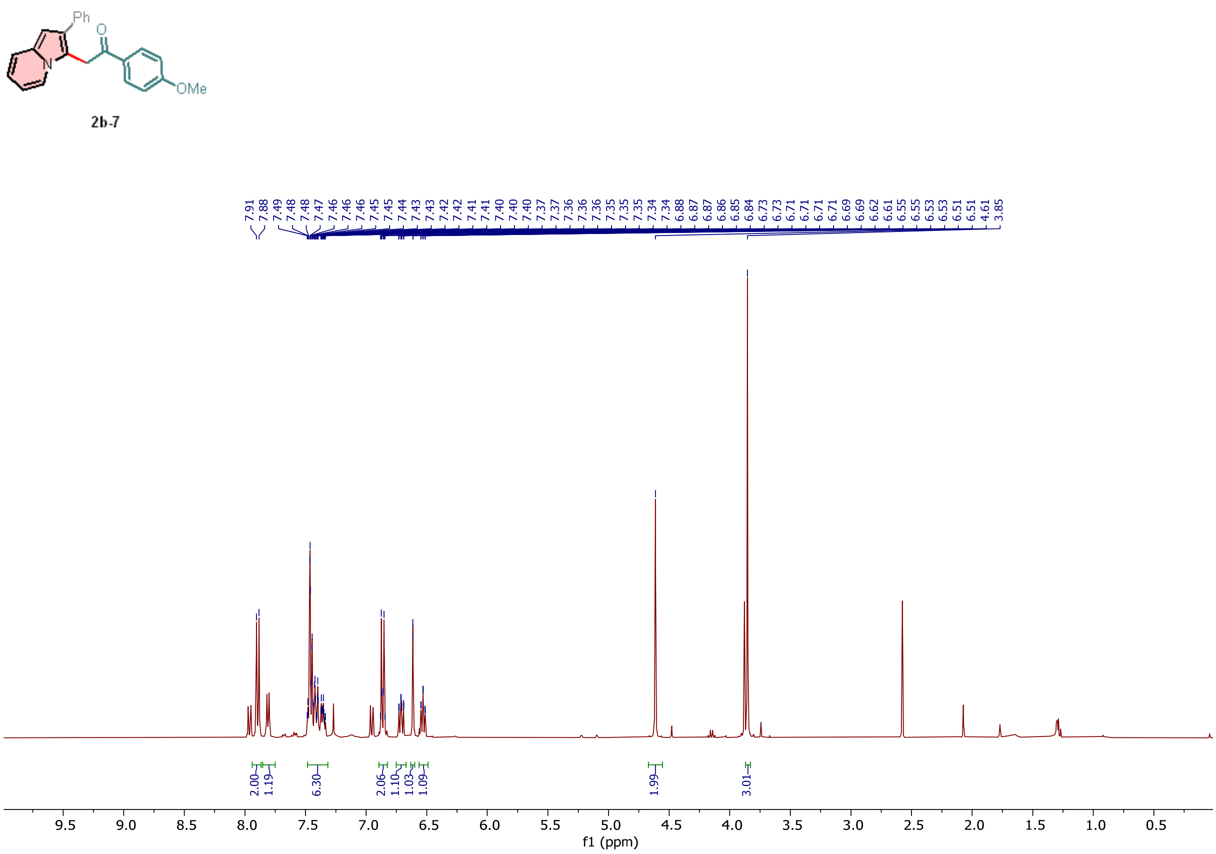

^{1}H NMR (400 MHz, $CDCl_3$) of **2b-7**.

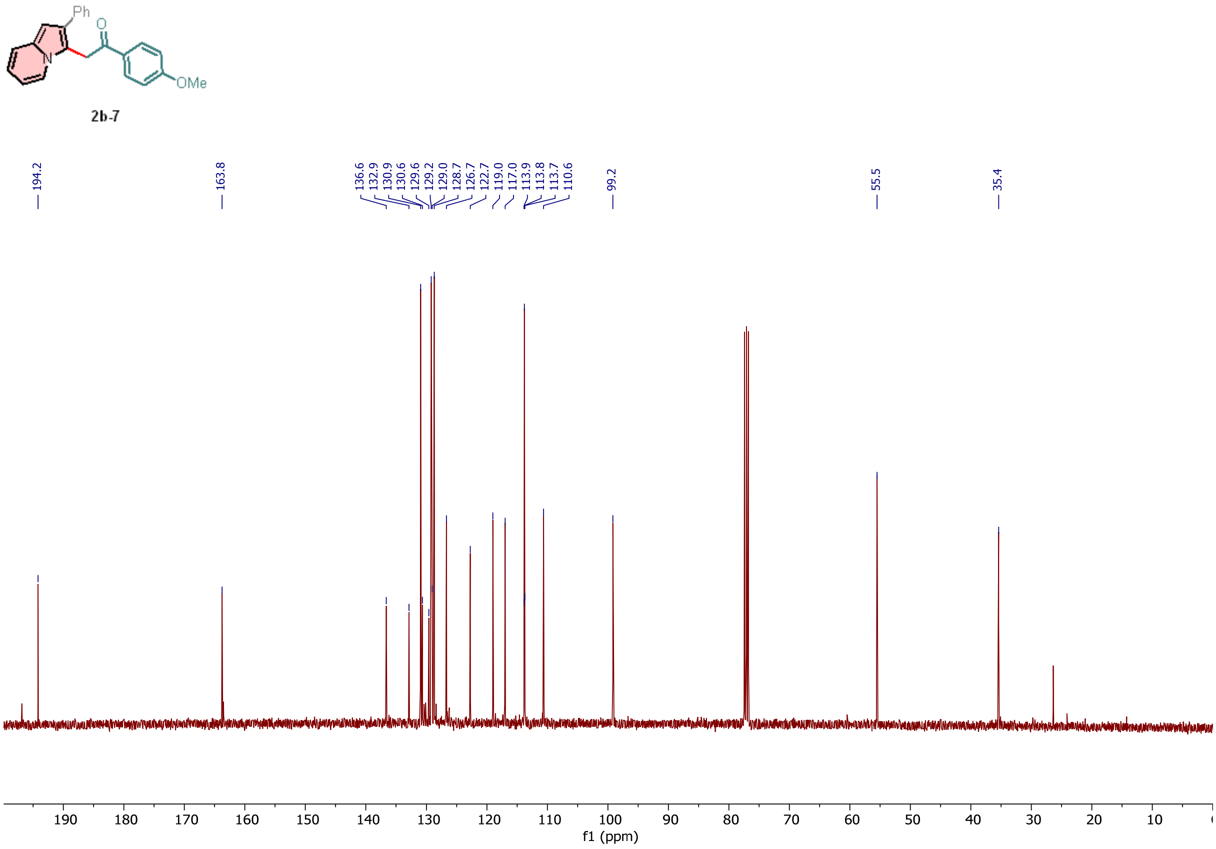

$^{13}C\{^1H\}$ NMR (100 MHz, $CDCl_3$) of **2b-7**.

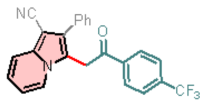

2a-8

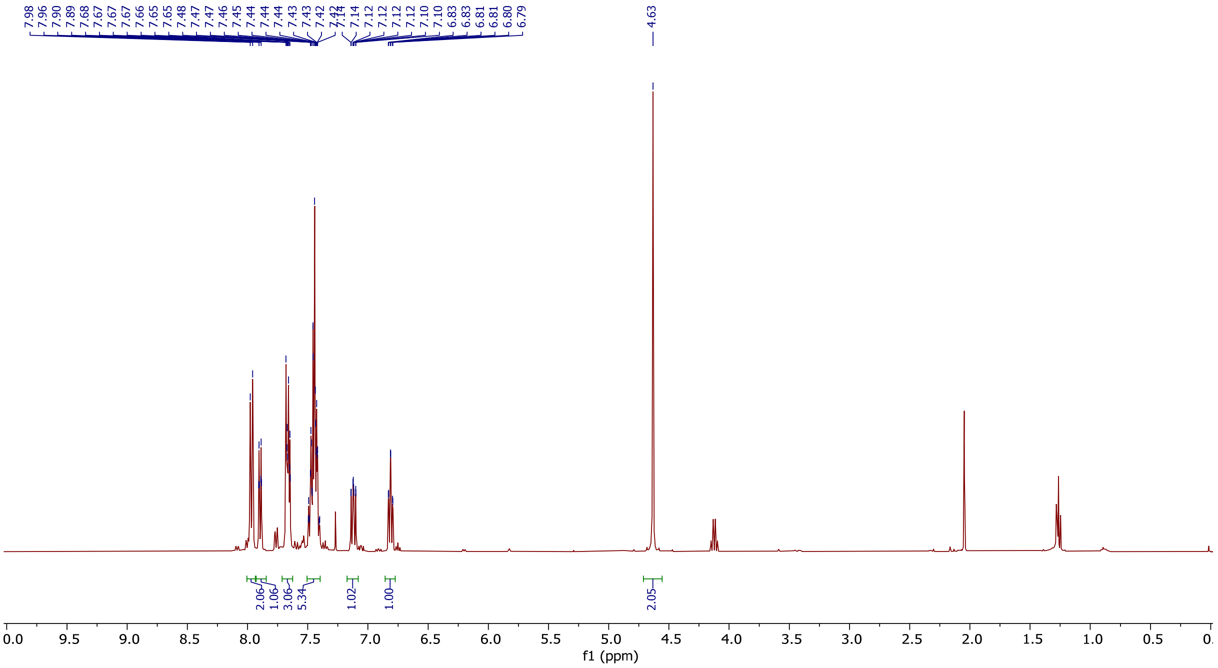

^{1}H NMR (400 MHz, $CDCl_3$) of **2a-8**.

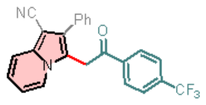

2a-8

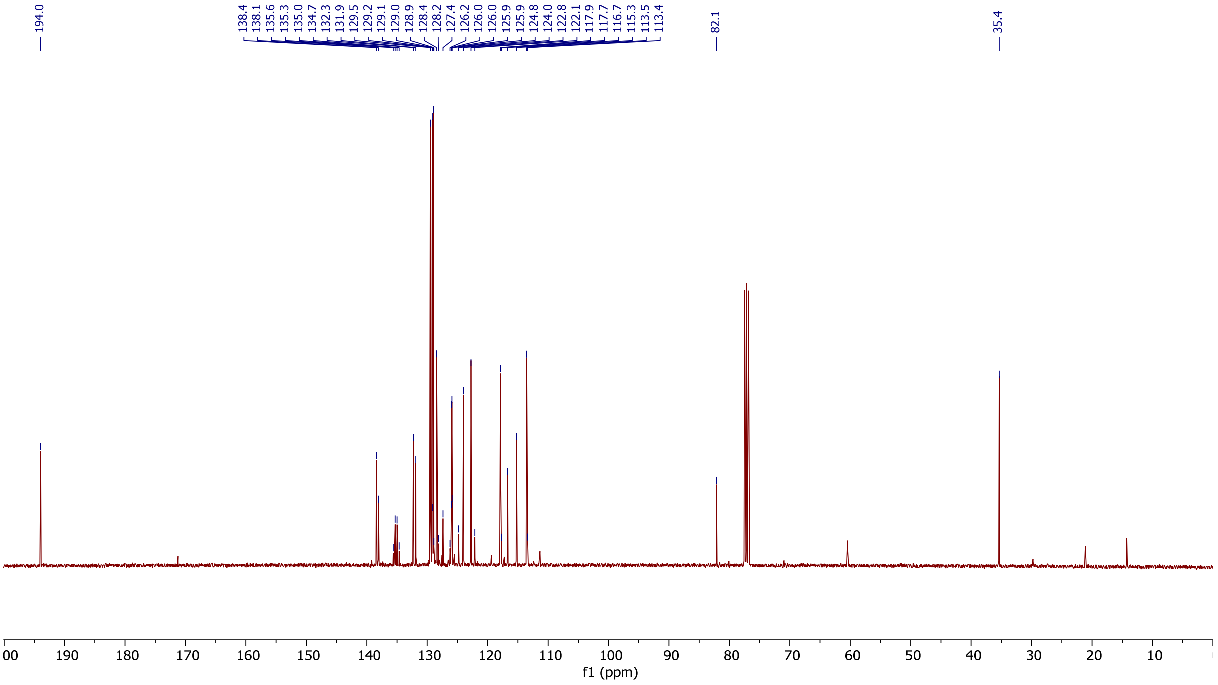

$^{13}C\{^1H\}$ NMR (100 MHz, $CDCl_3$) of **2a-8**.

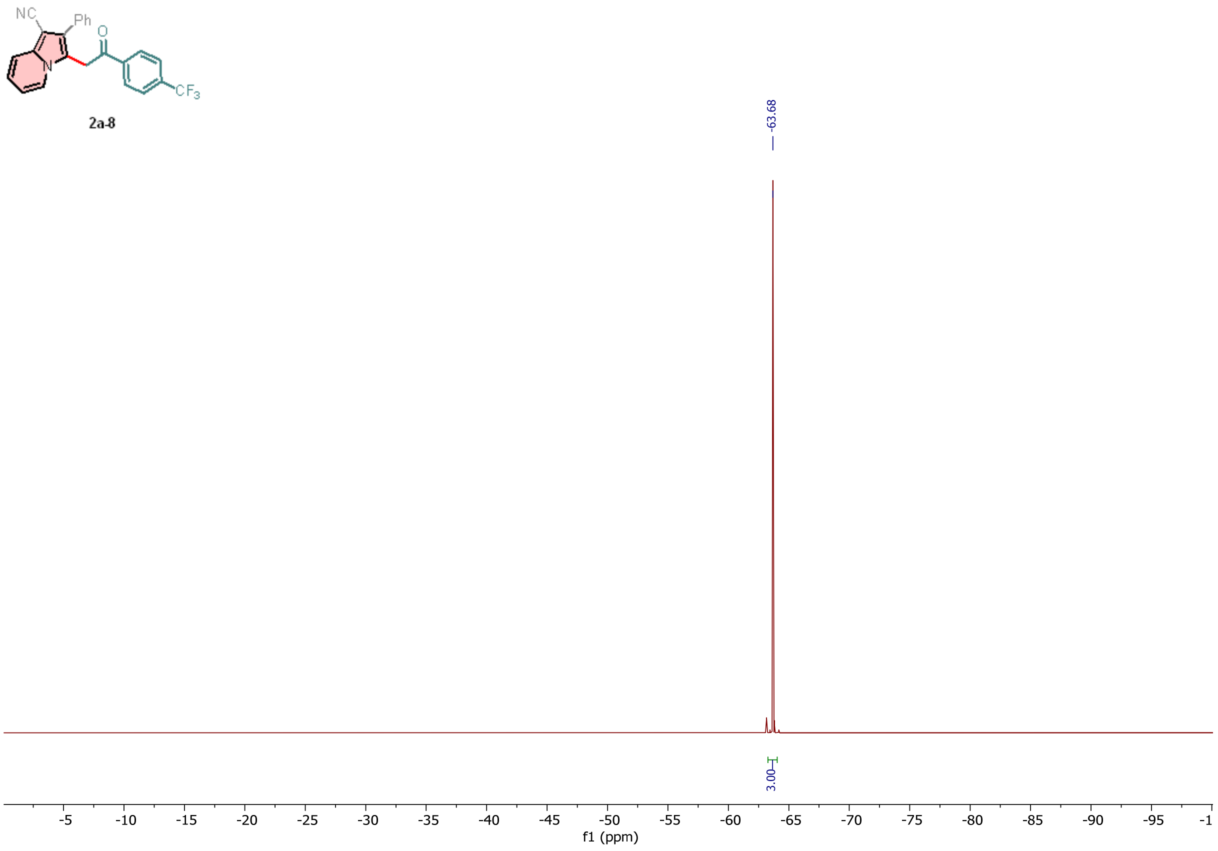

$^{19}F\{^{1}H\}$ NMR (377 MHz, $CDCl_3$) of **2a-8**.

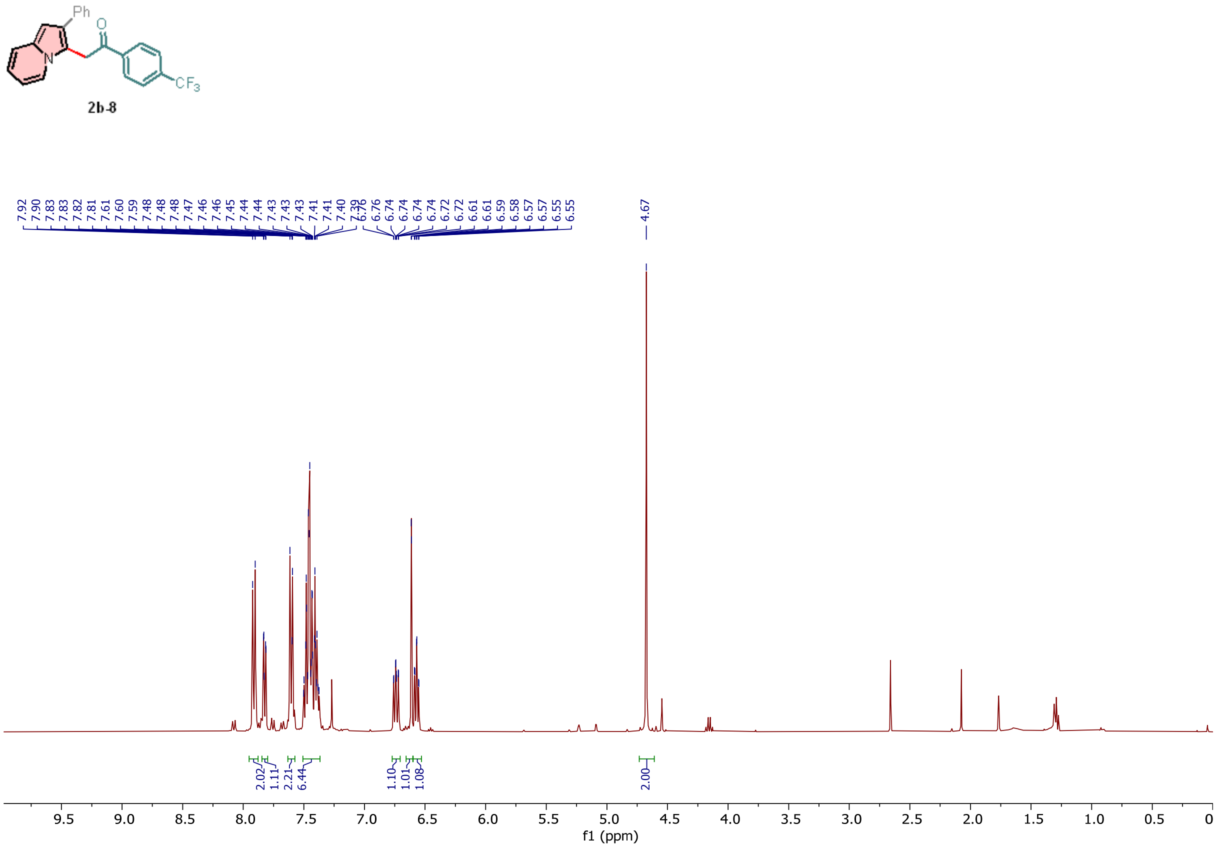

^{1}H NMR (400 MHz, $CDCl_3$) of **2b-8**.

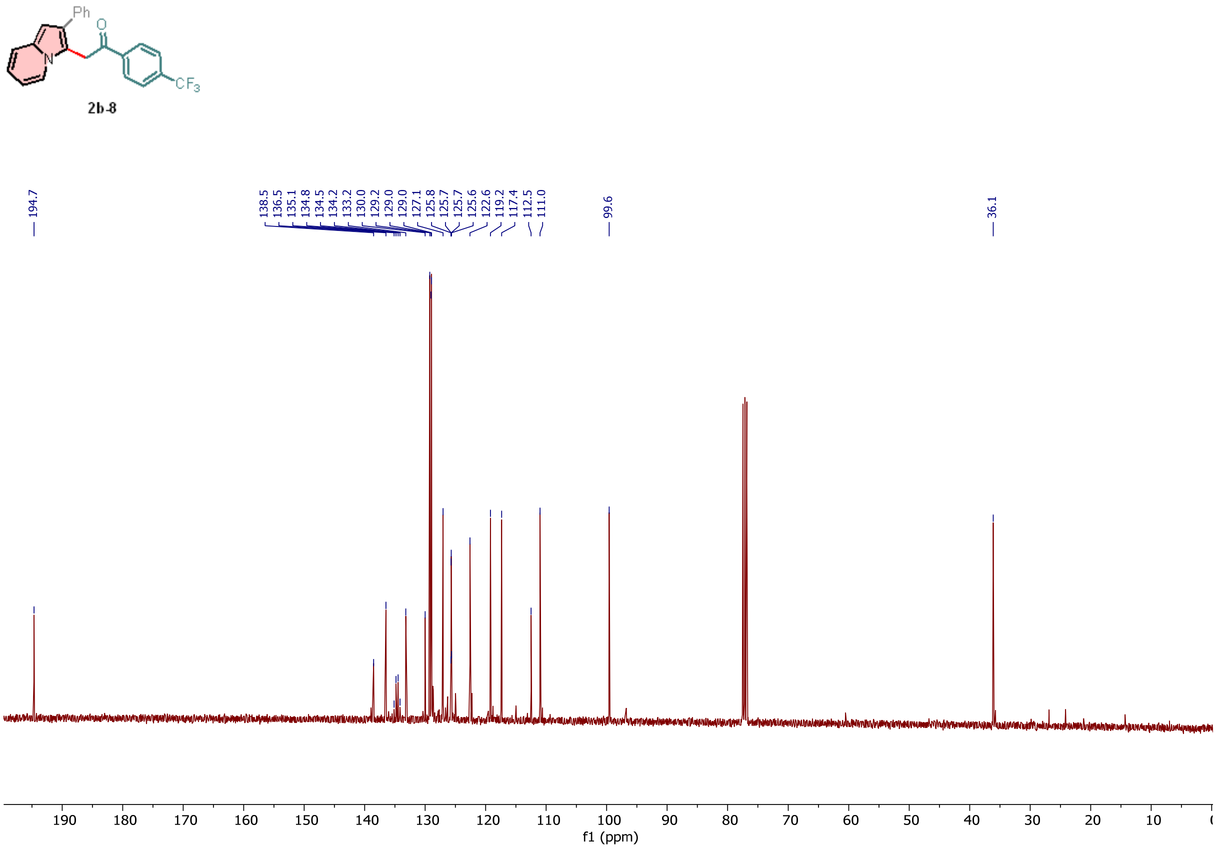

$^{13}C\{^{1}H\}$ NMR (100 MHz, $CDCl_3$) of **2b-8**.

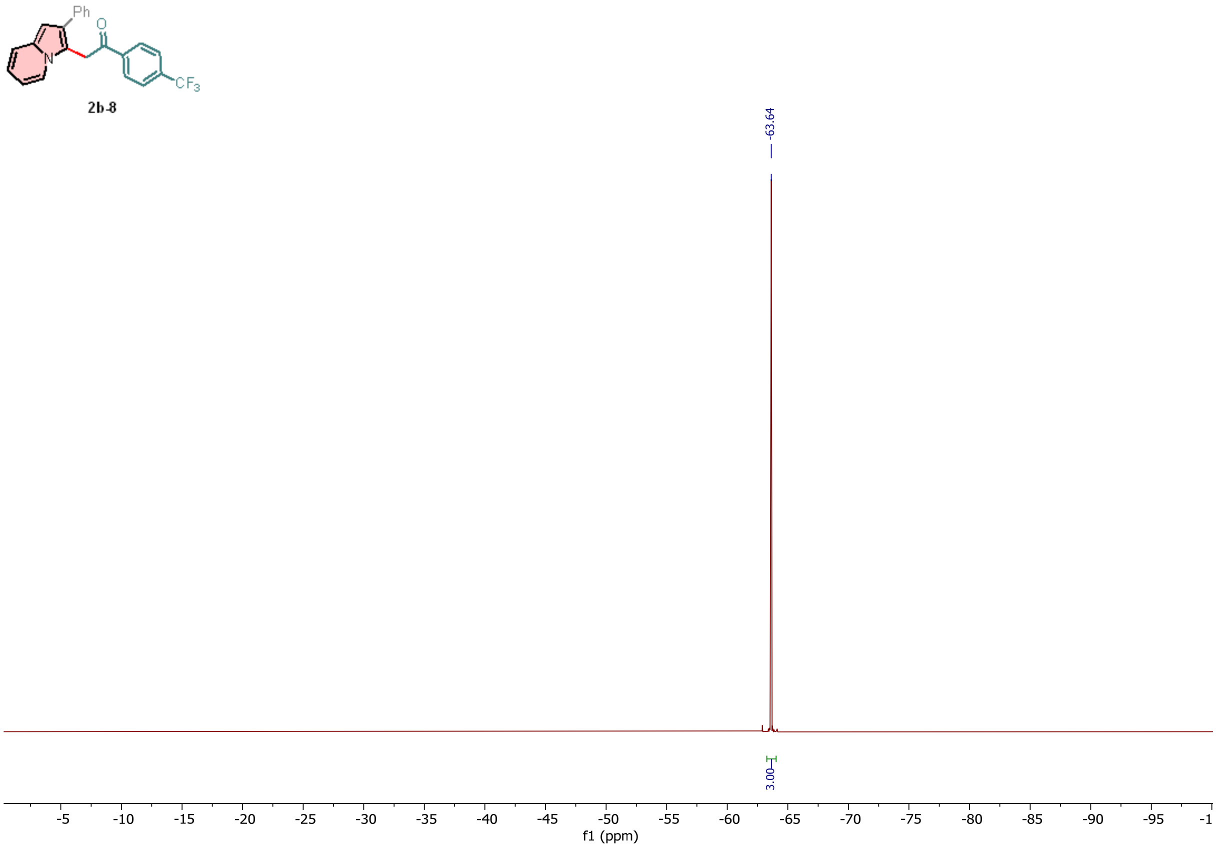

$^{19}F\{^{1}H\}$ NMR (377 MHz, $CDCl_3$) of **2b-8**.

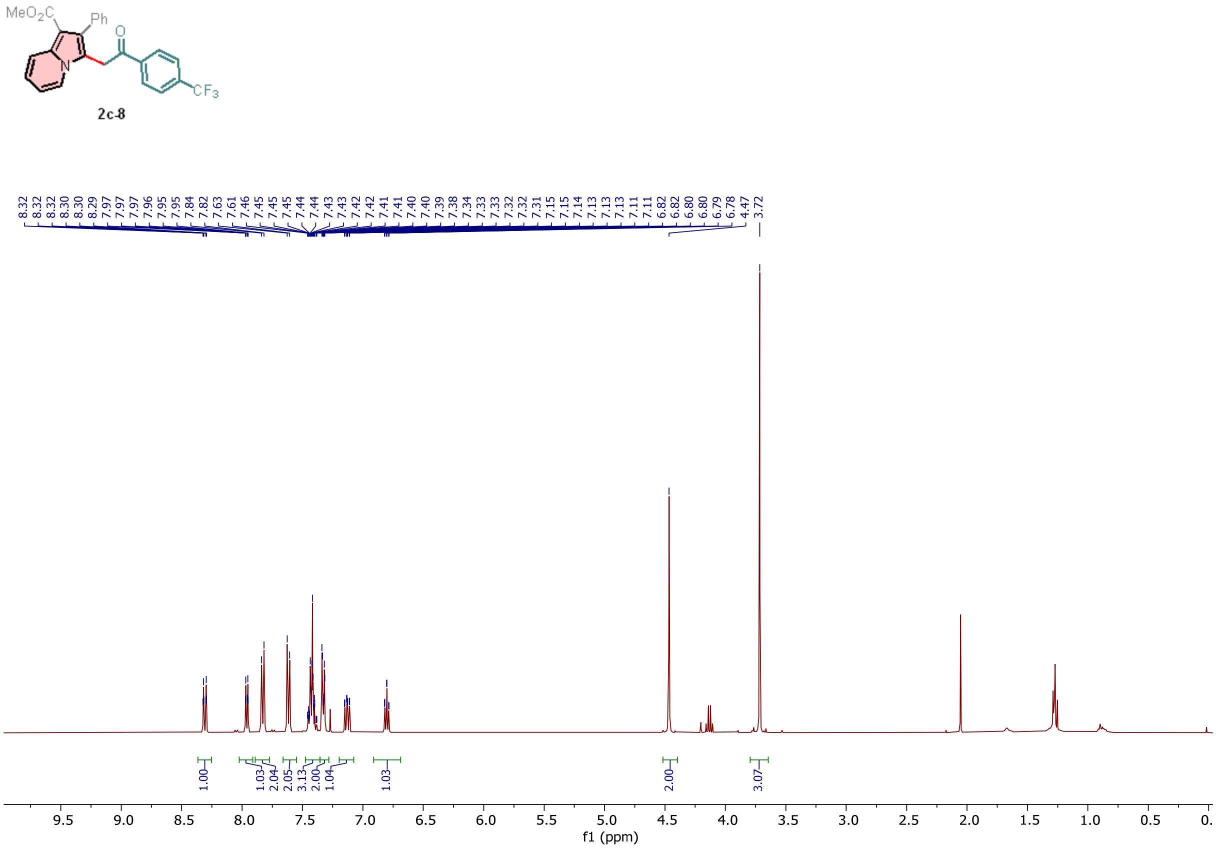

^{1}H NMR (400 MHz, $CDCl_3$) of **2c-8**.

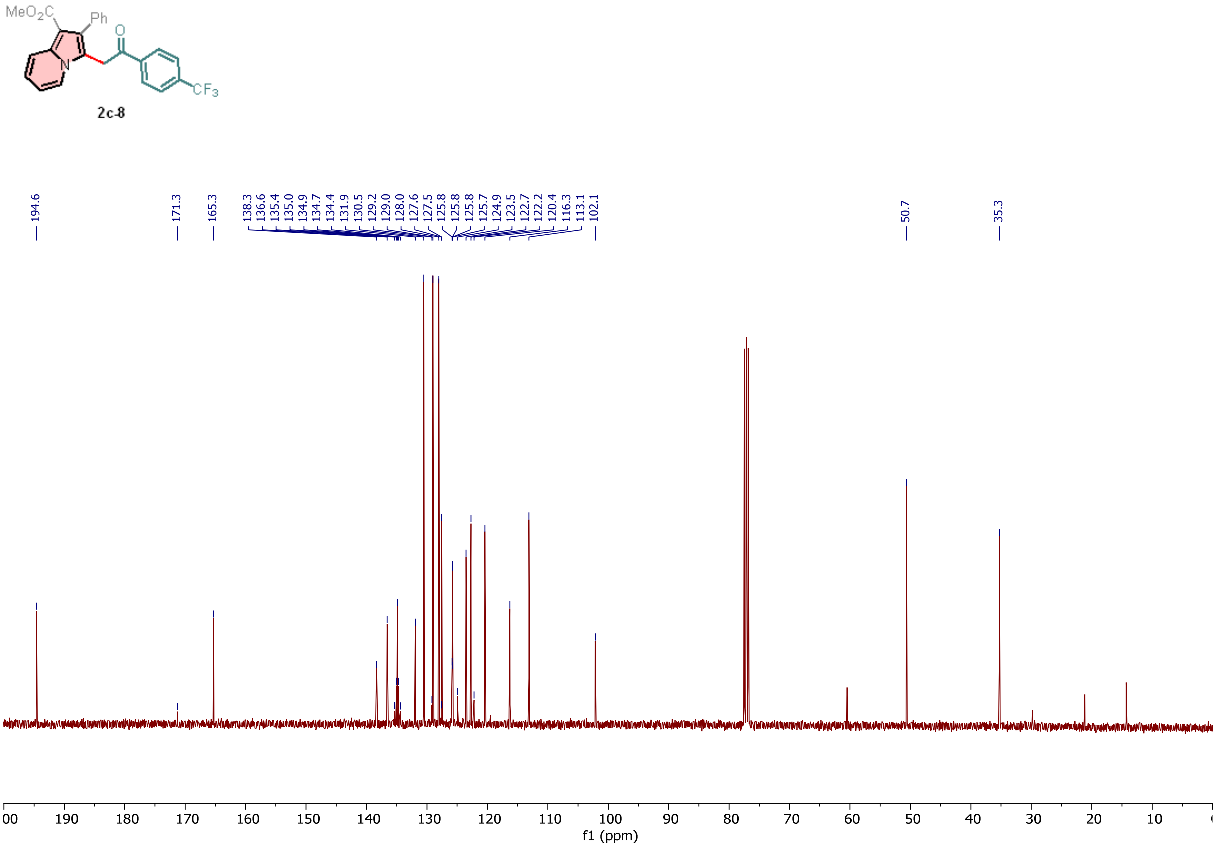

^{13}C{^{1}H} NMR (100 MHz, $CDCl_3$) of **2c-8**.

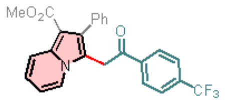

2c-8

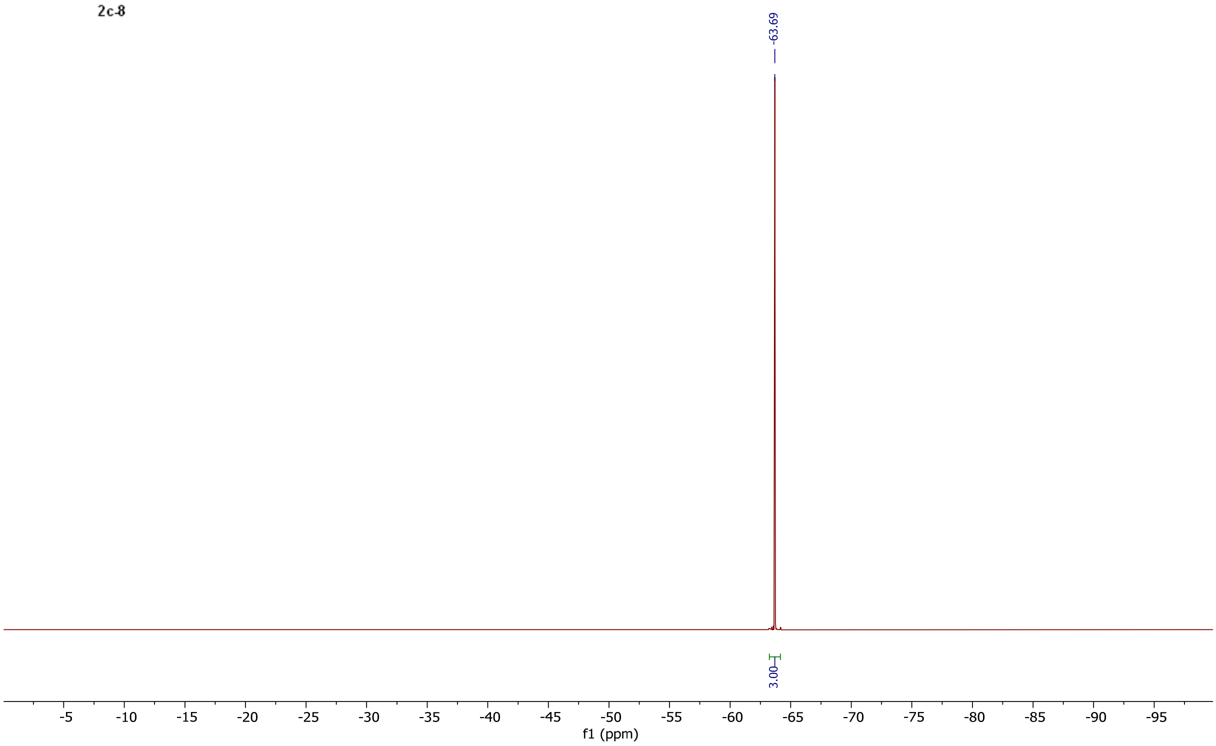

$^{19}F\{^{1}H\}$ NMR (377 MHz, $CDCl_3$) of **2c-8**.

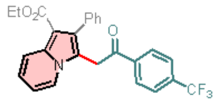

2d-8

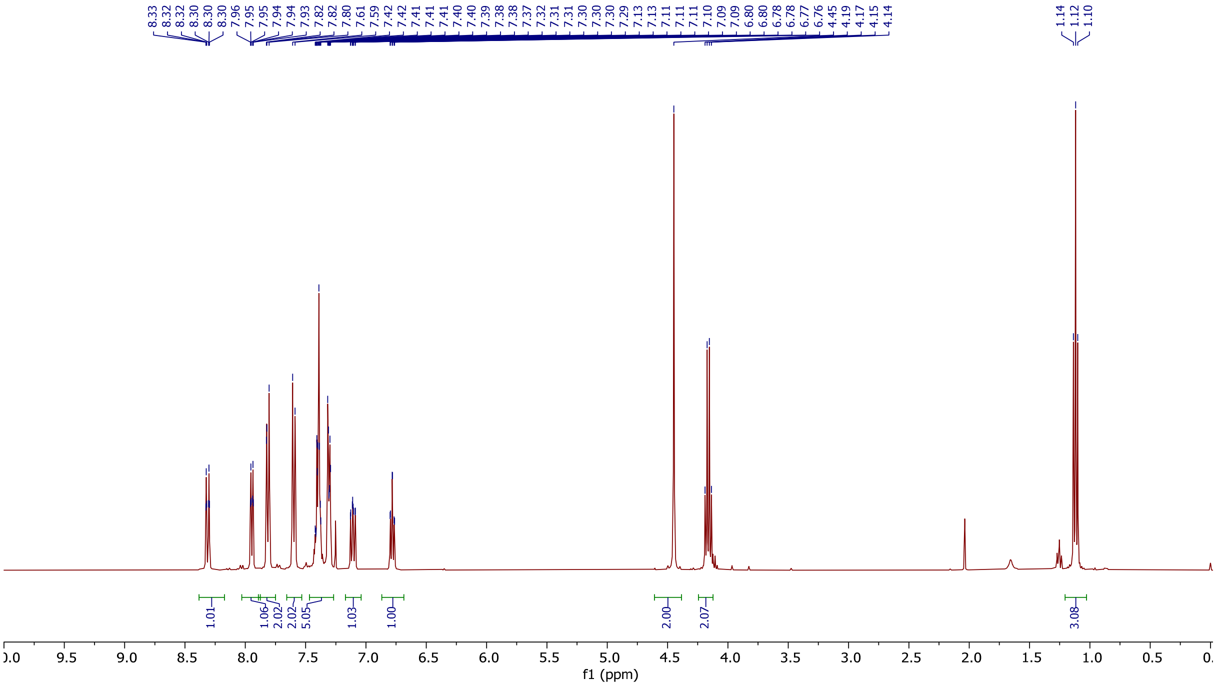

^{1}H NMR (400 MHz, $CDCl_3$) of **2d-8**.

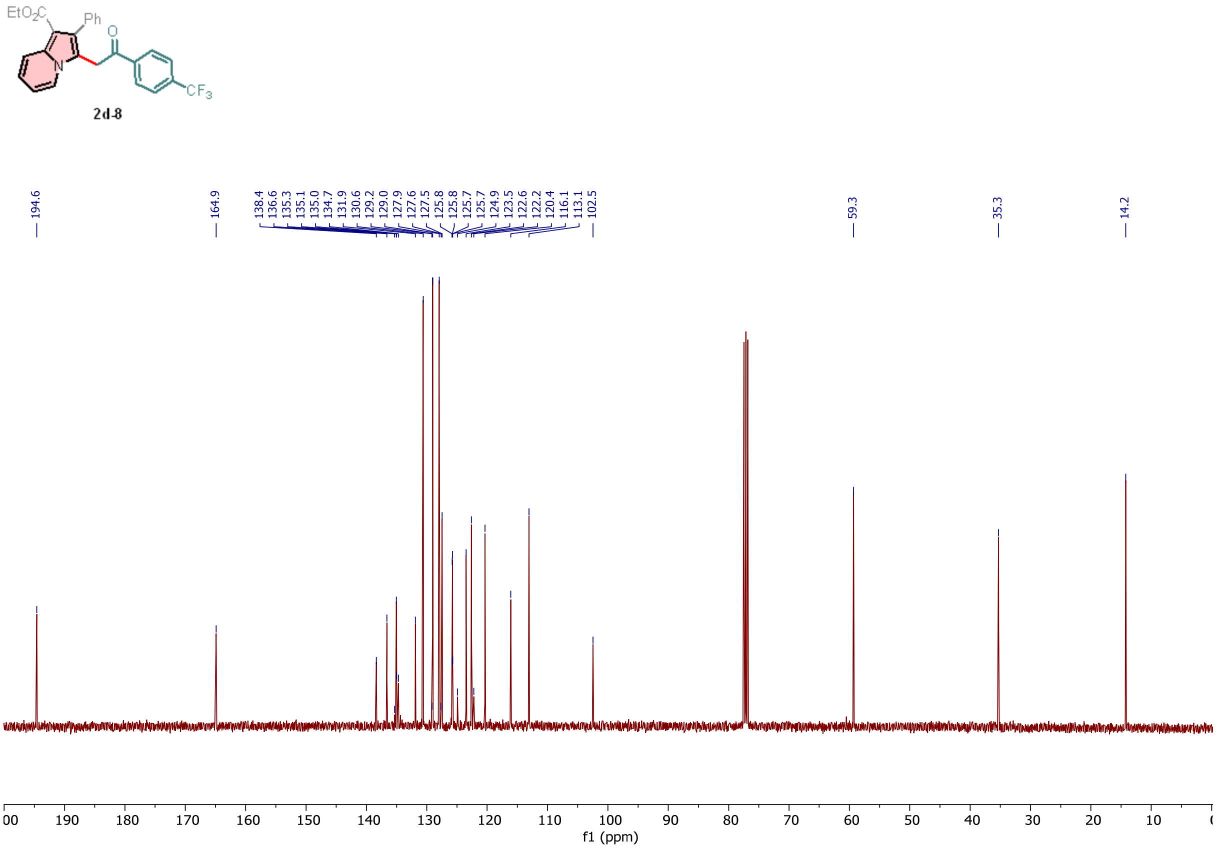

$^{13}C\{^{1}H\}$ NMR (100 MHz, $CDCl_3$) of **2d-8**.

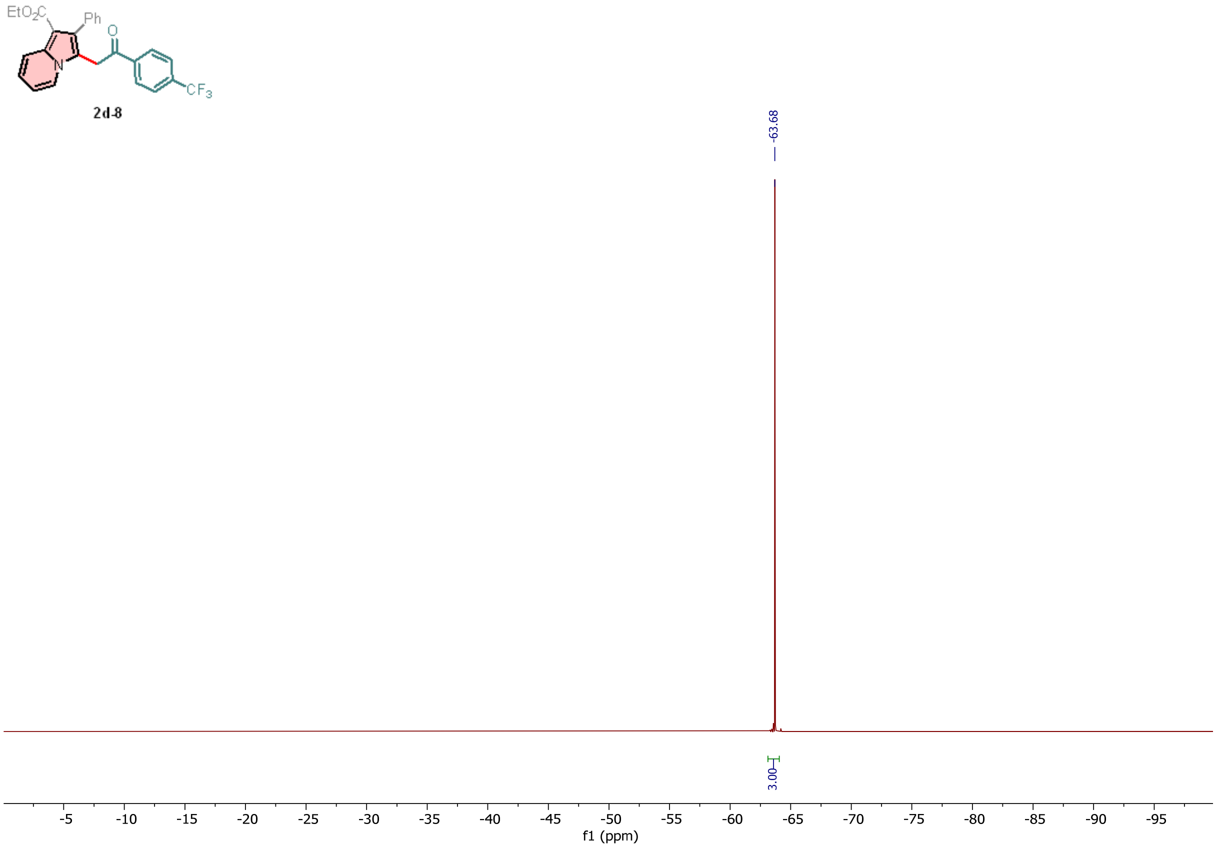

$^{19}F\{^{1}H\}$ NMR (377 MHz, $CDCl_3$) of **2d-8**.

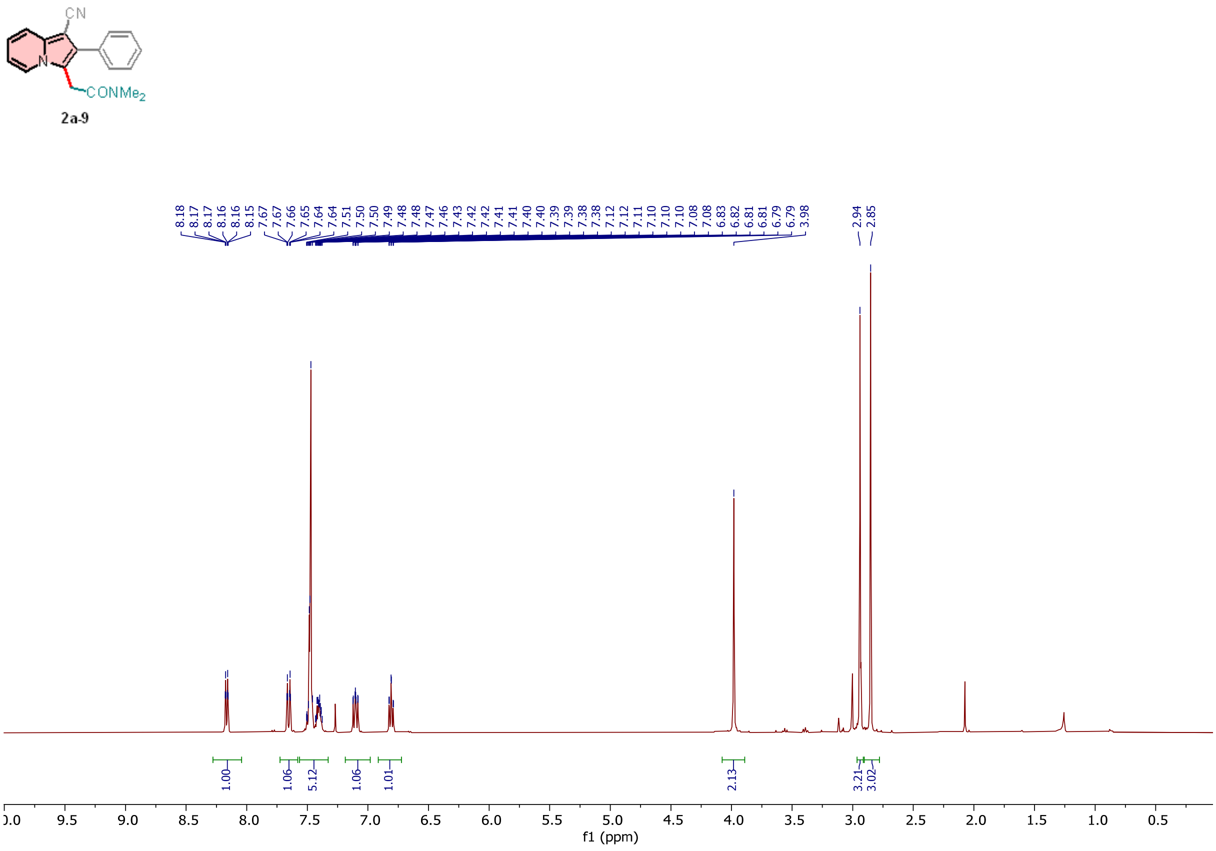

^{1}H NMR (400 MHz, $CDCl_3$) of **2a-9**.

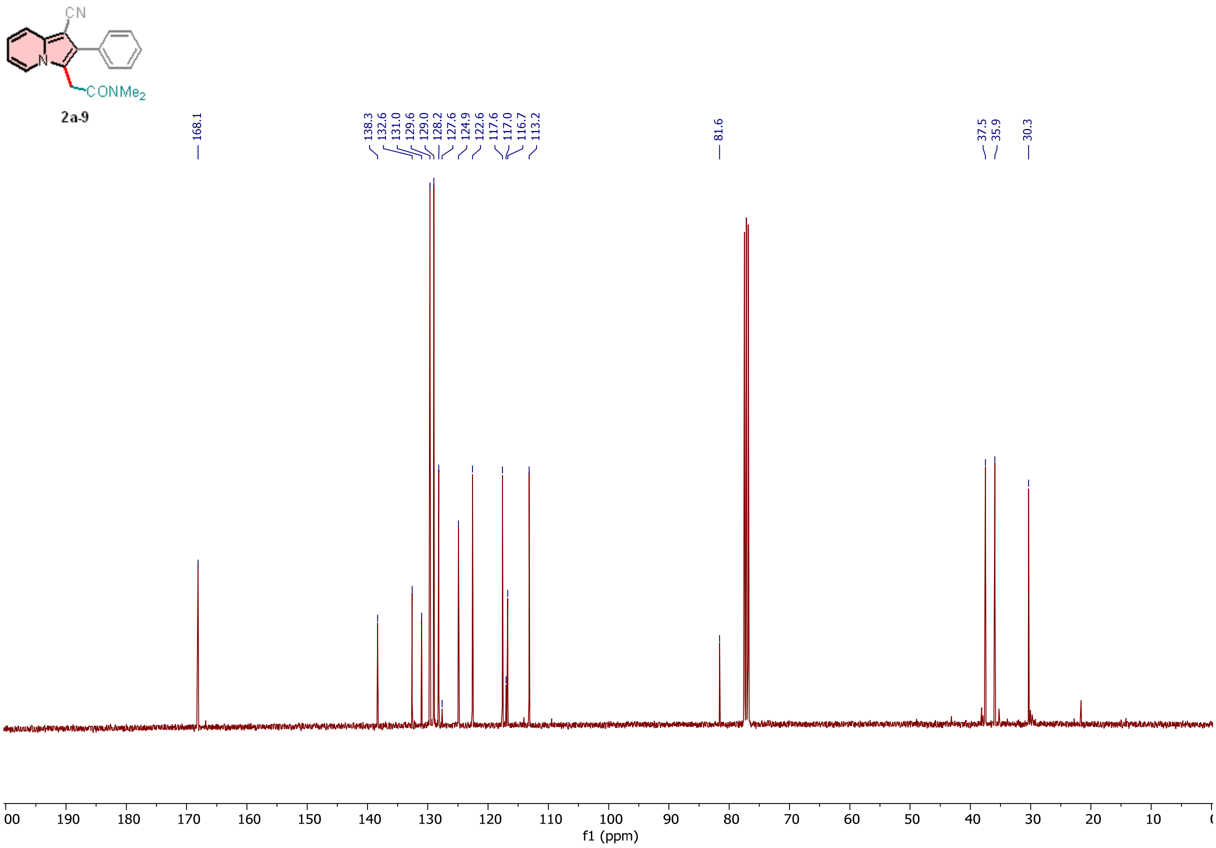

$^{13}C\{^{1}H\}$ NMR (100 MHz, $CDCl_3$) of **2a-9**.

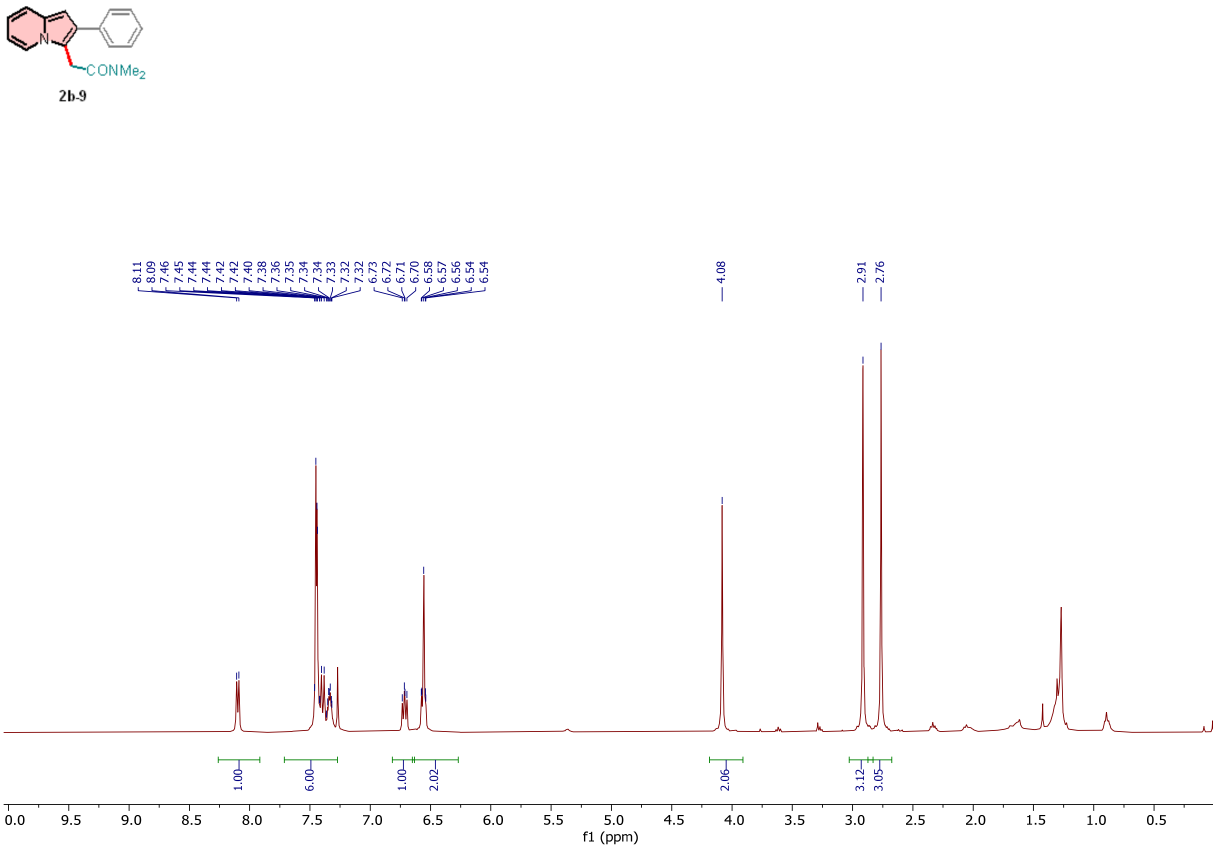

^{1}H NMR (400 MHz, CDCl$_3$) of **2b-9**.

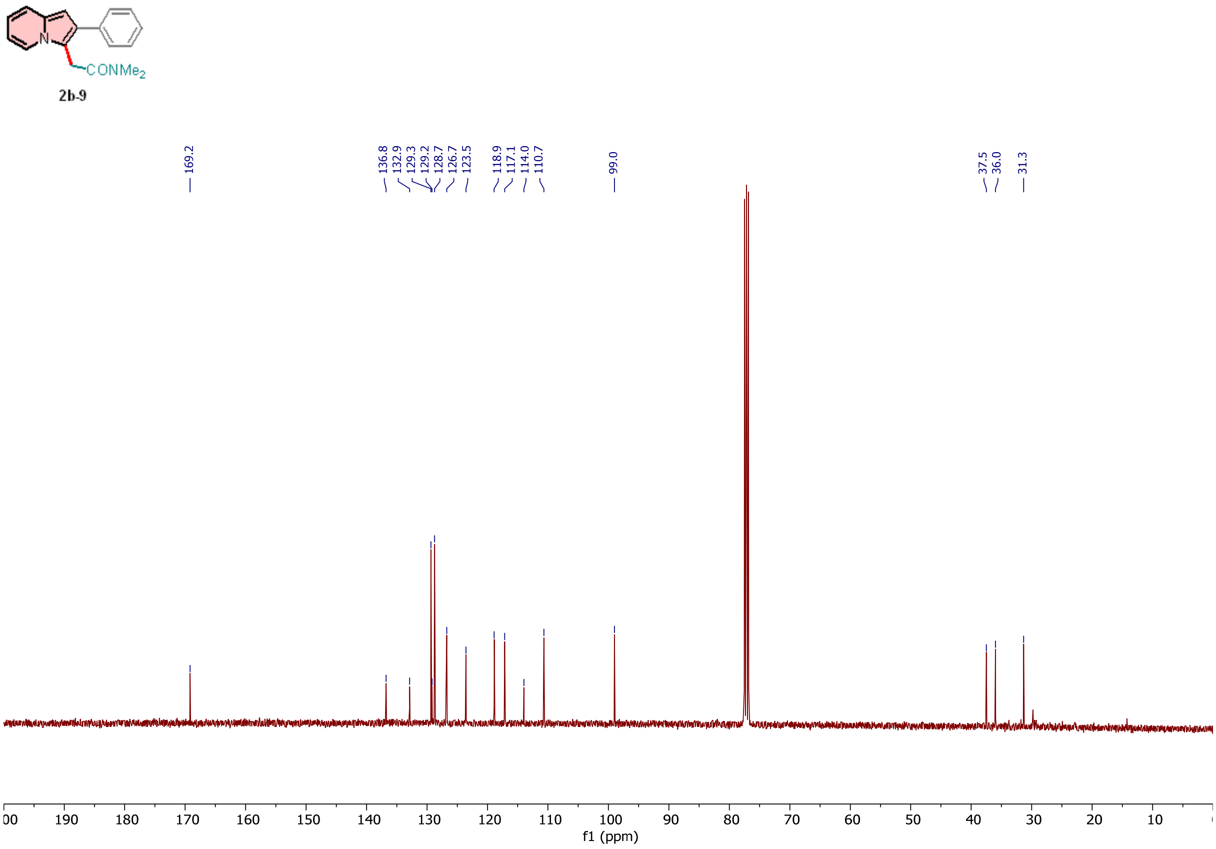

^{13}C{^{1}H} NMR (100 MHz, CDCl$_3$) of **2b-9**.

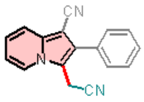

2a-10

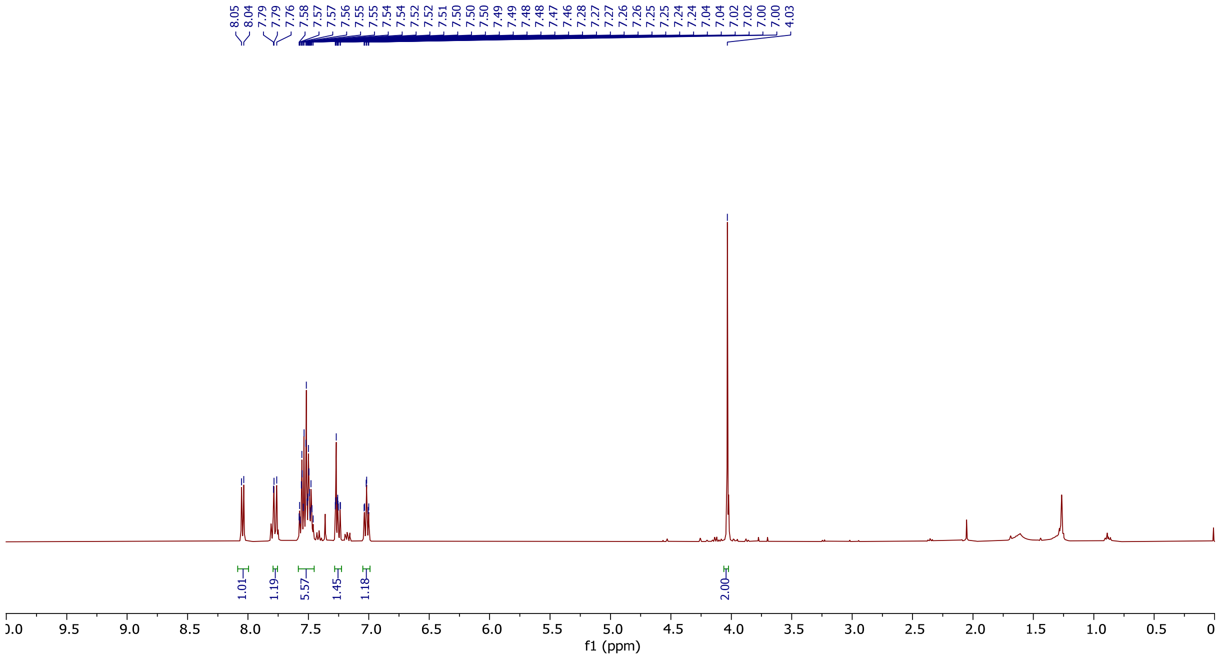

^{1}H NMR (400 MHz, $CDCl_3$) of **2a-10**.

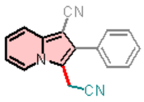

2a-10

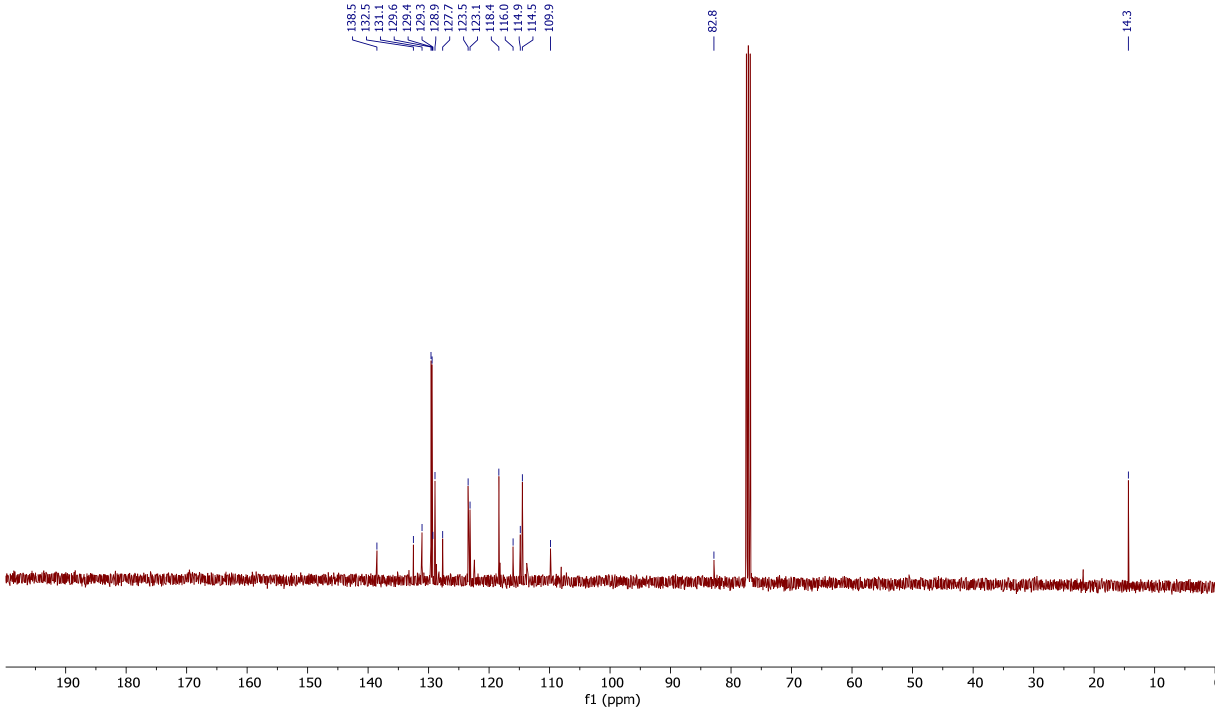

^{13}C{^{1}H} NMR (100 MHz, $CDCl_3$) of **2a-10**.

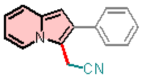

2b-10

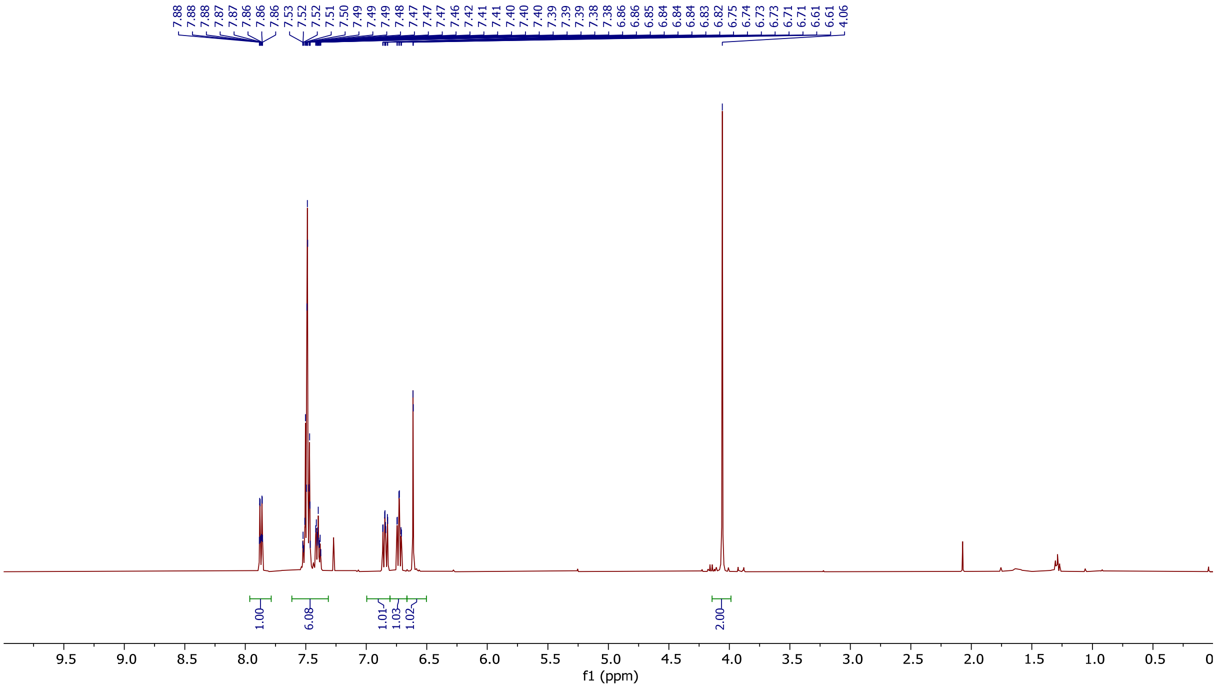

^{1}H NMR (400 MHz, $CDCl_3$) of **2b-10**.

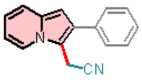

2b-10

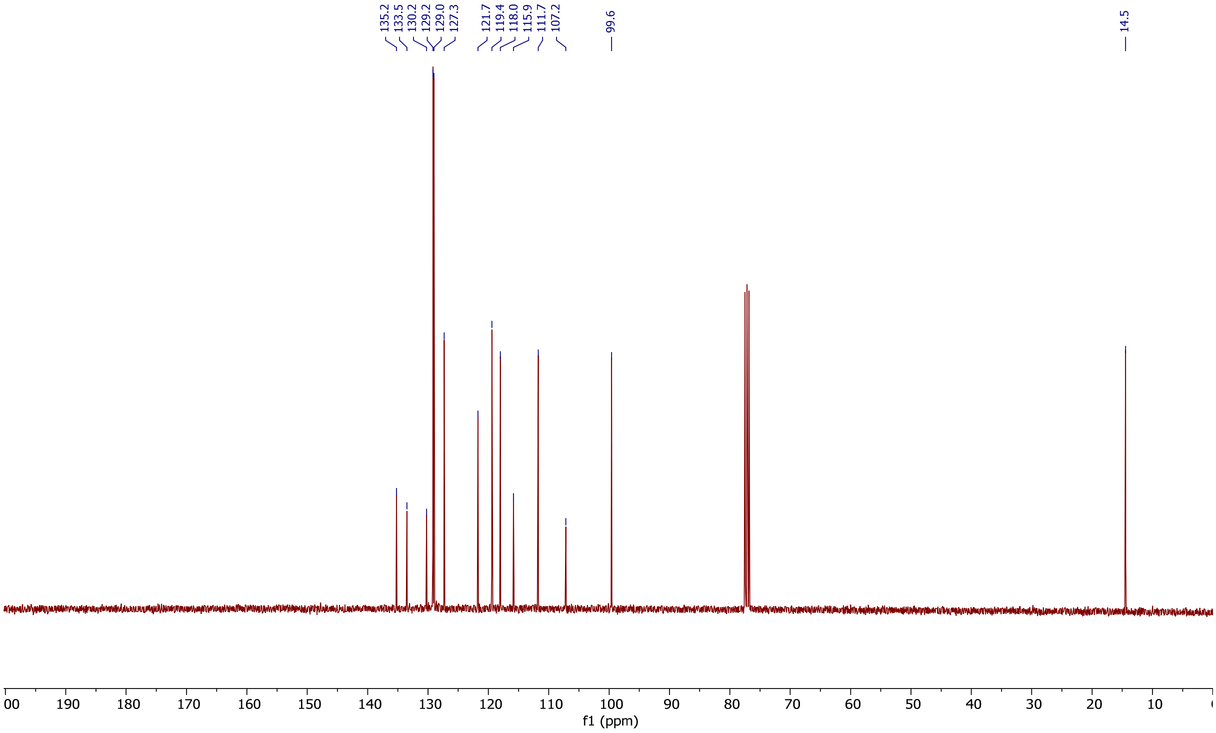

$^{13}C\{^{1}H\}$ NMR (100 MHz, $CDCl_3$) of **2b-10**.

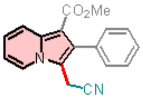

2c-10

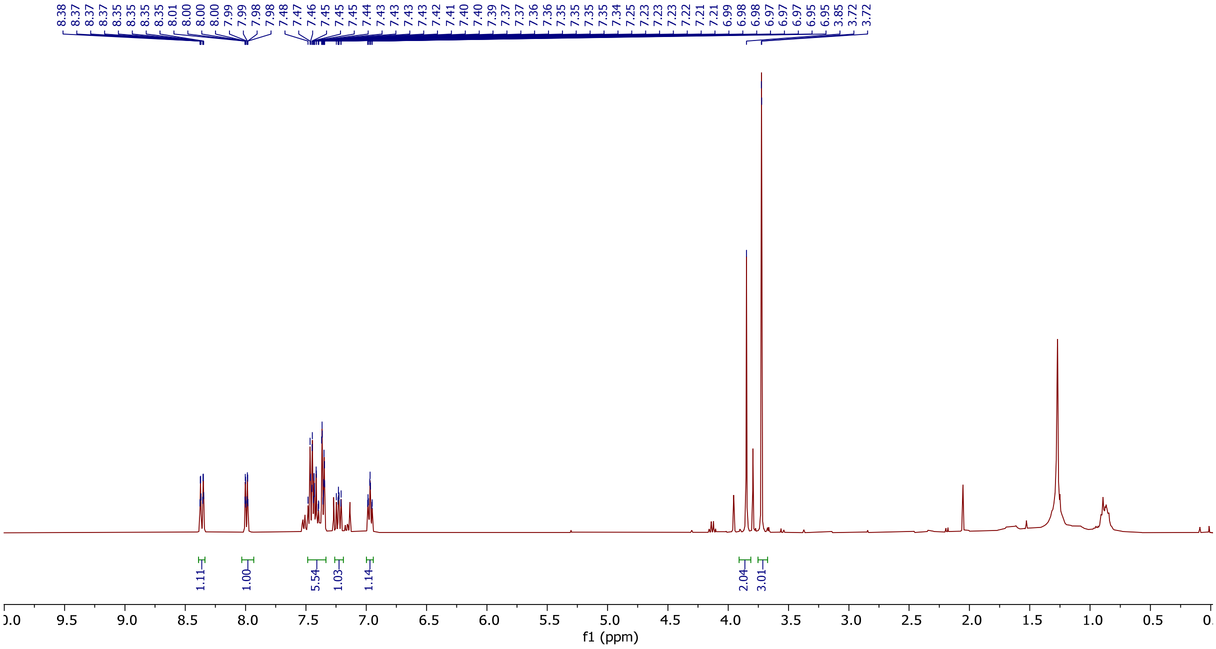

^{1}H NMR (400 MHz, $CDCl_3$) of **2c-10**.

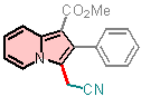

2c-10

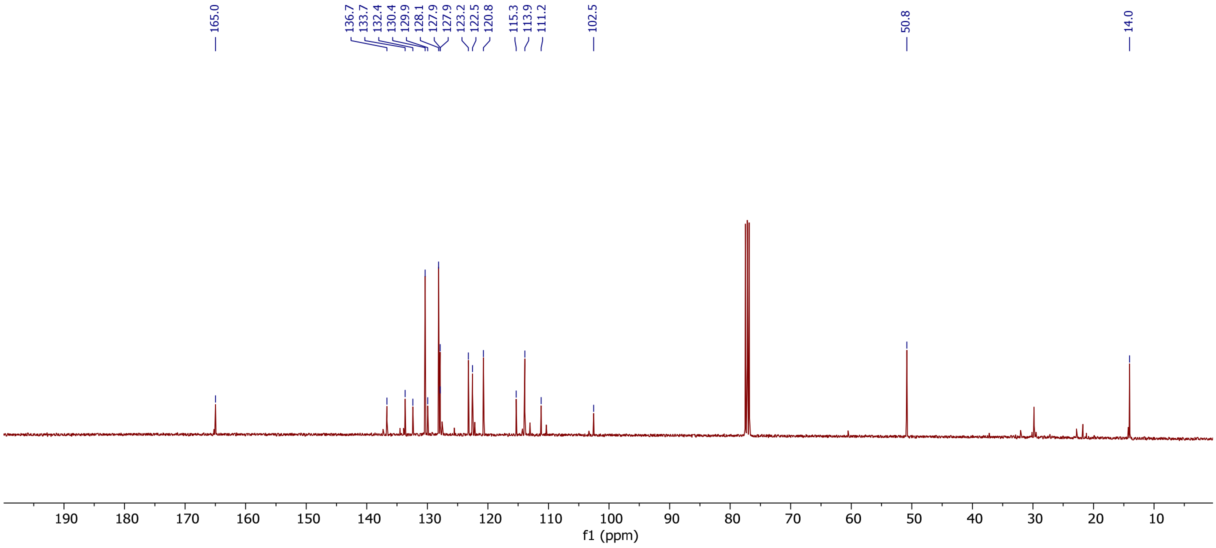

$^{13}C\{^1H\}$ NMR (100 MHz, $CDCl_3$) of **2c-10**.

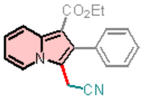

2d-10

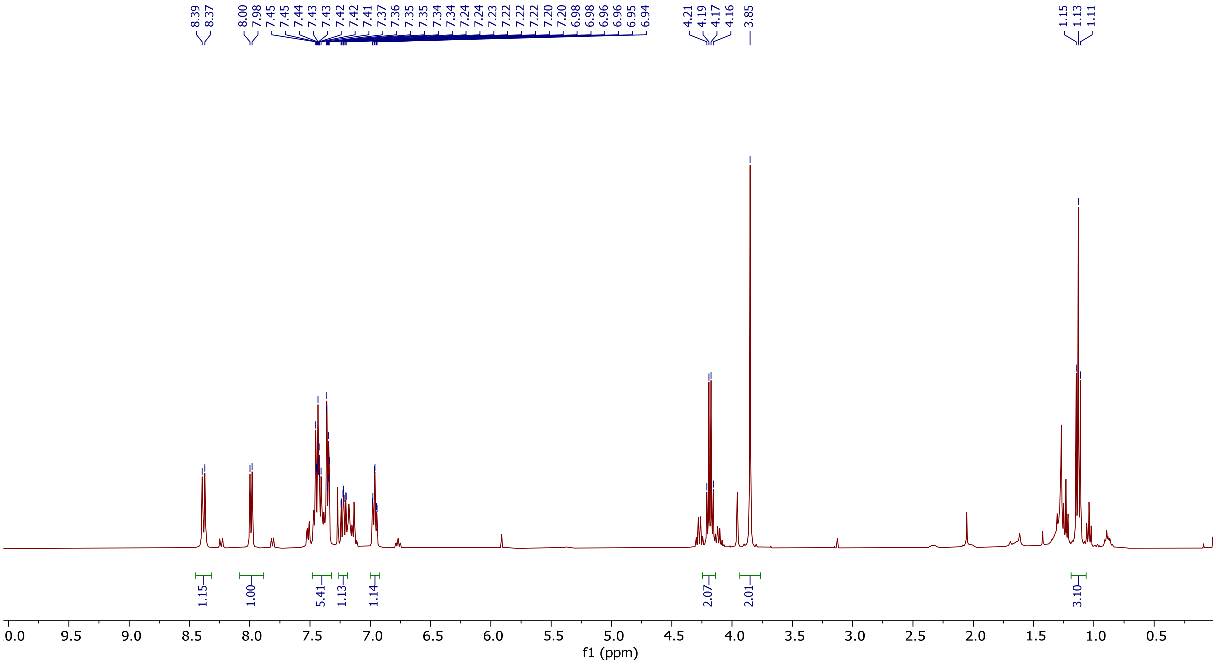

^{1}H NMR (400 MHz, $CDCl_3$) of **2d-10**.

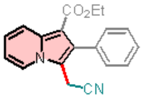

2d-10

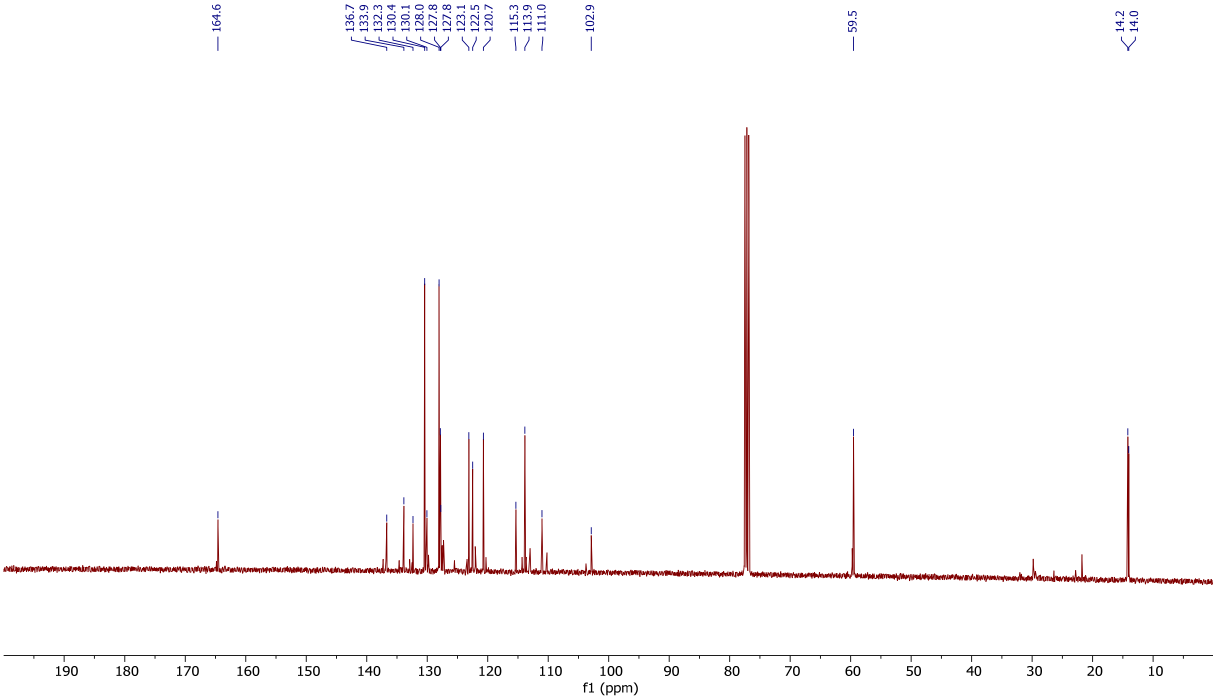

^{13}C{^{1}H} NMR (100 MHz, $CDCl_3$) of **2d-10**.

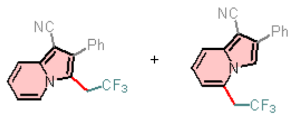

2a-11 + 2a-11' (4.5:1)

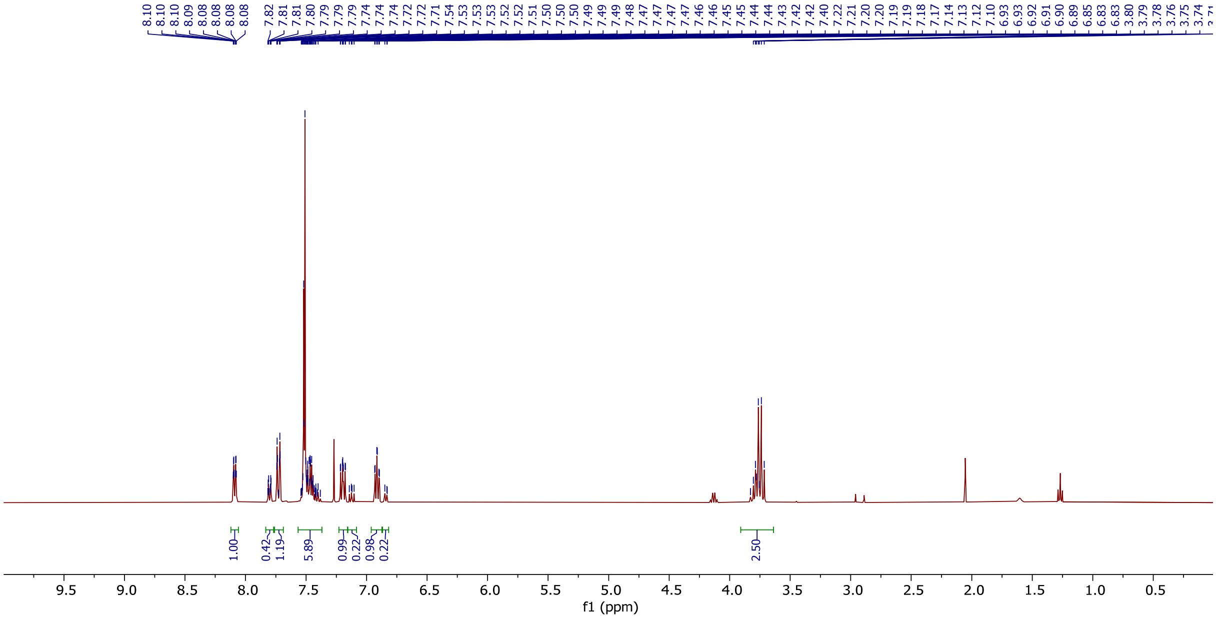

^{1}H NMR (400 MHz, $CDCl_3$) of **2a-11 + 2a-11'**.

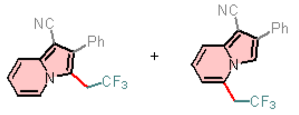

2a-11 + 2a-11' (4.5:1)

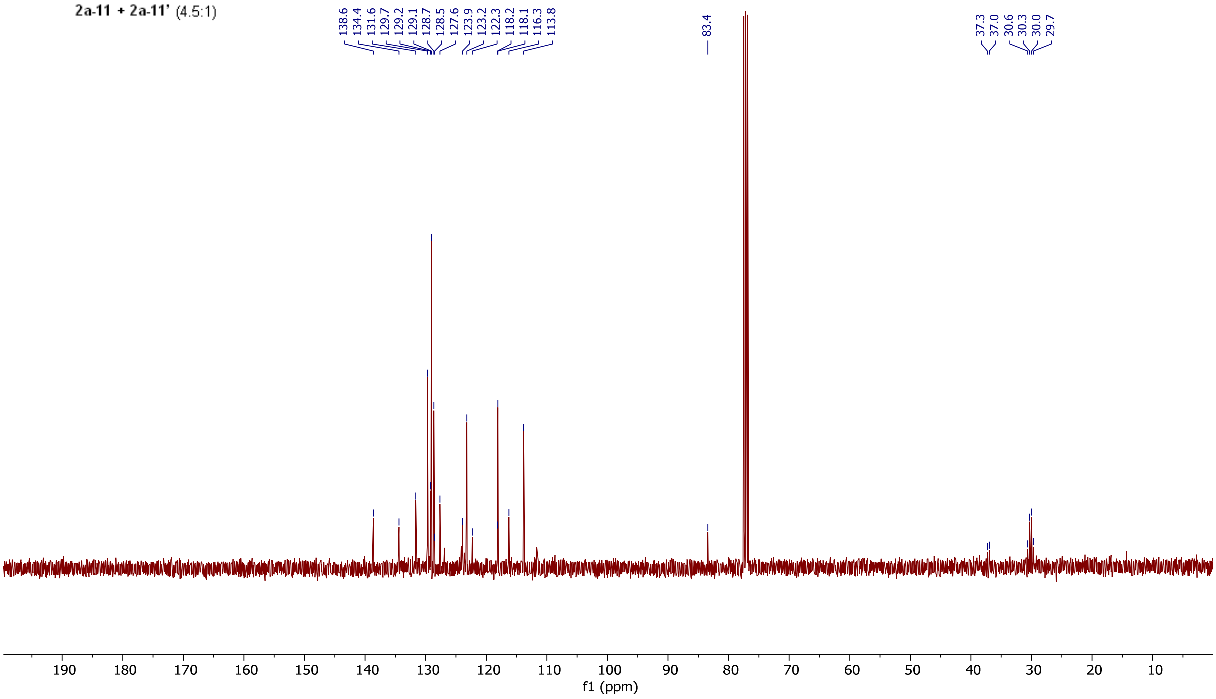

^{13}C{^{1}H} NMR (100 MHz, $CDCl_3$) of **2a-11 + 2a-11'**.

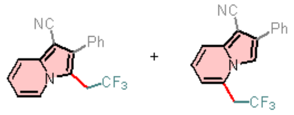

2a-11 + 2a-11' (4.5:1)

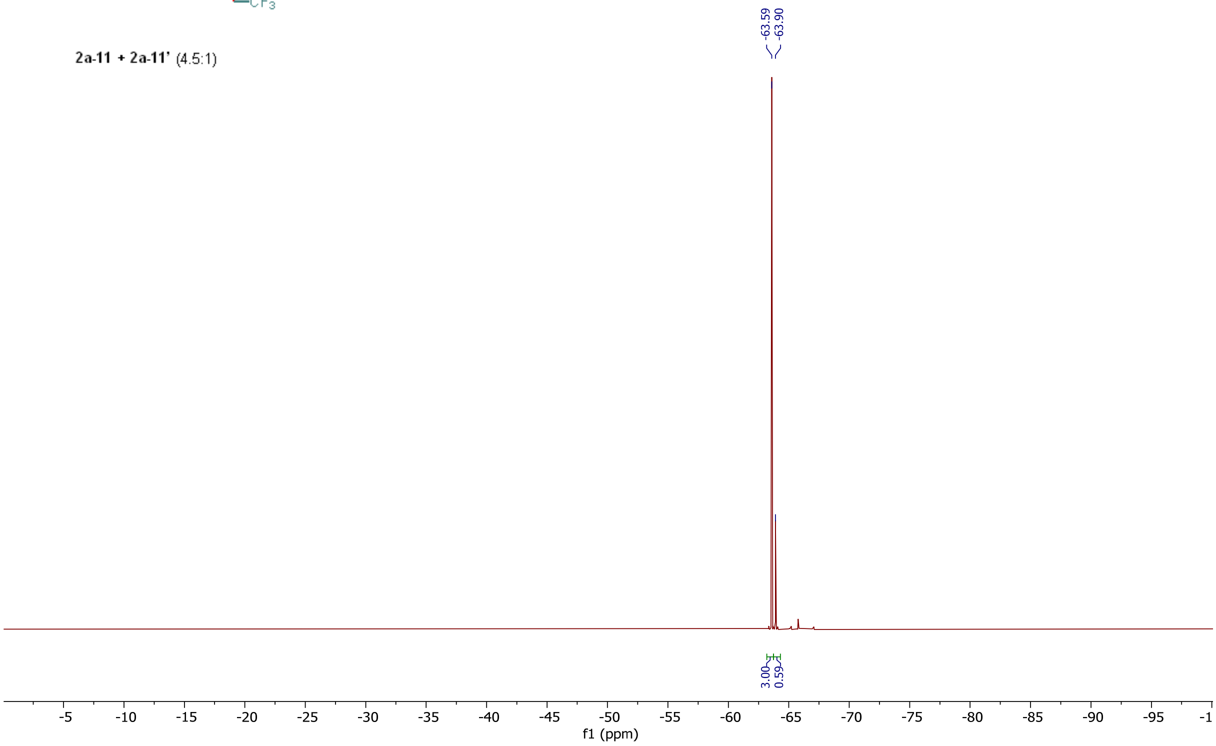

$^{19}F\{^1H\}$ NMR (377 MHz, $CDCl_3$) of **2a-11 + 2a-11'**.

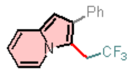

2b-11

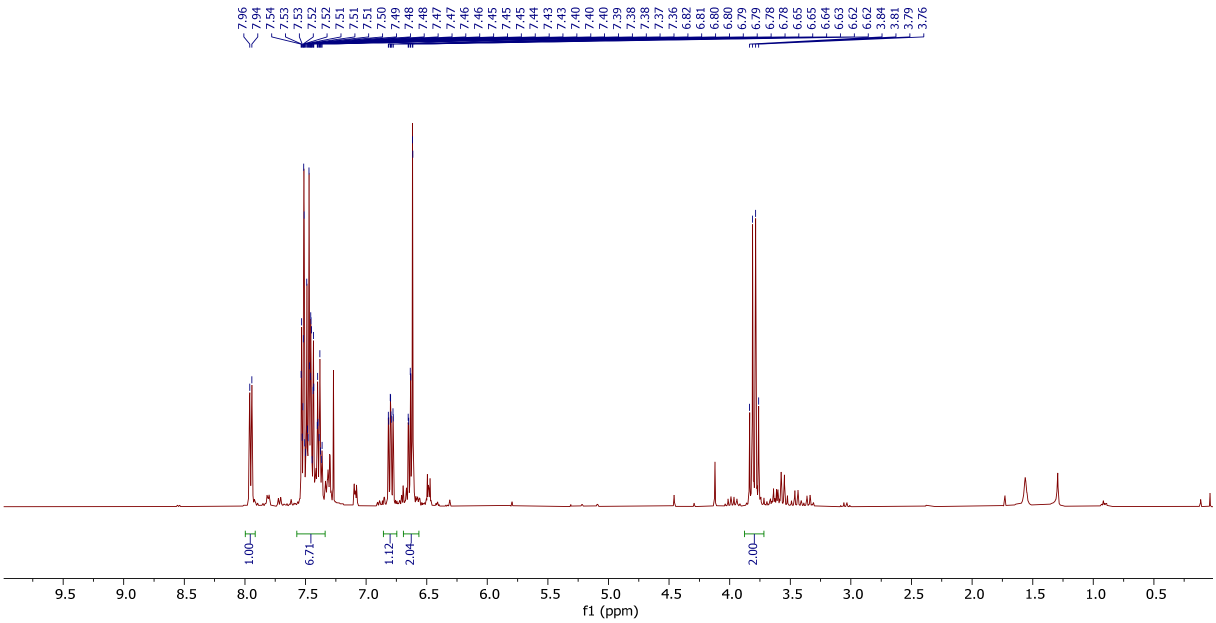

^1H NMR (400 MHz, $CDCl_3$) of **2b-11**.

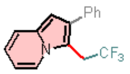

2b-11

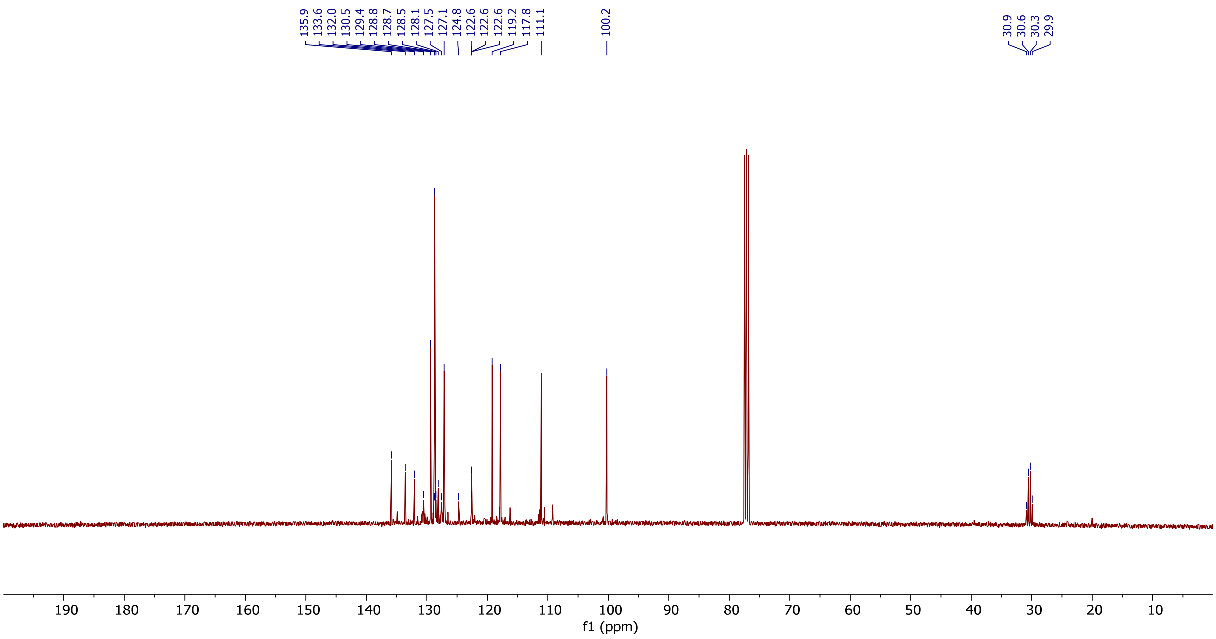

$^{13}C\{^{1}H\}$ NMR (100 MHz, $CDCl_3$) of **2b-11**.

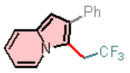

2b-11

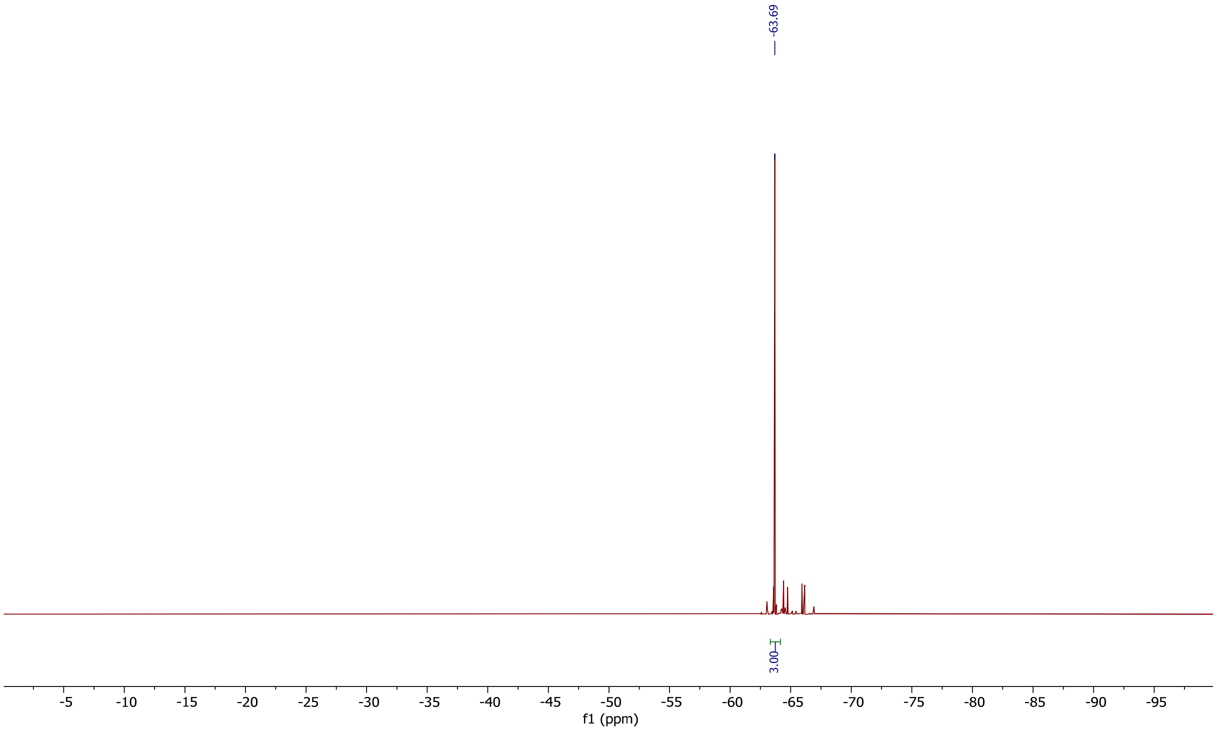

$^{19}F\{^{1}H\}$ NMR (377 MHz, $CDCl_3$) of **2b-11**.

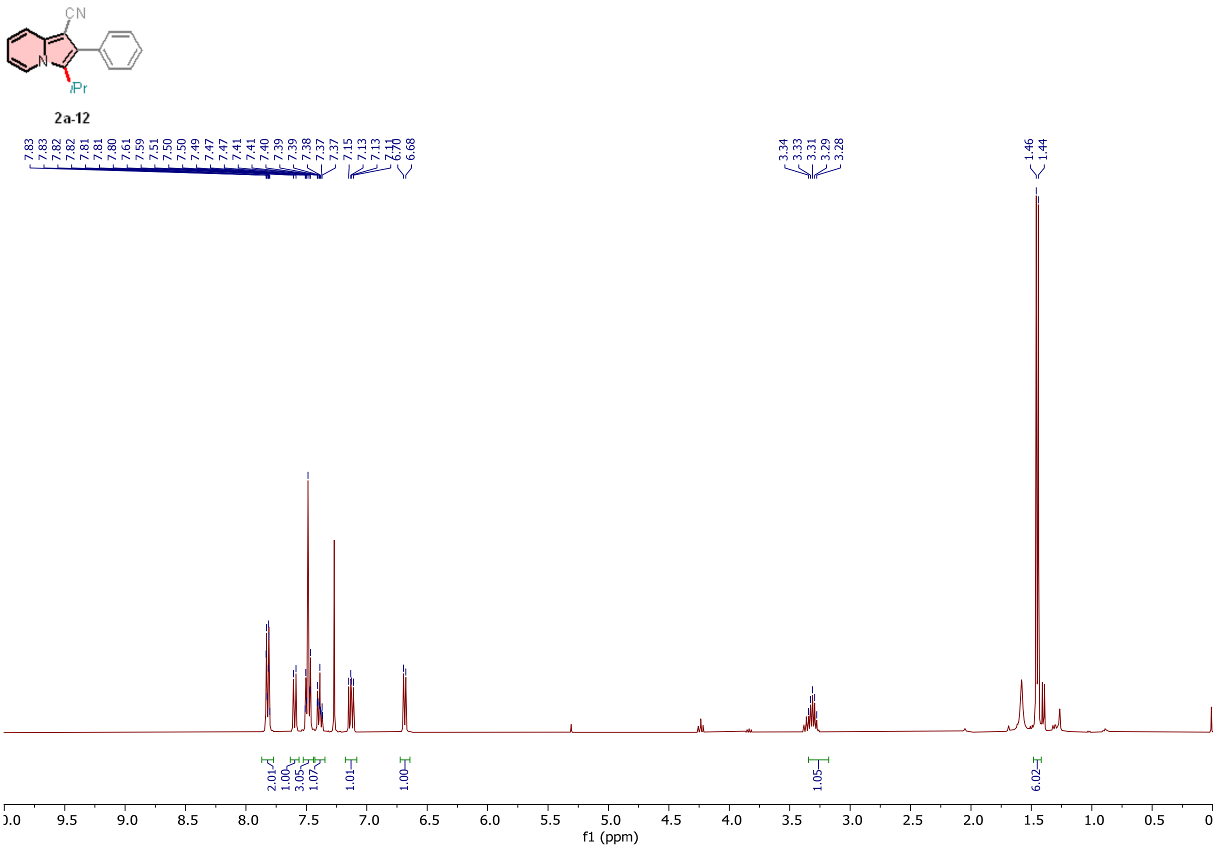

^{1}H NMR (400 MHz, $CDCl_3$) of **2a-12**.

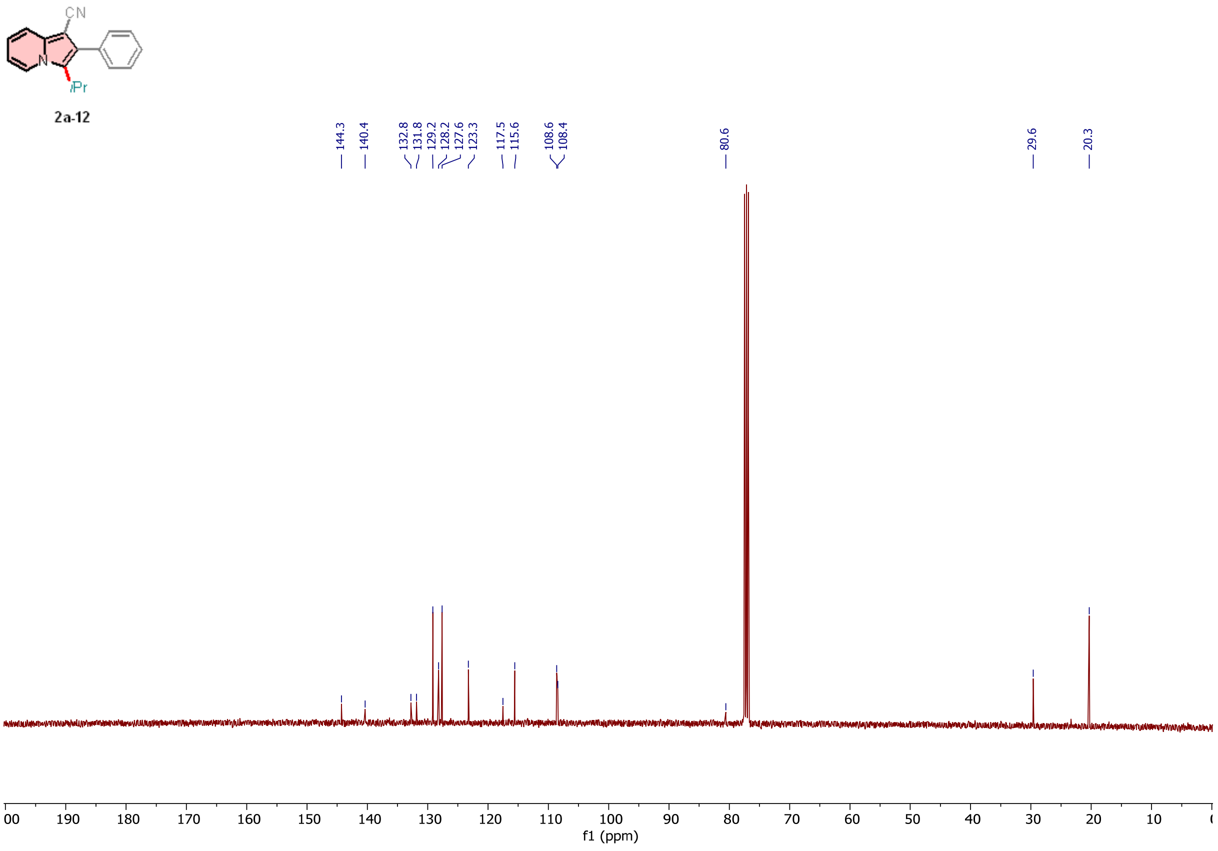

^{13}C{^{1}H} NMR (100 MHz, $CDCl_3$) of **2a-12**.

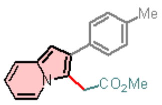

2e

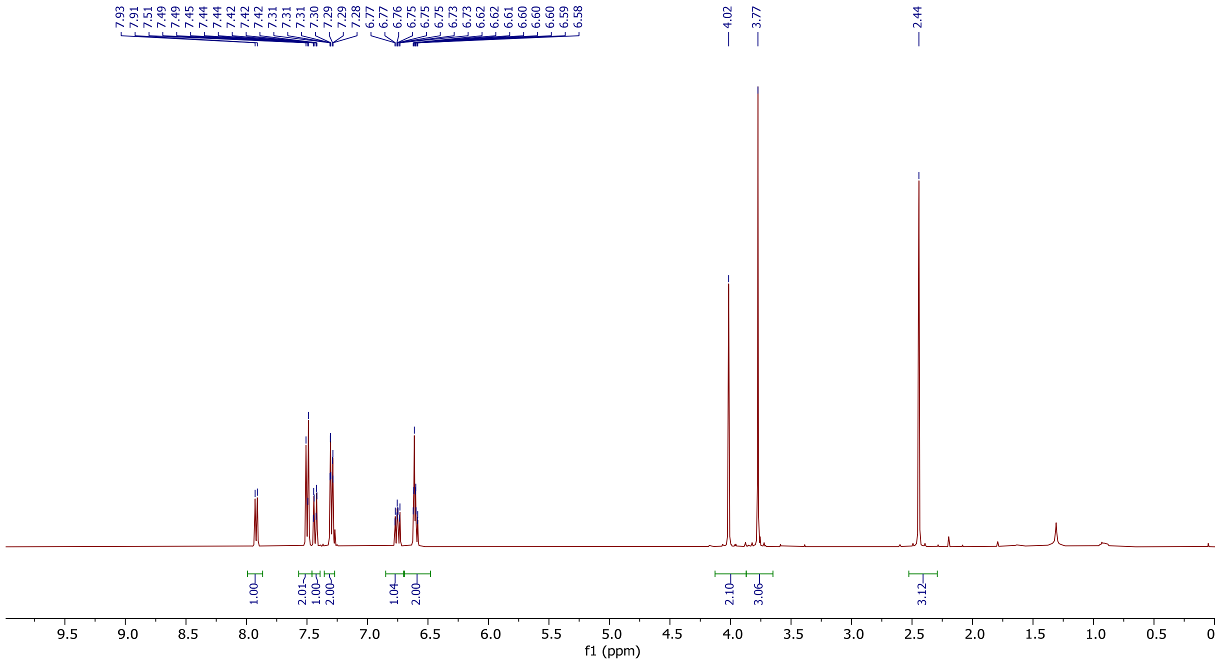

^{1}H NMR (400 MHz, $CDCl_3$) of **2e.**

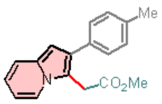

2e

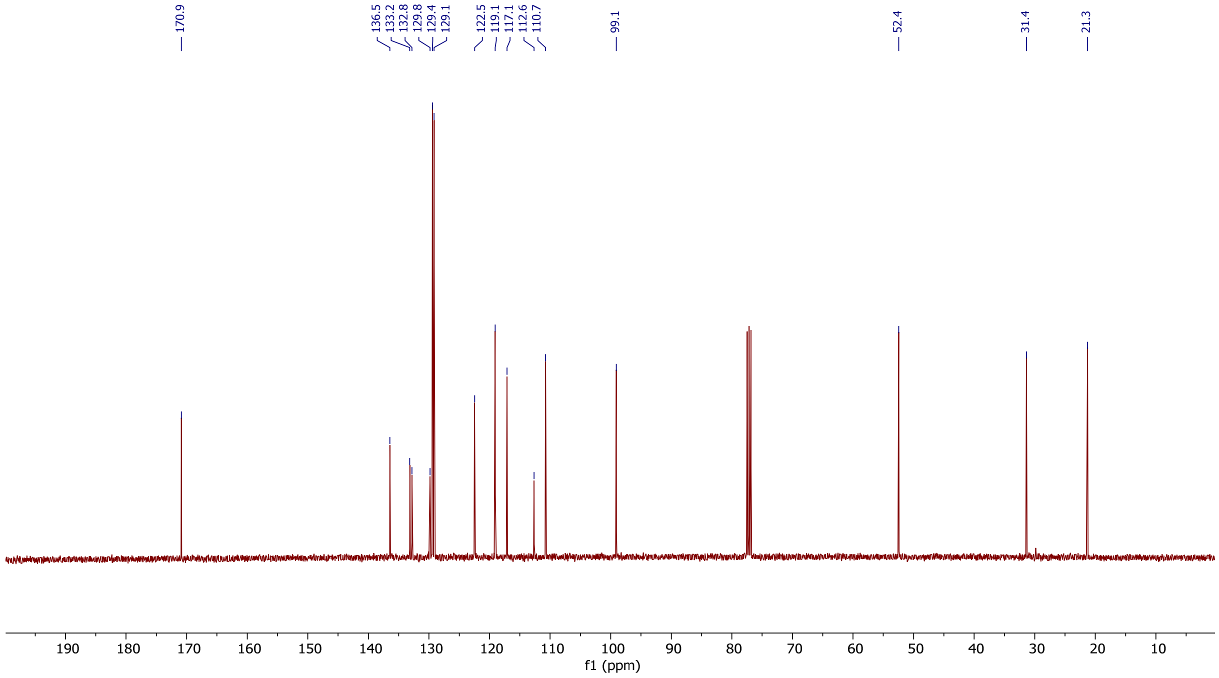

$^{13}C\{^{1}H\}$ NMR (100 MHz, $CDCl_3$) of **2e**.

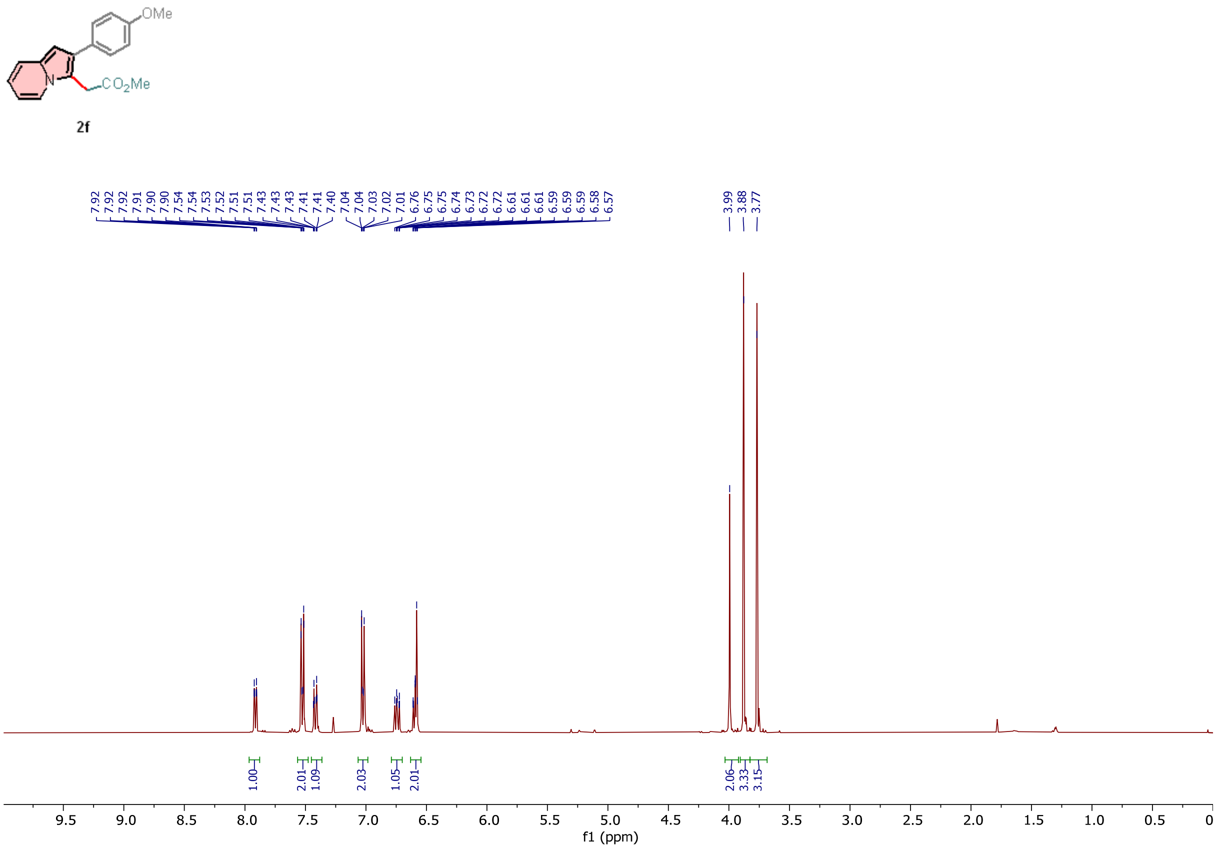

^{1}H NMR (400 MHz, $CDCl_3$) of **2f**.

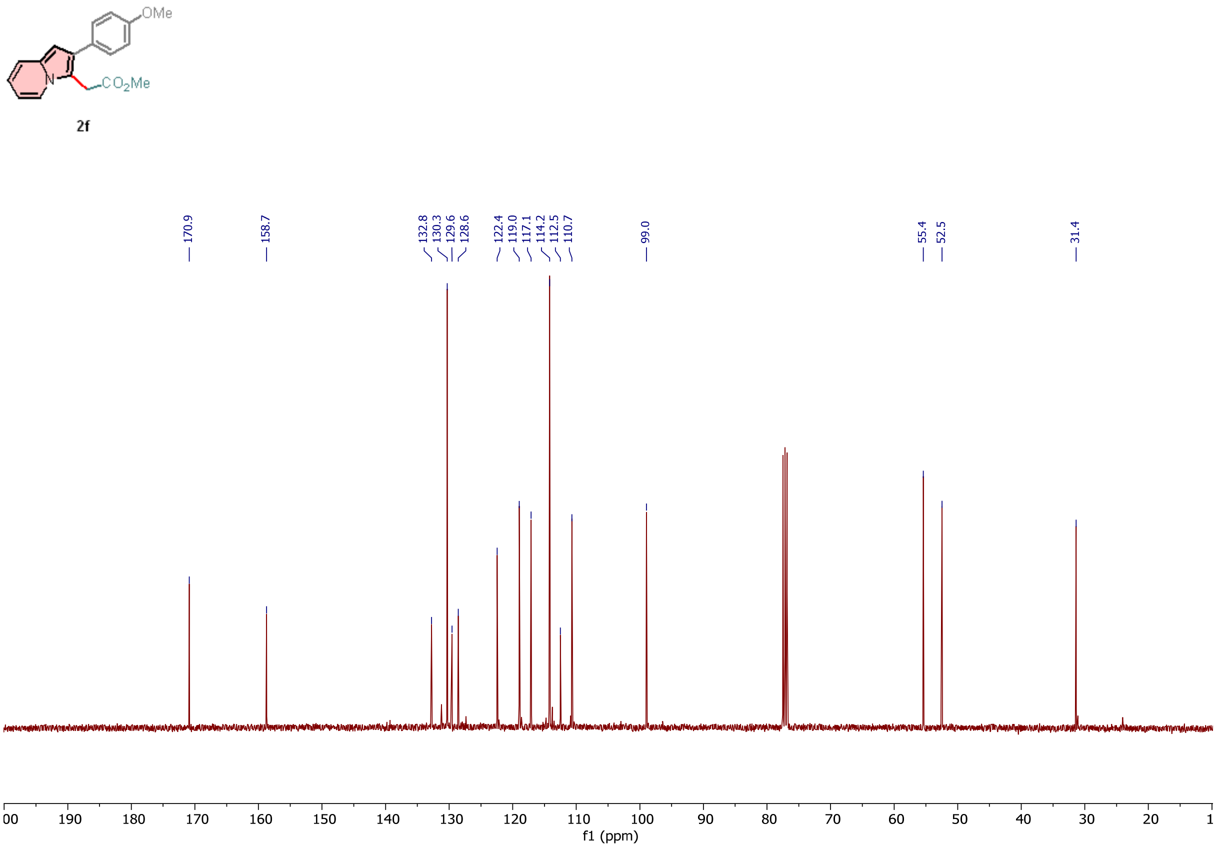

$^{13}C\{^{1}H\}$ NMR (100 MHz, $CDCl_3$) of **2f**.

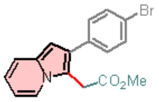

2g

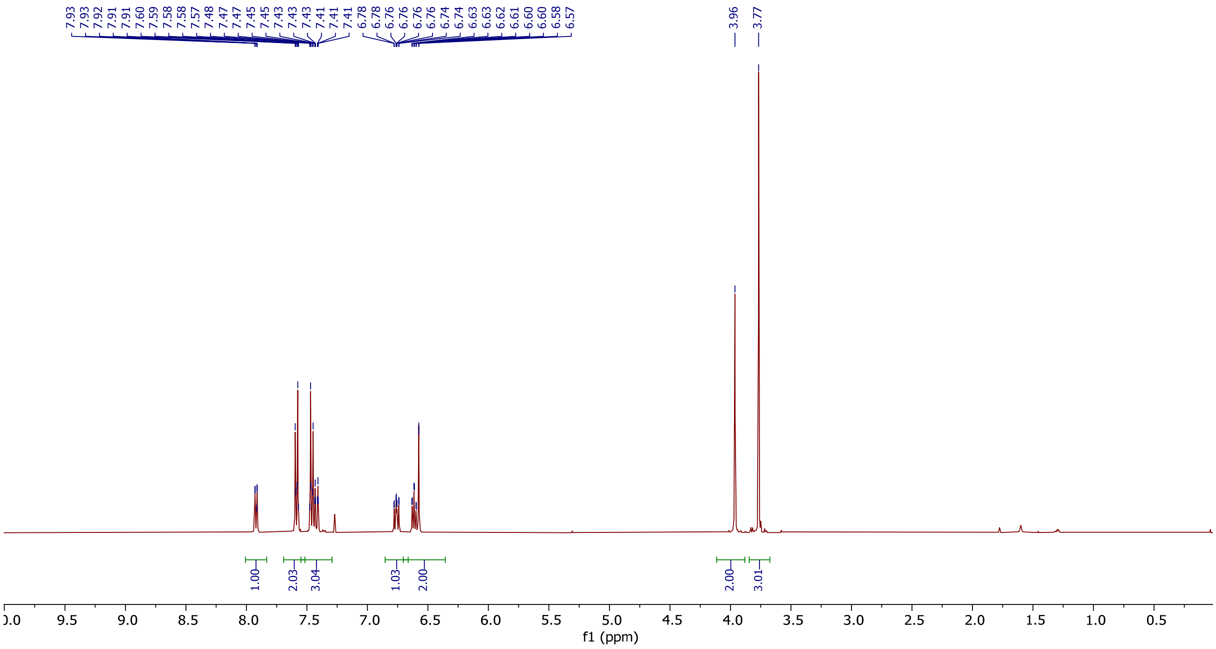

^{1}H NMR (400 MHz, $CDCl_3$) of **2g**.

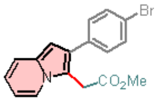

2g

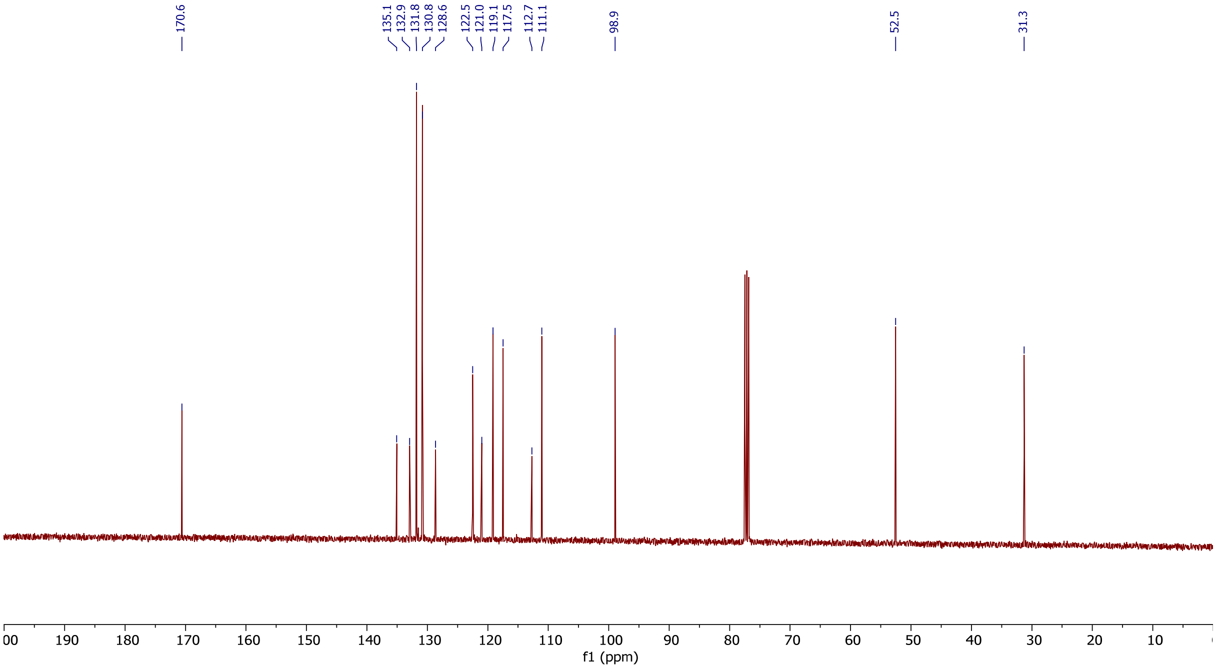

^{13}C{^{1}H} NMR (100 MHz, $CDCl_3$) of **2g**.

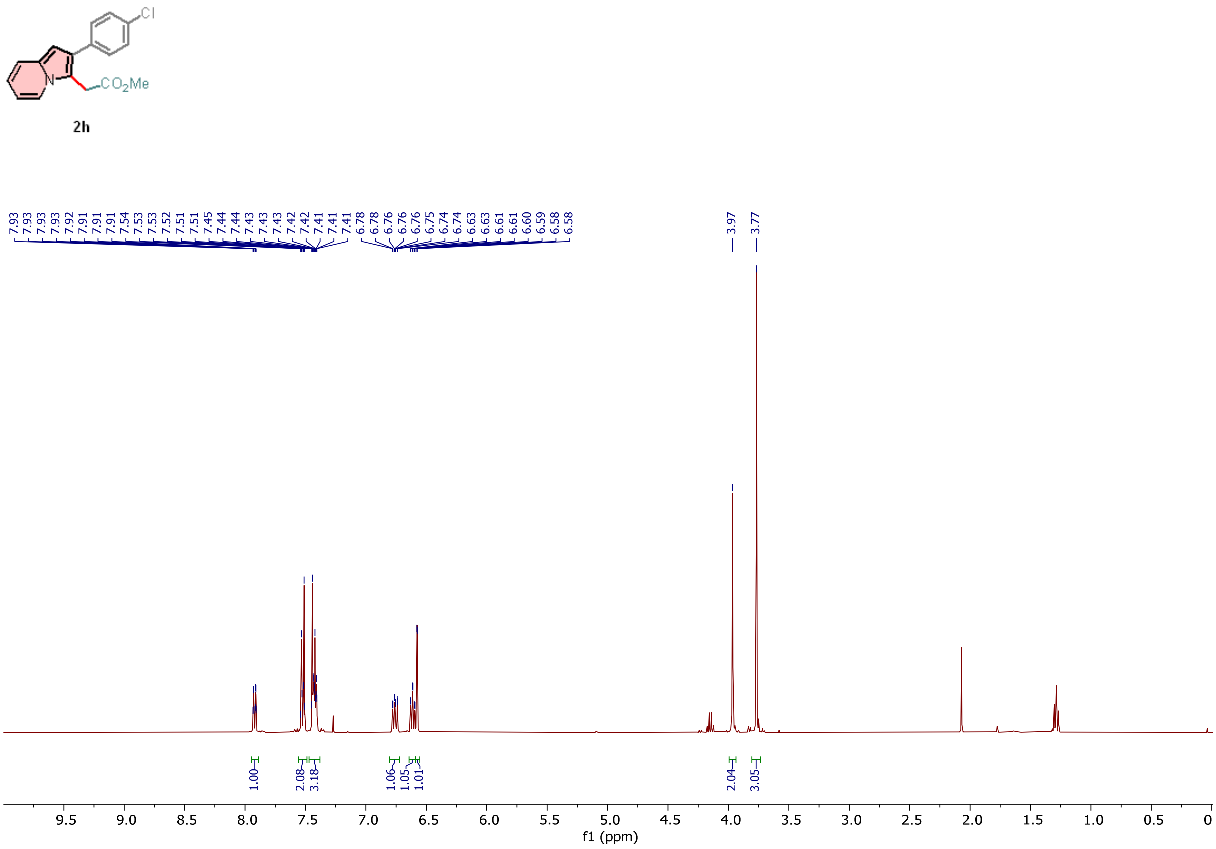

^1H NMR (400 MHz, $CDCl_3$) of **2h**.

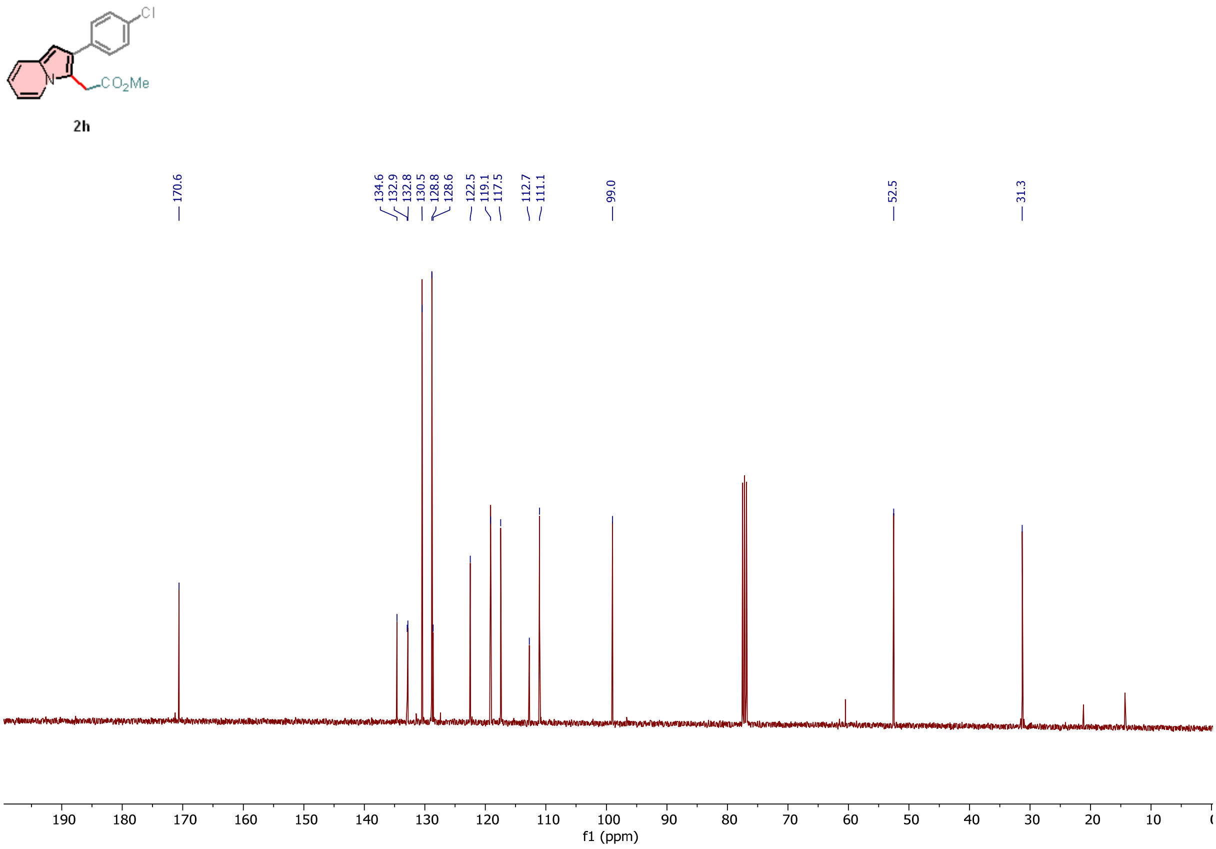

$^{13}C\{^1H\}$ NMR (100 MHz, $CDCl_3$) of **2h**.

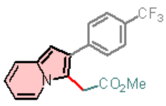

2i

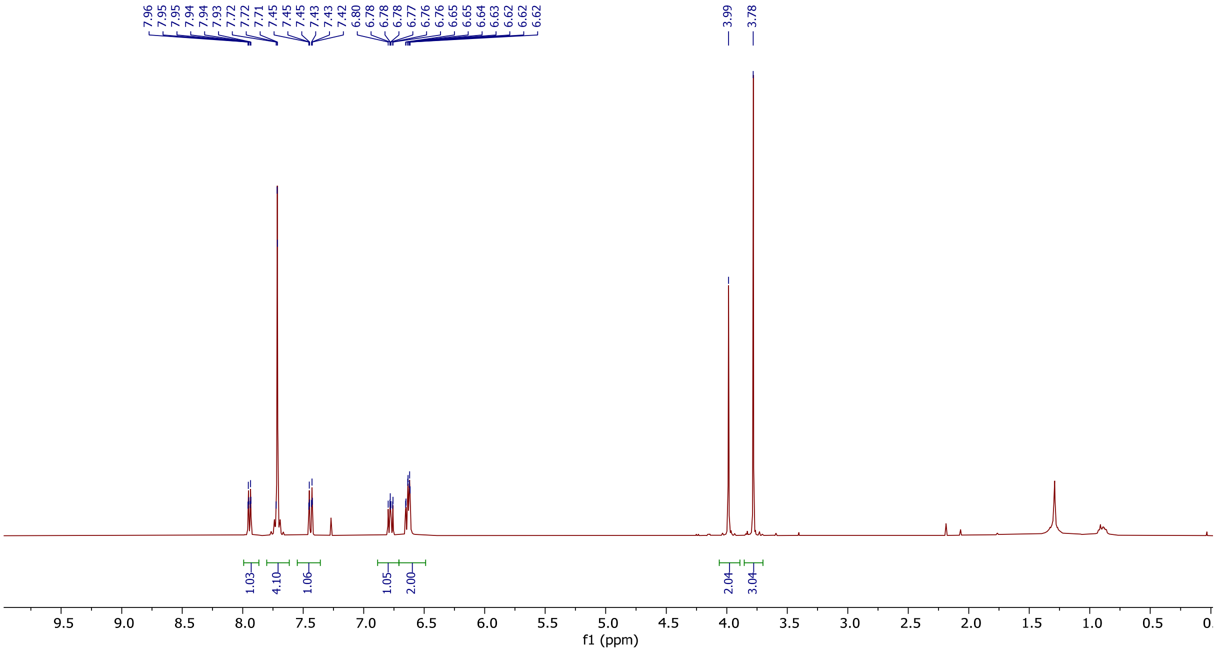

^{1}H NMR (400 MHz, $CDCl_3$) of **2i.**

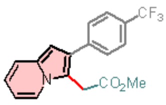

2i

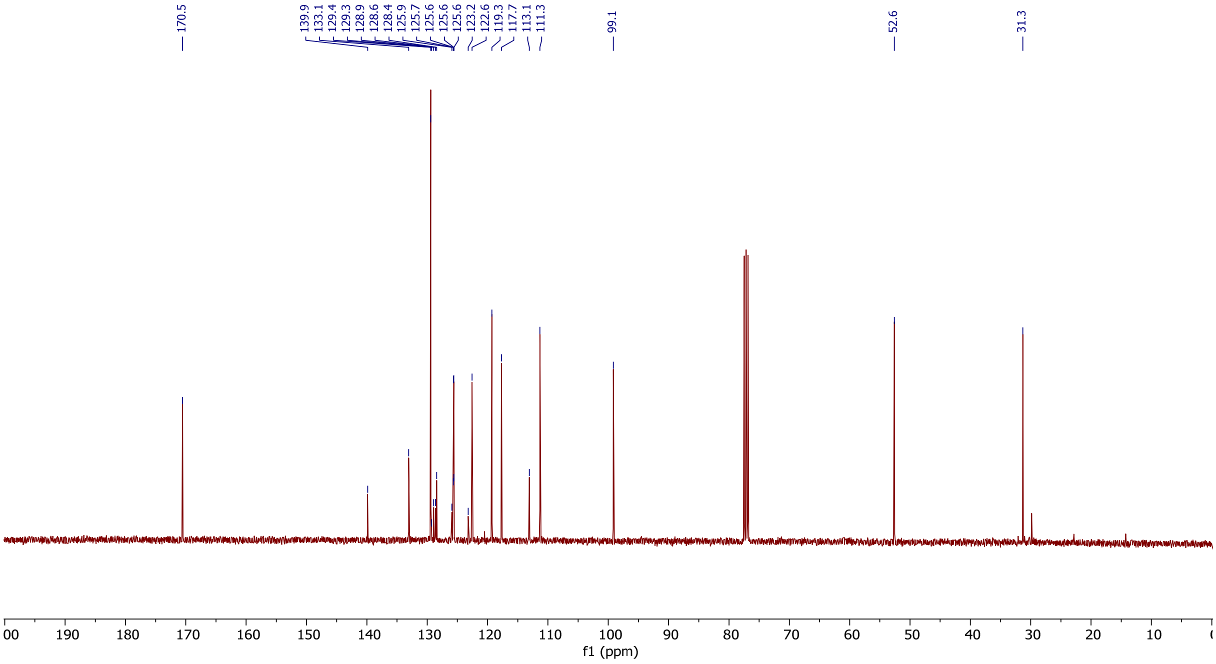

$^{13}C\{^{1}H\}$ NMR (100 MHz, $CDCl_3$) of **2i**.

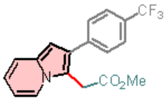

2i

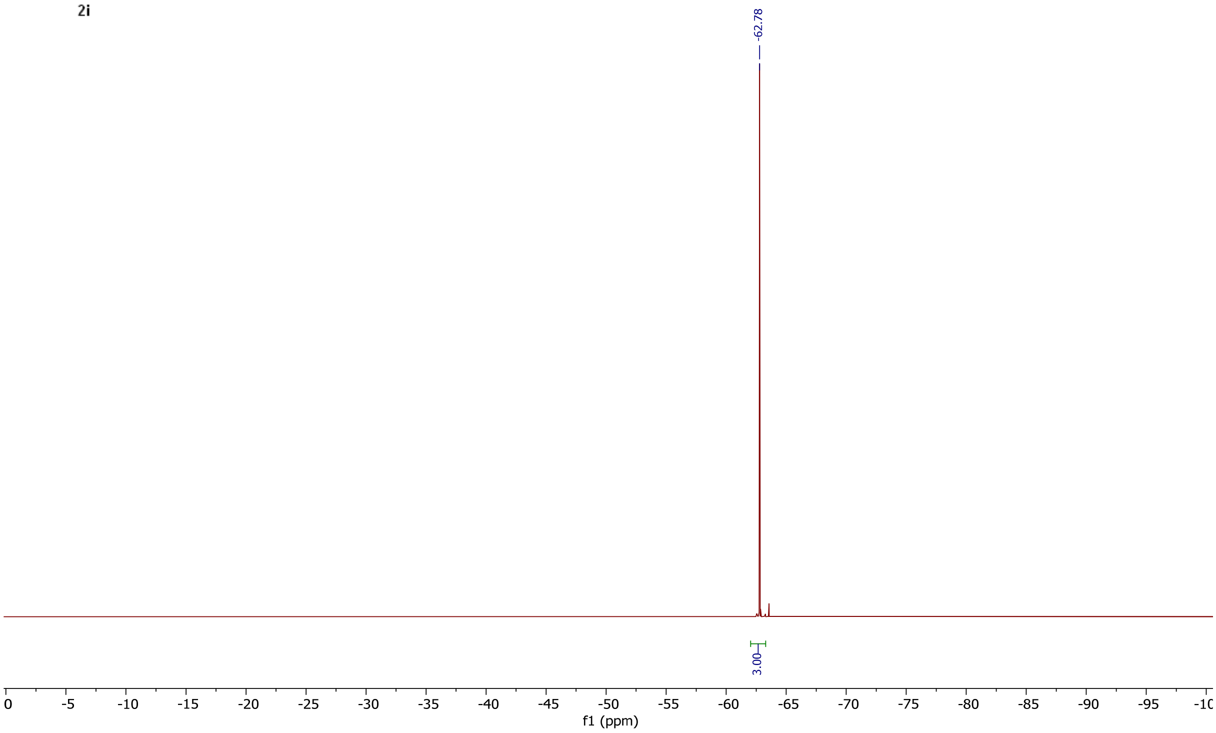

$^{19}F\{^{1}H\}$ NMR (377 MHz, $CDCl_3$) of **2i**.

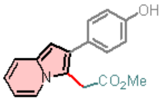

2j

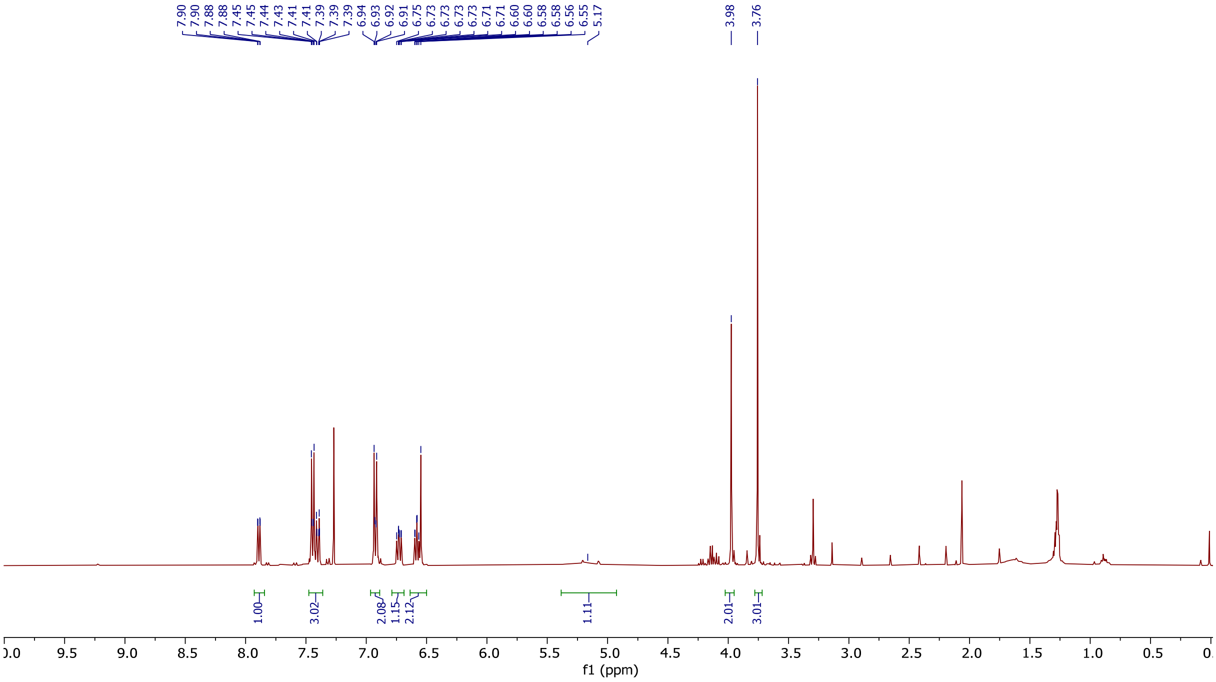

^{1}H NMR (400 MHz, $CDCl_3$) of **2j**.

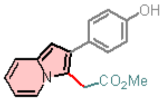

2j

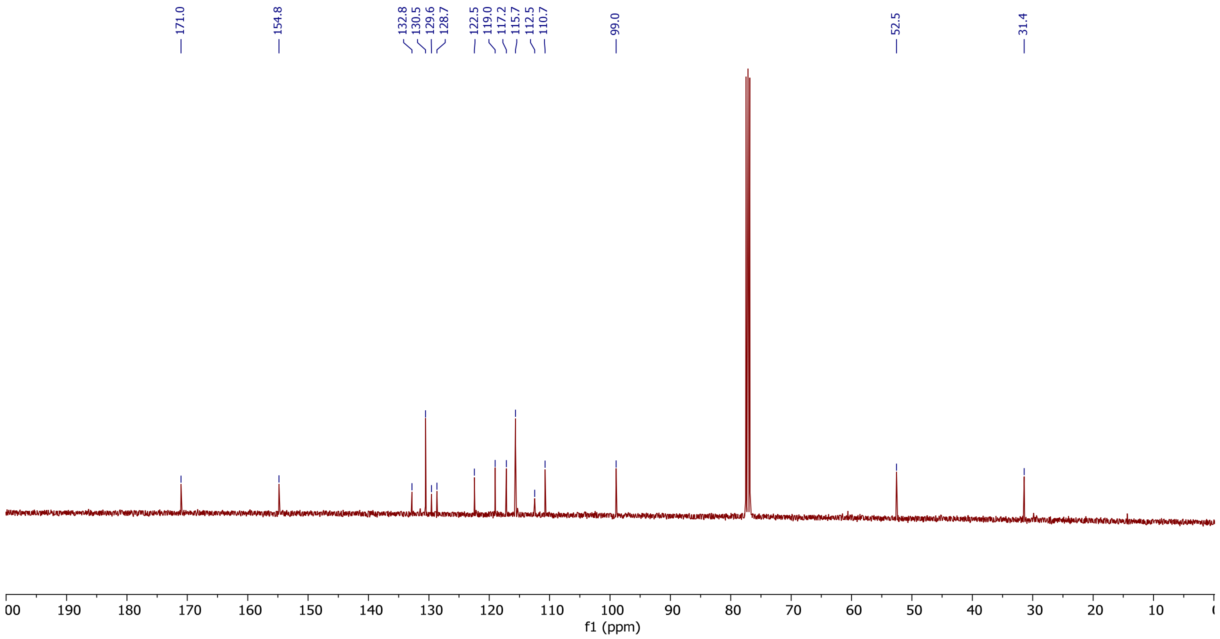

$^{13}C\{^{1}H\}$ NMR (100 MHz, $CDCl_3$) of **2j**.

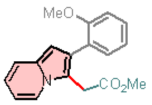

2k

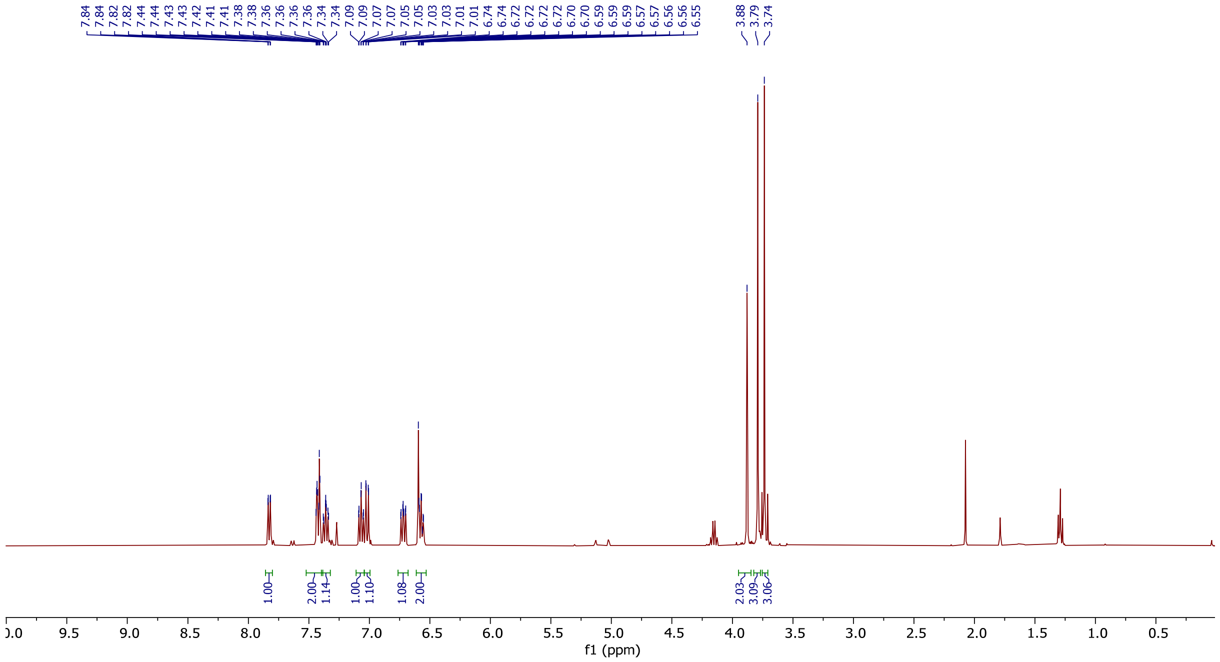

^{1}H NMR (400 MHz, $CDCl_3$) of **2k**.

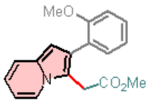

2k

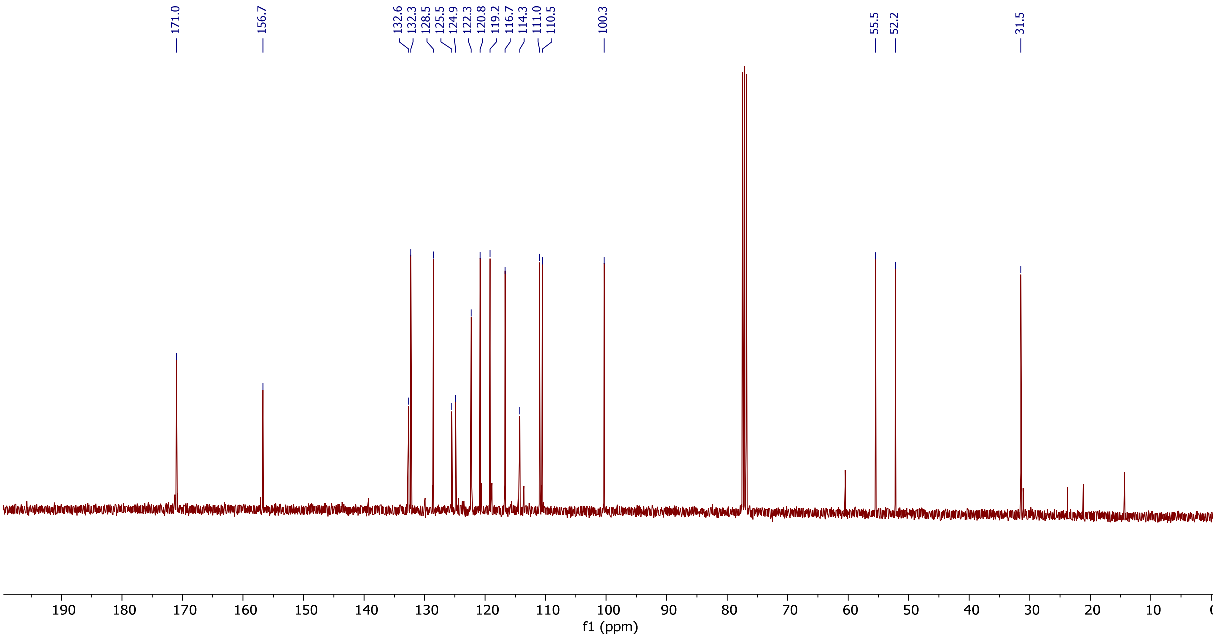

$^{13}C\{^{1}H\}$ NMR (100 MHz, $CDCl_3$) of **2k**.

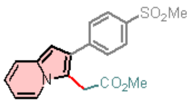

2l

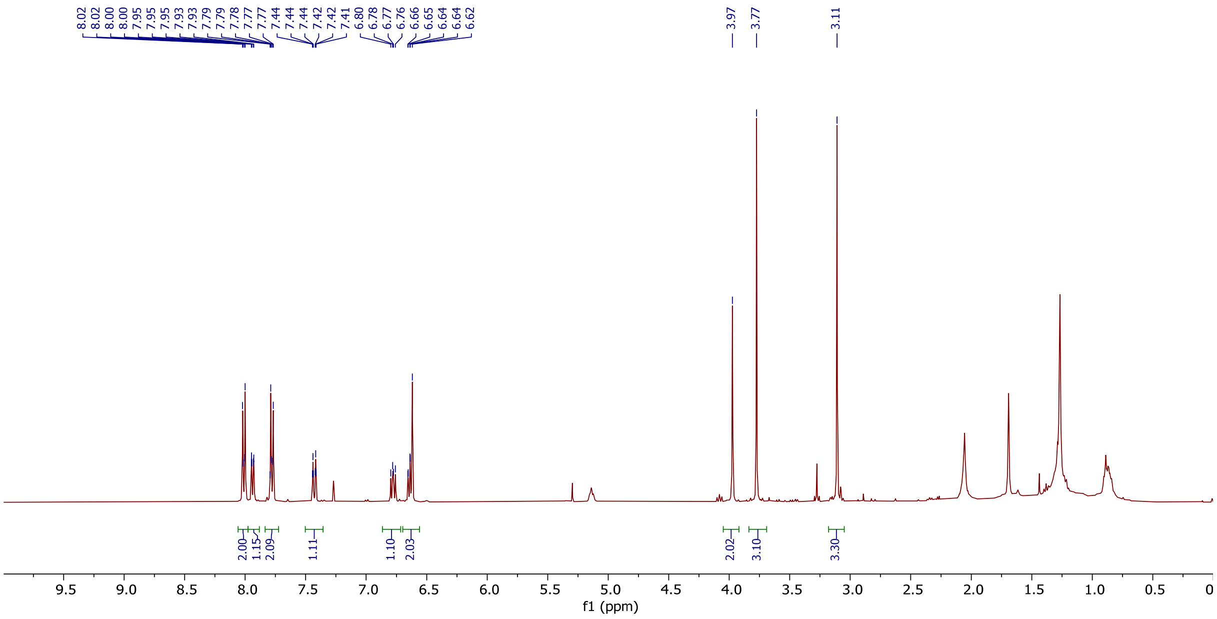

^{1}H NMR (400 MHz, $CDCl_3$) of **2l**.

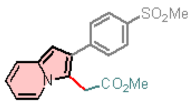

2l

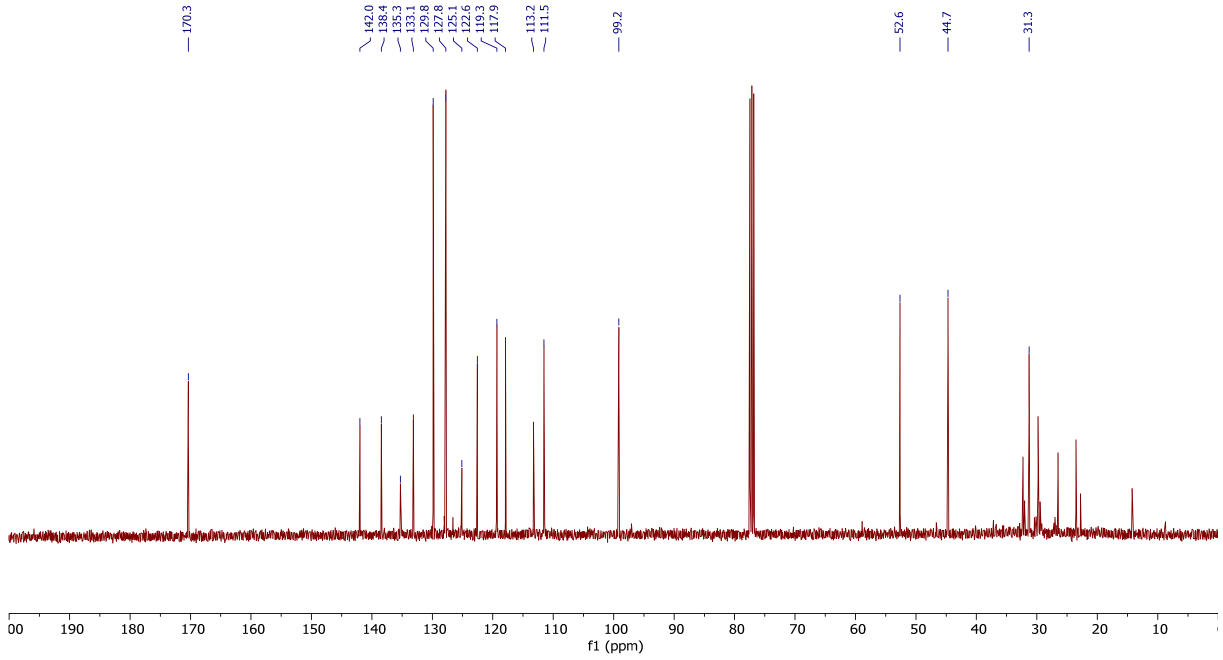

$^{13}C\{^{1}H\}$ NMR (100 MHz, $CDCl_3$) of **2l**.

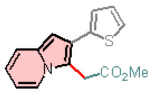

2m

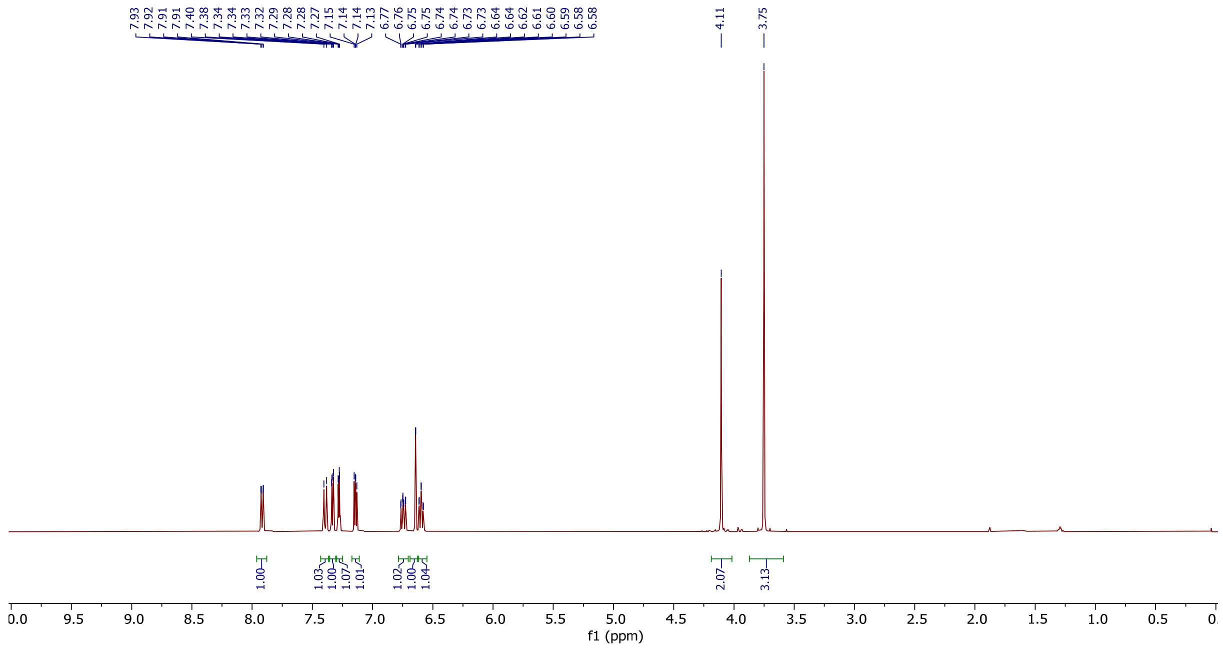

^{1}H NMR (400 MHz, $CDCl_3$) of **2m**.

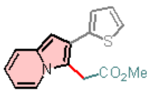

2m

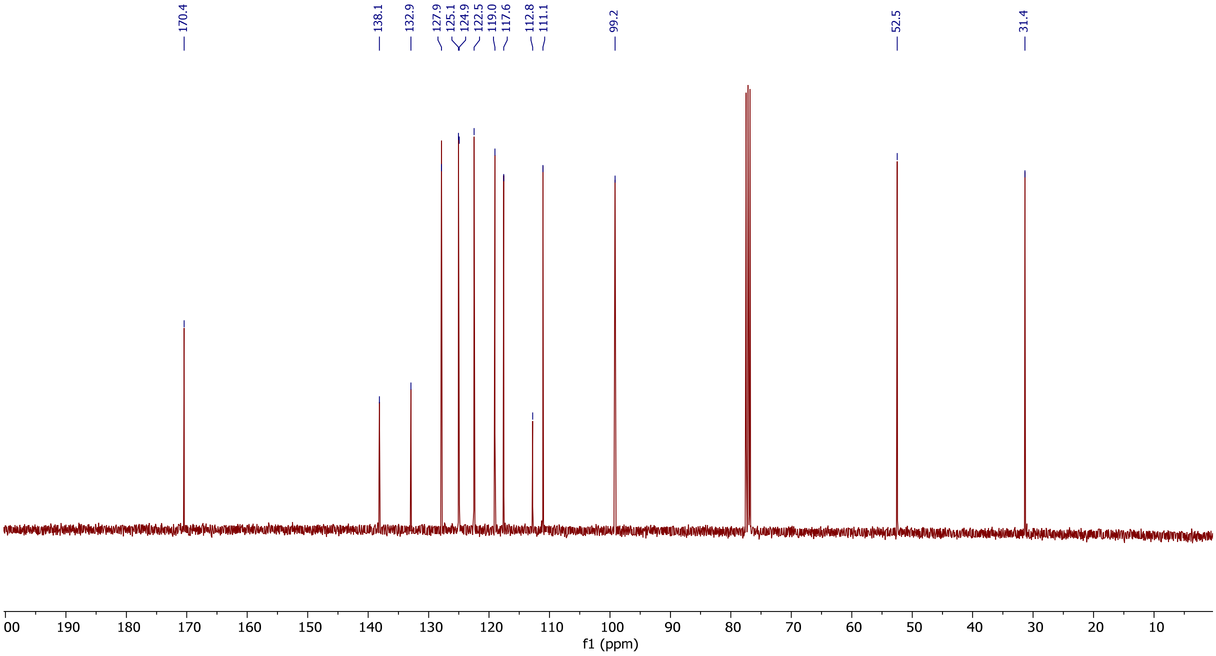

$^{13}C\{^{1}H\}$ NMR (100 MHz, $CDCl_3$) of **2m**.

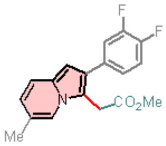

2n

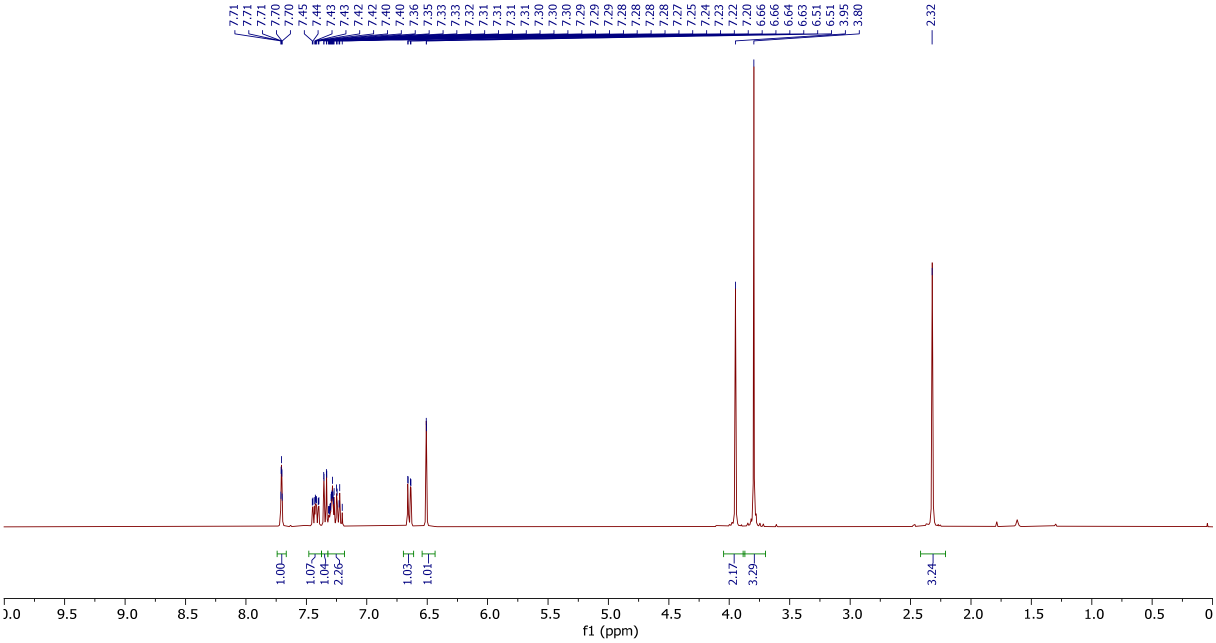

^{1}H NMR (400 MHz, $CDCl_3$) of **2n**.

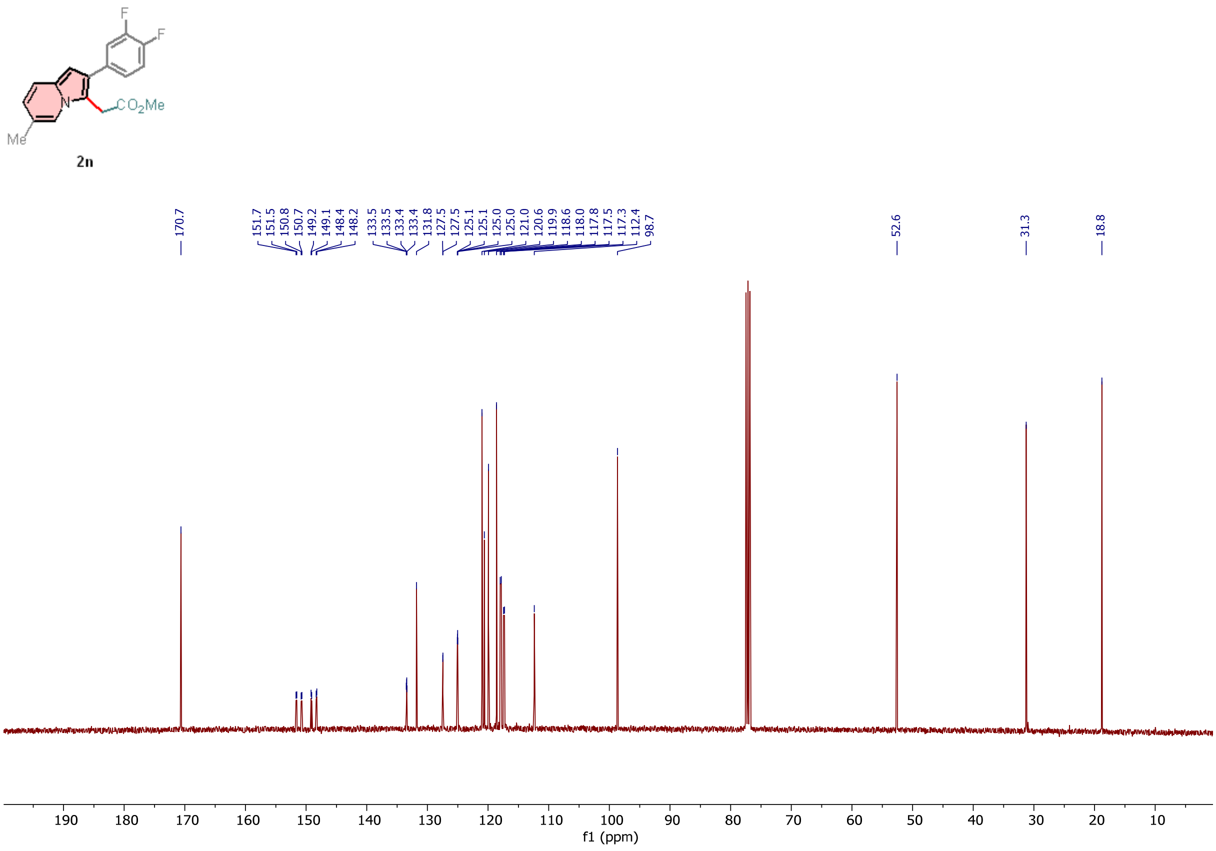

$^{13}C\{^1H\}$ NMR (100 MHz, $CDCl_3$) of **2n**.

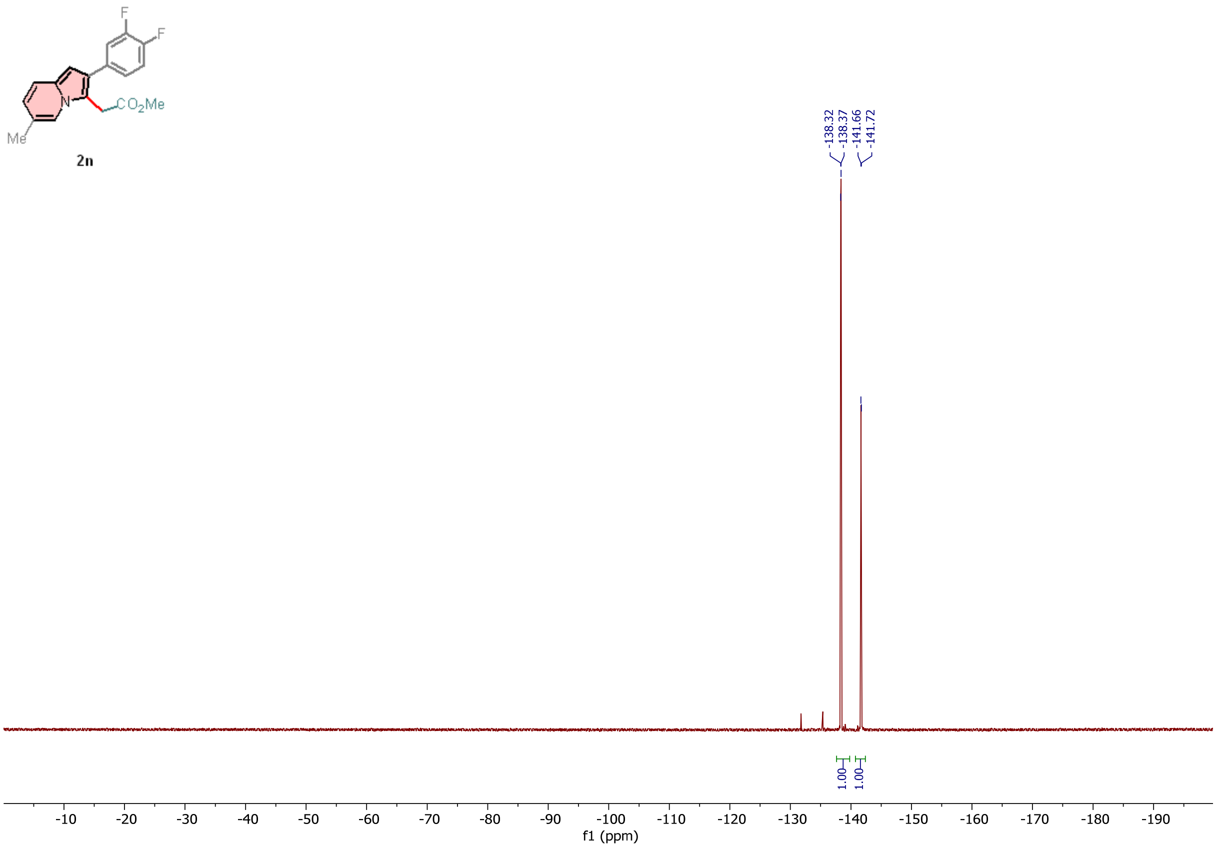

$^{19}F\{^1H\}$ NMR (377 MHz, $CDCl_3$) of **2n**.

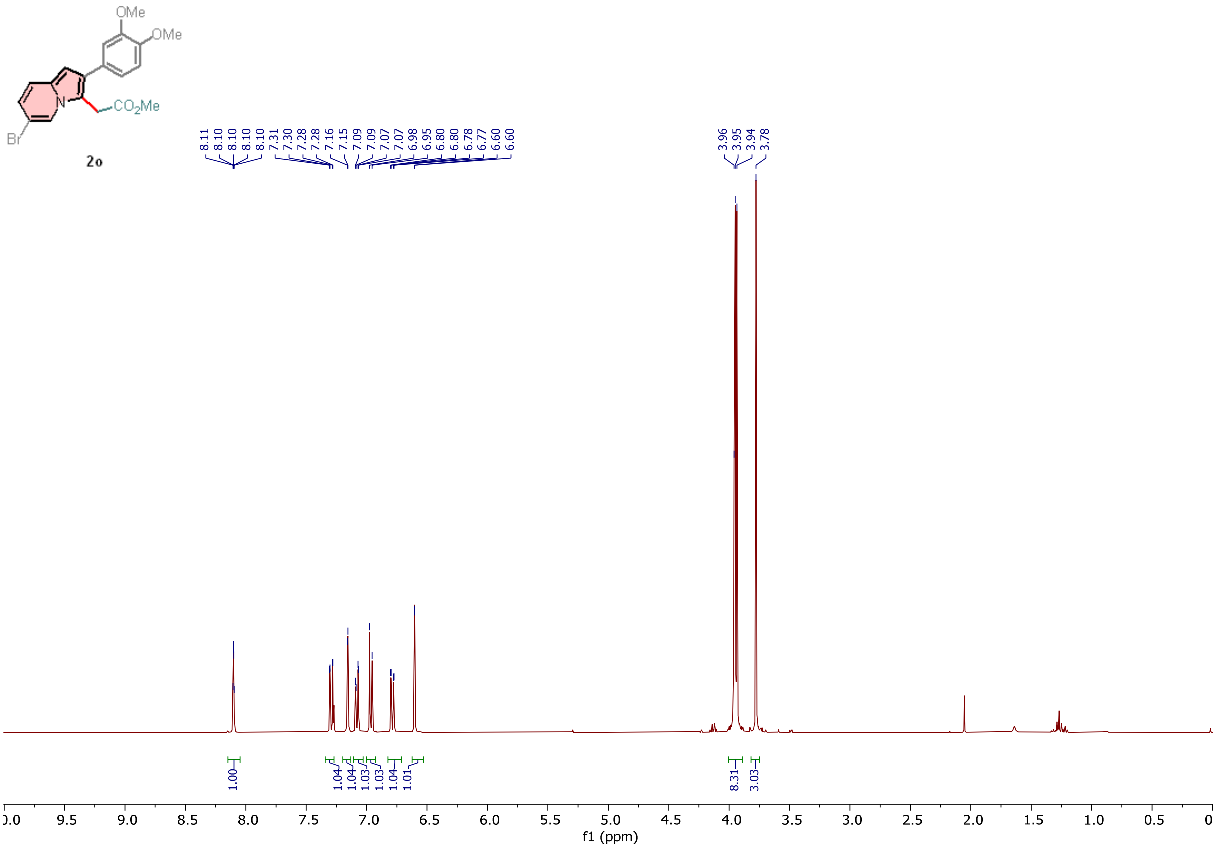

^{1}H NMR (400 MHz, $CDCl_3$) of **2o**.

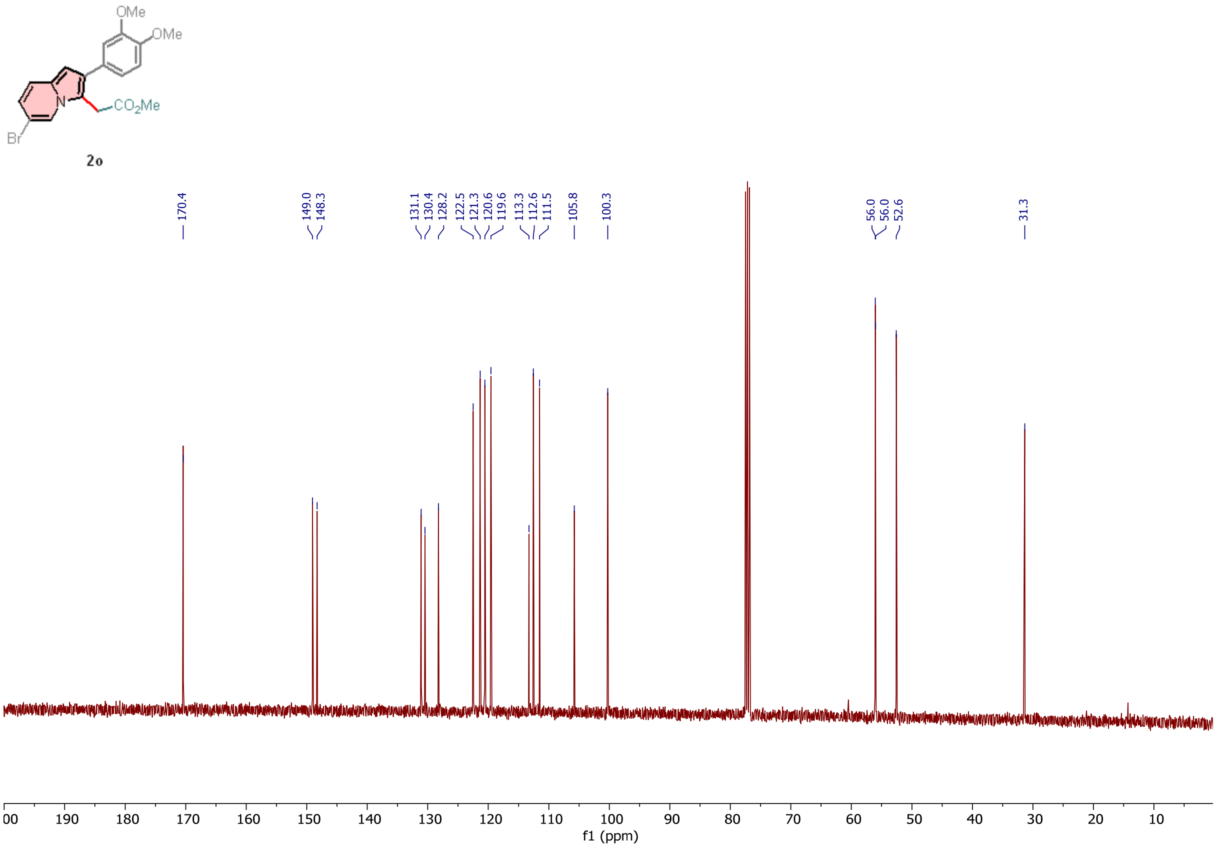

^{13}C{^{1}H} NMR (100 MHz, $CDCl_3$) of **2o**.

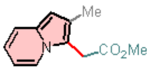

2p

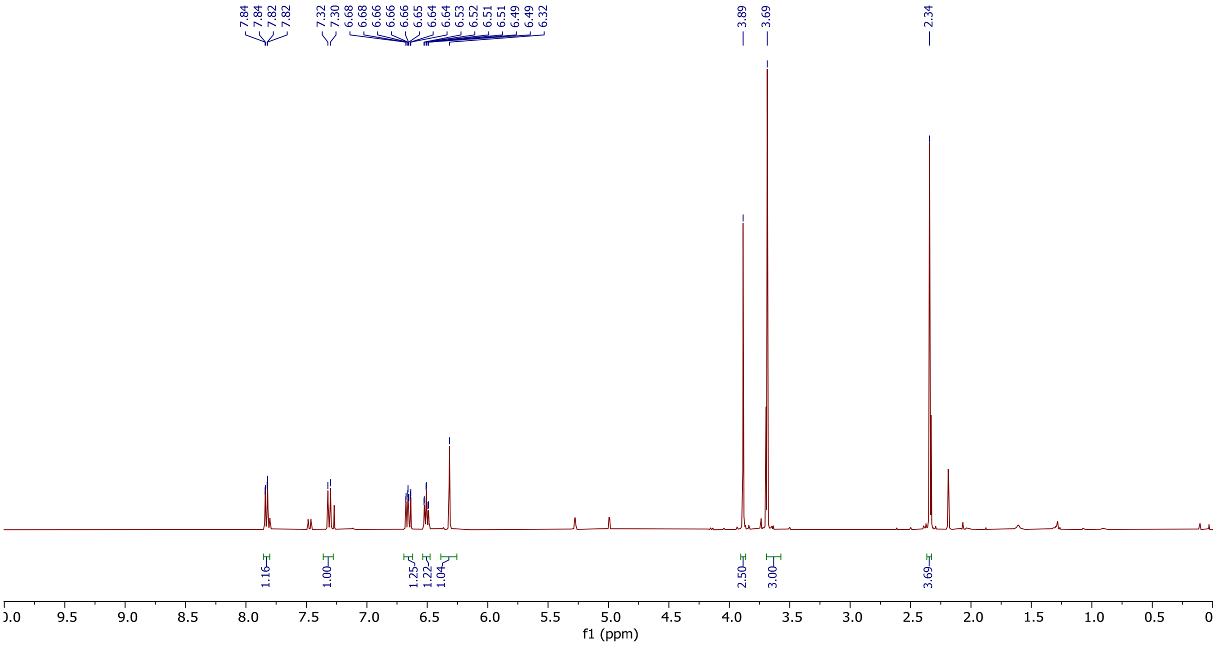

^{1}H NMR (400 MHz, $CDCl_3$) of **2p**.

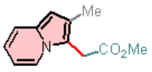

2p

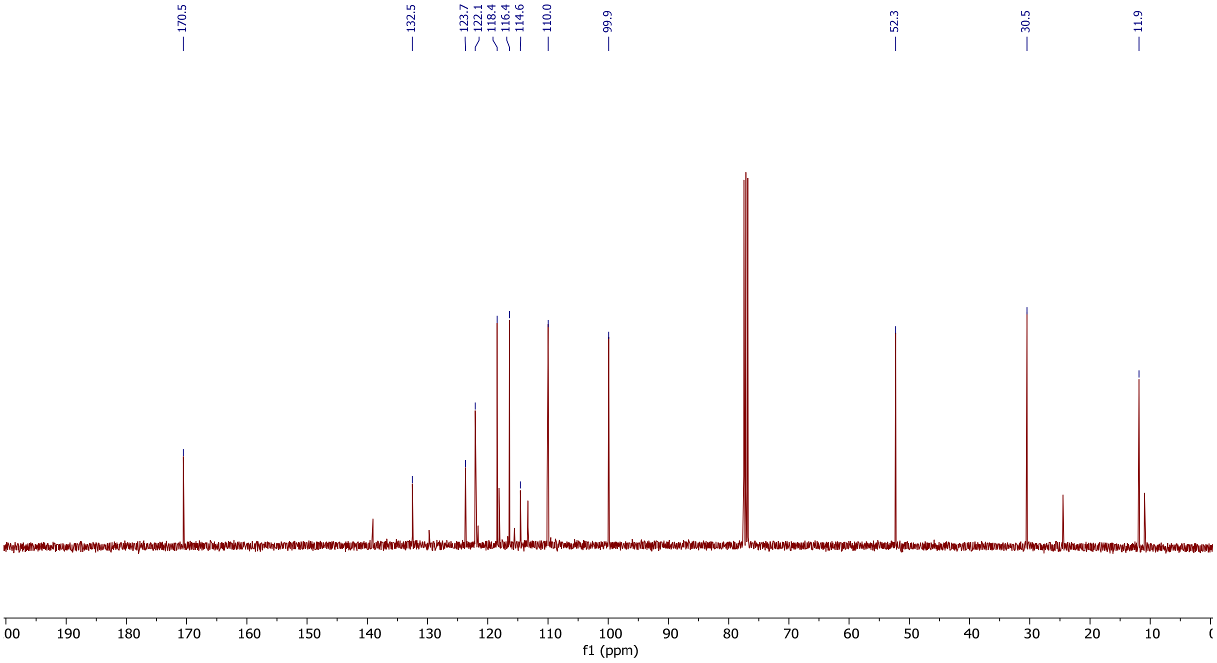

$^{13}C\{^{1}H\}$ NMR (100 MHz, $CDCl_3$) of **2p**.

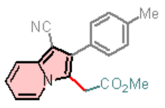

2q

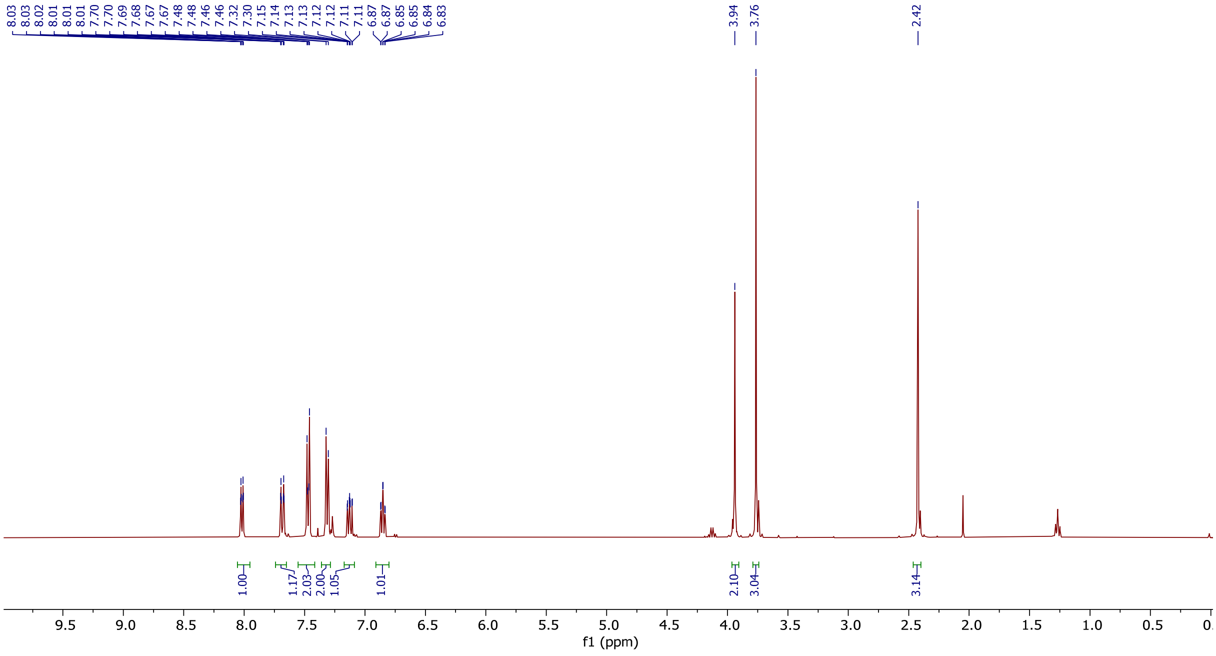

^{1}H NMR (400 MHz, $CDCl_3$) of **2q**.

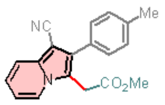

2q

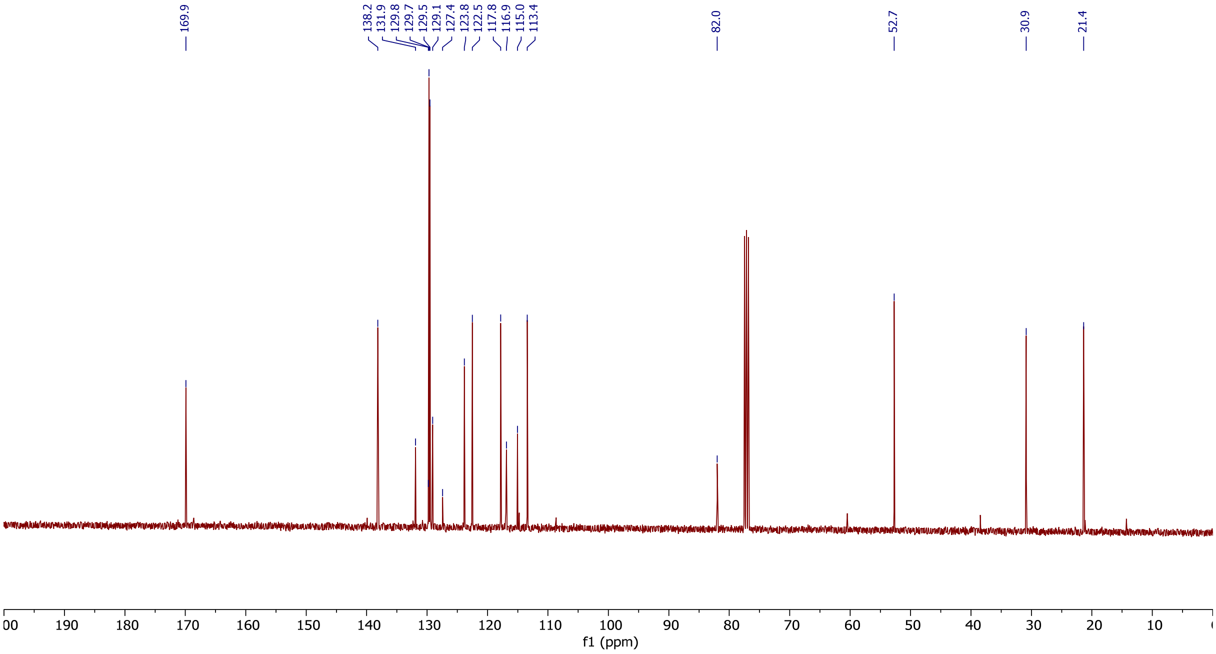

$^{13}C\{^{1}H\}$ NMR (100 MHz, $CDCl_3$) of **2q**.

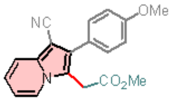

2r

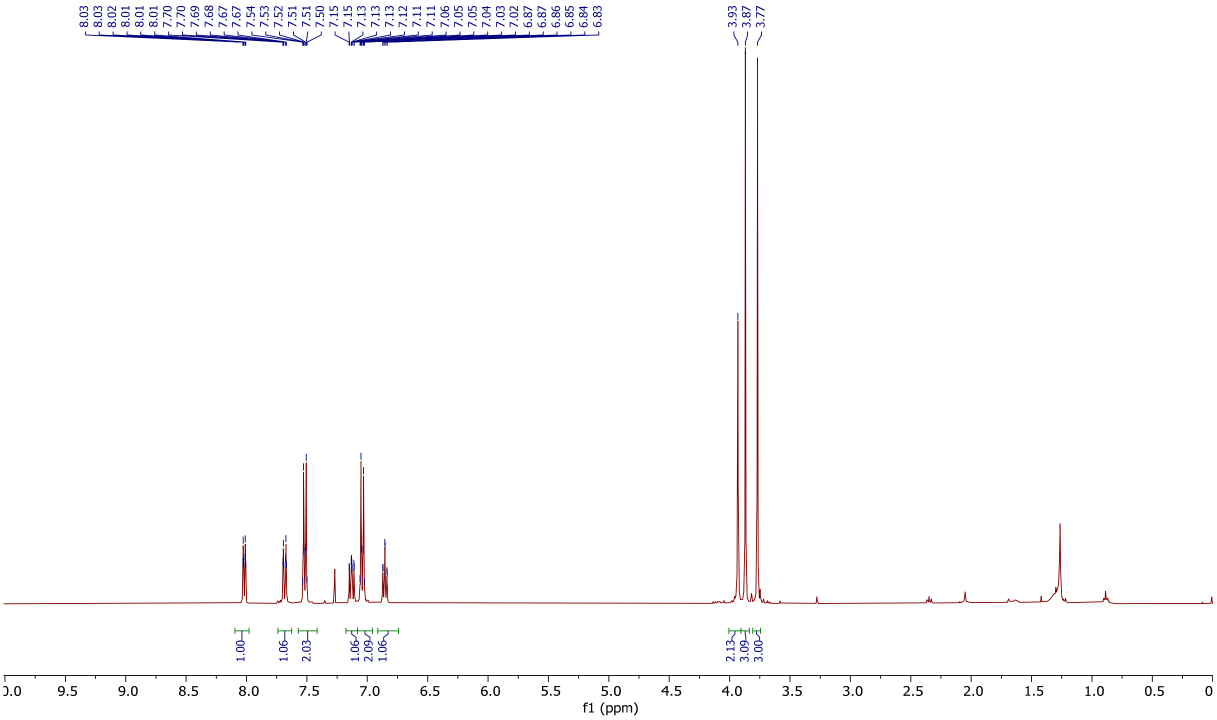

^{1}H NMR (400 MHz, $CDCl_3$) of **2r**.

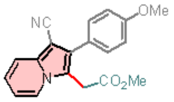

2r

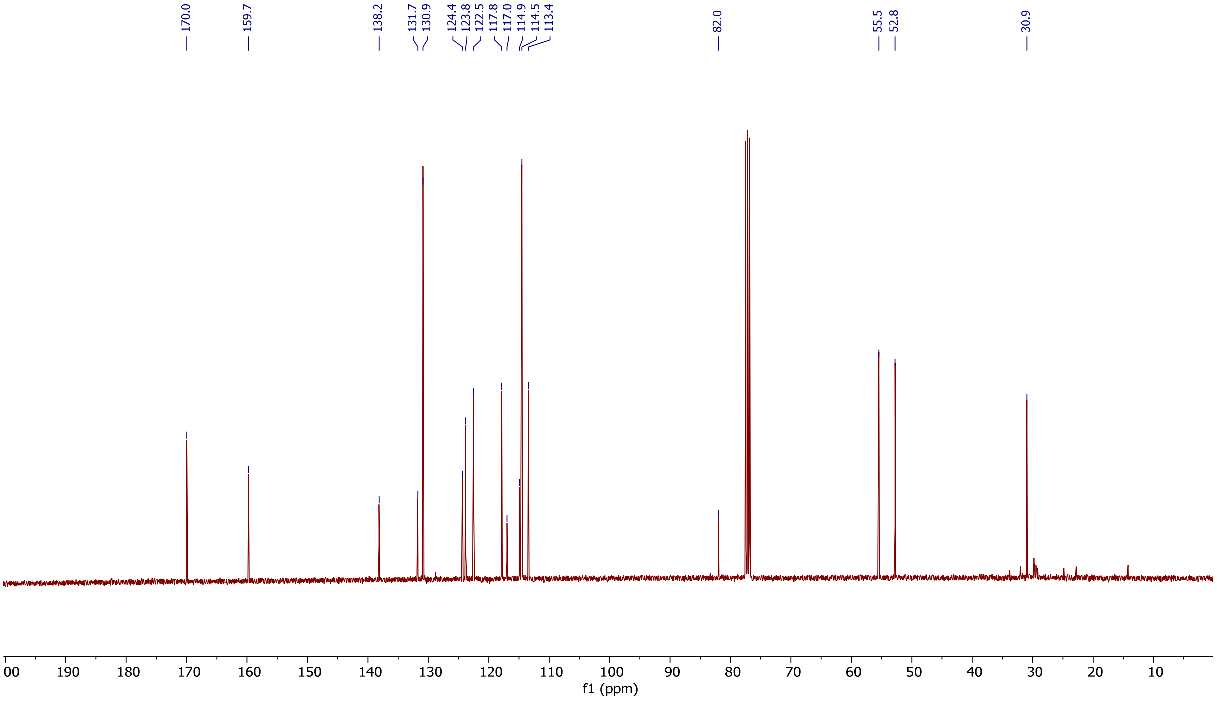

^{13}C{^{1}H} NMR (100 MHz, $CDCl_3$) of **2r**.

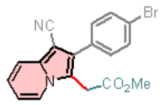

2s

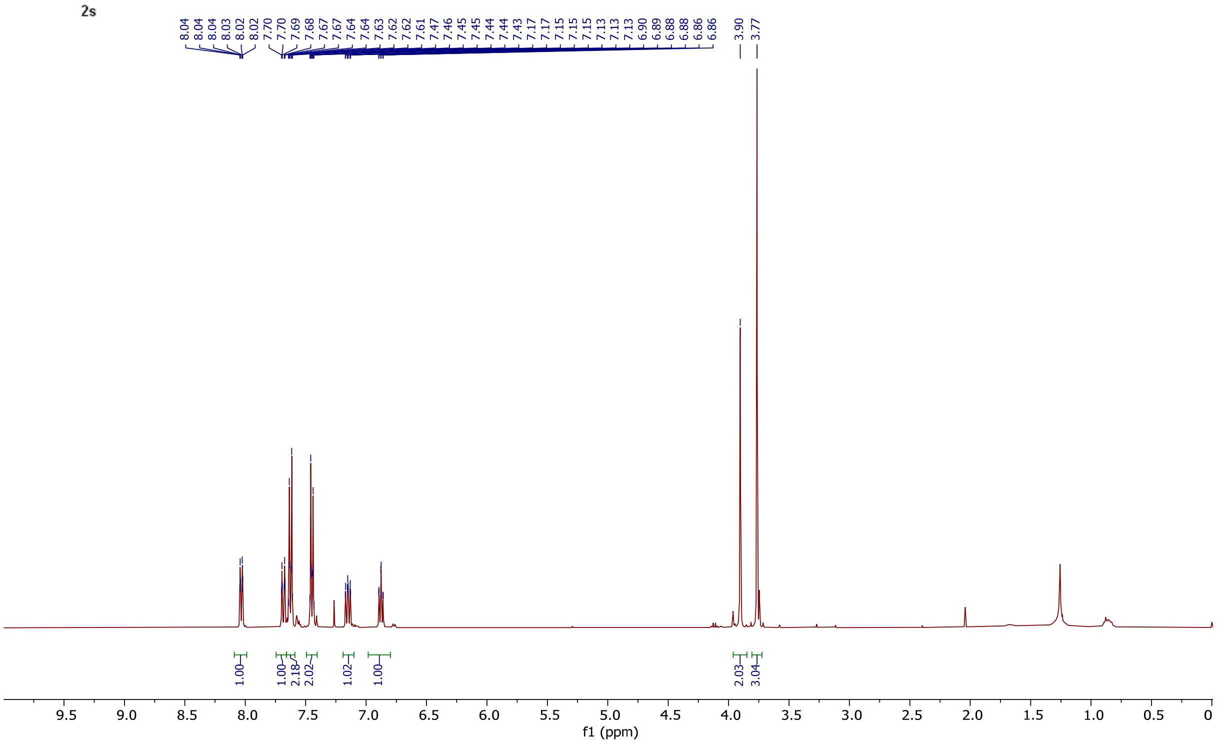

^{1}H NMR (400 MHz, $CDCl_3$) of **2s**.

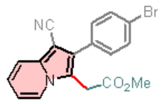

2s

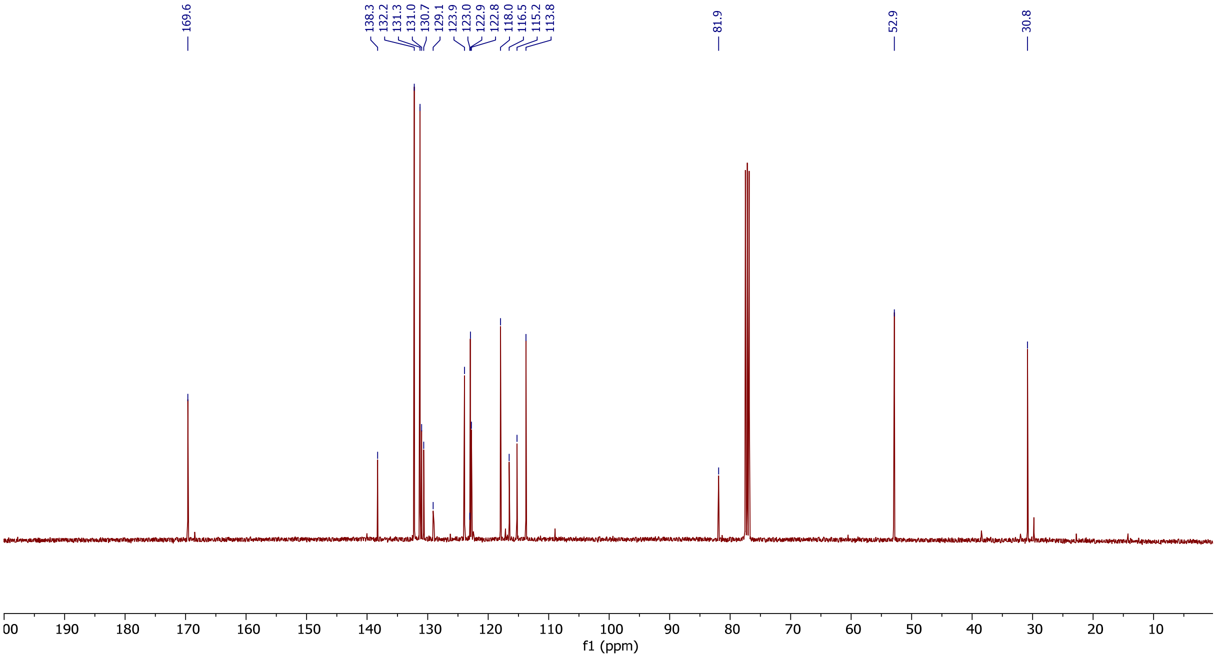

^{13}C{^{1}H} NMR (100 MHz, $CDCl_3$) of **2s**.

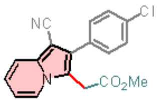

2t

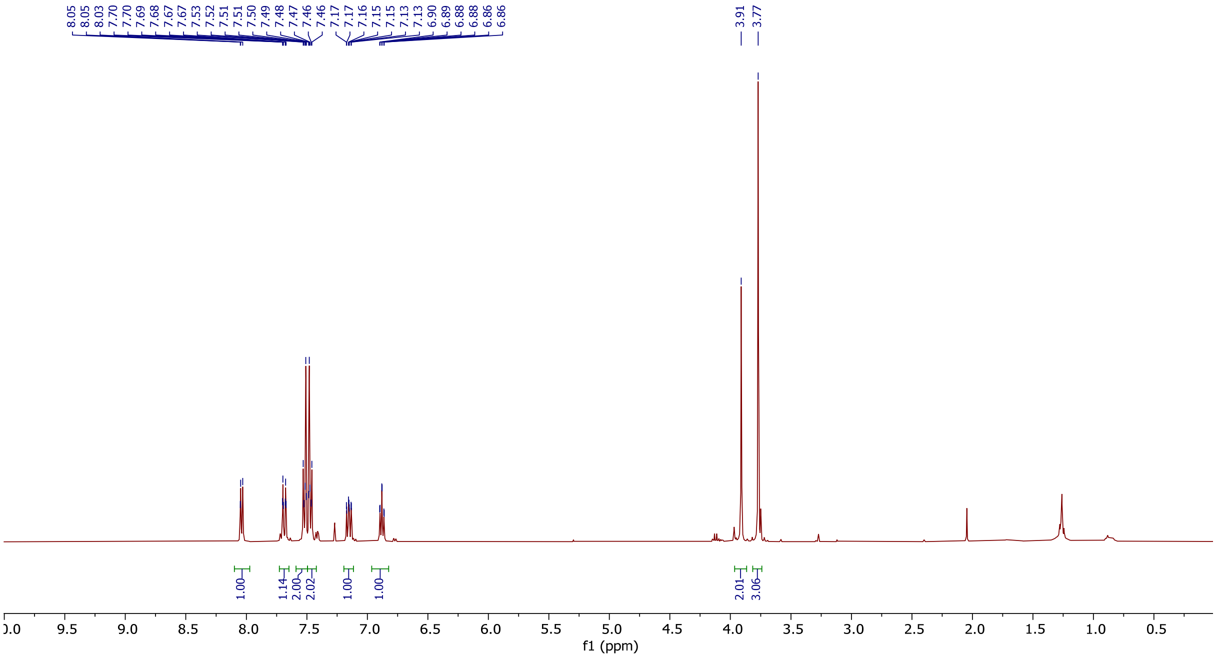

^{1}H NMR (400 MHz, $CDCl_3$) of **2t**.

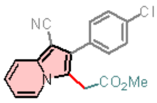

2t

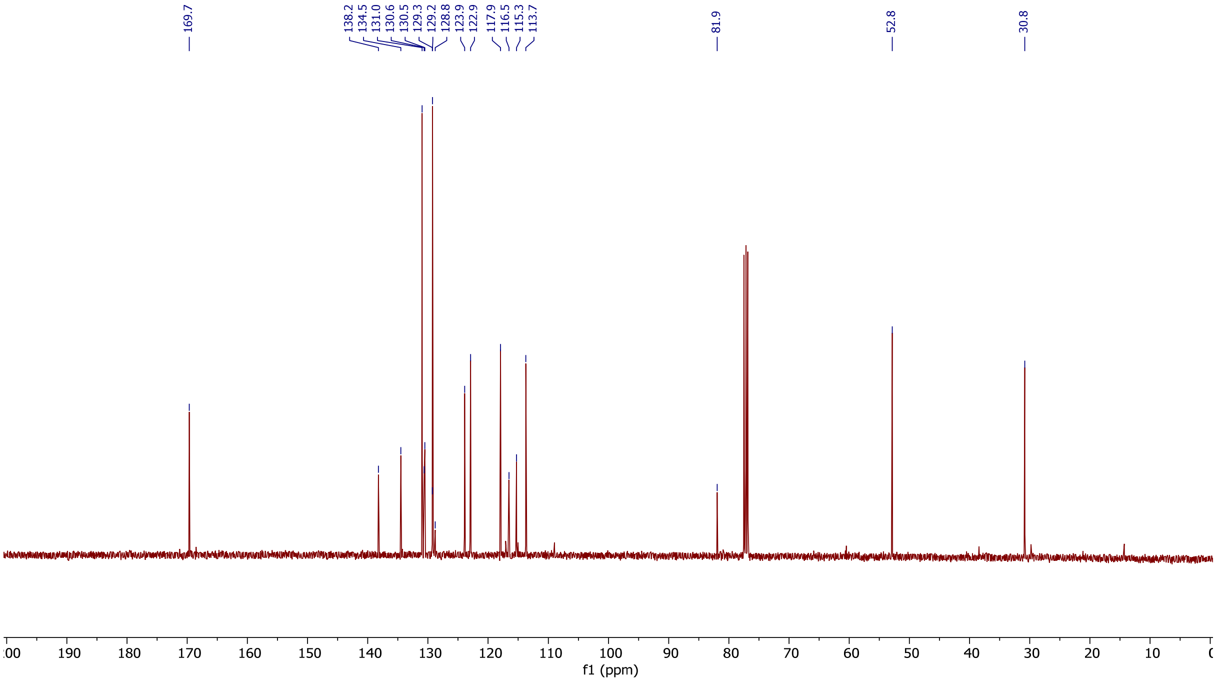

$^{13}C\{^{1}H\}$ NMR (100 MHz, $CDCl_3$) of **2t**.

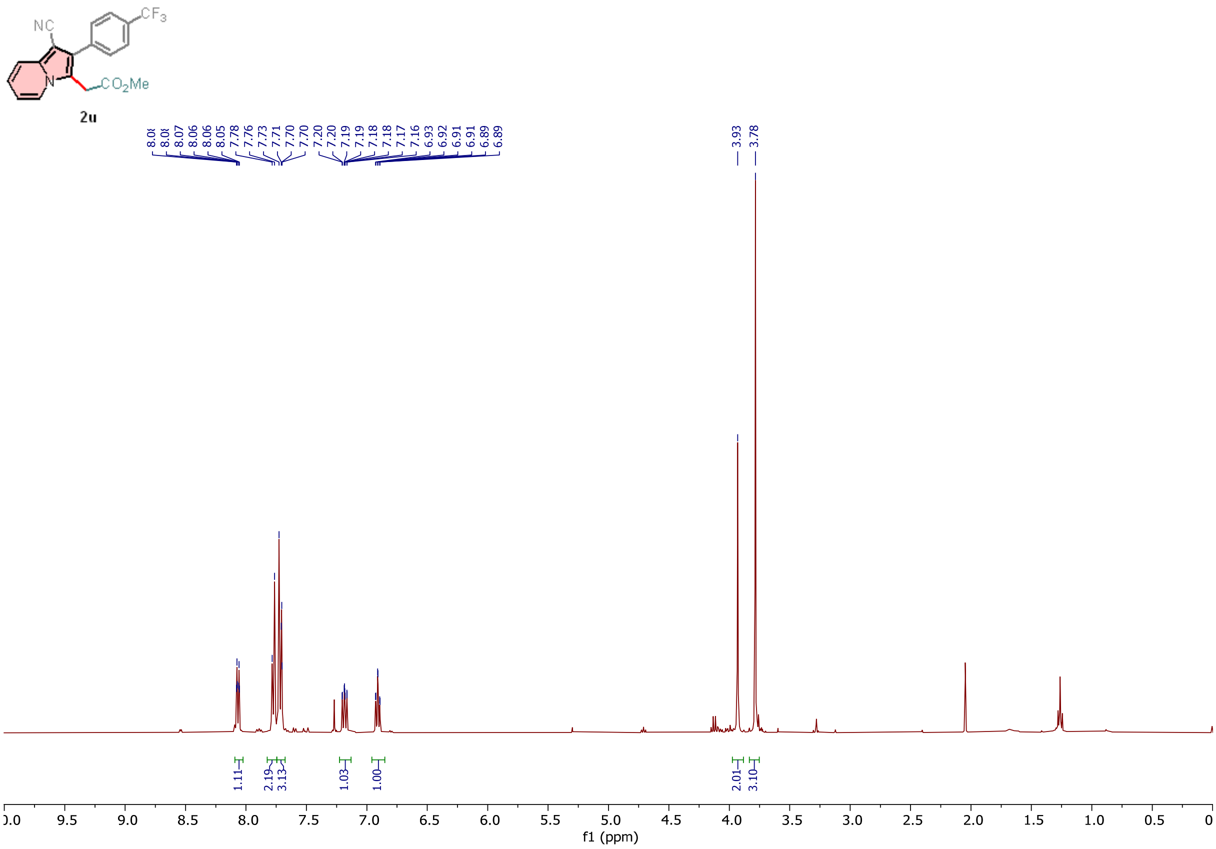

^{1}H NMR (400 MHz, CDCl$_3$) of **2u**.

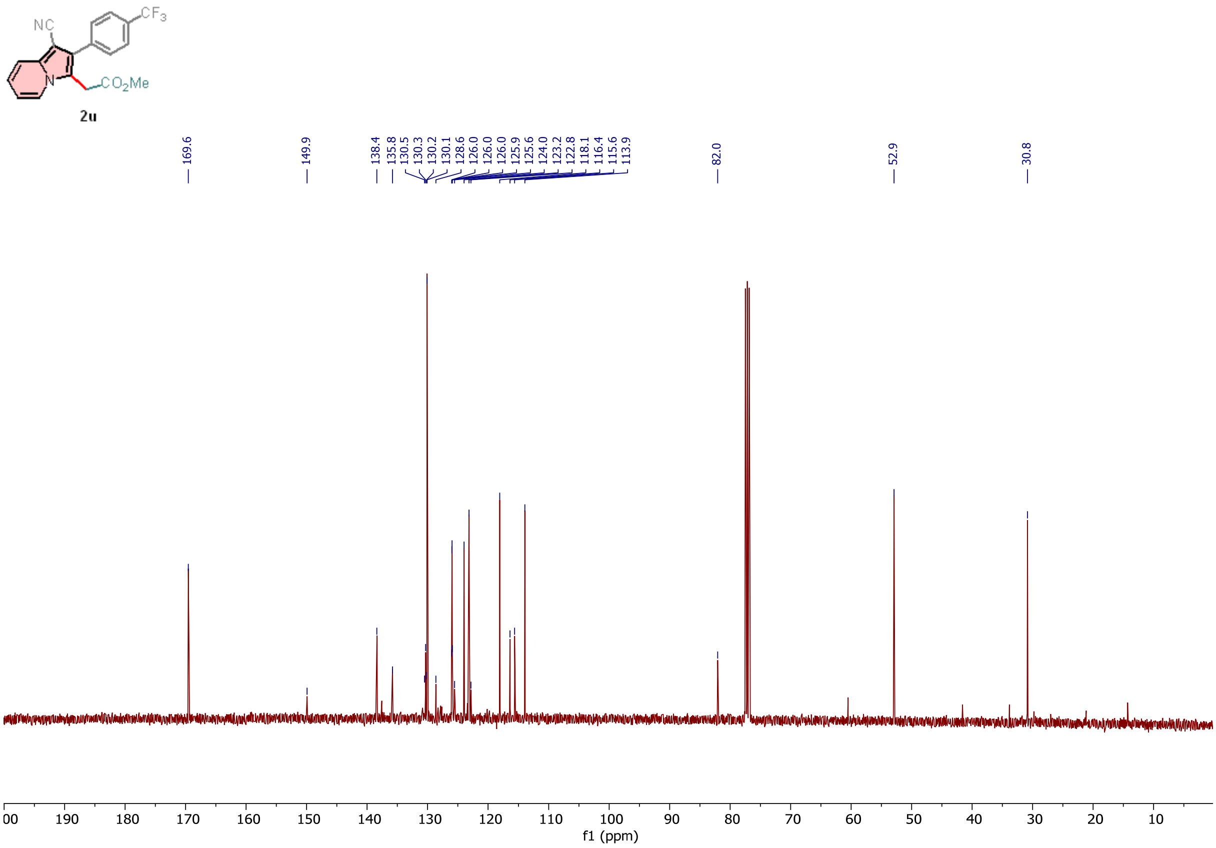

^{13}C{^{1}H} NMR (100 MHz, CDCl$_3$) of **2u**.

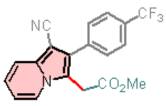

2u

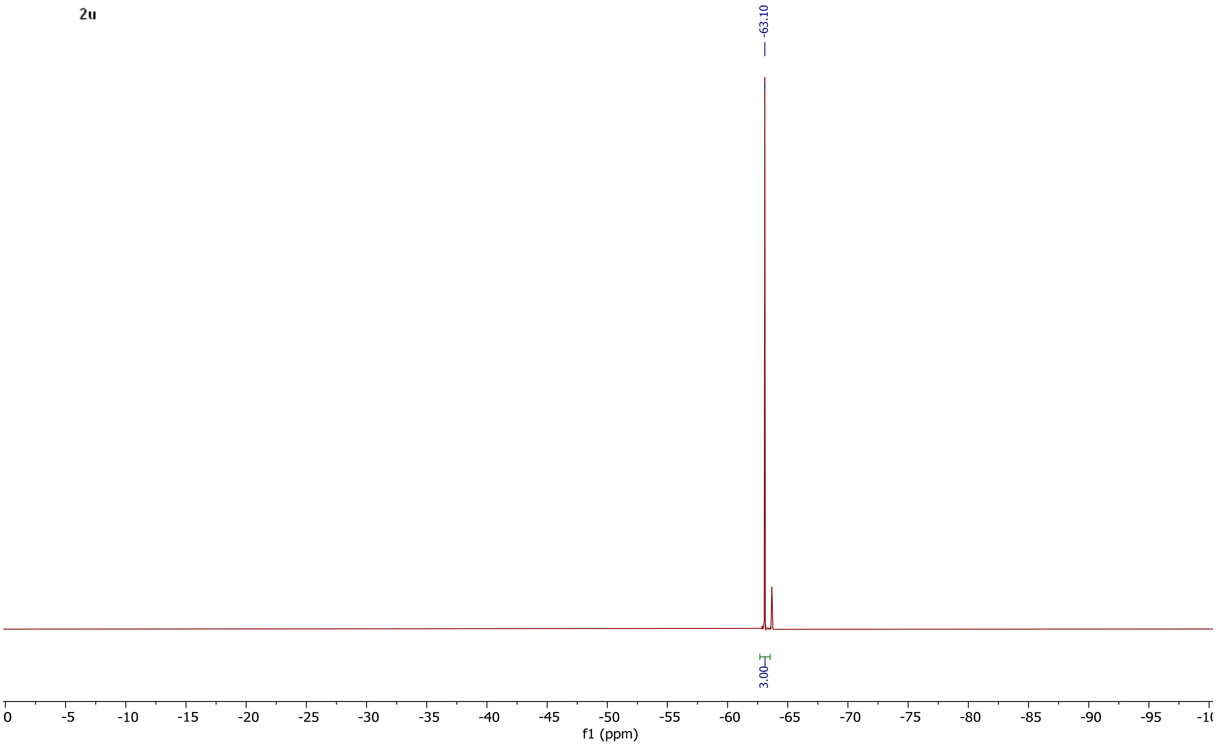

$^{19}F\{^1H\}$ NMR (377 MHz, $CDCl_3$) of **2u**.

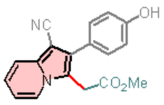

2v

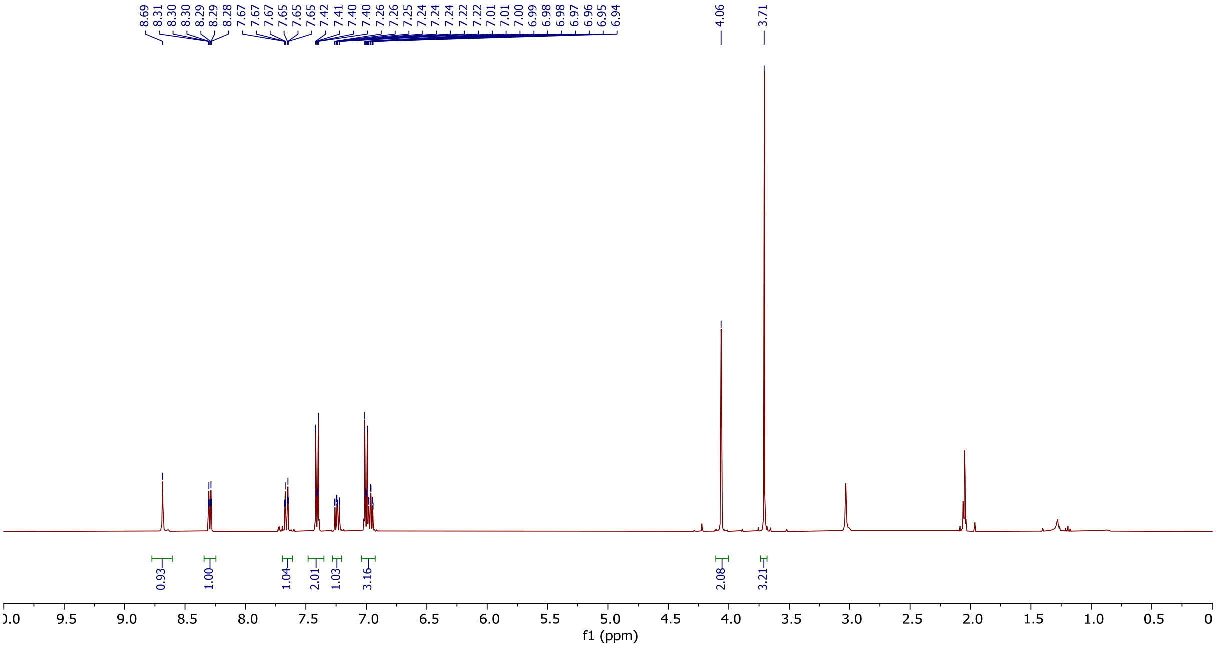

^1H NMR (400 MHz, Acetone-d^6) of **2v**.

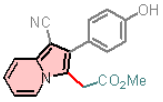

2v

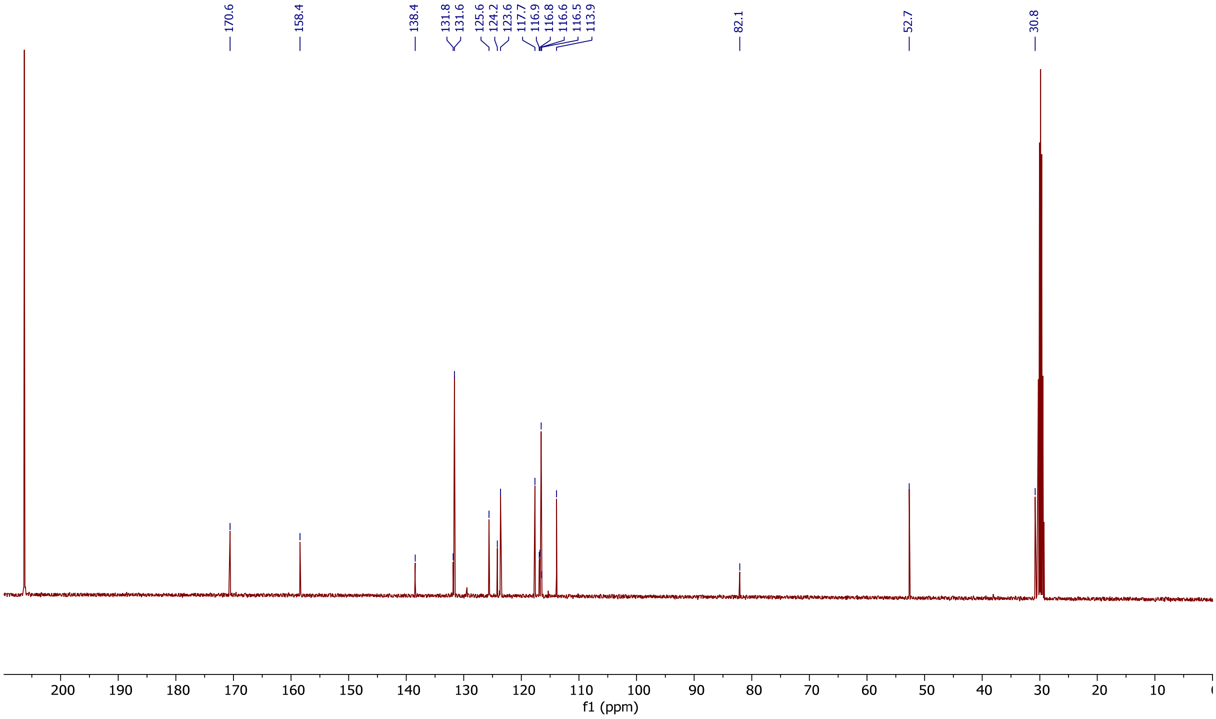

$^{13}C\{^1H\}$ NMR (100 MHz, Acetone-d^6) of **2v**.

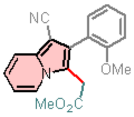

2w

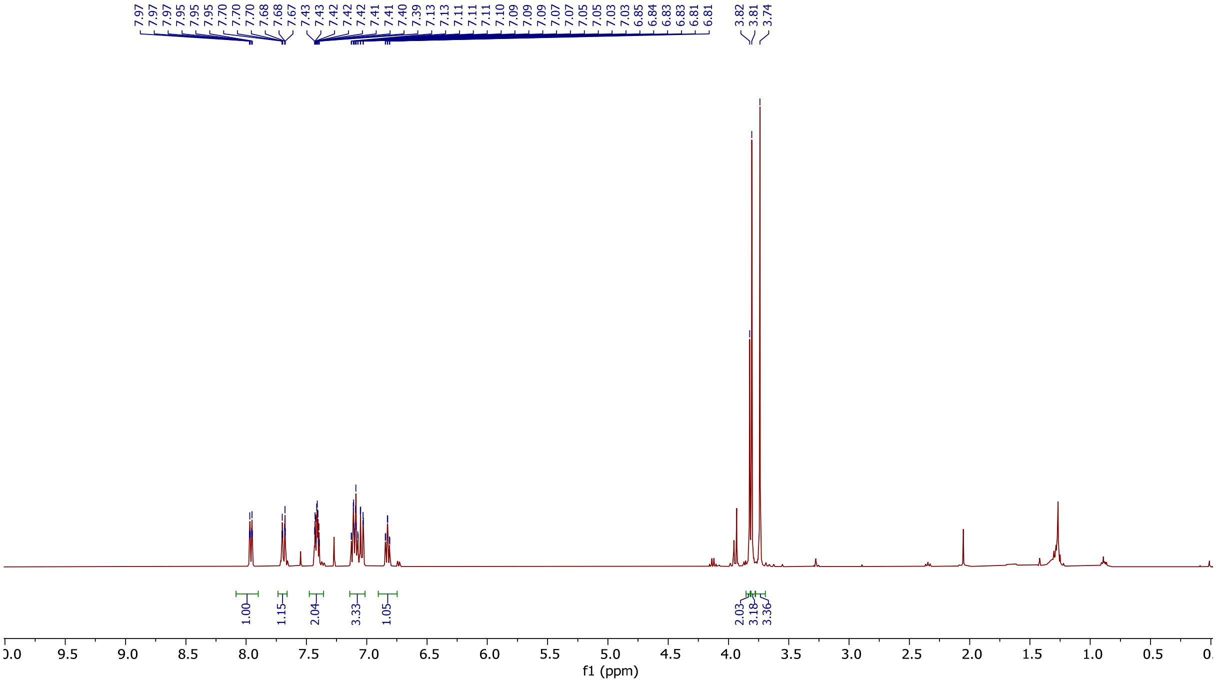

^1H NMR (400 MHz, $CDCl_3$) of **2w**.

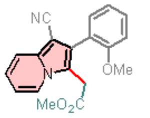

2w

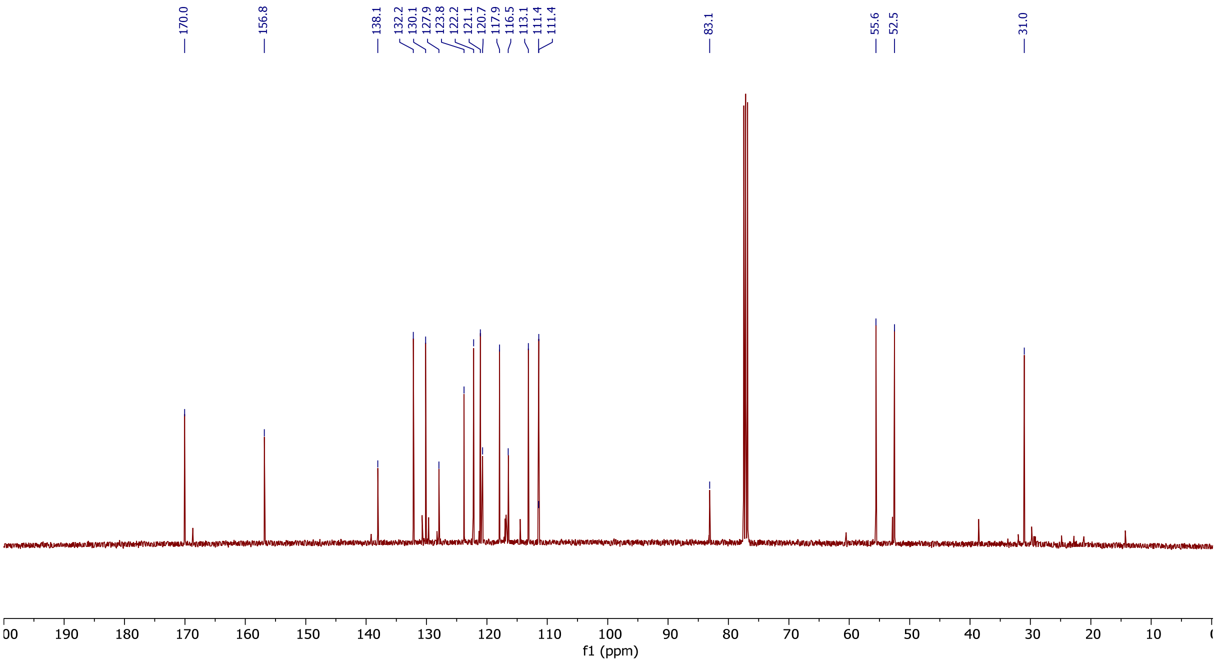

$^{13}C\{^{1}H\}$ NMR (100 MHz, $CDCl_3$) of **2w**.

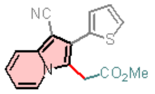

2X

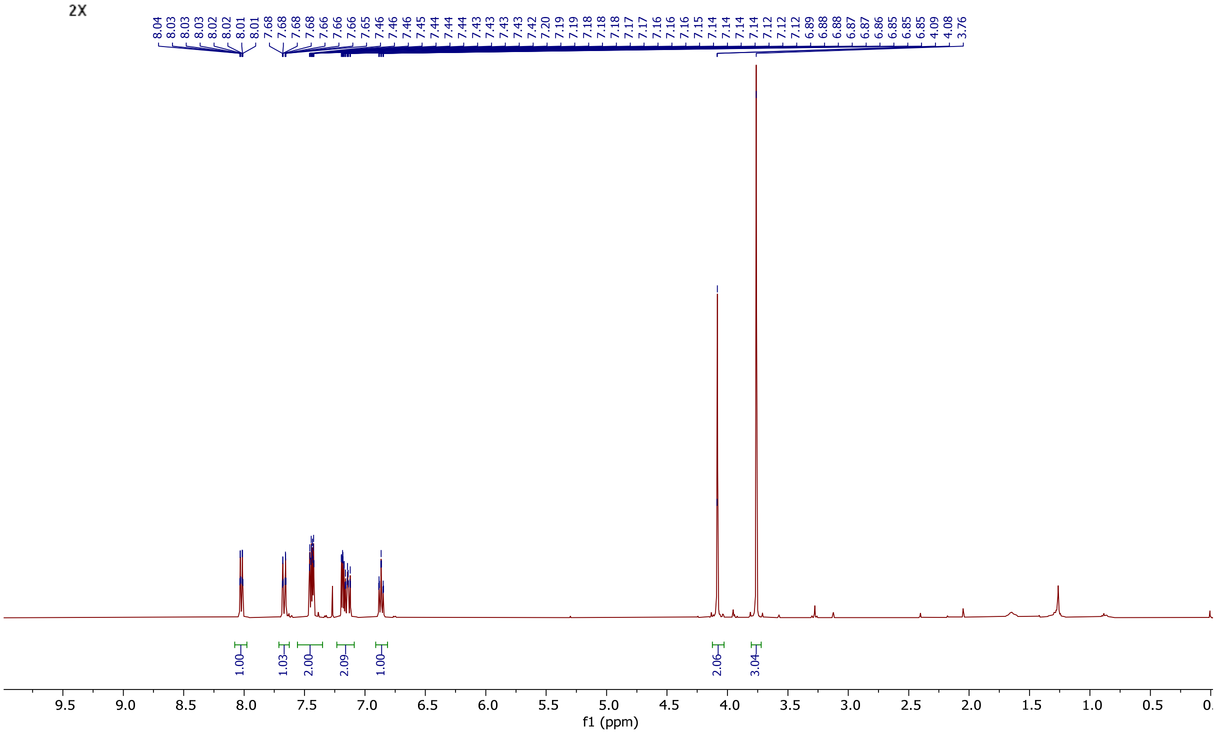

^{1}H NMR (400 MHz, $CDCl_3$) of **2x**.

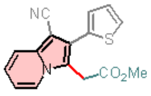

2X

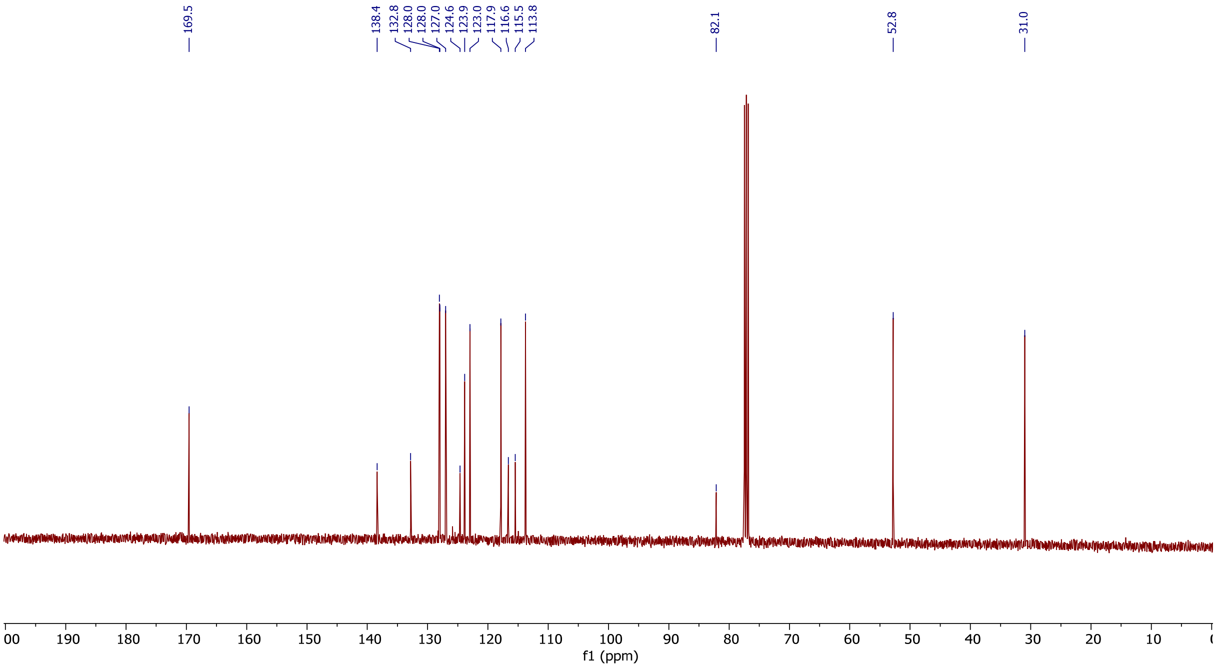

$^{13}C\{^{1}H\}$ NMR (100 MHz, $CDCl_3$) of **2x**.

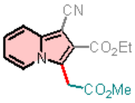

2y

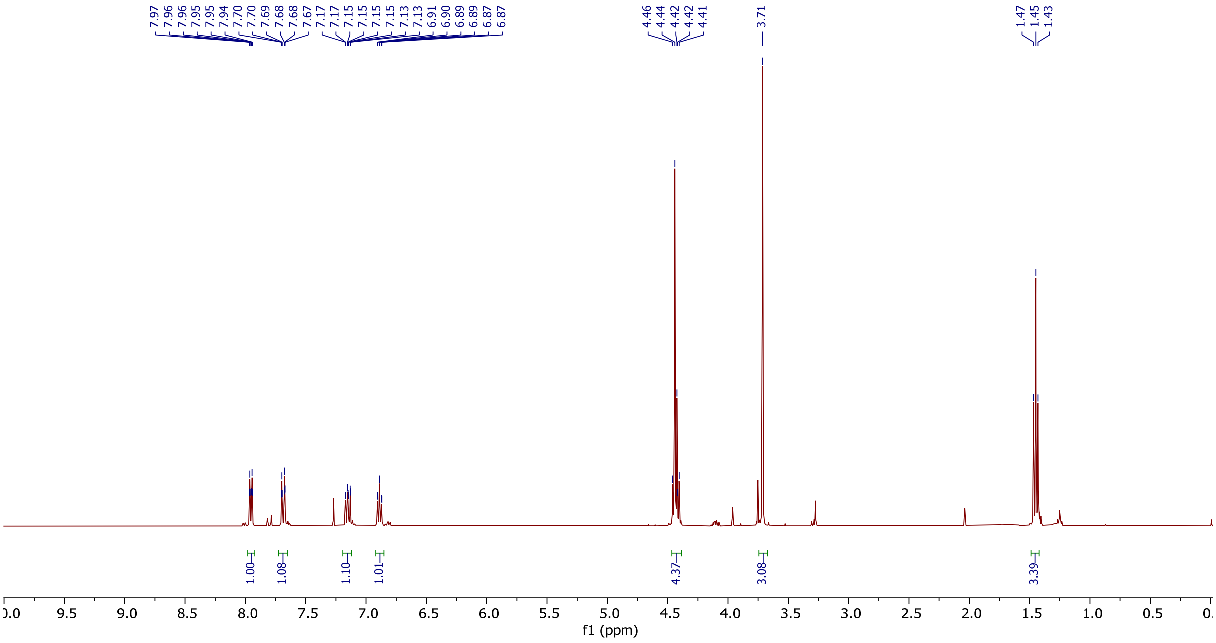

^{1}H NMR (400 MHz, $CDCl_3$) of **2y**.

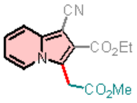

2y

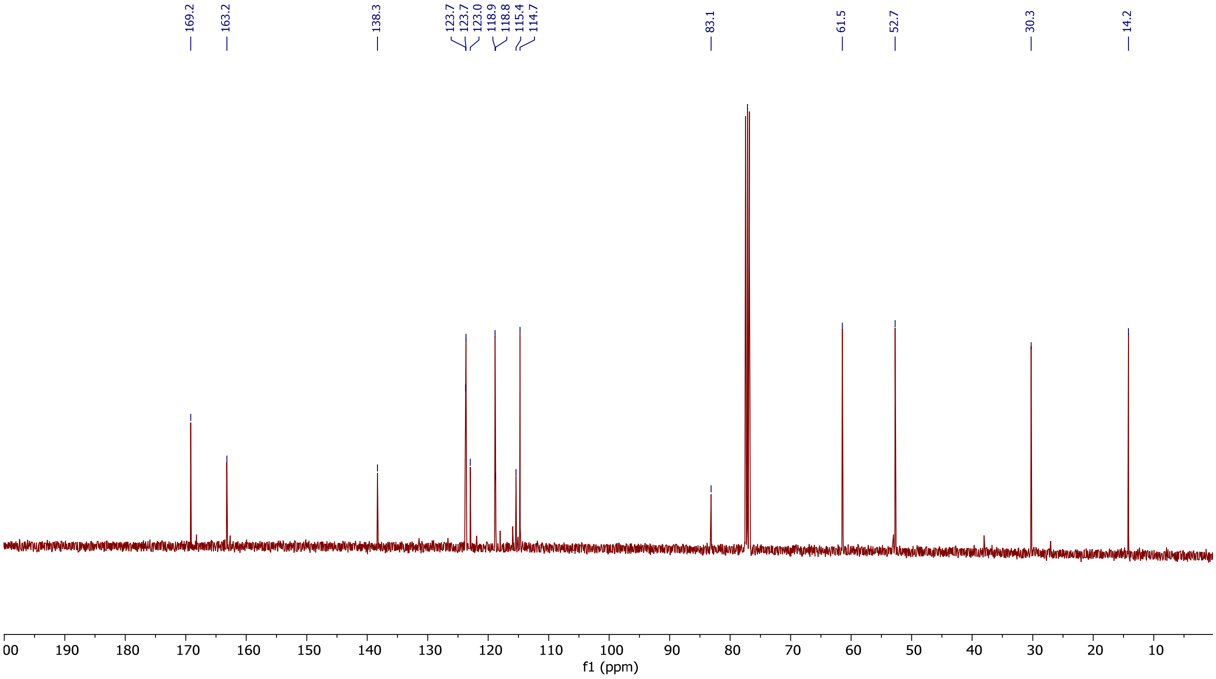

$^{13}C\{^{1}H\}$ NMR (100 MHz, $CDCl_3$) of **2y**.

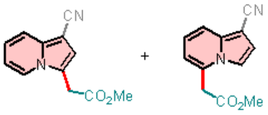

2z + 2z' (1:2)

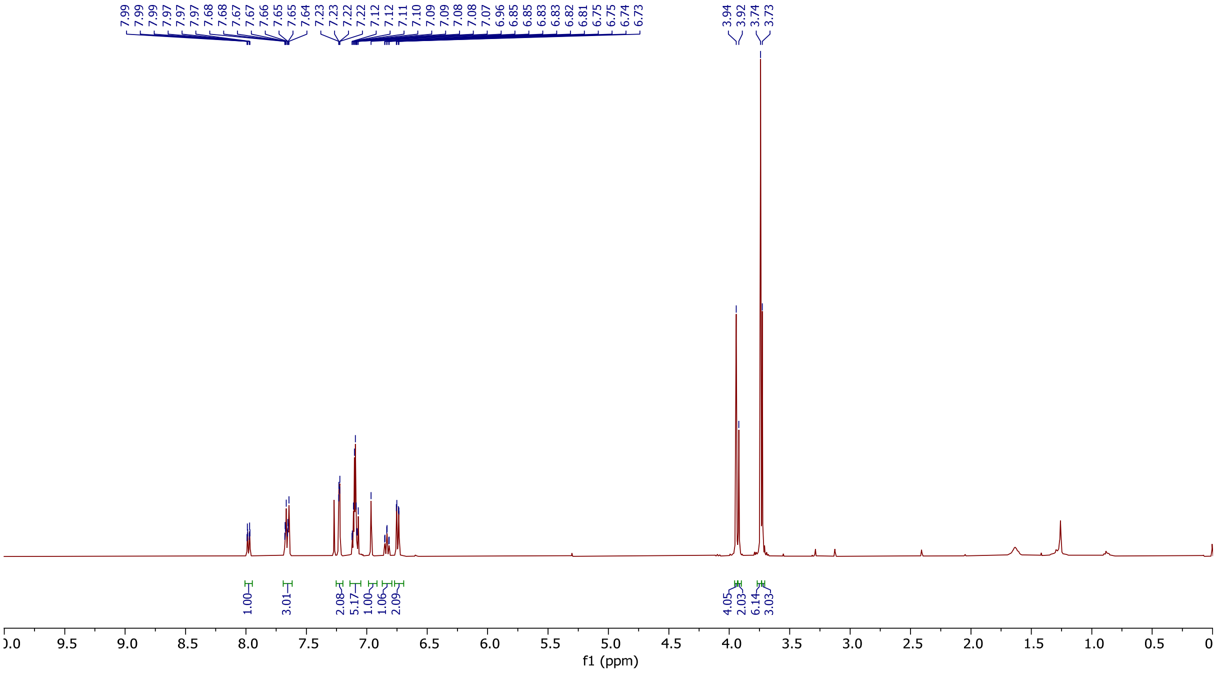

^{1}H NMR (400 MHz, $CDCl_3$) of **2z + 2z'**.

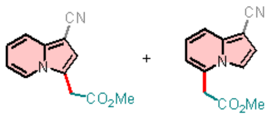

2z + **2z'** (1:2)

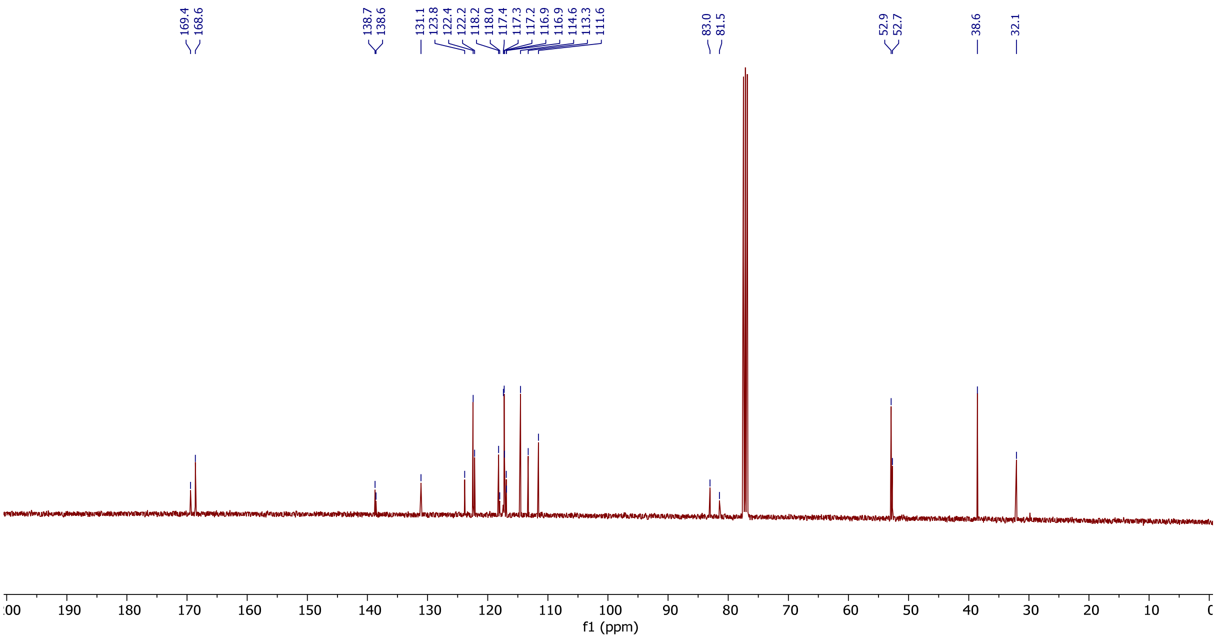

$^{13}C\{^{1}H\}$ NMR (100 MHz, $CDCl_3$) of **2z** + **2z'**.

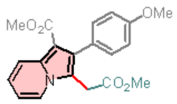

2aa

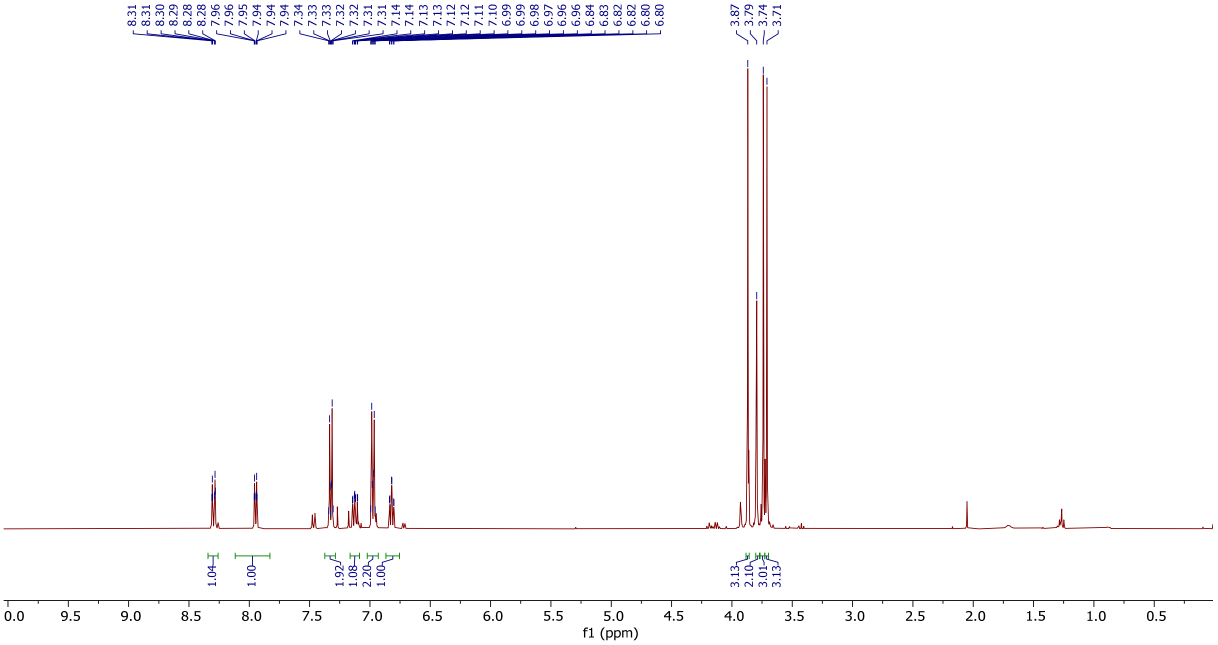

^{1}H NMR (400 MHz, $CDCl_3$) of **2aa**.

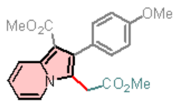

2aa

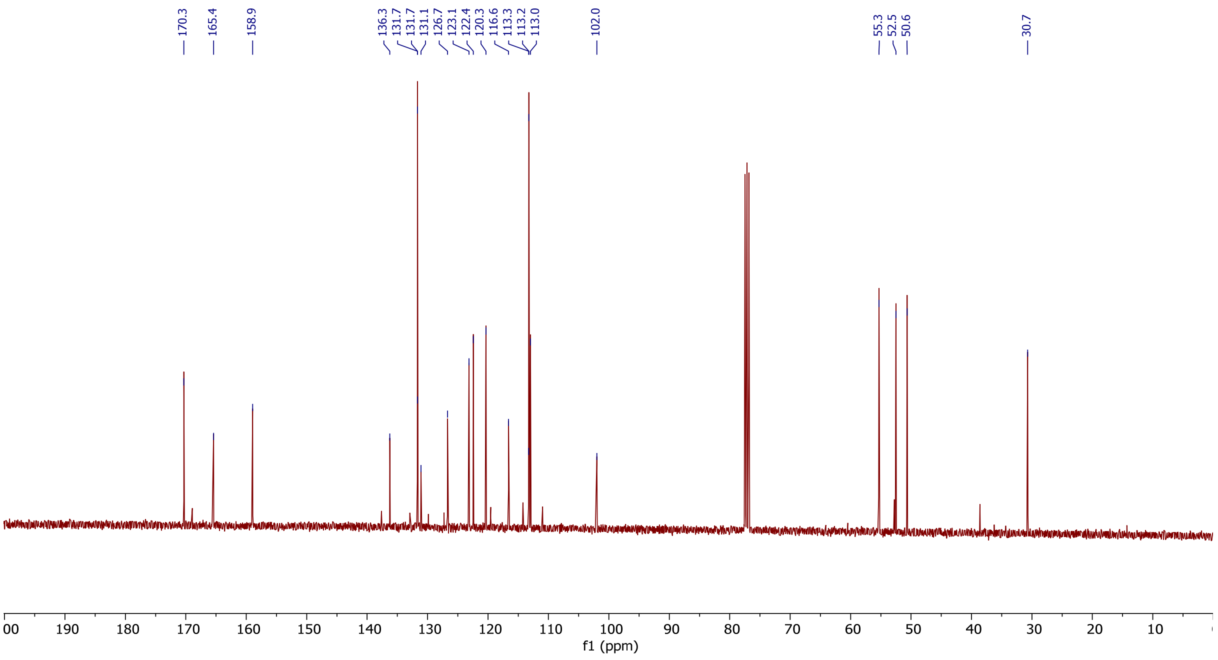

$^{13}C\{^{1}H\}$ NMR (100 MHz, $CDCl_3$) of **2aa**.

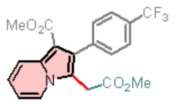

2ab

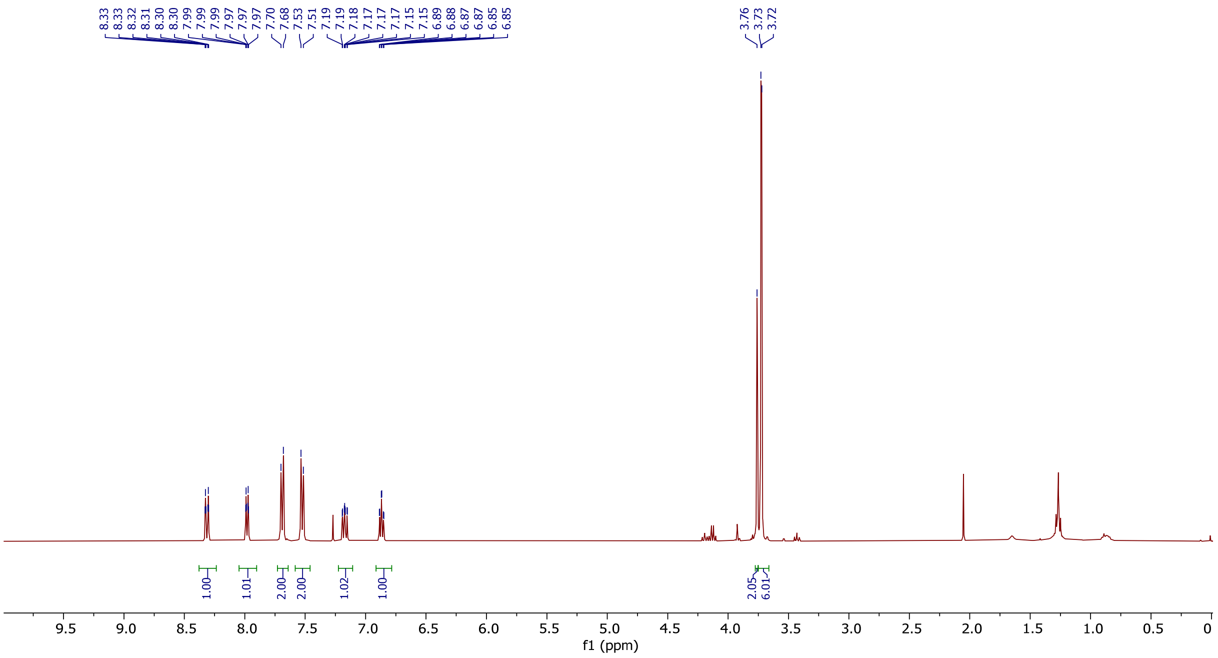

^{1}H NMR (400 MHz, $CDCl_3$) of **2ab**.

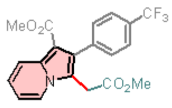

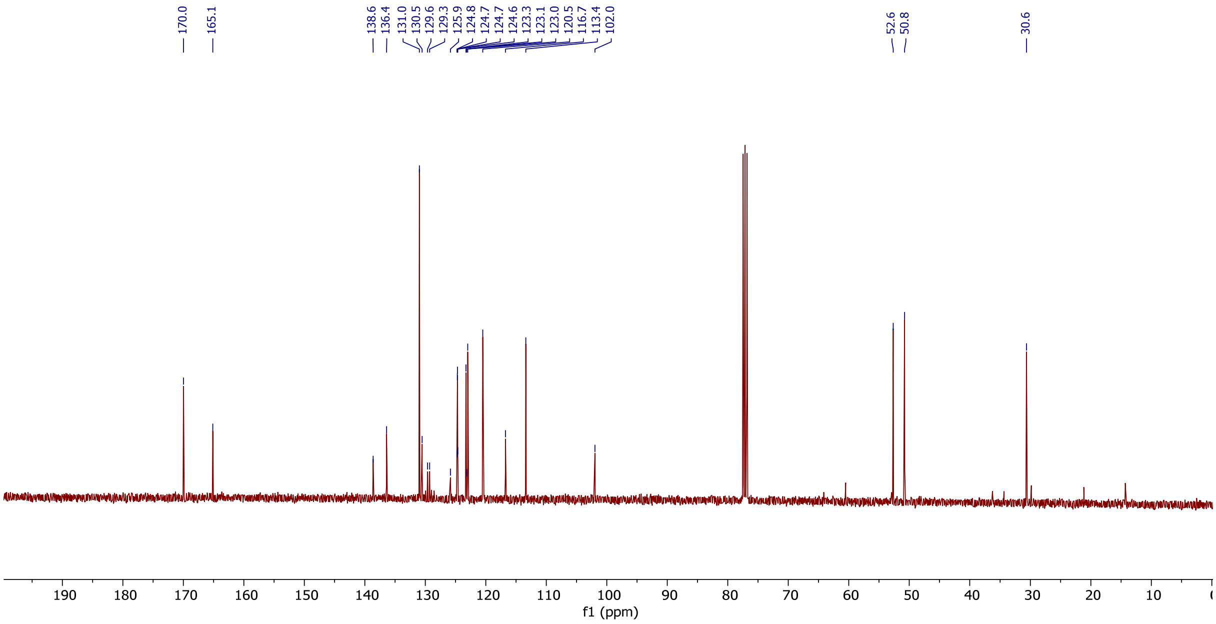

$^{13}C\{^{1}H\}$ NMR (100 MHz, $CDCl_3$) of **2ab**.

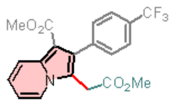

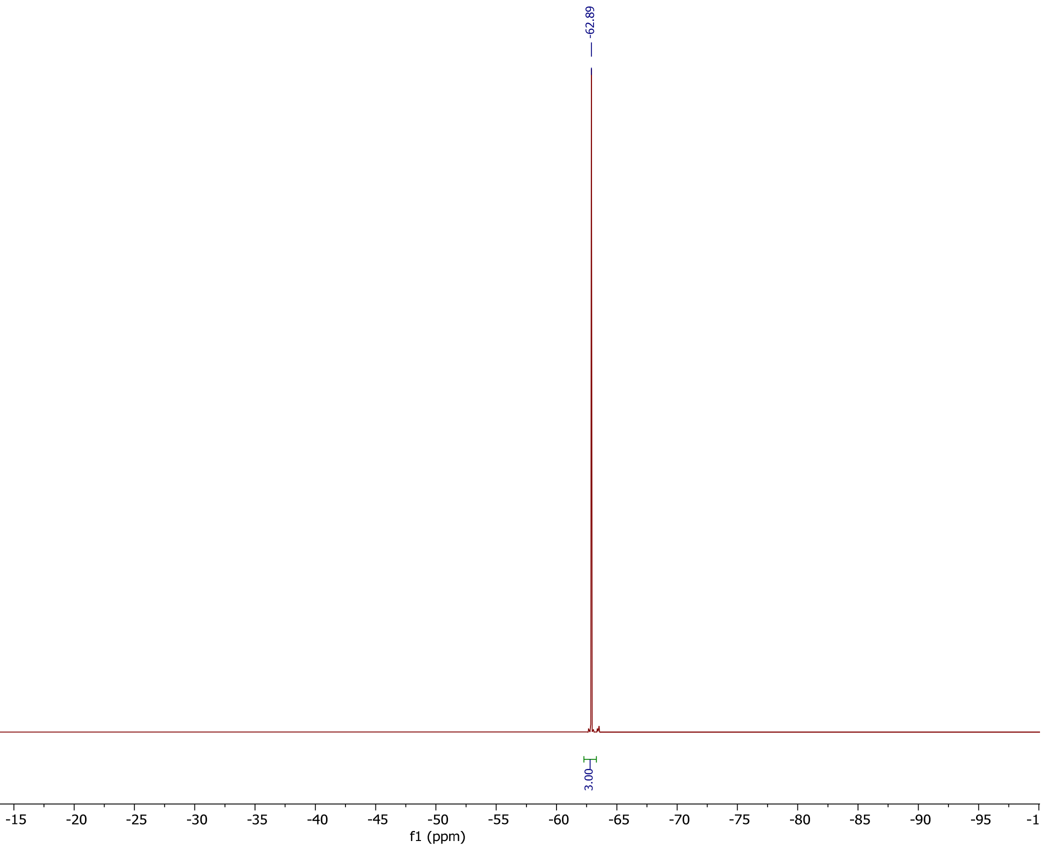

$^{19}F\{^{1}H\}$ NMR (377 MHz, $CDCl_3$) of **2ab**.

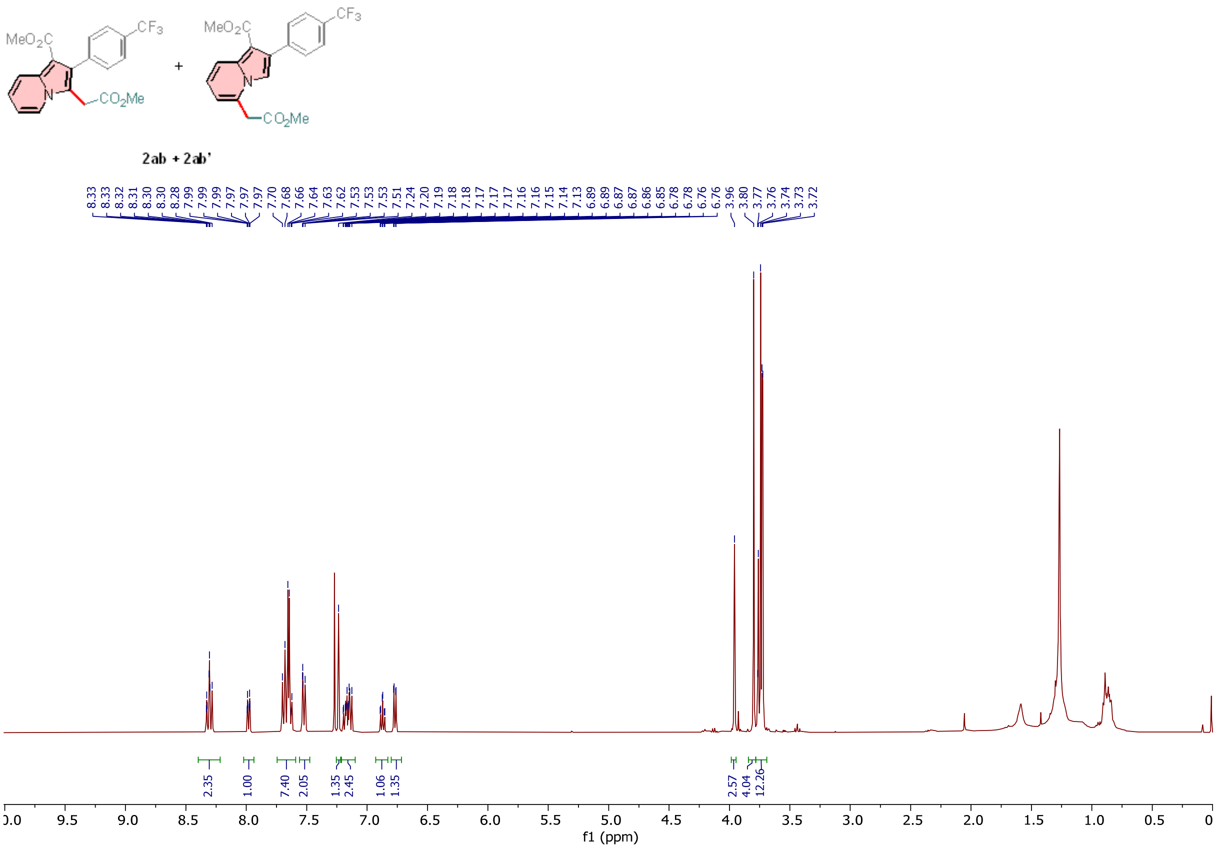

^{1}H NMR (400 MHz, $CDCl_3$) of **2ab + 2ab'**.

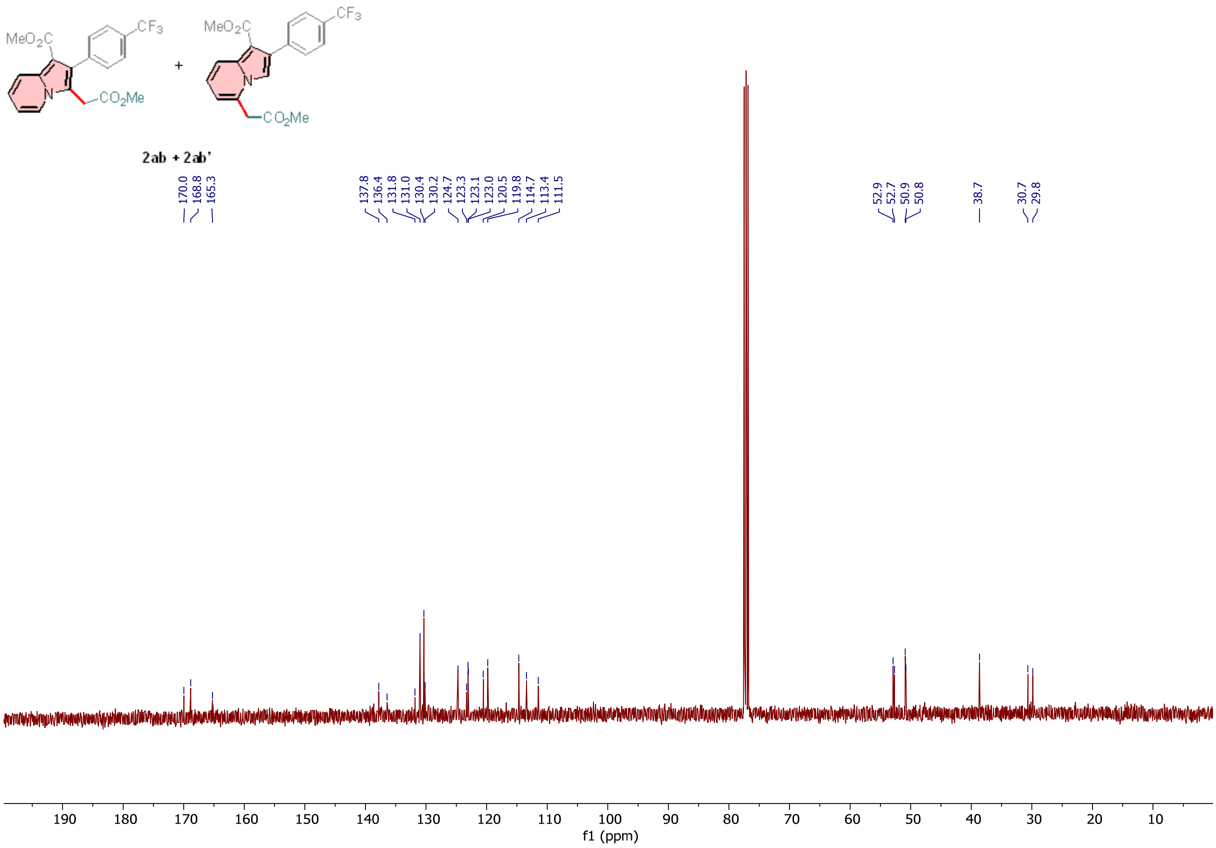

$^{13}C\{^{1}H\}$ NMR (100 MHz, $CDCl_3$) of **2ab + 2ab'**.

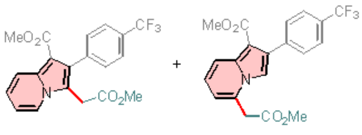

2ab + 2ab'

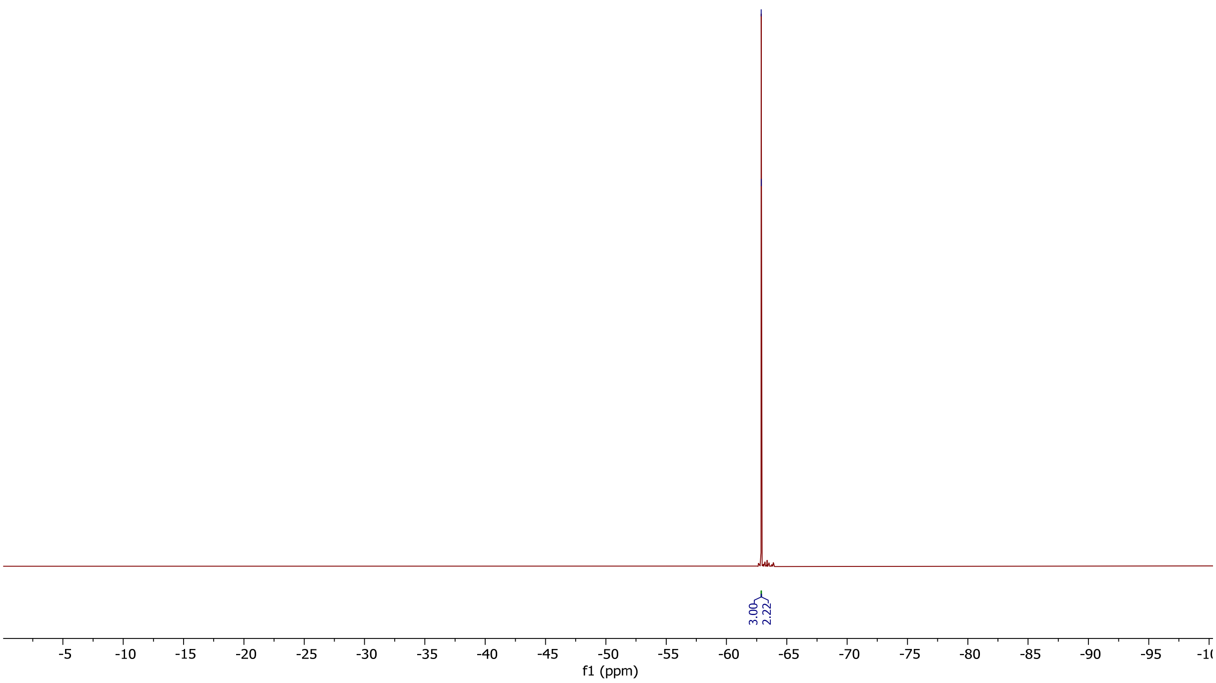

$^{19}F\{^{1}H\}$ NMR (377 MHz, $CDCl_3$) of **2ab + 2ab'**.

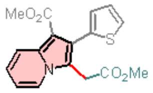

2ac

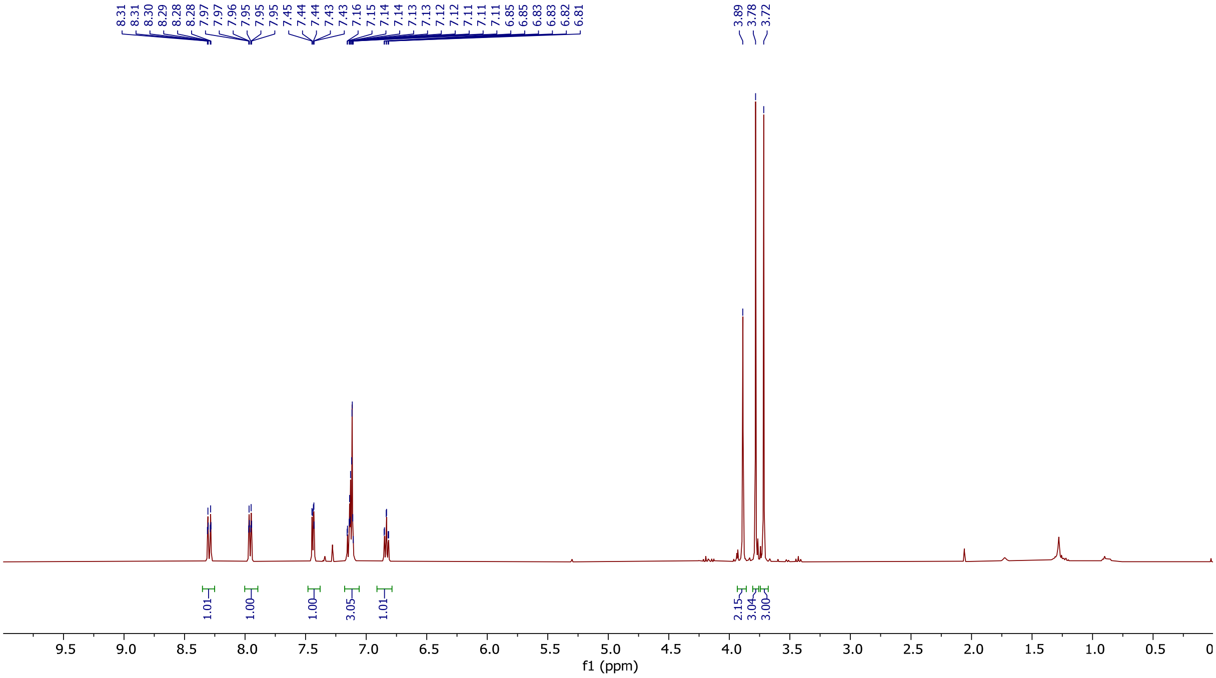

^{1}H NMR (400 MHz, $CDCl_3$) of **2ac**.

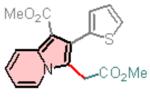

2ac

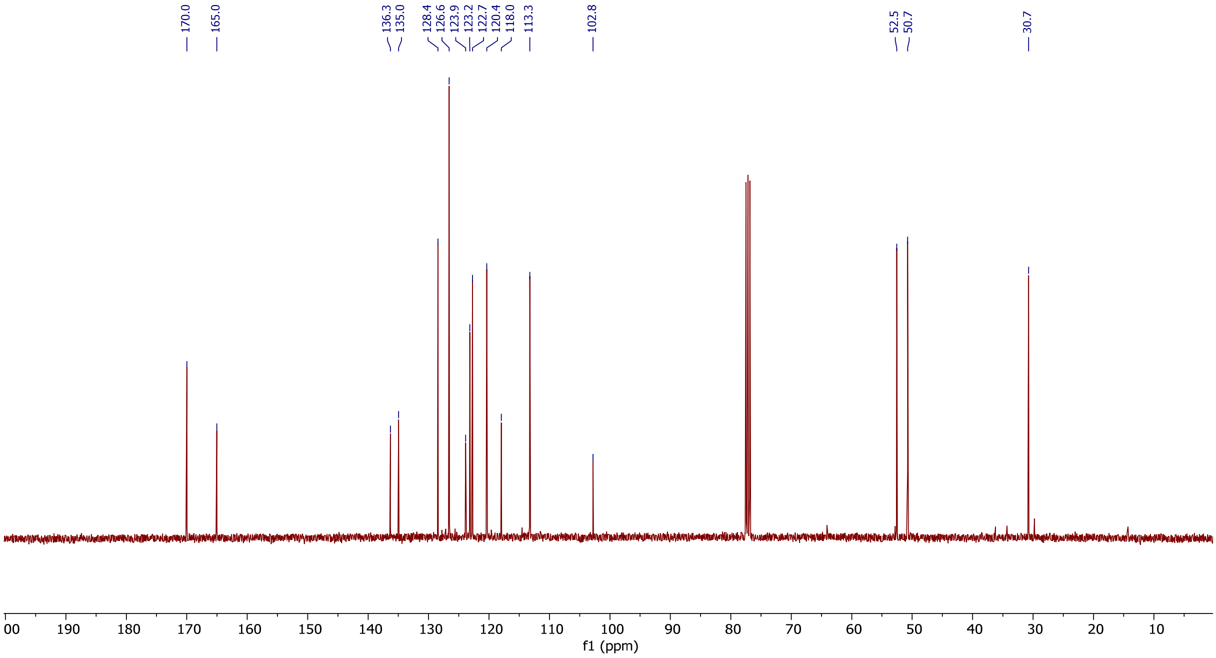

$^{13}C\{^{1}H\}$ NMR (100 MHz, $CDCl_3$) of **2ac**.

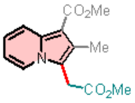

2ad

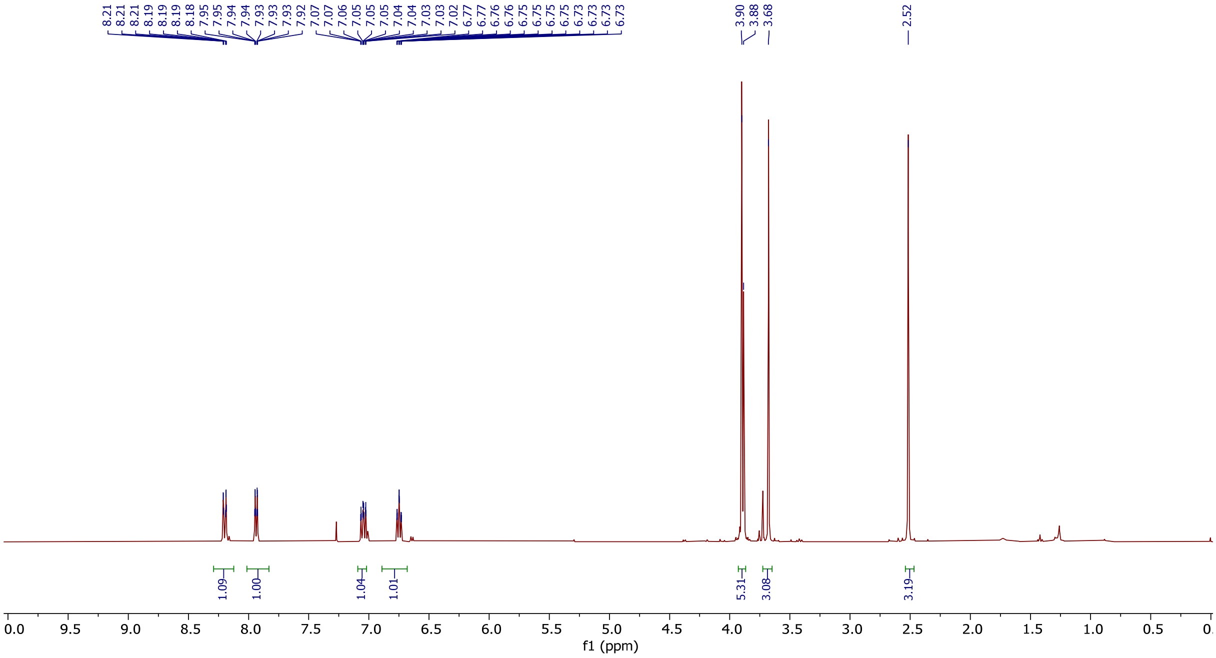

^{1}H NMR (400 MHz, $CDCl_3$) of **2ad**.

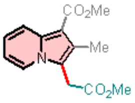

2ad

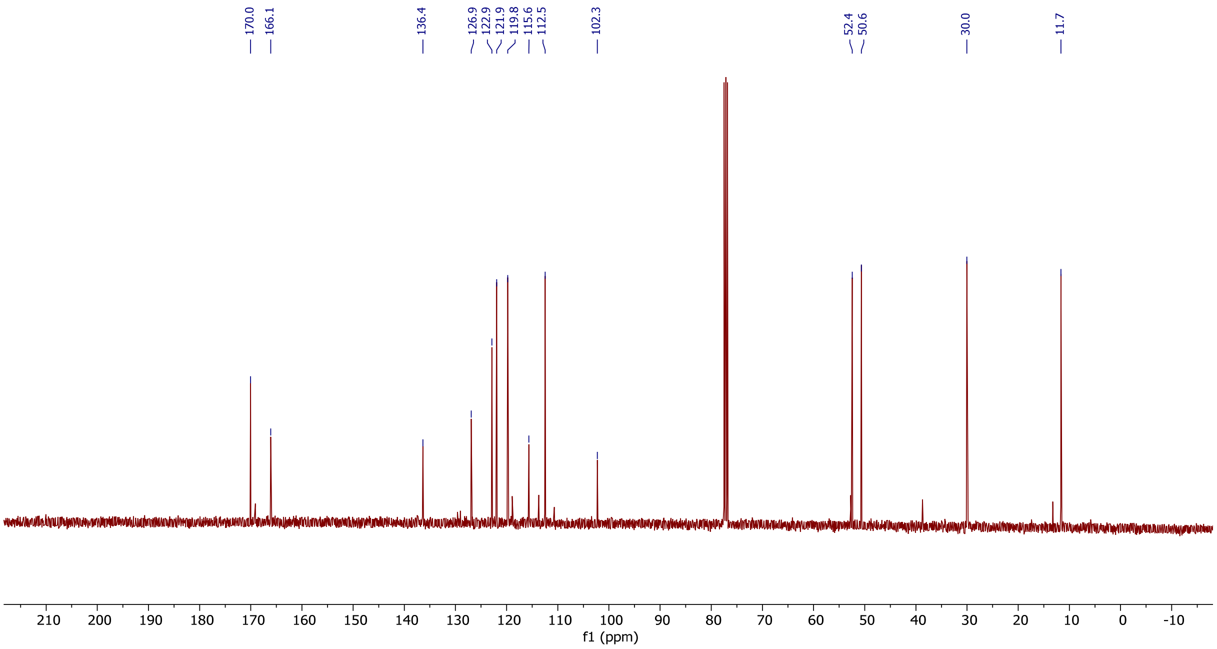

$^{13}C\{^{1}H\}$ NMR (100 MHz, $CDCl_3$) of **2ad.**

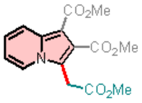

2ae

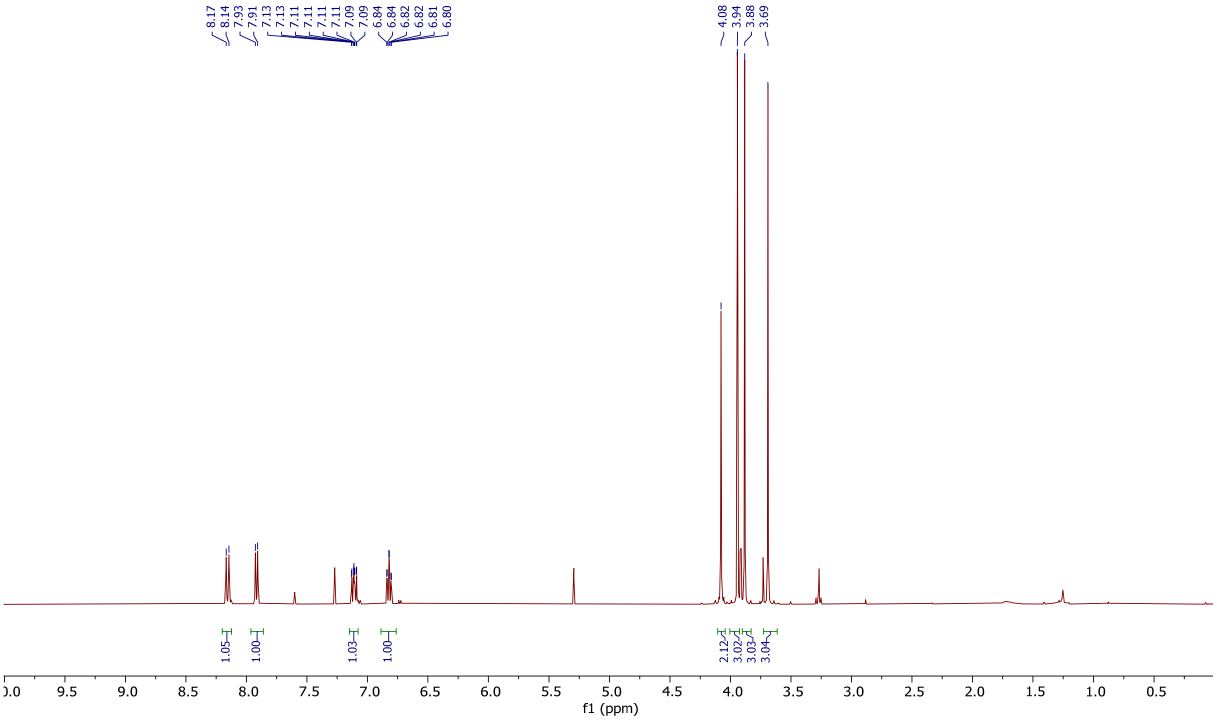

^{1}H NMR (400 MHz, $CDCl_3$) of **2ae**.

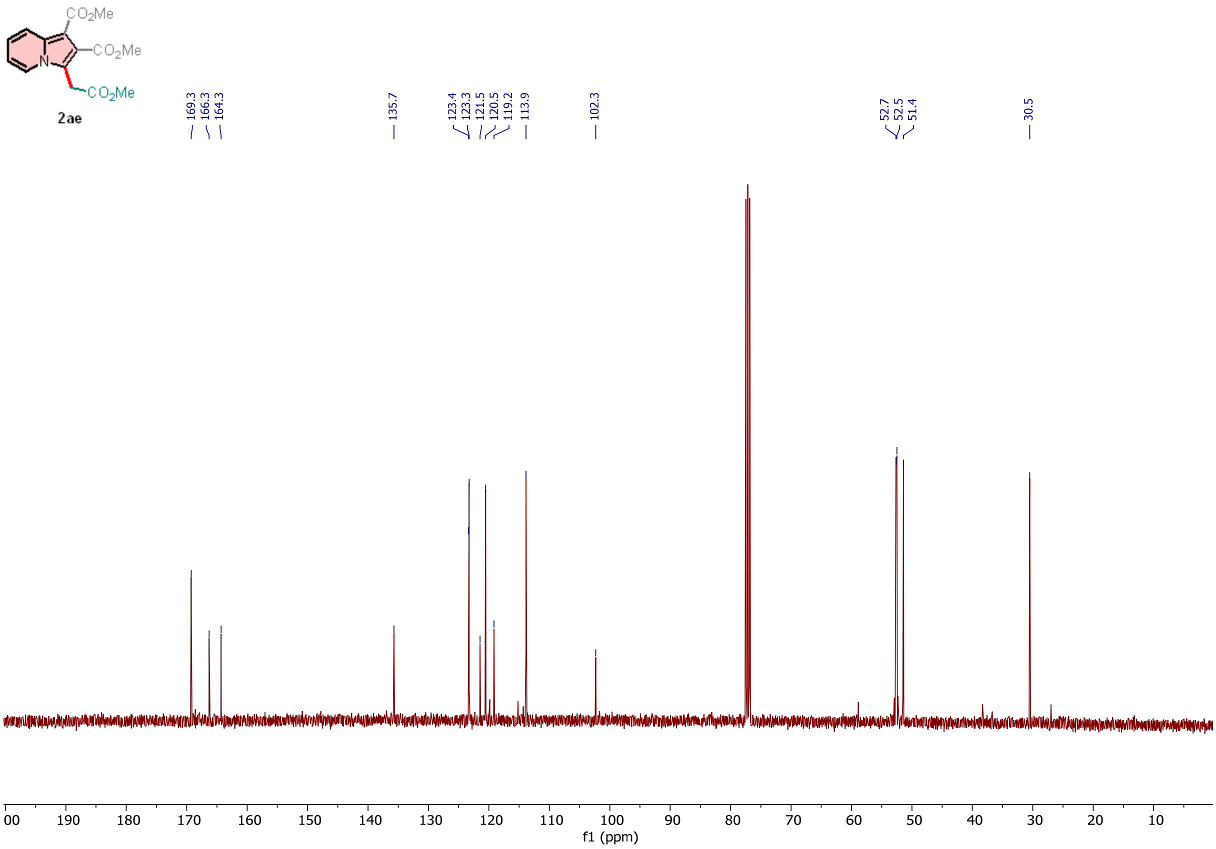

$^{13}C\{^1H\}$ NMR (100 MHz, $CDCl_3$) of **2ae**.

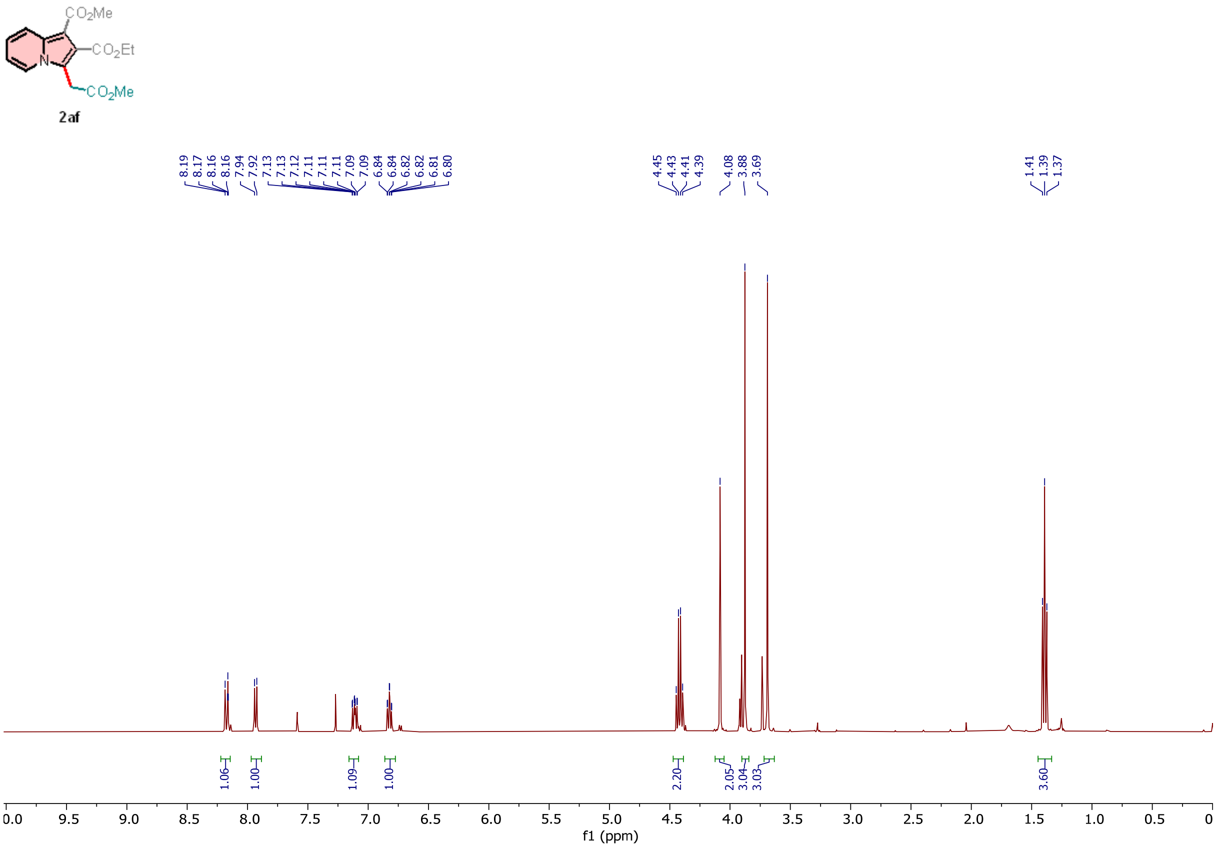

^1H NMR (400 MHz, $CDCl_3$) of **2af**.

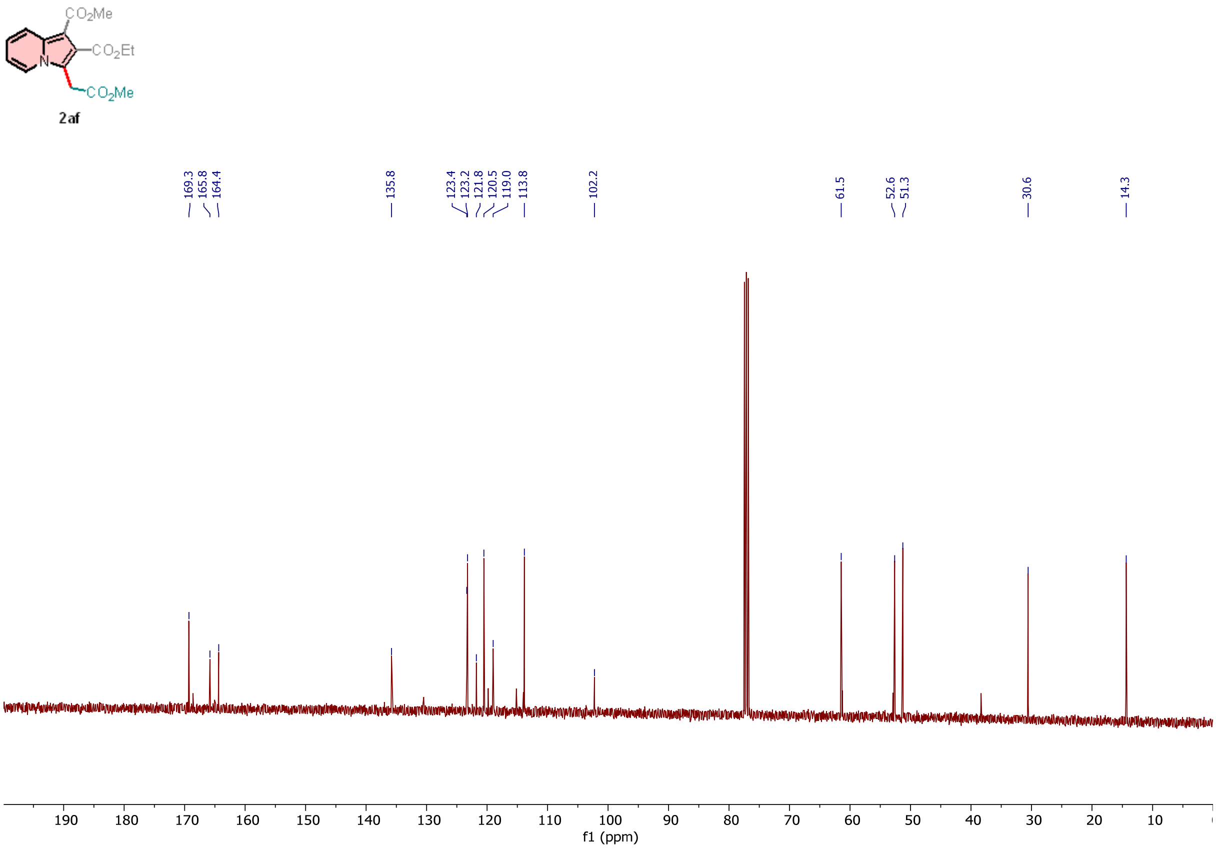

$^{13}C\{^1H\}$ NMR (100 MHz, $CDCl_3$) of **2af**.

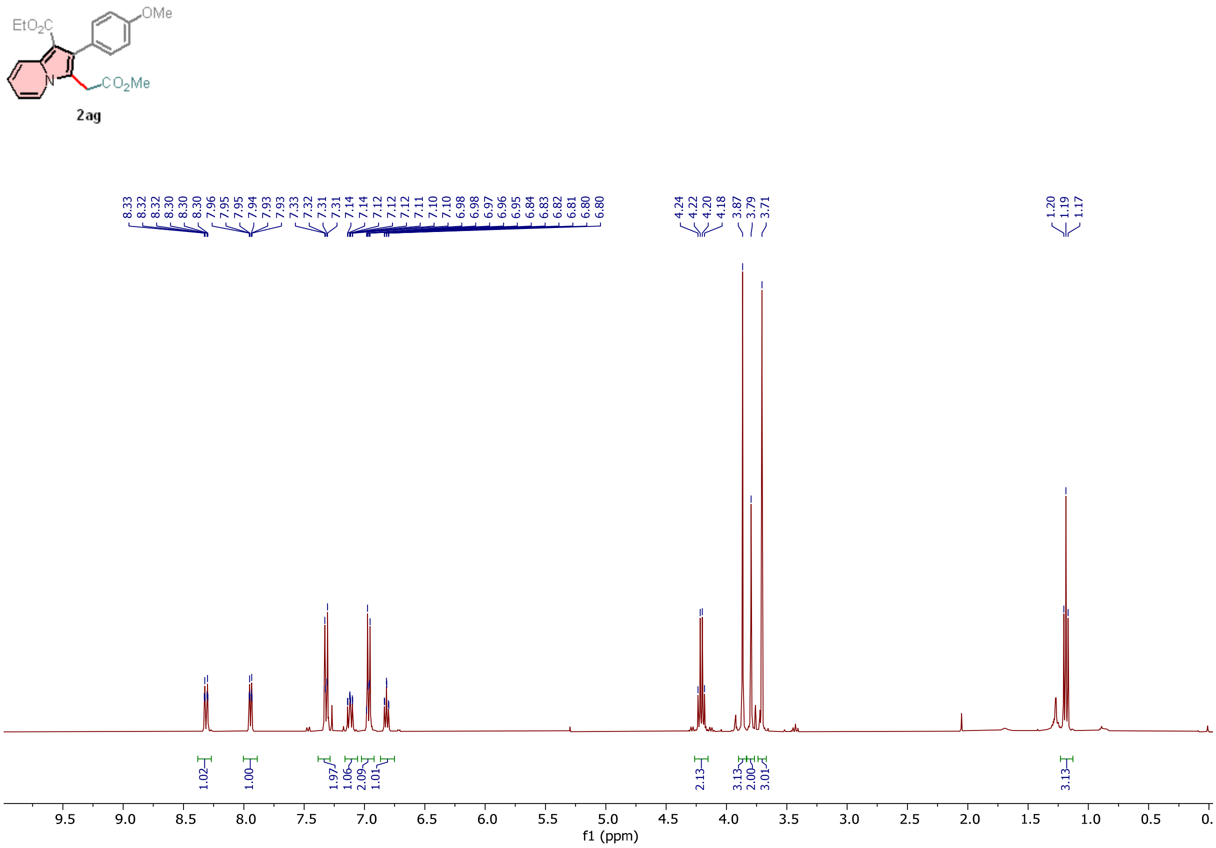

^1H NMR (400 MHz, $CDCl_3$) of **2ag**.

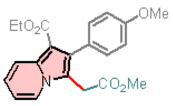

2ag

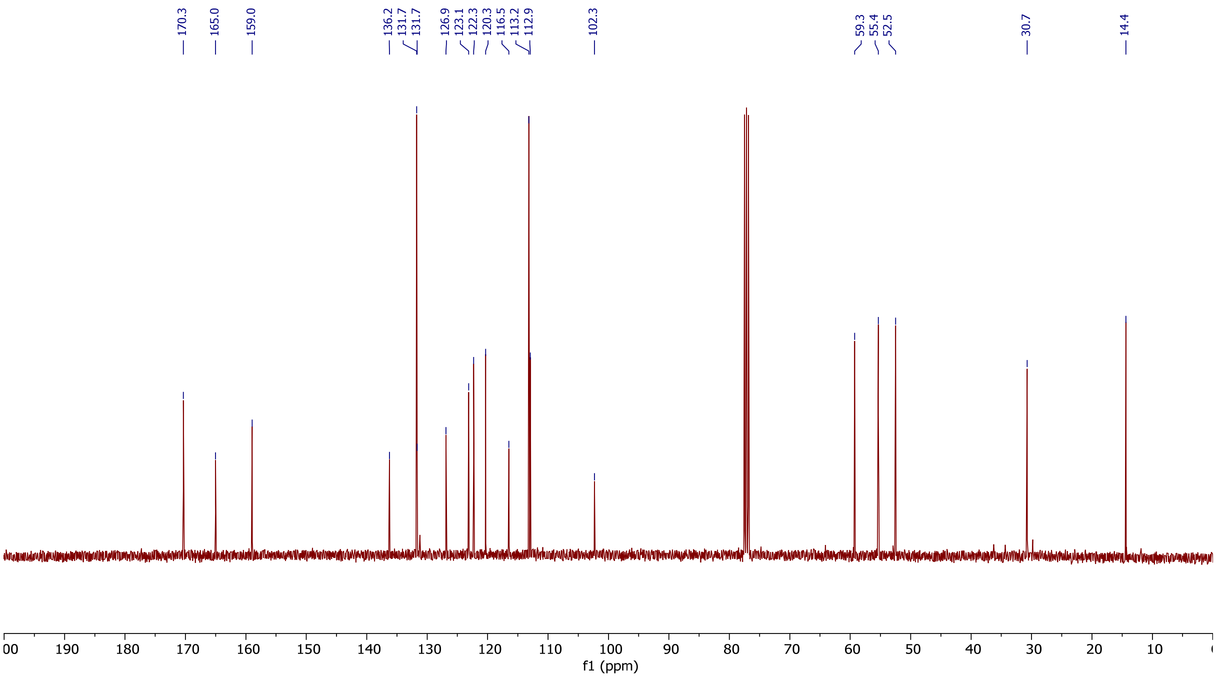

$^{13}C\{^{1}H\}$ NMR (100 MHz, $CDCl_3$) of **2ag**.

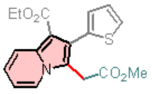

2ah

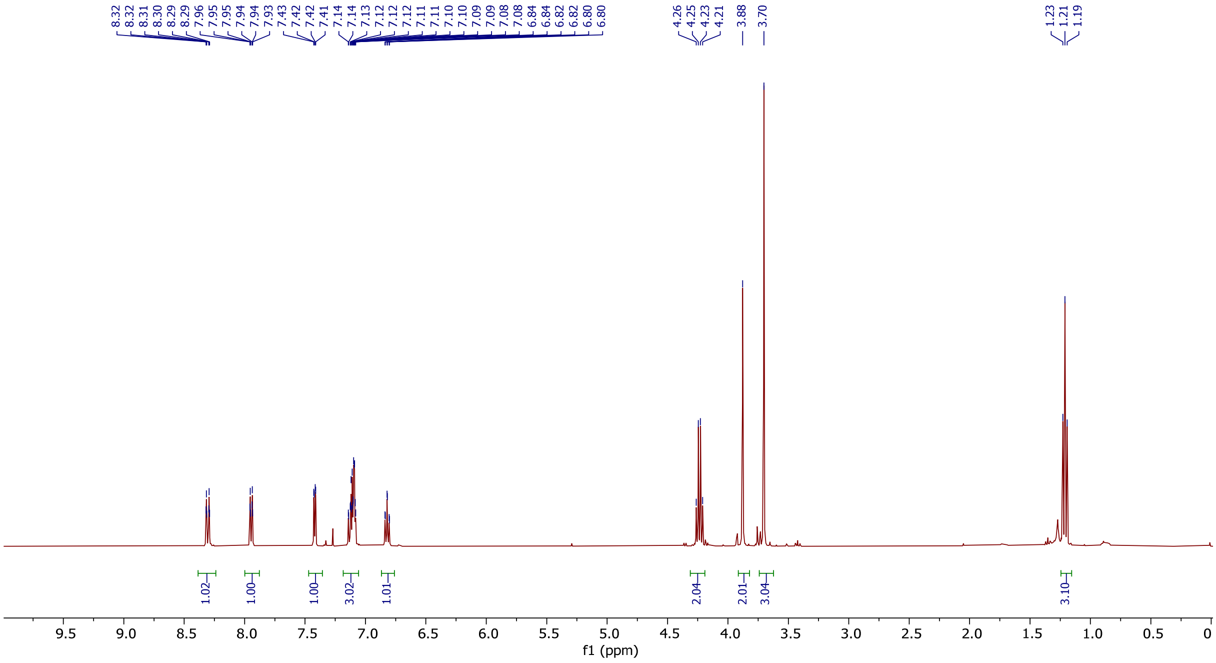

^{1}H NMR (400 MHz, $CDCl_3$) of **2ah**.

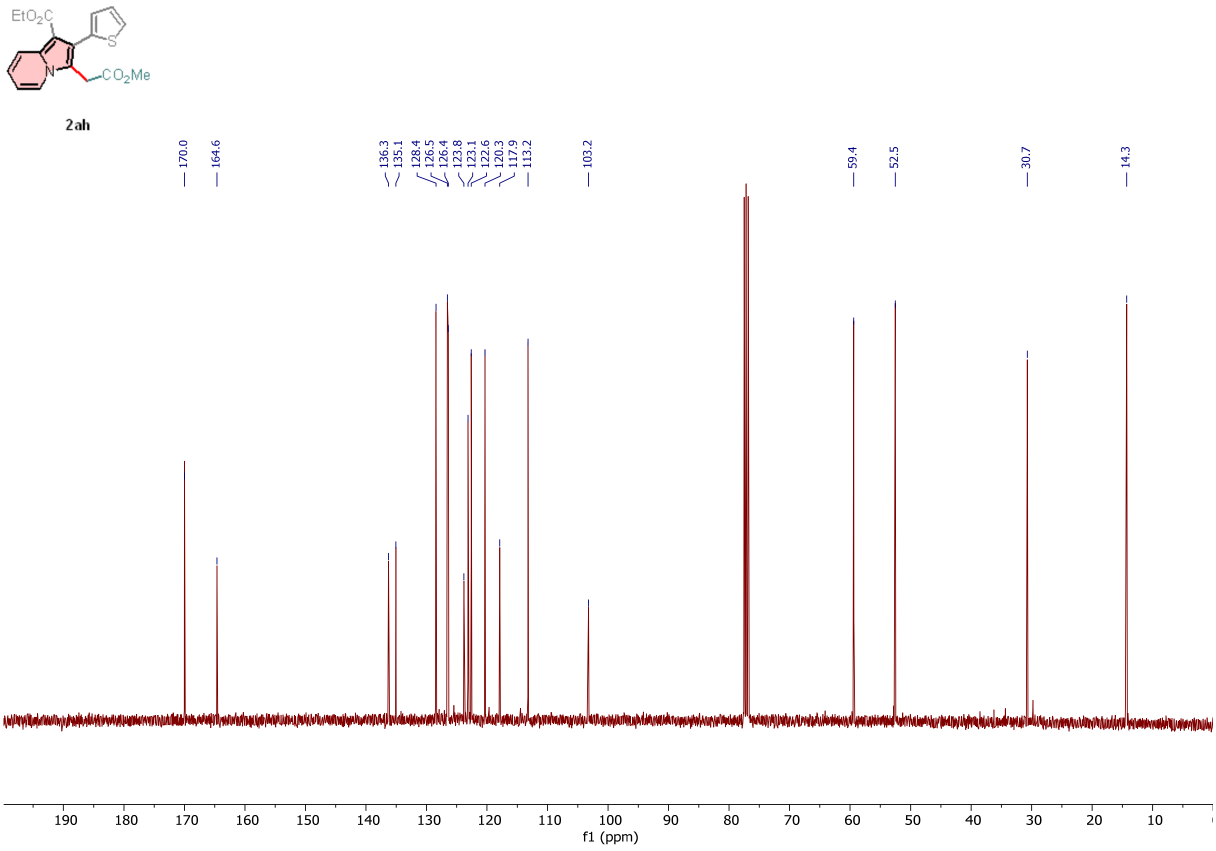

$^{13}C\{^{1}H\}$ NMR (100 MHz, $CDCl_3$) of **2ah**.

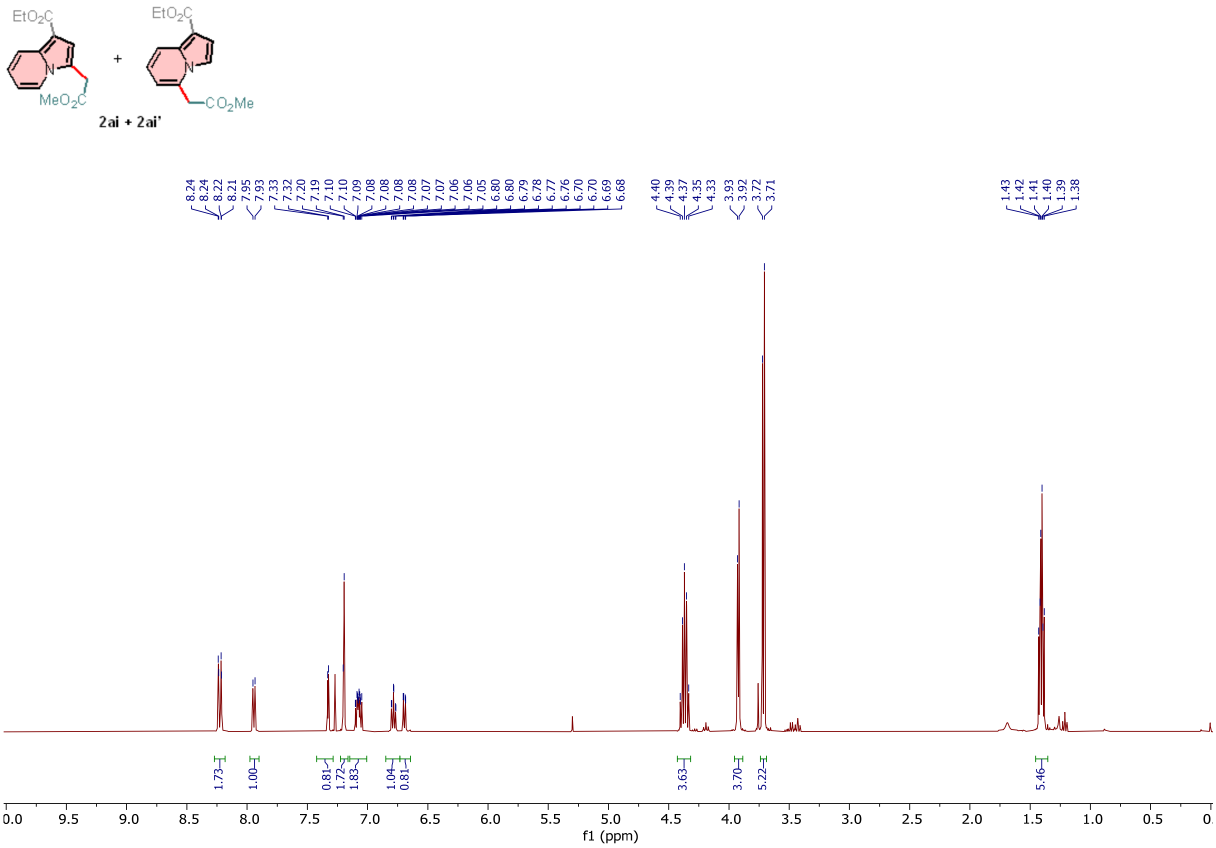

^{1}H NMR (400 MHz, $CDCl_3$) of **2ai + 2ai’**.

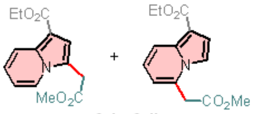

2ai + 2ai'

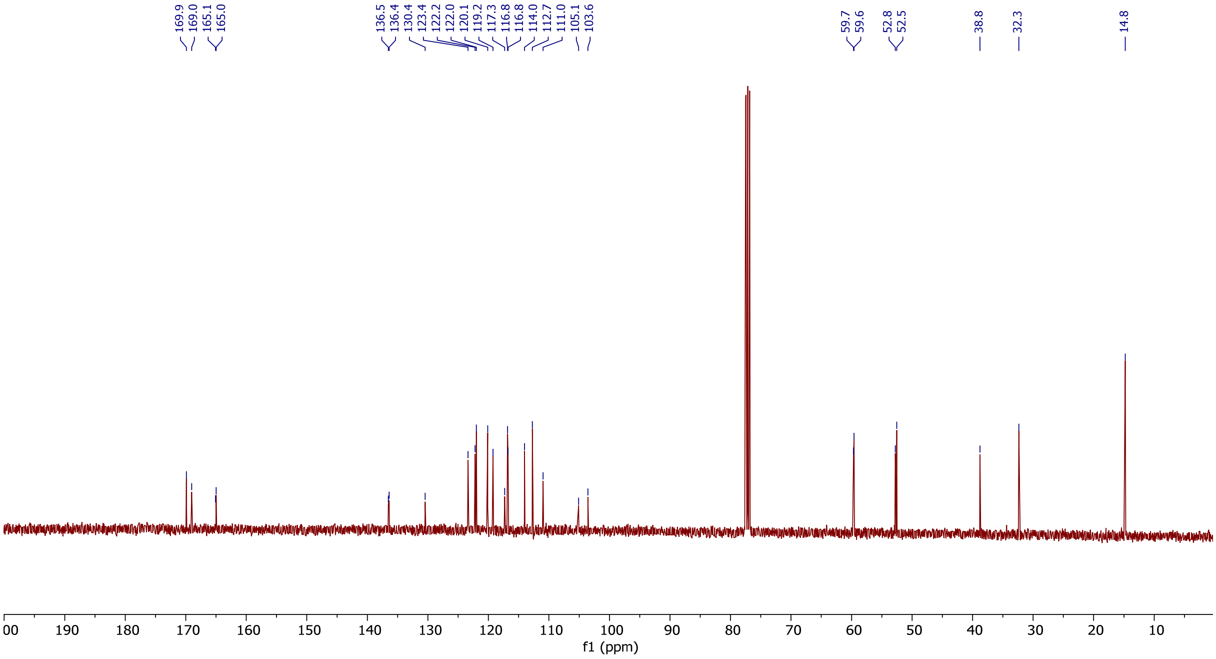

$^{13}C\{^1H\}$ NMR (100 MHz, $CDCl_3$) of **2ai + 2ai'**.

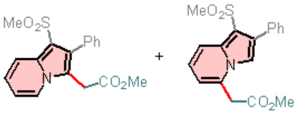

2aj + 2aj'

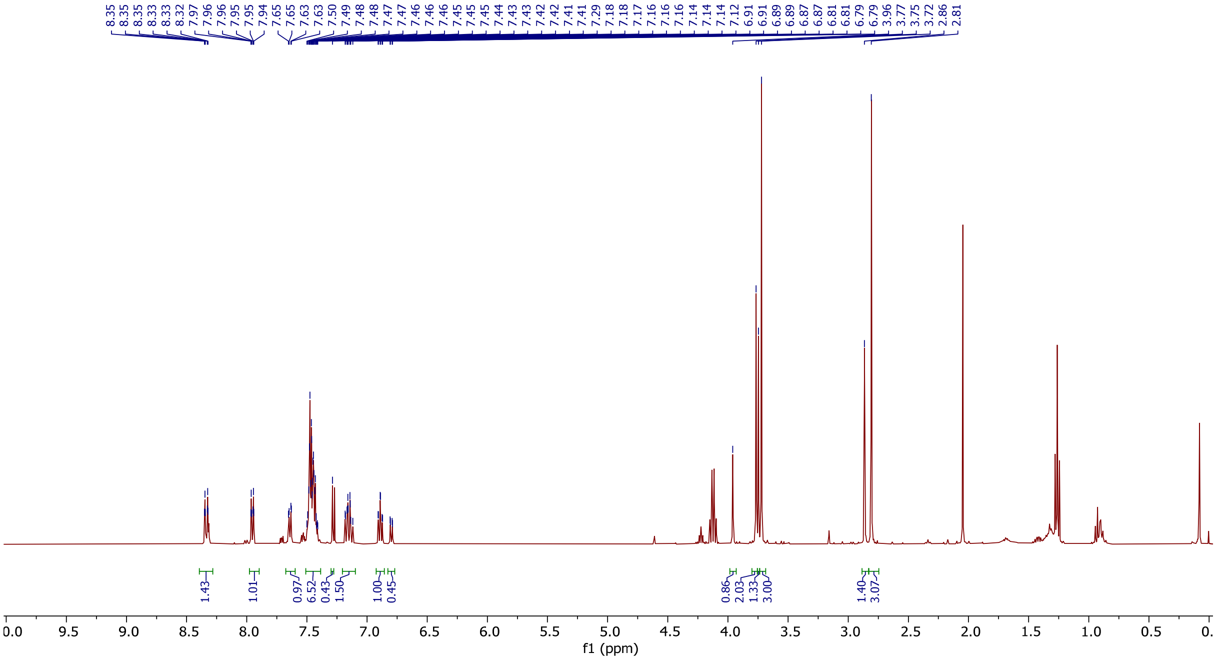

^1H NMR (400 MHz, $CDCl_3$) of **2aj + 2aj'**.

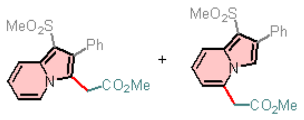

2aj + **2aj'**

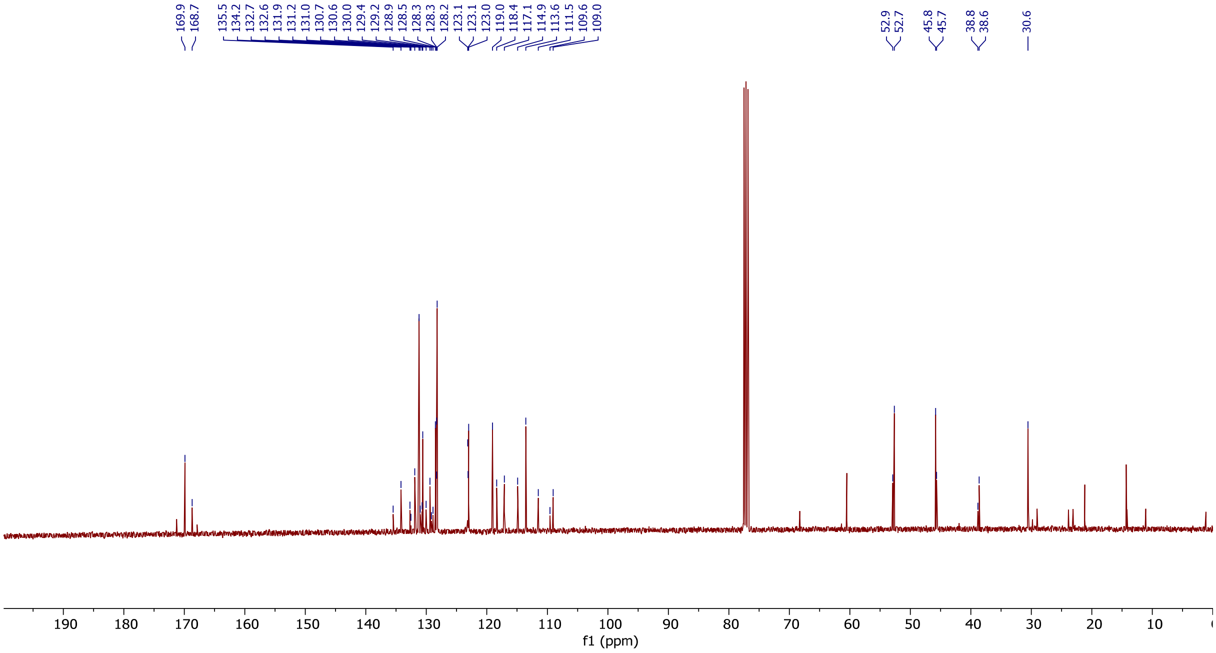

$^{13}C\{^{1}H\}$ NMR (100 MHz, $CDCl_3$) of **2aj + 2aj'**.

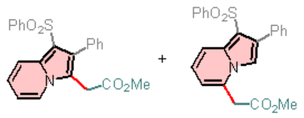

2ak + **2ak'**

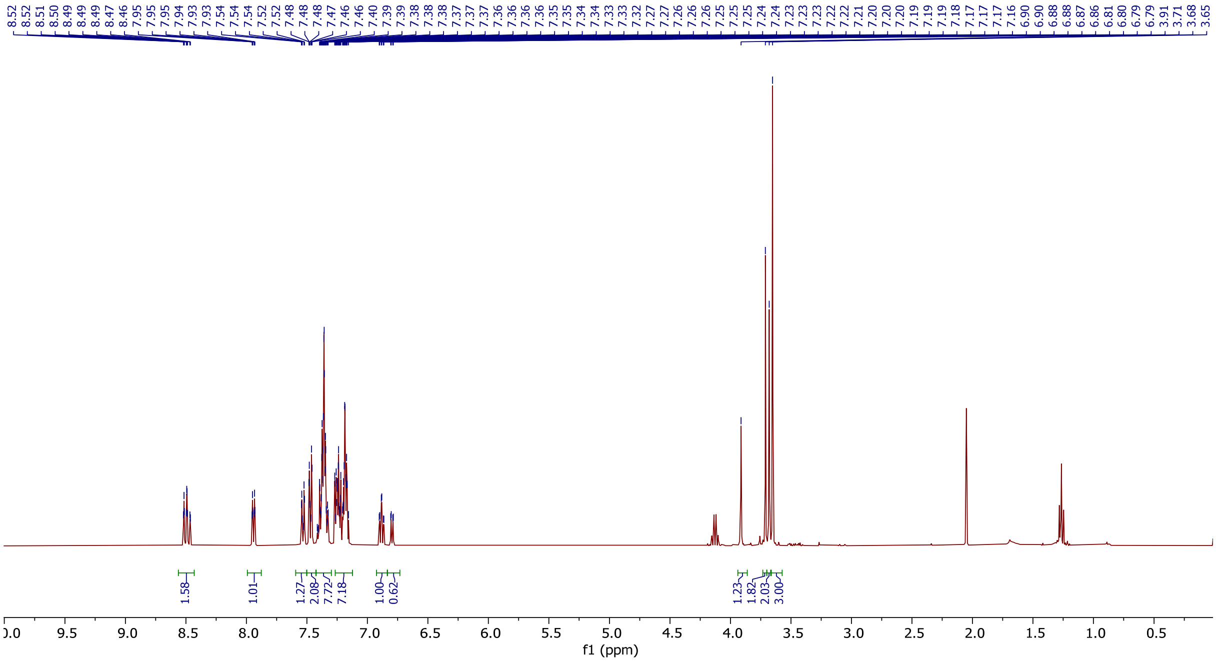

^{1}H NMR (400 MHz, $CDCl_3$) of **2ak + 2ak'**.

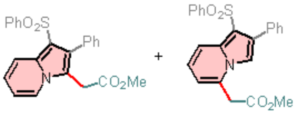

2ak + 2ak'

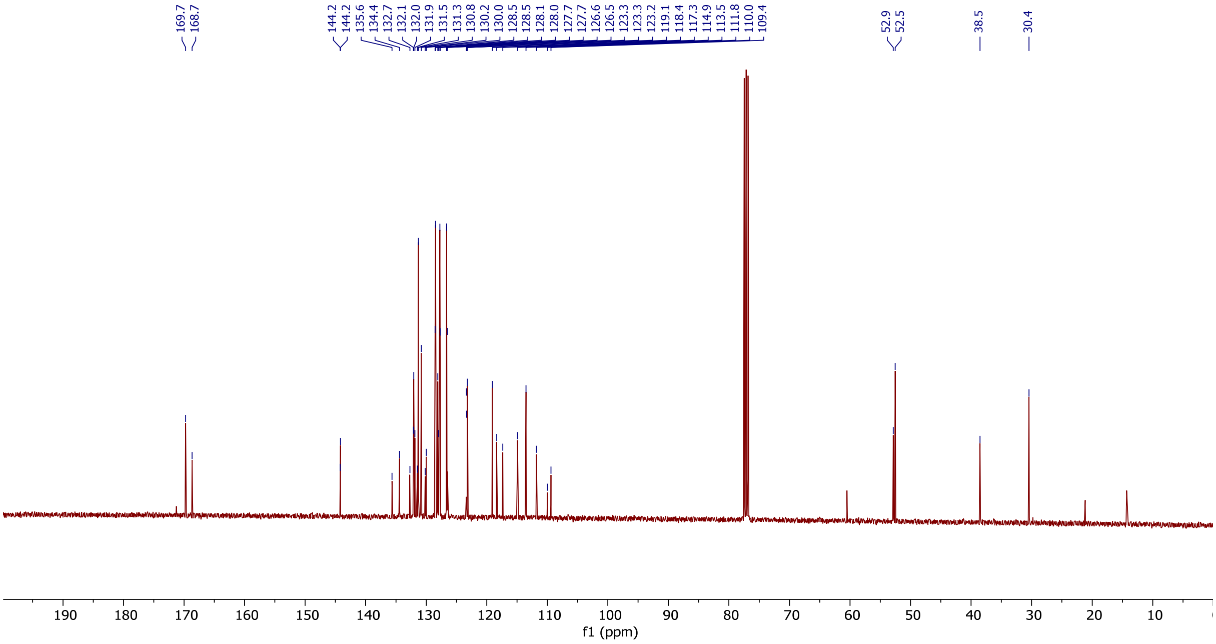

$^{13}C\{^1H\}$ NMR (100 MHz, $CDCl_3$) of **2ak + 2ak'**.

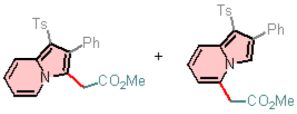

2al + 2al'

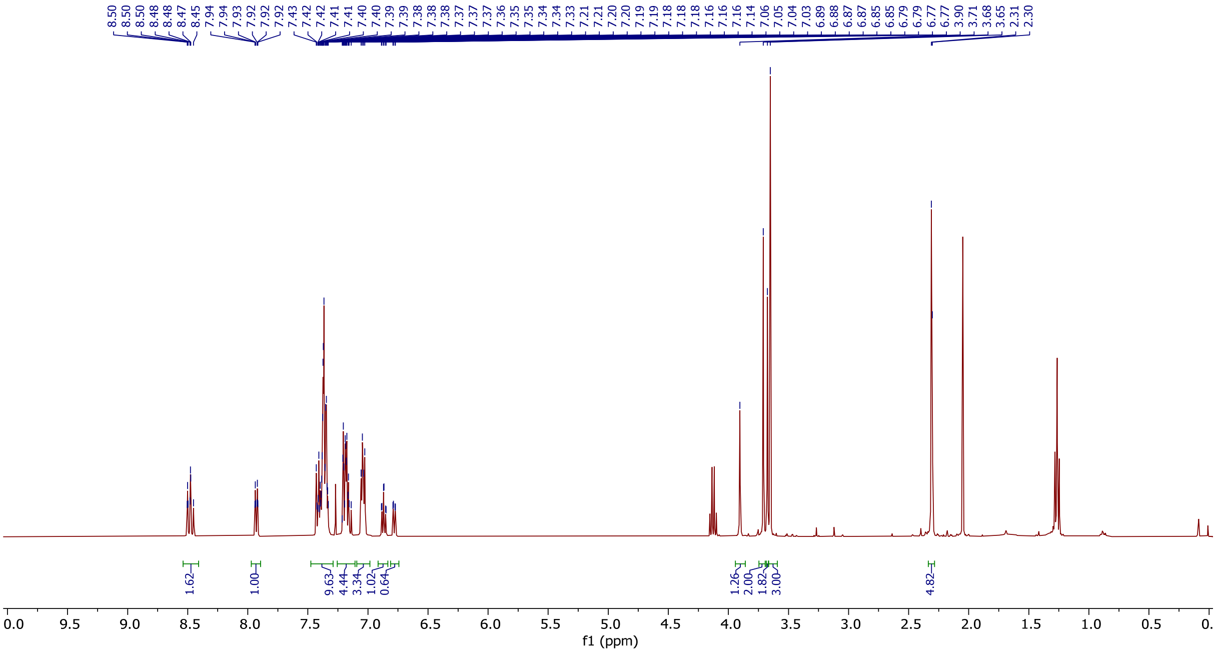

^1H NMR (400 MHz, $CDCl_3$) of **2al + 2al'**.

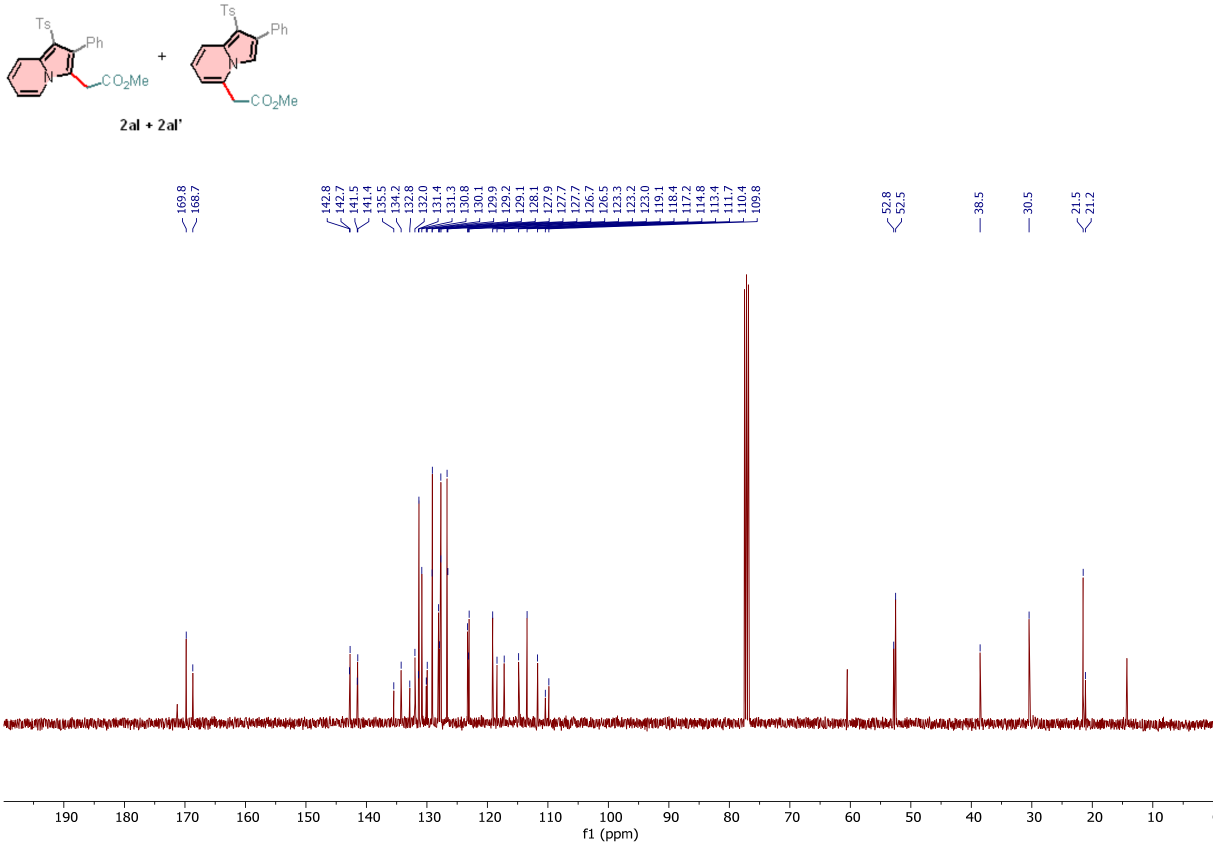

$^{13}C\{^{1}H\}$ NMR (100 MHz, $CDCl_3$) of **2al + 2al'**.

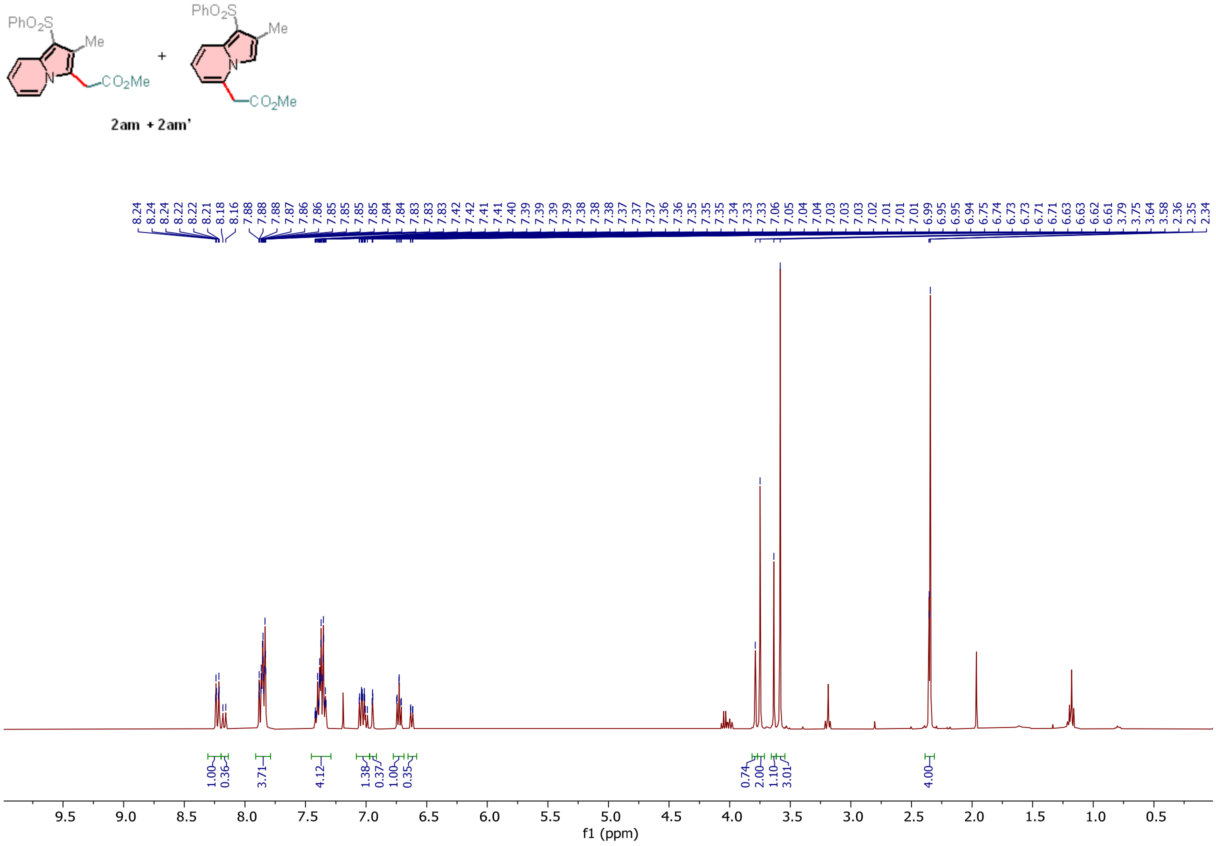

^{1}H NMR (400 MHz, $CDCl_3$) of **2am + 2am'**.

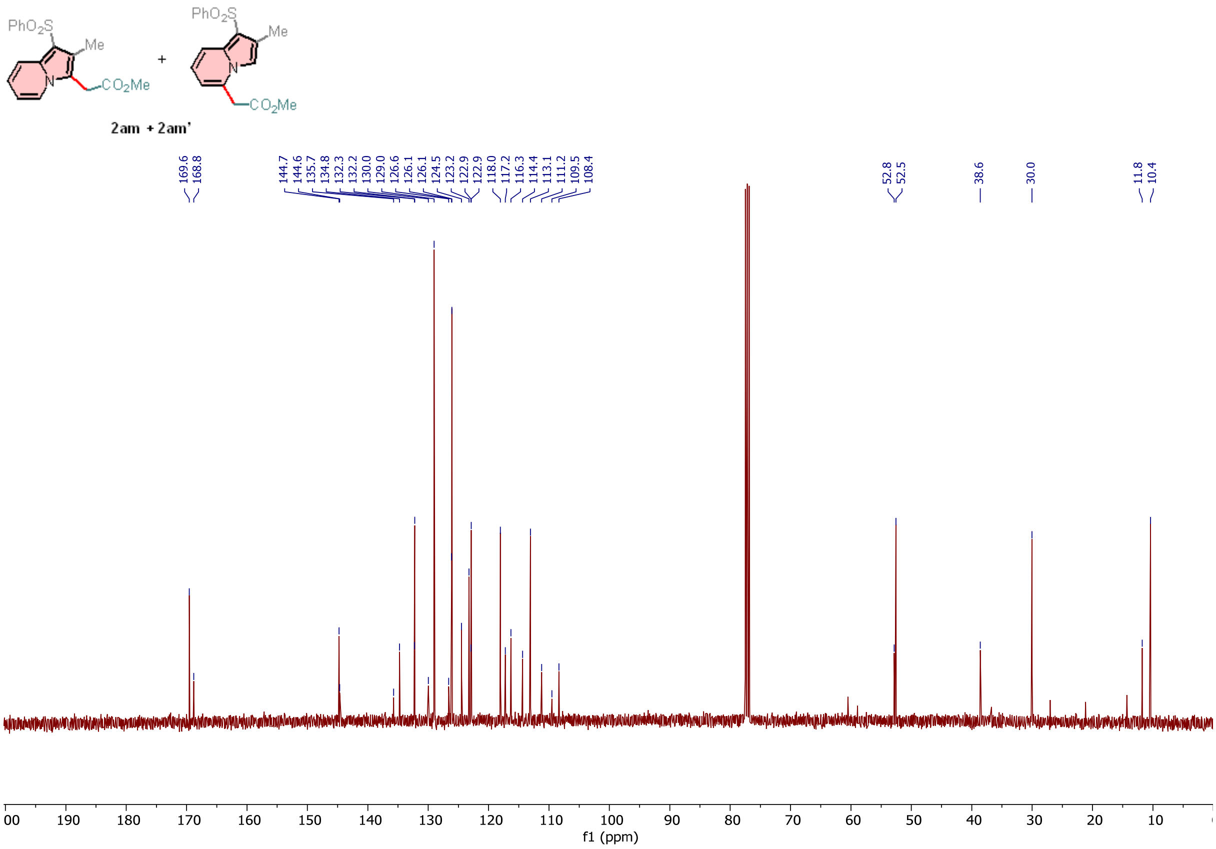

$^{13}C\{^{1}H\}$ NMR (100 MHz, $CDCl_3$) of **2am + 2am'**.

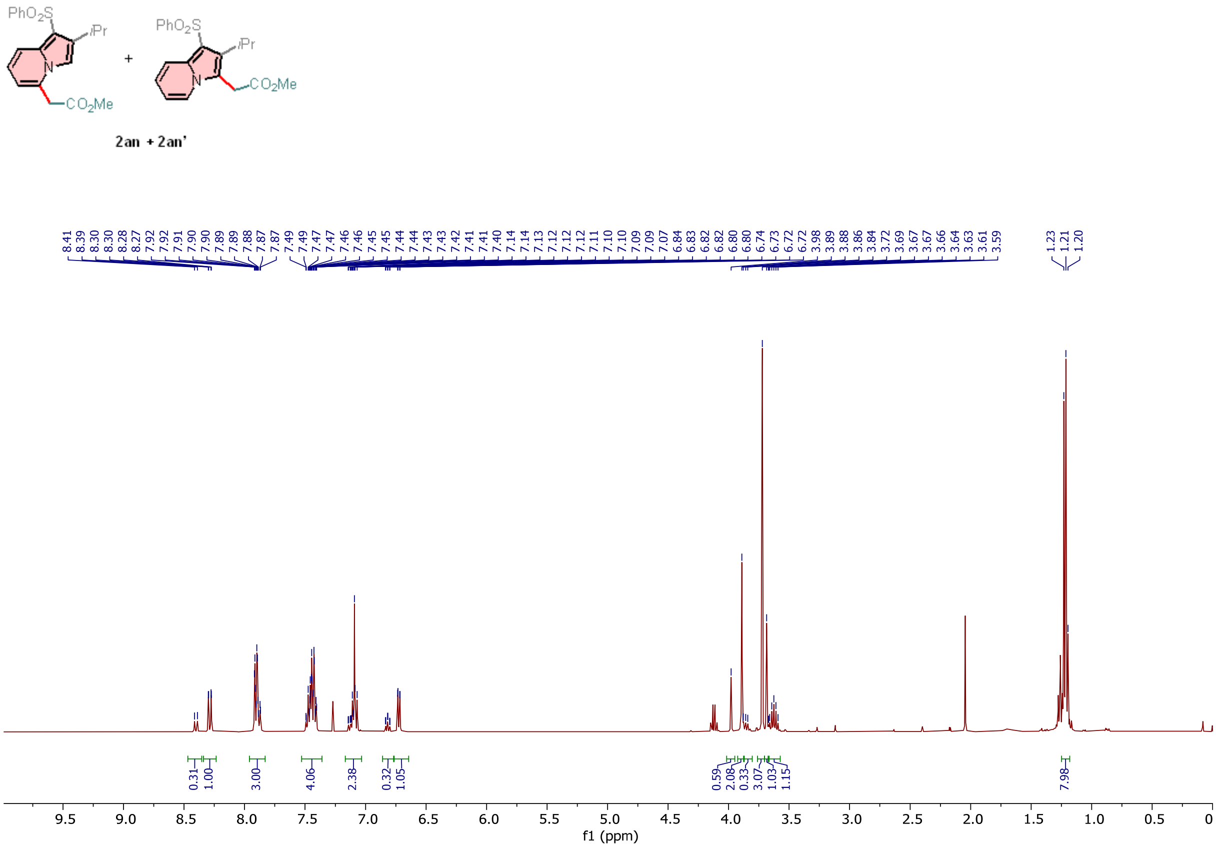

^{1}H NMR (400 MHz, $CDCl_3$) of **2an + 2an'**.

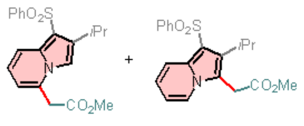

2an + 2an'

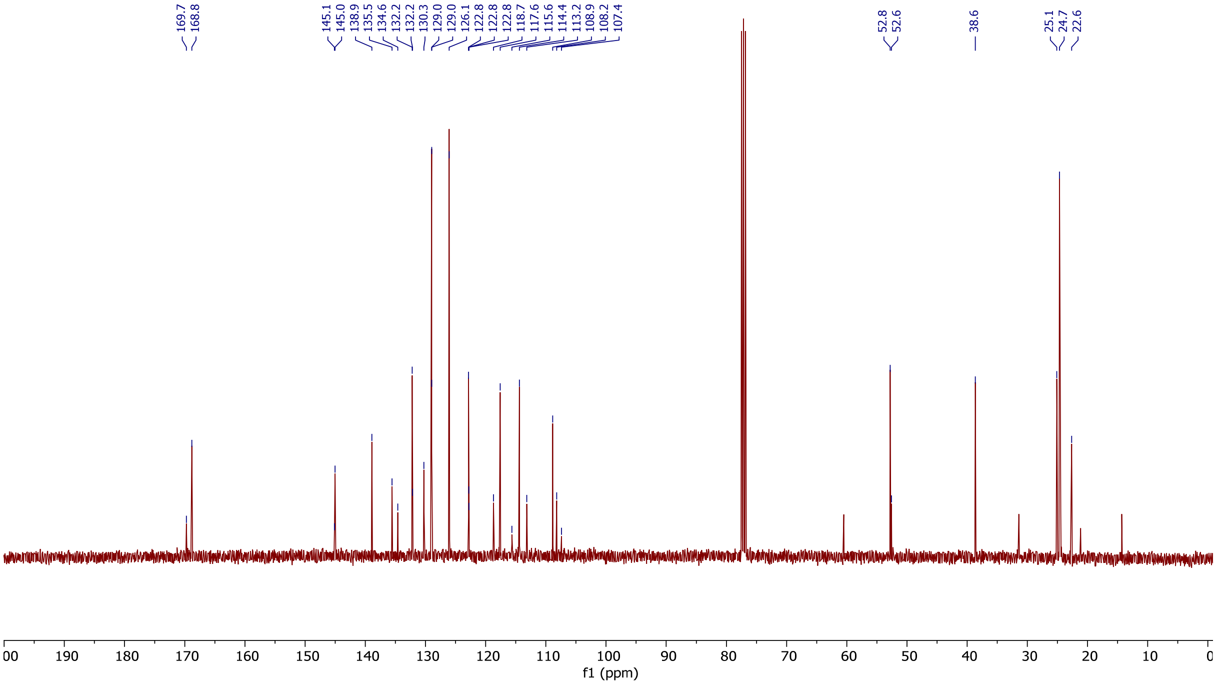

$^{13}C\{^{1}H\}$ NMR (100 MHz, $CDCl_3$) of **2an + 2an'**.

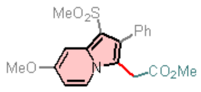

2ao

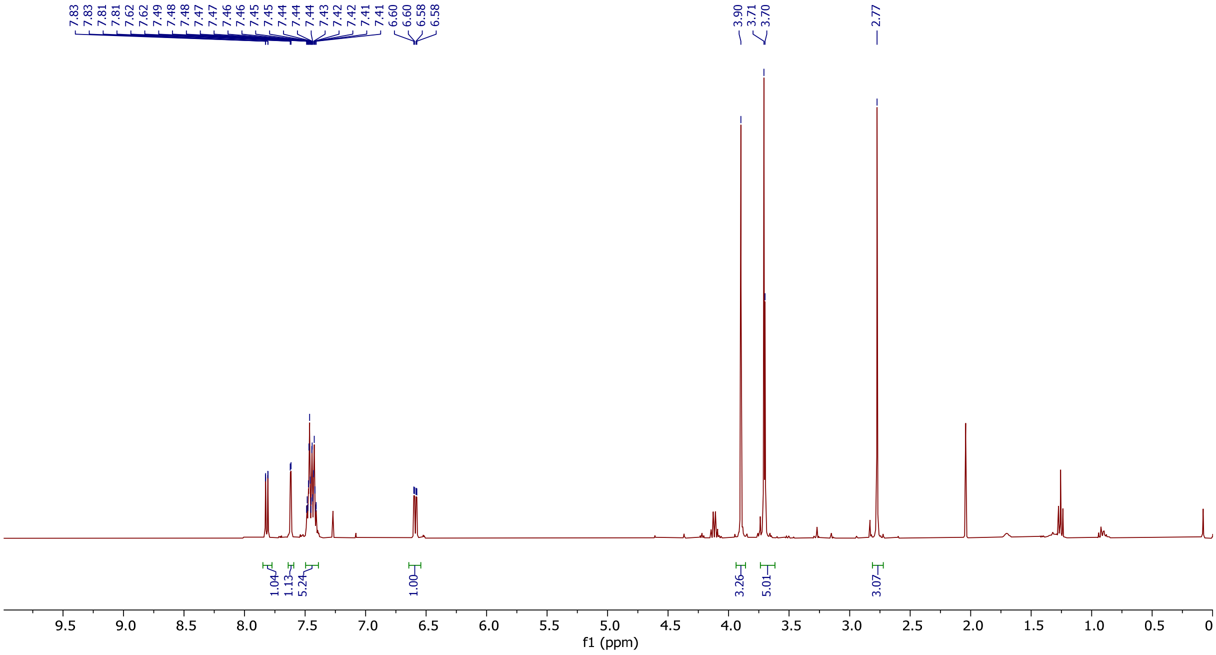

^{1}H NMR (400 MHz, $CDCl_3$) of **2ao**.

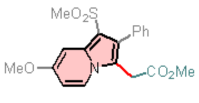

2ao

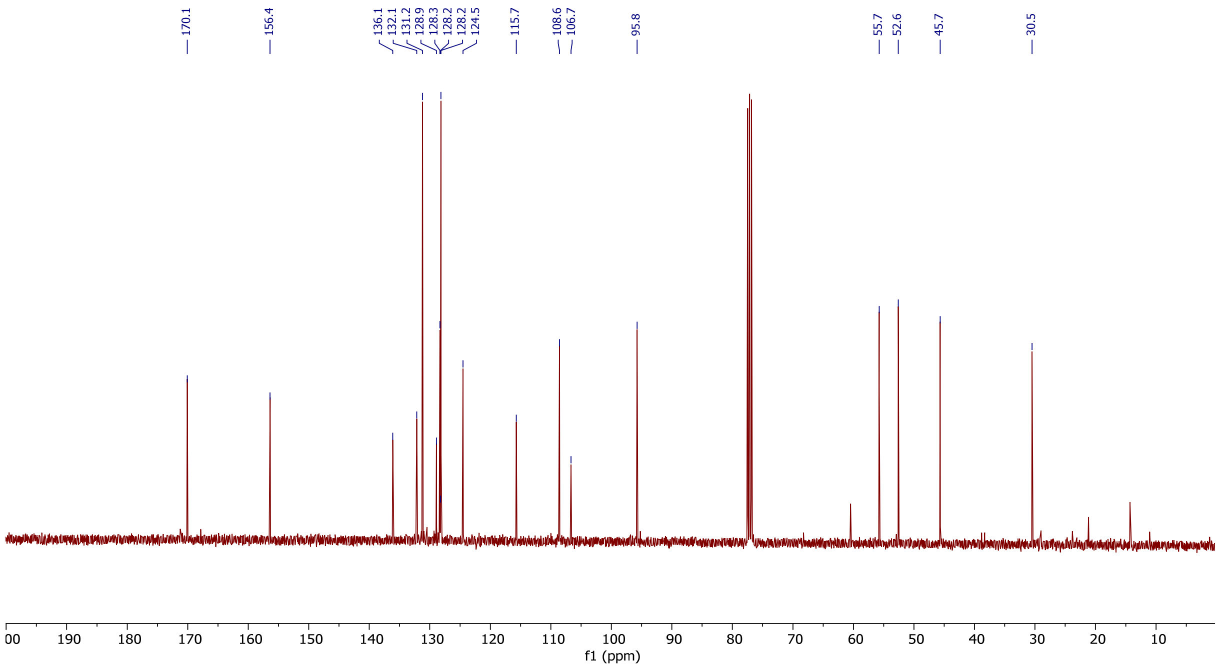

$^{13}C\{^{1}H\}$ NMR (100 MHz, $CDCl_3$) of **2ao**.

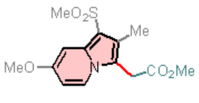

2ap

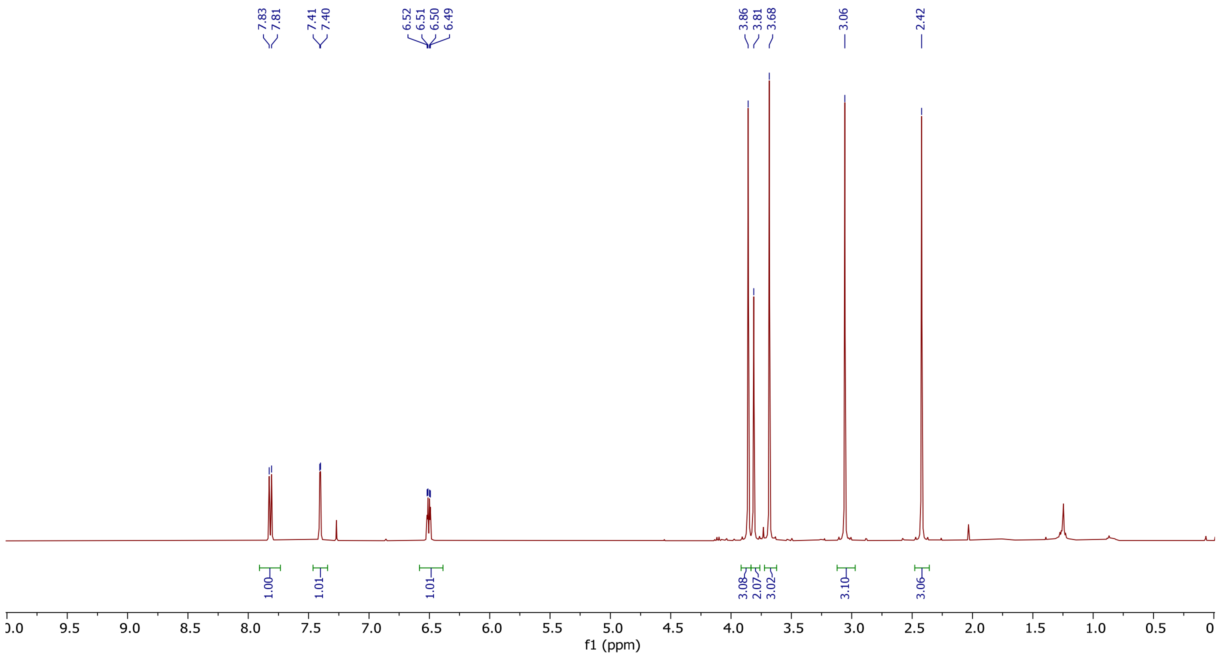

^{1}H NMR (400 MHz, $CDCl_3$) of **2ap**.

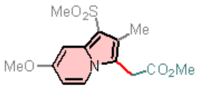

2ap

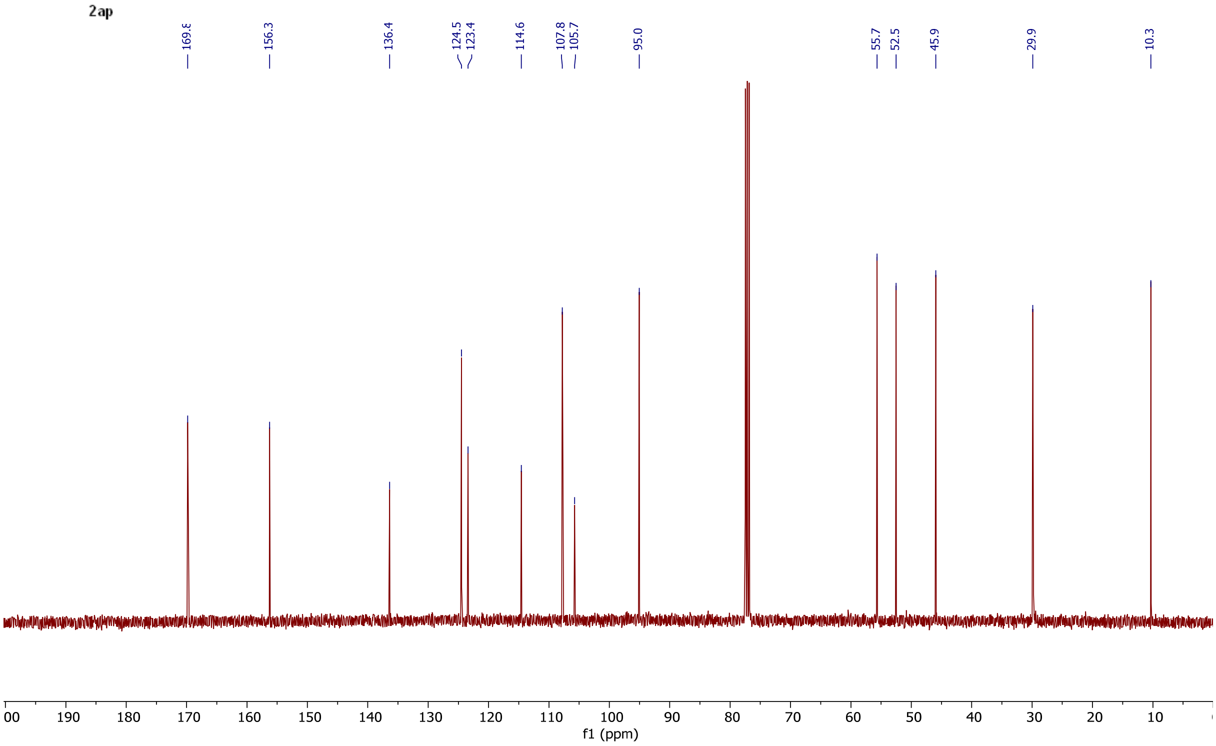

$^{13}C\{^{1}H\}$ NMR (100 MHz, $CDCl_3$) of **2ap**.

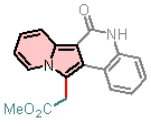

2aq

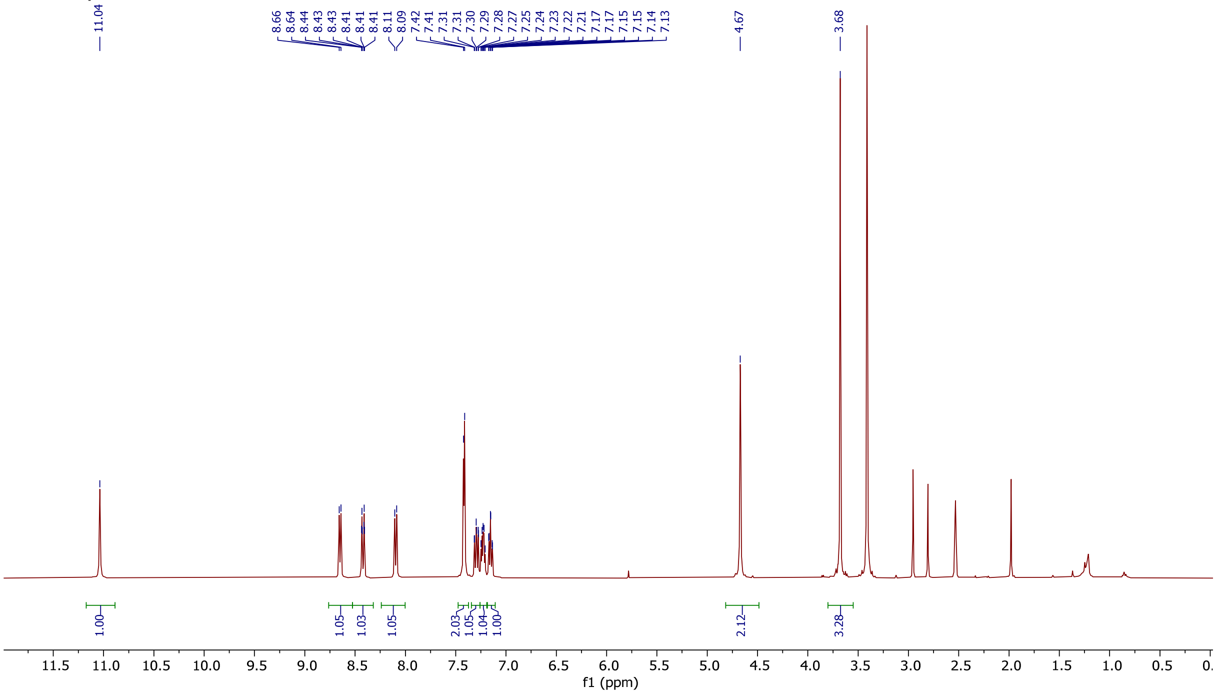

^{1}H NMR (400 MHz, DMSO-d^{6}) of **2aq**.

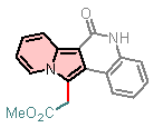

2aq

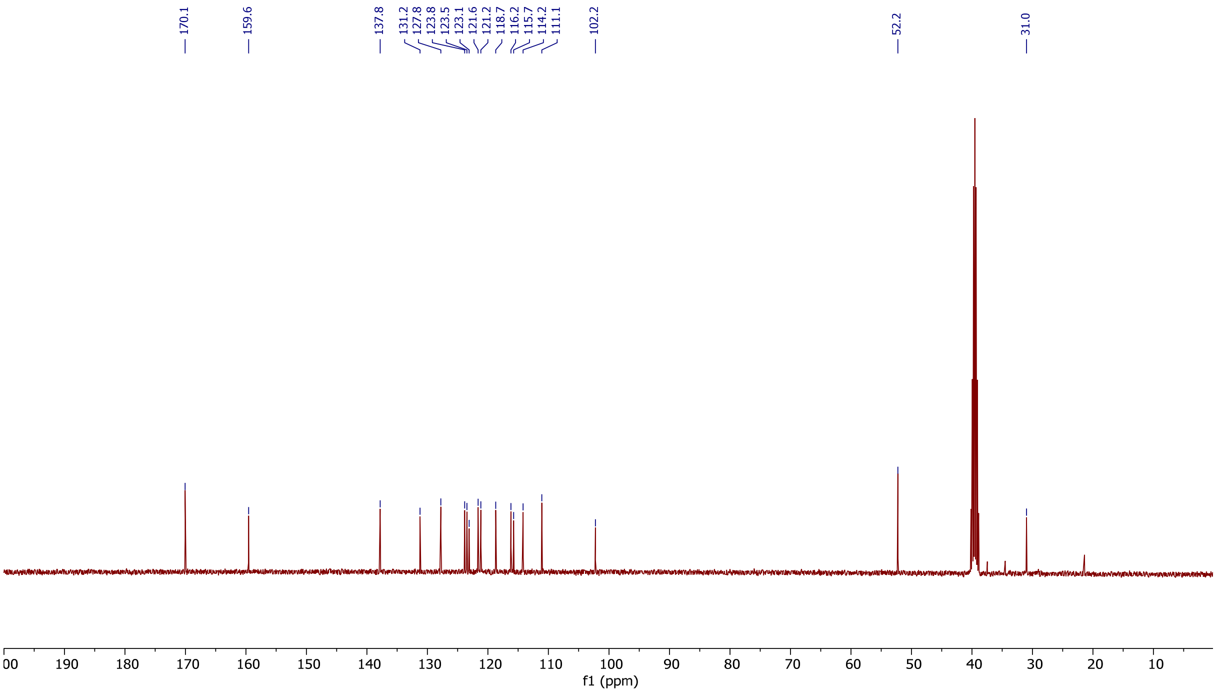

$^{13}C\{^{1}H\}$ NMR (100 MHz, DMSO-d^{6}) of **2aq**.

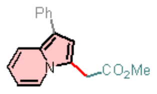

2ar

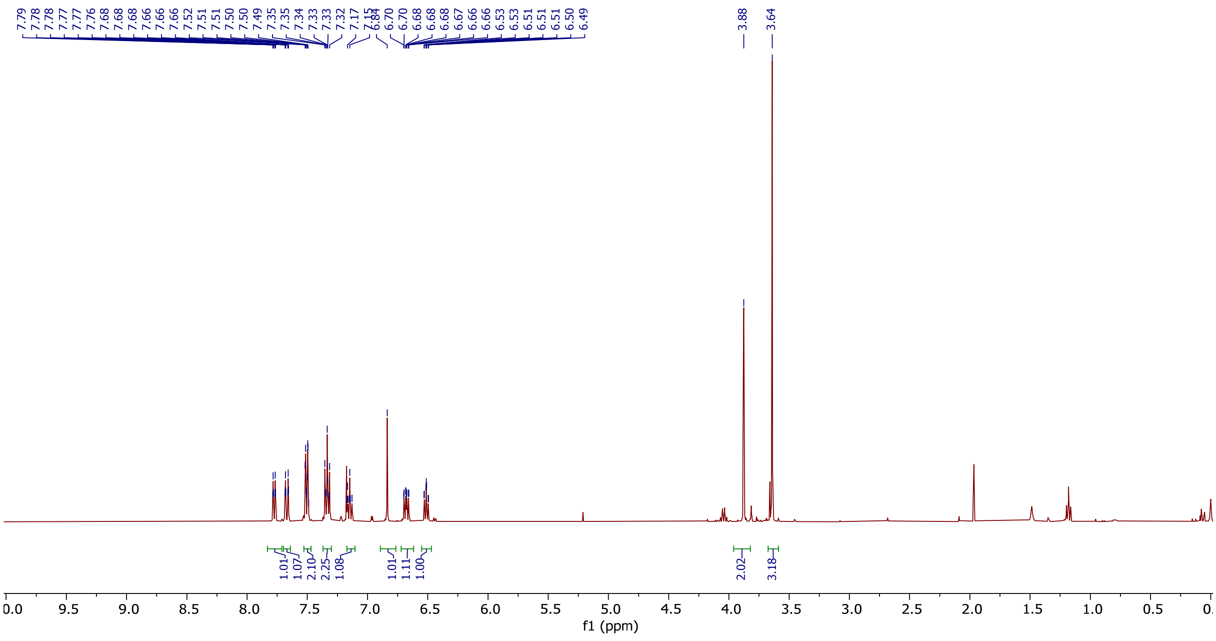

^{1}H NMR (400 MHz, $CDCl_3$) of **2ar**.

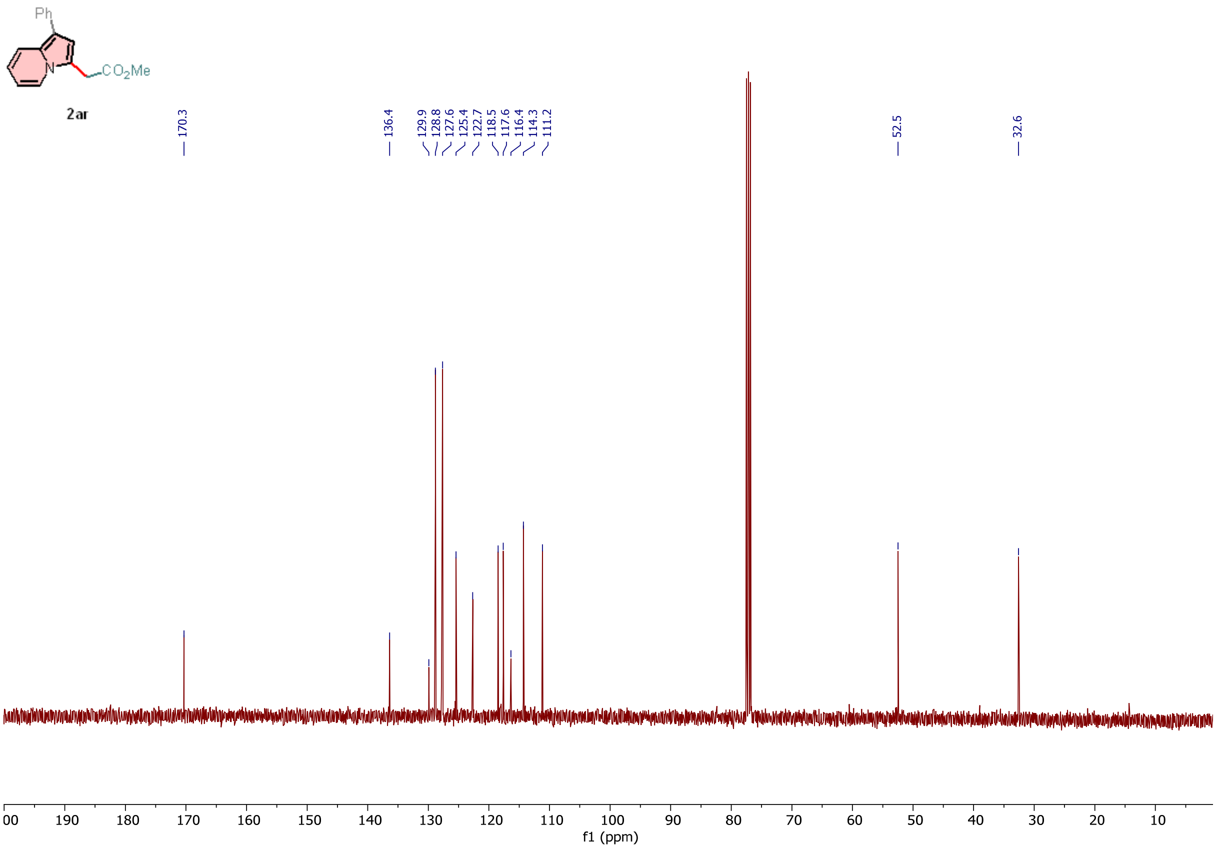

$^{13}C\{^{1}H\}$ NMR (100 MHz, $CDCl_3$) of **2ar**.

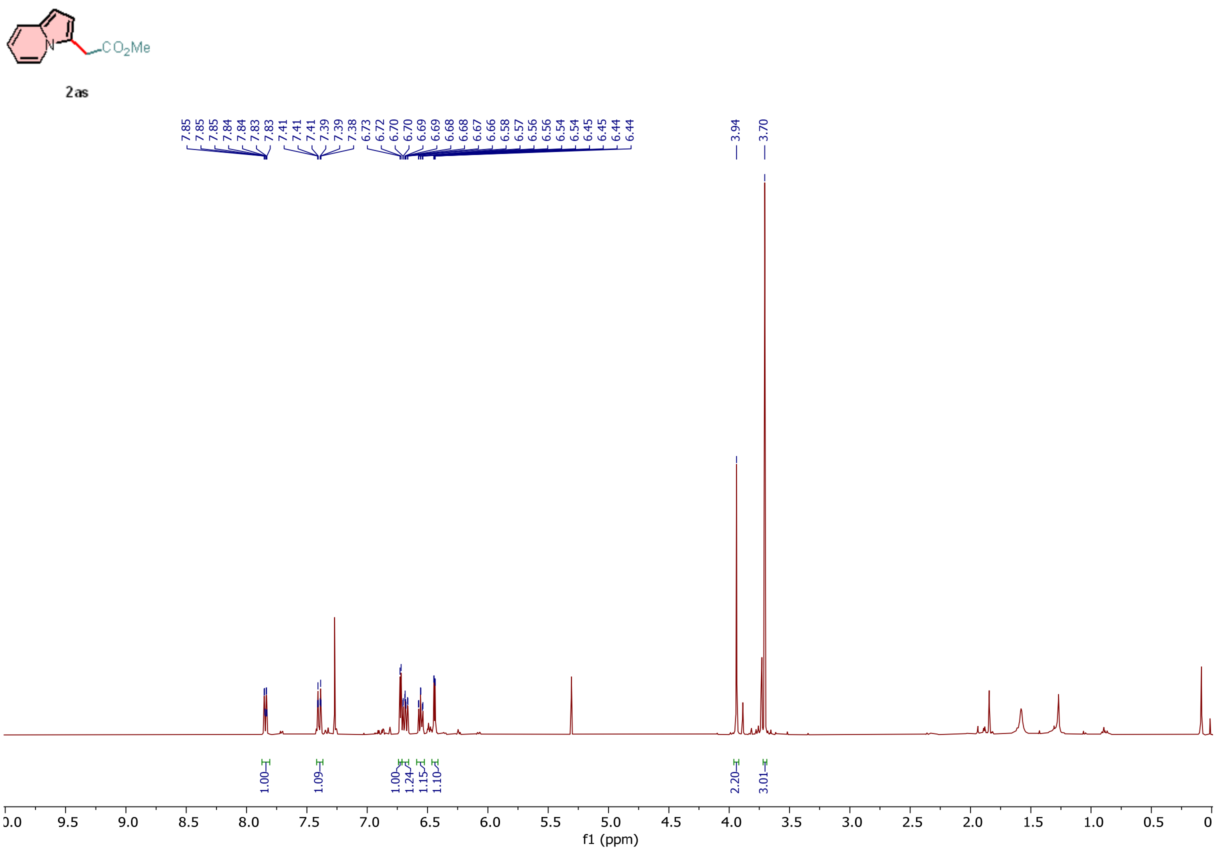

^{1}H NMR (400 MHz, $CDCl_3$) of **2as**.

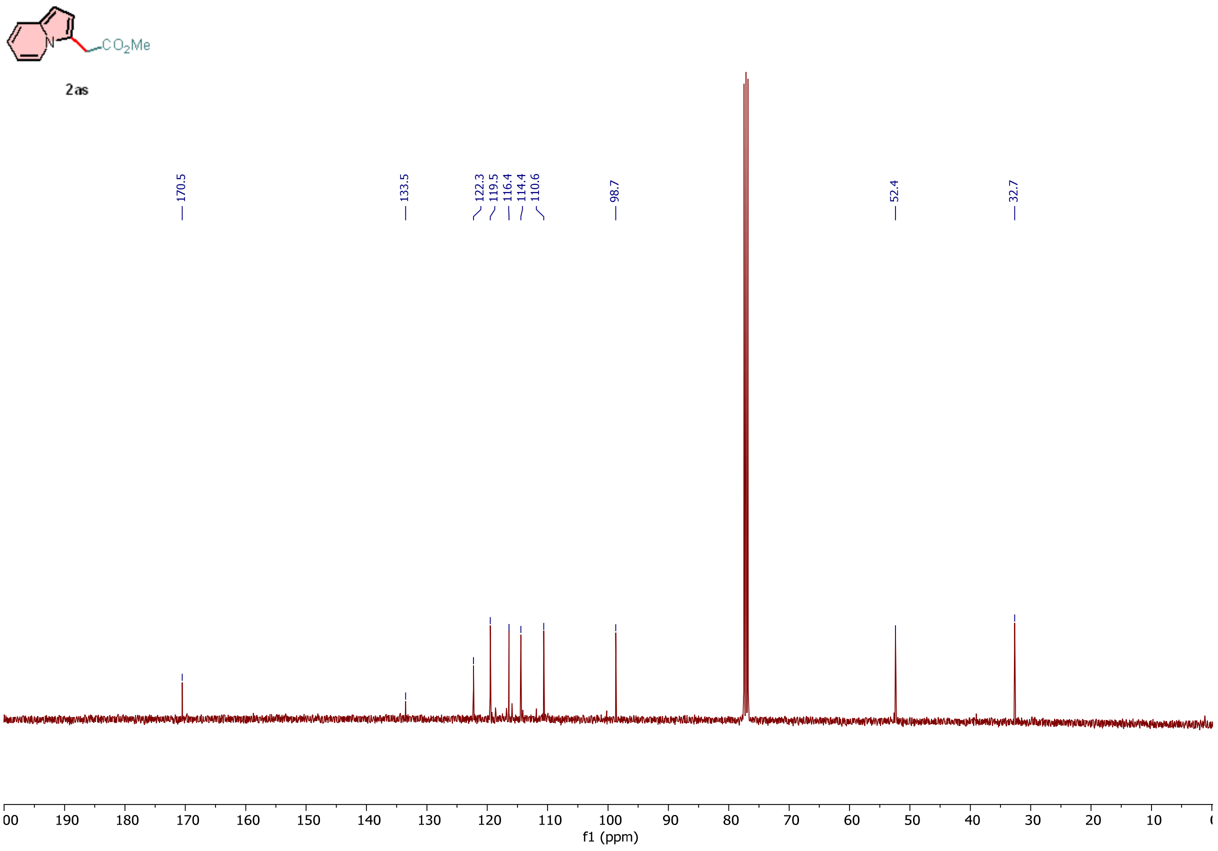

$^{13}C\{^{1}H\}$ NMR (100 MHz, $CDCl_3$) of **2as**.

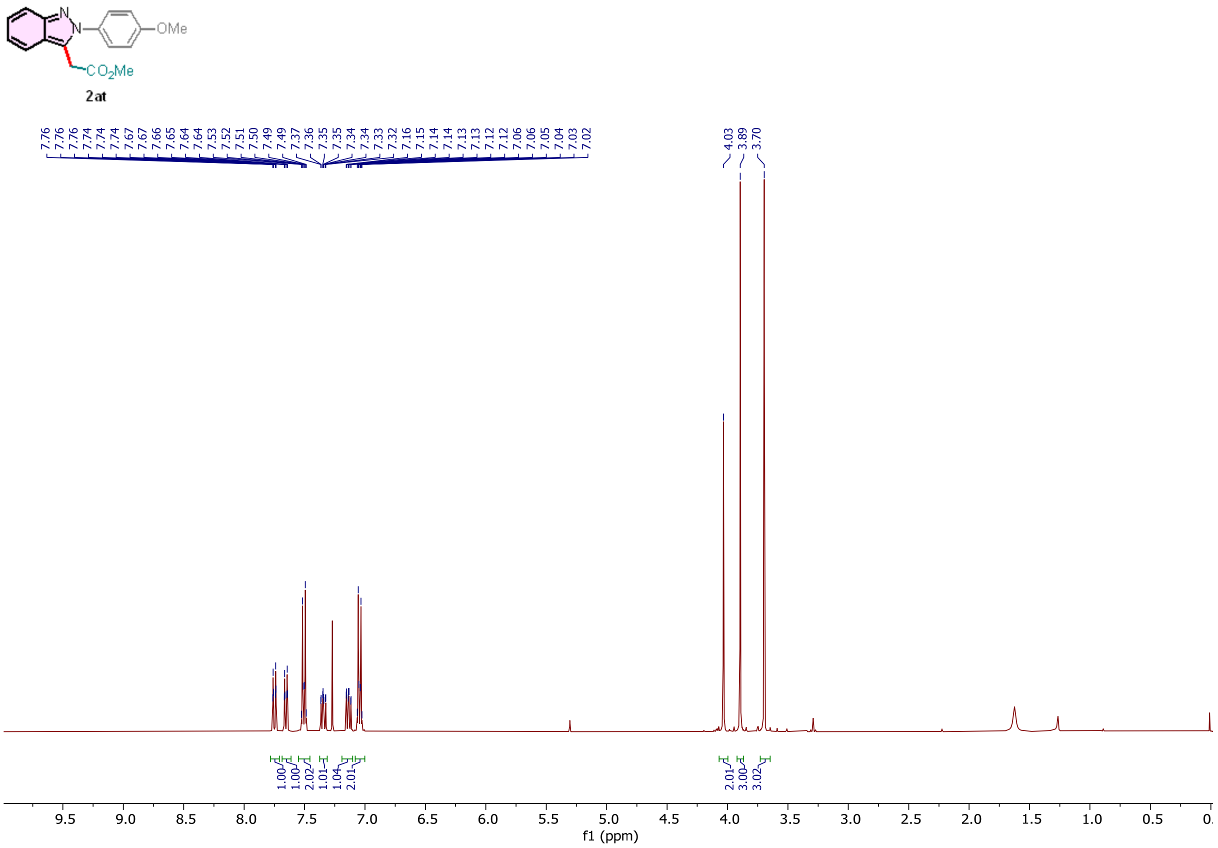

^{1}H NMR (400 MHz, $CDCl_3$) of **2at**.

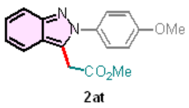

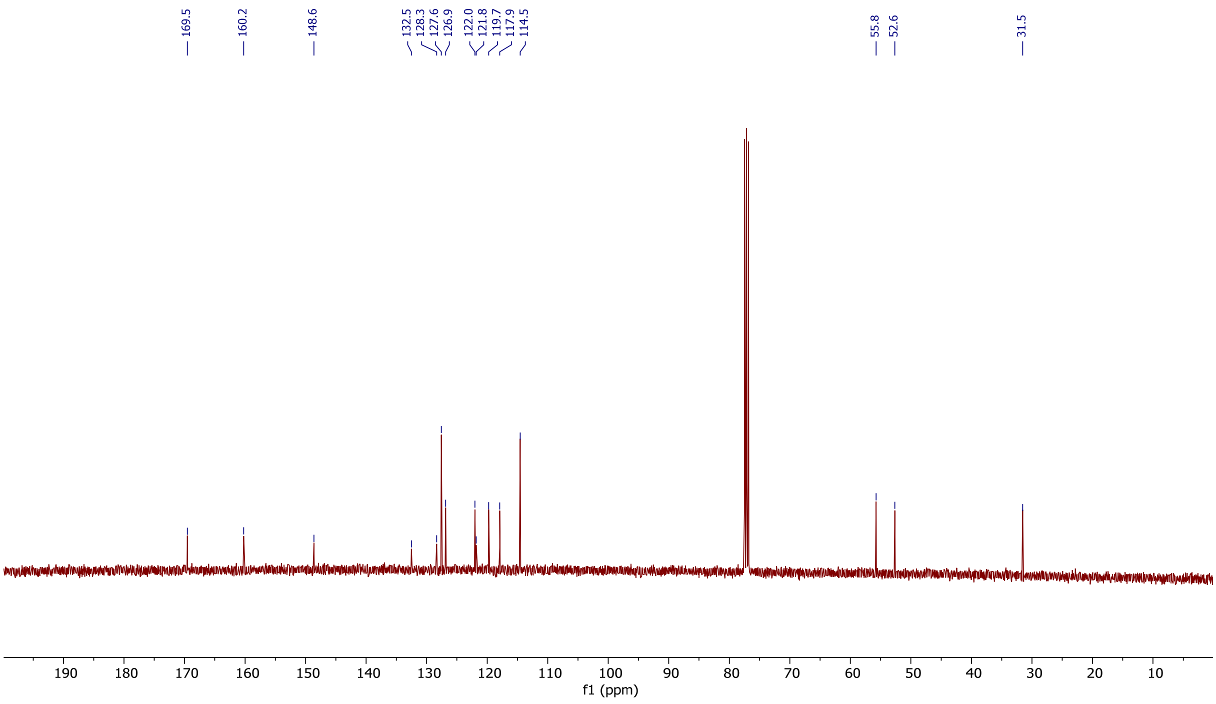

$^{13}C\{^{1}H\}$ NMR (100 MHz, $CDCl_3$) of **2at**.

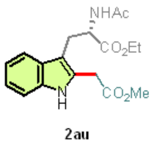

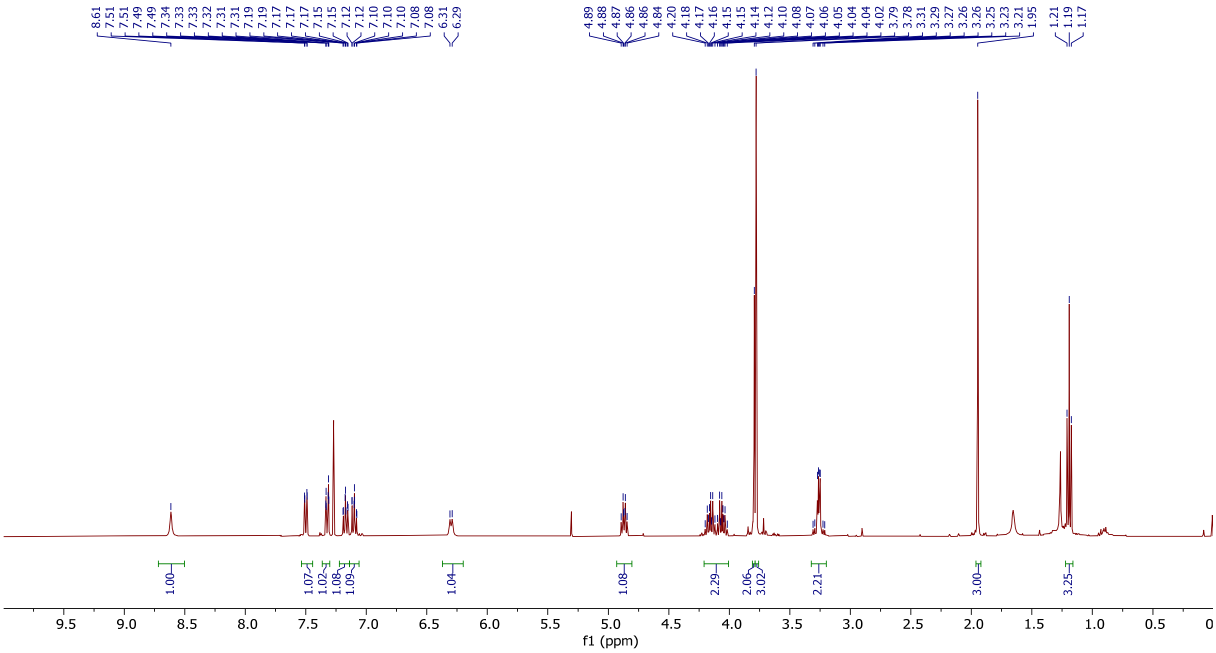

^{1}H NMR (400 MHz, $CDCl_3$) of **2au**.

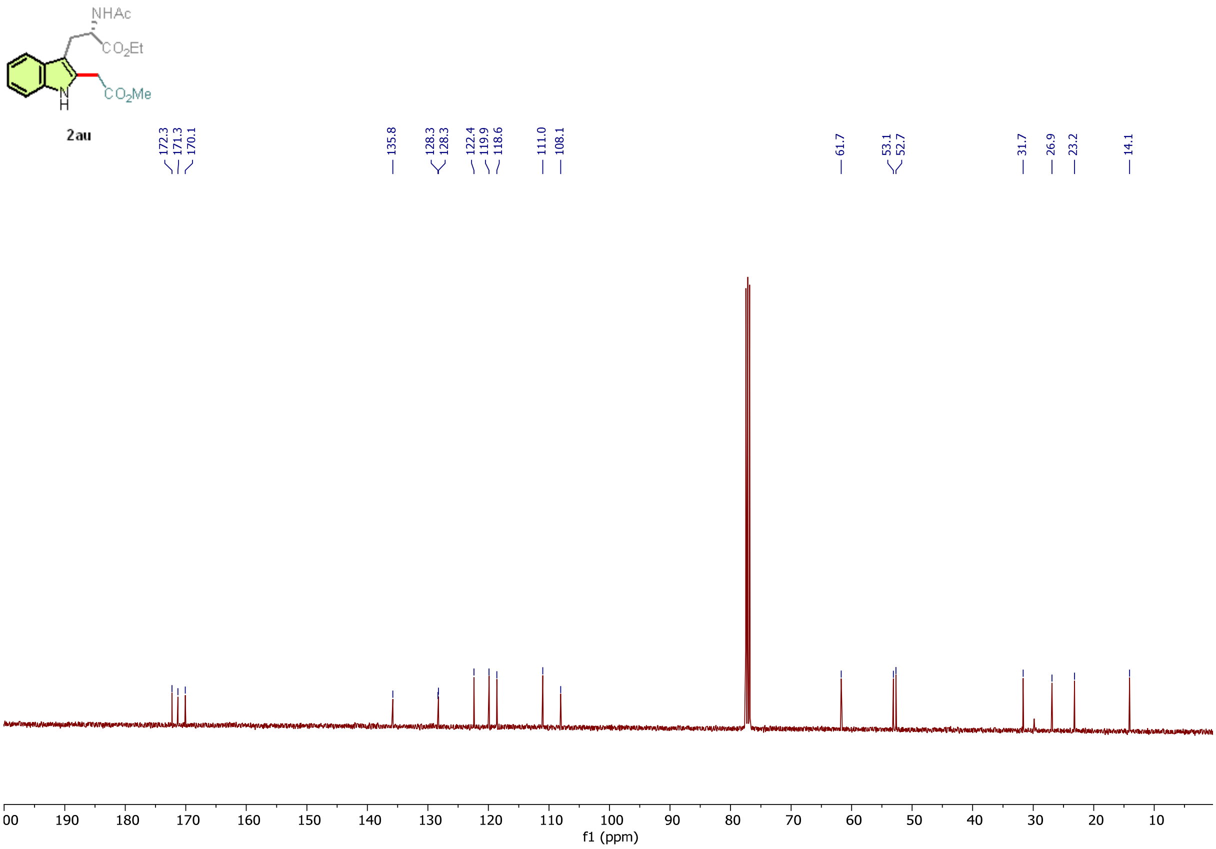

$^{13}C\{^{1}H\}$ NMR (100 MHz, $CDCl_3$) of **2au**.

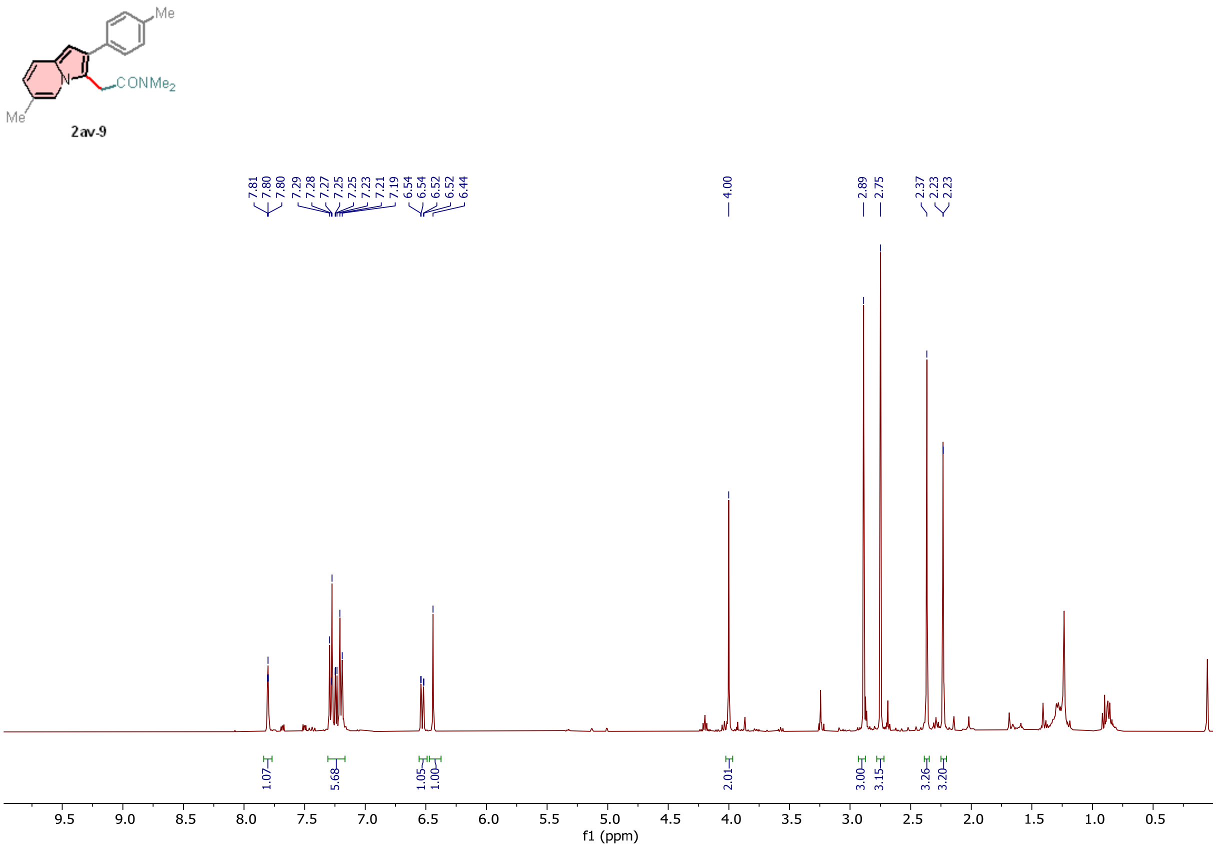

^{1}H NMR (400 MHz, $CDCl_3$) of **2av-9**.

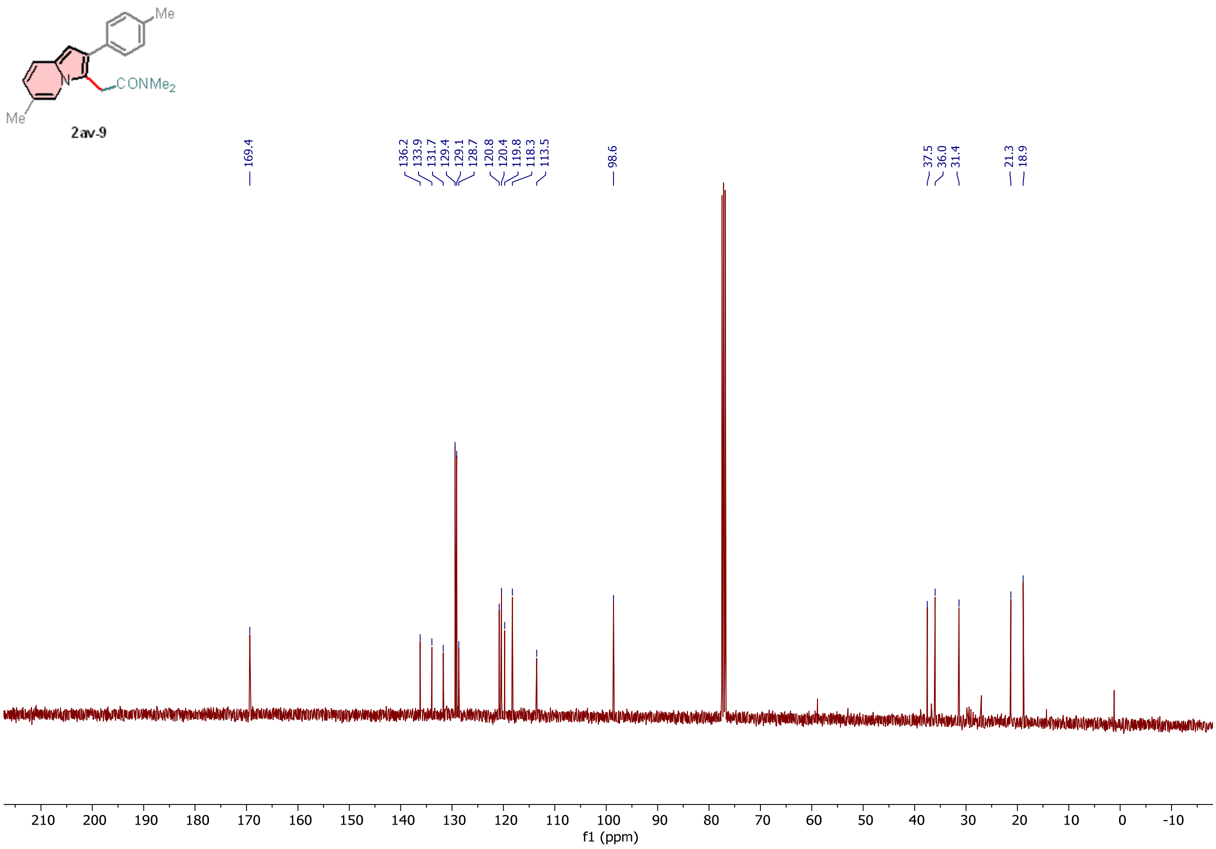

$^{13}C\{^{1}H\}$ NMR (100 MHz, $CDCl_3$) of **2av-9**

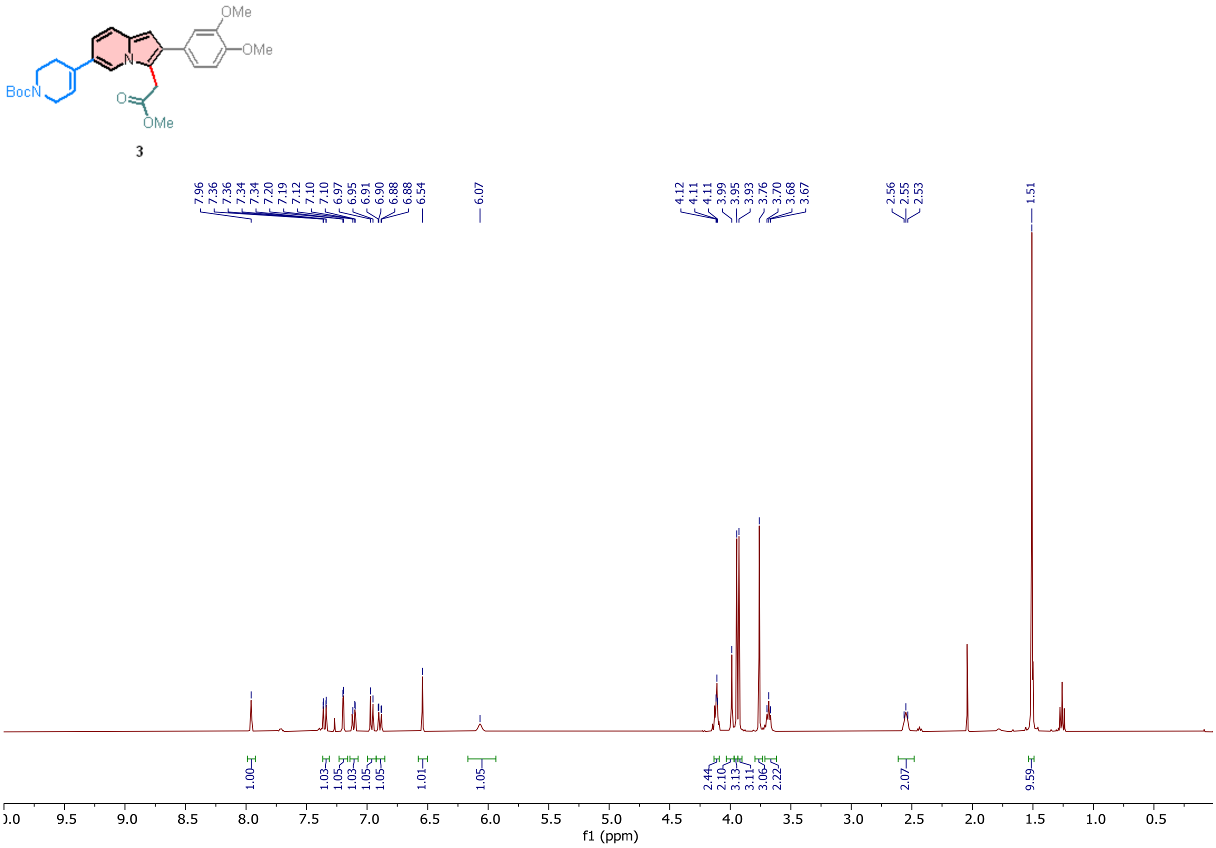

^{1}H NMR (400 MHz, $CDCl_3$) of **3**.

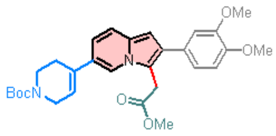

3

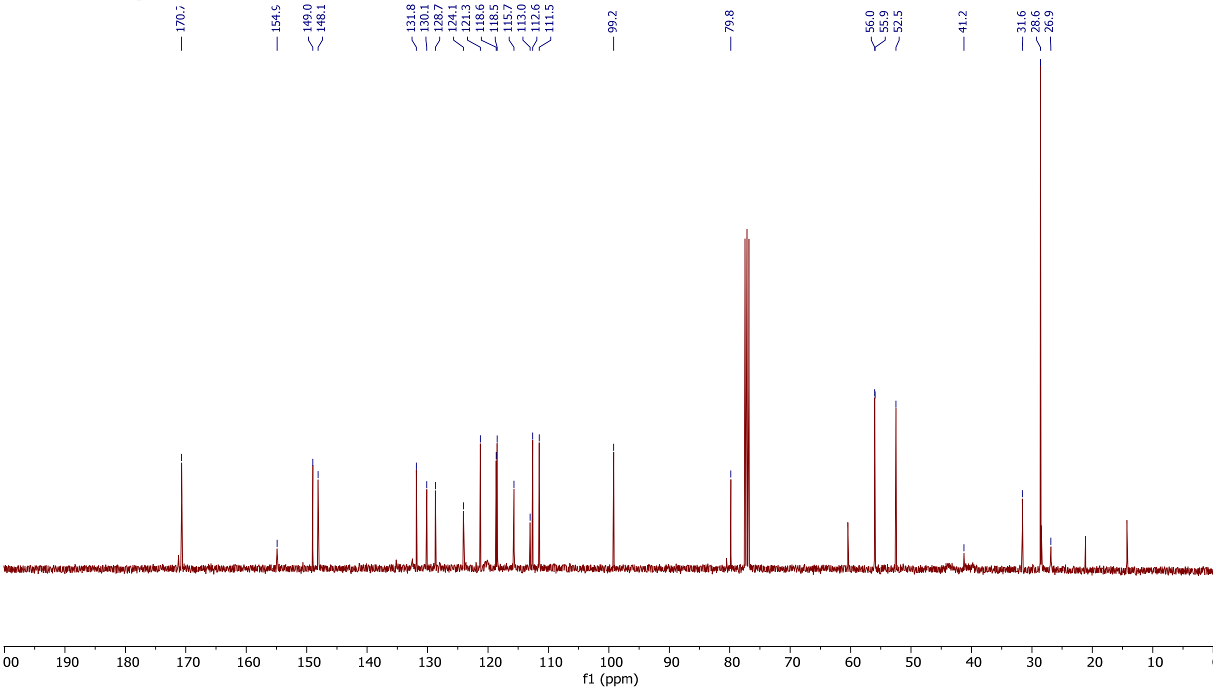

$^{13}C\{^{1}H\}$ NMR (100 MHz, $CDCl_3$) of **3**.

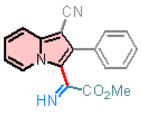

4a

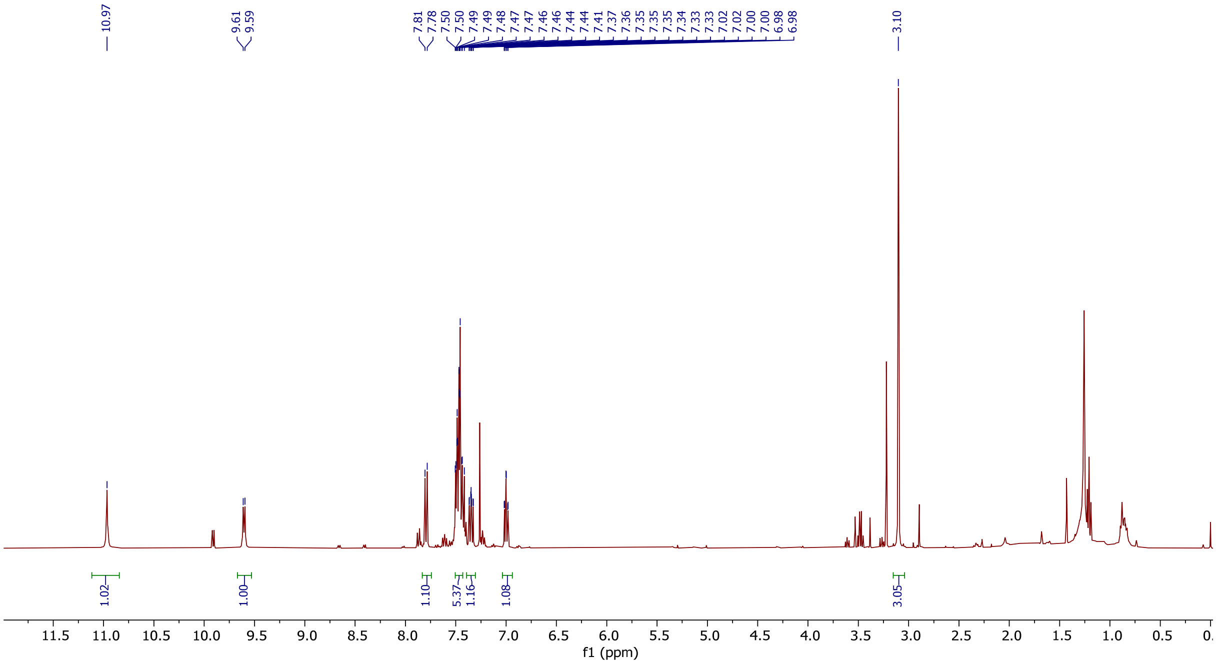

^{1}H NMR (400 MHz, $CDCl_3$) of **4a.**

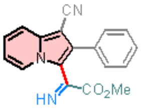

4a

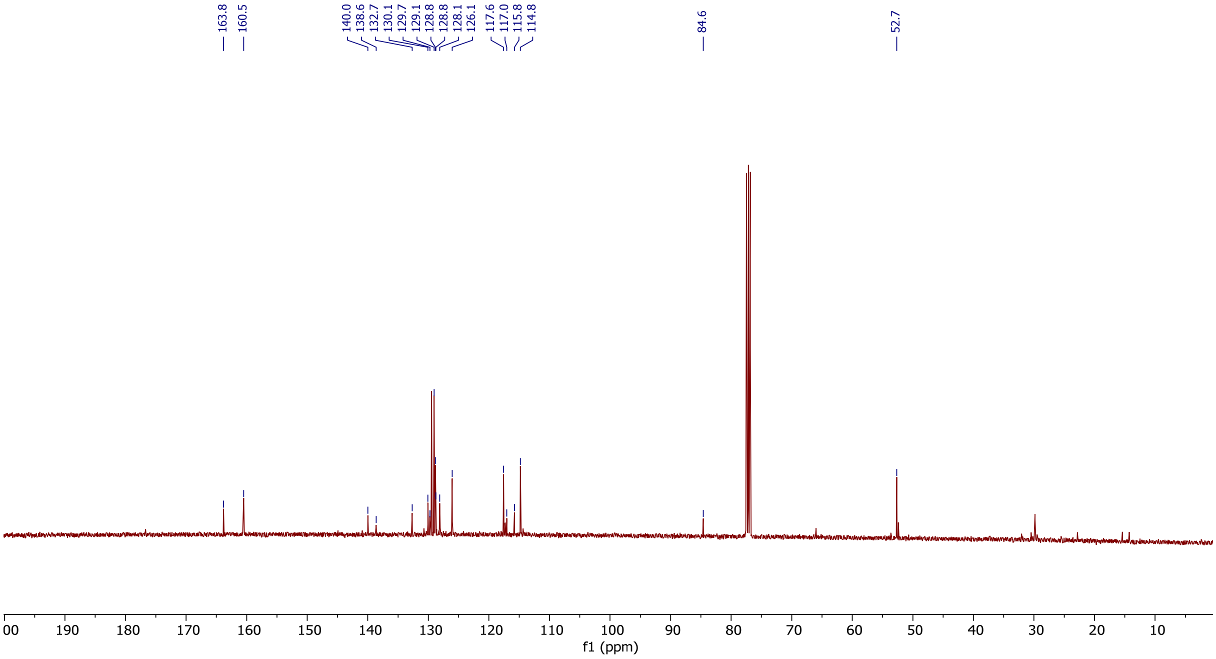

$^{13}C\{^1H\}$ NMR (100 MHz, $CDCl_3$) of **4a**.

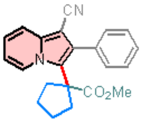

4b

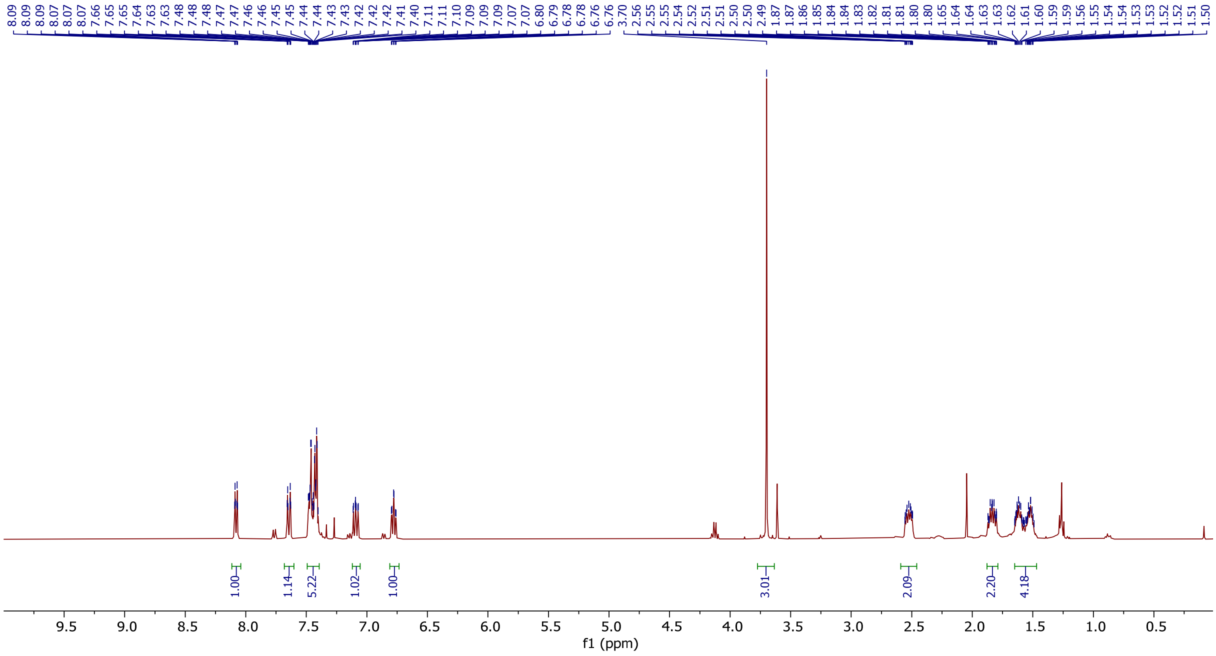

^1H NMR (400 MHz, $CDCl_3$) of **4b.**

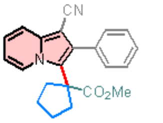

4b

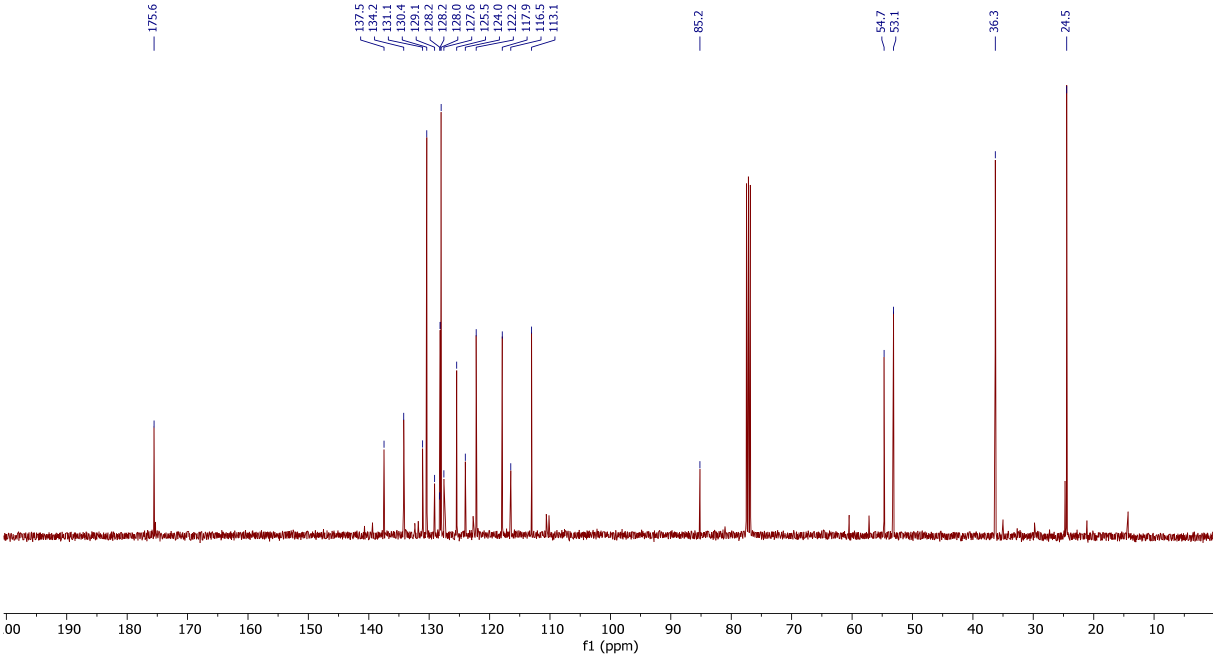

$^{13}C\{^{1}H\}$ NMR (100 MHz, $CDCl_3$) of **4b**.

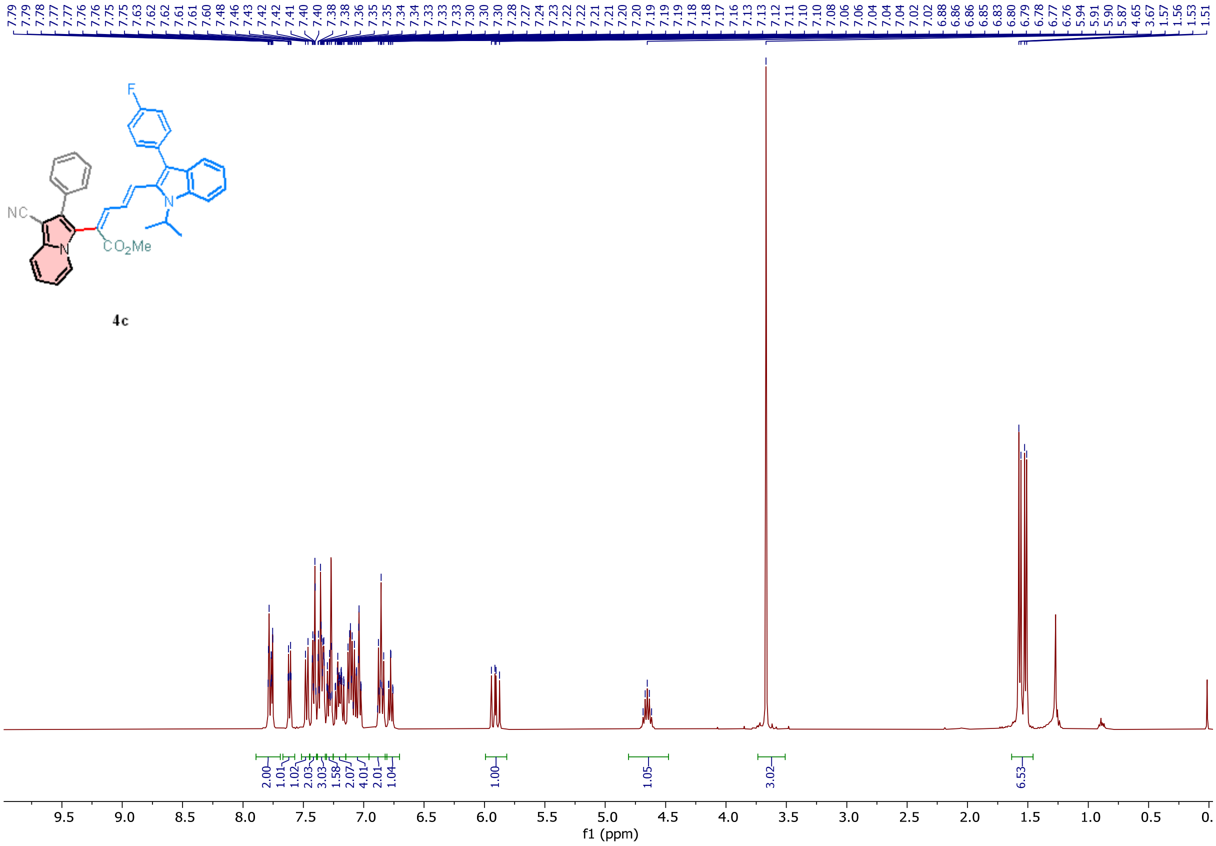

^{1}H NMR (400 MHz, $CDCl_3$) of **4c**.

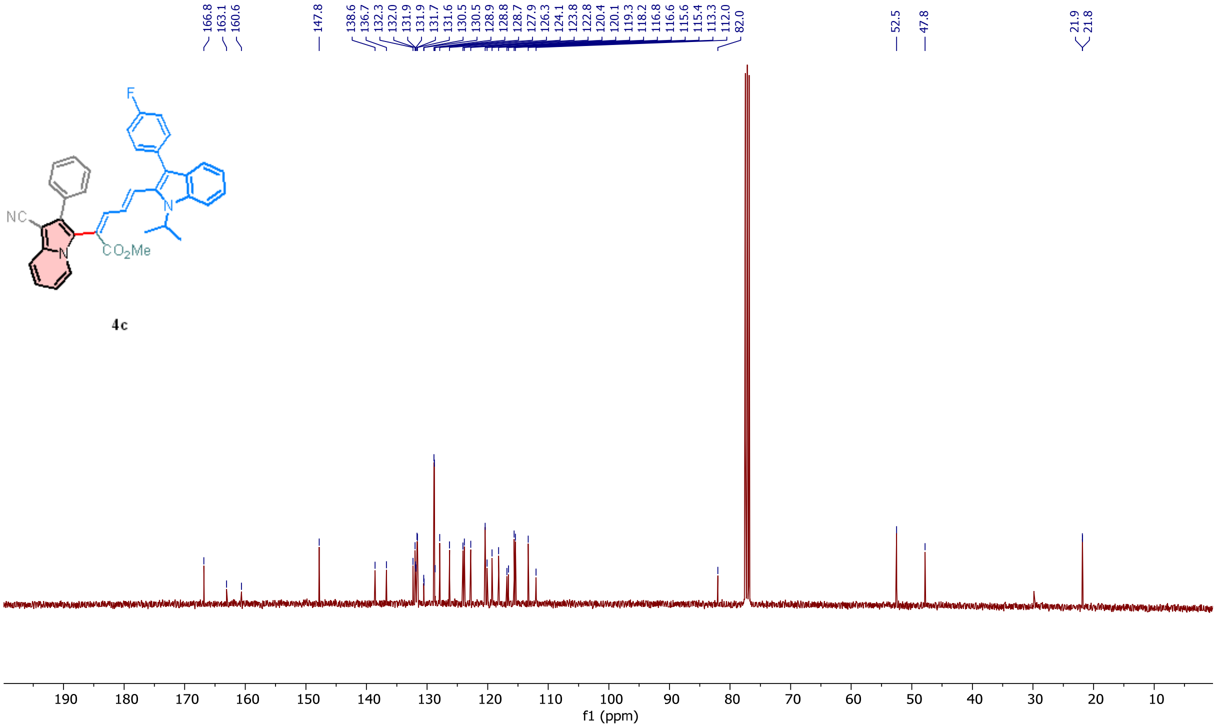

$^{13}C\{^1H\}$ NMR (100 MHz, $CDCl_3$) of **4c**.

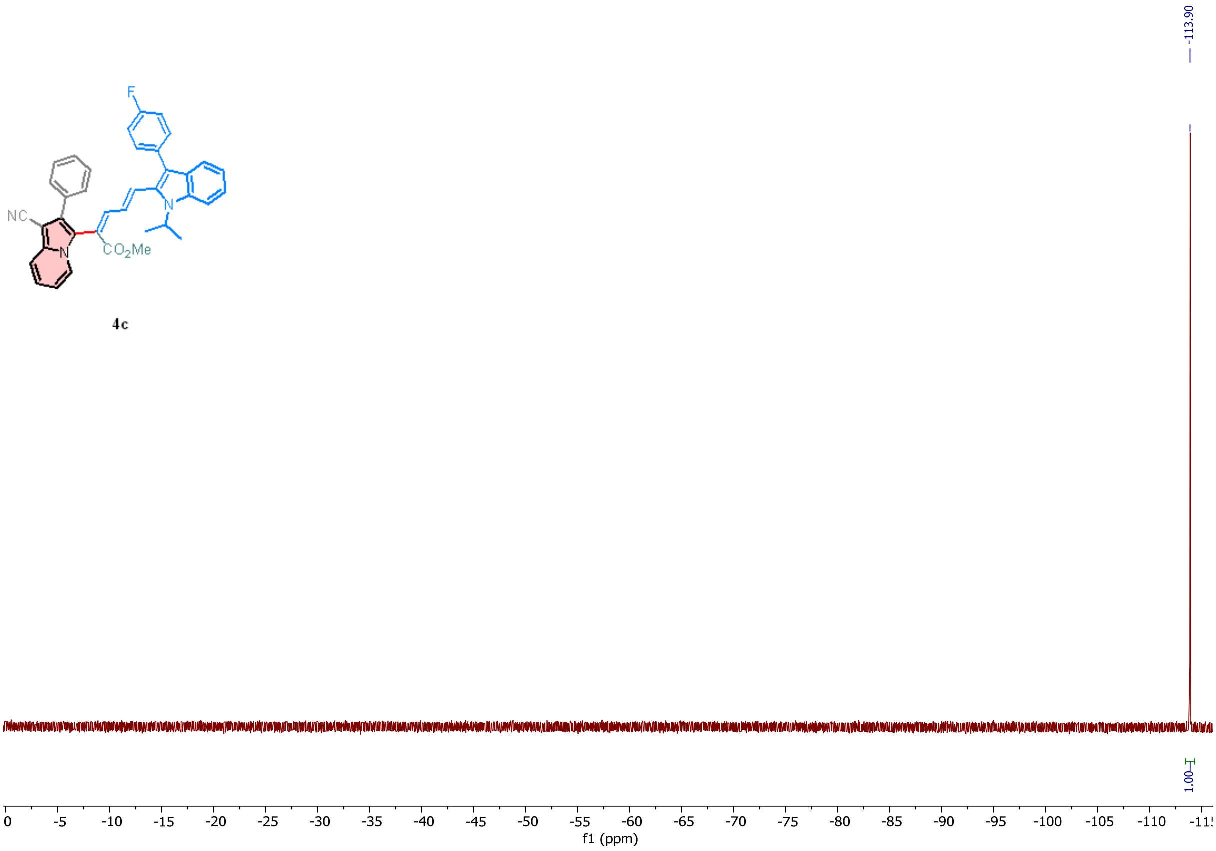

$^{19}F\{^1H\}$ NMR (377 MHz, $CDCl_3$) of **4c**.

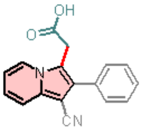

4d

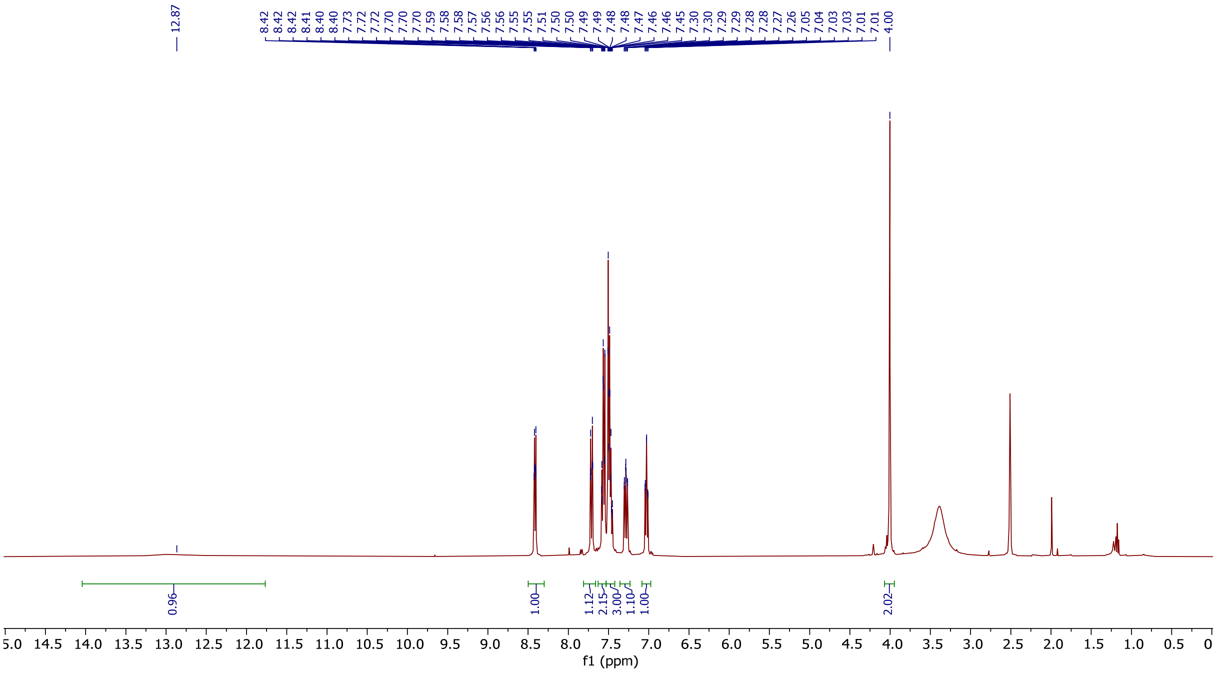

^{1}H NMR (400 MHz, DMSO-d^6) of **4d**.

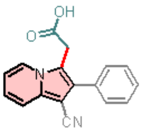

4d

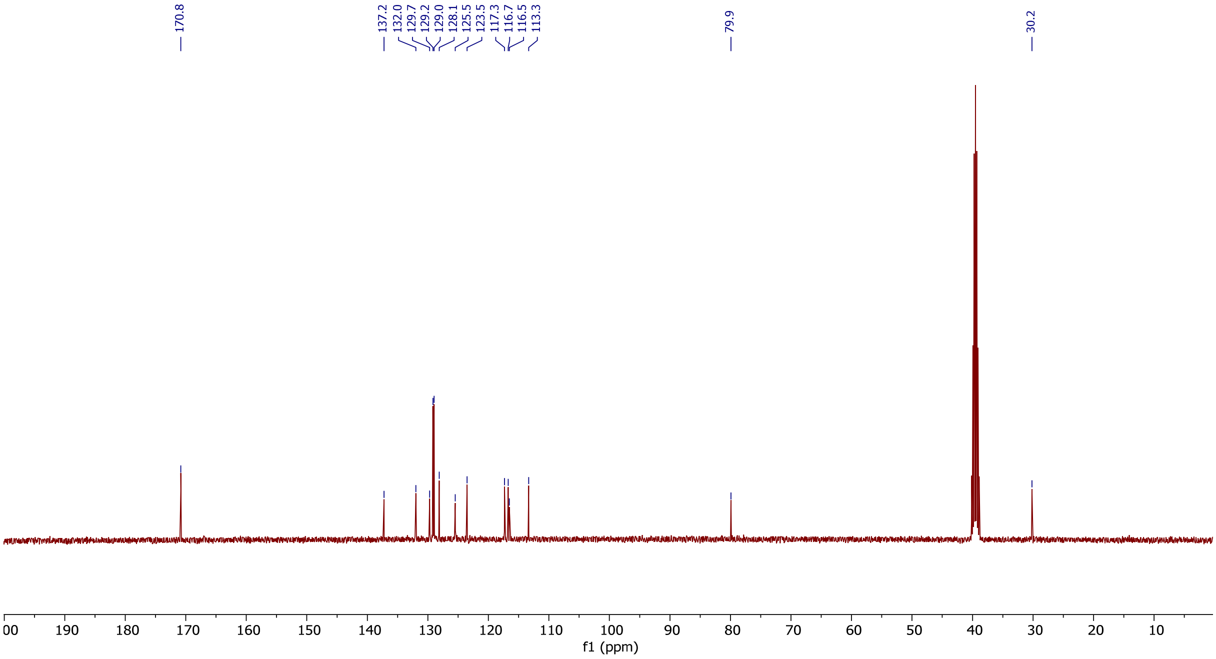

^{13}C{^{1}H} NMR (100 MHz, DMSO-d^6) of **4d**.

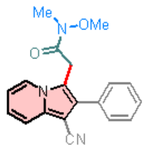

5a

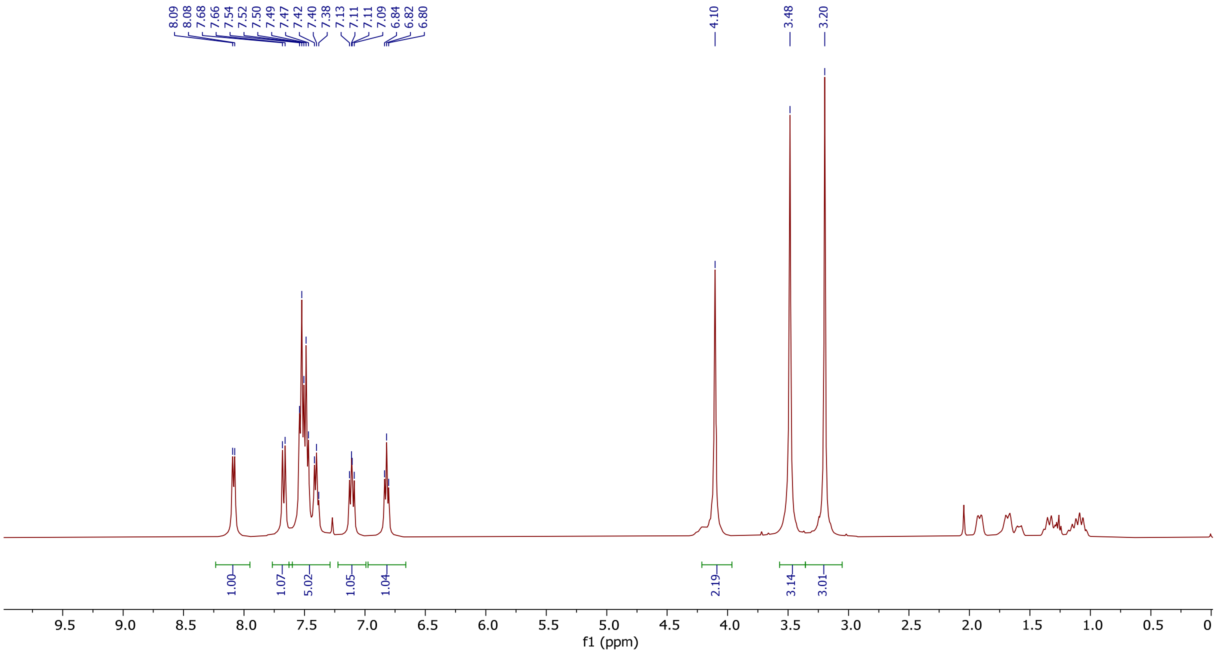

^{1}H NMR (400 MHz, $CDCl_3$) of **5a**.

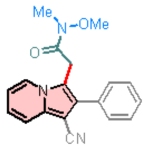

5a

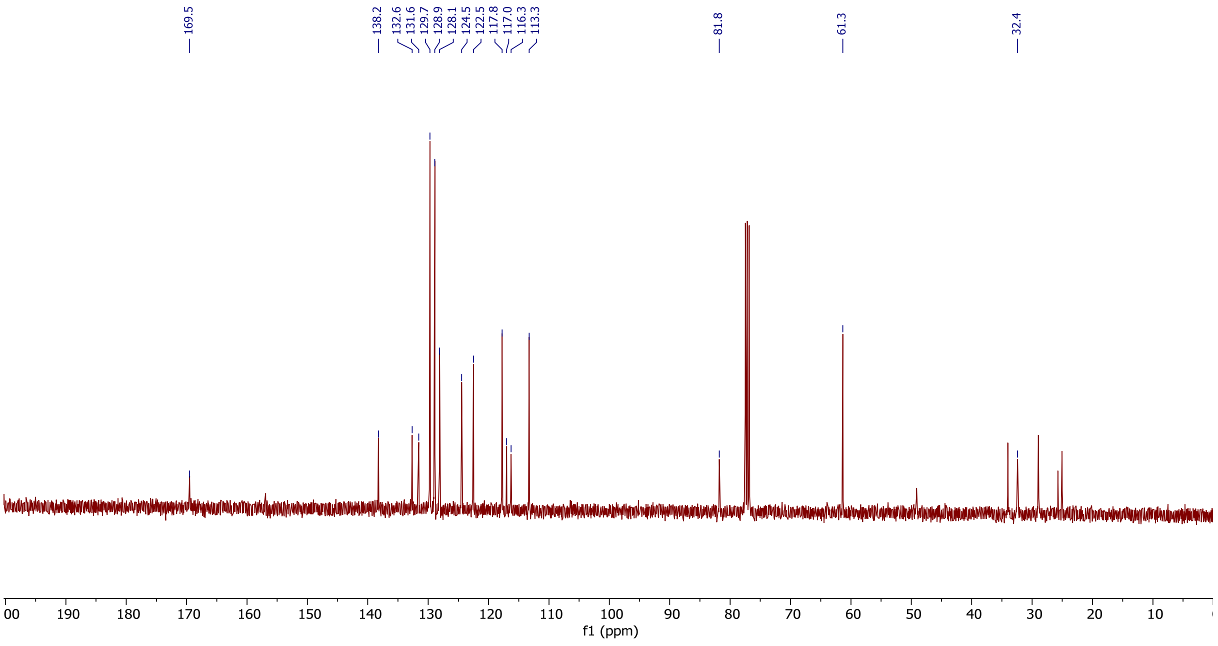

^{13}C{^{1}H} NMR (100 MHz, $CDCl_3$) of **5a**.

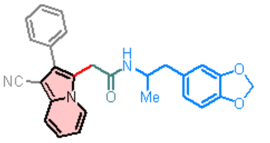

5b

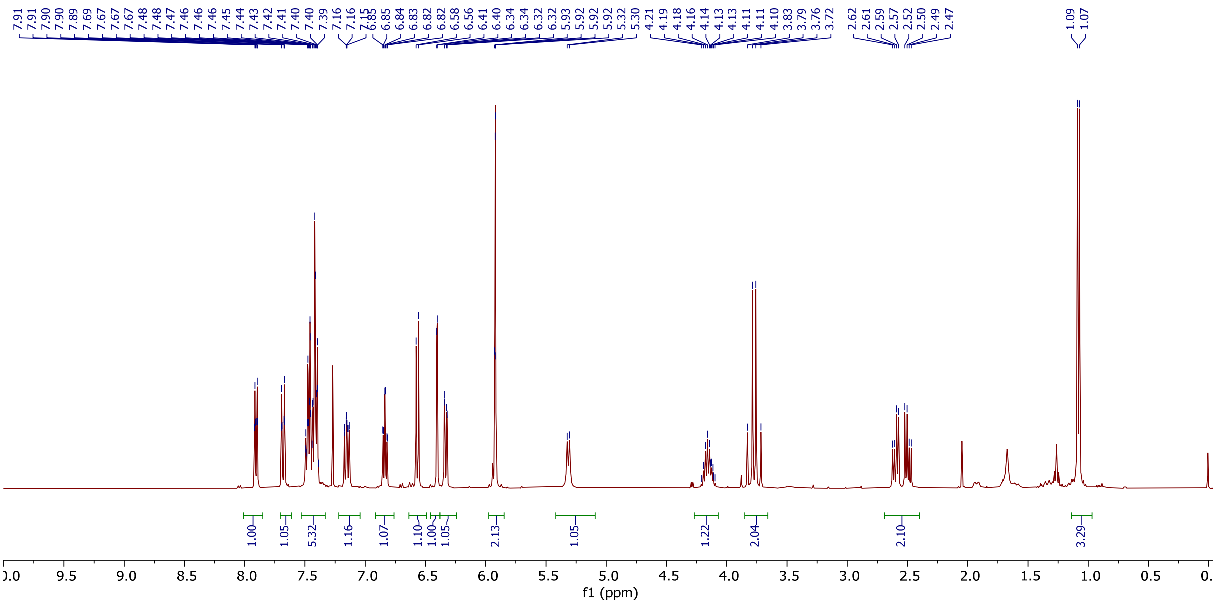

^{1}H NMR (400 MHz, $CDCl_3$) of **5b**.

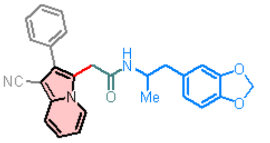

5b

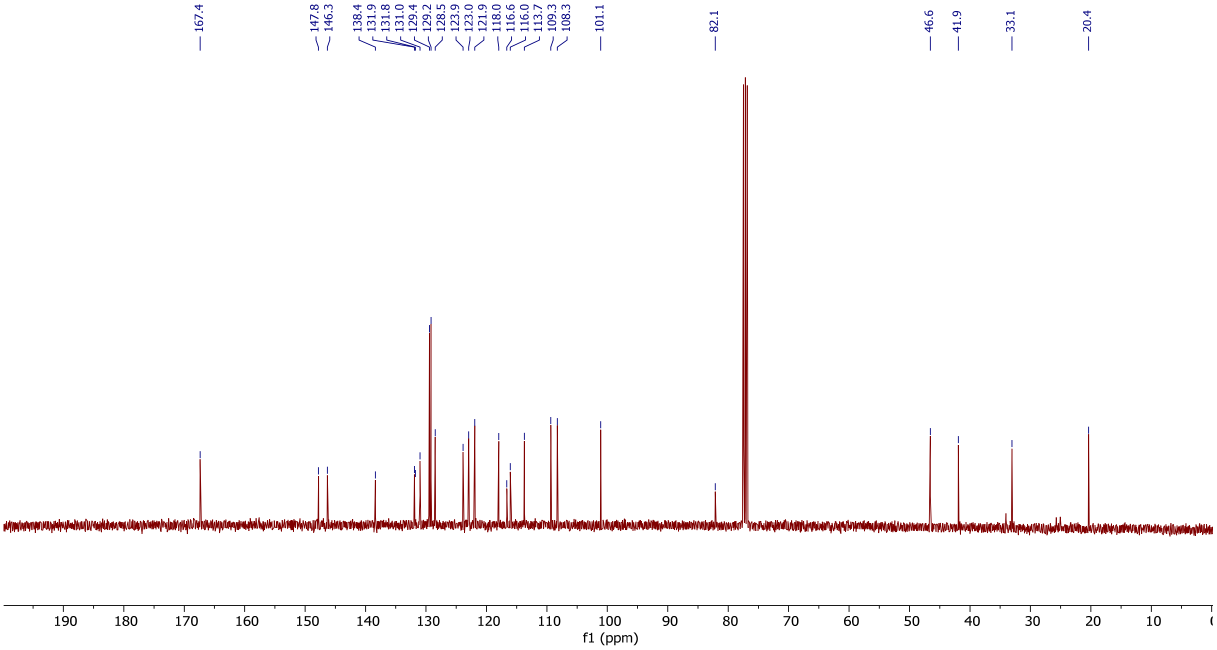

$^{13}C\{^{1}H\}$ NMR (100 MHz, $CDCl_3$) of **5b**.

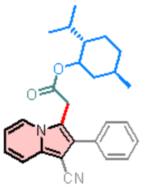

5c

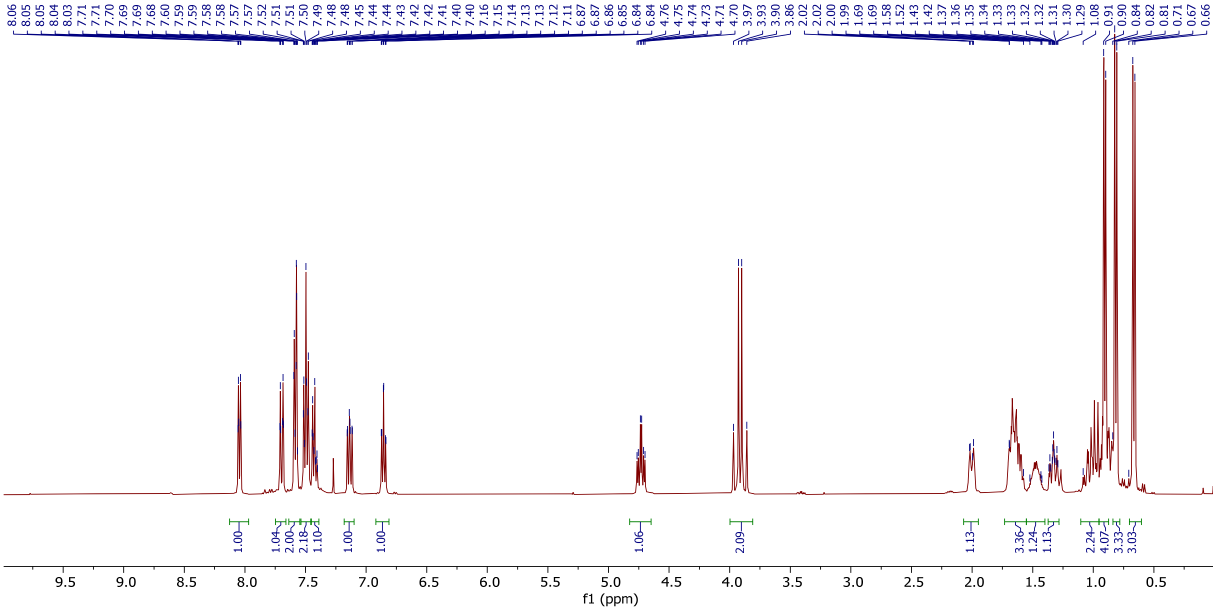

^{1}H NMR (400 MHz, $CDCl_3$) of **5c**.

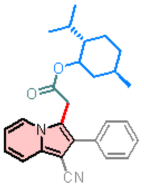

5c

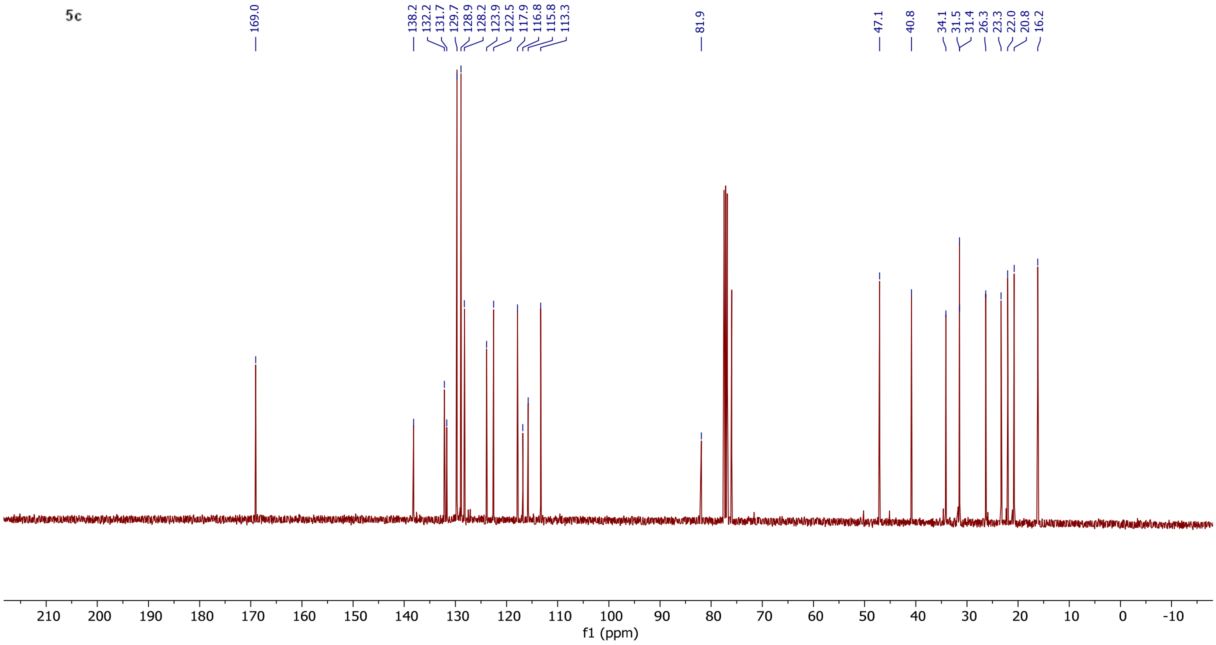

^{13}C{^{1}H} NMR (100 MHz, $CDCl_3$) of **5c**.

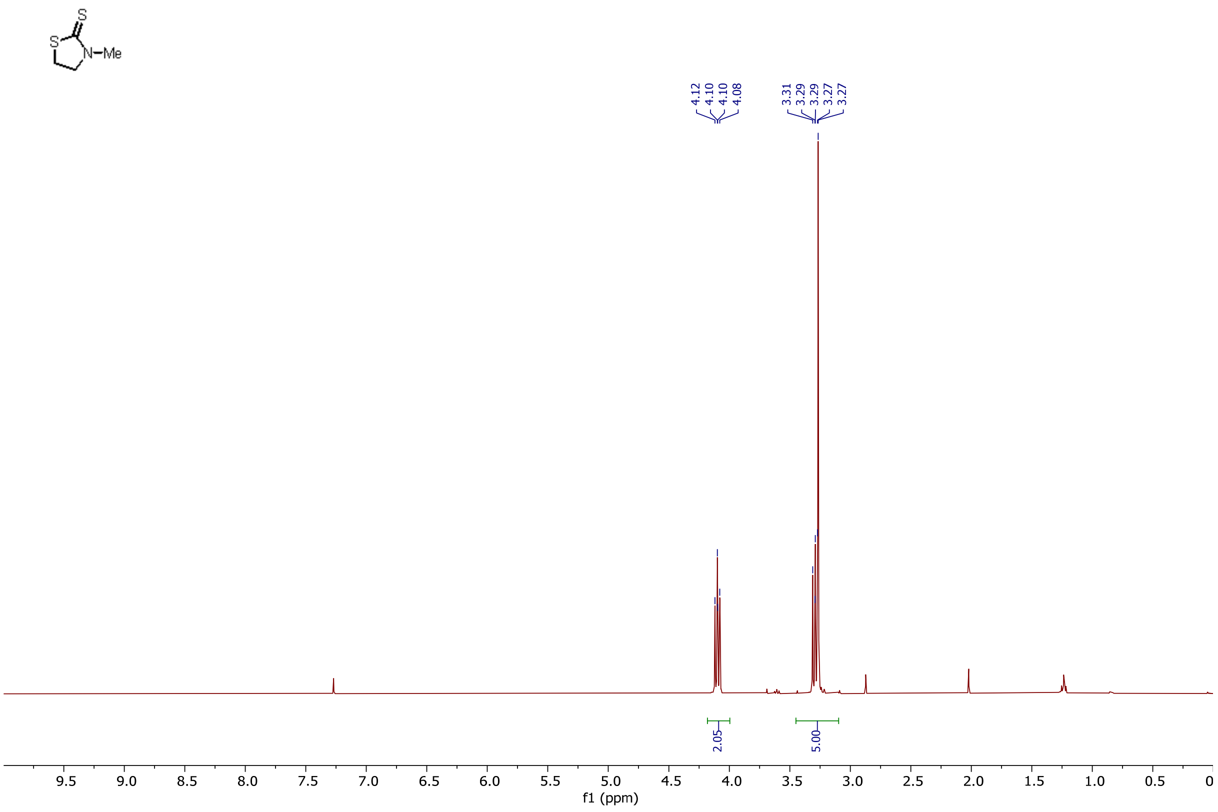

^{1}H NMR (400 MHz, $CDCl_3$) of 3-methylthiazolidine-2-thione.

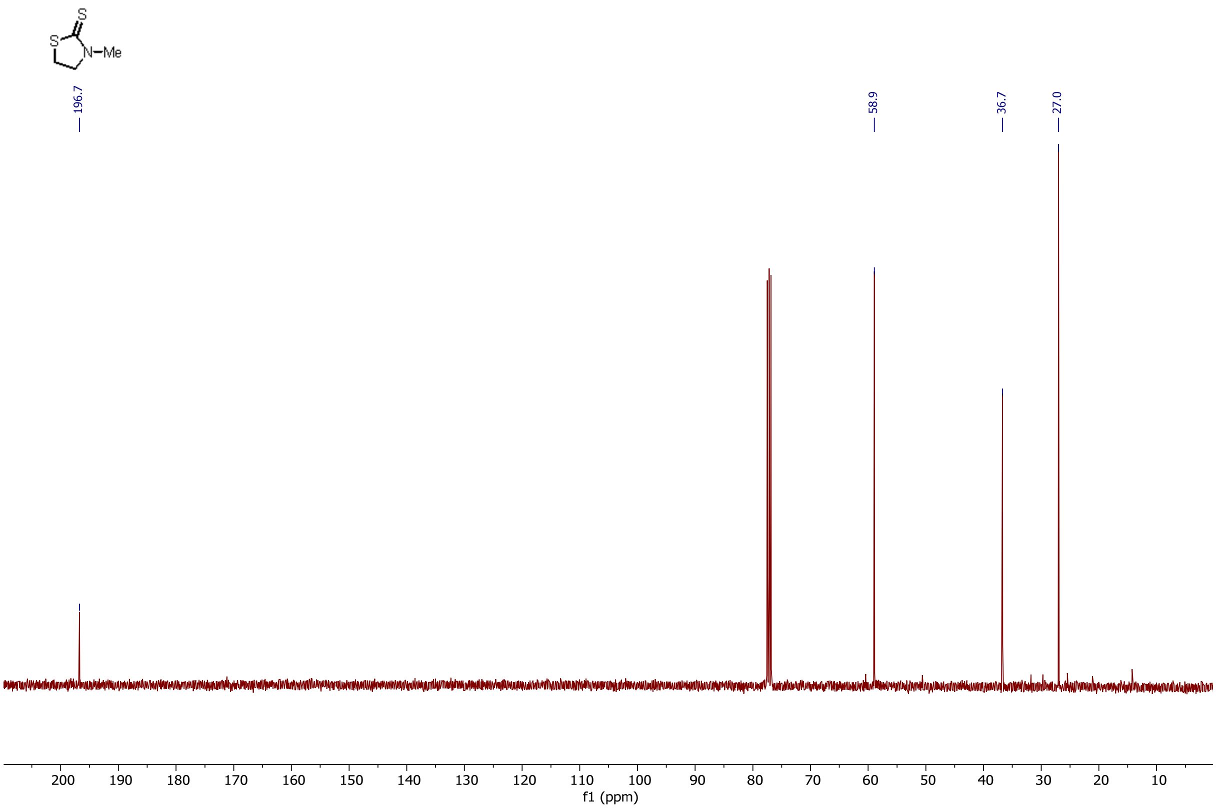

$^{13}C\{^{1}H\}$ NMR (100 MHz, $CDCl_3$) of 3-methylthiazolidine-2-thione.

12. References

1 D. R. Bragg and D. G. Wibberley, *J. Chem. Soc.*, 1963, 3277.
2 B. Li, Z. Chen, H. Cao and H. Zhao, *Org. Lett.*, 2018, **20**, 3291–3295.
3 W. Kim, H. Y. Kim and K. Oh, *J. Org. Chem.*, 2021, **86**, 15973–15991.
4 D. K. Singh, S. Kim, J. H. Lee, N. K. Lee, J. Kim, J. Lee and I. Kim, *Journal of Heterocyclic Chemistry*, 2020, **57**, 3018–3028.
5 L. Zhang, F. Liang, L. Sun, Y. Hu and H. Hu, *Synthesis*, 2000, **2000**, 1733–1737.
6 J.-L. Wang, G.-Y. Wu, J.-N. Luo, J.-L. Liu and C.-X. Zhuo, *J. Am. Chem. Soc.*, 2024, **146**, 5605–5613.
7 G. I. D. Cooper, I. Saha, J. Newman, R. H. Shin and P. G. Harran, *J. Org. Chem.*, 2024, **89**, 14665–14672.
8 Y. Tian, X.-X. Fu, R.-M. Cheng, J. Feng, M.-H. Shen, C.-F. Zhu and H.-D. Xu, *European Journal of Organic Chemistry*, 2024, **27**, e202400344.
9 A. A. Zemtsov, S. S. Ashirbaev, V. V. Levin, V. A. Kokorekin, A. A. Korlyukov and A. D. Dilman, *J. Org. Chem.*, 2019, **84**, 15745–15753.
10 A. Baron, C. Herrero, A. Quaranta, M.-F. Charlot, W. Leibl, B. Vauzeilles and A. Aukauloo, *Chem. Commun.*, 2011, **47**, 11011.
11 M. R. Norris, J. J. Concepcion, C. R. K. Glasson, Z. Fang, A. M. Lapides, D. L. Ashford, J. L. Templeton and T. J. Meyer, *Inorg. Chem.*, 2013, **52**, 12492–12501.
12 K. K. Stefanoni, M. Schmitz, J. Treuheit, C. Kerzig and R. Wilhelm, *J. Org. Chem.*, 2025, **90**, 6491–6503.
13 J. Grolleau, P. Frère and F. Gohier, *Synthesis*, 2015, **47**, 3901–3906.
14 B. Mühling, S. Theisinger and H. Meier, *Synthesis*, 2006, **2006**, 1009–1015.
15 O. Lavastre, L. Ollivier, P. H. Dixneuf and S. Sibandhit, *Tetrahedron*, 1996, **52**, 5495–5504.
